# Retrotransposons evolution and impact on lncRNA and protein coding genes in pigs

**DOI:** 10.1186/s13100-019-0161-8

**Published:** 2019-05-06

**Authors:** Cai Chen, Wei Wang, Xiaoyan Wang, Dan Shen, Saisai Wang, Yali Wang, Bo Gao, Klaus Wimmers, Jiude Mao, Kui Li, Chengyi Song

**Affiliations:** 1 Institute of Animal Mobilome and Genome, College of Animal Science & Technology, Yangzhou University, Yangzhou, 225009 Jiangsu China; 2 Leibniz Institute for Farm Animal Biology (FBN), 18196 Dummerstorf, Germany; 3 Life Science Center, University of Missouri, Columbia, MO 65211 USA; 4 Institute of Animal Science, Chinese Academy of Agricultural Sciences, Beijing, China

**Keywords:** Pig genome, Retrotransposon evolution, Gene overlapping, Retrotransposition activity, Promoter activity, Distribution bias

## Abstract

**Background:**

Retrotransposons are the major determinants of genome sizes and they have shaped both genes and genomes in mammalian organisms, but their overall activity, diversity, and evolution dynamics, particularly their impact on protein coding and lncRNA genes in pigs remain largely unknown.

**Results:**

In the present study, we performed de novo detection of retrotransposons in pigs by using multiple pipelines, four distinct families of pig-specific L1 s classified into 51 distinct subfamilies and representing four evolution models and three expansion waves of pig-specific SINEs represented by three distinct families were identified. ERVs were classified into 18 families and found two most “modern” subfamilies in the pig genome. The transposition activity of pig L1 was verified by experiment, the sense and antisense promoter activities of young L1 5′UTRs and ERV LTRs and expression profiles of young retrotransposons in multiple tissues and cell lines were also validated. Furthermore, retrotransposons had an extensive impact on lncRNA and protein coding genes at both the genomic and transcriptomic levels. Most protein coding and lncRNA (> 80%) genes contained retrotransposon insertions, and about half of protein coding genes (44.30%) and one-fourth (24.13%) of lncRNA genes contained the youngest retrotransposon insertions. Nearly half of protein coding genes (43.78%) could generate chimeric transcripts with retrotransposons. Significant distribution bias of retrotransposon composition, location, and orientation in lncRNA and protein coding genes, and their transcripts, were observed.

**Conclusions:**

In the current study, we characterized the classification and evolution profile of retrotransposons in pigs, experimentally proved the transposition activity of the young pig L1 subfamily, characterized the sense and antisense expression profiles and promoter activities of young retrotransposons, and investigated their impact on lncRNA and protein coding genes by defining the mobilome landscapes at the genomic and transcriptomic levels. These findings help provide a better understanding of retrotransposon evolution in mammal and their impact on the genome and transcriptome.

**Electronic supplementary material:**

The online version of this article (10.1186/s13100-019-0161-8) contains supplementary material, which is available to authorized users.

## Background

Transposable elements (TEs), also referred to as the mobilome, are DNA sequences that have the ability to integrate into the genome at a new site within their cell of origin. They can be divided into retrotransposons and DNA transposons based on their diverse structures and transposition mechanisms. Retrotransposons consists of short interspersed elements (SINEs), long interspersed elements (LINEs), and long terminal repeats (LTRs), including endogenous retroviruses (ERVs), all of which propagate by the reverse transcription (RT) of an RNA intermediate [1, 2]. TEs were once viewed merely as junk DNA and selfish DNA parasites. However, genome-scale studies over the past several decades have shown that TEs and their recognizable remnants span both prokaryote and eukaryote organisms, are major determinants of genome sizes [3–5], and account for about half of the human genome [6]; they even make up 85% of the maize genome [7]. TEs have shaped both genes and the entire genome and play a key role in genome function, speciation, and diversity [8, 9]. TEs also contribute substantially to the evolution of the genome at the DNA level, and they can undergo “molecular domestication” [10, 11]; at least 50 genes have been domesticated from mobile elements in the human genome [12]. Chimeric transcripts between TEs and protein coding genes tend to be common [13, 14]. TE insertions can also induce diverse structural variations of the genome [9, 15]. Furthermore, TEs contribute substantially to the evolution of many genes at the transcriptional level by acting as alternative promoters, enhancers, splice sites, or polyadenylation signals [16, 17], or the transcription factor binding sites for these genes [18]. It has also been suggested that a majority of primate-specific regulatory sequences are derived from TEs [19]. The epigenetic landscape can be altered by TE insertions [20]. Evolution of the sperm methylome of primates is associated with Alu and SVA retrotransposon insertions [21]. Methylation levels of retrotransposons are associated with carcinogenesis and metastasis [22–24]. In addition, growing evidence shows a close association of TEs with non-coding RNAs (ncRNAs), and a significant number of small ncRNAs originate from TEs [25]. Furthermore, TEs tend to enrich in the lncRNAs of human, mice, and zebrafish [26, 27], and retrotransposons make a strong contribution to lncRNA evolution, structure, and function in mammalian organisms [28].

Retrotransposons occupy one-third to half of the mammal genomes, which are dominated by LINEs and SINEs, followed by LTR retrotransposons [8]. The LINE family is the most successful TE family in both the common ancestors and extant species of mammals, and account for 20.42% in humans, 19.20% in mice, 19.54% in pigs, 21.21% in platypuses, and 28.60% in tammars, while SINEs, known as the partner of LINEs that require LINEs for their transposition, account for 13.14% in humans, 8.22% in mice, 13.08% in pigs, 21.53% in platypuses, and 11.70% in tammars. LTRs are the third major type of interspersed repeats in mammals, accounting for 8.29% in humans, 9.87% in mice, 4.48% in pigs, 0.12% in platypuses, and 3.90% in tammars [6, 29–32]. In addition, unusual evolution dynamics of L1 s in mammals are observed, with a single family of replicative dominant subfamilies evolved in one period, then being replaced by a more recently evolved family [33]. Studies in humans and mice also revealed the diversification evolution of L1 s, and the coexistence of multiple L1 subfamilies with different promoters in young and ancient families [34–36]. Little is known about the factors that determine the burst and decline of SINEs, but, clearly, SINE amplification is dependent on LINE activity, and activity correlation is observed for many SINE/LINE partners; for example, mammalian-wide interspersed repeat (MIR) (Ther-1) and L2 in humans and mice [6, 29], MEG and L1 in fruit bats [37, 38], and Alu and L1 subfamilies in humans [39]. Although most retrotransposons are no longer active in mammals, research has shown that most mammal genomes contain at least one family of actively accumulating retrotransposons [8, 40]. Examples include L1/LINEs in most mammals [41], RTE/LINEs in ruminants and marsupials [42], with the exception of LINEs in the megabat family, where the activity of L1 went extinct 24 million years ago [43], while ERVs/LTR in rodent genomes are believed to be active [29, 44]. The retrotransposition activities of L1 s and SINEs in humans and mice, including human L1 (L1H_S_), mouse L1 (T_F_ and G_F_), and both human SINE (AluYa5/8 and AluYb8/9) and mouse SINE (SINE B1 and SINE B2), have been verified experimentally [41].

Despite the prevalence of retrotransposons in mammalian genomes and their biological relevance, relatively few pig retrotransposons have been reported. Initially, the TE coverages in Duroc and Wuzhishan pig genomes have been well annotated in previous studies [30, 45]. The divergence distribution and phylogenetic analysis of retrotransposons in pigs revealed that the main repeating element groups are LINEs and SINEs, and only a single family of each is deduced to be putatively active [30, 45], and two complete pig ERVs were identified in Wuzhishan pig genome, which may carry the risk of pathogen transmission to human in xenotransplantation [45]. Whereas the overall activity, diversity, and evolution of retrotransposons, particularly the diversity at the family, and subfamily levels, and the evolution dynamics of the dominate L1, SINE, and ERV families, in the pig genome remain largely unknown. In addition, retrotransposon involvement in the structural and functional evolution of genes and genomes, as well as their impact on the transcriptome in pig, remain completely unknown.

In this study, we performed de novo detection of retrotransposons in pigs using multiple pipelines. We characterized the classification of LINEs, SINEs, and ERVs at the family and subfamily levels, highlighted the evolution dynamics of these families and subfamilies, and then determined the retrotransposition activity of L1 and the sense and antisense promoter activities and expression profiles of young retrotransposon subfamilies. Furthermore, we investigated the intersection between retrotransposons and host genes, including protein-encoding and lncRNA genes, as well as the impact of retrotransposons on the transcriptome. Overall, this study revealed the retrotransposon landscape and their evolution profiles in the pig genome, domesticated the retrotransposition activities of young L1 subfamilies, and defined the sense and antisense expression profiles and promoter activities of young retrotransposon subfamilies. Our data support the hypothesis that most copies of retrotransposons are fossils in the pig genome, but a few retrotransposon copies of L1 s, SINEs, and ERVs may still be active. Our analysis also reveals that the majority of protein coding and lncRNA genes contain retrotransposon insertions, and retrotransposons tend to be enriched in lncRNA, with nearly half of protein coding genes generating chimeric transcripts with retrotransposons.

## Results

### Four distinct families of pig-specific L1 s representing four evolution models

**Fig. 1:**
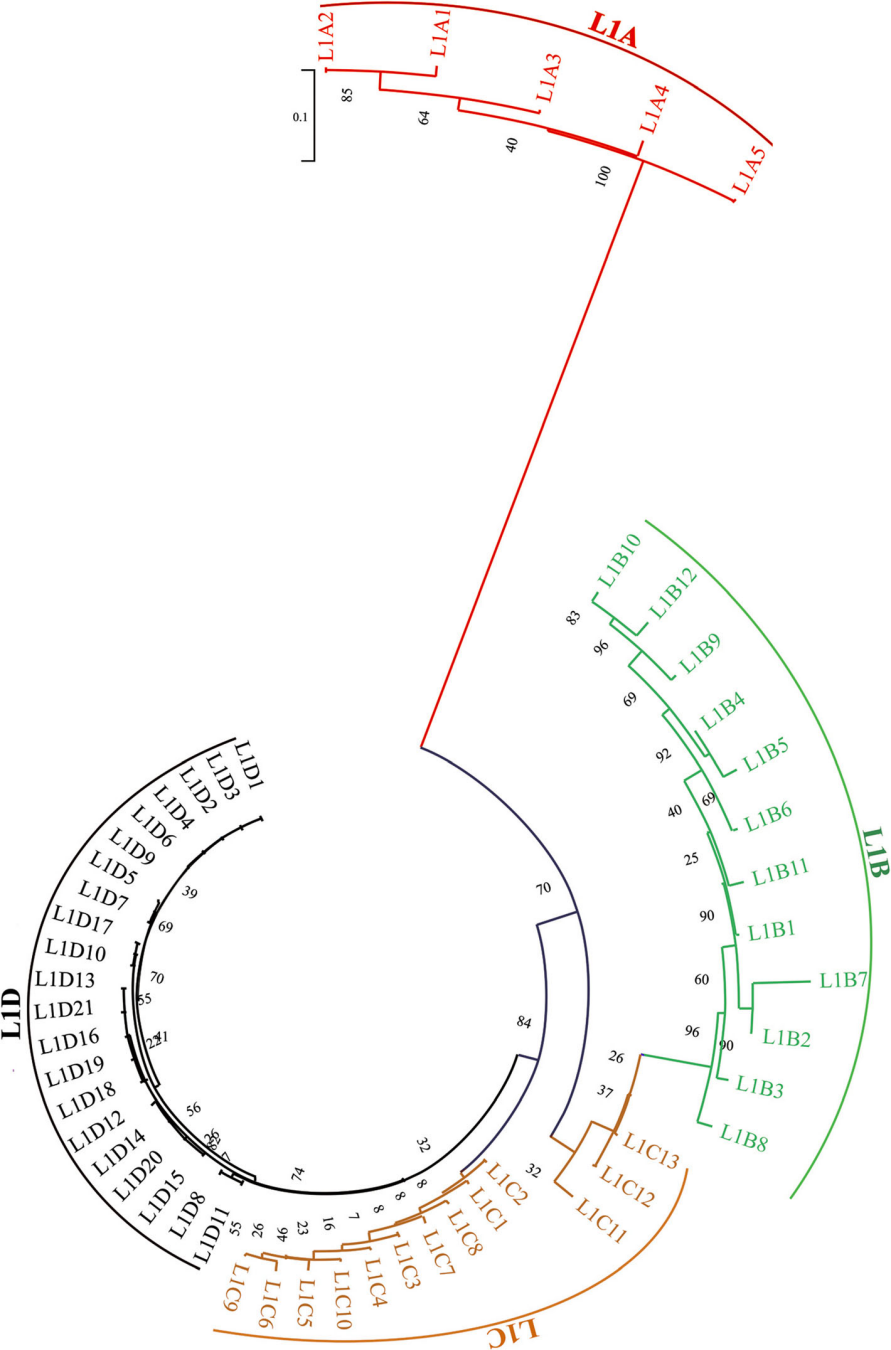
Neighbor-joining polygenic tree of pig L1 based on the 5′UTR and classified L1 s into four distinct families (L1A, L1B, L1C, and L1D)

**Fig. 2:**
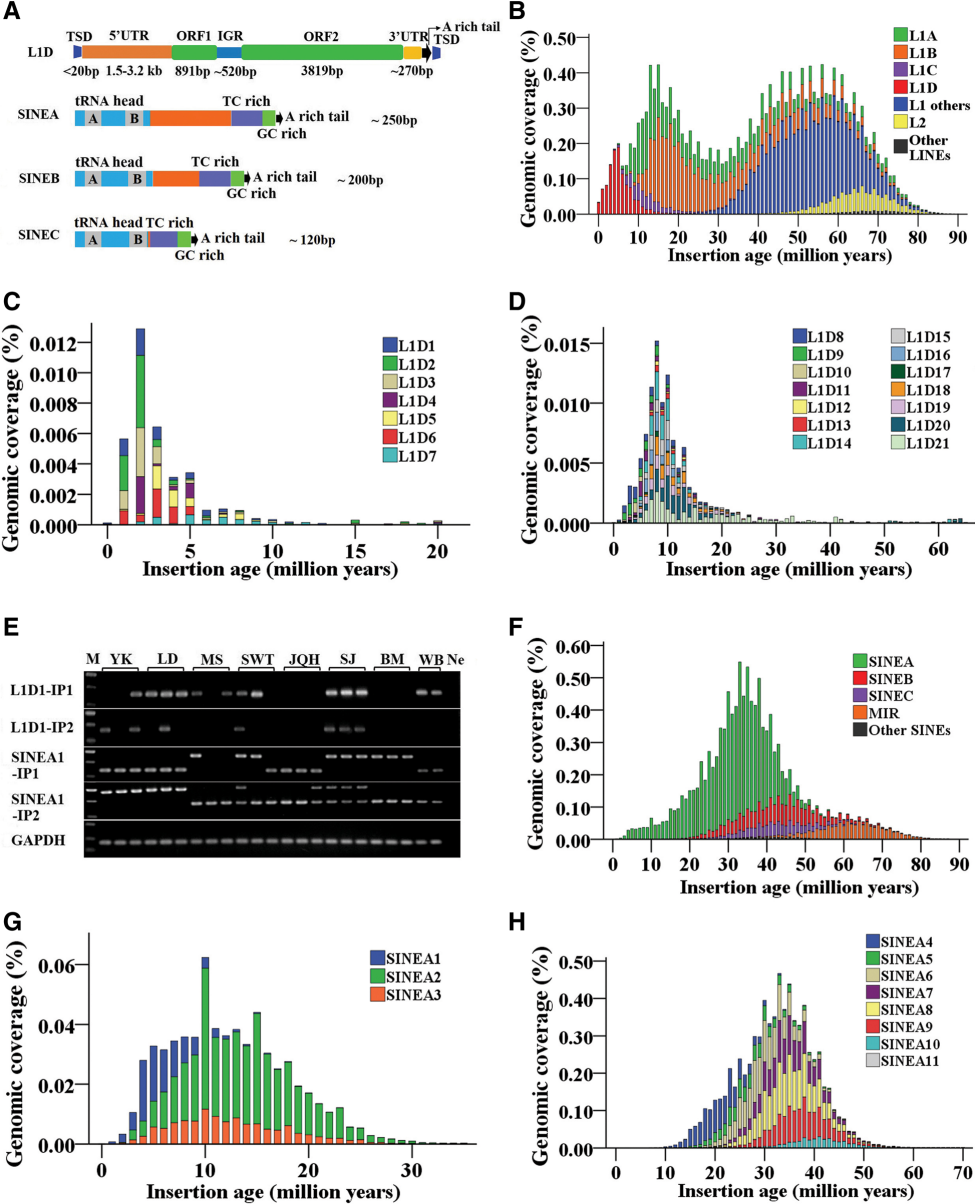
Evolution of L1 s and SINEs in the pig genome. **a** Structural schematics of the putatively active L1 s and pig-specific SINE families (SINEA, SINEB, and SINEC). **b** Age distribution of pig-specific L1 families. **c** and **d** Age distribution across the subfamilies (L1D1–21) of the youngest L1 family (L1D). **e** Insertion polymorphism (IP) detection of the youngest L1 (L1D1) and SINE (SINEA1) subfamilies by PCR. Breed name abbreviations: Meishan (MS), Shawutou (SWT), and Jiangquhai (JQH) pigs are native Chinese pig breeds from Jiangsu Province; the Sujiang (SJ) pig is a newly established breed based on the Duroc and Jiangquhai bloodlines; Bama (BM) pigs are miniature pigs from Guangxi Province; the wild boar (WB) was from Anhui Province; and the Landrace (LD) and Yorkshire (YK) pigs were from a breeding farm in Anhui Province. Ne, negative control without DNA. Two transposon loci in each of the youngest transposon subfamilies were selected for insertion polymorphism (IP) detection and labeled as IP1 and IP2. If an individual contains SINE insertion at SINE-IP1 or SINE-IP2 site, the band size would be 629 or 676 bp, respectively, and if no SINE insertion, the band would be 335 or 382 bp. The three bands showed in the M (marker) lane are 750 bp, 500 bp and 250 bp from top to bottom. **f**) Age distribution of pig-specific SINE families. **g** and **h** Age distribution across the subfamilies (SINEA1–11) of the youngest SINE family (SINEA). The *x*-axis represents the insertion age (Million years ago, Mya), and the *y*-axis represents the percentage of the genome composed of retrotransposon families/subfamilies (%) in Fig. **b**, **c**, **f**, and **g**

A total of 4154 L1 elements were identified by MGEScan-non-LTR, and they were aligned against the pig genome by Blat with an extension of 2500 bp of 5′UTR and 200 bp of 3′UTR to get the full lengths of the elements. In addition, 4495 L1 elements were downloaded from L1Base database and merged with these L1 s, and the redundancy was removed. Finally, we obtained 5937 L1 elements with unique position in the pig genome. These L1 s were classified into 51 distinct subfamilies, including one subfamily (L1_B-SS) deposited in Repbase, according to their 5′UTR sequences, and consensus sequences were derived for each. Two subfamilies (HAL1_Ssc and L1_3_Ssc) deposited in Repbase, but not detected by our protocol, were also included for annotation. A few older subfamilies with too few (< 10) copy numbers to derive accurate consensus sequences were removed from the dataset. It is very likely that additional, ancient, small copy number subfamilies exist, but were missed by our approach. The remaining subfamilies were further classified into four distinct families (named L1A, L1B, L1C, and L1D) based on the polygenic tree of 5′UTR (Fig. 1). The names, classification, characteristics, divergence, and copy numbers of these L1 s are summarized in Table 1 and Additional file 2: Table S1, and the consensus sequences of each subfamily are supplied in Additional file 1. The total length of the consensus varied between 5837 and 8822 bp, while the length of the 5′UTR varied widely from 551 bp to 3254 bp, and the 3′UTR (excluding polyA sequence) varied from 180 bp to 305 bp between subfamilies. The intergenic region (IGR) ranged from 390 bp to 529 bp, except two subfamilies (L1A1 and L1A2) containing very short IGRs (67 and 68 bp), while the lengths of open reading frame 1 (ORF1) (about 900 bp) and ORF2 (about 3800 bp) were relatively conservative across all subfamilies and families (Table 1 and Additional file 2: Table S1). The copy number of L1 elements, number of subfamilies, divergences, and the copy number of full length L1 elements varied significantly between families. The number of subfamilies across L1A, L1B, and L1C families, and the copy number of elements in each subfamily are generally similar, but subfamily L1A4 of L1A tended to show more elements compared with the other subfamilies of L1A, L1B, and L1C families. The family L1D represents the highest diversity, with 22 subfamilies, and this family also displays the highest activity, with several subfamilies containing members with the potential to encode, and most subfamilies show lower divergence compared with other families (Table 1 and Additional file 2: Table S1). In total, 98 putatively active L1 elements with a typical structure of mammal L1 were identified, and they distributed in 12 different subfamilies of L1D family. Most of them tend to have a longer 5′UTR compared with other subfamilies (Table 1 and Additional file 2: Table S1). Thus, in the pig genome, the putatively active L1 elements are 7–9 kb long and contain a 5′UTR with length ranging from 1.5 kb to 3.2 kb, a ca. 270 bp 3′UTR, two open-reading frames (296 aa ORF1 and 1272 aa ORF2), and a relatively long (ca. 520 bp) IGR that separates the two ORFs. L1 insertions typically end with an A-rich tail and are flanked by short (< 20 bp) target site duplication (Fig. 2a).

**Table 1:** Classification of L1 families in the pig Genome

L1 family	Subfamily Number	Length (bp)						Active L1 Number
		Consensus	5’UTR	ORF1	IGR	ORF2	3’UTR (No PolyA)	
L1A	7	5837–7404	931–1959	897–906	67–396	3655–3828	180–305	
L1B	12	5975–7740	551–2335	878–910	390–447	3766–3813	217–305	
L1C	13	6462–7532	1037–2024	879–891	385–529	3766–3814	247–268	
L1D	21	7072–8822	1562–3254	887–891	501–521	3807–3819	270–277	98

Analysis of the age distribution between the pig-specific L1 families (L1A, L1B, L1C, L1D) other L1 families (mammal common), L2 superfamily, and other LINEs revealed that the mammal common L1 s, L2, and other LINEs were fossils, represented ancient proliferation, and dominated the genome evolution between 30 and 80 million years ago (Mya); their activities have essentially ceased for over 30 million years (Fig. 2b), while the four pig-specific families of L1 have dominated evolution over the last 30 Mya. Further comparison of the age distribution across the four families clearly showed that they proliferated at different evolutionary periods and represented variable evolutionary profiles (Fig. 2b). Generally, both the L1A and L1B families displayed an extended accumulation during their evolutionary history; they amplified and evolved simultaneously for as long as 80 Mya, from 90 Mya to 10 Mya, and burst between 10 and 20 Mya. By contrast, both the L1C and L1D families amplified over the last 20 Mya in the evolution of the pig genome. L1C displayed a low expansion between 5 Mya and 20 Mya and tended to be dead in the last 5 Mya, whereas L1D represented the youngest and most active family in the pig L1 clade and showed a sharp amplification in the last 10 Mya, with peak activity at 5 Mya; indeed, they are potentially still active (Fig. 2b), which is also consistent with the results of age analysis and the identification of about 100 putatively active L1 elements in this family. Further analysis revealed that L1D1–7 subfamilies may represent the youngest subfamilies across this family, compared with other subfamilies (L1D8–21) (Fig. 2c and d), with each subfamily containing many putatively active L1 copies. This observation was also well supported by the insertion polymorphism analysis of L1D1 in both inter- and intrabreed pigs (Fig. 2e). The long history of expansions means that the abundance of most subfamilies of families L1A and L1B was significantly higher than that of L1C and L1D (Table 1 and Additional file 2: Table S1).

### Three expansion waves of pig-specific SINEs represented by three distinct families

Diverse pig-specific SINE elements have been identified in a previous study [30] and deposited in Repbase (https://www.girinst.org/), and all these SINEs are tRNA-derived. We also tried to use MITE-Hunter, which is a program for discovering miniature inverted-repeat TEs from genomic sequences and can be used to identify SINEs, and RepeatModeler to extract SINE elements; however, we did not find any new families. Thus, these SINEs in Repbase were classified into three families (named SINEA, SINEB, and SINEC) based on length and structure, as shown in Additional file 2: Figure S1 and Table S2 (family, new name and Repbase name, length) and Additional file 1. All SINE elements of SINEA, SINEB, and SINEC families showed similar structure organization, with a tRNA head, a TC-rich region, a GC-rich region, and an A-rich tail (Fig. 2a). Similar to Alu in humans [46] and B1 and B2 in mice [47], the tRNA head of pig SINE harbors the conserved A and B box sequences that are required for RNA polymerase III dependent transcription. The TC-rich region also presents in carnivore SINE elements [48]. The elements of SINEA family are approximately 250 bp in length, with the exception of a polyA tail, while the elements of SINEB and SINEC family are shorter, with about 200 bp and 120 bp lengths, respectively. Sequence length variations between the A and B box sequences of the tRNA head of SINEB and SINEC families’ elements have been observed, whereas the SINEA elements are highly conserved and display high sequence similarity, indicating that SINEA may represent the youngest family (Additional file 2: Figure S1 and Table S2).

Three waves of expansion of SINEs can be identified in pigs based on the tempo of their evolution, and each wave corresponds to the activity of one family. However, most of these families, including SINEB, SINEC, MIR, and others, have been extinct for at least 20 million years (Fig. 2f); the most recent expansion corresponded with the activity in the family of SINEA. This family dominated the evolution history of SINEs in the pig genome during the last 50 Mya, and still displayed activity during the last 10 Mya. Three subfamilies (SINEA1, SINEA2, and SINEA3) of this family represented the youngest SINE elements compared with other subfamilies (SINEA4–11), and may currently be active, with many copies inserted in the last 5 million years (Fig. 2g and h); this was also supported by the insertion polymorphisms of SINEA1 in both inter- and intrabreed pigs (Fig. 2e). While the MIR represents the oldest family, its retrotransposon activity peaked approximately 65 Mya, and SINEB and SINEC represents the second oldest family; its retrotransposon activity peaked approximately 40–45 Mya (Fig. 2f).

### Experimental evidence for the Retrotransposition competence of pig L1

**Fig. 3:**
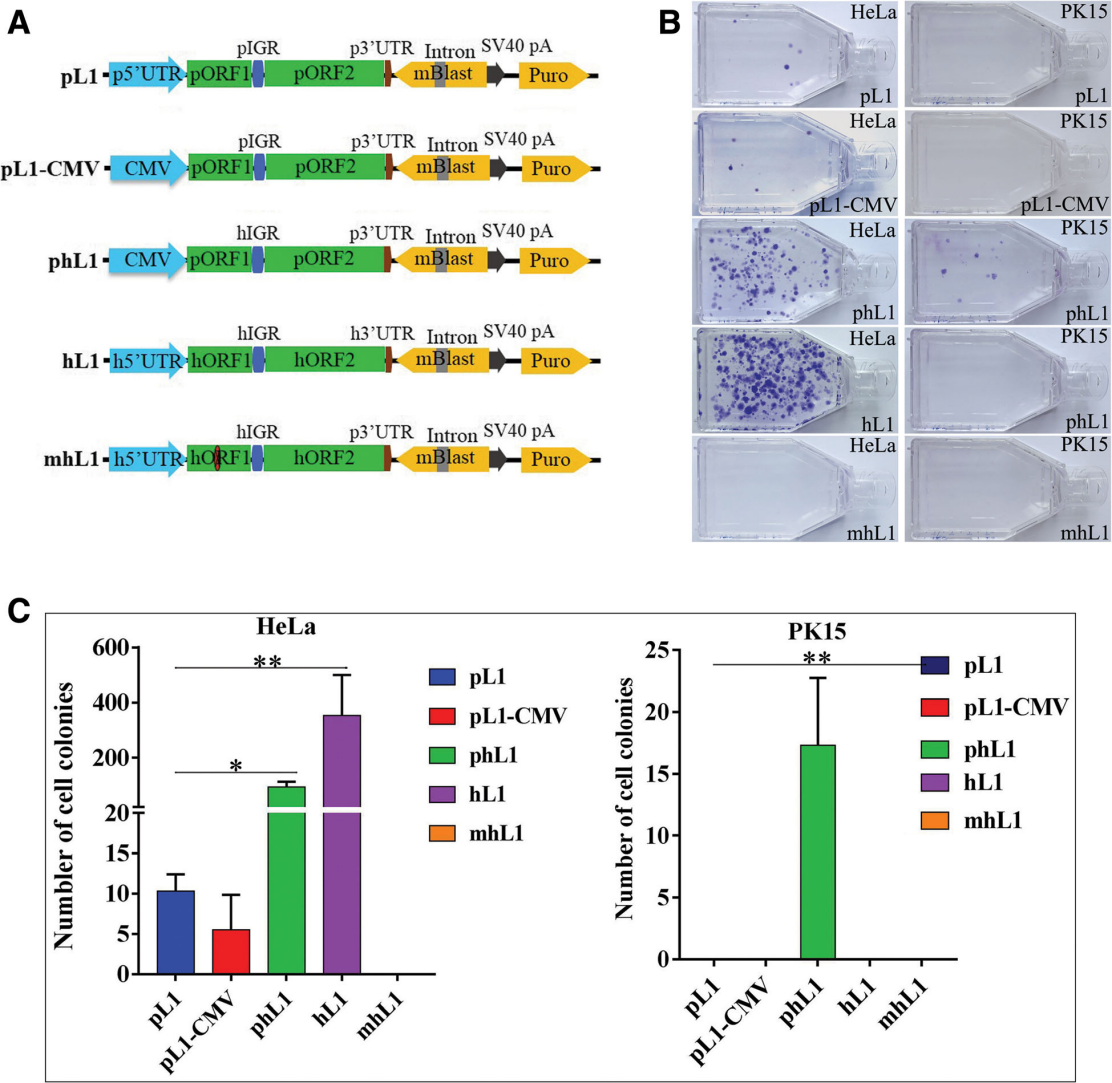
Retrotransposition activity analysis of pig L1. **a** Schematics of vectors used for retrotransposition assays. hL1 and mhL1 were used as positive and negative control, respectively. The pL1 vector contains 5′UTR, ORF1, IGR, ORF2, and 3′UTR of L1 cloned from the pig genome (L1D1 coordinate). The pL1-CMV is the same as pL1, but the 5′UTR of pig L1 was replaced with the CMV promoter. The phL1 is a chimeric vector derived by the CMV promoter, the two ORFs and 3′UTR were from pig, and the IGR was from human L1 (99-PUR-RPS-pBlaster1). All the vectors contain two selective cassettes (mBlast and Puro) for two-round selections. The mBlast cassette contains an inverted blasticidin resistance gene (black box) disrupted by a self-splicing intron [49–51]. The introns will only splice out from a transcript generated by the L1 or CMV promoter. The spliced RNA is reverse-transcribed, followed by integration of the cDNA into the genome. The new insert contains a functional Blast gene. Blasticidin resistance will be obtained only if retrotransposition occurs. **b** and **c** Number of clones formed after puromycin and blasticidin selection. Blast^R^ foci were fixed to flasks and stained with Giemsa for visualization. Bars represent the mean blasticidin resistant colonies ± standard deviation, shown as error bars for each construct

To determine the retrotransposition activities of L1, we used a retrotransposition assay with an indicator cassette consisting of blasticidin resistance gene in the antisense orientation (relative to L1) that is disrupted by an intron (γ-globin 2) in the sense orientation, which becomes functional only after a cycle of transcription, removal of the intron by splicing, RT, and integration [49–51]. We cloned the 5′UTR, ORF1, IGR, ORF2, and 3′UTR from the genomic coordinate of the youngest L1 (L1D1) subfamily and inserted it into the retrotransposon activity verification vectors, respectively, as described in the methods. We also used CMV as promoter to replace the 5′UTR of pig L1, and IGR of human L1 to replace the pig IGR. Human active L1 vector, which contains the most active L1 copy from the human genome, and mutant L1 vector, which is the same as active L1 vector but has an ORF1 mutant and cannot support retrotransposition [50], were used as positive and negative controls, respectively. The schematics of the constructs used are listed in Fig. 3a. We found that the cloned pig L1 was capable of retrotransposition in HeLa cells either with pig 5′UTR or CMV, but in a low level of retrotransposition activity compared with human L1 (Fig. 3b and c). Replacement of the pIGR with human IGR can improve the retrotransposition activity significantly. We also found that the retrotransposition activity of pig and human L1 s were cell-specific; weak retrotransposition activity of pig and human chimeric L1 (phL1) was observed in porcine kidney (PK15) cells, whereas human L1 did not work in the PK15 cell line (Fig. 3b and c).

### Identification of the Most “modern” ERV in the pig genome

**Fig. 4:**
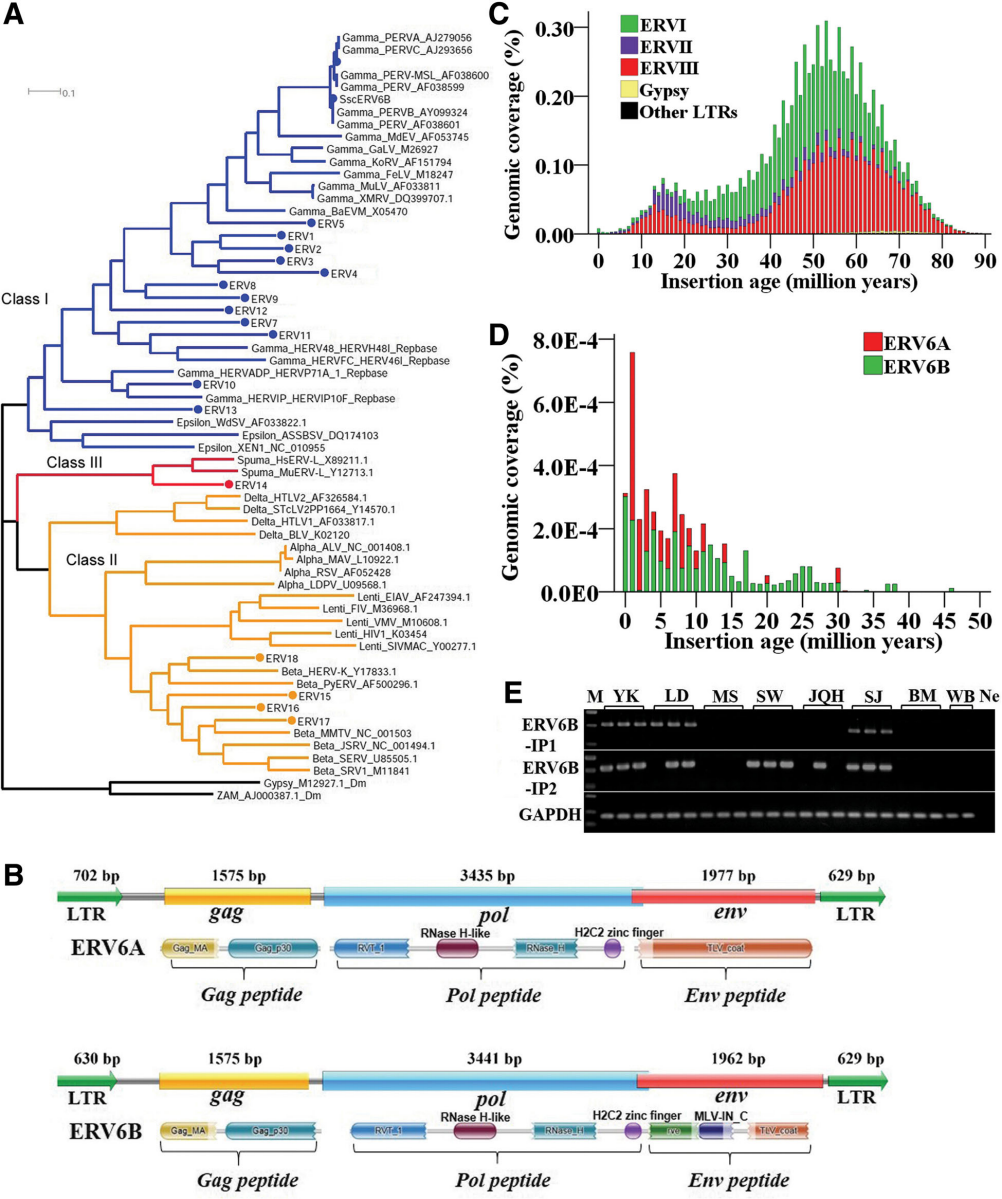
Evolution of ERVs in the pig genome. **a** ERVs were classified into 18 ERV families (ERV1–18) based on the phylogenetic tree inferred by using the Neighbor-joining method with the MEGA7 program, and the reference RT sequences from species other than pigs are included for comparison, shown with dots and described in the methods. **b** Structural schematics of the ERV6A and ERV6B, which featured LTR-*gag*-*pol*-*env*-LTR and were presumed to be active. Gag_MA: Matrix protein (MA), p15; Gag_p30: Gag P30 core shell protein; RVT_1: Reverse transcriptase (RNA-dependent DNA polymerase); RNase H-like: RNase H-like domain found in reverse transcriptase; rve: Integrase core domain; MLV-IN_C: Murine leukemia virus (MLV) integrase (IN) C-terminal domain; TLV_coat: ENV polyprotein (coat polyprotein) (**c**) Age distribution of pig ERV classes. **d** Age distribution of the youngest pig ERV subfamilies (ERV6A and ERV6B). **e** Insertion polymorphism detection of the youngest pig ERV subfamilies (ERV6B) by PCR. Breed name abbreviations are the same as those in Fig. 1f. The *x*-axis represents the insertion age (Mya), and the *y*-axis represents the percentage of the genome composed of retrotransposon families/subfamilies (%) in Fig. **c**, **d**

LTRharvest and RetroTector pipelines were used to detect ERVs in the pig genome DNA. A total of 2120 and 5456 ERV candidates were identified by using RetroTector and LTRharvest, respectively. Only ERVs with intact RT regions (ca. 0.7 kb) were retained, resulting in 29 and 240 ERVs from LTRharvest and RetroTector, respectively. They were then used for the subsequent phylogenetic analysis (Table 2 and Additional file 2: Table S3). These ERVs were classified into 18 families (ERV1–ERV18), including six families deposited in Repbase, based on the phylogenetic tree: 13 as gamma retroviruses of class I (ERV1–13), three as beta retroviruses of class II (ERV15–18), and one as spuma of class III (ERV14) (Fig. 4a, Additional file 2: Figure S2, and Table S3). The number of ERVs containing RT regions varied greatly among the types of retroviruses and families. Gamma retroviruses tended to have more ERV families and elements than did beta and spuma retroviruses. ERV candidates featuring two LTRs and three structural polyproteins common to all retroviruses, including group-specific antigen (*gag*), polymerase (*pol*), and envelope protein (*env*), were designated as full ERVs (Fig. 4b and Additional file 2: Figure S3). Most of the ERV families had decayed in pigs and tended to be inactive; only 19 copies of non-redundant ERV candidates were identified as full ERVs. Two of the latter, with the ability to encode long ERV proteins, were putatively active, and designated as “modern” ERVs (Fig. 4b and Additional file 2: Figure S3). All of the full and active ERVs identified in this study, as well as the transfection competent pig ERVs (γ1A, γ1B, and γ1C) identified in previous studies [52], were classified in the ERV6 family of gamma retroviruses, which were further classified into ERV6A and ERV6B subfamilies based on LTRs (Additional file 2: Table S3). The consensus or representative sequences were derived for each family or subfamily (Additional file 1; Additional file 2: Table S3). Most ERVs were typically between 8.5 Kb and 11 Kb in length, and the length of LTRs varied from 110 to 702 bp. Each of the two youngest subfamilies of ERVs (ERV6A and ERV6B) contained one putatively active ERV element with lengths of 8918 bp (chr5:92185133–92,194,050 -) and 8757 bp (chr9:138895584–138,904,340 -), respectively. The putatively active ERV element of ERV6A encoded an 1, 748 aa peptides containing *gag*, *pol*, and *env*, which are essential for replication, and flanked with 702 bp LTRs, while the active ERV of ERV6B subfamily encoded an 1, 776 aa peptide harboring *gag*, *pol*, and *env*, but flanked with 629 bp LTRs (Fig. 4b and Additional file 2: Figure S3).

**Table 2:** Number of ERV detected by LTRHarvest and Retrotector in the pig genome

Structure	Number of detected elements	
	LTRHarvest	Retrotector
Total	5456	2165
ERV containing RT (about 700 bp)	29	240
ERV containing *gag* (about 1500 bp)	20	80
ERV containing *pol* (about 3500 bp)	18	67
ERV containing *env* (about 3500 bp)	12	30
ERV containing *gag, pol*, and *env*	9	19
Copy number of Non-redundant FL ERVs	19	
Copy number of putative active ERV	2

Overall, the expansion profile of the three classes of ERVs was very different in the pig genome. Class I and III ERVs displayed abundant amplification and dominated the whole evolution history of ERVs in the pig genome, whereas class II ERVs were the least abundant and showed a very weak expansion during the whole evolution history of ERVs. The other LTRs, including Gypsy, displayed extremely low amplification. Most of the ERV families appeared to be defective, with a striking deceleration in activity over the last 10 million years, and most of them seemed to cease in the most recent 5 million years (Fig. 4c). However, one possible exception was the family of ERV6, which exhibited an extended expansion between 30 and 0 Mya and a burst in the last 10 million years, and displayed signs of current activity. By contrast, the ERV6B subfamily may represent the youngest ERVs in the pig genome (Fig. 4d), combining the insertion polymorphisms detection of ERV6B by PCR in both inter- and intrabreed pigs (Fig. 4e), strongly suggesting that the current activity of this subfamily may represent the most “modern” ERV.

### Young L1 5′UTRs and ERV LTRs displayed sense and antisense promoter activities

**Fig. 5:**
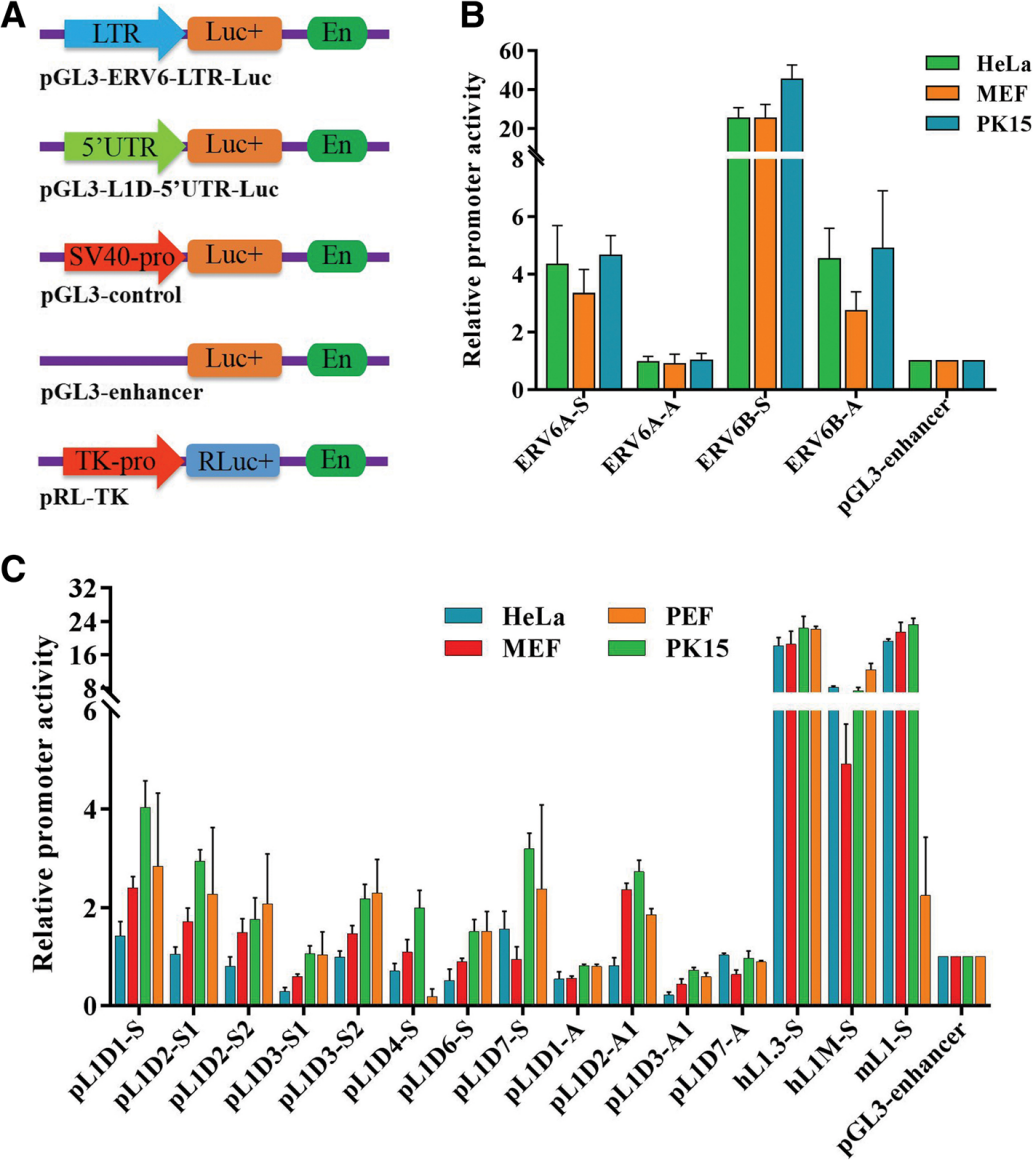
Sense and antisense promoter activities of pig L1 5′UTRs and ERV6 LTRs**. a** Schematics of vectors used for promoter activity detection by luciferase assay. The sense and antisense 5′UTR/L1 and LTRs of ERVs from young and putatively active subfamilies of L1 were cloned into the pGL3-enhancer luciferase reporter vector to investigate the promoter activity. **b** Sense and antisense promoter activities of ERV6A and ERV6B LTRs measured by luciferase assay. **c** Sense and antisense promoter activities of young L1 5′UTRs (L1D) measured by luciferase assay. Eight sense and four antisense L1 5′UTRs from different subfamilies of L1D family were cloned as described in the methods, and two 5′UTRs (hL1–3 and hL1-M) of active L1 s from human and one 5′UTR (mL1) of active L1 from mouse were used as positive controls

The sense and antisense LTRs from the putatively active family of ERV (ERV6A and ERV6B) were cloned into the pGL3 luciferase reporter vector to investigate the promoter activity based on the luciferase assay; the vector schematics are shown in Fig. 5a. ERV6B sense LTR had the highest promoter activity in three tested cell lines, while ERV6A sense LTR and ERV6B antisense LTR showed moderate promoter activity. The promoter activity of ERV6A antisense LTR was not detectable (Fig. 5b). We also explored the promoter activities of eight sense and four antisense 5′UTRs from young and putatively active subfamilies of L1D. Four of the sense 5′UTRs were members of the L1D1, L1D4, L1D6, and L1D7 subfamilies, and two of them were members of the L1D2 and L1D3 subfamilies. The four antisense 5′UTR were in the L1D1, L1D2, L1D3, and L1D7 subfamilies. Two 5′UTRs of active L1 s from the human genome and one 5′UTR of active L1 from the mouse genome were used as positive control; the schematics of these vectors are shown in Fig. 5a. The sense and antisense 5′UTRs of pig L1 s displayed lower or no promoter activity compared with human and mouse. Strong promoter activities were observed for all 5′UTRs of human and mouse in all four cell lines. Three sense 5′UTRs (L1D1, L1D2, L1D7) and one antisense 5′UTR (L1D2) of pig L1 s showed detectable promoter activity (Fig. 5c).

### Young L1 s and ERVs displayed sense and antisense expressions in multiple tissues and cell lines

**Fig. 6:**
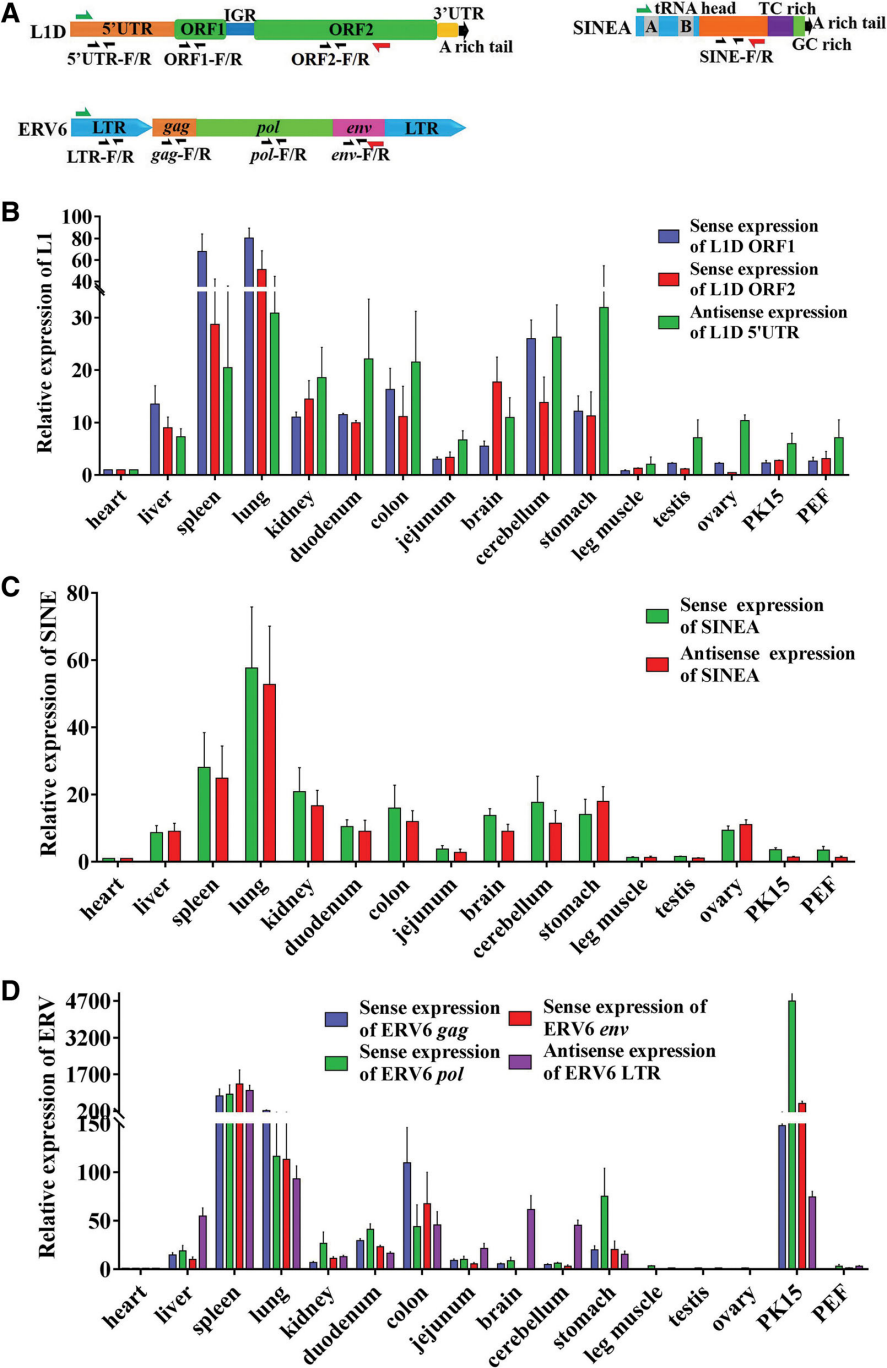
Sense and antisense expression profiles of pig L1D of L1 s, SINEA of SINEs, and ERV6B of ERVs. **a** Primer design for reverse transcription (RT) and real-time quantitative PCR (RT-qPCR) detection. The primer for sense and antisense RT are indicated by red and green arrowheads, respectively, and the primers of ORF1-F/R, ORF2-F/R, 5′UTR-F/R, pol-F/R, gag-F/R, env-F/R, LTR-F/R, SINE-F/R (black arrowheads), are used for RT-qPCR to detect the expression of 5′UTR, ORF1, and ORF2 of L1, LTR, *gag*, *pol*, and *env* of ERV6 and SINE, respectively. **b** Sense expression of ORF1 and ORF2, and antisense expression of 5′UTR of L1D in tissues and cells. **c** Sense and antisense expression of SINEA in tissues and cells. **d** Sense expression of *gag*, *pol*, and *env* of ERV6, and antisense expression of LTR of ERV6 in tissues and cells

The sense and antisense expressions of the youngest families from three types of retrotransposons, including L1D of L1 s, SINEA of SINEs, and ERV6 of ERVs, were evaluated by real-time qualification PCR (RT-qPCR) in 12 pig tissues (heart, liver, spleen, lung, kidney, duodenum, jejunum, brain, cerebellum, leg muscle, stomach, colon, testis, ovary) and two pig cell lines (PK15 and PEF). The primers were designed to target the conserved regions of 5′UTR, ORF1, and ORF2 of L1D1, and SINEA, and the conserved regions of LTRs, *gag*, *pol*, and *env* genes of ERV6 (Fig. 6a). The quality of RNA extracted from each sample was confirmed by RNA electrophoresis. RNAs treated with DNase and cDNAs were used as negative and positive control templates, respectively, for PCR amplification of ORF1 of L1 and *gag* of ERV to identify potential DNA contamination (data not shown). Overall, we found all types of detected young retrotransposons showed a similar expression profile between somatic tissues and cell lines; they all displayed antisense expression. Differential expression profiles across L1, SINE, and ERV retrotransposons were observed in the gonads (ovary and testis) (Fig. 6b-d). The sense expressions of L1 ORF1, L1 ORF2, ERV *gag*, ERV *pol*, and ERV *env* and the antisense expression of ERV LTR were repressed in the gonads, while clear antisense expression of L1 5′UTR was observed. In addition, both the sense and antisense transcripts of SINE were detected in the ovary, but neither were detected in the testis. The ORF1 and ORF2 of L1 displayed similar sense expression profiles in somatic tissues and cell lines, with high levels in the lungs and spleen, medium levels in the brain, cerebellum, colon, duodenum, kidney, liver, and stomach, and low levels in heart, jejunum, muscle, and PK15 and PEF cell lines. Antisense expression 5′UTR of L1 in these tissues and cells displayed similar patterns to ORF1 and ORF2, but with medium or low levels (Fig. 6b). The expression pattern of SINE in different somatic tissues and cell lines was similar to that of LINE. The sense and antisense expressions of SINE had almost the same pattern (Fig. 6c). The antisense expression of ERV6 LTR and the sense expression of ERV coding regions (*gag*, *pol*, *env*) had similar overall profiles to those of LINE and SNIE, but higher antisense expression levels of ERV6 LTR were observed in the brain and cerebellum (Fig. 6d). Taken together, our data suggest that these retrotransposons may share a common regulatory mechanism in somatic tissues and cell lines, but a differential regulatory mechanism in gonads.

### Over 80% of protein coding and lncRNA genes overlap with retrotransposon insertions

**Fig. 7:**
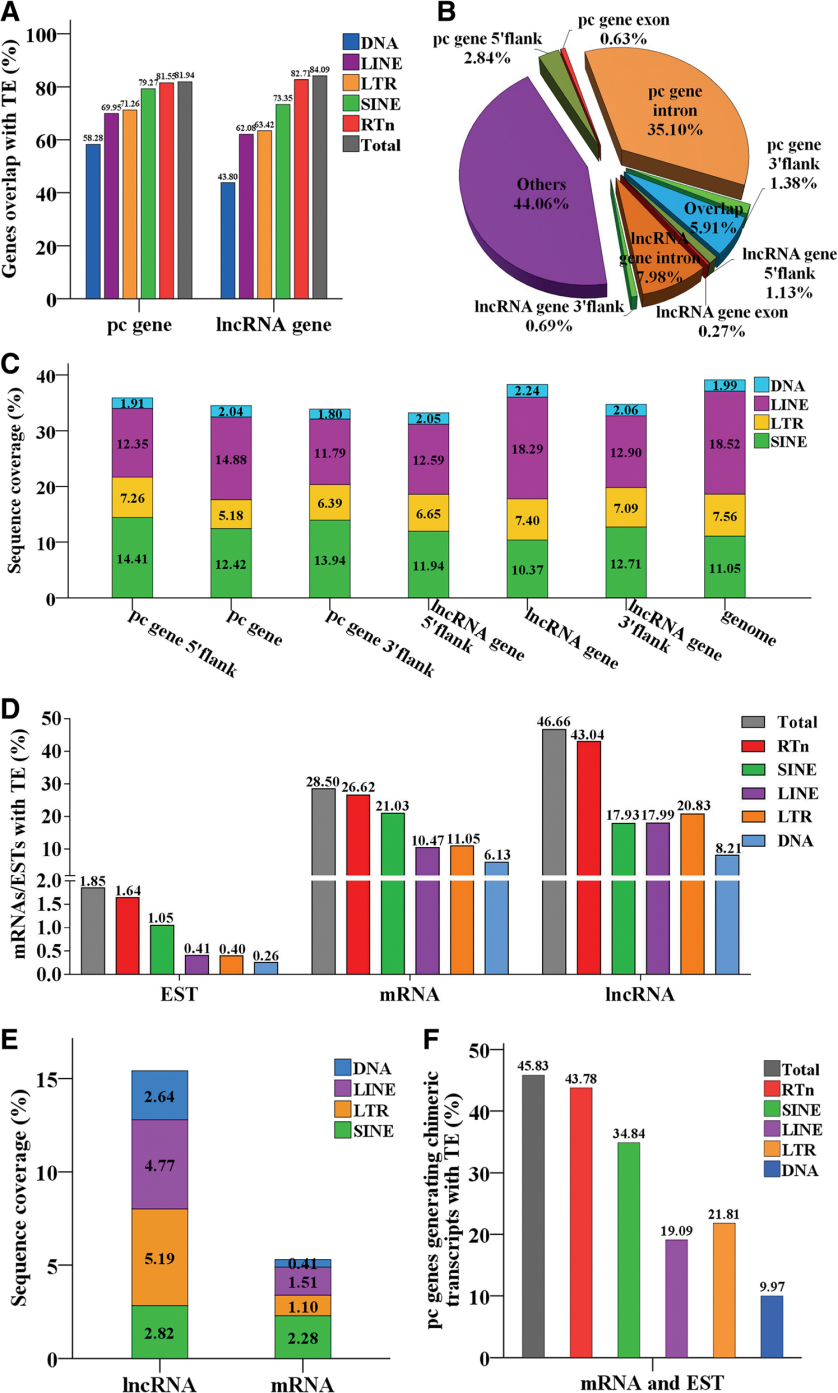
Retrotransposons contribution to protein coding and lncRNA genes. **a** The proportion of protein coding (pc) genes and lncRNA genes overlapping with retrotransposon insertions. **b** The proportion of TE insertions in the introns and exons of protein coding and lncRNA genes, and their flank regions. **c** The genomic coverage of retrotransposons in protein coding (pc) and lncRNA genic regions, and their flank regions. **d** The proportion of mRNAs, ESTs, and lncRNAs containing retrotransposon-derived sequences. **e** Sequence coverage of retrotransposons in lncRNAs and mRNAs. **f** The proportion of the protein coding genes generating chimeric transcripts with retrotransposons

The intersection analysis between protein coding genes, lncRNA genes, their flank regions, and TE insertion positions indicated that the majority of protein coding and lncRNA genes overlapped with TE insertions. In general, 81.94% (17,278 out of 21,087) of the protein coding genes and 84.09% (12,174 out of 14,477) of lncRNA genes contained TE insertions (Fig. 7a), accounting for about 35.73% and about 8.25% of the total TE insertions, respectively (Fig. 7b). In detail, 79.27% of protein coding and 73.35% of lncRNA genes harboring SINE insertions, 71.26% of protein coding and 63.42% of lncRNA genes harboring LTR insertions, 69.95% of protein coding and 62.08% of lncRNA genes harboring LINE insertions were observed respectively (Fig. 7a). One-third of TEs hit the introns of protein coding gene (35.10% of total TE insertions) and some hit lncRNA introns (7.98% of total TE insertions), but very few (< 1% of total TE insertions) were in the exons. Furthermore, a substantial proportion (5.91%) of TE insertions hit the overlapping regions of protein coding and lncRNA genes (Fig. 7b). In addition, 9341 (44.30%) protein coding genes and 3494 (24.13%) lncRNA genes contained insertions from the youngest retrotransposon subfamilies, including L1 s (L1D1–7), SINEs (SINEA1–3), and ERVs (ERV6A and ERV6B). The youngest SINE subfamilies (SINEA1–3) displayed the most extensive distribution in protein coding (9230/43.77%) and lncRNA (3402/23.50%) genes, and represented the highest insertion frequency compared with other retrotransposon types (Table 3).

**Table 3:** The number of lncRNA genes and protein coding genes contain the insertions from youngest retrotransposons

Young RTn	protein coding gene	lncRNA gene
L1D1	120	49
L1D2	129	60
L1D3	47	25
L1D4	61	29
L1D5	149	59
L1D6	108	28
L1D7	204	96
Total youngest L1 s	660 (3.13%)	286 (1.98%)
ERV6A	30	13
ERV6B	24	17
Total youngest ERVs	42 (0.20%)	21 (0.15%)
SINEA1	3464	1038
SINEA2	7696	2612
SINEA3	3786	1134
Total youngest SINEs	9230 (43.77%)	3402 (23.50%)
Total	9341(44.30%)	3494 (24.13%)

The percentage in parentheses is the percentage of protein coding/lncRNA genes with youngest retrotransposon insertions account for total protein coding/lncRNA genes

While the annotation of the mobilome in pig revealed that young retrotransposon subfamilies only occupied a small proportion of the pig genome, with less than 1% of total genome covered by the youngest subfamilies (L1D1–7/0.19%, SINEA1–3/0.63%, and ERV6/0.02%) (Additional file 2: Figure S4A), compared with the genome coverages of LINE (18.52%), LTR (7.56), and SINE (11.05%) (Additional file 2: Figure S4B). The pig-specific L1 s, SINEs, ERVs represented about 10.00, 10.00, and 7.00% of the genome, whereas the youngest subfamilies of L1 (L1D) and SINEA represented 1.13 and 7.64% of the genome, respectively (Additional file 2: Figure S4C). In addition, lncRNA and protein coding genic regions and their flank regions exhibited many biases in their retrotransposon composition and orientation relative to genomic averages (Fig. 7c and Additional file 2: Figure S4D). Though also the most prevalent TE families in the genic regions of lncRNA and protein coding genes, and their flank regions, LINEs were significantly depleted, with a range from 11.79 to 14.88% in protein coding and lncRNA genic, and their flank regions, with the exception of LINEs in lncRNA genic regions, where the LINEs represented similar coverage to the genomic average of about 18%. Both 5′- and 3′-flanks of lncRNA and protein coding genes tended to slightly enrich SINEs compared with their genic regions and genomic average, respectively (Fig. 7c). Most retrotransposons tended to insert into the opposite orientation in introns and exons of both protein coding and lncRNA genes; in particular, more than 30 and 15% of LINEs inserted in the opposite orientation in the introns of protein coding and lncRNA genes. A significant difference of insertion orientation frequency was observed for LINEs in introns of protein coding genes (*p* < 0.05), while SINEs in exons of protein coding genes and LTRs in exons of lncRNA genes displayed a bias of sense insertion orientation. The bias of sense insertion orientation of SINEs in exons of protein coding genes was also well supported by EST dataset analysis (Additional file 2: Figure S4D).

### Significant contribution of retrotransposons to the transcripts of lncRNA and protein coding genes

Intersection analysis showed that lncRNAs tended to enrich the TE-derived sequences compared with mRNAs, and nearly half (46.66%, 13,804/29,585) of lncRNAs overlapped with at least one TE (Fig. 7d), and 4.42% (1307/29,585) of lncRNAs designated as retrotransposon-lncRNAs, where more than 70% of the whole lncRNAs were covered by retrotransposons. In fact, about 15% of lncRNA sequences were occupied by TEs (Fig. 7e). By contrast, TEs overlapped only 28.50% of mRNAs, and covered only 5.30% of mRNA sequences (Fig. 7d and e). Retrotransposons were the major contributors of pig lncRNAs; they overlapped 43.04% of lncRNAs and covered 12.78% of their sequence (Fig. 7d and e). Furthermore, lncRNAs exhibited many biases in their TE composition relative to genomic averages. Though the LINEs and SINEs were the most prevalent in the pig genome, accounting for 18.52 and 11.05% of genomic sequences, respectively, both L1 s and SINEs are significantly depleted by about 4.0-fold. Conversely, LTRs are slightly enriched in lncRNAs compared with other retrotransposon types (Fig. 7e).

Almost half of the protein coding genes (45.83%) could generate chimeric transcripts with TEs (Fig. 7f). Retrotransposons were the major contributors of these chimeric transcripts, and they accounted for 4.89% of mRNA sequence; in total, 26.62% of mRNAs and 1.64% of ESTs contained retrotransposon-derived sequences, and these transcripts corresponded to 43.78% of protein coding genes (Fig. 7d and f). In addition, mRNAs comprised nonrandom distribution of retrotransposons, and SINEs were the most prevalent in mRNAs, overlapping with 21.03% of mRNAs and 1.05% of ESTs, respectively, accounting for 2.28% of mRNA sequences and corresponding to 34.84% of total protein coding genes. LINEs and LTRs only overlapped with about 10% of mRNAs and 0.4% of ESTs, respectively, and accounted for 1.10 and 1.51% of mRNA sequences, respectively, which corresponded to about 20% of protein coding genes (Fig. 7d and f). In addition, retrotransposons, mainly represented by SINEs, were primarily located in 3′UTRs of mRNAs, and overlapped 28.38% of 3′UTRs of mRNAs; this bias of SINEs was not observed for the 3′-end of lncRNAs (last exon). Conversely, less than 0.4% CDS and 3.64% of 5′UTRs overlapped with TEs, and there appeared to be significant deletion of TE-derived sequences (Additional file 2: Figure S4E).

## Discussion

### Both L1 s and SINEs displayed multiple wave amplifications dominated by different families in the evolution of the pig genome

The data presented here defined the classification of major retrotransposon types (L1 s, SINEs, and ERVs) at multiple levels, and the evolution dynamics analysis revealed that these retrotransposons presented multiple wave amplifications that were dominated by different families in the evolution of the pig genome. We classified pig-specific L1 s into four distinct families (L1A, L1B, L1C, and L1D) and 51 subfamilies. About 100 L1 copies were identified as intact and putatively active elements, which is similar to that in human, where it was postulated that out of the 1318 full-length L1 sequences, 146 were intact and putatively active, but substantially lower in number compared with that in mice, where 2811 out of 14,076 full-length L1 elements were estimated to be potentially retrocompetent [40]. Substantially different amplification dynamics of families of L1 s during the evolution history of the pig genome were observed. Ancestral pig genomes contained two distinct L1 families (L1A and L1B), which amplified and evolved simultaneously for about 80 million years, ranging from 85 Mya to 5 Mya. Then, two families (L1C and L1D) replaced their predecessors as the dominant families, and these were amplified over the last 20 million years. The four families occasionally coexisted in pig evolution for a short period of between 20 Mya and 5 Mya. This pattern of evolution was generally similar to that of humans, where several old and distinct L1 families coevolved for over 30 million years in the ancestral genome, and a new family of L1 amplified over the last 40 million years [53]. The families of L1A, L1B, and L1C were old and more divergent than that of L1D. The former showed no sign of current activity because of extensive accumulated mutations, whereas the L1D family represented the most active family of L1, which was also supported by the identification of about 100 intact L1 elements and the insertion polymorphisms of this family in both inter- and intra-breed pigs. These data suggest that L1D represented the most active family of L1 in pigs. Pig-specific SINEs, with a length between 102 and 265 bp (without polyA tail, Additional file 2: Table S2), were classified into three families (SINEA, SINEB, and SINEC) based on sequence similarity and length; the three families display periodic fluctuations with three large waves of fixation, and occasionally coevolved for a long period between 20 Mya and 80 Mya. SINEB and SINEC are old families, and the activity was extinct in the last 20 million years, while the SINEA family represented the most recent expansion and still displayed activity during the last 10 million years; SINEA1–3 represented the youngest subfamilies of this family. These data indicate that both L1 s and SINEs displayed periodic fluctuations with multiple wave amplifications, but were dominated by different families in the evolution of the pig genome, and some families of both L1 s and SINEs coevolved at particular stages.

### ERV6s are “modern” ERVs

ERVs, which are the dominant LTR retrotransposons within mammalian genomes, have been invading mammalian lineages for over 100 million years [54]. Early genome sequencing studies showed differences in the activity of retroviruses among mammalian species, with humans largely containing inactive ERV families [6] and mice containing numerous active ERV families [29]. These active ERVs are generally referred to as “modern” ERVs because they have integrated into the host genome after speciation and are closely related to exogenous viruses. They are still able to produce infectious viruses because of the lack of deactivating mutations. Active ERVs have also been found in other mammal species, such as in koala (KoRV) [55], Jaagsiekte sheep (JSRV) [56], and domestic cat (ERV-DC) [57]. In this study, we characterized the diversity, structure, activity, and evolutionary history of pig ERVs. Thousands of ERV candidates were identified in the present study, and most of these ERVs had decayed; only about 250 candidates contained intact RT regions, which were classified into 13 gamma ERVs, three beta ERVs, and one spuma ERV by phylogenetic analysis. Moreover, most of these families appeared to be more defective, with a striking deceleration in recent activity, with the exception of ERV6, which belonged to the gamma retroviruses of ERVI and included two subfamilies (ERV6A and ERV6B); this family still exhibited an extended period of expansion and showed signs of increased activity in the last 10 million years, with a few copies encoding long peptides with intact *gag*, *pol*, and *env* domains, which is in good agreement with the findings in the Wuzhishan pig genome [45]. Furthermore, all the active pig ERVs (γ1A, γ1B, and γ1C) reported in previous studies [52] were also classified into this family. The ERV6B subfamily tended to be the youngest and most active subfamily based on age analysis, and the insertion polymorphisms of this subfamily were also confirmed. Overall, these data suggest that most ERVs are fossils that are fixed in the pig genome, while ERV6s are “modern” ERVs that are putatively active and play a role in the evolution of the genome. In addition, theses ERVs carry potential risks for human xenotransplantation, which have been extensively noted [58, 59].

### Evidence for sense and antisense promoter activities of L1 5′UTRs and ERV LTRs

The insertions of retrotransposons may impact gene activity by offering alternative RNA polymerase II (Pol II) promoters. It seems that most retrotransposons harboring Pol II promoters, such as ERVs, often contained RNA polymerase II (Pol II) promoters within the LTR flanking coding sequence of the elements [60]. Both sense and antisense Pol II promoter activities of L1 s in humans and mice have been characterized. Moreover, the antisense coding capacity of human L1 has been established [61]. The antisense Pol II promoter of human L1 is located in the 5′UTR, while that of mice is located in the ORF region [62]. It has been confirmed that the L1 antisense promoter activity could drive chimeric transcripts [36, 63]. In the present study, we provided evidence to support the sense and antisense Pol II promoter activities in the 5′UTRs of pig L1 s, although the activity levels were low compared with those in humans and mice. The sense Pol II promoter activities of pig ERV have been characterized [64, 65], and our data confirmed the sense Pol II promoter activities of both ERV6A and ERV6B subfamilies. This was the first time we observed the antisense Pol II promoter activities of ERV6. The promoter activity analysis of these young retrotransposons offers a new perspective to understand their impact on genome, given that new insertions can provide new promoters. Such examples of host genes driven by TE promoters have been documented in diverse species over the past several decades [66–68]. Generally, TE promoters often show spatially or temporally regulated activity that is dependent on cell type and/or in response to environmental cues such as stress or infection [69, 70].

In addition, the detected young retrotransposons showed similar overall sense and antisense expression profiles in somatic tissues and cell lines in the current study, indicating that these retrotransposons may share a common regulatory mechanism in somatic tissues and cell lines. However, in the gonads (ovary and testis), their expression patterns are different, indicating different regulatory mechanisms. A lack of sense expressions of L1 and ERV, and obvious antisense expression of L1 5′UTR, was observed in the gonads, which supports previous studies, but also suggests that their expression may be restricted to various stages of gametogenesis [60]. Germline suppression of TE activity can be achieved through both the epigenetic mechanism, including DNA methylation and heterochromatin formation, and small RNA-mediated post-transcriptional regulations [71, 72]. The antisense expression of L1 may actually play a role in the repression of sense expressions of L1 in the gonads by an RNA interference pathway, as suggested previously [62, 73]. However, the activation of sense and antisense transcriptions of SINE in the ovaries is very interesting and suggests a biological role of SINE in this specific tissue, which may warrant further study to elucidate its physiological significance. On the other hand, the sense and antisense transcripts of these retrotransposons detected by qPCR in current study may not only originate from TE’s own promoters, but also generate from host gene promoters by co-expression (fusion expression) or other expression ways since the overlapping of retrotransposons and host genes is very common in genome.

### Retrotransposition competence of pig L1

We demonstrated that one subfamily of the youngest L1 s (L1D1) in pigs is capable of mobility by retrotransposition assay, which was also well supported by previous study, where it was found that a recent full-length endogenous L1 insertion in KPL2 gene caused the infertility of Yorkshire boars [74]. The retrotransposition activities of young L1 and SINE (Alu) in humans and L1 and SINE (B1) in mice have also been proven experimentally [41]. These data further suggest that most mammals contain retro-competent L1 and SINE. In addition, the retrotransposition activities of pig L1 were cell-specific, with high activity in human HeLa cells and very low activity in pig PK15 cells, while human L1 was not mobile in this cell line. Compared with human L1, the detected pig L1 (L1D1) displayed lower levels of retrotransposition activity, which could be an indication of either the low activity of the cloned element or the low overall retrotransposition activities of all pig-specific L1 s. In fact, the promoter activities of most detected young pig L1 subfamilies were lower than those of humans and mice in the present study, which supports the latter possibility. However, we also couldn’t exclude the possibility is that the retrotransposition of pig L1 may need additional cellular factors, and the retrotransposition assay need to be optimized in pig PK15 cells. As suggested by others, different components of L1, including 5′UTR, ORF1 and ORF2, and IGR, may impact the activity of L1 [43]; here, we also found that IGR plays an important role in the retrotransposition of L1. The retrotransposition activity of pig L1 was improved significantly with the replacement of human IGR, a finding that was also found in bat L1 [43], indicating that IGR plays a role in the evolution of L1. In addition, the risk of cross-species transmission of pig ERVs has been a concern in xenotransplantation [59]; here, our data provided experimental evidence of the retrotransposition-competent nature of pig L1 in human HeLa cells, indicating that the active pig L1 s and SINEs also carry a potential risk of horizontal transfer in xenotransplantation, which warrants further evaluation.

### Deep impact of retrotransposons on lncRNA and protein coding genes

Mammals are the best-studied vertebrates, largely because of the higher number of sequenced genomes spanning major lineages within the group [75]. Here, we found that the composition of TEs in the pig genome is dominated by retrotransposons, with LINE, LTR, and SINE accounting for 18.52, 7.56, and 11.05% of the sequenced genome, respectively, representing the typical mammalian characteristics [6, 29, 42]. However, the coverage of total repeat contents (40.72%) by this study is similar to that in early TE annotation of Duroc genome [30], but higher (38.2%) than that in Wuzhishan genome [45]. This disagreement may be due to an underestimation, since the Wuzhishan genome is far from complete compared with the reference genome of Duroc and dense repeat regions are underrepresented in the previous draft assembly. The high coverage of TEs in the genome and their ability to re-infect or move within the genome gives TEs an intrinsic propensity to possibly affect host genes. A significant association between the presence of intragenic L1 s and down-regulated genes in early embryogenesis was found in humans and mice [76]. L1 elements were present in an estimated 79% of human genes in at least one copy [6]. There are at least 124 documented LINE1-mediated insertions that have resulted in genetic disease in humans [77]. Many phenotype variations due to TE insertions have been observed in animals, such as SINE insertion causing body size variation and coat color pattern change in dogs [78–80], and ERV insertion causing eggshell color variation in chickens [81]. Two cases of phenotype variations due to L1 insertion were observed in pigs [82, 83].

Here, our data demonstrated that retrotransposons have an extensive impact on lncRNA and protein coding genes at both the genomic and transcriptomic levels. In pigs, 35.73 and 8.25% of the total TE insertions overlap with protein coding and lncRNA genes, respectively, and about 80% of protein coding and lncRNA genes contain retrotransposon insertions, which is generally similar to the estimations (about 90%) of the protein coding genes of bovines [84], mice, and humans [85, 86]. In addition, we found that although the youngest retrotransposons, including L1D1–7 of L1 s, SINEA1–3 of SINEs, and ERV6 of LTRs, occupy less than 1% of the genome; they overlap with about half of protein coding genes (44.30%) and one-fourth (24.13%) of lncRNA genes. These insertions may be new mobilization events, and the insertion polymorphisms of these families/subfamilies were also confirmed in the current study, indicating that the insertions of young retrotransposons may contribute to the structure variations of these genes, or even gene activities. These data also indicate that the retrotransposon insertion polymorphisms may be a very useful genetic marker to develop and warrants further study.

The intersection analysis between retrotransposon insertions and transcripts (ESTs and mRNAs) of protein coding genes revealed that at least 40% of protein coding genes are estimated to generate chimeric transcripts with retrotransposons, which are generally similar to the estimations in humans and mice, where 39% of human- and 35% of mouse-specific exons overlap with retrotransposons [87]. Retrotransposons are believed to be closely associated with the birth, evolution, expression, and function of lncRNAs in mammals, and strong contributors of lncRNAs [88, 89]. A significant negative correlation between the content of TEs and the level of expression of lncRNAs was observed [26, 28]. Very recently, a new class of natural lncRNAs that can activate translation by targeting sense mRNAs through the activity of embedded inverted SINEB2 elements, called SINEUPs, has been well characterized in mammals [90, 91]. The modular organization of SINEUPs strongly suggests that embedded TEs are fundamental for lncRNA function. This study also confirmed that the pig lncRNAs tend to enrich TE-derived sequences compared with mRNAs, which generally agrees with the findings in other mammals (including humans and mice) and fish (zebrafish) [26, 27]; however, the proportion (46.6%) of lncRNAs overlapping TEs in pigs is substantially lower than that in humans (83.4%), mice (68.2%), and zebrafish (66.5%) [26, 27]. This may be due to species differences; however, it clearly shows the importance of TE for lncRNA evolution. TE-derived sequences in the pig lncRNAs are dominated by retrotransposons, which overlap 43.04% of lncRNAs and cover 14.37% of their sequences; the same trend was also observed for the lncRNA sets in humans and mice [27, 28], suggesting that the high content of retrotransposon sequences is likely a contributing factor to sequence diversification and that the high complexity of lncRNAs is a general property in mammals.

In addition, significant biases in retrotransposon composition, orientation, and location in lncRNA and protein coding genes and their transcripts were observed. The mammal genomes are largely dominated by LINEs [6, 29, 30]. However, the most striking departure from this general trend is apparent in pig lncRNA and protein coding genic regions and their flanking regions. LINEs seem underrepresented in these regions, with the exception of the lncRNA genic region, which generally agrees with the trends in humans and mice [28]. In addition, LINEs and LTRs tend to insert in an antisense orientation in the introns of protein coding and lncRNA genes in pigs, and a similar trend of LINEs and LTRs has also been observed in the introns of protein coding genes of bovines [84] and humans [87]. Most retrotransposons (LINEs, LTRs, and SINEs) in the exons of protein coding and lncRNA genes in pigs are also preferentially inserted in the opposite orientation, whereas SINEs in exons of protein coding genes display a significant bias of sense insertion orientation, supporting the observations in bovine [84] and human studies [92]. LTRs in pig lncRNA exons also display sense orientation insertions, which is similar to humans [26], whereas SINEs tend to be enriched in the 3′-end of lncRNAs, and appear more often in the sense orientation in humans [26]; these biases of SINEs were not observed in pig lncRNAs. Furthermore, pig mRNAs and lncRNAs exhibit many biases in their retrotransposon composition and location. A relative under-repression of LINEs and SINEs, and slight enrichment of LTRs in pig lncRNAs were observed, similar to humans and mice [27, 88], whereas retrotransposons are primarily located in 3′UTRs of pig mRNAs; they were rarely located in 5′UTRs and coding regions. mRNAs, mainly represented by the 3′UTRs of mRNAs, tend to enrich SINEs other than LINEs and LTRs, also similar to humans and mice [92–94]. Global expression data indicate that the retrotransposon sequences in the 3′UTRs negatively affect the expression of mRNAs [93], suggesting that the SINEs in 3′UTRs may serve as targets for microRNAs [95, 96], thereby supporting another biological role of SINEs in the 3′UTRs of mRNAs.

In summary, these data indicate that redistribution of retrotransposons is a general property of mammalian lncRNA and protein coding genes and their transcripts. Retrotransposons in mammal genes may share a common regulation mechanism during evolution, and retrotransposons also play an important role in the structural organization, evolution, expression, and function of both protein coding and lncRNA genes.

## Conclusions

In the present study, we characterized the classification and evolution profile of retrotransposons in pigs. L1 s were detected and classified into four distinct families (L1A, L1B, L1C and L1D) and 51 subfamilies, and demonstrated that one youngest L1 s subfamily (L1D1) in pigs is capable of mobility by retrotransposition assay. SINEs were classified into three families (SINEA, SINEB, and SINEC) based on length and structure. ERVs were classified into 18 families (ERV1–ERV18) and most of ERVs had decayed, only ERV6 showed signs of increased activity in the last 10 million years, with a few copies encoding long peptides with intact gag, pol, and env domains. The sense and antisense expression profiles and promoter activities of young retrotransposons were characterized, young L1 5’UTRs and ERV LTRs displayed sense and antisense promoter activities. And we also investigated their impact on lncRNA and protein coding genes by defining the mobilome landscapes at the genomic and transcriptomic levels, significant distribution bias of retrotransposon composition, location, and orientation in lncRNA and protein coding genes, and their transcripts, were observed. These findings help provide a better understanding of retrotransposon evolution in mammal and their impact on the genome and transcriptome.

## Materials and methods

### Retrotransposons Mining in the pig Genome

The de novo detection of the L1 s in the pig genome was conducted with the MGEScan-non-LTR program [97]; however, most of the elements identified by MGEScan-non-LTR were incomplete. To obtain the full length of the elements, the sequences identified with the MGEScan-non-LTR program were aligned to the pig genome again by using Blat [98] (−minIdentity = 100, −minScore = 200). The alignment result file was converted into bed format file, and an additional 2500 bp 5′-flanking sequences and 200 bp 3′-flanking sequences were extended for each L1 sequence to define the boundaries of 5′UTR and 3′UTR by using the *bedtools slop* command (−s, −l 2500, −r 200). In addition, the available pig L1 elements in the L1Base database [40] (http://l1base.charite.de/l1base.php) were also downloaded with a bed file format. These two datasets were merged and the redundancy was removed (loci distance within 3000 in the same strand). Finally, the sequences of these L1 elements with unique positions in the pig genome were extracted by using the *bedtools getfasta* command (bedtools v2.27.0). The boundary of these L1 elements were defined by alignment and then clustered based on the 5′UTR sequence similarity; any clusters with fewer than 10 elements were removed. The final consensus sequence was constructed by using *cons* in EMBOSS explorer (http://www.bioinformatics.nl/emboss-explorer/) for each L1 cluster. The 5′UTRs of each consensus sequences were used for subsequent phylogenetic analysis.

ERVs were identified with LTRharvest [99] and RetroTector [100]. The LTR nucleotide similarity threshold used in LTRharvest was > 80%, with other parameters set to their defaults. A cutoff of 250 was used for RetroTector scores, as the majority of the elements with scores between 250 and 300 showed a conserved structure. Only ERVs with intact RT regions (about 0.5Kb) were retained and used for subsequent phylogenetic analysis and family classification. The consensus sequences or representative sequences were derived for each family/subfamily based on the phylogenetic tree.

### Phylogenetic analysis

Multiple alignments were constructed from the DNA sequences of the 5′UTR of L1 and the RT regions of ERV retrotransposons by using the ClustalX2 [101] program, respectively. We chose to use the DNA sequences to make the multiple alignments and build the phylogenetic tree, rather than the amino acid sequence, because of the presence of numerous frame-shift mutations and stop codons in the ancient retrotransposon elements. A Neighbor-Joining tree was generated from the alignment by using MEGA7 [102] with Kimura 2-parameter model and complete deletion as parameters. Bootstrap values were obtained from 100 replicates. The reference RT sequences of ERVs from species other than pigs were included for defining the classification of pig ERVs. The GenBank accession numbers and abbreviations of ERVs used for phylogenetic analysis are as follows: FeFV, feline foamy virus (AJ223851); HFV, human foamy virus (Y07725); HIV-1, human immunodeficiency virus 1 (K03454); SRV-1, simian SRV-1 type D retrovirus (M11841); MMTV, mouse mammary tumor virus (NC_001503); RERV, rabbit ERV (AF480925); RSV, rous sarcoma virus (AF052428); BLV, bovine leukemia virus (K02120); FELV, feline leukemia virus (M18247); KoRV, koala type C endogenous virus (AF151794); MDEV mus dunni endogenous virus (AF053745); and MuLV, Moloney murine leukemia virus (AF033811). In addition, γ1A (AJ279056), γ1B (AY099324), and γ1C (AJ293656) are the porcine ERVs identified previously.

### Retrotransposon annotation in the pig genome and transcriptome

The pig (Sscrofa11.1) genome was downloaded from the UCSC database (http://hgdownload.soe.ucsc.edu/goldenPath/susScr11/bigZips/). The lncRNA transcripts (29,585) and their coordinates of lncRNA genes (Bed format file) were downloaded from the NONCODE database (http://www.noncode.org/download.php). The Bed format file of lncRNA genes, which represents 17,811 lncRNA genes and corresponds to Sscrofa10.2, were converted into Sscrofa11.1 by LiftOver (http://genome.ucsc.edu/cgi-bin/hgLiftOver), and finally, the coordinates of 14,477 lncRNA genes were obtained. The coordinates of protein coding genes (21,087) and exons, the mRNAs (45,788) of protein coding genes, and the 5′UTR, 3′UTR, and CDS of protein coding genes were identified from the annotation of Sscrofa11.1 in Ensembl (ftp://ftp.ensembl.org/pub/release-91/gff3/sus_scrofa/). The total EST sequences (1,676,489) and their genomic coordinates were downloaded from the EST database (https://www.ncbi.nlm.nih.gov/nuccore) and the UCSC database (http://genome.ucsc.edu/cgi-bin/hgIntegrator), respectively. The 5′- and 3′-flank coordinates of protein coding and lncRNA genes were constructed based on the genes by extending 5 kb and 3 kb. The sequences of genes and flanks of genes were extracted from the genome by using *bedtools getfasta* according to their coordinates.

The newly identified L1 and ERV elements were combined to the known repeats in the pig genome, including SINEs from Repbase (version 20,170,127), and redundancies were filtered out to create a custom library. The distributions and coverage of TEs on the genome and transcriptome (lncRNAs and mRNAs) were then annotated with the custom library by using RepeatMasker (RepeatMasker -open-4.0.5) with a cutoff value of 250. The overlaps of TEs with protein coding (21,087) and lncRNA (14,477) gene introns and exons, and their flanking regions (5 kb upstream and 3 kb downstream), mRNAs (45,788), lncRNAs (29,585), CDS and UTRs (21,087 protein coding genes) were determined by intersecting these sets with TE annotations (described above) by using *bedtools*. Only overlaps of minimum 25 bp were retained.

The protein coding genes and TE chimeric transcripts in the pig genome were identified according to a high standard annotation strategy. Generally, the intersection between the coordinates of ESTs and protein coding genes were calculated, and only the ESTs with 90% of their coordinates overlapping with those of protein coding genes were retained; the remaining ESTs were then aligned to the mRNAs of protein coding genes by using Blat (−oneOff = 1, −minMatch = 4, −minScore = 90, −minIdentity = 95), and only the ESTs with more than 70% coverage of alignments with mRNAs were retained. Finally, these ESTs and mRNAs of protein coding genes were annotated de novo by using RepeatMasker (−cutoff 250, −nolow) with the custom library. The ESTs with over 80% of TE coverage, which may be completely transcribed from an active TE element, were also discarded. The remaining ESTs and mRNAs with at least 50 bases marked by repeats designated as TE chimeric transcripts were retained for statistical analysis. This strategy allows elimination of all the TE-cassettes that are inserted into protein coding genes but do not correspond to a protein sequence, or those that correspond to putative transcriptionally active TEs. Thus, overestimation of TE insertions in the protein coding genes of the pig was avoided.

### Age estimation

The average divergence and insertion ages of retrotransposons were estimated based on the divergence from consensus sequences by using RepeatMasker, and corrected as reported previously [103]. The average number of substitutions per site (K) for each fragment was estimated according to the divergence levels reported by RepeatMasker using the one-parameter Jukes-Cantor formula *K* = − 300/4 × Ln (1–*D* × 4/300), as described previously [29], where *D* represents the proportion of sites that differ between the fragmented repeat and the consensus sequence. Rough estimates of the ages of retrotransposons were obtained by using the equation *t* = *K*/2*r* [103], where *t* is the age and *r* is the average nucleotide substitution rate of mammalian genomes. Analysis of mammal genomes has shown that the rate of single nucleotide substitution remains relatively constant (1–2.2 × 10^− 9^ substitutions/site/year) [104, 105]. In the present study, we assumed an average mutation rate of 2.2 × 10^− 9^ per site per year for pigs. These time estimations do not necessarily represent exact dates, but provide relative approximations and simple calculations.

### Insertion polymorphism detection of Young retrotransposons

Seven domestic pig breeds (including Yorkshire, Landrace, Meishan, Shawutou, Jiangquhai, Sujiang, and Bama) and two wild boars were used for insertion polymorphism detection of the three youngest retrotransposon families/subfamilies (L1D1, SINEA1, and ERV6B) by PCR. Each domestic breed had three individuals. Meishan, Shawutou, and Jiangquhai pigs are native Chinese pig breeds from Jiangsu Province; the Sujiang pig is a newly established breed based on Duroc and Jiangquhai bloodlines; Bama pigs are miniature pigs from Guangxi Province; the wild boar was from Anhui Province; and the Landrace and Yorkshire pigs were from a breeding farm in Anhui Province. DNA was isolated from ear or blood samples of each sample by using the MiniBEST Universal Genomic DNA Extraction Kit Ver.5.0 (TaKaRa, Dalian, China). The concentration and quality of the DNA were measured using a spectrophotometer and electrophoresis in agarose gel. The primers (listed in Additional file 2: Table S5) designed for detection are shown in Additional file 2: Figure S5. For L1D1 and ERV6B, we designed a primer in its flanking region and another in their 5′UTR/LTR. For SINEA1, the primers were designed in its flanking regions, which span the SINEA1 insertion.

### Plasmid construction

#### Luciferase reporter vectors

Eight sense 5′UTRs of L1D family (one each from L1D1, L1D4, L1D6, and L1D7, and two from L1D2 and L1D3, respectively) and four antisense 5′UTRs of L1D family (L1D1, L1D2, L1D3, and L1D7), sense and antisense LTRs from both ERV6A and ERV6 were cloned from pig genomic DNA by nested PCR with Phanta Max Super-Fidelity DNA Polymerase (Vazyme, Nanjing, China). Two rounds of specific primers were designed according to the sequences from their genomic coordinates, and the restriction enzymes MluI/KpnI or MluI/SmaI were added to the 5′-flank of the second round of primers. We also cloned two 5′UTRs from human L1 (L1.3 and L1-M) [106] and one 5′UTR from mouse (mL1) [62] for positive control. Primers and their genomic coordinates are listed in Additional file 2: Table S6. PCR products were cloned into the cloning vector pLB (VT205; Tiangen, Beijing, China), and the correctness of the sequences was confirmed by sequencing. The 5′UTR or LTR was excised from the pLB vector by restriction enzyme digestion and inserted upstream of the firefly luciferase coding sequence in the pGL3-enhancer vector (Promega, Madison, WI, USA), respectively. The recombinant vectors were confirmed by sequencing again. The schematics of the vectors are shown in Fig. 4a.

#### Retrotransposon activity verification vectors

A total of five vectors (pL1, pL1CMV, phL1, hL1, and mhL1) were used in the retrotransposon activity analysis. The hL1 (99-PUR-RPS-pBlaster1) and mhL1 (99-PUR-JM111–5-15, the same as hL1, but ORF1 mutant and has no retrotransposon activity) were gifts from John L. Goodier and Haig H. Kazazian, Jr. [50] and were used as positive and negative controls, respectively. The 5′UTR and 3′UTR of pig L1D1, and the middle region of L1D1, including ORF1, IGR, and ORF2, were amplified by PCR from pig genomic coordinate (Sscrofa11.1 chr9:95235839–95,244,641), respectively. Human IGR was cloned from hL1 (99-PUR-RPS-pBlaster1). The human IGR was inserted into the middle of ORF1 and ORF2 of pig L1 by overlap PCR. The CMV promoter was cloned from the pEGFP-N1. These fragments were inserted into TA cloning vectors and confirmed by sequencing. They were assembled into vectors of pL1, pL1-CMV, and phL1 by ligase with the designed restriction enzyme sites. The primers used to amplify the fragments described above are listed in Additional file 2: Table S7. The pL1 vector contains 5′UTR, ORF1, IGR, ORF2 and 3′UTR of L1, which are all cloned from the pig genome (L1D1). The pL1-CMV is the same as pL1, but the 5′UTR of pig L1 was replaced with the CMV promoter. The vector phL1 is a chimeric vector derived by the CMV promoter, the two ORFs and 3′UTR were from pig L1, and the IGR was from human L1 (99-PUR-RPS-pBlaster1). All vectors contained two selective cassettes (mBlast and Puro) for two-round selections. The schematics of these vectors are listed in Fig. 2a.

### Cell culture

HeLa cells (CCL-2; ATCC, USA) and MEF cells (kindly provided by Dr. Han Wu from Chinese Academy of Medical Sciences) were cultured in DMEM medium supplemented with 10% fetal bovine serum (FBS), 100 U/mL penicillin and 0.1 mg/mL streptomycin. PEF cells (kindly provided by Dr. Kui Li from Chinese Academy of Agricultural Sciences) were grown in DMEM containing 20% FBS, 1x non-essential amino acids, 1 mM sodium pyruvate, and 2 mM l-glutamine. PK15 cells (kindly provided by Dr. Han Wu from Chinese Academy of Medical Sciences) were grown in DMEM containing 10% FBS and 2 mM l-glutamine, 100 U/mL penicillin, and 0.1 mg/mL streptomycin. Culture of cells was maintained in a humidified atmosphere with 5% CO_2_ in air at 37 °C. All cell culture reagents used were purchased from Thermo Fisher Scientific (Waltham, MA, USA). 

### Retrotransposition assay

Retrotransposition assays were performed as described by [50]. Briefly, 3 × 10^5^ HeLa cells or PK15 cells were seeded onto each well of 6-well plates 1 day prior to transfection, and transfected with 3 μg of DNA (plasmid pL1/pL1-CMV/phL1/hL1/mhL1) using the FuGene HD transfection reagent (Promega) (cell confluence > 80% on day of transfection). Then, 48 h after transfection, transfected cells were replated onto T75 flasks and selected in 3 μg/mL puromycin (InvivoGen, San Diego, CA, USA) for HeLa and 4 μg/mL puromycin for PK15 cells. After 5 days of selection, both the HeLa and PK15 cells were selected again in 4 μg/mL blasticidin (InvivoGen) for 10 days. The blasticidin-resistant colonies were then stained with 0.4% Giemsa (Solarbio, Beijing, China) and counted. For the transposition activity assay, at least three independent experiments were performed, and three independent parallel groups were set up for each experiment.

### Promoter activity assay

The promoters activity of 5′UTR from young pig L1 subfamilies and LTRs from ERV6 were tested using the Dual-Luciferase® Reporter Assay System. In short, 3 × 10^5^ HeLa, MEF, PEF, or PK15 cells were seeded onto each well of 6-well plates 1 day prior to transfection and then transfected with 2 μg of plasmid fire luciferase (pGL3-LTR/5’UTR-Luc/pGL3-control/pGL3-enhancer) and Renilla luciferase (pRL-TK) at a 10:1 ratio using the FuGene HD transfection reagent. After 48 h post-transfection, the cells were lysed and harvested. The luciferase activity from the lysed cells was detected according to the protocol of the Dual-Luciferase® Reporter Assay System kit (Promega) with a Modulus™ II Microplate Multimode Reader (Turner Biosystems, Sunnyvale, CA, USA). More than three independent experiments were performed.

### Real-time quantitative PCR

To evaluate the sense and antisense expression profiles of young retrotransposon, including L1D, SINEA, and ERV6, the primers were designed according to the conserved regions of 5′UTR, ORF1, and ORF2 of L1D, SINEA, and LTR, *gag*, *pol*, and *env* of ERV6. Expression levels were measured by real-time qPCR. Primer design for RT and qPCR detection are shown in Fig. 5a. Primer sequences and their genomic coordinates are listed in Additional file 2: Table S8. *GAPDH* was used as an internal control. Total RNAs were isolated from the multiple tissues of three female and three male pigs (Bama, Guangxi Province, China) at 3 months of age, and PK15 and PEF cells by using standard Trizol methods (Invitrogen, Carlsbad, CA, USA). To synthesize the first strand of cDNA, 1 μg of total RNA was reverse-transcribed by using gene-specific primers with the FastQuant RT Kit (with gDNase) (TianGen). The RNA treated with DNase and without RT was used as template of PCR to confirm no DNA contamination. The real-time qPCR was then performed using SYBR Premix Ex Taq II (Tli RNaseH Plus) (TaKaRa) with an Applied Biosystems® 7500 Real-Time PCR System (Applied Biosystems, Foster City, CA, USA).

### Statistical analyses

One-way ANOVA was used to determine differences in clones between groups in the retrotransposition assay using SPSS (version 16.0; Chicago, IL, USA). The LSD method was used for post-test analysis. The frequency difference of sense and antisense TE insertions was compared by using the χ^2^ test. A *p* value < 0.05 was considered to be significant in all analyses.

## Additional files

Additional file 1: The consensus sequences or representative sequences of L1, SINE and ERV. (DOCX 200 kb)

Additional file 2: **Table S1.** Detail information of pig L1 families in the pig genome. **Table S2.** (SINE name and length information after reclassification). **Table S3.** (Detailed information on ERV in the pig genome). **Table S4.** (Composition of interspersed repeats in the pig genome). **Table S5.** (Primers for insertion polymorphism detection of youngest retrotransposons). **Table S6.** (Primers for promoter activity assay of LTR /5′UTR of ERV/ L1). **Table S7.** (Primers for retrotransposon assay of L1). **Table S8.** (Primers for detection of the expression of retrotransposons by RT-qPCR). **Figure S1.** (Comparison and reclassification of SINE transposons derived from tRNA in Repbase libraries by sequence alignment). **Figure S2.** (ERVs were classified into three classes based on the NJ phylogenetic tree). **Figure S3.** (Schematic of the protein structures of full-length ERV6 members in pig genome). **Figure S4.** (Retrotransposon distribution in pig genome and the impact on genes). **Figure S5.** (Primers designed for the youngest retrotransposons insertion polymorphism detection). (DOCX 2580 kb)

## Abbreviations

env: Envelope protein
ERVs: Endogenous retroviruses
gag: Group-specific antigen
IGR: Intergenic region
LINEs: Long interspersed nuclear elements
LTRs: Long terminal repeats
MIR: Mammalian-wide interspersed repeat
Mya: Million years ago
ORF1: Open reading frame 1
pol: Polymerase
RT: Reverse transcription
SINEs: Short interspersed nuclear elements
TEs: Transposable elements
