# Supplementary material for: Retrotransposons evolution and impact on lncRNA and protein coding genes in pigs
Source: Mob DNA. 2019 May 6;10:19. doi: 10.1186/s13100-019-0161-8 (PMC6501411; doi:10.1186/s13100-019-0161-8)
Supplement: Supplementary file 1 — The consensus sequences or representative sequences of L1, SINE and ERV. (DOCX 200 kb) [file 13100_2019_161_MOESM1_ESM.docx]

>L1A1#LINE/L1A

GGAGGGATTAAGATGGCGGAATAGAAGGACTGGAGCTCAACTTCTCTCCTAAAAACAACA

AAATTCACAACTAAAGACTGAGCACTCTTCACCCAAATGGACCGGAAACCTTAAAAAAGA

TACCCTACTCCAGAAGAAAAAGAGGAGGCCACATCAAGAGGTAGGAGGGGCGATTTCAGA

TATAAACAACCCCATACCTCCTGGGTGGGAAGCTCCACAGACTGGAAACTAACTGGTTCA

CAGAGACTCACCTACAGGAGTGAGAGTTCTGAGCCCCACATCAAACTCTCACGTGTGGGG

ATCTGGCACGGGAGAAAGAGCCCCGGAGCATCTGGCATTGAAGGCCAGTGGGGCTTGTGC

GCAGGAGCTCCACGGGACTGGGGGAAACGGAGACCCCATTCTTAAAAGGCGCACACAGAC

TTTCACGTGCACTGGGTCCCAGGGCAAAGCAAAGTCTCCATGGGAATCTGGGTCAAACCT

GACTGCAGTTCTTGGAGGACATCCTGGGAAAACAGGGGTGAATGTGGCTTGTTGTGAGGG

AAGGACATTGAAAGCAAAGCTCTCGGGAATATTCAGCAGCTGCCTTTCTCTGGAGGTGGC

CATTTTGGGAAAATCTGGCCCCACCCATCAGTCAGCTGCTGAGAAGCCCCAGGGCAAACA

ACAACCCAGGTGGGATCACAGCCCCACCCCTCAGTAAACAGGCTACCTAAAGACCCCTCA

GGCACACAGCTGCCTCTAATCCCATCCAGAGACTAAGCCCCACCCACCAGAGGGATTAGA

ATCGGCTCCACCTACCAGGGGGCAGGCATCAGCCCCTCCCATCAGGAAGCCTACAGCAAG

CCCCCATACTGACTTCAGCCACAGGGGGGCAGACATCAGAAGTAAGAGAGGCTACAACTC

TATTATCTGTAAAAAGGTCACCACACCAAAAACCTATAAAAATGAAAAGACAGAGAACTA

TAACTCAGATGAGGGAGAAAGGAAAAACCCCAGAAAAACAGCTAAGCATGAGGAGATTCT

CAGCCTCCAGGAAAAAGACTTTAGACTGTTGATGCTGAAGATGATGCAAGACATTGGAAA

TAAACTGGAGGCAAAGATGGATAACTTACAGGAAACACTGACCAAAGAGATACAAGATAT

AAAACTTAAACAAGAAGAGATGCAAAATACAATAACTGAAATAAAAAATTCACTAGAAGC

AGCTAACAGCAGAATACAGGAGGCAGAAGAACGAATAAGCGAGGTGGAGGACAGATTAGT

GGAAATTACGGATGCAGAACAGAAAAGAGAAAAAAGATTGAAAACAAATGAAGAGAGTCT

CAGAGAACTCTGGGACAACGTTAAACGCACCAACATCCGTATTATAGGGGTGCCAGAAGG

AGAAGAGAGAGAGAAGGAGACAGAAAAAATATTCCAAGAGATAATAGCCGAAAACTTCCC

TAACATGGGGAAGGAATCACTCACTCAAATCCAGGAAGCACAACGAGTACCATATAAAAT

AAACCCAAGGAGGAACACCCCGAGACACATATTAATCAAACTGACCAAAATTAAAGACAA

AGAGAAAATCTTGAAAGCAGCTAGGGAAAAGAAACAAATAACATACAAGGGAACCCCAAT

AAGGTTATCGGCAGATTTTTCAACAGAAACTCTGCAGGCCAGAAGGGAGTGGCATGATAT

ACTTAACGTGATGAAAGGAAAAAACCTCCAACCAAGATTACTCTACCCAGCAAGGCTCTC

ATTCAGATTTGAAGGAGAAATCAAAACCTTCACAGATAAGCAAAAGCTGAGAGAATTCAG

CAACACTAAACCAGCCTTACAACAAATACTAAAGGAACTTCTCTAGGCAGAAAAGAACAA

GAGAAGAAGGAAAGAAAAAAGAGCAGCAAAAACAAATCCAAAGTAATTAATAAAATGGCA

ATAAGAACATACATATCAATAATTACCTTAAATGTTAATGGACTAAACGCCCCAACCAAA

AGACATAGACTGGCTGAATGGATACAAAAACAAGACCCATATATATGCTGTCTTCAAGAG

ACCCACTTCACTTCTAGGGACACATACAAATTGAAAGTGAGAGGATGGAAGAAAATATTT

CATGCAAACGGGGATCAAAAGAAAGCTGGAGTAGCAATACTCATATCAGACAAAATAGAC

TTTAAAATGAAGAATATTTTAAGGGACAAAGAAGGACATTACATAATGATCAAAGGATCA

ATCCAAGAAGATGATATAACAATTTTAAATATCTACGCACCCAACACAGGTTCACCACAA

TATATAAGGCAACTGCTAACAACCTTAAAAGGAGAAATCGACAATAACACAATCATAGTG

GGGGACTTTAACACCCCACTTACAGCAATGGACAGATCAACCAGACAGAAAATCAATAAG

GAAACACAGGCCCTGAATGAAGCATTAAACCAGATGGACTTAATAGATATTTATAGGACA

TTCCATCCAAAAGCAACAGAATACACATTCTTCTCAAGTGCACATGGAACATTCTCTAAG

ATTGATCACATCCTGGGCTACAAATCCAACCTCGTAACTTTAAGAAAATTGAAATCATAT

CAAGCATCTTTTCCGACCACAACGCTATACGACTGGAAATCAACAACAAGAAAAAAACTG

CAAAAAACACAAACACGTGGAGACTCAACAACATGCTACTAAACAACCAATGGATCACTG

AAGAAATCAAAGAGGAAATTAAAAAATACCTAGCAGCAAATGACAACGAAGATACGACAC

TCCAAAACCTATGGGATGCAGCAAAAGCCGTTCTAAGAGGAAAGTTTATAGCAATACAAG

CCCACCTCAGGAAACAAGAAAAAGCTCAAATAAACAAGCTAACTTTACATCTAAAGCAGC

TCGAGAGAGAAGAACAGACAAGACCTAAAGTTAGTAGAAGGAAAGAAATCATAAAGATCA

GAGCAGAAATCAATGAAATAGAAACAAAGAAAACCATAGAAAAGATCAATGAAACGAAAA

GCTGGTTCTTTGAAAAGATCAACAAAATTGATAAACCCCTAGCCAGACTTATCAAGCAAA

AAAGAGAGAGGACTCAAATCAATAAAATTAGAAATGAAAAAGGAGAAGTAACAACGGACA

TCACAGAAATACAAAGGATCATAAGAGACTACTATATGCAACTATATGCCAATAAAATGG

AAAACCTAGAAGAAATGGACAAATTCTTAGAAAAGTACAATCTTCCAAGACTAAACCAAG

ATGAAATAGAAAAGATGAATGGACCCATCACAAGAACTGAAATTGAAACTGTGATTAAAA

AACTTCCAACAAACAAAAGTCCAGGACCAGATGGCTTCACAGGCGAATTCTATCAAACAT

TTAGAGAAGAGCTAACACCTCTCCTTCTGAAACTATTTCAAAAAATTGCAGAGGAAGGGA

TACTCCCAAACTCATTCTATGAGGCCACCATCACCCTGGTACCAAAACCAGACAAAGATT

CCACAAAAAAAGAAAACTACAGGCCAATTTCACTGATGAACATCGATGCAAAAATCCTCA

ACAAAATACTAGCAAACCGCATCCAACAATACATTAAAAGGATTGTACATCATGATCAAG

TGGGATTTATCCCAGGGATGCAAGGGTTCTTCAATATCCGCAAATCCATCAGTGTGATAC

ACCACATTAACAAACTGAAGAATAAAAACCATATGATCCTCTCAATAGACGCGGAAAAAG

CCTTTGACAAAATCCAACACCCATTTCTGATAAAAACCCTTCAGAAAGTGGGCATAACGG

GAACCTACCTCAACATGATAAAGGCCATATATGACAAACCCACAGCGAACATCATTCTCA

ATGGTGAAAAGCTGAAAGAATTCCCGCTGAGATCAGGAACAAGACAAGGATGTCCGCTCT

CGCCACTACTCTTCAACATAGTTCTGGAAGTCCTAGCCACAGCAATCAGAGAAGTAAAAG

AAATAAAAGGAATCCAAATTGGAAAGGAAGAAGTAAAACTATCCTATTTGCAGATGACAT

GATACTATACCTAGAGAATCCTAAAGACTCTACCAGAAAACTGTTAGAGCTCATCCACGA

ATTTGGCAAAGTCGCAGGATACAAAATCAATACACAGAAATCGATGGCATTTCTATACAC

TAACAATGAAAGAGCAGAAAAAGAAATTAGGGAAGCAATCCCGTTTACCATCGCATCCAA

AAGAATAAAATACCTAGGAGTAAACCTACCTAAAGAGACAAAAGACCTGTACTCTGAAAA

CTATAAGCCACTGATGAAAGAAATCAAAGATGACACAAATAGATGGAAAGATATACCATG

CTCGTGGATTGGAAGAGTTAATATTATCAAAATGACTATACTACCTAAGGCAATCTACAG

ATTCAATGCAATCCCTATCAAATTACCAAGGACATTTTTCACAGAACTCGAACAAAATAT

TTTAAAGTTTGTTTGGAAGCACAAAAGACCCAGAATAGCCAAAGACATCCTGAAAAAGAA

AAATGGAGCTGGAGGAATCAGGCTCCCGGACTTCAGACTATACTACAAAGCAACAGTCAT

CAAAACCGCATGGTACTGGCACAAAGACAGAAATATAGATCAGTGGAACAGGATAGAAAG

CCCAGAATTAAACCCACGCACCTACAGCCAACTATCTATGACAAAGGAGGCAAGAATATA

CAATGGAGAAAGGACAGCTTGTTCAATAAGTGGTGCTGGGAAAACTGGACAGCCACATGG

AAAAGAATGAAATTAGAACACTCCCTAACACCATACACAAAAATAAACTCCAAATGGATT

AAAGACCTAGATATAAGACCAGACACTATCAAACTCTTAGAGGAAAACATAGGCCAAACA

CTCTCTGACATAAACGACAGCAACATCTTCTCAGATCCACCTCTCAGAGTATTGACAATA

AAAACAAAAATAAACAAATGGGACCTAATCAAACTTCAAAGTTTCTGCACAGCAAAGGAA

ACCCTAAACAACACAAAAAGACAACCCACAGAATGGGAGAAAATCTTTGCAAGTGAATCG

ACTGACAAGGGATTAATCTCCAAAATTTATAAACACCTTCTGCAGCTCCATACCAAAAAA

ACAAACAACCCCATCAAAAAATGGGCAGAAGATCTAAACAGACAGTTCTCCAAAGAAGAC

ATACAGATGGCCAAGAAACACATGAAAAGATGTTCAACATCACTCATTATTAGAGAAATG

CAAATCAAAACCACTTGAGGTACCACCTTACACCAGCCAGAATGGCCATCATCCAAAAGT

CTACAAACAATAAGTGCTGGAGAGGGTGTGGAGAAAAAGGAACCCTAGTACACTGTTGGT

GGGATTGTAAATTGGTGCAACCACTGTGGAAAGCAGTATGGAGATTCCTCAGAAAACTAA

ACATAGAACTACCATTTGATCCAGCAATCCCACTCCTGGGCATCTATCCAGAGAAAACCA

GACTCGCAAAGACACATGTACTCCAATGTTCATTGCAGCACTATTTACAATAGCCAAGAC

ATGGAAACAACCTAAATGTCCATCGACAGAGGAGTGGATCAAGAAGATGTGGTACATATA

CACAATGGAATATTACTCAGCCATTAAAAAGAACGAAATACCAGCATTTTTAGCAACATG

GATGGACCTAGAAACTATCATGCTAAGTGAAGTCAGCCATACAATGAGACACCAACATCA

AATGCTTTCACTGACATGTGGAATCTGAAAAAAGGACAGACTGAACTTCTTTGCAGAACA

GATGCTGACTCACAGACATTGAAAAACTTATGGTCTCCGGAGGAGACAGTTTGGGGGGTG

GGGGGATGTGCTTGGGCTGTGGGATGGAAATCCTGTGAAATCAGATTGTTATGATCATTA

TACAACTACAGATGTGATAAATTCATTTGAGTAATAAAAAAAAAAAAAAAAAAAAAA

>L1A6#LINE/L1A RepbaseID:HAL1_SSc

GAGAGGATTAAGATGGCGGAATAGAAGGACTGGAGCTCAACTTCTCTCCTAAAAACAACA

AAATTCACAACTAAAAGCTGAGCAATCTCCACCCGAATGGACCGGAAACCTTAAAAAAGA

TACCCTACTCCAGAAGAAAAAGAGGAGGNCACATCAAGAGGTAGGAGGGGCGATTTCGCG

ATATAAACAACCCCATACCTCCCGGGTGGGAAGCTCCACAGACTGAAAACTAACTGGTTC

ACAGAGACTCACCTACAGGAGTGAGAGTTCTGAGCCCCACATCAAACCCTCACGTGCTGG

GATCTGGCACTGGGAGAAAGAGCCCCTGGAGCATCTGGCATTGAAGGCCAGTGGGGCTTG

TGCGCAGGAGCTCCACAGGACTGGGGGAAACGGAGACCCCATTCTTAAAAGGCGCACACG

GACTTTCACGTGCACTGGGTCCCAGGGCAGAGCGAGGTCTCCATAGGAATCTGGGTCAAA

CCTGACTGCAGTTCTTGGAGGACATCCTGGGAAAACAGGGGTGAATGTGGCTTGTTGTGA

GGGAGGGACATTGAAGGCAAAGCTCTCGGGAATATTCAGCAGCTGCCTTTCTCTGGAGGT

GGCCATTTTGGGAAAATCTGGCCCCACCCGTCAGTCAGCGCTGAGAAGCCCCAGGGCAAA

CAACAATCCAGGTGGGATCACAGCCCCGCCCCTCAGTAAACAGGCTGCCTAAAGACCCCT

CAGGCACACAGCTGCCTCTAATCCCATCCAGAGACTAAGCCCCACCCACCAGAGGGATTA

GAATCGGCTCCACCTACCAGTGGGCAGGCATCAGCCCCTCCCATCAGGAAGCCTACAGCA

AGCCCCCATACCGACTTCAGCCACAAGGGGGGCAGACACCAGAAGTAAGAGAGGCTACAA

CTCTAGTATCTGTAAAAAGGTCACCACACCAAAAACCTATAAAAATGAAAAGACAGAGAA

CTATAACTCAGATGAGGGAGAAAGGAAAAACCCCAGAAAATCAGCTAAGCGATGAGGAGA

TTCTCAGCCTCCAGGAAAAAGACTTTAGACTGTTGATGCTGAAGATGATGCAAGACATTG

GAAATAAACTGGAGGCAAAGATGGATAACTTACAGGAAACACTGACCAAAGAGATACAAG

ATATAAAACTTAAACAAGAAGAGATGCAAAATACAATAACTGAAATAAAAAATTCACTAG

AAGCAGCTAACAGCAGAATACAGGAGGCAGAAGAACGAATAAGCGAGGTGGAGGACAGAT

TAGTGGAAATTACGGATGCAGAACAGAAAAGAGAAAAAAGATTGAAAACAAATGAAGAGA

GTCTCAGAGAACTCTGGGACAACGTTAAACGCACCAACATCCGTATTATAGGGGTGCCAG

AAGGAGAAGAGAGAGAGAAGGGGACAGAAAAAATATTCCAAGAGATAATAGCCGAAAACT

TCCCTAACATGGGAAAGGAACCACTCACTCAAATCCAGGAAGCACAACGAGTACCATATA

AAATAAACCCAAGGAGGAATACACCGAGACACATATTAATCAAACTGACCAAAATTAAAG

ACAAAGAGAAAATCTTGAAAGCAGCTAGGGAAAAGAAACAAGTAACATACAAGGGAACCC

CGATAAGGTTATCGGCAGATTTTTCAGCAGAAACTCTGCAGGCCAGAAGGGAGTGGCATG

ATATACTTAACGTGATGAAAGGAAAAAACCTCCAACCAAGATTACTTTACCCAGCAAGGC

TCTCATTCAGATTTGAAGGAGAAATCAAAACCTTCACAGATAAGCAAAAGTTGAGAGAAT

TCAGCAATACTAAACCAGCCTTACAACAAATACTAAAGGAACTTCTCTAGGCAGAAAAGA

AAAGACAGCAACAGGAAACAAAAATTCCACAAATGACAAGGCTCACCAGTAAAGGTATAT

ATACAGTAAAGATACGAAATCATCCATGCACAATTATACCACCAAAATCAGAAATCATGA

GAAGAGGTGGGTACAAATGCAGGACACTGGAGATGAACTTGCAATTAAGAGAACAACAAC

TTAAAACAATCTCATATACATATAGACTCTTATATAACTATATCAAACCAAAAATCTACA

ATTGATACACAAACAAGGAATCTCTGGAGAAGAAATATATCTCATAGTAAATAAGTGCGT

AATCCACCATGAAGGGGGTCATTTGACATCATTCTTGGAATGCTGAAGTCTTCTTAGATC

TGATTCTGATTTCCTCTCCATCAAAGAATTTATGATAATTATAATTAAATAATGCAACTG

TACAATTAAATAATACAGTTCTACAATTGTATTATTAATAACCATTTCTCTCCATGGTAC

AATGTAAGGTCTATAATGTCTTGTTTATCACATCACCTAGAATGGTGTAATGCCTNTATT

GAAGAATCAATAAGATGTAACAAATTGTGAGGACATATTTATATAGTTACACTGTTTTTT

AACCAATTTATGCATTTTATATTTTACTTATTTTCAGAGGGTGTATTATTTTTGTTATTC

TTACCAGTTAAGTTATTCTTTTTATTATGCTATATAAAATATTTAATGGTATATAAGGCT

TTCTTCTTTATAAATTTTAGTTGCATAATATACACAATTTTAATATAATGCTAATTTTTA

AAGAAAAATTGAAATGTAATAGATAATACTAAATAGTAAAGATGAAAGCCATACAAAATG

GGATAAAGCATAGAATAAATTTCCTATTTGTCTCAGCATATTTTCCATTCCACTTCTCCA

GAGGGAGACACTTAATCTGTTTCTTAAGATCCATGATATAATAATCTGTCTGAATGTAAT

TGTGTTTAAATATATGTATTTTATTCTGTAAAAAAAAAATATTCTGTGATAATCTATGTG

GGAAAAGAATGTGAAAGGGAATGGATGTGTGTACATGTATAACTGAATCTCTTTGTTGTA

CAGCAGAAATTATCACAACCTTGTAAATCAACTATACTTCAATAAATTTTTTTTAAAAAA

AATGAAAAAAAAAAAA

>L1A2#LINE/L1A

GGGATTAAGATGGCGGAATAGAAGGACTGGAGCTCAACTTCTCTCCTAAAAACAACAAAA

TTCACAACTAAAGCTGAGCATCTTCACCCAAATGGACCGGAAACCTTAAAAAAGATATCC

TACTCCAGAAGAAAAAGAGGAGGCCACATCAAGAGGTAGGAGGGGTGATTTCACGATATA

AACAACCCCATACCTCCTGGGTGGGAAGCTCCACAGACTGGAAACTAACTGGTTCACAGA

GACTCACCTACAGGAGTGAGAGTTCTGAGCCCCACATCAAACTCTCACGTGTGGGGATCT

GGCACTGGGAGAAAGAGCCCCTGGAGCATCTGGCATTGAAGGCCAGTGGGGCTTGTGTGC

AGGAGCTCCACGGGACTGGGGGAAACGGAGACCCCATTCTTAAAAGGCGCACACAGACTT

TCACGTGCACTGGGTCCCAGGGCAAAGCAAAGTCTCCATGGGAATCTGGGTCAAACCTGA

CTGCAGTTCTTGGAGGACATCCTGGGAAAACAGGGGTGAATGTGGCTTGTTGTGAGGGAA

GGACATTGAAAGCAAAGCTCTCGGGAATATTCAGCAGCATGCCTTTCTCTGGAGGTGGCC

ATTTTGGGAAAATCTGGCCCCACCCATCAGTCAGCGCTGAGAAGCCCCAGGGCAAACAAC

AATCCAGGTGGGATCACAGCCCCACCCCTCAGTAAACAGGCTACCTAAAGACCCCTCAGG

CACACAGCTGCCTCTAATCCCATCCAGAGACTAAGCCCCACCCACCAGAGGGATTAGAAT

CAGCTCCACCTACCAGTGGGCAGGCATCAGCCCCTCCCATCAGGAAGCCTACAGCAAGCC

CCCATACGACTTCAGCCACAAGGGGGGCAGACACCAGAAGTAAGAGAGGCTACAACTCTA

TTATCTGTAAAAAGGTCACCACACCAAAAACCTATAAAAATGAAAAGACAGAGAACTATA

ACTCAGATGAGGGAGAAAGGAAAAACCCCAGAAAATCAGCTAAGCGATGAGGAGATTCTC

AGCCTCCAGGAAAAAGACTTTAGACTGTTGATGCTGAAGATGATGCAAGACATTGGAAAT

AAACTGGAGGCAAAGATGGATAACTTACAGGAAACACTGACCAAAGAGATACAAGATATA

AAACTTAAACAAGAAGAGATGCAAAATACAATAACTGAAATAAAAAATTCACTAGAAGCA

GCTAACAGCAGAATACAGGAGGCAGAAGAACGAATAAGTGAGGTGGAGGACAGATTAGTG

GAAATTACGGATGCAGAACAGAAAAGAGAAAAAAGATTGAAAACAAATGAAGAGAGTCTC

AGAGAACTCTGGGACAAGTTAAACGCACCAACATCCGTATTATAGGGGTGCCAGAAGGAG

AAGAGAGAGAGAAGGGGACAGAAAAAATATTCCAAGAGATAATAGCCAAAACTTCCCTAA

CATGGGAAAGGAACCACTCACTCAAATCCAGGAAGCACAACGAGTACCATATAAAATAAA

CCCAAGGAGGAACACCCCGAGACACATATTAATCAAACTGACCAAAATTAAAGACAAAGA

GAAAATCTTGAAAGCAGCTAGGGAAAAGAAACAAATAACATACAAGGGAACCCCAATAAG

GTTATCGGCAGATTTTTCAGCAGAAACTCTGCAGGCCAGAAGGGAGTGGCATGATATACT

TAACGTGATGAAAGGAAAAAACCTCCAACCAAGATTACTCTACCCAGCAAGGCTCTCATT

CAGATTTGAAGGAGAAATCAAAACCTTCACAGATAAGCAAAAGCTGAGAGAATTCAGCAA

CACTAAACCAGCCTTACAACAAATACTAAAGGAACTTCTATAGGCAGAAAAGAACAAGAG

AAGAAGGAAAGAAAAAAGAGCACAAAAACAAATCCAAAGCAATTAATAAAATGGCAATAA

GAACATACATATCAATAATTACCTTAAATGTTAATGGACTAAACGCCCCAACCAAAAGAC

ATAGACTGGCTGAATGGATACAAAAACAAGACCCATATATATGCTGTCTTCAAGAGACCC

ACTTCACTTCTAGGGACACATACAAATTGAAAGTGAGAGGATGGAAGAAAATATTTCATG

CAAACGGGGATCAAAAGAAAGCTGGAGTAGCAATACTCATATCAGACAAAATAGACTTTA

AAATGAAGAATATTTTAAGGGACAAAGAAGGACATTACATAATGATCAAAGGATCAATCC

AAGAAGAAGATATAACAATTTTAAATATCTACACACCCAACAAGGTTCACCACAATATAT

AAGGCAACTGCTAACAACCTTAAAAGGACAAATCGACAATAACACAATCATAGTGGGGGA

CTTTAACACCCCACTTACAGCAATGGACAGATCAACCAGACAGAAAATCAATAAGGAAAC

ACAGGCCCTGAATGATGCATTAAACCAGATGGACTTAATAGATATTTATAGGACATTCCA

TCCAAAAGCAACAGAATACACATTCTTCTCAAGTGCACATGGAACATTCTCTAAGATTGA

TCATATCCTGGGCTACAAATCCAACCTCAGTAACTTTAAGAAAATTGAAATCATATCAAG

CATCTTTTCCGACCACAACGCTATACGACTGGAAATCAACAACAAGAAAAAAACTGCAAA

AAACACAAACACGTGGAGACTAAACAACATGCTACTAAACAACCAATGGATCACTGAAGA

AATCAAAGAGGAAATTAAAAAATACCTAGCAGCAAATGACAACGAAGATACGACACTCCA

AAACCTATGGGATGCAGCAAAAGCCGTTCTAAGAGGAAAGTTTATAGCAATACAAGCCCA

CCTCAGGAAACAAGAAAAAGCTCAAATAAACAAGCTAACTTTACATCTAAAGCAGCTCGA

GAGAGAAGAACAGACAAGACCTAAAGTTAGTAGAAGGAAAGAAATCATAAAGATCAGAGC

AGAAATCAATGAAATAGAAACAAAGAAAACCATAGAAAAGATCAATGAAACGAAAAGCTG

GTTCTTTGAAAAGATCAACAAAATTGATAAACCCTTAGCCAGACTTATCAAGCAAAAAAG

AGAGAGGACTCAAATCAATAAAATTAGAAATGAAAAAGGAGAAGTAACAACGGACATCAC

AGAAATACAAAGGATCATAAGAGACTACTATATGCAACTATATGCCAATAAAATGGAAAA

CCTAGAAGAAATGGACAAATTCTTAGAAAAGTACAATCTTCCAAGACTAAACCAAGATGA

AATAGAAAAGATGAATGGACCCATCACAAGAACTGAAATTGAAACTGTGATTAAAAAACT

TCCAACAAACAAAAGTCCAGGACCAGATGGCTTCACAGGCGAATTCTATCAAACATTTAG

AGAAGAGCTAACACCTCTCCTTCTGAAACTATTTCAAAAAATTGCAGAGGAAGGGATACT

CCCAAACTCATTCTATGAGGCCACCATCACCCTGGTACCAAAACCAGACAAAGATACCAC

AAAAAAAGAAAACTACAGGCCAATTTCACTGATGAACATCGATGCAAAAATCCTCAACAA

AATACTAGCAAACCACATCCAACAATACATTAAAAGGATTGTACATCATGATCAAGTGGG

ATTTATCCCAGGGATGCAAGGGTTCTTCAATATCCACAAATCCATCAGTGTGATACACCA

CATTAACAAACTGAAGAATAAAAACCATATGATGGAACCTACCTCAACATGATAAAGGCC

ATATATGACAAACCCACAGCAACATCATTCTCAATGGTGAAAAGCTGAAAGAATTCCCAC

TGAGATCAGGAACAAGACAAGGATGTCCGCTCTCGCCACTACTCTTCAACATAGTTCTGG

AAGTCCTAGCCACAGCAATCAGAGAAGTAAAAGAAATAAAAGGAATCCAAATTGGAAAGG

AAGAAGTAAAACTATCACTATTTGCAGATGACATGATACTATACCTAGAGAATCCTAAAG

ACTCTACCAGAAAACTGTTAGAGCTCATCCACGAATTTGGCAAAGTCGCAGGATACAAAA

TAATACACAGAAATCGACGGCATTTCTATACACTAACAATGAAAGAGCAGAAAGAGAAAT

TAGGGAAGCAATCCCGTTTACCATCACATCCAAAAGAATAAAATACCTAGGAGTAAACCT

ACCTAAAGAGACAAAAGACCTGTACTCTGAAAACTATAAGACACTGATGAAAGAAATCAA

AGATGACACAAATAGATGGAAAGATATACCATGCTCTGGATTGGAAGAGTTAATATTATC

AAAATGACTATACTACCTAAGGCAATCTACAGATTCAATGCAATCCCTATCAAATTACCA

AGGACATTTTTCACAGAACTCGAACAAAATATTTTAAAGTTTGTTTGGAAGCACAAAAGA

CCCAGAATAGCCAAAGACATCCTGAAAAAGAAAAATGGAGCTGGAGGAATCAGGCTCCCG

GACTTCAGACTATACTACAAAGCAACAATCATCAAAACTGTATGGTACTGGCACAAAGAC

AGAAATATAGATCAGTGGAACAGGATAGAAAGCCCAGAATTAAACCCACGCACCTACAGC

CAACTCATCTATGACAAAGGAGGCAAGAATATACAATGGAGAAAGGACAGCTTGTTCAAT

AAGTGGTGCTGGGAAAACTGGACAGCCACATGGAAAAGAATGAAATTAGAACACTCCCTA

ACACCATACACAAAAATAAACTCCAAATGGATTAAAGACCTAGATATAAGACCAGACACT

ATCAAACTCTTAGAGGAAAACATAGGCCAAACACTCTCTGACATAAACGACAGCAACATC

TTCTCAGATCCACCTCTTAGAGTATTGACAATAAAAACAAAAATAAACAAATGGGACCTA

TCAAACTTAAAGTTTCTGCACAGCAAAGGAAACCCTAAACAAACAAAAGACAACCCACAG

AATGGGAGAAAATCTTTGCAAGTGAATCAACTGACAAGGGATTAATCTCCAAAATTTATA

AACACCTTCTGCAGCTCCATACCAAAAAAACAAACAACCCCATCAAAAAATGGGCAGAAG

ATCTAAACAGACAATTCTCCAAAGAAGACATACAGATGGCCAAAAAACACATGAAAAGAT

GTTCAACATCACTCATTATTAGAGAAATGCAAATCAAAACCACTTGAGGTACCACCTTAC

ACCAGCCAGAATGGCCATCATCCAAAAGTCTACAAACAATAAGTGCTGGAGAGGGTGTGG

AGAAAAAGGAACCCTAGTACACTGTTGGTGGGATTGTAAATTGGTGCAACCACTGTGGAA

AGCAGTATGGAGATTCCTCAGAAAACTAAACATAGAACTACCATTTGATCCAGCAATCCC

ACTCCTGGGCATCTATCCAGAGAAAACCACACTCGCAAAGACACATGTACTCCAATGTTC

ATTGCAGCACTATTTGCAATAGCCAAGACATGGAAACAACCTAAATGTCCATCGACAGAG

GAGTGGATCAAGAAGAGTGGTACATATACACAATGGAATATTACTCAGCCATTAAAAAGA

ACGAAATACCAGCATTTTTAGCAACATGGATGGACCTAGAAATATCATGCTAAGTGAAGT

CAGCCATACAATGAGACACCAACATCAAATGCTTTCACTGACATGTGGAATCTGAAAAAA

GGACAGACTGAACTTCTTTGCAGAACAGATGCTGACTCACAGACATTGAAAAACTTATGG

TCTCCGGAGGAGACAGTTTGGGGGGTGGGGGGATGTGCTTGGGTTGGGATGGAAATCCTG

TGAAATGATTGTTATGATCATTATACAACTACAGATGTGATAAATTCATTTGAGTAATAA

AAAAAAAAAAAAAAAAA

>L1A3#LINE/L1A

GGGATTAAGATGGCAGAATAGAAGGACTGGAGCTCAACTTCTCTCCTAAAAACAACAAAA

TTACAACTAAAGGCTGAGCAATCTTCAACCAAATGGACGGAAACCTTCAAAAAGATATCC

TACTCCAGAAGACAAAGAGGAGGCCACATCAAGAGGTAGGAGGGGCGATTACATGATATA

AGCAACCCCATACCTCCGGGTGGGAAGCCCCACAGACTGGAAAGTAACTGGTTCACAGAG

ACTCACCTACAGGAGTGAGAGTTCTGAGCCCCACATCAAACTCCCACGTGTGGGGATCTG

GCACTGGGAGAAAGAGCCCCTGGAGCATCTGGCATTGAAGGCCAGTGGGGCTTGTGCCAG

GAGCTCCAGGGACTGGGGGAAATGGAGACCCCATTCTTAAAAGGCGCACACAGACTTTCA

CGTGCACTGGGTCCCAGGGCAAAGCAAAGTCTCCATAGGAATCTGGGTCAAACCTGACTG

CAGTTCTTGGAGGACCTCCTGGGAAAACAGGGGTGAATGTGGCTTGTTGTGGGGGAAGGA

CATTGGAAGCAAAGCTCTTGGGAATATTCAGCAGCTGCCTTTCTCTGGAGGTGGCCATTT

TGGGAAAATCTGGCCCCACCCATCAGTGCTGAGAAGCCCCAGGGCAAACAACAATCCAGG

TGGGATCACAGCCCCACCCCTCAGTAAACAGGCTGCCTAAAGACCCCCCAGGCACACAGC

TGCCTCTAATCCCATCCAGAGACAAAGCCCCACCCACCAGAGGGATAGGAATCAGCTCCA

CCTACCAGTGGGCAGGCATCAGTCCCTCCCATCAGGAAGCCTACAGCAAGCCCCCATACC

AACTTCAGCCACAAGGGGGGCAGACACCAGAAGTAAGAGAGGCTACAACTCTATTATCTG

TAAAAAGGTCACCACACCAAAAACCTATAAAAATGAAAAGACAGAGAACTATAACTCAGA

TGAGGGAGAAAGAAAAAACCCCAGAAAACAGCTAAGTGATCAGGAGATTCTCAGCCTCCA

GGAAAAAGACTTTAGACTGTTGATGCTGAAGATGATGCAAGACATTGGAAATAAACTGGA

GGCAAAGATGGATAACTTACAGGAAACACTGAGCAAAGAGATACAAGATATAAAACTTAA

ACAAGAAGAGATGCAAAATACAATAACTGAAATAAAAAATTCACTAGAAGCAGCTAACAG

CAGAATACAGGAGGCAGAAGAACGAATAAGCGAGGTGGAGGACAGATTAGTGGAAATTAC

GGATGCAGAACAGAAAAGAGAAAAAAGATTGAAAACAAATGAAGAGAGTCTCAGAGAACT

CTGGGACAAGTTAAAGCACCAACATCCATATTATAGGGGTGCCAGAAGGAGAAGAGAGAG

AGAAGGGGACAGAAAAAATATTCCAAGAGATAATAGCCGAAAACTTCCCTAACATGGGAA

AGGAACCACTCACTCAAATCCAGGAAGCACAACGAGTACCATATAAAATAAACCCAAGGA

GGAATACCCCGAGACACATATTAATCAAACTGACCAAAATTAAAGACAAAGAGAAAATCT

TGAAAGCAGCTAGGGAAAAGAAACAAATAACATACAAGGGAACCCCATAAGGTTATCGGC

AGATTTTTCAGCAGAAACTCTGCAGGCCAGAAGGGAGTGGCATGATATACTTAACGTGAT

GAAAGGAAAAAACCTCCAACCAAGATTACTTTACCCAGCAAGGCTCTCATTCAGATTTGA

AGGAGAAATCAAAACCTTCACAGATAAGCAAAAGCTAAGAGAATTCAGCAACACTAAACC

AGCTTTACAACAAATACTAAAGGAACTTCTCTAGGCAGAAAAGAAAAGGCGCAACAGAAA

CAAAAATACCACAAATGACAAGGCTCACCAGTAAAGGTATATATACAGTAAAGATAGAAA

TCATCCATGCACAATTATGCCACCAAAATCAGAAATCATGAGAAGAGGTGGGTACAAATG

CAGGACACTGGAGATGCACTTGCAATTAAGAGACCAACAACTTAAAACAATCTCATATAT

ATATAGACTCTTATATCAAAACTTCAGAATAACTGCAAACCAAAAATCTACAATTGATAC

ACAAACAAATAAGAAAAATCAACTCAAATACAACACTAAAGATAGTCATCAAACCACAAG

AGGAGAGAACAAGAGAAGAAGGGAAGAAAAAAGAGCAACAAAAACAAATCCAAAGCAATT

AATAAAATGGCAATAAGAACATACATATCAATAATTACCTTAAATGTTAATGGACTAAAT

GCCCCAACCAAAAGACATAGACTGGCTGAATGGATACAAAAACAAGACCCATATATATGC

TGTCTTCAAGAGACCCACTTCACTTCTAGGGACACATACAAATTGAAAGTGAGAGGATGG

AAGAAAATATTCCATGCAAACGGGAATCAAAAGAAAGCTGGAGTAGCAATACTCATATCA

GACAAAATAGACTTTAAAATGAAGAATATTTTAAGGGACAAGGAAGGTCACTACATAATG

ATCAAAGGATCAATCCAAGAAGAAGATATAACAATTTTAAATATCTACGCACCCAACATA

GGTTCACCACAATATATAAGGCAACTGCTAACAACCTTAAAAGGACAAATCGACAATAAC

ACAATAATAGTGGGGGACTTTAACACCCCACTTACAGCAATGGACAGATCATCCAGACAG

AAAATCAATAAGGAAACACAGGCCCTGAATGAAGCATTAGACCAGATGGACTTAATAGAT

ATTTATAGGACATTCCATCCAAAAGCAACAGAATACACATTCTTCTCAAGTGCACATGGA

ACATTCTCTAAGATTGATCACATCCTGGGCTACAAATCCAACCTCGGTAACTTTAAGAAA

ATTGAAATCATATCAAGCATCTTTTCCGACCACAACGCTATAGACTGGAAATCAACAACA

AGAAAAAAACTGCAAAAAACACAAACACGTGGAGACTAAACAACATGCTACTAAACAACC

AATGGATCACTGAAGAAATCAAAGAGGAAATTAAAAAATACCTAGAAGCAAATGACAACA

AAGATACGACACTCCAAAACCTATGGGATGCAGCAAAAGCCGTTCTAAGAGGAAAGTTTA

TAGCAATACAAGCCCACCTCAGGAAACAAGAAAAAGCTCAAATAAACAAGCTAACTTTAC

ATCTAAAGCAGCTGAGAGAGAAGAACAGACAAGACCTAAAGTTAGTAGAAGGAAAGAAAT

CATAAAGATCAGAGCAGAAATCAATGAAATAGAAACAAAGAAAACCATAGAAAAGATCAA

TGAAACGAAAAGCTGGTTCTTTGAAAAGATCAACAAAATTGATAAACCCTTAGCCAGACT

TATCAAGAAAAAAAGAGAGAGGACTCAAATCAATAAAATTAGAAATGAAAAAGGAGAAGT

AACAACGGACATCACAGAAATACAAAGGATCATAAGAGACTACTATATGCAACTATATGC

CAATAAAATGGAAAACCTAGAAGAAATGGACAAATTCTTAGAAAAGTACAATCTTCCAAG

ACTAAACCAAGATGAAATAGAAAAGATGAATGGACCAATCACAAGAACTGAAATTGAAAC

TGTGATTAAAAAACTTCCAACAAACAAAAGTCCAGGACCAGATGGCTTCACAGGCGAATT

CTATCAAACATTTAGAGAAGAGCTAACACCTATCCTTCTGAAACTATTTCAAAAAATTGC

AGAGGAAGGGATACTCCCAAACTCATTCTATGAGGCCACCATCACCCTGATACCAAAACC

AGACAAAGATACCACAAAAAAAAGAAAACTACAGGCCAATTTCACTGATGAACATGATGC

AAAAATCCTCAACAAAATACTAGCAAACCGCATCCAACAATACATTAAAAGGATTGTACA

TCATGATCAAGTGGGATTTATCCCAGGGATGCAAGGGTTCTTCAATATCCACAAATCCAT

CAGTGTGATACACCACATTAACAAACTGAAGAATAAAAACCATATGATCCTCTCAATAGA

TGCAGAAAAAGCCTTTGACAAAATCCAACACCCATTTCTGATAAAAACCCTTCAGAAAGT

GGGCATAGAGGGAACCTACCTCAACATAATAAAGGCCATATATGACAAACCCACAGCAAC

ATCATTCTCAATGGTGAAAAGCTGAAAGAATTCCCGCTGAGATCAGGAACAAGACAAGGA

TGTCCGCTCTCGCCACTACTCTTCAACATAGTTTTGGAAGTCCTAGCCACAGCAATCAGA

GAAGTAAAAGAAATAAAAGGAATCCAAATTGGAAAGGAAGAAGTAAAACTATCACTATTT

GCAGATGACATGATACTATACCTAGAGAATCCTAAAGACTCTACCAGAAAACTGTTAGAG

CTCATCCATGAATTTGGCAAAGTCGCAGGATACAAAATTAATACACAGAAATCGACGGCA

TTTCTATATACTAACAATGAAAGATCAGAAAGAGAAATTAGGGAAGCAATCCCATTTACC

ATCCATCCAAAAGAATAAAATACCTAGGAGTAAACCTACCTAAAGAGACAAAAGACCTGT

ACTCTGAAAACTATAAGACACTGATGAAAGAAATCAAAGATGACACAAATAGATGGAAAG

ACATACCATGCTCTTGGATTGGAAGAGTCAATATTATCAAAATGACTATACTACCCAAGG

CAATCTACAGATTCAATGCAATCCCTATCAAATTACCAAGGACATTTTTCACAGAACTCG

AACAAAATATTTTAAAGTTTGTTTGGAAGCACAAAAGACCCAGAATAGCCAAAGACATCC

TGAAAAAGAAAAATGGAGCTGGAGGAATCAGGCTCCCGACTTCAGACTATACTACAAAGC

AACAGTCATCAAAACCATATGGTACTGGCACAAAGACAGAAATATAGATCAGTGGAACAG

GATAGAAAGCCCAGAATTAAACCCACGCACCTACAGCCAACTAATCTATGACAAAGGAGG

CAAGAATATACAATGGAGAAAAGACAGCCTGTTCAATAAGTGGTGCTGGGAAAACTGGAC

AGCCACATGGAAAAGAATGAAATTAGAACACTCCCTAACACCATACACAAAAATAAACTC

AAAATGGATTAAAGACCTAGATATAAGACCAGACACTATAAAACTCTTAGAGGAAAACAT

AGGCCAAACACTCTCTGACATAAACGACAGCAACATCTTCTCAGATCCACCTCTTAGAGT

AATGACAGTAAAAACAAAAATAAACAAATGGGACCTAATCAAACTTAAAAGTTTCTGCAC

AGCAAAGGAAACCCTAAACAAAACGAAAAGACAACCCACAGAATGGGAGAAAATCTTTGC

AAATGAATCAACTGACAAGGGATTAATCTCCAAAATTTATAAACACCTTCTGCAGCTCAT

ACCAAAAAAACAAACAACCCCATCAAAAAATGGGCAGAAGATCTAAACAGACAGTTCTCC

AAAGAAGACATACAGATGGCCAAAAAACACATGAAAAGATGTTCAACATCACTCATTATT

AGAGAAATGCAAATCAAAACCACTATGAGGTACCACCTTACACCAGCCAGAATGGCCATC

ATCAAAAAGTCTACAAACAATAAGTGCTGGAGAGGGTGTGGAGAAAAAGGAACCCTATTA

CACTGTTGGTGGGATTGTAAATTGGTGCAACCACTGTGGAAAACAGTATGGAGATTCCTC

AGAAAACTAAAAATAGAACTACCATTTGATCCAGCAATCCCACTCCTGGGCATCTATCCA

GAGAAAACCATGACTCGCAAAGACACATGTACTCCAATGTTCATTGCAGCACTATTTCAA

TAGCCAAGACATGGAAACAACCTAAATGTCCATCGACAGAGGAGTGGATCAAGAAGATGT

GGTACATATACACAATGGAATATTACTCAGCCATTAAAAGGAAGAAATACCGGCATTTTT

AGCAACATGGATGGACCTAGAAATTATCATGCTAAGTGAAGTCAGCCAACAATGAGACAC

CAACATCAAATGCTTTCACTGACATGTGGAATCTGAAAAAAGGACAGAATGAACTTCTTT

GCAGAACAGATACTGACTCACAGACTTTGAAAAACTTATGGTTTCCAAAGGAGACAGTTT

GGGGGGTGGGGGGATGTGCTGGGGTTGTGGGATGGAAATCCTATAAAATTGGATTGTGAT

GATCATTGTACAACTATAAATGTAATAAATTCATTGAGTAATAAAAAAAAAAAAAAAAAA

>L1A4#LINE/L1A

GGAGATGGGATTAAGATGGCAGAATAGAAGGACTGGAGCTCAACTTCTCTCCTAAAAACA

ACAAAATTACAACCAAATGCTGAGCAATCTTCAACCAAATGGACTGGAAACTTTCAAAAA

GATATCCTACTCCAGAAGACAAAGAGGAGGCCACATCAAGAGGTAGGAGGGGCGATTATA

TGATATAAGCAACCCCATACCTCCCAGGTGGGAAGCCCCACAGACTGGAAAGTAACTGTA

TCACAGAGACTCACCTACAGGAGTGAGAGTTCTGAGCCCCACATCAAATTCCCATGCCTG

GGGATCTGGCATTGGGAGAAAGAGCCCCTGGAGCATCTGGCATTGAAGGCCAGTGGGGCT

TGTGTGCAGGAGCTCCACGGGACTGGGGGAAATGGAGACCCCATTCTTAAAAGGTGCACA

CAGACTTTCACGTGCACTGGGTCCCAGGGCAAAGCAAAGTCTCCATAGGAATCTGGGTCA

GACCTGACTGCAGTTCTTGGAGGACCTCCTGGGAAAACAGGGGTGAATGTGGCTTGTTGT

GGGGGAAGGACATTGGAAGCAAAGCTCTTGGGAATATTCATCAGCATGTTTTCTCTGGAG

GTGGCCATTTTGGGAAAATCTGGCCCCACCCATCAGTGCTGAGAAGCCCCAGGCCAAACA

ACAATCCAGGTGGGATCACAGCCCCACCCATCAGTAAACAGGCTGCCTAAAGACCCCCCA

GGCACACAGCCACCTCTAATCTCACCCAGAGACAAAGCCCCACCCACCAGAGGGATAAGA

ATCAGCTCCACCTACCAGTGGGCAGGCATCAGTCCCTCCCATCAGGAAGCCTACAGCAAG

CCCCCATACCAACTTCAGCCACAAGGGGGGCAGACACCAGAAGTAAGAGAGGCTACAACT

CTATTTCTGTAAAAAGGTCACCACACCAAAAACCTATAAAAATGAAAAGACAGAGAACTA

TAACTCAGATGAGGGAGAAAGAAAAAACCCAGAAAAACAGCTAAGTGATCAGGAGATTCT

CAGCCTCCAGGAAAAAGACTTTAGACTGTTGATGCTGAAGATGATGCAAGACATTGGAAA

TAAACTGGAGGCAAAGATGGATAACTTACAGGAAACACTGAGCAAAGAGATACAAGATAT

AAAACTTAAGCAAGAAGAGATGCAAAATACAATAACTGAAATAAAAAATTCACTAGAAGC

AGCCAACAGCAGAATACAGGAGGCAGAAGAACGAATAAGCGAGGTGGAGGACAGATTAGT

GGAAATCACGGATGTGGAACAGAAAAGAGAAAAAAGATTGAAAACAAATGAAGAGAGTCT

CAGAGAACTCTGGGACAATGTTAAACGCACCAACATCCATATTATAGGGGTGCCAGAAGG

AGAAGAGAGAGAGAAAGGGACAGAAAAAATATTCAAGAGATAATAGCCGAAAACTTCCCT

AACATGGGAAAGGAACCACTCACTCAAATCCAGGAAGCACAATGAGTACCATATAAAATA

AACCCAAGGAGGAACACCCCGAGACACATATTAATCAAACTGACCAAAATTAAAGACAAA

GAGAAAATATTGAAAGCAGCTAGGGAAAAGAAACAAATAACATACAAGGGAACCCCAATA

AGGTTATCGGCAGATTTTTCAGCAGAAACTCTGCAGGCCAGAAGGGAGTGGCATGATATA

CTTAACGTGATGAAAGGAAAAAACCTCCAACCAAGATTACTTTACCCAGCAAGGCTCTCA

TTCAGATTTGAAGGAGAAATCAAAAGCTTCACAGATAAGCAAAAGCTAAGAGAATTCAGC

AACACTAAACCAGCTTTACAACAAATACTAAAGGAACTTCTCTAGGCAGAAAAGAAAAGG

CGCAACAAGAAACAAAAATACCACAAATGACAAGGCTCACCAGTAAAGGTATATATACAG

TAAAGATAGAAATCATCCATGCACAATTATGCCACCAAAATCAGAAATCATGAGAAGAGG

AGGGTACAAATGCAGGACACTGGAGATGCACTTGCAATTAAGAGACCAACAACTTAAAAC

AATCTCATATATATATATAAATCTATATCAAAACTTCAGAATAACTGCAAACCAAAAATC

TACAATTGATACACAAACAAATAAGAAAAATCAACTCAAATACAACACTAAAGATAGTCA

TCAAACCACAAGAGGAGAGAACAAGAGAAGAAGGGAAGAAAAAAGAGCAACAAAAACAAA

TCCAAAGCAATTAATAAAATGGCAATAAGAACATACATATCAATAATTACCTTAAATGTT

AATGGACTAAATGCCCCAACCAAAAGACATAGACTGGCTGAATGGATACAAAAACAAGAC

CCATATATATGCTGTCTTCAAGAGACCCACTTCACTTCTAGGGACACATACAAATTGAAA

GTGAGAGGATGGAAGAAAATATTCCATGCAAACGGGAATCAAAAGAAAGCTGGAGTAGCA

ATACTCATATCAGACAAAATAGACTTTAAAATGAAGAATATTTTAAGAGACAAGGAAGGC

ACTACATAATGATCAAAGGATCAATCCAAGAAGAAGATATAACAATTTTAAATATCTACG

CACCCAACATAGGTTCACCACAATATATAAGGCAACTGCTAACAACCTTAAAAGGACAAA

TCGACAATAACACAATAATAGTGGGGGACTTTAACACCCCACTTACAGCAATGGACAGAT

CATCCAGACAGAAAATCAATAAGGAAACACAGGCCCTGAATGAAGCATTAGACCAGATGG

ACTTAATAGATATTTATAGGACATTCCATCCAAAAGCAACAGAATACACATTCTTCTCAA

GTGCACATGGAACATTCTCTAAGATTGATCACATCCTGGGCTACAAATCAAGCCTTGGTA

ACTTTAAGAAAATTGAAATCATATCAAGCATCTTTTCTGACCACAACGCTATATGACTGG

AAATCAACAACAAGAAAAAAACTGCAAAAAACACAAACACGTGGAGACTAAACAACATGC

TACTAAACAACCAATGGATCACTGAAGAAATCAAAGAGGAAATTAAAAAATACCTAGAAG

CAAATGACAACAAAGATACGACACTCCAAAACCTATGGGATGCAGCAAAAGCGTTCTAAG

AGGAAAGTTTATAGCAATACAAGCCCACCTCAGGAAACAAGAAAAAGCTCAAATAAACAA

GCTAACTTTACATCTAAAGCAGCTAGAGAGAGAAGAACAGACAAGACCTAAAGTTAGTAG

AAGGAAAGAAATCATAAAGATCAGAGCAGAAATCAATGAAATAGAAACAAAGAAAACCAT

AGAAAAGATCAATGAAACAAAAGCTGGTTCTTTGAAAAGATCAACAAAATTGATAAACCC

TTAGCCAGACTTATCAAAAAAAAAAGAGAGAGGACTCAAATCAATAAAATTAGAAATGAA

AAAGGAGAAGTAACAATGGACATCACAGAAATACAAAGGATCATAAGAGACTACTATATG

CAACTATATGCCAATAAAATGGAAAACCTAGAAGAAATGGACAAATTCTTAGAAAAGTAC

AATCTTCCAAGACTAAACCAAGATGAAATAGAAAAGATGAATGGACCAATCACAAGACTG

AAATTGAAACTGTGATTAAAAAACTTCCAACAAACAAAAGTCCAGGACCAGATGGCTTCA

CAGGCGAATTCTATCAAACATTTAGAGAAGAGCTAACACCTATCCTTCTGAAACTATTCC

AAAAAATTGCAGAGGAAGGGACACTCCCAAACTCATTCTATGAGGCCACCATCACCCTGA

TACCAAAACCAGACAAAGATACCACAAAAAAAAGAAAACTACAGGCCAATTTCACTGATG

AACATCGATGCAAAAATCCTCAACAAAATACTAGCAAACCGATCCAACAATACATTAAAA

GGATTGTACATCATGATCAAGTGGGATTTATCCCAGGGATGCAAGGGTTCTTCAATATCC

ACAAATCCATCAGTGTGATACACCACATTAACAAACTGAAGAATAAAAACCATATGATCC

TCTCAATAGATGCAGAAAAAGCCTTTGACAAAATCCAACACCCATTTCTGATAAAAAACC

CTTCAGAAAGTGGGCATAGAGGGAACCTACCTCAACATAATAAAGGCCATATATGACAAA

CCCACAGCTAACATCATTCTCAATGGTGAAAAGCTGAAAGAATTCCCGCTGAGATCAGGA

ACAAGACAAGGATGTCCGCTCTCACCACTACTATTCAACATAGTTTTGGAAGTCCTAGCC

ACAGCAATCAGAGAAGTAAAAGAAATAAAAGGAATCCAAATTGGAAAGGAAGAAGTAAAA

CTATCACTATTTGCAGATGACATGATACTATACCTAGAGAATCCTAAAGACTCTACCAGA

AAACTGTTAGAGCTCATCCATGAATTTGGCAAAGTTGCAGGATACAAAATTAATACACAG

AAATGACGCATTTCTATATACTAACAATGAAAGATCAGAAAGAGAAATTAGGGAAGCAAT

CCCATTTACCATCACATCCAAAAGAATAAAATACCTAGGAGTAAACCTACCTAAAGAGAC

AAAAGACCTGTACTCTGAAAACTATAAGACACTGATGAAAGAAATCAAAGATGACACAAA

TAGATGGAAAGACATACCATGCTCTTGGATTGGAAGAGTCAATATTATCAAAATGACTAT

ACTACCTAAGGCAATCTACAGATTCAATGCAATCCCTATCAAATTACCAAGGACATTTTT

CACAGAACTCGAACAAAATATTTTAAAGTTTGTTTGGAAGCACAAAAGACCCAGAATAGC

CAAAGACATCCTGAAAAAGAAAAATGGAGCTGGAGGAATCAGGCTCCCTGACTTCAGACT

ATACTACAAAGCAACAATCATCAAAACCATATGGTACTGGCACAAAGACAGAAATATAGA

TCAGTGGAACAGGATAGAAAGCCCAGAATTAAACCCACGCACCTACAGTCAACTAATCTA

TGACAAAGGAGGCAAGAATATACAATGGAGAAAAGACAGCCTGTTCAATAAGTGGTGCTG

GGAAAACTGGACAGCCACATGGAAAAGAATGAAATTAGAACACTCCCTAACACCATACAC

AAAAATAAACTCAAAATGGATTAAAGACCTAGATATAAGACCAGAACTATAAAACTCTTA

GAGGAAAACATAGGCCAAACACTCTCTGACATAAACGACAGCAACATCTTCTCAGATCCA

CCTCTTAGAGTAATGACAGTAAAAACAAAAATAAACAAATGGGACCTAATTAAACTTAAA

AGTTTCTGCACAGCAAAGGAAACCCTAAACAAAACGAAAAGACAACCCACAGAATGGGAG

AAAATCTTTGCAAATGAATCGACTGACAAGGGATTAATCTCCAAAATTTATAAACACCTT

CTGCAGCTCAATACCAAAAAAACAAACAACCCCATCAAAAAATGGGCAGAAGATCTAAAC

AGACAATTCTCCAAAGAAGACATACAGATGGCCAAAAAACACATGAAAAGATGTTCAACA

TCACTCATTATTAGAGAAATGCAAATCAAAACCACTATGAGGTACCACCTTACACCAGCC

AGAATGGCCATCATCAAAAAGTCTACAAACAATAAGTGCTGGAGAGGGTGTGGAGAAAAG

GAACCCTATTACACTGTTGGTGGGAATGTAAATTGGTGCAACCACTGTGGAAAACAGTAT

GGAGATTCCTCAGAAAACTAAAAATAGAACTACCATTTGATCCAGCAATCCCACTCCTGG

GCATCTATCCAGAGAAAACCAGACTGCAAAGACACATGTACTCCAATGTTCATTGCAGCA

CTATTTGCAATAGCCAAGACATGGAAACAACCTAAATGTCCATCGACAGAGGAGTGGATC

AAGAAGATGTGGTACATGGAGTTCCCGTCGTGGCGCAGTGGTTAACGAATCCGACTAGGA

ACCATGAGGTTGCGGGTTCGATCCCTGGCCTTGCTCAGTGGGTTAAGGATCTGGCATTGC

CGTGAGCTGTGGTGTAGGTTGCAGACGCGGCTCGGATCCCGCGTTGCTGTGGCTCTGGCG

TAGGCCGGTGGCTACAGCTCCGATTAGACCCCTAGCCTGGGAACCTCCATATGCCCAGGA

GCGGCCCAAGAAAAGCAAAAAACAAAAAGACAAAAAAAAAAAAAAAAAAA

>L1A5#LINE/L1A

GGGATTAAGATGGCGAATAGAAGGACTGGAGCTCAACTTCTCTCCTAAAAACAACAAAAT

TTACAACCAAATGCTGAGCAATCTTCAACCAAATGGACTGGAAACTTTCAAAAAGATATC

CTACTCCAGAAGACAAAGAGGAGGCCACATCAAGAGGTAGGAGGGGTGATTACTGATATA

AGCAACCCCATACCTCCCGGTGGGAAGCCCCACAGACTGGAAAGTAACTGTTCACAGAGA

CTCACCTACAGGAGTGAGAGTTCTGAGCCCCACATCAAACTCCCACGGTGGGGATCTGGC

ACTGGGAGAAAGAGCCCCCGGAGCATCTGGCATTGAAGGCCAGTGGGGCTTGTGTGCAGG

AGCTCCACAGGACTGGGGGAAATGGAGACCCCATTCTTAAAGGCACACACAGACTTTCAC

GTGCACTGGGTCCCAGGGCAAAGCAAAGTCTCCATAGGAATCTGGGTCAGACCTGACTGC

AGTTCTTGGAGGACCTCCTGGGAAAACAGGGGTGAATGTGGCTTGTTGTGGGGGAAGGAC

ATTGGAAGCAAAGCTCTTGGGAATATTCATCAGCTGCCTTTCTCTGGAGGTGGCCATTTT

GGGAAAATCTGGCCCCACCCATCAGTGCTGAGAAGCCCCAGGCCAAACAACAATCCAGGT

GGGATCACAGCCCCACCCATCAGTAAACAGGCTGCCTAAAGACCCCCCCAGGCACACAGC

CCCTCTAATCTCACCCAGAGACAAAGCCCCACCCACCAGAGGGATAGGAATCAGCTCCAC

CTACCAGTGGGCAGGCATCAGTCCCTCCCATCAGGAAGCCTACAGCAAGCCCCCATACCA

ACTTCAGCCACAAGGGGGGCAGACACCAGAAGTAAGAGAGGCTACAACTCTATTATCTGT

AAAAAGGTCACCACACCAAAAACCTATAAAAATGAAAAGACAGAGAACTATAACTCAGAT

AAGGGAGAAAGAAAAAAACCCCAAAAAAACAGCTAAGTGATCAGGAGATTCTCAGCCTCC

AGGAAAAAGACTTTAGACTGTTGATGCTGAAGATGATGCAAGACATTGGAAATAAACTGG

AGGCAAAGATGGATAATTTACAGGAAACACTGAGCAAAGAGATACAAGATATAAAATTAA

GCAAGAAGAGATGCAAAATACAATAACTGAAATAAAAAATTCATTAGAAGCAGCCAACAG

CAGAATACAGGAGGCAGAAGAAGAATAAGGAGGTGGAGGACAGATTAGTGGAAATCACGA

TGCAGAACAGAAAAGAGAAAAAAGATTGAAAACAAATGAAGAGAGTCTCAGAGAACTCTG

GGACAATGTTAAATGCACCAACATCCATATTATAGGGGTGCCAGAAGGAGAAGAGAGAGA

GAAAGGGACAGAAAAAATATTCAAGAGATAATAGCCAAAAACTTCCCTAACATGGGAAAG

GAACCACTCACTCAAATCCAGGAAGCACAACGAGTACCATATAAAATAAACCCAAGGAGG

AACACCCCGAGACACATATTAATCAAACTGACCAAAATTAAAGACAAAGAGAAAATCTTG

AAAGCAGCTAGGGAAAAGAAACAAATAACATACAAGGGAACCCCATAAGGTTATCAGCAG

ATTTTTCAGCAGAAACTCTGCAGGCCAGAAGGGAGTGGCATGATATACTTAAGTGATGAA

AGGAAAAAAACCTCCAACCAAGATTACTTTACCCAGCAAGGCTCTCATTCAGATTTGAAG

GAGAAATCAAAAGCTTCACAGATAAGCAAAAGCTAAGAGAATTCAGCAACACTAAACCAG

CTTTACAACAAATACTAAAGGAACTTCTCTAGGCAGAAAAGAAAAGGCCACAACAGAAAC

AAAAATACCACAAATGACAAGGCTCACCAGTAAAGGTATATATACAGTAAAGATAGAAAT

CATCCATGCACAATTATGCCACCAAAATCAGAAATCATGAGAAGAGGAGGGTACAAATGC

AGGACACTGGAGATGCACTTGCAATTAAGAGACCAACAACTTAAAACAATCTCTTATATA

TATATAGACTTATATCAAAACTTCAGAATAACTGCAAACCAAAAATCTACAATTGATACA

CAAACAAATAAGAAAAATCAACTCAAATACAACACTAAAGATAGTCATCAAACCACAAGA

GGAGAGAACAAGAGAAGAAGGGAAGAAAAAAGAGCAACAAAAACAAATCCAAAGCAATTA

ATAAAATGGCAATAAGAACATACATATCAATAATTACCTTAAATGTTAATGGACTAAATG

CCCCAACCAAAAGACATAGACTGGCTGAATGGATACAAAAACAAGACCCATATATATGCT

GTCTTCAAGAGACCCACTTCACTTCTAGGGACACATACAAATTGAAAGTGAGAGGATGGA

AGAAAATATTCCATGCAAAGGGAATCAAAAGAAAGCTGGAGTAGCAATACTCATATCAGA

CAAAATAGACTTTAAAATGAAGAATATTTTAAGGGACAAGGAAGGTCACTACATAATGAT

CAAAGGATCAATCCAAGAAGAAGATATAACAATTTTAAATATCTATGCACCCAACATAGG

TTCACCACAATATATAAGGCAACTGCTAACAACCTTAAAAGGACAAATCGACAATAACAC

AATATAGTGGGGGACTTTAACACCCCACTTACAGCAATGGACAGATCATCCAGACAGAAA

ATCAATAAGGAAACACAGGCCCTGAATGAAGCATTAGACCAGATGGACTTAATAGATATT

TATAGGACATTCCATCCAAAAGCAACAGAATACACATTCTTCTCAAGTGCACATGGAACA

TTCTCTAAGATTGATCACATCCTGGGCTACAAATCAAACCTCGGTAACTTTAAGAAAATT

GAAATTATCAAGCATCTTTTCTGACCACAATGCTATATGACTGGAAATCAACAACAAGAA

AAAAACTGCAAAAAACACAAACACGTGGAGACTAAACAACATGCTACTAAACAACCAATG

GATCACTGAAGAAATCAAAGAGGAAATTAAAAAATACCTAGAAGCAAATGACAACAAAGA

TACGACACTCCAAAACCTATGGGATGCAGCAAAAGCGTTCTAAGAGGAAAGTTTATAGCA

ATACAAGCCCACCTCAGGAAACAAGAAAAAGCTCAAATAAACAAGCTAACTTTACATCTA

AAGCAGCTAGAGAGAGAAGAACAGACAAGACCTAAAGTTAGTAGAAGGAAAGAAATCATA

AAGATCAGAGCAGAAAAGCTGGTTCTTTGAAAAGATCAACAAAATTGATAAACCCTTAGC

CAGACTTATCAAGAAAAAAAAGAGAGAGGACTCAAATCAATAAAATTAGAAATGAAAAAG

GAGAAGTAACAACGGACATCACAGAAATACAAAGGATCATAAGAGACTACTATATGCAAC

TATATGCCAATAAAATGGAAAACCTAGAAGAAATGGACAAATTCTTAGAAAAGTACAATC

TTCCAAGACTAAACCAAGATGAAATAGAAAAGATGAATGGACCAATCACAAGAACTGAAA

TTGAAACTGTGATTAAAAAACTTCCAACAAAATAGAGAGACCCTGACAAATTGCAGAGGA

AGGGAACTCCCAAATGAGGCCACCATCACCCTGATACCAAAACCAGACAAAGATACCACA

AAAAAAGAAAACTACAGGCCAATTTCACTGATGAACATAGATGCAAAAATCCTCAACAAA

ATACTAGCAAACCCATCCAACAATACATTAAAAGGATTGTACATCATGATCAAGTGGGAT

TTATCCCAGGGATGCAAGGGTTCTTCAATATCTGCAAATCAATCAGTGTGATACACCACA

TTAACAAACTGAAGAATAAAAACCATATGATCCTCTCAATAGATGCAGAAAAAGCCTTTG

ACAAAATCCAACACCCATTTCTGATAAAAACCCTTCAGAAAGTGGGCATAGAGGGAACCT

ACCTCAACATAATAAAGGCCATATATGACAAACCCACAGCAACATCATTCTCAATGGTGA

AAAGCTGAAAGAATTCCCACTGAGATCAGGAACAAGACAAGGATGTCCCTCTCGCCACTA

CTTTCAACATAGTTTTGGAAGTCCTAGCCACAGCAATCAGAGAAGTAAAAGAAATAAAAG

GAATCCAAATTGGAAAGGAAGAAGTAAAACTATCACTATTTGCAGATGACATGATACTAT

ACCTAGAGAATCCTAAAGACTCTACCAGAAAACTGTTAGAGCTCATCCATGAATTTGGCA

AAGTCCAGGATACAAAATTAATACACAGAAATTGACGCATTTCTATATACTAACAATGAA

AGATCAGAAAGAGAAATTAGGGAAGCAATCCCATTTACCATCACATCCAAAAGAATAAAA

TACCTAGGAGTAAACCTACCTAAAGAGACAAAAGACCTGTACTCTGAAAACTATAAGACA

CTGATGAAAGAAATCAAAGATGACACAAATAGATGGAAAGACATACCATGCTCTTGGATT

GGAAGAGTCAATATTATCAAAATGACTATACTACCCAAGGCAATCTACAGATTCAATGCA

ATCCCTATCAAATTACCAAGGACATTTTTCACAGAACTCGAACAAAATATTTTAAAGTTT

GTTTGGAAGCACAAAAGACCCAGAATAGCCAAAGACATCCTGAAAAAGAAAAATGGAGCT

GGAGGAATCAGGCTCCCTGACTTCAGACTATACTACAAAGCAACAGTCATCAAAACGTAT

GGTACTGGCACAAAGACAGAAATATAGATCAGTGGAACAGGATAGAAAGCCCAGAATTAA

ACCCACGCACCTACAGCCAACTAATCTATGACAAAGGAGGCAAGAATATACAATGGAGAA

AAGACAGCCTGTTCAATAAGTGGTGCTGGGAAAACTGGACAGCCACATGTAAAAGAATGA

AATTAGAACACTCCCTAACACCATACACAAAAATAAACTCAAATGGATTAAAGACCTAGA

TATAAGACCAGACACTATAAAACTCTTAGAGGAAAACATAGGCCAAACACTCTCTGACAT

AAACGACAGCAACATCTTCTCAGATCCACCTCTTAGAGTAATGACATAAAAACAAAAATA

AACAAATGGGACTTAATTAAACTTAAAAGTTTCTGCACAGCAAAGGAAACCCTAAACAAA

ATGAAAAGACAACCCACAGAATGGGAGAAAATCTTTGCAAATGAATCAACTGACAAGGGA

TTAATCTCCAAAATTTATAAACACCTCCTACAGCTCAATACCAAAAAAAACAACCCCATC

AAAAAATGGGCAGAAGATCTAAACAGACAATTCTCCAAAGAAGACATACAGATGGCCAAA

AAACACATGAAAAGATGTTCAACATCACTCATTATTAGAGAAATGCAAATCAAAACCACT

ATGAGGTACCACCTTACACCAGCCAGAATGGCCATCATCAAAAAGTCTACAAACAATAAT

GCTGGAGAGGGTGTGGAGAAAAAGGAACCCTATTACACTGTTGGATTGTAAATTGGTGCA

ACCACTGTGGAAAACAGTATGGAGATTCCTCAGAAAACTAAAAATAGAACTACCATTTGA

TCCAGCAATCCCACTCCTGGGCATCTATCCAGAGAAAACCATGACTCGAAAAGACACATG

TACTCCAATGTTCATTGCAGCACTATTTCAATAGCCAAGACATGGAAACAACCTAAATGT

CCATCGACAGAGGAGTGGATCAAGAAGATGTGGTACATATACACAATGGAATATTACTCA

GCCATTAAAAGGAAGAAATAGGCATTTTTAGCAACATGGATGGAGAAATTATCATGCTAA

GTGAAGTCAGTCAACAATGAGACACCAACATCAAATGCTTCACTGACATGTGGAATCTGA

AAAAAGACAAATGAACTTCTTTGCAGAACAGATACTGACTCACAGACTTTGAAAAACTTA

TGGTTTCCAAAGGAGACAGTTTGGGGGGTGGGGGGATGCCTGGGGTGTGGGATGGAAATC

CTATAAAATTGGATTGTGATGATCATTGTACAACTATAAATGTAATAAATTCATAGTAAT

AAAAAAAAAAAAA

>L1A7#LINE/L1A RepbaseID:L1_3_SSc

GGGGAGGTCTAAGATGGCGGAGTAGCAGGACGTAAGGCTCATCTACTCCCACAAATACAT

TGAAAATACAACTATATGTGGAACTATTCGCACAGAAAATTTGCGAAAAACTGACAAAAG

ACCTCAAGATTATGATAGAACAAGAAAAACATTACAAAACCAGATAGGACATAAGAAAGA

AGAAGAACAAGAGAAAAAGAAGCGAAAGGAAATGAGATGGGACCTGCTCTCCCAGGAGGG

AGCTGGAAAGAGGAATAGCTCCTGCACCCCGGGAAGTTCCCCCACCAGCGACGAGATCAG

CCAAAAACGGAGGAGGAACTCTAGACAAATGGTCTGAAGCAGTTAAGAAGGAGACAGTCC

TCCACAAACGGTCAGTGCTACGGGCAACGGGCAACCGTAGGCGGGTGCCAGCAGGTGTTG

GGAACTAAAACTTTGGCCTTAGAGATCAGACCCAAAGAGGGAGCTGGGGCCGGCTACGTG

GGGGGAAAAAACTGGGTTGGCCGTGCGGAAACAGCCTGGAGGGACTAGAGTATAGGGCGA

CCACAATGGAGAGCATCTAAGCGGAGGCAACCCAGTCGGGCTTAGAGACTAGACGCCATT

GTTTGGGGGTGCTGGAAGGAAGGGGCGGGATCCACCACTACAGCCTTGTTCCCTACGCCC

GCTTTCTCAAGCTGCAGGACACTGCCTGCCTCAGCTCGGGAACCACTTGGCCGTAGCGGC

TGCCTAGCAACGGCTGGGAAGCAGCCCCGCCCTTAGGGCTACAGACTTCTTGGAGCAGGT

GGAGTAACCGCCTGGGGGAATAACACAAGTTCCCGGTTCCTGCCGGCGGGGGCCCTGCGC

TAGTGGAGCCCAACCTGCGCCAGAAGAGCTGCCGGCGGTGGCGGCGCTCATCCCCCGAGA

GATCAGGGAAAACACAGGTGCTTTGGCTCCACCCAGAGAATCTGCAGGGGCTCTGACCAG

CAGGGCAGCGCCAGAAAAGCTGCTGGCGGCAGCGCCCACTTCCTGCAAAAGCAACGACCT

GCTTGAGCAGGGAAAACACAGGAGTCTCAGCTCCGCCCAGAGAGGCAGCACCCATTCCCG

CGAGAGGGCCGCCGGTGCCCGCTCCCTGCCGATCAGGGAAAACACAGGCACCTTGGCTCC

GCCCAGAGAATCTACAGAGGCTCGCGCCAGCAAAGCAGCGCCAGAAAAACTGCTGGTGGC

AGCGCCCACTTCCTGCAAAAGCAGTGACCTGCTTGAGCAGGGAAAACACAGGCAAACACA

GGCGTCTCAGCTCCGCCCAGAGAATCTGCAGAGGCTCTGCCAGCGCGGCAGCGCCCATTC

CCGTGAGAGGGCTGCCGGTGCCCGCTCCCTGTGATCAGGGAAAACACAGGCGCCTTGGCT

CCGCCCAGAGAATCTGCAGAAGCTCGCGCCAGCAAAGCAGCGCCAGAAAAGCTGCTGGCG

GCAGCGCCCATTCCCACGAGAGGGCTGCCGGTGCCCGCTCTCTGACGGAGCTGTGGGCTG

CCCAACCGGCGAAAAGCACAAGATACAGGACCCCTGCCAGTGGCCCTGTGAAGGCTCACG

TCAGTGGCACTCTTTCCACACCAAGGGAGCCGGGGGAGGCCCATTAACCCACATTCCAAT

CTGCAGGGCTACAGAGAGCACTCCTACCAGCAGCAAGGCAGGGGGAGGGACCGCCAGAAA

CGCACATCCTGCAGCTACAGAGAGAGCCTGTGAGCAACAATTAGGGAGAAGCCCCCACCC

CTGAGGCTTTAGAGAGAGATCCCTAGAGTGGCCAAGCAAGGAGAGTGCCGGCAGAACAGC

GGCACCTGCTCCTATATCTACAGAGAGCAGTCCCTAGAGCCTACCCAGCAAAGAGGTACA

CGTGAACCACTACCACCCCTGAGAACTCCAGGACTGGGCACCTGCTTCAACATCTACATA

CCAACTGTCAAGGGGACGATAAACAGAAAGGTAGACACTATGAGACGACAAAGAAGCAGC

TTTCAGACAAAGGAACAAGACAAAAACACACAAAAACCACTAAATGATGAGGAGATAGGC

AATTTACCTGAAAAAGAATTCAGAGTGATGATAGTAAAGATGATCCAAGATCTTGGAAAA

AGAATGGAGACACAGATCGAGAACTTAAAAGAAATGTTTAACAAAGAGCTAGAGGATTTG

AAGAGCAAAATGAATAGTGCAATAGCTGAAATGAAAAATAATCTAGAAGGAACCAATAGC

AGGTTAACGGAGGCAGAAGAACGAATAAGTGAGGTGGAAGACAGAGTGGTGGAAATCACT

GCTACAGAAAAGAATAAAGAAAAAAGAATGAAAAAAACTGAGGAGAGTCTAAGAGACATC

TGGGACAACATTAAACGTACCAACATTCGCATCATAGGGGTTCCAGAAGGAGAAGAGAGA

GAAAAAGGGCCAGAGAAAATATTTGAAGAGATTATAACCGAAAATTTCCCAAATATGGGA

AAGGAAACGCTCACTCAAGTGGAAGAAGCACAGAGAATTCCATACAAAATAAACCCCAGA

AGAAATACAGCAAGACACATACTAATCAAACTGACAAAAATTAAGTTCAAAGAAAAAATA

TTAAGAGCCACAAGGGAGAAGCAACAAATCACATATAAGGGAATTCCCATAAGGATAACA

GCCGATCTCTCAGCAGAAACTCTACAAGCCAGAAGGGAGTGGCAAAATATATTTGAGGTG

ATGAAGAGGAGGAACCTAGAACCAAGAATACTCTACCCAGCAAAGCTCTCATTCAGATTT

GATGGAGAGATCAAAAGCTTCACAGACAAGCAAAAGCTAAGAGAATTCAGCACCTCCGAA

CCAGCTCTACATCAACTACTAAAGGAACTTCTCTAAGCAGAAAAGGAAAAGCCACAATTA

GAAACAAGAATATTAAAAATGAAGAAGCTCACTGGTAAAGGCAAAGATAATTTAAAAGTA

GGAAATCACCCATTGACAAATATGATATCTAAACTAGCAAGCATGAGAAGAGGAGAGGAC

AAATGCAGAACATTGAAAATGCATTTGAAATTAAGGAGACCAGCAAGACGAAACAATTCT

GCACACATATAGATGGATATATCAAAATATAAGGGGAACCACTAGTCAAATAACTATAAT

GGTCACACACATAAAAAAGAAAAACCAAGCCAAACATAACACTAAACATGGTCAGCAAAT

CGCAAGAAAAGACAACAAAAAGAGGAAGGGAAGAAAAAAGACCCAAAATAGCAATCCAAA

AATTTTAAGTAAATGACAATAAATACATACTTATCCATAATTACATTGAATGTAAACGGA

TTAAATGCTCCAACTAAAAGAAAAAGACTAGCTGAATGGATTCAAAAGCAAGACCCATAT

ATATGCTGTCTACAGGAGACCCACTTCAGATCTAAGGACACATACAGACTTAAAGTGAGG

GGATGGAATAAAATATTACATGCAAATGGAAATTATAGAAAAGCAGGAGTAGCAATACTC

ATATCAGACAAAATAGATTTTAAAATAAAGAAGGTCACAAGAGACAAAGAAGGACATTAC

ATAATGATCAAAGGATCAATACAAGAAGAAGAAATAACAATTTTAAATATATATGCACCC

AACATAGGAGCAGCACAATATATAAGGCAACTACTAACAGCCATAAAAGGGGAAATTGAC

AGTAACACAATAATAGTGGGGGACTTTAACACCCCACTTACAGCAATGGACAGATCATCC

AGACAGAAAATCAGCAAGGAAACACAGGCCTTAAATGATACACTAGACCAAATAGACTTA

ACTGATATCTATAGAACTTTTCACCCCAAAGCAGCAGAATATACTTTCTTCTCAAGTGCA

CACGGAACATTCTCCAGGATAGATCACATCTTGGGCCACAGATCAAGCCTTGATAAATTT

TTAAAAATTGAAATTATTTCAAGCATTTTCTCCGATCACAATGCTATGAGACTAGAAATA

AACTACAAGAAAAAAAAAAAAAAAAAGCAGCAAAAACCAAACTCTTGGAAGCTAAACAAT

ATGCTACTAAACAACCAATGGATCATTGAAGAAATCAAAGAGGAAATTAAAAGATACATA

GAGACAAATGACAATGAAGATACAACAATCCAAAACCTATGGGACACAGCAAAAGCAGTT

CTGAGAGGGAAGTTTATAGCAATACAATCTTATCTCAGGAAAGAAGAAAAAATGCAAATA

AACAACCTAACCTTACACCTTAAACATCTAGAGAAGGAAGAACAGACAAAGCCCAAAATT

AGTAGAAGGAAAGAAATCATAAAGATTAGAGCAGAAATTAATAACATAGAGACAAAGAAA

ACAATTGAGAAGATCAATGAAACCAAAAGTTGGTTCTTTGAAAAGATCAACAAAATTGAT

AAACCGTTAGCCAGACTCATCAAGAAAAAAAGGGAGAGGGTTCAAATCAATAAAATTAGA

AATGAAAAAGGAGAAGTTACAACTGACACTGCAGAAATACAAAGGATCATGAGAGACTAC

TATGAGCAACTGTATGCCAATAAAATGGACAACCTAGAAGAAATGGACAGATTCTTACAA

AGGTACAACCTACCAAGACTGAACCAGGAAGAAATAGAAAATATGAATAGACCAATCACA

AGTACTGAAATTGAAAATGTGATTTAAAAACTTCCAAAAAACAAAAGTCCAGGACCTGAT

GGCTTCACAGCTGAGTTCTACCAAACATTCAGAGAAGAGTTAACACCTCTTCTTCTGAAA

CTTTTCCAGAAAATTGCAGAGGAAGGAACACTCCCAAGCTCATTCTATGAAGCCACCATT

ACCCTGATACCAAAACCAGACAAAGATACCACAAAAAAAGAAAATTACAGGCCAATATCA

CTGATGAACATAGATGCAAAAATCCTCAACAAAATATTAGCAAACCGCATACAAATATAC

ATTAAAAAGATCATACACCATGATCAAGTAGGATTTATCCCAGGGATGCAAGGATTCTTC

AATATCCGCAAATCTATCAATGTGATACACCACATCAACAAACTGAAAAATAAAAACCAT

ATGATCATCTCAATAGATGCAGAAAAAGCTTTTGACAAAATTCAACACCCATTCCTGATA

AAAACTCTCCAGAGAGTGGGCATAGAGGGGAACTACCTCAATATAATAAAAGCCATTTAT

GACAAACCCACAGCTAACATCATTCTCAATGGTGAAAAACTGAAAGCATTTCCACTAAGA

TCAGGAACAAGACAAGGATGTCCTCTTTCACCAATGTTATTCAACATAGTCTTGGAAGTC

CTAGCCATGGCAATCAGAGAAGAAAAAGAAATAAAAGGAATCCAAATTGGAAAAGAAGAA

GTAAAACTATCACTGTTTGCAGATGACATGATTCTATACCTAGAAAATCCTAAAGACACT

ACCAGAAAACTATTAGAGCTCATCAATGAATTTGGTAAAGTCGCAGGATACAAAATTAAT

ACACAGAAATCGATTGCATTTCTATATACTAACAATGAAAGATCAGAAAGAGAAATTAGG

GAAACAATCCCATTTACCATTGCATCAAAAAGAATAAAATACCTAGGAATAAACCTACCT

AAAGAGACAAAAGACCTGTACTCTGAAAACTATAAGACACTGATGAAAGAAATCAAAGAA

GACACAAATAGATGGAAAAATATACCATGCTCTTGGATTGGAAGAATCAATATAGTCAAA

ATGACAATACTACCTAAGGCAATCTACAGATTCAATGCAATCCCTATCAAATTACCAATG

ACATTCTTTACAGAACTAGAACAAAATATTTTAAAATTTGTATGGAAACACAAAAGACCT

CGAATAGCCAAAGCAATATTGAAAAGAAAAAACGGAACTGGAGGAATCAGGCTCCCTGAC

TTCAGACTCTACTACAAAGCTACAGTCATCAAGACGGTATGGTACTGGCACAAAAACAGA

AATATAGATCAATGGAACAGGATAGAAAGCCCAGAAATAAACCCAAACACCTATGGTCAA

TTAATCTATGACAAAGGAGGCAAGAACATACAGTGGAGGAAAGATAGTCTCTTCAATAAA

TGGTGCTGGGAAAACTGGACAACTGCATGTAAGAGAATGAAATTAGAACATTGTCTAACA

CCATACACAAAAATAAACTCAAAATGGATTAAAGACCTAAATGTAAGGCCAGACACTATA

AAACTCCTAGAGGAAAACATAGGCAGAACGCTCTTTGACATAAATCACAGCAACATCTTG

TTTGATCCACCTCCTAGAATAAAGACAATAAAGACACAAATAAACCAATGGGACCTAATT

AAACTCAAAAGCTTCTGCACAGCAAAGGAAACCATTAAAAAAATGAAAAGACAACCCACA

GAATGGGAGAAAATTTTTGCAAATGACTCAACCGACAAAGGTTTAATCTCCAAAATATAC

AAACAACTCATACAACTCAACAACAAAAAAACAAACAACCCAATTGAAAAATGGGCAGAA

GACCTAAATAGACATTTCACCAAAGAAGACATACAGATGGCCAGCAGGCACATGAAAAAA

TGCTCAACATCACTAATTATTAGAGAAATGCAAATCAAAACTACAATGAGGTACCACCTC

ACACCAGTTAGAGTGGCCATCATTAACAAGTCAACAAATAACAAATGCTGGAGAGGATGT

GGAGAAAAGGGAACCCTCCTACACTGTTGGTGGGAATGTAAATTGGTACAACCACTATGG

AAAACAGTATGGAAATACCTCAGAAAACTAAATATAGAACTACCATATGACCCAGCAATC

CCACTCCTGGGCATATATCCGGACAAAACTTTCATTGAAAAAGATACATGCACCCGTATG

TTCATCGCAGCACTATTCACAATAGCCAAGACATGGAAACAACCTAAATGCCCATCGACG

GATGAATGGATTAGGAAGATGTGGTACATATATACAATGGAATACTACTCAGCCATAAAA

AAGGACAAAATAATGCCATTTGCAGCAACATGGATGGAACTAGAGACTCTCATACTGAGT

GAAGTAAGTCAGAAAGAGAAAGACAGACACCATATGATATCACTTATATGTGGAGTCTAA

AATATGGCACAAATGATCTATCTACAAAACAGAAAAGATCATGGACATGTAGGACAGACT

CGTGTTTGCCAGGGGGGAGGGGGAGGGAGTGGGATGGATTGGGAGTCTGGGGTTAGTAGA

TGAAAACTCTTGCATTTGGAGTGGACGGGCAATGAGATCCTGCTGTATAGCACAGGGAAC

TATATATCTAATCACTTGTGATGGAACATGATGGAGGATAATGTGAGAAAAAGAATGTAT

ATATATGTATGACTGGGTCACTTTGCTGTACAGCAGAAATTGACAGAACATTGTAAATCA

ATCATAAAAAATTTAAAAAAAAAA

>L1B1#LINE/L1B RepbaseID:L1B-SS

GGGAGGCGGAGCAAGATGGCAGAGGAGTAAGAGGTCGCGCACACCTTCTCCCACAAACAC

ATCAAAAAAACACATCTACATGTAAAACTACTCCACAGAACATCAACTGAATGCTGGCAG

AAGAACTTAAACCTCCAAAAAGGGCAAGAAACTCTTGACATAACTGGGTAGAACAAAAGA

AAAAAAAGAGAGAGAAAAGGAATCAGGATGGGACTAGCATTCCCGAGAGGGAGCTGTGAA

GGAGAAAAGGAACCCACCTCCTGGGAAGCCACCTAACTGACGAAAGATCGCTGAGTCGGG

GGACCTCAAAGTCCGAGAAAAGCGCAGCAGCTGGACTGAGAACAGCAAAGCAGAGTGAGA

GCCGCACAGATCATCTGAACCACGGCCGGACACCACAGCCTGAGATGCTCGGGCGGGGGC

TGGGCCTGAGACTCAGGCTCCGGAGGTCAGTCCCGGGGAGAGAACTGGGGATGGCATGAG

GGGCTAAGGAGCAGTGCCCAGGGCTAAGGAGGGGAACACCATGGCAGAGGGAACCCGGAG

AAGGTCGGACCCGCAGGAGAGGCAAGGCGCCATTGTTGGGGAGGGGAGAGGAGGAGGGGC

GGGCCGCCATAGGAAACTCCCTGCCTGGAGCATGCGCATGCCCACGGCTTAGAGGGTGGG

GCGGCTCTGCGGAGGCTAGGGGCAGAAGCCTCTTGCTCATTTAGGGGAGATTAGGCACTT

CTTGTGCAGGCTACCGGTGGCCAGGCACCTATTGTGTGGGCTAAGGGCATAAGGGGGCTA

AGTGCGACGTGGTGCCTCTTGCACGATCTACAGGTGGCAGGGACAGACTGGGCGGTCATC

TCAGAGGCCAGAGGGAGGCGTGGCTTGCCACCACTGGGGGCCTGTGAGTGGGCTCCACCG

CGACCCCAGTCACCTCAGGGGTTGGCAAAAAAGAAAAAAAGAGGGCATTGCAACCAAGCA

CCATACGCTGTTGCTCTCACTCCCCTGGGAACACACCCACCCTGCAGCTGCCACTGCCAA

ATGCTCTGGGTGACCCCAGACACTTGGTCACTGTCCCTTCCCAAGACCCTACAACTAGGA

GCAACTGGTGCAGCACCTCCTGCGTGGGCTAAGTGGGACAGGGTGCTTCTTGCTGGTCTG

CAGGGGCAGGGGCAAACCCCACAGTTATCCCTGATTCCAGAGGTGGGCGTGGCCCACTGC

CATTGGAGATATCTGAACAAAAATCACTTGCAGCCCCATTCACCTAAAGGGCACCACAGA

GGAGGGCATTGTGACCAAACACCACCTGTTGGTGCTCTCGCACCCCTGGGAACACACCCG

CCCTGCTGCTGCCACTGCCAAACTTCTGGGCAGTACCCACAGCCTGATCTCTGTCACTTC

CCAGGATCCTGCAACTAGGAGCACCCTTTACAACACCTCATATGTGGGCTAAGTGAGACG

GGTTGCTTCATGCATGGTCTACAGGTGGAGGGGCAAACCACCCAGTTATCACTGACTCTA

GAGATGGTCATGGCCTGCTCCCAATAGGGGTCCCTGAACAGGCACCACCTGTGGTTCCAA

TCACCTCAGAGGAGGGCATTGCAATCAAACACAAGCTGTTGTTGCTCTCACTCCCCTGGG

AACTCACGCACCCTGCTGTTGCTACTGCCAAATGCTCTGGTCACCTTGAAGATCTGCCTA

GAGCTTATTACCACTTCCCAGGGCCCTGCAACTAGGAGTAGCCTGTGCCACCTTCCTGCA

GGTCCTTGCTGCTATTGAGAGCCCAATGACCAGGCACTGACTGCTGGCCCTACCCATTGC

CTCCTTCTCCCTGAAAACACACTGAGCATCCTGGGAACAACAGCCTGCTCACACCAAAGA

AAGAGACAGCAAATATTCAAACTCCCACACCAAAAATAAATAGTAACCCCCCAAAAATAC

AAAGGGGTATTCTTGCATAGAAGTAGCCTTACAAGACTACAGTTTGGCTTCCCCAAACTC

ACAGAAAAAGAAAAACACAAGCAAGATGAAGAAGCACAGAAACCATTCCCAGTTAAAGGA

ACAGGAGAATTCACCTAAAGCAGTCAACAATGAAACAGACCTCTGCAGTCTGACAGACAT

TGAGTTCAAAAGGGAGGTATTGAAAATACTGAAGGAATTAAGAGAGGATATGAACAGTAA

TGCAGATTCCTTTAGAAAGGAACTAGAAAATATAAGGAGGAGCCAAGAAAAACTAGAAAA

TTCATTTGCAGAGATACAAACTGAGCTTAAGGCAATAAAGAGCAGAATGAATAATGCAGA

GGAATGAATTAGTGATGTGGAAGATAAAATAATGGAAATCACCCAATCAGGACAGCAGAC

AGAAAACCAAATGAAAAAACATGAAAGCAATATAAGAGACCTATGGGATAATATAAAGCA

GGCCAATCTATGCATAATAGGAATTCCAGAAGGAAAAGAAAAAGAAAAGGGGATTGAAAA

TATATTTGAAGAAATTATGGCTGAAAACTTTCCAAATCTAAAGGATACTGATATCAAGAT

ACAGGAAGCACAGAGGGCCCCAAACAAGCTGAACCCAAACAGGCCCACACCAAGACATAT

TATAATAAAAATGGCAAAAGTTAAAGATAAAGAGAGGATTCTAAAGGCAGCAAGAGAAAA

GCAAAGCATTAATTATAAGGGAACCCCCATAAGGCTATCAGCTGATTTCTCTACAGAAAC

ACTACAGGCCAGAAGGGAGTGGCAAGATATATTTAAAGTGCTGAAAGGAAAAAAATTGCA

ACCTAGAATACTCTATCCAGCAAGAATATCATTTAAAATAGAAGGGGAAATAAAGAATTT

CTCCAACAAACAAAAGCTAAAAGAGTACAGCAATACTAAACCCATTCTAAAAGAAATACT

GAAAGGGCTTCTCTAAATTAAAAAAAAAAAAAAAAAGAAGAATTAGGATGGAGGAAACCA

CAATTGGAAAGCAATCACTTAAATAAGCCAGCATACAGATCTAAACATGAAGATGTTAAA

AAAAAAGACATCAAAATCATACAATGTGGGGAAGGAAAGTAAGAAAATATAGATTCTTTT

TTTTATTTTAATGATGTGTTTGAGCCTATATGACTATCAGGCAAAAGCAAGCAGATATAG

GAAGGGCTTAACTGCTTAAAAAACAGGGCAACCACAAATCAAAACCAAACATTACATTCA

CAAAAACTGAAAAGAAAAGTACTCAAGCATAAAATAAATGGAAACCATCCAACCCAAAAA

AGAAAAAAAAAAGAGAAACATAGAATCAACTGGAAAACAAGGTTTAAAATGGCAATAAAT

ACATATCTATCAATAATCACCTTAAATGTCAATGGACTGAATGCTCCAATCAAAAGACAC

AGAGTGGCAGATTGGATAAAAAAGCAAAAACCTTCAATCTGCTGCCTACAAGAAACTCAC

CTTAGAGCAAAGGACACATATAGATTGAAAGTGAGGGGATGGGAAAAGATATTTCATGCC

AATGGACAAGACAGGAAAGCAGGAGTTGCAATACTCATATCAGACAAAATAGACTTTAAA

ATGAAGGCCATAAAGAAAGACAAAGAAGGACACTATTTAATGGTTAAAGGATCCATTCAA

GAAGAGGATATTACAATCATCAATATATATGCCCCTAATATAGGAGCACCCAGATACCTA

CAACAAATACTAACAGACATAAAAGGAGAAATTGATGGGAATACAATCATAGTAGGAGAC

TTTAACACCCCACTCACATCAATGGACAGATCCTCTAGACAGAAAATCAATAAGGCAACA

GAGATCCTAAAGGACACAATAGAAAAGTTAGACTTAATTGACATTTTCAGGACATTACAT

CCAAAAAAACAGAATATACATTCTTCTCAAGTGCACATGGAACATTCTCAAGAATTGATC

ACATACTGGGGCACAAAGCTAACCTCAACAAATTTAAGAGTATAGAAATTATTTCAAGTA

TCTTCTCTGACCACAATGGCATGAAACTAGAAATCAACCACAGGAAAAGAAATGAGAAAA

AACTACTACATGGAGACTAAACAACATGCTACTAAAAAACCAATGGGTCAATGAGGAAAT

CAAGAAGGAAATTAAAAAATACCTCGAGACAAATGATAATGAAGACACACCACTCAAAAT

CTATGGGATGCCCAAAAGCAGTGCTCAGAGGGAAATTCATAGCAATACAGGCCTTCCTCA

AAAAAGAAGAAAAATCTCAAATCACAACTTAACCCACCACCTAAATGAATTAGAAAAAGA

AGAACAAACAAAACCTAAAGTCAGCAGAAGGAAGGAAATCATAAAGATCAAAGAGGAAAT

CAATAAAATAGAGATTCAAAAAACAATGAAAAAATCAATAAAACCAAGAGCTGGTTCTTT

GAAAAGGTAAACAAAATTGACAAACCTCTGGCTAGACTCACCAAGAAGAGGAGAGAAAAA

ACCCAAATAAACAAAATAGAAATGAAAAAGGAGAAGTCACAACGATACTACAGAAATACA

AAAAACCATGAGAGAATACTATGAACAATTGTATGCCAACAAATTTGACAACCTAGAAGA

AATGGACAACTTTCTAGAGACTTACAGCCTGCCAAAACTGAATCAAGAAGAAATAGATCA

ACTGAACAGACCGATCACTAGAAATGAAATTGAATATGTCATAAAAACACTCCCTACAAA

TAAAAGTCCAGGACCAGATGGCTTCACAGGTGAATTCTACCAAACATACAAAGAGGAACT

TATACCCATCCTCCTTAAACTTTTTCAAAAGGTTGAAGAAGAAGGAACACTCCCAAAGAC

ATTCTATGATGCCACCATCACCCTAATTCCAAAACCAGACAAAGATACCACCAAAAAAGA

AAACTATAGGCCAATATCTTTGATGAATATAGATGCAAAAATTCTCAACAAAATTTTAGC

CAACCGAATCCAACAACATATAAAAAGATCATACACCACGACCAGGTGGGATTCATCCCA

GGTGCACAAGGATGGTTCAACATATGCAAATCAATCAACGTCATACACCACATTAACAAA

AGAAAAGTCAAAAACCACATGATCATCTCAATAGATGCAGAAAAAGCATTTGACAAAGTC

CAACATCCATTCATGATCAAAACTCTTACCAAAGTGGGTATAGAGGGAACATACCTTAAC

ATAATCAAAGCCATTTATGACAAACCCACAGCAAATATAATACTCAATGGAGAAAAGCTG

AAAGCCTTCCCACTAAAATCTGGAACAAGACAAGGATGCCCACTCTCACCACTGTTATTC

AACATAGTACTGGAAGTCCTAGCCACAGCAATCAGACAAACAAAAGAAATAAAAGGCATC

CAAATAGGAAGAGAAGAGGTAAAACTGTCACTGTATGCAGATGACATGATACTATATATA

GAAAACCCTAAGGACTCAACCCAAAAACTACTTGAACTGATCAACAAATTCAGCAAAGTA

GCAGGATATAAGATTAACATTCAGAAATCAGTCGCATTTCTGTATACTAACAATGAAATA

TTAGAAAAGGAATACAAAAATACAATACCTTTTAAAATTGCACCCCAAAAAATCAAATAC

CTGGGAATACACCTGACCAAGGAGGTAAAGGACTTATATGCTGAGAACTATAAAACATTA

ATCAAGGAAATTAAAGAAGATGTAAAGAAATGGAAAGATATTCCATGCTCCTGGGTTGGA

AAAATTAATATTGTAAAAATGGCCATACTACCCAAAGCAATCTACAGATTCAATGCAATC

CCTATCAAATTACCCATGACATTTTTCACAGAACTAGAACAAACAATCCAAAAATTTATA

TGGAACCACAAAAGACCCAGAATTGCCAAAGCAATTCTGAGGAACAAAAACCAAGCAGGA

GGCATAACTCTCCCAGACTTCAGGCAATATTACAAAGCCACAGTCATCAAGACAGTGTGG

TACTGGTACCAAAACAGACAGACAGACCAATGGAACAGAATAGAGAACCCAGAAATAAAC

CCAGACACCTATGGTCAATTAATCTTTGACAAAGGAGGCAAGAACATAAAATGGGAAAAA

GACAGTCTTTTCAGCAAGTATTGCTGGGAAACCTGGACAGCTGCATGCAAATCAATGAAA

CTAGAACACACCCTCACACCATGCACAAAAATAAACTCAAAATGGCTGAAAGACTTAAAT

ATAAGACAAGACACCATCAAACTCCTGGAAGAGAACATAGGCAAAACATTCTCTGACATC

AACCTTATGAATATTTTCTCAGGTCAGTCTCCCAAAGCAACAGAAATAAAAGCAAAAATA

AACCAATGGGACCTAATCAAACTGACAAGCTTTTGCACAGCAAAGGAAACCAAAAAGAAA

ACAAAAAGACAACTTACAGAATGGGAGAAAATAGTTTCAAATGATGCAACTGACAAGGGC

TTAATCTCTAGAATATACAAGCAACTTATACAACTCAACAGCAAAAAAGCCAACAACCCA

ATGGAAAAATGGGCAAAAGACCTGAATAGACATTTTTCCAAGGAAGATATACAGATGGCC

AACAAGCACATGAAAAAATGCTCAACATCCCTGATTATTAGAGAAATGCAAATCAAAACT

ACCATGAGATACCACCTCACACCAGTCAGAATGGCCATCATTAATAAGTCCACAAATAAC

AAATGCTGGAGGGGGTGTGGAGAAAAGGGAACCCTCCTGCACTGTTGGTGGGAATGTAAG

CTGGTACAACCACTATGGAGAACAGTATGGAGGTACCTTAGAAATCTATACATAGAACTA

CCATATGACCCAGCAATCCCACTCTTGGGCATATATCCGGACAAAACTTTCCTTAAAAAA

GACACATGCACCCGCATGTTCATTGCAGCACTATTCACAATAGCCAAGACATGGAAACAA

CCCAAATGTCCATTGACAGATGATTGGATTAGGAATGTGGTATATATACACAATGGAATA

CTACTCAGCCATAAAAAAGAACAAAATAATGCCATTTGCAGCAACATGGATGGAACTAGA

GACTCTCATACTGAGTGAAGTAAGTCAGAAAGAGAAAGACAAATACCATATGATATCACT

TATATCTGGAATCTAATATAGGCACAAATGAACCTTTCCACAGAAAAGAAAATCATGGAC

TTGGAGAATAGACTTGTGGTTGCCAAGGGGGAGGGGGAGGGAGTGGGGTGGATTGGGAGC

TTGGGGTTAATAGATGCAAACTATTGCCTTTGGAATGGATTAGCAATGAGATCCTGCTGT

GTAGCACTGGGAACTATGTCTAGTCACTTATGATGGAGCATGATAATGTGAGAAAATAGA

ATGTGTACATGTATGTGTAACTGGGTCACCATGCTGTACAGTAGAAAAAAAAATTGTATT

GGGGAAATAACTATTAAAAAAAAAAAAAAAAAAAAAAAA

>L1B2#LINE/L1B

GGGCAGAGCAAGATGGCGAGGAGTAAGAGGTCAGCTCACCTTCTCCCACAAACACATCAA

AAAAACACATCTACATGTAAAATGACTCACACAGAACATCAACTGAATGCTGGCAGAAGA

ACTTAAACCTCCAAAAAGGGCAAGAAACTCTTGACATAACTGGGTAGAACAAAAGAAAAA

AAAAGAGAGAGAAAAGGAATCAGGAGGGACTAGCATTCCTGAGAGGGAGCTGTGAAGGAG

AAAAGGAACCCACATCCTGGGAAGCCACCTAACTGAGGAAAGATCAGCCAAGTCGGAGGG

ACCTCAAAGTCCCGAGAAAAGCCAGCAGCTGGACTGAGAACAGCAAAGCAGAGTGAGAGC

CGCACAGATCATCTGAACCACCGGCCTGGACACCACAGCCTGAGATCTCGGCGGGGCTGG

GCACTGAGACTTAGGCTCCGAGGTCAGTCCTGGGAGAGGACTGGGGCTGGCTGTGTGGGG

ACAGCCTGAGGGGCTAAGGAGCAGTGTGCCACGGTGGGGGAGTGGTATGCCATGGGCTGA

GGAGGGGAACGCCACGCAGAGGGAACCCAGGAGAAGGTCCGGACCCCAGGAGAGGCAAGG

TGCCATTGTTGGGGAGGGGAGAGGAGGAGGGGCGGACCCCATAGGAAACTCCCTGCACCG

AGCTGCATGCCTGTGGGCTCTCAGAGGGTGGGGGCTCTGGTGCAGGCTACAGGGGCGAGA

AGCCTCTTGCTCATTTAGGGGAGATGGGCCTTCTTGTGCAGGCTACCGTGGCCAGGCACC

TCTTGTGTGGGCTAAGGGCATCAGGGGGCTAAGTGCGATGTGGTGCCTCTTGCATGATCT

ACAGGTGGCAGGGACAGACTGCGGCAGTCTCTCAGAGGCCAGAGGGAGGCTGGCTTGCCA

CCACTGGGGGCCTGTGAGTGGGCTCCACCTGCACCCCAGTCACCTCAGGGGTTGGCAAAA

AAAAAAAAAGAGGGCACTGCAACCAAGCACCATAGCTGTTGCTCTCACTCCCCTGGGAAC

ACACCCGCCCTGCAGCTGCCACTGCCAAATGCTCTGGGCAGCCCCAGACACTTGGTCACT

GTCCCTTCCCAAGACCCTACAACTAGGAGCAGCTGGTGCAGCACCTCCTGCATGGGCTAA

GTGGGACAGGGTGCTTCTTGCATGGTCTGCAGGTGGCAGGGGCAAACCACCACAGTTATC

CCTGATTCCAGAGGTGGGCTGGCCCACCACCACTGGGGATACCTGAACAAAAATCACTTG

CAGCCCCAACCACCTCAGAGGGCACCACAGAGAAGGACATTGGAGAACACCACCTGTTGT

TGCTCTTGCTCCCCTGGGAGCACACCCCCTGCTGCTGCCACTCCAAACTCTGGGAGTGCC

CAGATGCTTGATCACTGTCCCTTCCCAGGAACCTGAACTAGGAGCAGCTTGTCAGCACCT

CCTTGGGGGCTAAGCAGGAAGGTGCTTCTGCATGGTCTACAGGAGGGGGGGCAAAACCAG

TTATCTCTGGCTCCAGAGGTGGGAGTGGCTCCACCACTAGGGGTCCGTGAACAGACACCA

CTTGCAGCCCCATTCACCTCAAGGGCACCACAGAGAGGGCATTGTGACTGAACACCACCT

GTTGGTGCTCTCCACCCCTGGGAACACACCGCCCTGCTGCTGCCACTGCCAAATGCTCTG

GGCAGTACCCACAGCCTGATCTCTGTCACTTCCCAGGATCCTGCAACTAGGAGCAGCCTG

TACAGCACCTCCTATGTGGGCTAAGTGAGACAGGTTGCTTCATGCATGGTCTACAGGTGG

TGGGGGCAAACCACCACAGTTATCACTGACTCTAGAGATGGTCATGGCCTGCTCCACTAG

GGGTCCCTGAACAGGCACCACCTGCGTTCCAATCACCTCAGAGGAGGGCATTGCAATCAA

ACACAAGCTGTTGTTGCTCTCACTCCCCTGGGAACTCACACCCTGCTGCTGCTACTGCCA

AATGCTCTGGTCACCTTGTAGATCTGCCTAGAGCTCATTACCACTTCCCAGGGCCCTGCA

ACTAGGAGTAGCCTGTGCCACCTTCCTGCAGGTCCTTGCTGCTGTTGAGAGCCCAACAAC

CAGGCACTGACTGCTGGCCCTACCCATTGCCTCCTTCTCCCTGAAAACACACTGAGCATC

CTGGGAATAACAGCCTGCTCACACCAAGAAAGAGACAGCAAATATTCAAACTCCCACACC

AAAAATAAATAGTAACCCCCCCAAAAATACAAAGGGGTATTCTTGCATAGAAGTAGCCTT

ACAAGACTACAGTTTGGCTTCCCCAAACTCACAGAAAAAGAAAAACACAAGCAAGATGAA

GAAGCTCAGAAACCATTCCCAGTTAAAGAACAGGAGAATTCACCTAAAGCAGTCAACAAT

GAAACAGACCTCTGCAGTCTGACAGACATTGAGTTCAAAAGGGAGATAGTGAAAATACTG

AAGGAATTAAGAGAGGATATGAACAGTAATGCAGATTCCTTTAGAAAGGAACTAGAAAAT

ATAAGGAGGAGCCAAGAAAAACTAGAAAATTCATTTGCAGAGATACAAACTGAGCTAAAG

GCAATAAAGAGCAGAATGAATAATGCAGAGGAAGAATTAGTGACGTGGAAGATAGAATAA

TGGAAATCACCCAATCAGGACAGCAGACAGAAAACCAAATGAAAAAAACATGAAAGCAAT

ATAAGAGACCTATGGGATAATATAAAGTGGGCCAATCTATGCATAATAGGAATTCCAGAA

GGAAAGAAAAAGAAAAGGGGATTGAAAATATATTTGAAGAAATTATGGCTGAAAACTTTC

CAAATCTAAAGGATACTGATATCAAGATACAGGAAGCACAGAGGGCCCCAAACAAGTTGA

ACCCAAACAGGCCCACACCAAGACATATTATAATAAAAATGGCAAAAGTTAAAGATAAAG

AGAGGATTCTAAAGGCAGCAAGAGAAAAGCAAAGCATTAATTATAAGGGAACCCCCATAA

GGCTATCAGCTGATTTCTCTACAGAAACACTACAGGCCAGAAGGGAGTGGCAAGATATAT

TTAAAGTGCTGAAAGGAAAAAATTTGCAACCTAGAATACTCTATCCAGCAAGAATATCAT

TTAAAATAGAAGGGGAAATAAAGAATTTCTCCAACAAACAAAAGCTAAAAGAGTACAGCA

ATACTAAACCCATTCTAAAAGAAATACTGAAAGGGCTTCTCTAAATTAAAAAAAAAAAAA

AAAAGAATTAGGATGGAGGAAACCACAATTGGAAAGCAGTCACTTAAATAAGCCAGCATA

CAGATCTAAACATGAAGATGTTAAAAAAAAAAAAGACATCAAAATCATACAATGTGGGGA

AGGAAAGTAAGAAAATATAGATTCTTTTTTTATTTTAATGATGTGTTTGAGCCTATATGA

CTATCAGGCTAAAGCAAGCAGATATAGGAAGGGGTTAACATACTTAAAAAACAGGGCAAC

CACAAATCAAAACCAAACATTACATTCACAAAAACTGAAAAGAAAAGTACTCAAGCATAA

AATAAATGGAAACCATCCAACCAAAAAAAAAAAGAAAAGAGAAACATAGAATCAACTGGA

AAACAAGGTTTAAAATGGCAATAAATACATATCTATCAATAATCACCTTAAATGTCAATG

GACTGAATGCTCCAATCAAAAGACACAGAGTGGCAGATTGGATAAAAAAGCAAAAACCTT

CAATCTGCTGTCTACAAGAAACTCACCTTAGGGCAAAGGACACATATAGATTGAAAGTGA

GGGGATGGGAAAAGATATTTCATGCCAATGGACAAGACAGGAAAGCAGGAGTTGCAATAC

TCATATCAGACAAAATAGACTTTAAAATGAAGGCCATAAAGAAAGACAAAGAAGGACACT

ATTTAATGGTTAAAGGATCCATTCAAGAAGAGGATATTACAATCTCAATATATATGCCCC

TAATATAGGAGCACCCAGATACCTCCAACAAATACTAACAGACATAAAAGGAGAAATTGA

TGGGAATACAATCATAGTAGGAGACTTTAACACCCCACTCACATCAATGGACAGATCCTC

TAGACAGAAAATCAATAAGGCAACAGAGATCCTAAAGGACACAATAGAAAAGTTAGACTT

AATTGACATTTTCAGGACATTACATCCAAAAAAATAGAATATACATTCTTCTCAAGTGCA

CATGGAACATTCTCAAGAATTGATCACATACTGGGGCACAAAGCTAACCTCAACAAATTT

AAGAGTATAGAAATTATTTCAAGTATCTTCTCTGACCACAATGGCATGAAACTAGAAATC

AACCACAGGAAAAGAAATGAGAAAAAACTGACTACATGGAGACTAAACAACATGCTACTA

AAAAACCAATGGGTCAATGAGGAAATCAAGAAGGAAATTAAAAAATACCTCGAGACAAAT

GATAATGAAGACACAACCACTCAAAATCTATGGGATGCTGCAAAAGCAGTGCTCAGAGGG

AAATTCATAGCAATACAGGCCTTCCTCAAAAAAGAAGAAAAATCTCAAATCGACAACTTA

ACCCACCACCTAAATGAATTAGAAAAAGAAGAACAAACAAAACCTAAAGTCAGCAGAAGG

AAGGAAATCATAAAGATCAAAGAGGAAATCAATAAAATAGAGATTAAAAAAACAATAGAA

AAAATCAATAAAACCAAGAGCTGGTTCTTTGAAAAGGTAAACAAAATTGACAAACCTCTG

GCTAGACTCACCAAGAAGAGGAGAGAAAAAACCCAAATAAACAAAATAAGAAATGAAAAA

GGAGAAGTCACAACGATACTACAGAAATACAAAAAACATGAGAGAATACTATGAACAATT

GTATGCCAACAAATTTGACAACCTAGAAGAAATGGACAACTTTCTAGAGACTTACAGCCT

GCCAAAACTGAATCAAGAAGAAATAGATCAACTGAACAGACCGATCACTAGAAATGAAAT

TGAATATGTCATAAAAACACTCCCTACAAATAAAAGTCCAGGACCAGATGGCTTCACAGG

TGAATTCTACCAAACATACAAAGAGGAACTTATACCCATCCTCCTTAAACTTTTTCAAAA

GGTTGAAGAAGAAGGAACACTCCCAAAGACATTCTATGATGCCACCATCACCCTAATTCC

AAAACCAGACAAAGATACCACCAAAAAAGAAAACTATAGGCCAATATCTTTGATGAATAT

AGACACAAAAATTCTCAACAAAATTTTAGCCAACTGAATCCAACAACATATAAAAAAGAT

CATACACCACGACCAGGTGGGATTCATCCCAGGTTCACAAGGATGGTTCAACATATGCAA

ATCAATCAACGTCATACACCACATTAACAAAAGAAAAGTCAAAAACCACATGATCATCTC

AATAGATGCAGAAAAAGCATTTGACAAAGTCCAACATCCATTCATGATCAAAACTCTTAC

CAAAGTGGGTATAGAGGGAACATACCTTAACATAATAAAGCCATTTATGACAAACCCACA

GCAAATATAATACTCAATGGAGAAAAGCTGAAAGCCTTCCCACTAAAATCTGGAACAAGA

CAAGGATGCCCACTCTCACCACTGTTATTCAACATAGTACTGGAAGTCCTAGCCACAGCA

ATCAGACAAACAAAAGAAATAAAAGGCATCCAAATAGGAAGAGAAGAGGTAAAACTGTCA

CTGTATGCAGATGACATGATACTATATATAGAAAACCCTAAGGACTCAACCCAAAAACTA

CTTGAACTGATCAACAAATTCAGCAAAGTAGCAGGATATAAGATTAACATTCAGAAATCA

GTCATTTCTGTATACTAACAATGAAATATTAGAAAAGGAATACAAAAATACAATACCTTT

TAAAATTGCACCCCAAAAAATCAAATACCTGGGAATACACCTGACCAAGGAGGTAAAAGA

CTTATATGCTGAGAACTATAAAACATTAATCAAGGAAATTAAAGAAGATGTAAAGAAATG

GAAAGATATTCCATGCTCCTGGGTTGGAAAAATTAATATTGTAAAAATGGCCATACTACC

CAAAGCAATCTACAGATTCAATGCAATCCCTATCAAATTACCCATGACATTTTTCACAGA

ACTAGAACAAACAATCCAAAAATTTATATGGAACCACAAAAGACCCAGAATTGCCAAAGC

AATCTGAGGAACAAAAACCAAGCAGGAGGCATAACTCTCCCAGACTTCAGGCAATATTAC

AAAGCCACAGTCATCAAGACAGTGTGGTACTGGTACCAAAACAGACATACAGACCAATGG

AACAGAATAGAGAACCCAGAAATAAACCCAGACACCTATGGTCAATTAATCTTTGACAAA

GGAGGCAAGAACATAAAATGGGAAAAAGACAGTCTTTTCAGCAAGATTGCTGGGAAACCT

GGACAGCTGCATGCAAATCAATGAAACTAGAACACACCCTCACACCATGCACAAAAATAA

ACTCAAAATGGCTGAAAGACTTAAATATAAGACAAGACACCATCAAACTCCTGGAAGAGA

ACATAGGCAAAACATTCTCTGACATCAACCTTACAAATATTTTCTCAGGTCAGTCTCCCA

AAGCAACAGAAATAAAAGCAAAAATAAACCAATGGGACCTAATCAAACTGACAAGCTTTT

GCACAGCAAAGGAAACCAAAAAGAAAACAAAAAGACAACTTACAGAATGGGAGAAAATAG

TTTCAAATGATGCAACTGACAAGGGCTTAATCTCTAAAATATACAAGCAACTTATACAAC

TCAACAGCAAAAAAGCCAACAACCCAATGAAAAATGGGCAAAAGACCTGAATAGACATTT

CTCCAAGGAAGATATACAGATGGCCAACAAGCACATGAAAAAATGCTCAACATCCCTGAT

TATTAGAGAAATGCAAATCAAAACTACCATGAGATACCACCTCACACCAGTCAGAATGGC

CATCATTAATAAGTCCACAAATAACAAATGCTGGAGGGGGTGTGGAGAAAAGGGAACCCT

CCTGCACTGTTGGTGGGAATGTAAGCTGGTACAACCACTATGGAGAACAGTATGGAGGTA

CCTTAGAAATCTATACATAGAACTACCATATGACCCAGCAATCCCACTCTTGGGCATATA

TCCAGACAAAACTTTCCTTAAAAAGACACATGCACCCCATGTTCATTGCAGCACTATTCA

CAATAGCCAAGACATGGAAACAACCCAAATGTCCATGACAGATGATTGGATTAGGAAGAT

GTGGTATATATACACAATGGAATACTACTCAGCCATAAAAAAGAACAAAATAATGCCATT

TGCAGCAACATGGATGGAACTAGAGACTCTCATCTGAGTGAAGTAAGTCAGAAAGAGAAA

GACAAATACCATATGATATCACTTATATCTGGAATCTAATATAGGCACAAATGAACCTTT

CCACAGAAAAGAAAATCATGGACTTGGAGAATAGACTTGTGGTTGCCAAGGGGGAGGGGG

AGGGAGTGGGATGGATTGGGAGCTTGGGGTTAATAGATGCAACTATTGCCTTTGGAATGG

ATTAGCAATGAGATCCTGCTGTGTAGCACTGGGAACTATGTCTAGTCACTTATGATGGAG

CATGATAATGTGAGAAAAAAGAATGTATACATGTATGTGTAACTGGGTCACCATGCTGTA

CAGTAGAAAAAAAATATATTGGAATAAAAAAAAAAAAAAATAAATAAAAAAAAAAAAAAA

>L1B3#LINE/L1B

GGGGGCGGGCAAGATGGCGGAAGAGTAAGAGGTCACGCTCACCCTCTCCCACAAACACAT

CAAAAAAACACATCTACATGTAAAACGACTGCACAGAACATCAACTGAATGCTGGCAGAA

GAACTTAAACCTCCAAAAAGGGCAAGAAACTCTTGACATAACTGGGTAGAACAAAAGAAA

AAAAGAGAGAGAGAAAAAGGAATCAGGATGGGACTAGCATTCCTGAGAGGGAGCTGTGAA

GGAGAAAAGGAACCCACACCCTGGGAAGCCACCTAACGAGAAAGATCAGCCGAGTGAGGG

ACCTCAAAGATGCCGAGAAAAGTGCAGCAGCAGGCTGAGAACAGAAAAGCAGAGTGAGAG

AGCACAGATCATCTGAACCACTGGCCAGACACCACAGCCTGAGAGCTCGGGTGGGGGCTG

GGCGCTGAGACTTAGGCTCCAGAGGTCAGTCCCTGGGAGAGGGCTGGGGTTGGCGGTGTG

GAGACAGCCTAAGGGGCTAAGGAGCAGTGCGCCAGGCAGGGGAGCGTACTAAGGGCTGGG

GAGTGGAAAGCCATGGCAGAGGGAACCCGGGAGAAGGTCCGGACCCCAGGAGAGGCAAGG

CACCATTGTTGGGGAGGGGAGAGGAGGAGGGGCGACCCCATAGGAAACTCCTTGTGCCCA

GCTGCTGCGCCCCTCAGAGGGGAGCATCCCGCGCAGCCCCACCTAAGGCGCTCGGCGCAC

CCGGCGGAGAAGCCCTTGCTTTTCGAGACTGGGCCCTCTCCAGGACTGCAGGACCCTTGT

TGGGCTAGGTCAGGGGGCTAAGTGCACCAAATCTCCCCACCCGCACCGGGACGGATCTCC

GCAGGGAAGTGCTCAGCCCACGACCTGCAGGTGGGGGAGCAAACCACCAGTTATCCCTGA

CTCCAGAGGTGGGCGTGGCCCACCACCACTGGGGATATCTGAACAAAAATCACTTGCAGC

CCCAACCACTCAGAGGGCATCACAGAGAAGGACATACAACGAACACCACCTGATGTTGCT

CTCGCTCCCCGGGAGCACACCCCCCTGCAGCTGCCACTGCCAAACGTTCTGGGCAGTGCC

CAGATGCTTGATCACTGTCCCTTCCCAGGAACCTGCAACTAAGAGCACCTTGTGCAGCAC

ATCCTGTGGGGGCTAAGCAGGACAGGTGCTTCTTGCATGATTTACAGGTGGCGGGGGCAA

ACCACTGCAGTTATCTCTGGCTCCAGAGGTGGCAGTGGCCCGACACCACTAGGGGTCGTG

AACAGGCACCACTTGCAGCCCCATCACCTCAAGGGCACACAGAGGAGGGCATTGTGACCA

AACCAACTGTTGGTGCTCTGCACCCTTGGGAACACACCAGCCCTGCTGCTGCCACTGCTG

AATGTTCGGGCAGTACCCAATGCCAGATCTCTGTCACTTCCCAGGATCCTGCAACTAGGA

ACAGCCTGTGCAGCACCTCCTATGTGGGCTAAGTAGATGGGTTGCTTCATGCATGGTCTA

CAGGTGGAGAGGCAAACCACCACAGTTATCACTGACCTAGAGATGGTCATGGCCCACTCC

CACTAGGGGTCCCTGAACAGGCACCACCTGTGTTCCAATCACCTCAGAGGAGGGCACTGC

AATCAAACACAGCTGTTGTTGCTCTCACTCCCCTGGGAACTCATGCACCCTGCTGCTGCT

ACTGCCAAATGCTCTGGTCACCTTGTAGATCCTTCTAGAGCTCATTACCACTTCCCAGGG

CCCTGCAAATAGGAGTACCTGTGCCACCTTCCTGCAGGTCTTTGCTTCTGTTGAGAGCCC

AATGACCAGGCACTGACTGCTGGCCCTACCCATTGCCTCCTTCTCCCTGAAAACACACTG

AGCATCCTGGGGATAACAGCCTGCTCACACCAAAGAAAGAGACAGCAAAATTCAAACTCC

CACACCAAAAATAAATAGTAACCCCCCCCAAAAAATACAAAGGGGTATCTTGCATAGAAA

TAGCTTACAAGACTACAGTTTGGCTTCCCCAAACTCACAGAAAAAGAAAAACACAAGCAA

GATGAAGAAGCTCAGAAACCATTCCCAGTTAAAGCAAAGGAGAATTCACCTAAAGCAGTC

AACAATGAAACAGACCTCTGCAGTCTGACAGACATTGAGTTCAAAAGGGAGATAGTGAAA

ATACTGAAGGAATTAAGAGAGGATATGAACAGTAATGCAGATTCCCTTAGAAAGGAACTA

GAAAATATAAGGAGGAGCCAAGAAAAACTAGAAAATTCATTTGCAGAGATACAAACTGAG

CTAAAGGCAATAAAGACAGAATGAATAATGCAGAGGAATGAATTAGTGACGTGGAAGATA

GAATAATGGAAATCACCCAATCAGGACAGCAGACAGAAAACCAAATGAAAAAACATGAAA

GCAATATAAGAGATCTATGGGATAATATAAAGCAGGCCAATCTACCATAATAGGAATTCC

AGAAGGAGAAGAAAAAGAAAAGGGGATTGAAAATATATTTGAAGAAATTATGGCTGAAAA

CTTTCCAAATCTAAAGGAACTGATATCAAGATACAGGAAGCACAGAGGGCCCCAAACAAG

TTGAACCCAAACAGGCCCACACCAAGACATATTATAATAAAAATGGCAAAAGTTAAAATA

AAGAGAGGATTCTAAAGGCAGCAAGAGAAAAGCAAAGCATTAATTATAAGGGAACCCCAT

AAGGCTATCAGCTGATTTCTCTACAGAAACACTACAGGCCAGAAGGGAGTGGCAAGATAT

ATTTAAAGTGCTGAAAGGAAAAAATTGCAACCTAGAATACTCTATCCAGCAAGAATATCA

TTTAAAATAGAAGGGGAAATAAAGAATTTCTCCAACAAACAAAAGCTAAAAGAGTACAGC

AATACTAAACCCATTCTAAAAGAAATACTGAAAGGGCTTCTCTAAATTAAAAAAAAAAAA

AAGGAAGAACTAGGATGGAGGAAACCACAATTGGAAAGCAGTCACTTAAATAAGCCAGCA

TACAGATCTAAACATGAAGATGTTAAAAAAAAAAAAAAGACATCAAAATCATACAATGTG

GGAAGGGAAGTAAGAAAAAATAGATTCTTTTTTTTTTTTTTATGTTGTGTTTGAGCCTAT

ATGATATCAGGCTAAAGCAAGCAGATATAGGAAGGGGTTAACATACTTAAAAAACAGGGC

AACCACAAATCAAAACCGAACATTACATTCACAAAAACATGAAAAGTACTCAAGCATAAA

ATAAATGGAAACCATCCAACCAAAAAAAAAAAAAGAAAAGAGAAACATAGAATCAACTGG

AAAACAAGGTTTAAAATGGCAATAAATACATATCTATCAATAATCACCTTAAATGTCAAT

GGACTGAATGCTCCAATCAAAAGACACAGAGTGGCAGATTGGATAAAAAAGCAAAAACCT

TCAATCTGCTGCTACAAGAGACTCACCTTAGGGCAAAGGACACATATAGATTGAAAGTGA

GGGGATGGGAAAAGATATTTCATGCCAATGGACAAGACAGGAAAGCAGGAGTTGCAATAC

TCATATCAGACAAAATAGACTTTAAAATGAAGGCCATAAAGAAAGACAAAGAAGGACACT

ATTTAATGGTTAAAGGATCCATTCAAGAAGAGGATATTACAATCGTCAATATATATGCCC

CTAATATAGGAGCACCCAGATACCTACAACAAATACTAACAGACATAAAAGGAGAAATTG

ATGGGAATACAATCATAGTAGGAGACTTTAACACCCCACTCACATCAATGGACAGATCCT

CTAGACAGAAAATCAATAAGGCAACAGAGATCCTAAAGGAAACAATAGAAAAGTTAGACT

TAATGACATTTTCAGGACATTACATCCAAAAAAATCAGAATATACATTCTTCTCAAGTGC

ACATGGAACATTCTCAAGGATTGATCACATATGGGGCACAAAGCTAACCTCAACAAATTT

AAGAGTATAGAAATTATTTCAAGTATCTTCTCTGACCACAATGGCATGAAACTAGAAATC

AACCACAGGAAAAGAAATGAGAAAAAACTTACTACATGGAGACTAAACAACATGCTACTA

AAAAACCAATGGGTCAATGAGGAAATCAAGAAGGAAATTAAAAAATACCTCAGACAAATG

ATAATGAAGACACAACCACTCAAAATCTATGGGATGCTCAAAAGCAGTGCTCAGAGGGAA

GTTCATAGCAATACAGGCCTTCCTCAAAAAAGAAGAAAAATCTCAAATGACAACTTAACC

CACCACCTAAATGAATTAGAAAAAGAAGAACAAACAAAACCTAAAGTCAGCAGAAGGAAG

GAAATCATAAAGATCAGAGAGGAAATCAATAAAATAGAGATTCAAAAAACAATAGAAAAA

ATCAATAAACCAAGAGCTGGTTCTTTGAAAAGGTAAACAAAATTGACAAACCTCTGGCTA

GACTCACCAAGAAGAGGAGAGAAAAAACCCAAATAAACAAAATAAGAAATGAAAAAGAGA

AGTCACAATGGATACTACAGAAATACAAAAAAACAATGAGAGAATACTATAACAATTATA

TGCCAACAAATTTGACAACCTAGAAGAAATGGACAACTTTCTAGAGACTTACAGCCTGCC

AAAACTGAATCAAGAAGAAATAGATCAACTGAACAGACTGATCACTAGAAATGAAATTGA

ATATGTCATAAAAACACTCCCTACAAATAAAAGTCCAGGACCAGATGGCTTCACAGGGAA

TTCTACCAAACATACAAAGAGGAACTTATACCCATCCTCCTTAAACTTTTTCAAAAGGTT

GAAGAAGAAGGAACACTCCCAAAGACATTCTATGATGCCACCATCACCCTAATTCCAAAA

CCAGACAAAGATACCACCAAAAAAGAAAACTATAGGCCAATATCTTTGATGAATATAGAG

CAAAAATTCTCAACAAAATTTTAGCCAACCGAATCCAACAACATATAAAAAAGATCATAC

ACCACGACCAGGTGGGATTCATCCCAGGTTCACAAGGATGGTTCAACATAGCAAATCAAT

CAACATCATACACCACATTAACAAAAGAAAAGTCAAAAACCACATGATCATCTCAATAGA

TGCAGAAAAAGCATTTGACAAAGTCCAACATCCATTCATGATAAAAACTCTTACCAAAGT

GGGTATAGAGGGAACATACCTTAACATAATCAAAGCCATTTATGACAAACCCACAGCAAA

TATAATACTCAATGGAGAAAAGCTGAAAGCCTTCCCACTAAAATCTGGAACAAGACAAGG

ATGCCCACTCTCACCACTGTTATTCAACATAGTACTGGAAGTCCTAGCCACAGCAATCAG

ACAAACAAAAGAAATAAAAGGCATCCAAATAGGAAGAGAAGAGGTAAAACTGTCACTGTA

TGCAGATGACATGATACTATATATAGAAAACCCTAAGGACTCAACCCAAAAACTACTTGA

ACTGATCAACAAATTCAGCAAAGTAGCAGGATATAAGATTAACATTCAGAAATCAGTCCA

TTTCTGTATACTAACAATGAAATATTAGAAAAGGAATACAAAAATACAATACCTTTTAAA

ATTGCACCCCAAAAAATCAAATACCTGGGAATACACCTGACCAAGGAGGTAAAAGACTTA

TATGCTGAGAACTATAAAACATTAATCAAGGAAATTAAAGAAGATGTAAAGAAATGGAAA

GATATTCCATGCTCCTGGGTTGGAAAAATTAATATTGTAAAAATGGCCATACTACCCAAA

GCAATCTACAGATTCAATGCAATCCCTATCAAATTACCCATGACATTTTTCACAGAACTA

GAACAAACAATCCAAAAATTTATATGGAACCACAAAAGACCCAGAATTGCCAAAGCAATT

CTGAGGAACAAAAACCAAGCAGGAGGCATAACTCTCCCAGACTTCAGGCAATATTACAAA

GCCACAGTCATCAAGACAGTGTGGTACTGGTACCAAAACAGACATACAGACCAATGGAAC

AGAATAGAGAACCCAGAAATAAACCCAGACACCTATGGTCAATTAATCTTTGACAAAGGA

GGCAAGAACATAAAATGGGAAAAAGACAGTCTTTTCAGCAAGTATTGCTGGGAAACCTGG

ACAGCTGCATGCAAATCAATGAAACTAGAACACACCCTCACACCATGCACAAAAATAAAC

TCAAAATGGCTTAAAGACTTAAATATAAGACAAGACACCATCAAACTCCTGGAAGAGAAC

ATAGGCAAAACATTCTCTGACATCAACCTTACAAATGTTTTCTCAGGTCAGTCTCCCAAA

GCAACAGAAATAAAAGCAAAAATAAACCAATGGGACCTAATCAAACTGACAAGCTTTTGC

ACAGCAAAGGAAACCAAAAAAAAAACAAAAAGACAACTTACAGAATGGGAGAAAATAGTT

TCAAATGATGCAACTGACAAGGGCTTAATCTCTAAAATATACAAACAACTTATACAACTC

AACAGCAAAAAAGCCAACAACCCAATGAAAAATGGGCAAAAGACCTGAATAGACATTTCT

CCAAGAAGATATACAGATGGCCAACAAGCACATGAAAAAATGCTCAACATCACTGATTAT

TAGAGAAATGCAAATCAAAACTACCATGAGATACCACCTCACACCAGTCAGAATGGCCAT

CATTAATAAGTCCACAAATAACAAATGCTGGAGGGGGTGTGGAGAAAAGGGAACCCTCCT

GCACTGTTGGTGGGAATGTAAGCTGGTACAACCACTATGGAGAACAGTATGGAGGTACCT

TAGAAAACTATACATAGAACTACCATATGACCCAGCAATCCCACTCTTGGGCATATATCC

AGACAAAACTTTCCTTGAAAAAGACACATGCACCCGCATGTTCATTGCAGCACTATTCAC

AATAGCCAAGACATGGAAACAACCCAAATGTCCATTGACAGATGATTGGATTAGGAAGAT

GTGGTATATATACACAATGGAATACTACTCAGCCATAAAAAAGAACAAAATAATGCCATT

TGCAGCAACATGGATGGAACTAGAGACTCTCATACTGAGTGAAGTAAGTCAGAAAGAGAA

AGACAAATACCATATGATATCACTTATATCTGGAATCTAATATACGGCACAAATGAACCT

TTCCACAGAAAAGAAAATCATGGACTTGGAGAATAGACTTGTGGTTGCCAAGGGGGAGGG

GGAGGGAGTGGGATGGATTGGGAGCTTGGGGTTAATAGATGCAGACTATTGCCTTTGGAA

TGGATTAGCAATGAGATCCTGCTGTGTAGCACTGGGAACTATGTCTAGTCACTTATGATG

GAACATGATAATGTGAGAAAAAAGAATGTATACATGTATGTGTAACTGGGTCACCATGCT

GTACAGTAGAAAAGAAAAATAAAAGCATAAAAAAAAAAATAAAAAAAAAATATAAAAAAA

AA

>L1B4#LINE/L1B

GGAAGGATCAAGATGGCAGAGGAGTAAGAGTGTGCTCACCTTCTCCCACAAACACATCAA

AAAAAAAATCTACATGTAGAAGATTCACACAGAACATCTACTGAATGCTGGCAGAAGAAC

TTAAACCTCCAAAAAGGGCAAGAAACCCTCCACATAACTGGGTAGAACAAAAGAAAAAAA

AGAGAGAGAGAAAAGGAATCAGGATGGGACTAGCATTCCTGAGAGGGAGCTGTAAGAGAA

AAGGAACCCACACCCTGGGAAGCCACCTAACCGAGGGAGATCAGCAGATGGAGGGACCTC

AAAGTCACTGAGAAAAGCACAGCAGCTGGACTGAGGAGGGCAAAGCAGAGTGAGAGCCAC

ACAGATCATCTGCACCACTGCCCGGACACCACAGCCTGAGATGCTCGGGGGGGCTGGGCA

CTGAGACTCAGGCTCCGAGGTCAGTTCTGGGAGAGGACTAGGTTGGCTGTGTGGAGACAG

CCTGAGGGGCTAGGGAGCAGTGTGCCATGGGCTGGGGAGAGAAACCACAGCGAGGGAACC

CAGGAGGAGGTCTGGGCCCAGGAGAAGCAAGGGCCATTGTTGGGGAGGGTGAGAGGAGGA

GGGGTGGACCACCATAGGAATCTCTTTCCCTGTGCACGGGCTCTCAGAGGGTGGGGCACC

TCTGGCCAGGCTATGGGTGGCGAGAGGCCACTTGCTGGCTACAGGAGACGGGGCTCTTGT

GGGCTAGGGTGGGGGACTCTTGTGGGGCTAAGGGCAGGGGGTAAGTGAGTGGTCCTCTTG

GTGATCTACAGGAGCAGGGACAAACCACAGCAGTCATCTCAGAAACCAGAGGGAGGTGGC

CTCCACCACTAGGGTCTGTGAAAGGCTCCACCTGGGCCCCAGTCACCTCAGAGGTGGCAA

AAAAAGGGCACTGCAACAGCACCACCCGTTGTTGCTCTCACTCCCCTGGGAACACACCCC

TGCTGCTGCCACTGCCAAAGCTCTGGGCAGCCCACAATGTGATCACTGTCACTTCCCAGG

ACCCTGCAACTAGGAGCAGCCTGTGCAGCACCTCCTGCTGGGCTAAGGGGAGGGGTGCTT

CTTGCATGGTCTACAGGTGGCAGGGGCAAACCACTGCAGTTATCTCTGACTCCAGAGGTG

GGCATGGCCCCACCACTAGGGGTCCTGAACAGGCACCACTTGCGGCCCCAATCACCTCAG

AGGGCACCACAGAGGAGGGCATTGTAACAACACCACCTGTTGTTGCTCTCACTCCCCTGG

GAACTCACACACCCTGCTGCTGCCACTGCCAAATGCTCTGGGCACCCTAAATCTGCCTGA

GGCTCATTACCACTTCCCAGGGCCCTGCAACTAGGAGTAGCCTGTGCCACCTTCCTGCAG

GTCCTTGCTACTGTCAAGAGCCCAGCAACCAGGCACTGACTACTAGCCCTACCCATTGCC

TCCTTCTCCCTGGAAACACACTCAGCACCCTGGGGATAACAGCCTGCTCACACTAAGAAA

GAGACAGCAAATATCCAAACTCCCACCAAAAATAAATAGTAACCCCCCACAAAATACAAA

GGGGACTCTTGCATAGAAATAGCCCTCCAAGACTACAGTTTGGTTTCCCTAAACTCACAG

AAAAAGAAAAAATAAGCAAAATGAAGAAGCTCAGGAACCATTCCCAGTTAAAAGAACAGG

AGAATTCACCTGAAGCAGCAAACAATGAAACAGACCTCTGCAGTCTAATAGACACTGAGT

TCAAAAGGGAGATAGTGAAAATACTGAAGGAATTAAGGTGAATATGAAGGAATTAAGAGA

GATATAACAGTAATGCAGATTACTTTAGAAAGGAACTAGAAAATATAAGGAGGAACCAAG

AAAAATTAGAAAATTCATTTGCAGAGATGCAAACTGAGTTAAAGGCACTAAAGAGCAGAA

TGAATAATGCAGAGGAATGAATTAGTGACTTGGAAGATAGAATAATGGAAATCACCCAAT

CAGGACAGCAGACAGAAAACCAAATGAAAAAAATGAAAGCAATATAAGAGATCTATGGGA

TAATATAAAGCAGGCCAATCTAGCATAATAGGAATTCCAGAAGGAGAAGAAAAAGAAAAG

GGGATTGAAAATATATTTGAAGAAATTATGTCTGAAAACTTTCCAAATCTAAAGGAAACG

ATATCAAGATACAGGAAGCACAGAGGGCCCCAAACAAGTTGAACCCAAACAGGCCCACAC

CAAGACATATTATAATAAAAATGGCAAAAGTTAAAGATAAAGAGATTCTAAAGGCAGCAA

GAGAAAACAAAGCATTAATTATAAGGGAACCCCCATAAGGCTATCAGCTGATTTCTCTAC

AGAAACACTACAGGCCAGAAGAGAGTGGCAAGATATATTCAAAGTTCTAAAAGGGAAAAA

TTTGCAGCCTAGAATACTCTACCCAGCAAGAATATCATTTAAAATAGAAGGAGAAATAAA

GAATTTCTCCAACAAACAAAACTAAAAGAGTACAGCAATACTAAACCCATTCTAAAAGAA

ATACTGAAAGGGCTTCTCTAAATAAAAAAGAAGTAAGAAGAAATAGGATGGAGGAAATCA

CAATTGGAAAGCAATCACTTAAATAAGCCAGTATACAGATCTAAAAGAAAAAAAAAACCT

ATGTAAAAGTGAGATAAACACAAGGAACAGCAAAAGGACAAACATGAAGATGTTAAAAAA

GGACTTCAAAATCATAGAATGTGGGGAAGGAAAGTAAGAAAATCTAGACTCTTTTTTTTT

AAAATGTGTTTGAGCCTATATGACTATCAGGCTAAAGCAAGCAGATATAGGAAGGGGTTA

ACATACTTAAAAAACAGGGCAACCACAAATCAAAACCAAACATTACATTCACAAAAACTA

AAAAGAAAAGACACAAGCATAAAATAAATGGAAATCATCCAACCAAAAAAAAAAGGAACA

AAGGAGAAACATAGAATCAACTGGAAAACAAGGTTTAAAATGGCAATAAATACATATTTA

TCAATAATTACCTTAAATGTCAATGGACTGAATGCTCCAATCAAAAGACACAGAGTGGCA

GATTGGATAAAAAAGCAAGAGCCTACAATATGCTGTCTACAAGAGACTCACCTTAGGGCA

AAGGACACATATAATTGAAAGTGAGGGGATGGGAAAAGATATTTCATGCCAATGGAAAGA

CAGGAAAGCAGGAGTTGCAATACTCATATCAGACAAAATAGACTTTAAAATGAAGGCCAT

AAAGAAAGACAAAGAAGGACACTATTTAATGATAAAAGGATCCATTCAAGAAGAGGATAT

TACAATCATCAATATATATGCCCCTAATATAGGAGCACCCAGATACTACAACAAATACTA

ACAGACATAAAAGGAGAAATTGATGGGAATACAATAATAGTAGGAGACTTTAACACCCCA

CTCACATCAATGGACAGATCCTCTAGACAGAAAATCAATAAGGCAACAGAGATCCTAAAT

GACACAATAGAAAAGTTAGACTTAATTGACATTTTCAGGACATTACATCCAAAAAAAATC

AGAATATACATTCTTTTCAAGTGCACATGGAACATTCTCAAGGATTGACCACATACTGGG

GCACAAAACTAACCTCAACAAATTTAAGAGTATAGAAATTATTTCAAGTATCTTCTCTGA

CCACAATGGCATGAAACTAGAAATCAACCACAGGAAAAGAAATGAGAAAAAACTGACTAC

ATGGAGACTAAACAACATGCTACTAAAAAACCAATGGGTCAATGAGGAAATCAAAAAGGA

AATTAAAAAATACCTCGAGACAAATGATAATGAAGACACAACCATTCAAAATCTATGGGA

TGCTCAAAAGCAGTGCTTAGAGGGAAATTCATAGCAATACAGGCCTTCCTCAAAAAAGAA

GAAAAATCTCAAATCAACAACTTAACCCACCACCTAAATGAATTAGAAAAAGAAGAACAA

ACAAAACCTAAAGTCAGCAGAAGGAAGGAAATCATAAAGATCAGAGAGGAAATCAATAAA

ATAGAGATTCAAAAAACAATAGAAAAAAATAATAAAACCAAGAGCTGGTTCTTTGAAAAG

GTAAACAAAATTGACAAACCTCTGGCCAGACTCACCAAGAAGAGGAGAGAAAAAACCCAA

ATAAACAAAATAAGAAATGAAAAAGGAGAAATCTCAAGGATACTGCAGAAATACAAAAAA

TAAGAGAATACTATGAACAATTATATGCCAACAAATTTGACAACCTAGAAGAAATGGACA

ACTTTCTAGAGACTTACAGCCTGCCAAAACTGAATCAAGAAGAAATAGATCAACTGAACA

GACTGATCACTAGAAATGAAATTGAATATGTAATAAAAACACTCCCTACAAACAAAAGTC

CAGGACCAGATGGCTTCACAGGTGAATTCTACCAAACATACAAAGAAGAACTTATACCCA

TCCTTCTTAAACTTTTCCAAAAGGTTGAAGAAGAAGGAACACTCCCAAAGACATTCTATG

AAGCCACCATCACCCTAATACCAAAACCAGACAAAGATACTACCAAAAAAGAAAATTATA

GGCCAATATCTTTGATGAATATAGATGCAAAAATTCTCAACAAAATTTTAGCCAACCAAA

TCCAACAACATATAAAAAAGATCATACACCAGACCAAGTGGGATTCATCCCAGGTTCACA

AGGATGGTTCAACATATGCAAATCAATCAACTCATACACCACATTAACAAAAGAAAAGTC

AAAAACCACATGATCATCTCAATAGATGCAGAAAAAGCATTTGACAAAGTCCAACATCCA

TTCATGATAAAACTCTTACCAAAGTGGGTATAGAGGGAACATACCTTAACATAATAAAAG

CCATTTATGACAAACCCACAGCAAATATAATACTCAATGGAGAAAAGCTGAAAGCCTTCC

CACTAAAATCTGGAACAAGACAAGGATGCCCACTCTCACCACTTTTATTCAACATAGTAT

TGGAAGTCCTAGCCACAGCAATCAGACAAACAAAAGAAATAAAATGTATCCAAATTGGAA

GAGAAGAGGTAAAATTGTCACTGTATGCAGATGACATGATACTATATATAGAAAACCCTA

AGGACTCCACACAAAAACTACTTGAACTGATCAACAAATTCAGCAAAGTAGCAGGATACA

AGATTAACATTCAGAAATCGTTGCATTTCTGTATACTAACAATGAAATATTAGAAAAGGA

ATACAAAAATACAATACCTTTTAAAATTGCACCCAAAAAAATTAAATACCTGGGAATAAA

CCTGACCAAGGAGGTGAAAGACTTATATGCTGAGAACTATAAAACATTAATCAAGGAAAT

TAAAGAGGATTCAAAGAAATGGAAAGATATTCCATGCTCCTGGGTTGGAAAAATTAATAT

TGTAAAAATGGCCATACTACCCAAAGCAATCTACAGATTCAATGCAATCCCTATCAAATT

ACCCATGACATTTTTCACAGAACTAGAACAAACAATCCAAAAATTTATATGGAACCACAA

AAGACCCAGAATTGCCAAAGCAATCCTGAGGAACAAAAACCAAGCAGGAGGCATAACTCT

CCCAGACTTCAGGCAATATTACAAAGCCACAGTAATCAAGACAGTGTGGTACTGGTACCA

AAACAGACATACAGACCAATGGAACAGAATAGAGAACCCAGAAATAAACCCAGACACCTA

TGGTCAATTAATCTTCGACAAAGGAGGCAAGAATATAAAATGGGAAAAAGACAGTCTTTT

CAGCAAGTGTGCTGGGAAAACTGGACAGCTGCATGTAAATCAATGAAACTGGAACACACC

CTCACACCATGCACAAAAATAAACTCAAAATGGCTTAAAGACTTAAATATAAGACAAGAC

ACCATCAAACTCCTAGAAGAGAACATAGGCAAAACATTCTCTGACATCAACCTTACAAAT

GTTTTCTTAGGTCAGTCTCCCAAGGCAACAGAAATAAAAGCAAAAATAAACCAATGGGAC

CTAATCAAACTGACAAGCTTTTGCACAGCAAAGGAAACCATAAAAAAAAAAAGACAACTA

AGAATGGGAGAAAATAGTTTCAAATGATGCAACTGACAAGGGCTTAATCTCTAAAATATA

CAAACAACTTATACAACTCAACAGCAAAAAACCAACAACCCAATTGAAAAATGGGCAAAA

GACCTGAATAGACATTTCTCCAAAGAAGATATACAGATGGCCAACAAGCACATGAAAAAA

TGCTCAACATCACTAATTATTAGAGAAATGCAAATCAAAACTACTATGAGGTACCACCTC

ACACCAGTCAGAATGGCCATCATTAAAAGTCACAAATAACAAATGCTGGAGAGGGTGTGG

AGAAAAGGGAACCCTCCTCACTGTTGGTGGGAATGTAAATTGGTACAACCACTATGGAAA

ACAGTATGGAGGTACCTCAGAAAACTAAATATAGAACTACCATATGACCCAGCAATCCCA

CTCTTGGGCATATATCCAGACAAAACTTTCCTTGAAAAAGATACATGCACCTGTATGTTC

ATTGCAGCACTATTCACAATAGCCAAGACATGGAAACAACCTAAATGTCCATGACAGATG

AATGGATTAAGAAGATGTGGTATATATACACAATGGAATACTACTCAGCCATAAAAAAGA

ACAAAATAATGCCATTTGCAGCAACATGGATGGAACTAGAGACTCTCATACTAAGTGAAG

TAAGTCAGAAAGAGAAAGACAAATACCATATGATATCACTTATATCTGGAATCTAATATA

TGGCACAAATGAACCTTTCCACAGAAAAGAAACTCATGGACTTGGAGAACAGACTTGTGG

TTGCCAAGGGGGAGGGGGAGGGAGTGGGATGGACTGGGAGTTTGGGGTTAATAGATGCAA

ACTATTGCATTTGGAATGGATAAGCAATGAGATCCTGCTGTATAGCACAGGGAACTATAT

CTAGTCACTTGTGATGGAACATGATGGAGGATAATGTGAGAAAAAGAATGTATATATTAT

GTTACTGGGTCACTTTGCTGTACAGTAGAAAATGACAGAACACTGTAAACAACTATAATG

GAAAAAATAAAAATCATAAAAAAAAAAA

>L1B5#LINE/L1B

GAGAAGGATCAAGATGGTGGAGGAGTAAGAGTCGTGCTCACCTTCTCCCACAAACACATC

AAAAAAACACATCTACATGTAAACGATTCACACAGAACATCTACTGAATGCTGGCAGAAG

AACTTAAACCTCCAAAAAGGGCAAGAAACCCTACATAACTGGGTAGAACAAAAGAAAAAA

GAGAGAGAAAAGGAATCAGGATGGGACTAGCATTCCTGAGAGGGAGCTGTGAAAGAGAAA

AGGAACCCACACCCTGGGAAGCCACCTAACTGACGGGAGATCAGCCAGACAGAGGGACCT

CAAAGTCGCTGAGAAAAGCACAGCAGCTGGACTGAGGAGGGCAAAGCAGAGTGAGAGCCA

CACAGATCATCTGCACCACCCCCCAGACACCACAGCCTGAGATGCTCGGCGGGGGCTGGG

GCTGAGACTCAGGCTCTGGAGGTCAGTTCGGGGAGAGGACTAGGGTTGGCTGTGTGGAGA

CAGCCTGAGGGGCTAGGGAGCAGTGTGCCATGGGCTGGGGAGTGAACGCCAAGCAAGGGA

ACCCAGGAGGAGGTCTGGGCCCCAGGAGAAGCAAGGTGCCATTGTTGGGGAGGGGAGAGG

AGGAGGGGAGACGCCATAGGAATCTCTTCCCTGCACAGGGCTCTCAGAGGGTGGGGCGCC

TCTGGCCAGGCTACAGGTGGTGAGAAGCCACTTGCTCAGGCTAGGGAGACTGGGGCCTCT

TGTGAGGCTACAGGTGGCCAGGCACCTCTTGTGTGGGCTAAGGGCAGGGGGGCTAAGTGC

AATGTGGTGCCTCTTGCATGATCTACAGGGGGGACAAACCACAGCAGTCATCTCAGAAAC

CAGAGGGAGGCATGGCCTGCCACCACTGGGGTCTGTGAACAGGCTCCACCTGTGGCCCCA

GTCACCTCAGAGGTTGGCAAAAAAAGAGGGCACTGCAACTGAGCACCACCCATTGTTGCT

CTCACTCCCCTGGGAACACACCCCCCTGCTGCTGCCACTGCCAAATGCTCTAGGCAGCGC

CCACACATGCTTGATCACTGTCACTTCCCAGGACCCTGCAACTAGGAGCAGCCTGTGCAG

CACCTCCTGCATGGGCTAAGTGGGATGGGTGCTTCTTGCATGGTCTACAGGTGGCAGGGG

CAAACCACCACAGTTATCTCTGACTCCAGAGGTGGGCATGGCCCACCACCACTAGGGGTC

CTGAACAGGCACCACTTGGGCCCCAATCACCTCAGAGGGCACCACAGAGGAGGGCATTGA

ACTGAACACCACCTGTTGTTGCTCTCACTCCCCTGGGAACTCACACACCCTGCCCTGCCA

CTGCCAAATGCTCTGGGCACCCTAAATCTGCCTGAGGCTCATTACCACTTCCCAGGGCCC

TGCAACTAGGAGTAGCCTGTGCCACCTTCCTGCAGGTCCTTGCTACTGTCAAGAGCCCAG

CAACCAGGCACTGACTACTAGCCCTACCCATTGCCTCCTTCTCCCTGGAAACACACTCAG

CACCCTGGGGATAACAGCCTGCTCACACTGAAGAAAGAGACAGCAAATATCCAAACTCCC

ATGCCAAAAATAAATAGTAACCCCCCACAAAATACAAAGGGGTGCTCTTGCATAGAAGTA

GCCCTCCAAGACTACAGTTTGGTTTCCCTAAACTCACAGAAAAAGAAAAATATAAGCAAA

ATGAAGAAGCTCAGGAACCATTCCCAGTTAAAAGAACAGGAGAATTCACCTGAAGCAGCA

AACAATGAAACAGACCTCTGCAGTCTAATAGACACTGAGTTCAAAAGGGAGATAGTGAAA

ATACTGAAGGAATTAAGGTGAATATGAAGGAATTAAGAGGGATATAAACAGTAATGCAGA

TTACTTTAGAAAGGAACTAGAAAATATAAGGAGGAACCAAGAAAAATTAGAAAATTCATT

TGCAGAGATGCAAACTGAGCTAAAGGCACTAAAGAGCAGAATGAATAATGCAGAGGAATG

AATTAGTGACTTGGAAGATAGAATAATGGAAATCACCCAATCAGGACAGCAGACAGAAAA

CCAAATGAAAAAACATGAAAGCAATATAAGAGATCTATGGGATAATATAAAGAGGCCAAT

CTAGCATAATAGGAATTCCAGAAGGAGAAGAAAAAGAAAAGGGGATTGAAAATATATTTG

AAGAAATTATGTCTGAAAACTTTCCAAATCTAAAGGAAACTGATATCAAGATACAGGAAG

CACAGAGGGCCCCAAACAAGTTGAACCCAAACAGGCCCACACCAAGACATATTATAATAA

AAATGGCAAAAGTTAAAGAAAAGAGAGGATTCTAAAGGCAGCAAGAGAAAAACAAAGCTT

AATTATAAGGGAACCCCCATAAGGCTATCAGCTGATTTCTCTACAGAAACACTACAGGCC

AGAAGAGAGTGGCAAGATATATTCAAAGTTCTAAAAGGAAAAATTTGCAGCCTAGAATAC

TCTACCCAGCAAGAATATCATTTAAAATAGAAGGAGAAATAAAGAATTTCTCCAACAAAC

AAAAGCTAAAAGAGTACAGCAATACTAAACCCATTCTAAAAGAAATACTGAAAGGGCTTC

TCTAAATAAAAAAAAAGTAAGAAGAAATAGGATGGAGGAAATCACAATTGGAAAGCAATC

ACTTAAATAAGCCAGTATACAGATCTAAAAGAAAAAAAAAAAAACCTATTGTAAAAGTGA

TGATAAACACAAGGAACAGCAAAAGGACAAACATGAAGATGTTAAAAAAGGACTTCAAAA

TCATAGAATGTGGGGAAGGAAAGTAAGAAAATCTAGACTTTTTTTTTTATAATGTGTTTG

AGCCTATATGACTATCAGGCTAAAGCAAGCAGATATAGGAAGGGGTTAACATACTTGAAA

AACAGGGCAACCACAAATCAAAACCAAACATTACATTCACAAAAACTAAAAAGAAAAGAC

ACAAGCATAAAATAAATGGAAATCATCCAACCAAAAAAAAGAAAGGAACAAAGGAGAAAC

ATAGAATCAACTGGAAAACAAGGTTTAAAATGGCAATAAATACATATTTATCAATAATTA

CCTTAAATGTCAATGGACTGAATGCTCCAATCAAAAGACAAGAGTGGCAGATTGGATAAA

AAAGCAAAAGCCTACAATATGCTGTCTACAAGAGACTCACCTTAGGGCAAAGGACACATA

TAGATTGAAAGTGAGGGGATGGGAAAAGATATTTCATGCCAATGGACAAGACAGGAAAGC

AGGAGTTGCAATACTCATATCAGACAAAATAGACTTTAAAATGAAGGCCATAAAGAAAGA

CAAAGAAGGACACTATTTAATGATAAAAGGATCCATTCAAGAAGAGGATATTACAATCAT

CAATATATATGCCCCTAATATAGGAGCACCCAGATACATACAACAAATACTAACAGACAT

AAAAGGAGAAATTGATGGGAATACAATAATAGTAGGAGACTTTAACACCCCACTCACATC

AATGGACAGATCCTCTAGACAGAAAATCAATAAGGCAACAGAGATCCTAAATGACACAAT

AGAAAAGTTAGACTTAATTGACATTTTCAGGACATTACATCCAAAAAAAATCAGAATATA

CATTCTTTCAAGTGCACATGGAACATTCTCAAGGATTGATCACATACTGGGGCACAAAAC

TAACCTCAACAAATTTAAGAGTATAGAAATTATTTCAAGTATCTTCTCTGACCACAATGG

CATGAAACTAGAAATCAACCACAGGAAAAGAAATGAGAAAAAACACTACATGGAGACTAA

ACAACATGCTACTAAAAACCAATGGGTCAATGAGGAAATCAAGAAGGAAATTAAAAAATA

CCTGAGACAAATGATAATGAAGACACAACCATTCAAAATCTATGGGATGCCACAAAAGCA

GTGCTTAGAGGGAAATTCATAGCAATACAGGCCTTCCTCAAAAAAGAAGAAAAATCTCAA

ATACAACTTAACCCACCACCTAAATGAATTAGAAAAAGAAGAACAAACAAAACCTAAAGT

CAGCAGAAGGAAGGAAATCATAAAGATCAGAGAGGAAATCAATAAAATAGAGATTCAAAA

AACAATAGAAAAAAATCAATAAAACCAAGAGCTGGTTCTTTGAAAAGGTAAACAAAATTG

ACAAACCTCTGGCCAGACTCACCAAGAAGAGGAGAGAAAAAACCCAAATAAACAAAATAA

GAAATGAAAAAGGGGAAATCTCAATGGATACTGCAGAAAAAAAAAAACCATAAGAGAATA

CTATGAACAATTATATGCCAACAAATTTGACAACCTAGAAGAAATGGACAACTTTCTAGA

GACTTACAGCCTGCCAAAACTGAATCAAGAAGAAATAGATCAACTGAACAGACCGATCAC

TAGAAATGAAATTGAATATGTAATAAAAACACTCCCTACAAATAAAAGTCCAGGACCAGA

TGGCTTCACAGGTGAATTCTACCAAACATACAAAGAAGAACTTATACCCATCCTTCTTAA

ACTTTTCCAAAAGGTTGAAGAAGAAGGAACACTCCCAAAGACATTCTATGAAGCCACCAT

CACCCTAATACCAAAACCAGACAAAGATACTACCAAAAAAGAAAATTATAGGCCAATATC

TTTGATGAATATAGACCAAAAATTCTCAACAAAATTTTAGCCAACTGAATCCAACAACAT

ATAAAAAAGATCATACACCAGACCAAGTGGGATTCATCCCAGGTTCACAAGGATGGTTCA

ACATATGCAAATCAATCAACATCATACACCACATTAACAAAAGAAAAGTCAAAAACCACA

TGATCATCTCAATAGATGCAGAAAAAGCATTTGACAAAGTCCAACATCCATTCATGATAA

AAACTCTTACCAAAGTGGGTATAGAGGGAACATACCTTAACATAATAAAAGCCATTTATG

ACAAACCCACAGCAAATATAATACTCAATGGAGAAAAGCTGAAAGCCTTCCCACTAAAAT

CTGGAACAAGACAAGGATGCCCACTCTCACCACTTTTATTCAACATAGTATTGGAAGTCC

TAGCCACAGCAATCAGACAAACAAAAGAAATAAAAGTATCCAAATTGGAAGAGAAGAGGT

AAAATTGTCACTGTATGCAGATGACATGATACTATATATAGAAAACCCTAAGGACTCCAC

ACAAAAACTACTTGAACTGATCAACAAATTCAGCAAAGTAGCAGGATAAAGATTAACATT

CAGAAATCAGTTGCATTTCTGTATACTAACAATGAAATATTAGAAAAGGAATAAAAAATA

CAATACCTTTTAAAATTGCACCCCAAAAATTAAATACCTAGGAATAAACCTGACCAAGGA

GGTGAAAGACTTATATGCTGAGAACTATAAAACATTAATCAAGGAAATTAAAGAGGATTC

AAAGAAATGGAAAGATATTCCATGCTCCTGGGTTGGAAAAATTAATATTGTAAAAATGGC

CATACTACCCAAAGCAATCTACAGATTCAATGCAATCCCTATCAAATTACCCATGACATT

TTTCACAGAACTAGAACAAACAATCCAAAAATTTATATGGAACCACAAAAGACCCAGAAT

TGCCAAAGCAATCCTGAGGAACAAAAACCAAGCAGGAGGCATAACTCTCCCAGACTTCAG

GCAATATTACAAAGCCACAGTAATCAAGACAGTGTGGTACTGGTACCAAAACAGACATAC

AGACCAATGGAACAGAATAGAGAACCCAGAAATAAACCCAGACACCTATGGTCAATTAAT

CTTCGACAAAGGAGGCAAGAATATAAAATGGGAAAAAGACAGTCTTTTCAGCAAGTGGTG

CTGGGAAAACTGGACAGCTGCATGTAAATCAATGAAACTGGAACACACCCTCACACCATG

CACAAAAATAAACTCAAAATGGCTTAAAGACTTAAACATAAGACAAGACACCATCAAACT

CCTAGAAGAGAACATAGGCAAAACATTCTCTGACATCAACCTTACAAATGTTTTCTTAGG

TCAGTCTCCCAAGGCAACAGAAATAAAAGCAAAAATAAACCAATGGGACCTAATCAAACT

GACAAGCTTTTGCACAGCAAAGGAAACCTAAAAAAAAAAAAAGACAACCTACAGAATGGG

AGAAAATAGTTTCAAATGATGCAACTGACAAGGGCTTAATCTCTAAAATATACAAACAAC

TTATACAACTCAACAGCAAAAAACCAACAACCCAATTGAAAAATGGGCAAAAGACCTGAA

TAGACATTTCTCCAAAGAAGATATACAGATGGCCAACAAGCACATGAAAAAATGCTCAAC

ATCACTGATTATTAGAGAAATGCAAATCAAAACTACTATGAGGTACCACCTCACACCAGT

CAGAATGGCCATCATTAATAAGTCCACAAATAACAAATGCTGGAGAGGGTGTGGAGAAAA

GGGAACCCTCCTACACTGTTGGTGGGAATGTAAATTGGTACAACCACTATGGAAAACAGT

ATGGAGGTACCTCAGAAAACTAAATATAGAACTACCATATGACCCAGCAATCCCACTCTT

GGGCATATATCCAGACAAAACTTTCCTTGAAAAAGACACATGCACCCCATGTTCATTGCA

GCACTATTCACAATAGCCAAGACATGGAAACAACCTAAATGTCCATGACAGATGAATGGA

TTAAGAAGATGTGGTATATATACACAATGGAATACTACTCAGCCATAAAAAAGAACAAAA

TAATGCCATTTGCAGCAACATGGATGGAACTAGAGACTCTCATACTAAGTGAAGTAAGTC

AGAAAGAGAAAGACAAATACCATATGATATCACTTATATCTGGAATCTAATATATGGCAC

AAATGAACCTTTCCACAGAAAAGAAATCATGGACTTGGAGAACAGACTTGTGGTTGCCAA

GGGGGAGGGGGAGGGAGTGGGATGGACTGGGAATTTGGGGTTAATAGATGCAAACTATTG

CATTTGGAATGGATAAGCAATGAGATCCTGCTGTATAGCACGGGAACTATATCTAGTCAC

TTGTGATGGAGCATATGGAGGATAATGTGAGAAAAAGAATTATATATGTATGTGTGACTG

GGTCTTTGCTGTACAGTAGAAATTGACAGAACACTGTAAACCAACTATAATGGAAAAAAT

AAAAATCATAAAAAAAAAAAAAA

>L1B6#LINE/L1B

GGGGGAAGGATCAAGATGGGGAGGAGTAAGAGTCACGCTCACCTTCTCCCACAAACACAT

CAAAAAAACACATCTACATGTAAAACGATTCGCACAGAACATCTACTGAATGCTGGCAGA

AGAACTTAAACCTCCAAAAAGGGCAAGAAACTCTTGACATAACTGGGTAGAACAAAAGAA

AAAAAGAAAGAGAGAGAAAAGGAATCAGGACGGGACTAGCATTCCTGAGAGGGAGCTGTG

AAAGAGAAAAGGAACCCACATCCTGGGAAGCCACCTAACTGACGGGAGATCAGCTGAGAT

GGAGGGACCTCAAAGTCGCTGAGAAAAGCACAGCAGCTGGACTGAGGAGGGCAAAGTAGA

GTGAGAGCCGCACAGATCATCTGCACCACCACCCGGACACCACAGCCTGAGATGCTCGGG

TGGGGGCTGGGCACTGAGACTCAGGCTCTAGAGGTCAGTCCCGGGGAGAGGACTAGGGTT

GGCTGTGTGGGGACAGCCTGAGGGGCTAAGGAGGTGGCCATGGGCGGGGAGCAGTGTGCT

ATGGGCTGGGGAGTGGAACGCCATGCAGAGGGAACCCAGGAGGAGGTCTGGGCCCCAGGA

GAAGCAAGGCGCCATTGTTGGGGAGGGTGAGAGGAGGAGGGGGGACCACCATAGGAATCT

CCCTGCACACGCGTGGGCTCTCAGAGGGCGGGGTGCTCTGGCCAGGCTACGGGTGGCGAG

AAGCCACTTGCTCGGCTACGGGAGACTGGGTGCTTCTTGTGCAGGCTATGGTGGCTGGGC

ACCTCTTGTGTGGGCTAAGGGCAGCGGGGGGCTAAGTGCAACATGGTGCCTCTTGCGTGA

TCTACAGGTGGCAGGGATAGACCCGGCAGTCTCTTGGAGGCCAGAGGGAGGGTGGCCTGC

CACCACTGGGGGCCTGTGAGTGGGCTCCACCTGGGCCCCAGTCACCTCAGGGGTCGCAAA

AAAAAAAAAAGGGCACTGCAACCAAGCACCACACGTTGTTGCTCTCACTCCCCTGGGAAC

ACACCTGCCCTGCTGCTGCCACTGCCAAATACTCTGGGCAGCCCCAGATGCTTGATCACT

GTCCCTTCCCAAGACCCTACAACTAGGAGCAGCTGGTCAGCACCTCCTGCATGGGCTAAG

TGGGATGGGGTGCTTCTTGCATGGTCTACAGGTGGCGGGGCAAACCACCGCAGTTATCTC

TGACTCCAGAGGTGGGCGTGGCCCACCACCACTAGGGGTCCCTGAACAGGCACCACTTGC

AGCCCCAACACCTCAGAGGGCACCACAGAGGAGGGCATTGTGACGAACACCACCTGTTGT

TGCTCTCGCTCCCCTGGGAGCACACCGCCCTGCTGCTGCCACTGCCAAATGCTCTGGGCA

GTGCCCACATGCTTGATCACTGTCACTTCCCAGGATCCTGCAACTAGGAGCAGCCTGTGC

AGCACCTCCTGTGTGGGCTAAGTGGATGGGGTGCTTCTTGCATGGTCTACAGGTGGCAGG

GGCAAACCACCACAGTTATCACTGACTCCAGAGGTGGGCATGGCCCGCTACCACTAGGGG

TCCCTGAACAGGCACCACTTGAGCTCCAATCACCTCAGAGGAGGGCATTGCAATCAACAC

TACCTGTTGTTGCTCTCACTCCCCTGGGAACTCACACACCCTGCTGCTGCCACTGCCAAA

TGCTCTGGGCACCTTGTAAATCTGCCTAAGGCTCATTACCACTTCCCAGGGCCCTGCAAC

TAGGAGTAGCCTGGCCACCTTCCTGCAGGTCCTTGCTGCTGTTAAGAGCCCAGCAACCAG

GCACTGACTACTAGCCCTACCCATTGCCTCCTTCTCCCTGGAAACACACTCAGCACCCTG

GGGATAACAGCCTGCTCACACCGAAGAAAGAGACAGCAAATATTCAAACTCCCATGCCAA

AAATAAATAGTAACCCCCCACAAAATACAAAGGGGTGCTCTTGCATAGAAGTAGCCCTCC

AAGACTACAGTTTGGCTTCCCTAAACTCACAGAAAAAGAAAAACATAAGCAAGATGAAGA

AGCTCAGGAACCATTCCCAGTTAAAAGAACAGGAGAATTCACCTGAAGCAGCAAACAATG

AAACAGACCTCTGCAGTCTGATAGACACTGAGTTCAAAAGGGAGGTAGTGAAAATACTGA

AGGAATTAAGGCTGAATATCAAGGAATTAAGAGCAGATATGAACAGTAATGCAGATTCCT

TTAGAAAGGAACTAGAAAATATAAGGAGGAACATAGAAAAATTAGAAAATTCATTTGCAG

AGATGCAAACTGAGCTAAAGGCACTAAAGAGCAGAATGAATAATGCAGAGGAATGAATTA

GTGACTTGGAAGATAGAATAATGGAAATCACCCAATCAGGACAGCAGACAGAAAACCAAA

TGAAAAAACATGAAAGCAATATAAGAGATCTATGGGATAATATAAAGCAGGCCAATCTAC

ACATAATAGGAATTCCAGAAGGAGAAGAAAAAGAAAAGGGGATTGAAAATATATTTGAAG

AAATTATGTCTGAAAACTTTCCAAATCTAAAGGAAACTGATATCAAGATACAGGAAGCAC

AGAGGGCCCCAAACAAGTTGAACCCAAATAGGCCCACACCAAGACATATTATAATAAAAA

TGGCAAAAGTTAAAGAAAGAGAGGATTCTAAAGGCAGCAAGAGAAAAACAAAGCATTAAT

TATAAGGGAACCCCCATAAGGCTATCAGCTGATTTCTCTACAGAAACACTACAGGCCAGA

AGGGAGTGGCAAGATATATTCAAAGTTCTGAAAGGAAAAAATTTGCAGCCTAGAATACTC

TATCCAGCAAGAATATCATTTAAAATAGAAGGGGAAATAAAGAATTTCTCCAACAAACAA

AAGCTAAAAGAGTACAGCAATACTAAACCCATTCTAAAAGAAATACTGAAAGGGCTTCTC

TAAATAAAAAAAGAAGTAAGAAGAAATAGGATGGAGGAAACCACAATTGGAAAGCAATCA

CTTAAATAAGCCAGCATACAGATCTAAAAGAGAAAAAAAAAACTACTGTAAAAGGATGAT

AAACAAAGGAACAGCAAAAGGACAAACATGAAGATATTAAAAAAAGACTTCAAAATCATA

GAATGTGGGGAAGGAAAGTAAGAAAATATAGATTCTTTTTTTTTTTTTAATAATGTGTTT

GAGCCTATATGACTATCAGGCTAAAGCAAGCAGATACAGGAAGGGGTTAACATACTTAAA

AAACAGGGCAACCACAAATCAAAACCAAACATTACATTCACAAAAACTAAAAAGAAAAGT

ACTCAAGCATAAAATAAATGGAAATCATCCAACCAAAAAAAGAAAGGAAGAAAGGAGAAA

CATAGAATCAACTGGAAAACAAGGTTTAAAATGGCAATAAATACATATCTATCAATAATT

ACCTTAAATGTCAATGGACTGAATGCTCCAATCAAAAGACACAGAGTGGCAGATTGGATA

AAAAACAAAAACCTACAATCTGCTGTCTACAAGAGACTCACCTTAGGGCAAAGGACACAT

ATAGATTGAAAGTGAGGGGATGGGAAAAGATATTTCATGCCAATGGACAAGACAGGAAAG

CAGGAGTTGCAATACTCATATCAGACAAAATAGACTTTAAAATGAAGGCCATAAAGAAAG

ACAAAGAAGGACACTATTTAATGGTAAAAGGATCCATTCAAGAAGAGGATATTACAATCA

TCAATATATATGCCCCTAATATAGGAGCACCCAGATACCTACAACAAATACTAACAGACA

TAAAAGGAGAAATTGATGGGAATACAATCATAGTAGGAGACTTTAACACCCCACTCACAT

CAATGGACAGATCCTCTAGACAGAAAATCAATAAGGCAACAGAGATCCTAAAGGACACAA

TAGAAAAGTTAGACTTAATCGACATTTTCAGGACATTACATCCAAAAAAATCAGAATATA

CATTCTTCTCAAGTGCACATGGAACATTCTCAAGGATTGATCACATACTGGGGCACAAAG

CTAACCTCAACAAATTTAAGAGTATAGAAATTATTTCAAGTATCTTCTCTGACCACAATG

GCATGAAACTAGAAATCAACCACAGGAAAAGAAATGAGAAAAAACTAACTACATGGAGAC

TAAACAACATGCTACTAAAAAACCAATGGGTCAATGAGGAAATCAAGAAGGAAATTAAAA

AATACCTCGAGACAAATGATAATGAAGACACAACCACTCAAAATCTATGGGATGCCACAA

AAGCAGTGCTCAGAGGGAAATTCATAGCAATACAGGCCTTCCTCAAAAAAGAAGAAAAAT

CTCAAATTGACAACTTAACCCACACCTAAATGAATTAGAAAAAGAAGAACAAACAAAACC

TAAAGTCAGCAGAAGGAAGGAAATCATAAAGATCAGAGAGGAAATCAATAAAATAGAGAT

TCAAAAAACAATAGAAAAAAATCAATAAAACCAAGAGCTGGTTCTTTGAAAAGGTAAACA

AAATTGACAAACCTCTGGCCAGACTCACCAAGAAGAGGAGAGAAAAAACCCAAATAAACA

AAATAAGAAATGAAAAAGGAGAAATCACAACAGATACTGCAGAAATACAAAAAACCATAA

GAGAATACTATGAACAATTGTATGCCAACAAATTTGACAACCTAGAAGAAATGGACAACT

TTCTAGAGACTTACAGCCTGCCAAAACTGAATCAAGAAGAAATAGATCAACTGAACAGAC

CGATCACTAGAAATGAAATTGAATATGTCATAAAAACACTCCCTACAAATAAAAGTCCAG

GACCAGATGGCTTCACAGGCAAATTCTACCAAACATACAAAGAGGAACTTATACCCATCC

TCCTTAAACTTTTCCAAAAGGTTGAAGAAGAAGGAACACTCCCAAAGACATTCTATGATG

CCACCATCACCCTAATACCAAAACCAGACAAAGATACCACCAAAAAAGAAAACTATAGGC

CAATATCTTTGATGAATATAGATGCAAAAATTCTCAACAAAATTTTAGCCAACTGAATCC

AACAACATATAAAAAAGATCATACACCATGACCAAGTGGGATTCATCCCAGGTTCACAAG

GATGGTTCAACATATGCAAATCAATCAACGTCATACACCACATTAACAAAAGAAAAGTCA

AAAACCACATGATCATCTCAATAGATGCAGAAAAAGCATTTGACAAAGTCCAACATCCAT

TCATGATAAAAACTCTTACCAAAGTGGGTATAGAGGGAACATACCTTAACATAATAAAAG

CCATTTATGACAAACCCACAGCAAATATAATACTCAATGGAGAAAAGCTGAAAGCCTTCC

CACTAATCTGGAACAAGACAAGGATGCCCACTCTCACCACTGTTATTCAACATAGTATTG

GAAGTCCTAGCCACAGCAATCAGACAAAAAAAGAAATAAAAGGTATCCAAATTGGAAGAG

AAGAGGTAAAATTGTCACTGTATGCAGATGACATGATACTATATATAGAAAACCCTAAGG

ACTCAACCCAAAAACTACTTGAACTGATCAACAAATTCAGCAAAGTAGCAGGATATAAGA

TTAACATCAGAAATCAGTTGCATTTCTGTATACTAACAATGAAATATTAGAAAAGGAATA

CAAAAATACAATACCTTTTAAAATTGCACCCCAAAAAATCAAATACCTGGGAATACACCT

GACCAAGGAGGTGAAAGACTTATATGCTGAGAACTATAAAACATTAATCAAGGAAATTAA

AGAGGATCAAAGAAATGGAAAGATATTCCATGCTCCTGGGTTGGAAAAATTAATATTGTA

AAAATGGCCATACTACCCAAAGCAATCTACAGATTCAATGCAATCCCTATCAAATTACCC

ATGACATTTTTCACAGAACTAGAACAAACAATCCAAAAATTTATATGGAACCACAAAAGA

CCCAGAATTGCCAAAGCAATCCTGAGGAACAAAAACCAAGCAGGAGGCATAACTCTCCCA

GACTTCAGGCAATATTACAAAGCCACAGTCATCAAGACAGTGTGGTACTGGTACCAAAAC

AGACAAAGACCAATGGAACAGAATAGAGAACCCAGAAATAAACCCAGACACCTAGTCAAT

TAATCTTCGACAAAGGAGGCAAGAATATAAAATGGGAAAAAGACAGTCTTTTCAGCAAGT

GTGCTGGGAAACTGGACAGCTGCATGTAAATCAATGAAACTGGAACACACCCTCACACCA

TGCACAAAAATAAACTCAAAATGGCTTAAAGACTTAAATAAGACAAGACACCATCAAACT

CCTAGAAGAGAACATAGGCAAAACATTCTCTGACATCAACCTTACAAATGTTTTCTCAGG

TCAGTCTCCCAAGGCAACAGAAATAAAAGCAAAAATAAACCAATGGGACCTAATCAAACT

GACAAGCTTTTGCACAGCAAAGGAAACCATAAAAAAAAAACAAAAAGACAACTTACAGAA

TGGGAGAAAATAGTTTCAAATGATGCAACTGACAAGGGCTTAATCTCTAAAATATACAAA

CAACTTATACAACTCAACAGCAAAAAAGCCAACAACCCAATGGAAAAATGGGCAAAAGAC

CTGAATAGACATTTCTCCAAAGAAGATATACAGATGGCCAACAAGCACATGAAAAAATGC

TCAACATCACTGATTATTAGAGAAATGCAAATCAAAACTACTATGAGATACCACCTCACA

CCAGTCAGAATGGCCATCATTAATAAGTCCACAAATAACAAATGCTGGAGAGGGTGTGGA

GAAAAGGGAACCCTCCTGCACTGTTGGTGGGAATGTAAATTGGTACAACCACTATGGAAA

ACAGTATGGAGGTACCTTAGAAAACTATACATAGAACTACCATATGACCCAGCAATCCCA

CTCTTGGGCATATATCCAGACAAAACTTTCCTTGAAAAAGACACATGCACCCATATGTTC

ATTGCAGCACTATTCACAATAGCCAAGACATGGAAACAACCTAAATGTCCATCGACAGAT

GAATGGATTAAGAAGATGTGGTATATATACACAATGGAATACTACTCAGCCATAAAAAAG

AACAAAATAATGCCATTTGCAGCAACATGGATGGAACTAGAGACTCTCATACTGAGTGAA

GTAAGTCAGAAAGAGAAAGACAAATACCATATGATATCACTTATATCTGGAATCTAATAT

ATGGCACAAATGAACCTTTCCACAGAAAAGAAAATCATGGACTTGGAGAACAGACTTGTG

GTTGCCAAGGGGAGGGGGAGGGAGTGGGATGGACTGGGAATTTGGGGTTAATAGATGCAA

ACTATTGCCTTTGGAAATGGATAAGCAATGAGATCCTGCTGTATAGCACTGGGAACTATA

TCTAGTCACTTATGATGGAGCATGATAATGTGAGAAAAAAGAATGTATATATGTATGTGT

GACTGGGTCACCTTGCTGTACAGTAGAAAATTGACAGAACACTGTAAACCAGCTATAATG

GAAAAAATAAAAACATAATAAAAAAATAAATAAAATGAAAAAAAA

>L1B7#LINE/L1B

GGACAGATGGGAGGATAAGATCCTACCTTCTCCCACAAACACATCAAAAAAACACATCTA

CATGTAAAAACTCAACAGAACATCACTAATGCTGGCAGAAGAACTTAAACCTCCAAAAAG

GGCAAGAAACTCTTGACATAACTGTAGAACAAAAGAAAAAAAAAGAGAAAAAGAATCAGG

ATGGACTAGCATTCCTGAGAGGGAGCTGTGAAGAGAAAAGGAACACATCCTGGGAAGCCA

CCTAACTGACGAATCACAGGACCTCAAAGCCAGAAAAGAAGCAGCTGGACTGAGAGCAAA

GAGAGTGAGACCCACAGATCATCTGCACCACCGACACCACACCTAGAGCTCGGGGGCTGG

GCTGAGACTTAGGCTCCGAGGTCAGTCGGGAGAGGACTGGGTGGCGTGTGGGAAGCCTAA

GGGCTAGAGCAGTGTGCCATGGTGGGATGGTTCAGGCTGGGGAGGGAACCAGCAGAGGGA

ACCGGAGAAGGTCTGGCCTGCAGGAGAGCAAGTGCTTGTTGAGGAGAGAGGGGGACCCCA

TAGGAATCTCCCTGAACACAGGGCTCTCAGAGGGGGGGCTCTGGTCAGGCTATGGGTGCA

GAAGCCCTTGCTCAGTGGGAGACTGGGCGCTTCTTGTGCAGGCTATGGGTGGCGGCACCT

CTTGTGTGGGCTAAGGGCAGGCTAAGTGAGTGGTGCCTCTTGTGATCTACAGGTGGCAGG

GAAGACCTGGTGGTCTCTCAGAGGCCAGAGGGAGGCATGGCTGCCACCACTGGGGGCCTG

TGAGTGGGCTCCACCTGTGGCCCAGTCACCTCAGGGGTTGGCAAAAAAAAAAAAAGAGGG

CACTGCAACCAAGCACCACATTATTGCTCTCACTCCCCTGGGAACACACCCGCCCTGCAG

CTGCCACTGCCAAATGCTCTGGGCAGTGCCCAGACACTTGTCACTGTCCCTTCCCAAGAC

CCTACAACTAGGAGCAGCTGGTGCAGCACCTCCTGCATGGGCTAAGTGGGACAGGGTGCT

TCTTGCATGGTCTGCAGGTGGTGGGGGCAAACCACCACAGTTATCTCTGACTCCAGAGGT

GGGCGTGGCCCACCGCCACTAGGGATCCCTGAACAAAATCACTTGCAGCCCCAACCACCT

CAGAGGGCACCACAGAGAAGGACATTGCAATGGAACACCACCTGTTGTTGCTCTACTCCC

CTGGGAGCACACCGCCCTGCTGCTGCCACTGCCAAATGCTCTGGGCAGTGCCCAGACTTG

ATCACTGTCCCTTCCCAGGAACCTACAACTAGGAGCAGCTTGTGCAGCACCTCCTGTGGG

GGCTAAGCAGGATGAGGTGCTTCTTGCATGGTCTACAGGTGGCAGGGGCAAACACCACAG

TTATCTCTGACTCCAGAGGTGGGAGTGGCTGCCACCACTAGGGGTCTGAACAGGCACCAC

TTGCAGCCCCATCACCTCAAGGGCACCACAGAGGAGGGCATTGTGACGAACACCACCTGT

TGTGCTCTCACACCCCTGGGAGCACACCGCCCTGCTGCTGCCACTGCCAAATGCTCCAGG

CAGTGCCCACATGCTTGATCTCTGTCACTTCCCAGGATCCTGCAACTAGGAGCAGCCTGT

GCAGCACCTCCTTGTGGGCTAAGTGAGACAGGGTGCTTCATGCATGGTCTACAGGTGGAG

GGGCAAACCACCACAGTTATCACTGACTCCAGAGGTGGGTGGCCGCTACCACTAGGGGTC

CCTGAACAGGCACCACCTGCGTCCAATCACCTCAGAGGAGGGCACTGCAATAACACTACT

GTTGTTGCTCTCACTCCCCTGGGAACTCACACACCCTGCTGCTGCCACTGCCAAATGCTC

TGGGCACCTTGTAAATCTGCCTAGGGCTCATTACCACTTCCCAGGGCCCTGCAACTAGGA

GTAGCCTGTGCCACCTTCCTGCAGGTCCTTGCTGCTGTTGAGAGCCCAGCAACCAGGCAC

TGACTACTAGCCCTACCCATTGCCTCCTTCTCCCTGAAAACACACTAGCATCCTGGGGAT

AACAGCCTGCTCACACCAAAGAAAGAGACAGCAAATATTCAAACTCCCACACCAAAAATA

AATAGTAACCCCCCCAAAAATACAAAGGGGTGCTCTTGCATAGAAGTAGCCCTCCAAGAC

TACAGTTTGGCTTCCCTAAACTCACAGAAAAAGAAAAACATAAGCAAGATGAAGAAGCTC

AGAAACCATTCCCAGTTAAAGGAACAGAGAATTCACCTAAAGCAGCAAACAATGAAACAG

ACCTCTGCAGTCTGACAGACATTGAGTTCAAAAGGGAGATAGTGAAAATACTGAAGGAAT

TAAGGCTGAATATCAAGGAATTAAGAGAGGATATGAACAGTAATGCAGATTCCTTTAGAA

AGGAACTAGAAAATATAAGGAGGACAAAGAAAAATTAGAAAATTCATTTGCAGAGATGCA

AACTGAGCTAAAGGCAATAAAGAGCAGAATGAATAATGCAGAGGAACGAATTAGTGACTT

GGAAGATAGAATAATGGAAATCACCCAATCAGGACAGCAGACAGAAAACCAAATGAAAAA

ACATGAAAGCAATATAAGAGATCTATGGGATAATATAAAGTGGGCCAATCTACCATAATA

GGAATTCCAGAAGGAGAAGAAAAAGAAAAGGGGATTGAAAATATATTTGAAGAAATTATG

TCTGAAAACTTTCCAAATCTAAAGGAAACTGATATCAAGATACAGGAAGCACAGAGGGCC

CCAAACAAGTTGAACCCAAACAGGCCCACACCAAGACATATTATAATAAAAATGGCAAAA

GTTAAAGATAAGAGAGGATTCTAAAGGCAGCAAGAGAAAAGCAAAGCATTAATTATAAGG

GAACCCCCATAAGGCTATCAGCTGATTTCTCTACAGAAACACTACAGGCCAGAAGGGAGT

GGCAAGATATATTTAAAGTTCTGAAAGGAAAAAAATTGCAACCTAGAATACTCTATCCAG

CAAGAATATCATTTAAAATAGAAGGGGAAATAAAGAATTTCTCCAACAAACAAAAGCTAA

AAGAGTACAGCAATACTAAACCCATTCTAAAAGAAATACTGAAAGGGCTTCTCTAAATTA

AAAAAAAAAAAGTAAGAAGAAATAGGATGGAGGAAACCACAATTGGAAAGCAATCACTTA

AATAAGCCAGCATACAGATCTAAAAAAAATATTAAAAGTAAACAAGGAACAGCAAAGACA

AACATGAAGATGTTAAAAAAAAAACTCAAAATCATAAATGTGGGAAGGAAAGTAAGAAAA

TATAGATTTTTTTTTTTTAATAATGTGTTTGAGCCTATATGACTATCAGGCTAAAGCAAG

CAGATACAGGAAGGGGTTAACATACTTAAAAAACAGGGCAACCACAAATCAAAACCAAAC

ATTACATTCACAAAAACTGAAAAGAAAAGTACTCAAGCATAAAATAAATGGAAATCATCC

AACAAAAAAAGAAAGGAAGAAAGGAGAAACAAGAATCAACTGGAAAACAAGGTTTAAAAT

GGCAATAAATACATATCTATCAATAATCACCTTAAATGTCAATGGACTGAATGCTCCAAT

CAAAAGACACAGAGTGGCAGATTGGATAAAAAAGCAAAAACCTTCAATCTGCTGTCTACA

AGAGACTCACCTTAGGGCAAAGGACACATATAGATTGAAAGTGAGGGGATGGGAAAAGAT

ATTTCATGCCAATGGACAAGACAGGAAAGCAGGAGTTGCAATACTCATATCAGACAAAAT

AGACTTTAAAATGAAGGCCATAAAGAAAGACAAAGAAGGACACTATTTAATGGTAAAAGG

ATCCATTCAAGAAGAGGATATTACAATATCAATATATATGCCCCTAATATAGGAGCACCC

AGATACCTACAACAAATACTAACAGACATAAAAGGAGAAATTGATGGGAATACAATCATA

GTAGGAGACTTTAACACCCCACTCACATCAATGGACAGATCCTCTAGACAGAAAATCAAT

AAGGCAACAGAGATCCTAAAGGACACAATAGAAAAGTTAGACTTAATTGACATTTTCAGG

ACATTACATCCAAAAAAATCAGAATATACATTCTTCTCAAGTGCACATGGAACATTCTCA

AGGATTGATCACATACTGGGGCACAAAGCTAACCTCAACAAATTTAAGAGTATAGAAATT

ATTTCAAGTATCTTCTCTGACCACAATGGCATGAAACTAGAAATCAACCACAGGAAAAGA

AATGAGAAAAAACTAACTACATGGAGACTAAACAACATGCTACTAAAAAACCAATGGGTC

AATGAGGAAATCAAGAAGGAAATTAAAAAATACCTCGAGACAAATGATAATGAAGATACA

ACCACTCAAAATCTATGGGATGCCACAAAAGCAGTGCTCAGAGGGAAATTCATAGCAATA

CAGGCCTTCCTCAAAAAAGAAGAAAAATCTCAAATTGACAACTTAACCCACCACCTAAAT

GAATTAGAAAAAGAAACAAACAAAACCTAAAGTCAGCAGAAGGAAGGAAATCATAAAGAT

CAGAGAGGAAATCAATAAAATAGAGATTCAAAAAAAAAAAAAAATCAATAAAACCAAGAG

CTGGTTCTTTGAAAAGGTAAACAAAATTGACAAACCTCTGGCCAGACTCACCAAGAAGAG

GAGAGAAAAAACCCAAATAAACAAAATAAGAAATGAAAAAGGAGAAGTCACAACAGATAC

TACAGAAATACAAAAAAACCATAAGAGAATACTATGAACAATTGTATGCCAACAAATTTG

ACAACCTAGAAGAAATGGACAACTTTCTAGAGACTTACAGCCTGCCAAAACTGAATCAAG

AAGAAATAGATCAACTGAACAGACCAATCACTAGAAATGAAATTGAATATGTCATAAAAA

CACTCCCTACAAATAAAAGTCCAGGACCAGATGGCTTCACAGGCGAATTCTACCAAACAT

ACAAAGAGGAACTTATACCCATCCTCCTTAAACTTTTCCAAAAGGTTGAAGAAGAAGGAA

CACTCCCAAAGACATTCTATGATGCCACCATCACCCTAATTCCAAAACCAGACAAAGATA

CCACCAAAAAAGAAAACTATAGGCCAATATCTTTGATGAATATAGATGCAAAAATTCTCA

ACAAAATTTTAGCCAACCGAATCCAACAACATATAAAAAAGATCATACACCATGACCAGG

TGGGATTCATCCCAGGTTCACAAGGATGGTTCAACATATGCAAATCAATCAACGTCATAC

ACCACATTAACAAAAGAAAAGTCAAAAACCACATGATCATCTCAATAGATGCAGAAAAAG

CATTTGACAAAGTCCAACATCCATTCATGATCAAAACTCTTACCAAAGTGGGTATAGAGG

GAACATACCTTAACATAATCAAAGCCATTTATGACAAACCCACAGCAAATATAATACTCA

ATGGAGAAAAGCTGAAAGCCTTCCCACTAAAATCTGGAACAAGACAAGGATGCCCACTCT

CACCACTGTTATTCAACATAGTATTGGAAGTCCTAGCCACAGCAATCAGACAAACAAAAG

AAATAAAAGGCATCCAAATTGGAAGAGAAGAGGTAAAACTGTCACTGTATGCAGATGACA

TGATACTATATATAGAAAACCCTAAGGACTCAACCCAAAAACTACTTGAACTGATCAACA

AATTCAGCAAAGTAGCAGGATATAAGATTAACATTCAGAAATCAGTCGCATTTCTGTATA

CTAACAATGAAATATTAGAAAAGGAATACAAAAATACAATACCTTTTAAAATTGCACCCC

AAAAAAATCAAATACCTGGGAATACACCTGACCAAGGAGGTAAAAGACTTATATGCTGAG

AACTATAAAACATTAATCAAGGAAATTAAAGAAGATGTAAAGAAATGGAAAGATATTCCA

TGCTCCTGGGTTGGAAAAATTAATATTGTAAAAATGGCCATACTACCCAAAGCAATCTAC

AGATTCAATGCAATCCCTATCAAATTACCCATGACATTTTTCACAGAACTAGAACAAACA

ATCCAAAAATTTATATGGAACCACAAAAGACCCAGAATTGCCAAAGCAATTCTGAGGAAC

AAAAACCAAGCAGGAGGCATAACTCTCCCAGACTTCAGGCAATATTACAAAGCCACAGTC

ATCAAGACAGTGTGGTACTGGTACCAAAACAGACAGACAGACCAATGGAACAGAATAGAG

AACCCAGAAATAAACCCAGACACCTATGGTCAATTAATCTTTGACAAAGGAGGCAAGAAC

ATAAAATGGGAAAAAGACAGTCTTTTCAGCAAGTGTGCTGGGAAACCTGGACAGCTGCAT

GTAAATCAATGAAACTAGAACACACCCTCACACCATGCACAAAAATAAACTCAAAATGGC

TTAAAGACTTAAATATAAGACAAGACACCATCAAACTCCTGGAAGAGAACATAGGCAAAA

CATTCTCTGACATCAACCTTACAAATTTTTCTCAGGTCAGTCTCCCAAAGCAACAGAAAT

AAAAGCAAAAATAAACCAATGGGACCTAATCAAACTGACAAGCTTTTGCACAGCAAAGGA

AACCATAAAAAAAACAAAAAGACAACTTACAGAATGGGAGAAAATAGTTTCAAATGATGC

AACTGACAAGGGCTTAATCTCTAAAATATACAAACAACTTATACAACTCAACAGCAAAAA

AGCCAACAACCCAATGGAAAAATGGGCAAAAGACCTGAATAGACATTTCTCCAAGGAAGA

TATACAGATGGCCAACAAGCACATGAAAAAATGCTCAACATCACTGATTATTAGAGAAAT

GCAAATCAAAACTACCATGAGATACCACCTCACACCAGTCAGAATGGCCATCATTAATAA

GTCCACAAATAACAAATGCTGGAGGGGGTGTGGAGAAAAGGGAACCCTCCTGCACTGTTG

GTGGGAATGTAAGCTGGTACAACCACTATGGAGAACAGTATGGAGGTACCTTAGAAAACT

ATACATAGAACTACCATATGACCCAGCAATCCCACTCTTGGGCATATATCCAGACAAAAC

TTTCCTTGAAAAAGACACATGCACCCGCATGTTCATTGCAGCACTATTCACAATAGCCAA

GACATGGAAACAACCCAAATGTCCATCAACAGATGATTGGATTAGGAAGATGTGGTATAT

ATACACAATGGAATACTACTCAGCCATAAAAAAGAACAAAATAATGCCATTTGCAGCAAC

ATGGATGGAACTAGAGACTCTCATACTGAGTGAAGTAAGTCAGAAAGAGAAAGACAAATA

CCATATGATATCACTTATATCTGGAATCTAATATATGGCACAAATGAACCTTTCCACAGA

AAAGAAAATCATGGACATGGAGAACAGACTTGTGGTTGCCAAGGGGGAGGGGGAGGGAGT

GGGATGGATTGGGAATTTGGGGTTAATAGATGCAAACTATTGCCTTTGGAATGGATAAGC

AATGAGATCCTGCTGTATAGCACTGGGAACTATATCTAGTCACTTATGATGGAGCATGAT

AATGTGAGAAAAAAGAATGTATACATGTATGTGTAACTGGGTCACCTTGCTGTACAGTAG

AAAATTGACAGAACACTGTAAACCAGCTATAATGGAAAAAATAAAAATCATTATAAAAAA

AAAAAAAA

>L1B8#LINE/L1B

GGGGGCAAGATGGAGAAGAGTAAGAGGAGCTCACCCTCTCCCACAAACACATCAAAAAAA

CACATCTACATGTAAAAGATTCACACAGAACATCAACTGAATGCTGGCAGAAGAACTTAA

ACCTCCAAAAAGGGCAAGAAACTCTTGACATAACTGGGTAGAACAAAAGAAAAAAGAGAG

AGAGAAAAGGAATCAGGATGGGACTAGCACTCCCTGAGGGAGCTGTGAAGGAGAAAGGGA

ACCCACACCCTGGGAAGCCACCTAACCAACGGAAAGATCAGCCGAGTCGGAGGGAACTCC

AAGAAGCCAAGAAAAGCACAGCAGCAGGTCTGAGAACCAAAAAGCAGAGTGAGAGATGCA

CAGACCATCTGAACCACTGGCACAGACACCACAGCCTGAGATGCTCGGTGGGGGCTGGGC

ACTGAGACTTAGGCTCCAGAGGTCAGTCCCAGGGAGCGGCTGGGGTTGGGTGTGGAGACA

GCCTAAGGGACTAGGGAGCAGTGCCTGCGGGGGGGAGTGGTACGCTAAGGGCTGGGGAGT

GGAAAGCCACGGCAGAGGGAACCGGGAGAAGGTCCGACCCCAGGAGAGGCAAGGCACCAC

TGTTGGGAAGGGGAGAGGAGGAGGGGGGCCCATAGAAAACTCCTTGGGCCCCAGTGTGCA

TCTTGCCCACTGGCTTGCAGAGAGCTGAGCTTCCTGGGCCTGCCCCCCGCGCCCACCCCA

GCCCGCCCTGCTCCGCACCCCCCGCCCTGGACTCCTGCCGACCCACCACTCCGGCACCCC

CCCACCCCAGGAGCCGCCCCACAGCCCCCCTGGACTGTGCCAAACTACAGTTTGGCTTCC

CCAAATTCACAGAAAAAGAAAAACACAAGCACAATGAAGAAGCTCAGAAACCATTCCCAG

TTAAAGCAACAGGAGAATTCACCTAAAGCAGTCAACAATGAAACAGACCTCTGCAGTCTG

ACAGACTTTGAGTTCAAAAGGGAGATAGTGAAAATACTGAAGGAATTAAGAGAGGATATG

AACAGTAATGCAGATTCCCTCAGAAAGGAACTAGAAAATATAAGGAGGAGCCAAGAAAAA

CTAGAAAATTCATTTGCAGAGATACAAACGAGCTAAAGGCAATAAAGACCAGAATGAATA

ATGCAGAGGAATGAATTAGTGACGTGGAAGATAGAATAATGGAAATCACCCAAACAGGAC

AGCAGACAGAAAACCAAATGAAAAAACATGAAAGCAATATAAGAGACCTATGGGATAATA

TAAAGTGGCCAATCTAGCATAATAGGAATTCCAGAAGGAGAAGAAAAAGAAAAGGGGATT

GAAAATATATTTGAAGAAATTATGGCTGAAAACTTTCCAAATCTAAAGGATACTGATATC

AAGATACAGGAAGCACAGAGGGCCCCAAACAAGTTGAACCCAAACAGGCCCACACCAAGA

CATATTATAATAAAAATGGAAAAGTTAAAGAAAAGAGAGGATTCTAAAGGCAGCAAGAGA

AAAGCAAAGTGTTAATTATAAGGGAACCCCAATAAGGCTATCAGCTGATTTCTCTACAGA

AACACTACAGGCCAGAAGGGAGTGGCAAGATATATTTAAAGTCTGAAAGGAAAAAATTGC

AACCTAGAATACTCTATCCAGCAAGAATATCATTTAAAATAGAAGGGGAAATAAAGAATT

TCTCCAACAAACAAAAGCTGAAAGAGTACAGCAATATGAAACCCATTCTAAAAGAAATAC

TGAAAGGGCTTCTCTAAATTAAAAAAAAAAAAAAAGAGAACTAGGATGGAGGAAACCACA

ATTGGAAAGCAGTCACTTAAATAAGCCAGCATACAGATCCAAACATGAAGATGTTAAAAA

AAAAAAAAAAAAAAGACATCAAAATCATACAATGTGGGAAGGGAAGTAAGAAAAAATAGA

TTCTTTTTTTTTTTTAATGATGTGTTTGAGCCTATATGATTATCAGGCTAAAGTAAGCAG

ATATAGGAAGGGGTTAACATACTTAAAAAACAGGGCAACCACAAATCAAAACCAAACATT

ACATTCACAAAAACTGAAAAGAAAAGTACTCAAGCATAAAATAAATGGAAACCATCCAAC

CAAAAAAAGAAAAGAAGAAAGAGAAACATAGAATCAACTGGAAAACAAGGTTTAAAATGG

CAATAAATAAATATCTATCAATAATCACCTTAAATGTCAATGGACTGAATGCTCCAATCA

AAAGACACAGAGTGGCAGATTGGATAAAAAAGCAAAAACCTTCAATCTGCTGCCTACAAG

AAACTCACCTTAGGGCAAAGGACACATATAGATTGAAAGTGAGGGGATGGGAAAAGATAT

TTCATGCCAATGGACAAGACAGGAAAGCAGGAGTAGCAATACTCATATCAGACAAAATAG

ACTTTAAAACGAAGGCCATAAAGAAAGACAAAGAAGGACACTATTTAATGGTTAAAGGAT

CCATTCAAGAAGAGGATATTACAATCTCAATATATATGCCCCTAATATAGGAGCACCCAG

ATACCTCCAACAAATACTAACAGACATAAAAGGAGAAATTGATGGGAATACAATCATAGT

AGGAGACTTTAACACCCCACTCACATCAATGGACAGATCCTCTAGACAGAAAATCAATAA

AGCAACAGAGATCCTAAAGGAAACAATAGAAAAGTTAGACTTAATCGACATTTTCAGGAC

ATTACATCCAAAAAAATCAGAATACACATTCTTCTCAAGTGCACATGGAACATTCTCAAG

AATTGATCACATATTGGGGCACAAAGCTAACCTCAACAAATTTAAGAGTATAGAAATTAT

TTCAAGTATCTTCTCTGACCACAATGGCATGAAACTAGAAATCAACCACAGGAAAAGAAA

TGAGAAAAAACCTACTACATGGAGACTAAACAACATGCTACTAAAAAACCAATGGGTCAA

TGAGGAAATCAAGAAGGAAATTAAAAAATACCTCGAGACAAATGATAATGAAGACACAAC

CACTCAAAATCTATGGGATGCCACAAAAGCAGTGCTCAGAGGGAAGTTCATAGCAATACA

GGCCTTCCTCAAAAAAGAAGAAAAATCTCAAATTGACAACTTAACCCACCACCTAAATGA

ATTAAAAAGAAGAACAAGAAAAACCTAAAGTCAGCAGAAGGAAGGAAATCATAAAGATCA

AAGAGGAAATCAATAAAATAGAGATTCAAAAAACAATAGACAAAATCAATAAACCAAGAG

CTGGTTCTTTGAAAAGGTAAACAAAATTGACAAACCTCTGGCTAGACTCACCAAGAAGAG

GAGAGAAAAAACCCAAATAAACAAAATAAGAAATGAAAAAGGAGAATCACAACAGATACT

ACAGAAATACAAAAAACCATGAGAGAATACTATCAACAATTATATGCCAACAAATTTGAC

AACCTAGAAGAAATGGACAACTTTCTAGAGACTTACAGCCTGCCAAAACTGAATCAAGAA

GAAATAGATCAACTGAACAGACCGATCACTAGAAATGAAATTGAATATGTCATAAAAACA

CTCCCTACAAATAAAAGTCCAGGACCAGATGGCTTCACAGGTGAATTCTACCAAACATTC

AAAGAGGAACTTATACCCATCCTCCTTAAACTTTTCCAAAAGGTTGAAGAAGAAGGAACA

CTCCCAAAGACATTCTATGATGCCACCATCACCCTAATTCCAAAACCAGACAAAGATACC

ACCAAAAAAGAAAACTATGGCCAATATCTTTGATGAATATAGATGCAAAAATTCTCAACA

AAATTTTAGCCAACTGAATCCAACAACATATCAAAAAGATCATACACCACGACCAGGTGG

GATTCATCCCAGGTTCACAAGGATGGTTCAACATATGCAAATCAATCAATGTCATACACC

ACATTAACAAAAGAAAAGTCAAAAACCACATGATCATCTCAATAGATGCAGAAAAAGCAT

TTGACAAAGTCCAACATCCATTCATGATAAAAACTCTTACCAAAGTGGGTATAGAGGGAA

CATTCCTTAACATAATCAAAGCCATTTATGACAAACCCACAGCAAATATAATACTCAATG

GAGAAAAGCTGAAAGCCTTCCCACTAAAATCTGGAACAAGACAGGGATGCCCACTCTCAC

CACTGTTATTCAACATAGTATTGGAAGTCCTAGCCACAGCAATCAGACAAACAAAAGAAA

TAAAAGGCATCCAAATAGGAAGAGAAGAGGTAAAACTGTCACTGTATGCAGATGACATGA

TACTATATATAGAAAACCCTAAGGACTCAACCCAAAAACTACTTGAACTGATCAACAAAT

TCAGCAAAGTAGCAGGATATAAGATTAACATTCAGAAATCAGTCACATTTCTGTATACTA

ACAATGAAATATTAGAAAAGGAATACAAAAATACAATACCTTTTAAAATTGCACCCCAAA

AAATCAAATACCTGGGAATACACCTGACCAAGGAGGTAAAGGACTTATATGCGAGAACTA

TAAAACATTAATCAAGGAAATTAAAGAAGATGTAAAGAAATGGAAAGATATTCCATGCTC

CTGGGTTGGAAAAATTAATATTGTAAAAATGGCCATACTACCCAAAGCAATCTACAGATT

CAATGCAATCCCTATCAAATTACCCATGACATTTTTCACAGAACTAGAACAAACAATCCA

AAAATTTATATGGAACCACAAAAGACCCAGAATTGCCAAAGCAATCCTGAGGAACAAAAA

CCAAGCAGGAGGCATAACTCTCCCAGACTTCAGGCAATATTACAAAGCCACAGTCATCAA

GACAGTGTGGTACTGGTACCAAAACAGACAGACAGACCAATGGAACAGAATAGAGAACCC

AGAAATAAACCCAGACACCTATGGTCAATTAATCTTTGACAAAGGAGGCAAGAACATAAA

ATGGGAAAAGACAGTCTTTTCAGCAAGAATTGCTGGGAAACCTGGACAGCTGCATGCAAA

TCAATGAAACTAGAACACACCCTCACACCATGCACAAAAATAAACTCAAAATGGCTGAAA

GACTTAAATATAAGACAAGACACCATCAAACTCCTGAAGAGAACATAGGCAAAACATTCT

CTGACATCAACCTTACAAATGTTTTCTCAGGTCAGTCTCCCAAAGCAACAGAAATAAAAG

CAAAAATAAACCAATGGGACCTAATCAAACTGACAAGCTTTTGCACAGCAAAGGAAACCA

AAAAGAAAACAAAAAGACAACTTACAGAATGGGAGAAAATAGTTTCAAATGATGCAACTG

ACAAGGGCTTAATCTCTAAAATATACAAGCAACTTATACAACTCAACAGCAAAAAAGCCA

ACAACCCAATGAAAAATGGGCAAAAGACCTGAATAGACATTTCTCCAAGGAAGATATACA

GATGGCCAACAAGCACATGAAAAAATGCTCAACATCACTGATTATTAGAGAAATGCAAAT

CAAAACTACCAGAGATACCACCTCACACCAGTCAGAATGGCCATCATTAATAAGTCCACA

AATAACAAATGCTGGAGGGGGTGTGGAGAAAAGGGAACCCTCCTGCACTGTTGGTGGGAA

TGTAAGCTGGTACAACCACTATGGAGAACAGTATGGAGGTACCTTAGAAATCTATACATA

GAACTACCATATGACCCAGCAATCCCACTCTTGGGCATATATCCGGACAAAACTTTCCTT

AAAAAAGACACATGCACCCCATGTTCATTGCAGCACTATTCACAATAGCCAAGACATGGA

AACAACCCAAATGTCCATCACAGATGATTGGATTAGGAAGATGTGGTATATATACACAAT

GGAATACTACTCAGCCATAAAAAAAGAACGAAATAATGCCATTTGCAGCAACATGGATGG

AACTAGAGACTCTCATACTGAGTGAAGTAAGTCAGAAAGAGAAAGACAAATACCATATGA

TATCACTTATATCTGGAATCTAATATATGGCACAAATGAACCTTTCCACAGAAAAGAAAA

TCATGGACTTGGAGAATAGACTTGTGGTTGCCAAGGGGGAGGGGGAGGGAGTGGGATGGA

TTGGGAGCTTGGGGTTAATGGATGCAAATATTGCCTTTGGAATGGATTAGCAATGAGATC

CTGCTGTGTAGCACTGAGAACTATGTCTAGTCACTTATGATGGAGCATGATAATGTGAGA

AAAAGAATGTATACATGTATGTGTAACTGGGTCACCATGCTGTACAGTAGAAAAAAAATT

GTATTGGGGAAATAACATAAAAAAAAAAAAATTAAAATTAAAAAAAAAAATAAAATA

>L1B9#LINE/L1B

GGAGGGATCAAGATGGGGAGGAGTAAGACTGGGCTCACCTTCTCCCACAAACACATCAAA

AAAAAAAATCTACATGTAGAACGATTCACACAGAACATCTACTGAATGCTGGCAGAAGAC

CTTAAACCTCCAAAAAGGGCAAGAAACCCTCCACATAACTGGGTAGAACAAAAGGAAAAA

AAAAAGAGAGAGAAAAAGGAATCAGGACGGGACTAGCACTCCTGAGAGGGAGCTGTGAAA

GAGGAAAGGAACCCACACCCTGGGAAGCCACCTAACTGATGGGAGATCAGCCAAGATGGA

GGGACCTCAAAGCTCAGAGAAAAGCACAGCAGCTGGACTGAGGAGGGCAAAGCAGAGTGA

GAGCCCACAGACCATCGTACCACCCCCCGGACACCACAGCCTGAGATGCTCGGGCAGGGG

CTGGGCACTGAGACTCAGGCTCTGGAGGTCAGTTCCAGGGAGAGGACTAGGGTTGGCTGT

GTGGAGACAGCCTGAGGGGCTAGGGAGTGGTGTGCCAGGCTGGGGAGCGAGTGCCACAGC

CAAGGGAACCCGGGAGGAGGTCTGGGCCTGCAGGAGAAGCAAGGACCATTGTTGGGGAGG

GCAAGAGGAGGAGGGGTGGACACCATAGGAATATCTTTCCCTGCCAGCTGGACTCTCAGA

GGGTGGGGCTAGGGTGAGGCCCCTTGCTGGGCTATGGGTGACGGCCCTCTTGTGTGGCTA

TGGGTGGCGGCCGCGGGCTAAGGGAGGGACCTCTTGGTGGTCTACAGGTGTGGGGGCAAA

CCACGCAGTCATCTCAGACCCAGAGGTGGGCATGGCCCCCACCACTAGGGGTCCATGAAC

AGGCACCACCTGGGCCCCAGTCACCTCAGAGGTCAGCAAGAGGAGGGCACTGCAACCGAG

CACCACCCATTGTTGCTCTCACTCCCCTGGGAAGCACACCCCTGCTGCTGCCACTGCCAA

ATGCTCTGGGCACCACCTACACCTGCCTGATCACTGTCACTTCCCAGGGCCCTGCAACTA

GGAGCAGCCTGTGCCACCTTCCCACAGGTCCTTGCCACTGTCAAGGGCCCAGCAACCAGG

CACTGGCTACTAGCCCTGCCCATTGCCTCCATCTCCCTGGAAGCAGCACAGCACCCTAAA

AATAAATAGTAAGCCCTCACAAAATACCCAGGGGCTCACATATAAATAGCCCTCCAAGAC

TACAGTAGTTGTTTTCCCTAAACTCACAGAATAAGAAAAATATAAGCAAAATGAAGAAGC

TCAGGAACCATTCCCAGTTAAAAGAACAGGAGAATTCACCTGAAGGAGCAAACAATGAAA

CAGACCTCTGCAGTCTAACAGACACCGAGTTCAAAAAGGAGATAGTGAAAATACTGAAGG

AATTAAGAGGAATATGAAGGAATTAAGAGCGATATGAACAGTAATGCAGATTACTTTAGA

AAGGAACTAGAAAATATAAGGAGGAGCCAAGAAAAATTAGAAAATTCATTTGCAGAGATG

CAAGCTGAGTTAAAGGCACTGAAGAGCAGAATGAATAATGCAGAGGAAAATTAGTGACTT

GGAAGATAGAATAATGGAAATCACCCAATCAGGACAGCAGACAGAAAACCAAATAAAAAA

CATGAAAGCAATATAAGAGATCTATGGGATAATATAAAGTGGGCCAATCTATGCATAATA

GGGATTCCAGAAGGAGAAGAAAAAGAAAAGGGGATTGAAAATATATTTGAAGAAATTATG

GCTGAAAACTTTCCAAATCTAAAGGAAACAGATATCAAGATACAGGAAGCACAGAGGGCC

CCAAACAAGTTGAACCCAAACAGGCCCACACCAAGACATATTATAATAAAAATGGCAAAA

GTTAAAGATAAAGAGAGGATTCTAAAGGCAGCAAGAGAAAAACAAAGAGTTAATTATAAG

GGAACCCCCATAAGGCTATCAGCTGATTTCTCTACAGAAACACTACAGGCCAGAAGAGAG

TGGCAAGATATATTCAAAGTTCTAAAAGGGAAAAATTTGCAGCCTAGAATACTCTACCCA

GCAAGAATATCATTTAAAATAGAAGGAGAAATAAAGAATTTCTCCAACAAACAAAAACTA

AAAGAGTACAGCAATACTAAACCCATTCTAAAAGAAATACTGAAAGGGCTCCTCTAAATA

AAAAAGAAGTAAGAAGAAATAGGATGGAGGAAATCACAATTGGAAAGCAATCACTTAAAT

AAGCCAGTATACAGATCTAAAAGGAAAAAAAAAAATATTGTAAAAGAGATAAACACAAGG

AACAGCAAAAGGACAAACATGAAGATGTTAAAAAAGGACTCAAAATCATAAAATGTGGGG

AAGGAAAGTAAGAAAATCTAGATCTTTTTTTTTAGAATGTGTTTGAGCCTATATGACTAT

CAGGCTAAAGCAAGCAGATATAGGAAGGGGTTAACATACTTGAAAAACAGGGCAACCACA

AATCAAAACCAAACATACATTCACAAAAACTAAAAAGAAAGGACACAAGCATAAAATAAA

AGGAAATCATCCAACCAAAAAAAAAAGGAACAAAGGAGAAACATAGAATCAACTGGAAAA

CAAGGTTTAAAATGGCAATAAATACATATTTATCAATAATTACCTTAAATGTCAATGGAC

TGAATGCTCCAATCAAAAGACAAGAGTGGCAGACTGGATAAAAAAACAAGAGCCTACAAT

ATGCTGCCTACAAGAGACTCACCTTAGGGCAAAGGACACATATAAATTGAAAGTGAGGGG

ATGGAAAAAGATATTTCATGGAATGGAAAAGACAGGAAAGCAGGAGTTGCAATACTCATA

TCAGACAAAATAGACTTTAAAACGAAGGCCATAAAGAAAGACAAAAAGGACACTATTTAA

TGATAAAAGGATCCATTCAAGAAGAGGATATTACATTCAATATATATGCCCCTAATATAG

GAGCACCCAATACATACAACAAATACTAACAGACATAAAAGGAGAAATTGATGGGAATAC

AATAATAGTAGGAGACTTTAACACCCCACTCACATCAATGGACAGATCCTCTAGACAGAA

AATCAATAAGGCAACAGAGATCCTAAATGACACAATAGAAAAGTTAGACTTAATTGACAT

TTTCAGGACATTACATCCAAAAAAATCAGAATATACATTCTTTTCAAGTGCACATGGAAC

ATTCTCAAGGATTGACCACATACTGGGGCACAAAACTAACCTCAACAAATTTAAGAGTAT

AGAAATTATTTCAAGTATCTTCTCTGACCACAATGGCATGAAACTAGAAATCAACCACAG

GAAAAGAAATGAGAAAAAACTGACTACATGGAGACTAAACAACATGCTACTAAAAAACCA

ATGGGTCAATGAGGAAATCAAAAGGGAAATTAAAAAAATCTGAGACAAATGATAATGAAA

ACACAACCATTCAAAATCTATGGGATGCTACAAAAGCAGTGCTTAGAGGGAAGTTCATAG

CGATACAGGCCTTCCTCAAAAAAGAAGAAAAATCTCAAATCAACAACTTAACCACCACCT

AAAAGAATTAGAAAAAGAAACAAACAAAACCTAAAGTCAGCAGAAGGAAGGAAATCATAA

AGATCAGAGAGGAAATCAATAAAATAGAGATTCAAAAAACAATAGAAAAAAAAATCAATA

AAACCAAGAGCTGGTTCTTTGAAAGGGTAAACAAAATTGACAAACCTCTGGCCAGACTCA

CCAAGAAGAGGAGAGAAAGAACTCAAATAAACAAAATAAGAAATGAAAAAGGAGAAATCT

CAACAGATACTGCAGAAATACAAAAAAAATAAGAGAATACTATGAACAATTATATGCCAA

CAAATTTGACAACCTAGAAGAAATGGACAACTTTCTAGAGACATACAGCCCACCAAAACT

GAATCAAGAAGAAATAGATCAATGAACAGACTAATCACTAGAAATGAAATTGAATATGTA

ATAAAAACACTCCCTACAAACAAAAGTCCAGGACCAGATGGCTTCACAGGAATTCTACCA

AACATACAAAGAAGAATTATACCCATCCTTCTTAAACTTTTCCAAAAGGTTGAAGAAGAA

GGAAACTCCCAAAGACATTCTATGAAGCCACCATCACCCTAATACCAAAACCAGACAAAG

ATACTACCAAAAAAGAAAATTATAGGCCAATATCTTTGATGAATATAGATGCAAAAATTC

TCAACAAAATTTTAGCCAACGAATCCAACAACATATAAAAAAGATCATACACCAGACCAA

GTGGGATTCATCCCAAGTTCACAAGGATGGTTCAACATAGCAAATCAATCAAGTCATACA

CCACATTAACAAAAGAAAAGTCAAAAACCACATGATCATCTCAATAGATGCAGAAAAAGC

ATTTGACAAAATCCAACATCCATTCATGATAAAAACTCTTACCAAAGTGGGTATAGAGGG

AACATACCTTAACATAATAAAAGCCATTTATGACAAACCCACAGCCAATATAATACTCAA

TGGAGAAAAGCTGAAAGCCTTCCCACTAAAATCTGGAACAAGACAAGGATGCCCACTCTC

ACCACTTTTATTCAACATAGTATTGGAAGTCCTAGCCACAGCAATCAGACAAACAAAAGA

AATAAAAGGTATCCAAATTGGAAGAGAAGAGGTAAAATTGTCACTTATGCAGATGACATG

ATACTATATATAGAAAACCCTAAGGACTCCACACAAAAACTACTCAACTGATCAATGAAT

TCAGCAAAGTAGCAGGATACAAGATTAACATTCAGAAATGGTTGCATTTCTGTATACTAA

CAATGAAATATTAGAAAAGGAATATAAAAATACAATACCTTTTAAAATCACACCCCCAAA

ATTAAATACCTAGGAATAAACCTGACCAAGGAGGTGAAAGACTTATATGCTGAGAACTAT

AAAACATTAATCAAGGAAATTAAAGAGGATTCAAAGAAATGGAAAGATATTCCATGCTCC

TGGATTGGAAGAATTAATATTGTTAAAATGGCCATACTACCCAAAGCAATCTACAGATTC

AATGCAATCCCTATCAAATTACCCATGACATTTTTCACAGAACTAGAACAAACAATCCAA

AAATTTATATGGAACCATAAAAGACCCAGAATTGCCAAAGCAATCCTGAGGAACAAAAAC

CAAGCAGGAGGCATAACTCTCCCAGACTTCAGACAATATTACAAAGCTACAGTAATCAAG

ACAGTGTGGTACTGGTACAAAACAGACATACAGACCAATGGAACAGAATAGAGAACCCAG

AAATAAACCCAGACACCTATGGTCAATTAATCTTTGACAAAGGAGGCAAGAATATAAAAT

GGGAAAAAGACAGTCTCTTCAGCAAGTGGTGCTGGGAAAACTGGACAGCTGCATGTAAAT

CAATGAAACTAGAACACACCCTCACACCATGCACAAAAATAAACTCAAAATGGCTTAAAG

ACTTAAACATAAGACAAGACACCATAAAACTCCTAGAAGAGAACATAGGCAAAACATTCT

CTGACATCAACCTACAAATGTTTTCTTAGGTCAGTCTCCCAAGGCAAAGAAATAAAAACA

AAAATAAACCAATGGGACCTAATCAAACTTACAAGCTTTTGCACAGCAAAGGAAACCATA

AAAAAAAAAAAGACAACCTATGGAATGGGAGAAAATAGTTTCAAATGATGCAACTGACAA

GGGCTTAATCTCCAAAATATACAAACAACTCATACAACTCAACACAAAAAAAAAAACAAC

AACCCAATTGAAAAATGGGCAGAAGACCTGAATAGACATTTCTCCAAAGAAGAATACAGA

TGGCCAACAGGCACATGAAAAAATGCTCAACATCACTAATTATTAGAGAAATGCAAATCA

AAACTACAATGAGGTACCACCTCACACGTCAGAATGGCCATCATTAAAAGTCTACAAATA

ACAAATGCTGGAGAGGGTGTGGAGAAAAGGGAACCCTCCTACACTGTTGGTGGGAATGTA

AATTGGTACAACCACTATGGAAAACAGTATGGAGGTACCTCAGAAAACTAAATATAGAAC

TACCATATGATCCAGCAATCCCACTCCTGGGCATATATCAGACAAAACTTTCATTGAAAA

AGATACATGCACCCCTATGTTCATGCAGCACTATTCACAATAGCCAAGACATGAACAACC

TAAATGTCCATCGACAGATGAATGGATTAAGAAGATGTGGTAATATACACAATGGAATAC

TACTCAGCCATAAAAAAGAACAAAATAATGCCATTTGCAGCAACATGGATGGAACTAGAG

ACTCTCATACTAAGTGAAGTAGTCAGAAAGAGAAAGACAAATACCATATGATATCACTTA

TATCTGGAATCTAATATATGGCACAAATGAACCTATCTACAGAAAAGAAACAAACTCATG

GACTGGAGAACAGACTTGTGGTTGCCAGGGAGGGGGAGGGAGTGGGATGGACTGGGAGTT

TGGGGTTAGTAGATGCAAACTATTGCATTTGGAGTGGATAAGCAATGAGATCCTGCTGTA

TAGCACAGGGAACTATATCTATCACTTGTGATGGAACATGATGGAGGATAATGTGAGAAA

AAAATTATATATATTATTTATACTGGGTCACTTTGCTGTACAGCAGAAATTGACAGAACA

TTGTAAATCAACTATAATAAAAAAATAATAAAAAAAAAA

>L1B10#LINE/L1B

GGGTCAAGATGGCAGAGGAGTAAGAGTGGCACTCACCTTCTCCCACAAAACAAAAAAAAA

ACCATCTACATGTAGAACAATTCACAGAACATCTACTGAACACTGGCAGAAGACCTTAAA

CCTCCAAAAAGGGCAAGAAACCCTCCACATAACTGGGTAGAACAAAAGGAAAAAAAGAGA

GAGAGAAAAGAAAAAAAAGAATCAGGATGGACCAGCACTCCTGAGAGGGAGCTGTGAAAG

AGGAAAGGAATCTGCACCTGGGAGGCCACCTAACTGATGGGGAGATCAGCCAGGATAGAG

GGGGACCTCAAAGCCTCGAGAAAAGCACAGCAGCCAGACTGAGGAGGGCAAAGCAGAGAG

AGAGCCCACAGACCATCGGTACCACTCCCGGACACCACAGCCTGAGACACTCGGGGGGGC

TGGGCACTGAGACTCAGGCTTGAGGTCAGTTCCAGGGAGAGGACTAGGGTTGGCTGTGTG

GAGACAGCCTGAGGGGCTAGGGAGGGTGTCCAGCGGGAGGAGCCAAGCAGGGAGTGAGGA

GGAGGCCTGGGCCCACAGGAGAAGCAAGGTGCCATTGTTGGGGAGGAAGAGGAGGAGGGG

GGACTGCCATAGGAAATCTTTCTCTGCACACACACAGGCTCTCAGGCAGAGGGCACCTCT

TGCTGGGCTATGGGGAACGCAGCCATCTCAGACTCAGAGGTGGTGGCCCCCACCACTAGT

CCTGAACAGGCACCACCTGGGCCCCAGTACTCAGGGTGCACAGAGGAGGGCACTGCAACT

AAGGCCACCCATTGCCCTCACTCCCTGGGAATGCACATGCCCTGCTGTTGCCGCAAAGGC

TCTGGGGCCTACACCTCCCTGAGGGTCACTGCCACTTCCCAGGGCCCTGCAACCAGGAGC

AGCCTGCCACCTCCCCATGGGTCCTTGCCACTGTCAAGGGCCCAGCAACCAGGCACTGGC

TACAGGCCTGCCCATTGCCTCCATCTCCCTGGAAGCAGACAGGCTGTACACCGCACACCC

TATCAAGGGGATAACAGCCTGCACACACTGAGGAAAGAGAAGCAAGCATCCAAACCAAAA

GCAGCCCTCACCAAAAAAAAAAAAAGTAAGCCCTCACAAGCTACCCAGGGACACTCTTGC

ATATAAATAGCCCTCCAAGACTACAGTAGATATTGTTTTCCCTAAACTCACAGAATAAGA

AAAATATAAGCAAAATGAAGAAGCTCAGGAACCATTCCCAGTTAAAAGAACAGGAGAATT

CCCCTGAAGGAGCAAACAATGAAAAGACCTCTGCAGTCTAACAGACACCGAGTTCAAAAA

GGAGATATGAAAATACTGAAGGAATTAAGAGCGGATATACAGTAATGCAGATTACTTTAG

AAAGAACTAGAAATATAAGGAGGAGCCAAGAAAAATTAGAAAATTCATTTGCAGAGAACA

AGCTGAGTTAAAGGCATGAAAGCAGAATGAATAATGCAGAGGAATGAATAAGTGACTTGG

AAGATAGAATAATGGAAATCACCCAATCAGGACAGCAGACAGAAAACCAAATGAAAAAAC

ATGAAAGCAATATAAGAGATCTATGGGATAATATAAAGTGGGCCAATCTATGCATAATAG

GGATTCCAGAAGGAGAAGAAAAAGAAAAGGGGATTGAAAATATATTTGAAGAAATTATGG

CTGAAAACTTTCCAAATCTAAAGGAAACAGATATCAAGATACAGGAAGCACAGAGGGCCC

CAAACAAGTTGAACCCAAACAGACCTACACCAAGAATATTATAATAAAAATGGCAAAAGT

TAAAGATAAGGAAGGATTCTAAAGGCAGCAAGAGAAAAACAAAGAGTTAATTATAAGGGA

ACCCCCATAAGGCTATCAGCTGATTTCTCTACAGAAACACTACAGGCCAGAAGAGAGTGG

CAAGATATATTCAAAGTTCTAAAAGGGAAAAATTTGCAGCCTAGAATACTCTACCCAGCA

AGATATCATTTAAAATAGAAGGAGAAATAAAGAATTTCTCAACAAACAAAAACTAAAAGA

ATACAGCAATACTAAACCCATTCTAAAAGAAATACTGAAAGGTCTCCTCTAAATAGGAAA

GAAGTAAGAAGATATAGGATGGAGGAAATCACAATTGGAAAGTAATCACTTAAATAAGCC

AGATACAGATCAAAAAAAAAATATTTGTAAAGAAATAAACACAAGGAACAGCAAAAGGAT

AAACATGAAGATGTAAAAAAAAATCAAAATCATAAAATGTGGGGAAGGAAAGTAAGAAAA

TCTTTTTTTTTTTTTAGAATGTGTTTGACCTATATGACTATCAGCTAAAGCAAGCAGATA

TAGGAAGGGGTTAACATACTTGAAAAACAGGGCAACCACAAATCAAAACCAAACAATACA

TTCACAAAAACTAAAAAGAAGAGGACACAAGCATAAAATAAAAGGAAATCATCCAACCAA

AAAAAGAAAGGAACAAAGGAGAAACATAGAATCAACTGGAAAACAAGGTTTAAAATGGCA

ATAAATACATATTTATCAATAATTACCTTAAATGTCAATGGACTGAATGCTCCAATCAAA

AGACATAGAGTGGCAGACTGGATAAAAAAACAAGAGCCTACAATATGCTGCCTACAAGAG

ACTCACCTTAGGGCAAAGGACACATATAAATTGAAAGTGAGGGGATGGAAAAAGATATTT

CATGGAATGGAAAGACAGGAAAGCAGGAGTTGCAATACTCATATCAGACAAAATAGACTT

TAAAACAAAGGCCATAAAGAAAGATAAAGAGACACTATTTAATGATAAAAGGATCAATTC

AAGAAGAGGATATTACACTTATCAATATATATGCCCCTAATATAGGAGCACCCAAATACA

TACAACAAATACTAACAGACATAAAAGGAGAAATTGATGGGAATACAATAATAGTAGGAG

ACTTTAACACCCACTCACATCAATGGACAGATCCTCTAGACAGAAAATCAATAAGGCAAC

AGAGATCCTAAATGACACAATAGAAAGTTAGACTTAATTGATATTTTCAGGACATTACAT

CCAAAAAAACAGAATATACATTCTTTTCAAGTGCACATGGAACATTCTCAAGGATTGACC

ACATACTGGGGCACAAAACTAACCTCAACAAATTTAAGAGTATAGAAATTATTTCAAGCA

TCTTCTCTGACCACAATGGCATGAAACTAGAAATCAACCACAGGAAAAGAAATGAGAAAA

AACTGACTACATGGAGACTAAACAACATGCTACTAAAAAACCAATGGGTCAATGAGGAAA

TCAAAAAGGAAATTAAAAAATACCTTGAGACAAATGACAATGAAAACACAACCATAAAAT

CTATGGGATGCCAAAAGCAGTTCTTAGAGGGAAGTTCATAGATACAGGCTTCCTCAAAAA

AGAAGAAAAATCTCAAATCAACAACTTAACCTACCACCTAAAAGAATTAGAAAAAGAAGA

ACAAACAAAACCTAAAGTCAGCAGAAGGAAGGAAATCATAAAGATCAGAGAGGAAATCAA

TAAAATAGAATAAAAAAAAAAAAAAAATCAATAAAACCAAGAGCTGGTTCTTTGAAAGGG

TAAACAAAATTGACAAACCTCTGGCCAGACTCACAAGAAGAAGAGAGAAAGAACCCAAAT

AAACAAAATAAGAAATGAAAAAGGAGAAATCTCAATGGATACTGCAGAAATACAAAAAAA

TAAGAGAATACTATGAACAATTATATGCCAACAAATTTGACAACCTAGAAGAAATGGACA

ACTTTCTAGAACATACAGCCCACCAAAACTGAATCAAGAAGAAATAGATCATTTGAACAG

ACGATCACTAGAAATGAAATTGAATATGTAATAAAAACACTCCCTACAAACAAAAGTCCA

GGACCAGATGGCTTCACAGGGAATTCTACCAAACATACAAAGAAGAACTTATACCCATCC

TTCTTAAACTTTTCAAAAATAGAAGAAGGAACACTCCCAAAGACATTCTATGAACCACCA

TCACCCTAATACCAAAACCAGACAAAGATACTACCAAAAAAGAAAATTATAGGCCAATAT

CTTTGATGAATATAGATGAAAAATTCTCAACAAAATTTTAGCCAACCAAATCCAACAACA

CATAAAAAAGATCATACACCATGACCAAGTGGGATTCATCCCAAGTTCACAAGGATGGTT

CAACATATGCAAATCAATCAATGTCATACACCACATTAACAAAAGAAAAGTCAAAAACCA

CATGATCATCTCAATAGATGCAGAAAAAGCATTTGACAAAATCCAACATCCATTCATGAT

AAAAACTCTTACCAAAGTGGGTATAGAGGGAACATACTTAACATAATAAAAGCCATTTAT

GACAAACCCACAGCCAATATAATACTCAATGGAGAAAAGCTGAAAGCCTTCCTACTAAAA

TCTGGAACAAGACAAGGATGCCCACTCTCACCACTTTTATTCAACATAGTATTGGAAGTC

CTAGCCACAGCAATCAGACAAACAAAAGAAAAAAAGGTATCCAAATTGGAAGAGAAGAGG

TAAAATTGTCACTTATGCAGATGACATGATACTATATATAGAAAACCCTAAGGACTCCAC

ACAAAAACTACTTGAACTGATCAAAAATTCAGCAAAGTAGCAGGATACAAGATTAACATT

CAGAAATCAGTTGCATTTCTGTATACTAACAATGAAATATTAGAAAAGGAATATAAAAAA

TATTAAAATTCACCCCCAAAATTAAATACCTAGGAATAAACCTGACCAAGGAGGTGAAAG

ACTTATATGCTGAGAACTATAAAACATTAATCAAGGAAATTAAAGAGGATTCAAAGAAAT

GGAAAGATATCCATGCTCCTGGATTGGAAGAATTAATATTGTTAAAATGGCCATACTACC

CAAAGCAATCTACAGATTTAATGCGATCCCTATCAAATTACCCATGACATTTTTCACAGA

ACTAGAACAAAAATCCAAAAATTTATATGGAACCATAAAAGACCCAGAATTGCCAAAGCA

ATCCTGAGGAAAAAAACAAGCAGGAGGCATAACTCTCCCAGACTTCAGACAATATTACAA

AGCTACAGTAATCAAGACAGTGTGGTACTGGTACAAAAACAGACATACAGACCAATGGAA

CAGAATAGAGAGCCCAGAAATAAACCCAGACACCTATGGTCAATTAATCTTTAACAAAGG

AGGCAAGAATATAAAATGGGAAAAAGACAGTCTCTTCAGCAAGTGGTGCTGGGAAAACTG

GACAGCTGCATGTAAATCAATGAAACTAGAACACACCCTCACACCATGCACAAAAAAAAC

TCAAAATGGCTTAAAGACTTAAACATAAGACAAGACACCATAAAACTCCTAGAAGAGAAC

ATAGGCAAAACATTCTCTGACATCAACCTACAAATGTTTTCTTAGGTCAGTCTCCCAAGG

CAATAGAAATAAAAACAAAAATAAACCAATGGGACCTAATCAAACTTACAAGCTTTTGCA

CAGCAAAGGAAACCATAAAAAAAAAAACAAAAAGACAACCTATGGAATGGGAGAAAATAG

TTGCAAATGATGCAACGACAAGGGCTTAATCTCCAAAATATACAAACAACTCATACAACT

CAACAGCAAAAAAACAAAACCCAATTGAAAAATGGGCAGAAGACCTAAATAGACATTTCT

CCAAAGAAGACATATAGATGGCCAACAGGCACATGAAAAAATGCTCAACATCACTAATTA

TTAGAGAAATGCAAATCAAAACTACAATGAGGTACCACCTCACACAGTCAGAATGGCTAT

CATTAATAAGTCTACAAATAACAAATGCTGGAGAGGGTGTGGAGAAAAGGGAACCCTCCT

ACACTGTTGGTGGGAATGTAAATTGGTACAACCACTATGGAAAACAGTATGGAGGTTCCT

CAGAAAACTAAATATAGAACTACCATATGATCCAGCAATCCCACTCCTGGGCATATATCT

GGACAAAACTTTAATTCAAAAAGATACATGCACCCCTATGTTCATTGCAGCACTATTCAC

AATAGCCAAGACATGGAAACAACCTAAATGTCCATCAACAGATGAATGGATTAAGAAGAT

GTGGTACATATACACAATGGAATACTACTCAGCCATAAAAAAGAACAAAATAATGCCATT

TGCAGCAACATGGATGGAACTAGAGATTCTCATACTAAGTGAAGTAAGTCAGAAAGAGAA

AGACAAATACCATATGATATCACTTATATGTGGAATCTAAAATATGGCACAAATGAACCT

ATCTACAAACAGAAACAGACTCACAGACATGGAGAACAGACTTGTGGTTGCCAAGGGGAG

GGGGAGGGAGTGGGATGGACTGGGAGTTTGGGGTTAGTAGATGCAAACTATTACATTTAG

AATGGATAAGCAATGAGGTCCTGCTGTATAGCACAGGGAACTATATCCAATCACTTGTGA

TAGAACATGATGGAAGATAATATGAGAAAAAATGTATATATATTTATACTGGGTCACTTT

GCTGTACAGCAGAAATTGAAAATATTTAAAAATATAATAAATAAAAAATTAAAAAAA

>L1B11#LINE/L1B

GAGCAAGATGGTGGAGGAGTAAGAGTCTGCTCACCTTCTCCCACAAACACATCAAAAAAA

AACAATCTACATGTAAAACGACTCGCACAGAACATCAACTGAATGCTGGCAGAAGAACTT

AAACCTCCAAAAAGGGCAAGAAACTCTTGACATAACTGGGTAGAACAAAAGAAAAAAAGA

GAGAGAAAAGGAATCAGGATGGGACTAGCATTCCTGAGAGGGAGCTGTGAAGGAGAAAAG

GAACCCACATCCTGGGAAGCCACCTAACTGACGAAAGATCAGCTGAGATGGAGGGACCTC

AAAGTCACCAAGAAAAGCACAGCAGCTGGACTGAGGACGGAAAGCAGAGTGAGAGCCCAC

AGATCATCTGAACCACGGCCCAGACACCACAGCCTGAGATGCTCGGGCGGGGCTGGGGCT

GAGACTTAGGCTCTGGAGGTCGCCTATTCCCACTGTGACCACACAGAAAGCAGAAATATC

AACCAGGGGTTCTTGCATAGAATAGCCAAGCTACATTTGCTTCCCAAATAGAAAAAGAAA

AACAAAGCAAGATGAAGAAGCACAGAAACCATTCCCAGTTAAAGGAACAGGAGAATTCAC

CTAAAGCAGCAACAATGAAACAGACCTCTGCAGTCTGACAGACATTGAGTTCAAAAGGGA

GATAGTGAAAATACTGAAGGAATTAAGTAAGAAAAGAGAGGATATGAACAGTAATGCAGA

TTCCTTTAGAAAGGAACTAGAAAATATAAGGAGGAGCCAAGAAAAATTAGAAAATTCATT

TGCAGAGATGCAAACTGAGCTAAAGGCACTAAAGAGCAGAATGAATAATGCAGAGGAAGA

ATTAGTGACTTGGAAGATAGAATAATGGAAATCACCCAATCAGGACAGCAGACAGAAAAC

CAAATGAAAAAAACATGAAAGCAATATAAGAGATCTATGGGATAATATAAAGGGCCAATC

TACACATAATAGGAATTCCAGAAGGAGAAGAAAAAGAAAAGGGGATTGAAAATATATTTG

AAGAAATTATGGCTGAAAACTTTCCAAATCTAAAGGAAACTGATATCAAGATACAGGAAG

CACAGAGGGCCCCAAACAAGTTGAACCCAAACAGGCCCACACCAAGACATATTATAATAA

AAATGGCAAAAGTTAAAGATAAAGAGAGGATTCTAAAGGCAGCAAGAGAAAAGCAAAGTT

AATTATAAGGGAACCCCCATAAGGCTATCAGCTGATTTCTCTACAGAAACACTACAGGCC

AGAAGGGAGTGGCAAGATATATTTAAAGTCTGAAAGGAAAAAATTTGCAACCTAGAATAC

TCTATCCAGCAAGAATATCATTTAAAATAGAAGGGGAAATAAAGAATTTCTCCAACAAAC

AAAAGCTAAAAGAGTACAGCAATACTAAACCCATTCTAAAAGAAATACTGAAAGGGCTTC

TCTAAATTAAAAAAAAAAAAAAGAAGAGAAGAAGAATAGGATGGAGGAAACCACAATTGG

AAAGAATCACTTAAAAAAGCCAGCATACAGATAATCATGAAGATGTAAAAAAAAAAAAAA

AAAAAAAAAGACATCAAAATCATAAATGTGGGGAAGGAAAGTAAGAAAATATAGATTCTT

TTTTTTTTTAATGATGTGTTTGAGCCTATATGACTATCAGGCTAAAGCAAGCAGATATAG

GAAGGGGTTAACATACTTAAAAAACAGGGCAACCACAAATCAAAACCAAACATTACATTC

ACAAAAACTGAAAAGAAAAGTACTCAAGCATAAAATAAATGGAAATCATCCAACCAAAAA

AAGAAAGGAAGAAAGGAGAAACATAGAATCAACTGGAAAAAAGGTTTAAAATGGCAATAA

ATACATATCTATCAATAATCACCTTAAATGTCAATGGACTGAATGCTCCAATCAAAAGAC

ACAGAGTGGCAGATTGGATAAAAAAGCAAAAACCTTCAATCTGCTGCCTACAAGAAACTC

ACCTTAGAGCAAAGACACATATAGATTGAAAGTGAGGGGATGGGAAAAGATATTTCATGC

CAATGGACAAGACAGGAAAGCAGGAGTTGCAATACTCATATCAGACAAAATAGACTTTAA

AAGAAGGCCATAAAGAAAGACAAAGAAGGACACTATTTAATGGTTAAAGGATCCATTCAA

GAAGAGGATATTACAATCATCAATATATATGCCCCTAATATAGGAGCACCCAGATACCTA

CAACAAATACTAACAGACATAAAAGGAGAAATTGATGGGAATACAATCATAGTAGGAGAC

TTTAACACCCCACTCACATCAATGGACAGATCCTCTAGACAGAAAATCAATAAGGCAACA

GAGATCCTAAAGGACACAATAGAAAAGTTAGACTTAATCGACATTTTCAGGACATTACAT

CCAAAAAAATCAGAATATACATTCTTCTCAAGTGCACATGGAACATTCTCAAGAATTGAT

CACATACTGGGGCACAAAGCTAACCTCAACAAATTTAAGAGTATAGAAATTATTTCAAGT

ATCTTCTCTGACCACAATGGCATGAAACTAGAAATCAACCACAGGAAAAGAAATGAGAAA

AAACTACTACATGGAGACTAAACAACATGCTACTAAAAAACCAATGGGTCAATGAGGAAA

TCAAGAAGGAAATTAAAAAATACCTTGAGACAAATGATAATGAAGACACAACCACTCAAA

ATCTATGGGATGCCACAAAAGCAGTGCTCAGAGGGAAATTCATAGCAATAAGGCCTTCCT

CAAAAAAGAAGAAAAATCTCAAATCGACAACTTAACCCACCACCTAAATGAATTAGAAAA

AGAAGAAAAAAAAACCTAAAGTCAGCAGAAGGAAGGAAATCATAAAGATCAAAGAGGAAA

TCAATAAAATAGAGATTCAAAAAACAATAGAAAAAATCAATAAAACCAAGAGCTGGTTCT

TTGAAAAGGTAAACAAAATTGACAAACCTCTGGCCAGACTCACCAAGAAGAGGAGAGAAA

AAACCCAAATAAACAAAATAAGAAATGAAAAAGGAGAATCACAATGGATACTGCAGAAAT

ACAAAAAACACATAAGAGAATACTATGAACAATTATATGCCAACAAATTTGACAACCTGA

AGAAATGGACAACTTTCTAGAGACTTACAGCCTGCCAAAACTGAATCAAGAAGAAATAGA

TCAACTGAACAGACCATCACTAGAAATGAAATTGAATATGTCATAAAAACACTCCCTACA

AATAAAAGTCCAGGACCAGATGGCTTCACAGGTGAATTCTACCAAACATACAAAGAGGAA

TTATACCCATCCTCCTTAAACTTTTCAAAAGGTTGAAGAAGAAACACTCCCAAAGACATT

CTATGATGCCACCATCACCCTAATTCCAAAACCAGACAAAGATACCACCAAAAAAGAAAA

CTATAGGCCAATATCTTTGATGAATATAGACACAAAAATTCTCAACAAAATTTTAGCCAA

CGAATCCAACAACATATAAAAAAGATCATACACCACGACCAGGTGGGATTCATCCCAGGT

TCACAAGGATGGTTCAACATATGCAAATCAATCAATGTCATACACCACATTAACAAAAGA

AAAGTCAAAAACCACATGATCATCTCAATAGATGCAGAAAAAGCATTTGACAAAGTCCAA

CATCCATTCATGATAAAAACTCTTACCAAAGTGGGTATAGAGGGAACATACCTTAACATA

ATCAAAGCCATTTATGACAAACCCACAGCAAATATAATACTCAATGGAGAAAAGCTGAAA

GCCTTCCCACTAAAATCTGGAACAAGACAAGGATGCCCACTCTCACCACTGTTATTCAAC

ATAGTATTGGAAGTCCTAGCCACAGCAATCAGACAAACAAAAGAAATAAAAGGTATCCAA

ATTGGAAGAGAAGAGGTAAAACTGTCACTGTATGCAGATGACATGATACTATATATAGAA

AACCCTAAGGACTCAACCAAAAACTACTTGAACTGATCAACAAATTCAGCAAAGTAGCAG

GATATAAGATTAACATTCAGAAATCAGTGCATTTCTGTATACTAACAATGAAATATTAGA

AAAGGAATACAAAAATACAATACCTTTTAAAATTGCACCCCAAAAAATCAAATACCTGGG

AATACACCTGACCAAGGAGGTAAAAGACTTATATGCTGAGAACTATAAAACATTAATCAA

GGAAATTAAAGAGGATTCAAAGAAATGGAAAGATATTCCATGCTCCTGGGTTGGAAAAAT

TAATATTGTAAAAATGGCCATACTACCCAAAGCAATCTACAGATTCAATGCAATCCCTAT

CAAATTACCCATGACATTTTTCACAGAACTAGAACAAACAATCCAAAAATTTATATGGAA

CCACAAAAGACCCAGAATTGCCAAAGCAATCCTGAGGAACAAAAACCAAGCAGGAGGCAT

AACTCTCCCAGACTTCAGGCAATATTACAAAGCCACAGTCATCAAGACAGTGTGGTACTG

GTACCAAAACAGACATACAGACCAATGGAACAGAATAGAGAACCCAGAAATAAACCCAGA

CACCTATGGTCAATTAATCTTTGACAAAGGAGGCAAGAACATAAAATGGGAAAAAGAAGT

CTTTTCAGCAAGTATTGCTGGGAAACCTGGACAGCTGCATGCAAATCAATGAAACTAGAA

CACACCCTCACACCATGCACAAAAATAAACTCAAAATGGCTTAAAGACTTAAATATAAGA

CAAGACACCATCAAACTCCTGGAAGAGAACATAGGCAAAACATTCTCTGACATCAACCTT

ACAAATTTTTCTCAGGTCAGTCTCCCAAAGCAACAGAAATAAAAGCAAAAATAAACCAAT

GGGACCTAATCAAACTGACAAGCTTTTGCACAGCAAAGGAAACCAAAAAAAAACAAAAAG

ACAACTTACAGAATGGGAGAAAATAGTTTCAAATGATGCAACTGACAAGGGCTTAATCTC

TAAAATATACAAACAACTTATACAACTCAACAGCAAAAAACAACAACCCAATGGAAAAAT

GGGCAAAAGACCTGAATAGACATTTCTCCAAAGAAGATATACAGATGGCCAACAAGCACA

TGAAAAAATGCTCAACATCCCTGATTATTAGAGAAATGCAAATCAAAACTACCATGAGAT

ACCACCTCACACCAGTCAGAATGGCCATCATTAATAAGTCCACAAATAACAAATGCTGGA

GAGGGTGTGGAGAAAAGGGAACCCTCCTGCACTGTTGGTGGGAATGTAAGCTGGTACAAC

CACTATGGAGAACAGTATGGAGGTACCTTAGAAAACTATACATAGAACTACCATATGACC

CAGCAATCCCACTCTTGGGCATATATCCGGACAAAACTTTCCTTAAAAAGACACATGCAC

CCCATGTTCATTGCAGCACTATTCACAATAGCCAAGACATGGAAACAACCCAAATGTCCA

TTGACAGAGATTGGATTAGGAAGATGTGGTATATATACACAATGGAATACTACTCAGCCA

TAAAAAAGAATGAAATAATGCCATTTGCAGCAACATGGATGGAACTAGAGACTCTCATAC

TGAGTGAAGTAAGTCAGAAAGAGAAAGACAAATACCATATGATATCACTTATATCTGGAA

TCTAATATATGGCACAAATGAACCTTTCCACAGAAAAGAAAATCATGGACTTGGAGAATA

GACTTGTGGTTGCCAAGGGGAGGGGGAGGGAGTGGGATGGATTGGGAATTTGGGGTTAAT

AGATGCAAACTATTGCCTTTGGAATGGATAAGCAATGAGATCCTGCTGTGTAGCACTGGG

AACTATATCTAGTCACTTATGATGGAGCATGATAATGTGAGAAAAAAGAATGTATACATG

TATGTGTGACTGGTCACCTTGCTGTACAGTAGAAAATTGAAGAAATTGTAAATATAAAAA

AAAAAAATATAATAAAAAAAAAAAAAAAATAAAAA

>L1B12#LINE/L1B

GAAGGGATCAAGATGGTAGAGGAGTAAGACATGGTACTCACCTTCTCCCACAAACACATA

AAAAAAAAAATCTACATGTAGAACAATTCACACAGAACATCTACTGAACACTGGCAGAAG

ACCTTAAACCTCCAAAAAGGGCAAGAAACCCTCCACATAACTGGGTAGAACAAAAGGAAA

AAAAAGAGAGAGAAGAAAAAAAGGAATCAGGATGGGACCAGCACTCCTGAGAGGGAGCTG

TGAAAGAGGAAAGGAACCCCACCCTGGGAGGCCACCTAACTGAGGGAGATCAGCTAGATG

GAGGACCTCAAAGCCTCAGAGAAAAGTGCAGCAGCTGGACTGAGGAGGGCAAAGCAGAGA

GAGAGCCCACAGACCATTGGTACCACCACCCAGACACCACAGCCTGAGACACTTGGGCAG

GGGCTGGGTCTGAGACTCAGGCTCTAGAGGTCAGTTCTGGGGAGAGGACTAGGGTTGGCT

GTGTGGAGACAGCCTGAGGGGCTAGGGAGGTGTGCCATGGGCTGGGAGCGAGCCAAGCAG

GGAGCTAGGAGGAGGCCTGGGCCTACAGGAGAAGCAAGGCACCATTGTTGGGGAGGGCAA

GAGGAGAGGGGGACCACCATAGGAATATCTTTCTCTGCATCCAGGCTCTCAGGGGGGGCC

TCTTCAGCTAGGGCAGGCAAACCACCAGCATCTGACTAGAGGTGGGCTGGCACCACCACT

AGGGGTCCCTGAACAGGCACCACCTGTGGCCCCAGTCACCTCAGGGGTCCCAAGGAGGCA

CTGCAACCAGACCACCCTTGCCTCACCCCTGGAAACACATGCCCTGCTATTGCCACTGCC

AAAGGTCTGGCCTCTACACCTCCCTGAGGGTCACTGCCACTTCCAGGGCCCTGCAACCAG

GAGCAGCCTGCCACCTCCCTGGGTCCTCCACTGTCAAGGGCCAGCAACCAGGCACTAGCT

ACAGGCCCTGCCCATTGCCCCCATCTCCCTGGAAGCAGTGCAGGCTACACCGCACACCCT

ACAAGGGGATAACAGCTGCACACACTGAGGAAAGAGACAGCAAGCATCCAAACCAAAAGC

AGCCCAAACAAAAAAAAAAAGTAAGCCCTCACAAACTACCCAGGGGGCTCACATATAAAT

AGCCCTCCAAGACTACAGTAGTTGTTTTCCCTAAACTCACAGAATAAGAAAAATATAAGC

AAAATGAAGAAGCTCAGGAACCATTCCCAGTTAAAAGAACAGGAGAATTCCCTGAAGGAG

CAAACAATGAAACAGACCTCTGCAGTCTAACAGACACTGAGTTCAAAAAGGAGATAGTGA

AAATACTGAAGGAATTAAGAGCAGATATGAACAGTAATGCAGATTACTTTAGAAAGGAAC

TAGAAAATATAAGGAGGAGCCAAGAAAAATAGAAAATTCATTTGCAGAGAGCAAGCTGAG

TTAAAGGCATTGAAGAGCAGAATGAATAATGCAGAGGAATGAATAAGTGACTTGGAAGAT

AGAATAATGGAAATCACCCAATCAGGACAGCAGACAGAAAACCAAATGAAAAAAAAAATG

AAAGCAATATAAGAGATCTATGGGATAATATAAAGTGGGCCAATCTATGCATAATAGGGA

TTCCAGAAGGAGAAGAAAAAGAAAAGGGGATTGAAAATATATTTGAAGAAATTATGGCTG

AAAACTTTCCAAATCTAAAGGAAACAGATATCAAGATACAGGAAGCACAGAGGGCCCCAA

ACAAGTTGAACCCAAACAGACCTACACCAAGACATATTATAATAAAAATGGCAAAAGTTA

AGATAGGAGAGGATTCTAAAGGCAGCAAGAGAAAAACAAAGAGTTAATTATAAGGGAACC

CCCATAAGGCTATCAGCTGATTTCTCTACAGAAACACTACAGGCCAGAAGAGAGTGGCAA

GATATATTCAAAGTTCTAAAAGGGAAAAATTTGCAGCCTAGAATACTCTACCCAGCAAGA

ATATCATTTAAAATAGAAGGAGAAATAAAGAATTTCTCCAACAAACAAAAACTAAAAGAA

TACAGCAATACTAAACCCATTCTAAAAGAAATACTGAAAGTCTCCTCTAAATAGGAAAGA

AGTAAGAAGATATAGGATGGAGGAAATCACAATTGGAAAGTAATCACTTAAATAAGCCAG

TATACAGATCTAAAAAAAAAAAAACTTGTAAAAGTGATGATAAACACAAGGAACAGCAAA

AGGATAAACATGAAGATGTAAAAAAAGGACATCAAAATCATAAAATGTGGGGAAGGAAAG

TAAGAAAATCTAGTTTTTTTAGAATGTGTTTGAGCCTATATGACTATCAGGCTAAAGCAA

GCAGATATAGGAAGGGGTTAACATACTTGAAAAACAGGGCAACCACAAATCAAAACCAAA

CAATACATTCACAAAAACTAAAAAGAAGAGGACACAAGCATAAAATAAAAGGAAATCATC

CAACCAAAAAAAAAGAAAGGAACAAAGGAGAAACATAGAATCAACTGGAAAACAAGGTTT

AAAATGGCAATAAATACATATTTATCAATAATTACCTTAAATGTCAATGGACTGAATGCT

CCAATCAAAAGACATAGAGTGGCAGACTGGATAAAAAAAAAAAAACCTACAATATGCTGC

CTACAAGAGACTCACCTTAGGGCAAAGGACACATATAAATTGAAAGTGAGGGGATGGAAA

AAGATATTTCATGTGAATGGAAAAGACAGGAAAGCAGGAGTTGCAATACTCATATCAGAC

AAAATAGACTTTAAAACAAAGGCCATAAAGAAAGACAAAGAAGGACACTATTTAATGATA

AAAGGATCAATTCAAGAAGAGGATATTACACTGTCAATATATATGCCCCTAATATAGGAG

CACCCAAATACATACAACAAATACTAACAGACATAAAAGGAGAAATTGATGGGAATACAA

TAATAGTAGGAGACTTTAACACCCCACTCACATCAATGGACAGATCCTCTAGACAGAAAA

TCAATAAGGCAACAGAGATCCTAAATGACACAATAGAAAAGTTAGACTTAATTGATATTT

TCAGGACATTACATCCAAAAAAATCAGAATATACATTCTTTTCAAGTGCACATGGAACAT

TCTCAAGGATTGACCACATACTGGGGCACAAAACTAACCTCAACAAATTTAAGAGTATAG

AAATTATTTCAAGTATCTTCTCTGACCACAATGGCATGAAACTAGAAATCAACCACAGGA

AAAGAAATGAGAAAAAACTGACTACATGGAGACTAAACAACATGCTACTAAAAAAACCAA

TGGGTCAATGAGGAAATCAAAAGGAAATTAAAAAATACCTGAGACAAATGACAATGAAAA

CACAACCATTCAAAATCTATGGGATGCTGCAAAAGCAGTTCTTAGAGGGAAGTTCATAGC

AATACAGGCCTTCCTCAAAAAAGAAGAAAAATCTCAATCAACAACTTAACCTACCACCTA

AAAGAATTAGAAAAAGAAAACAAACAAAACCTAAAGTCAGCAGAAGGAAGGAAATCATAA

AGATCAGAGAGGAAATCAATAAAATAGAGATTCAAAAAAAAAAAAAAAAATCAATAAAAC

CAAGAGCTGGTTCTTTGAAAGGGTAAACAAAATTGACAAACCTCTGGCCAGACTCACCAA

GAAGAAGAGAGAAAGAACTCAAATAAAAAAATAAGAAATGAAAAAGGAGAAATCTCAATG

GATACTGCAGAAATACAAAAAAATAAGAGAATACTATGAACAATTATATGCCAACAAATT

TGACAACCTAGAAGAAATGGACAACTTTCTAGAGACATACAGCCCACCAAAACTGAATCA

AGAAGAAATAGATCATTTGAACAGACGATCACTAGAAATGAAATTGAATATGTAATAAAA

ACACTCCCTACAAACAAAAGTCCAGGACCAGATGGCTTCACAGGTGAATTCTACCAAACA

TACAAAGAAGAACTTATACCCATCCTTCTTAAACTTTTCCAAAAGATTGAAGAAGAAGGA

ACACTCCCAAAGACATTCTATGAAGCCACCATCACCCTAATACCAAAACCAGAAAAGATA

CTACCAAAAAAGAAAATTATAGGCCAATATCTTTGATGAATATAGATGCAAAAATTCTCA

ACAAAATTTTAGCCAACTGAATCCAACAACACATAAAAAAGATCATACACATGACCAAGT

GGGATTCATCCCAAGTTCACAAGGATGGTTCAACATATACAAATCAATCAATGTCATACA

CCACATTAACAAAAGAAAAGTCAAAAACCACATGATCATCTCAATAGATGCAGAAAAAGC

ATTTGACAAAATCCAACATCCATTCATGATAAAAACTCTTACCAAAGTGGGTATAGAGGG

AACATACCTTAACATAATAAAAGCCATTTATGACAAACCCACAGCCAATATAATACTCAA

TGGAGAAAAGCTGAAAGCCTTCCTACTAAAATCTGGAACAAGACAAGGATGCCCACTCTC

ACCACTTTTATTCAACATAGTATTGGAAGTCCTAGCCACAGCAATCAGACAAACAAAAGA

AATAAAAGGTATCCAAATTGGAAGAGAAGAGGTAAAACTGTCACTTATGCAGATGACATG

ATACTATATATAGAAAACCCTAAGGACTCCACACAAAAACTACTCAAACTGATCAATGAA

TTCAGCAAAGTAGCAGGATACAAGATTAACATTCAGAAATCAGTTGCATTTCTGTATACT

AACAATGAAATATTAGAAAAGGAATATAAAAAACAATACCTTTTAAAATTCACCCCCAAA

AAATTAAATACCTAGGAATAAACCTGACCAAGGAGGTGAAAGACTTATATGCTGAGAACT

ATAAAACATTAATCAAGGAAATTAAAGAGGATTCAAAGAAATGGAAAGATATTCCATGCT

CCTGGATTGGAAGAATTAATATTGTTAAAATGGCCATACTACCCAAAGCAATCTACAGAT

TCAATGCAATCCCTATCAAATTACCCATGACATTTTTCACAGAACTAGAACAAACAATCC

AAAAATTTATATGGAACCATAAAAGACCCAGAATTGCCAAAGCAATCCTGAGGAAAAAAA

CCAAGCAGGAGGCATAACTCTCCCAGACTTCAGACAATATTACAAAGCTACAGTAATCAA

GACAGTGTGGTACTGGTACAAAAACAGACATACAGACCAATGGAACAGAATAGAGAGCCC

AGAAATAAACCCAGACACCTATGGTCAATTAATCTTCAACAAAGGAGGCAAGAATATAAA

ATGGGAAAAAGACAGTCTCTTCAGCAAGTGGTGCTGGGAAAACTGGACAGCTGCATGTAA

ATCAATGAAACTAGAACACACCCTCACACCATGCACAAAAATAAACTCAAAATGGCTTAA

AGACTTAAACATAAGACAAGACACCATAAAATTCCTAGAAGAGAACATAGGCAAAACATT

CTCTGACATCAACCTACAAATGTTTTCTTAGGTCAGTCTCCCAAGGCAATAGAAATAAAA

ACAAAAATAAACCAATGGGACCTAATCAAACTTACAAGCTTTTGCACAGCAAAGGAAACC

ATAAAAAAAAAAAAGACAACCTATGGAATGGGAGAAAATAGTTGCAAATGATGCAACTGA

CAAGGGCTTAATCTCTAAAATATACAAACAACTCATACAACTCAACACAAAAAAAACAAA

CAACCCAATTGAAAAATGGGCAGAAGACCTAAATAGACATTTCTCCAAAGAAGACATACA

GATGGCCAACAGGCACATGAAAAAATGCTCAACATCACTAATTATTAGAGAAATGCAAAT

CAAAACTACAATGAGGTACCACCTCACACCAGTCAGAATGGCCATCATTAATAAGTCTAC

AAATAACAAATGCTGGAGAGGGTGTGGAGAAAAGGGAACCCTCCTACACTGTTGGTGGGA

ATGTAAATTGGTACAACCACTATGGAAAACAGTATGGAGGTACCTCAGAAAACTAAATAT

AGAACTACCATATGATCCAGCAATCCCACTCCTGGGCATATATCTAGACAAAACTTTCAT

TCAAAAAGATACATGCACCTATGTTCATTGCAGCACTATTCACAATAGCCAAGACATGGA

AACAACCTAAATGTCCATCACAGATGAATGGATTAAGAAGATGTGGTACATATACACAAT

GGAATACTACTCAGCCATAAAAAGAACAAAATAATGCCATTTGCAGCAACATGGATGGAA

CTAGAGATTCTCATACTAAGTGAAGTAAGTCAGAAAGAGAAAGACAAATACCATATGATA

TCACTTATATGTGGAATCTAAAATATGGCACAGATGAACCTATCTACAGAAAGAAACAAC

TCACGACATGGAGAACAGACTTGTGGTTGCCAAGGGGGGGAGGAGTGGGATGGACTGGGA

GTTTGGGGTTATAGATGCAAACTATTACATTTAGAATGGATAAGCAATGAGATCCTGCTG

TATAGCACAGGGAACTATATCCAATCACTTGTGATGGAACATGATGGAAGATAATATGAA

AAAAA

>L1C1#LINE/L1C

GGAGGGAGACAAGATGGCGGAAGAGTAGGGGGACACGCTCGCCCTCTCCCACAAACACAA

CAAAAAAAAACACATCTACAGGATAAATGACTCGCACAGAACAGCAACCAATCGCTGGCA

GAAGAACCTAAACTCCAATAACGGCAAGAAGTTCGTGACATTACTAGGTAAAACAAGAGA

AAAGAGGAGAGTGAGAGAAGGTGAATCTGAGCTGGACGGGCGCTCCCGAAAGGGAACTGC

GGAGGAGAAAGGGATCCTGCACCCTGGAAAGTCACCTACTCGGGGGAAAGATCAAACGAA

CCAGAGGAATCTCCAGATGCAGAGAAGAGTGTAGCAGTAAGTTGGAGTTCTGAAAAGCAG

ATCGAGAACCGAACGGACCATCTGAACTACGGGCACAGTCACCAAAAATTGAGACGCCTG

GGTGGGGGCTGGGCACCGAGACCTCGGCTCCGGAGGTTAGTCCCCGGAAAGGGCTGGGGG

GGCGGGGTGGAGCGGAGACTGCTTGGGAGGTCTAGAAACCGGTCTGTCAAGTTTGACGGG

GCAGAGACTGCCTGGGAGACTAGAAAACAAAGCTGTCACAGGGGAAGGGAGCAATACTCT

AGGGGCGGGGAAGTGGAAAGCCACATCAGAGGGAACCTGGGAGAAGAGCCTGGTCTGCGC

CCGTGCTGGGGAGGGGAGAGAAGAAGGGGTGGGTCCCCATAGAATACTCCCCATGCCACA

GCAAGCTTACCGGCCTGCTAGCTAGCAGAAAGCTGTGCTTCCCAGTGCATCCCCTCCCCC

CACCCCCACCACCCCCTACGCACTCGCCAGACCTGGGGCTGCCTGCCATCTGGGAGGGCT

GGCCTCAACAATTGCCTGAAGCCTACCACCCAGGGGCTGTCCCTGCACAGGCCTGCTTGC

CCTTTGGAGGGGCTACACCCCCGCAGAGCAGCACCAAACACCACCAGCCCCCGAGAAAAG

GCCTGCAGCCCAGAAAAGCTAGAACAAGCCTAGCCAGGCCTGAAAAGATCTGCCTAATTC

TCAGACAGTCTTTCTGAGTTGGGCTGCCCTGGGGAGGAGCCTCTTGGGTTGGCAGTGGCC

CAGCTAGCCCCCAAGCCCTCAGGGGGTGCCGAACTCTGCGGAACAGCTGCAAGGACTGCC

AGCTCCGGCAGGACCCCCTGACAGAAAAGCAGCCCAGAAAAGCTACAACAAGCCTGGCCA

GGCTGTGAAAAGATCTGCCTAATTCTCAGACGTCCTTCTGAGTTGGGCTGCCCTGGGGAA

GAGCCTCTTGGGTTCTCAGTGACCCAGATAGCTGCTCCAGCCCTCAGGGGGTGCTGCACT

CCTGAGGAACAGCTGCCCAACACCGCCAACCCCCTGCAAGAACCCCACAGCCTAAAAACA

CCAGAGCAAGCTCTGCCTGACCGAGTGAAATCTGCTACCATCGTGGTGTGGACCTCCCAG

TCCTGTCTGCCCTCAGGAAGTCCTCCTTTGCTTCAAAGAGACCCTGTTAGCCCCATCAAC

ACTCCAGAAAAGCCACACTGCCTCAAAAAAGACTGACCAACAACGCCAGCCCTCAGGAAA

CATTCCACGGCAGTGACAAGGCAAACACTGCCCGGTCACGGAGAGTACAACTCCCTCAGG

AAAAAGAAAACAACAAGCAAGATGAAGAAGCTGAGAAACCACCCCCAGTCAAACCAACAG

GAGAACTCACCTAAAACAGTCAACAATGAAACAGATCTCTGCAGTCTGACAGACCTGGAG

TTCAAAAGAGAAATAGTGAAAATACTGAAGGAATTAAGAGAAGATATGAACAGTAATGCA

GATACCCTCAGAAAGGAACTAGAAAATATAAGGAGGAGCCAAGAAAAACTAGAACATTCA

TTTGCAGAGATACAAACTGAACTAAGGGCAGTAAAAACCAGAATGAATAATGCAGAAGAA

CGAATCAGTGATATGGAAGATAGAATAATGGAAATCACCCAATCAGGTCAGCAGACAGAA

AACCAAATCAAAAAACAGGAAAGCAATATAAGAGACCTATGGGATAATATAAAGCGGGCC

AATCTACGCATAATAGGAATTCCAGAAGGAGTAGAAAAAGATAAGGGAATGGAAAATATA

TTTGAAGAAATTATCGCTGGAAACTTCCCAAATCTAAAGGATACTGGGTTCAAGATACAA

GAAGCACAGAGGGCCCCAAACAAACTGAACCCAAATAGACCCACACCAAGACACATCATA

ATAAAAATGGCAAAAGTTAGTGATAAAGAGAGGATCCTAAAGGCAGCAAGAGAAAAGCAG

AATGTTACCTACAAGGGAACCCCCATAAGATTATCAGCTGATTTCTCTACAGAAACACTA

CAGGCCAGGAGGGAATGGCAAGAGATATTTAAAGTGCTAAAAGGAAAAAATATGCAACCT

AGAATACTCTATCCAGCAAGAATATCATTTAAAATAGAAGGGGAAATAAAAATTTTTTCC

AACAAACAAAAACTAAAAGAATACAGCAACACAAAACCCAGGCTAAAAGAAATACTGAAA

GGGCTTCTCTAAACCAAAAAGAAAGGAAGGAAAGGGAAGAAAAAAAAAAAAAAAAAAAAA

AGAAGAAGAGGAAGAACTAGGACTGAGGAAACCACAATCAGAGAGCAGTCACTCAAATAA

GCCAGCATACAGATTTAATCATGAACATGTTTCAAACAAAATAAAATTAAAAAGAAAAAA

AAAGAGTCATCAAAATCATAAAATGTGGGCAAGGGATGTTAGGAAATAAATAGACCCTTT

TTTTTGTTTGTTTGTATGTTTCTCTTCTTAATTTTAATATAGTAATGAAGTGTTTGAACC

TACAGGACCATCAGGCTAAAACACACAATTATAGGAAGGGGTTAGCATACTTAAAAAACA

GGGCAACCACAAGCCAAAACCAAATATTGCATTCGCAAAAAATGAAAAAAAAAAAAAAAA

AAACACTCAAGCAGATAATAACAGGAGACCATCCAACCAAAAAAAAAAAAAAAAAAAAAA

AAAGAAAGGAAGAATGGAGAACCATAGAATCAACTGGAACACGAGGTTCAAAATGGCAAT

AAATAATCATCTATCAATTATCACCTTAAATGTCAATGGACTGAATGCCCCAATCAAAAG

ACACAGAGTGGCTGAGTGGATAAAAAGGCAAAAACCTTCAATATGCTGCCTACAAGAAAC

TCACCTTAGGACAAAGGATACATATAGATTGAAAGTGAAAGGGTGGGGAAAAATATTTCA

CGCCAATAGACATGACAGAAAAGCAGGAGTTGCAACACTCATATCAGACAAAATAGACTT

TAAAACAAAAGACATAAAGAAAGACAAAGAAGGACACTACTTAATGATTAAGGGATCCAT

CCAAGGAGAGGATGTTACTATCGTCAACATATATGCCCCAAATATAGGAGCACCCAGATA

CATACAACAAATATTAACAGACATAAAGGGAGATATTGATGGGAATACAATCATAGTAGG

AGACTTTAATACCCCCCTCACATCAATGGACAGATCCTCTAGACAGAAAACCAATAAAGC

AACAGAGATCCTAAAGGAAACAATAGAAAAGTTAGACTTAATTGATATCTTCAGGACACT

ACATCCAAAAAAATCAGAATACACATTCTTCTCAAATGCTCATGGAACATTCTCAAGAAT

CGACCACATATTGGGACACAAAGCTAACCTCAATAAATTTAGGAGCATAGAAATTATCTC

AAGTATCTTCTCTGACCACAATGCCATGAAATTAGAAATCAACCATGGGAAAAGGAAAGA

GAAAAAACCTACTACATGGAGACTAAACAACATGCTACTAAAAAACCAATGGGTCAATGA

GGAAATCAAGAAGGAAATTAAAAACTACCTTGAAACAAATGATAATGAAGACACAACCTC

TCAAAATCTATGGGATGCTGCGAAAGCAGTGCTCAGAGGGAAATTTATAGCAATACAGGC

CTTTCTCAAAAAAGAAGAAAGATCCCAAATTGACAACTTAACCCTCCACCTAAAGAATTA

GAAAAAGAAGAACAAAAAAGACCTAAAGTCAGCAGAAGGAAGGAAATTATAAAGATCAAA

GAAGAAATCAATAAAATAGAGACTCAAAAAACAATAGAGAAAATTAATAAAACCAAGAGC

TGGTTCTTTGAAAAGGTAAACAAAATTGACAAACCCCTGGCCAGACTCACTAAAAAGAGG

AGAGAAAGAACCCAAATAACCAAAATTATAAATGAAAAAGGAGAAATCACAACGGATACA

GCAGAAATACAAAAAACCATAAGAGAATACTATGAACAACTATATGGCAACAAGTTTGAC

AATCTGGAAGAAATGGACAATTTTCTAGAATCTTACAGCCTGCCAAAACTGAATCAAGAG

AAACAGACCAACTGAACAGACCATCACTAGAAATGAAATTGAAGAGTCATAAAACACTCC

CTACAAATAAAAGTCCAGGACCAGATGGCTTCACAGGGAATTCTATCAAACATATAAAGA

GGAATGGTGCCCATCCTCCTTAAACTCTTTCAAAAGGTTGAAGAAGAAGGAATACTCCCA

AAGACATTCTATGATGCCACCATCACCCTATTCCAAAACCAGACAGAGATACCACCAAAA

AAGAAAACTATCGCCCAATATCATTGATGAATATAGATGCAAAAATTCTCAACAAAATCT

TAGCCAACCGAATCCAACAACATATCAAAAAAATCATACACCATGACCAGGTTGGGTTCA

TCCCAGGTTCACAAGGATGGTTCAACATACGCAAATCAATCAACATCATACACCACATTA

ACAAAAAAAAGTCAAAAATCATATGATCATCTCAATAGAGCAGAAAAAGCATTTGACAAA

GTCCAACATCCATTCATGATCAAGACCCTCGCCAAAGTGGGTATAGAGGGAACATTCCTG

AATATAATCAAAGCCATTTATGATAAACCCACAGCAAATATAATCTCAATGGGGAAAAAC

TGAAAGCCTTCTCACTCAAATCTGGAACAAGACAGGGATGCCCACTCTCACCACTGCTCT

TCAACATAGTTTTGGAAGTCCTAGCCACAGCAATTAGACAAACAAAAGAAATAAAAGGCA

TCCATATAGGAAGAGAAGAGATAAAACTGTCACTGTATGCAGATGACATGATACTATACA

TAGAAAACCCTAAGGACTCAACCCAAAACTACTTGAACTGATTAATAATTCAGCAAAGTA

GCAGGATATAAGATTAACATTCAGAAGTCAGTCCATTTCTGTATACCAGCAATGAAATAT

TAGAAAAGGAATACAAAAATACATACCTTTTAAAATTGCACCTCACAAAATCAAATACCT

CGGAATACACCTGACCAAGAGGTAAAGGACCTATATGCCGAGAACTATAAAACTTTAATC

AAAGAAATCAAAGAAGATGTAAAGAAATGGAAAGATATTCCATGTTCCTGGATTGGAAAA

TCAATATTGTAAAAATGGCCATACTACCCAAAGCAATCTACAGATTCAATGCAATCCCTA

TCAAATTACCCATGACATTTTTCACAGAACTAGAACAAACAATCCAAACATTTATATGGA

ACAACAAAAGACCCAGAATCGCCAAAGCAATCCTGAGAAACAAAAACCAAGCAGGAGGCA

TAACTCTCCCAGACTTCAAGAAATACTACAAAGCCACAGTCATCAAAACAGTGTGGTACT

GGTATCAAAACAGACAGACAGACCAATGGAACAGAATAGAGAACCTGGAAATAAACCCTG

ACACCTATGGTCAATTAATCTTTGACAAGGGAGGCAAGAACATAAAATGGGAAAAAGAAA

GTCTATTCAGCAAGCATTGCTGGGAAACCTGGACAGCTGCATGCAAAGCAATGAAACTAG

AACACACCCTCACACCATGCACAAAAATAAACTCAAATGGCTGAAAGACTTAAATATAGA

CAGGACACCATCAAACTCCTAGAAGAAACATAGGCAAAACACTCTCTGACATCAACATCA

TGAATATTTTCTCAGGTCAGTCTCCCAAAGCAATAGAAATTAGAGCAAAAATAAACCCAT

GGGACCTCATCAAACTGAAAAGCTTTTGCACAGCAAAGGAAACCCAAAAGAAAACAAAAA

GACAACTTACAGAATGGGAGAAAATAGTTTCAAATGATGCAACGACAAGGGCTTAATCTC

TAGAATATATAAACAACTTATACAACCCAACAGCAAAAAACCAATCAATCAATGGAAAAA

TGGGCAAAAGACCTGAATAGACTTTCTCCAAAGAAGATATACAGATGGCCACAAACACAT

GAAAAAATGCTCAACATCGCTGATTATAAGAGAAATGCAAATCAAAACTACCATGAGATA

CCACCTCACACCAGTCAGAATGGCCATCATTAATAAATCCACAAATAACAAGTGCTGGAG

GGGCTGTGGAGAAAAGGGAACCCTCCTGCACTGCTGGTGGGAATGTAAACTGGTACAGCC

ACTATGGAGAACAGTTTGGAGATACCTTAGAAATCTATACATAGAACTTCCATATGACCC

CGCAATCCCACTCTTGGGCATCTATCCGGACAAAACTCTACTTAAAAGAGACACATGCAC

CCGCATGTTCATTGCAGCACTATTCACAATAGCCAGGACATGGAAACAACCCAAATGTCC

ATCACAGATGATTGGATTCGGAAGAGGTGGTATATATACACAATGGAATACTACTCAGCC

ATAAAAAAGAATGACATAATGCCATTTGCAGCAACATGGATGGAACTAGAGAATCTCATA

CTGAGTGAAATGAGCCAGAAAGACAAAGACAAATACCATATGATATCACTTATAACTGGA

ATCTAATATCCAGCACAAATGAACATCTCCTCAGAAAAGAAAATCATGGACTTGGAGAAA

GACTTGTGGCTGCCTGATGGGAGGGGGAGGGAGTGGGAGGGATCGGGAGCTTGGGCTTAT

CAGACACAACTTAGAATAGATTTACAAGGAGATCCTGCTGATAGCATTGAGAACTTGTCT

AGATACTCATGTTGCAACAGAACAAAGGGTGGGGAAAATGTAATTTAATGTATACATGTA

ATAACTGATCCCCTTGCTGTACAGTGGGAAAAAAAAAAAAAAAAA

>L1C2#LINE/L1C

GGGAGGAGACAAGATGGGGAAGAGTAGGGGGACACGCTCGCCCTCTCCCACAAACACAAC

AAAAAAAACACATCTACAGGATAAATGACTCGCACAGAACAGCAACCAATCGCTGGCAGA

AGAACCTAAACTCCAATAACGGCAAGAAGTTCGTGACATTACTAGGTAAAACAAGAGAAA

AGAGGAGAGTGAGAGAAGGTGAATCCGAGCTGGACGGGTGCTCCCGAAAGGGAACTGGGA

GGAGAAAGGGATCCCGCACCCTGGAAAGTCACCTACTCGGGGGAAAGATCAAACGAACCG

GAGGAATCTCCAGATGCAGAGAAGAGTGTAGCAGTAAGTTGGAGTTCTGAAAAGCAGATC

AAGAACCGAACGGACCATCTGAACTACGGGCACAGTCACCAAAAATTGAGACGCCTGGGT

GGGGGCTGGGCACCGAGACCTCGGCTCGGAGGTTAGTCCCCGGGAAAGGGCTGGGGGGGG

GGGGGTGGAGCGGAGACTGCTTGGGAGGTCTAGAAACCGGTCTGTCAAGTTTGACGGGGC

AGAGACTGCCTGGGAGACTAGAAAACAAAGCTGTCGCAGAGGAAGGGAGCAATACTCTAG

GGGCGGGGAAGTGGAAAGCCACATCAGAGGGAACCTGGGAGAAGAGCCTGGTCTGCGCCC

TGCTGGGGAGGGGAGAGAAGAAGGGGTGGGTCCCCATAGAATACTCCCCACGCCACAGCA

AGCTTACGGCCCACTAGCTAGCAGAAAGCTGTGCTTCCCAGTGCATCCCCTCCCCCCACC

CCCGCCACCCCCTACGCACTCGCCGGACCTGGGGCTGCCTGCCATCGGGAGGGCTGGCCT

CAACAATTGCCTGAAGCCTACCACCACAGGGGCTGTCCCTGCACAGGCCTGCTTGCCCTT

TGGAGGGGCTACACCCCCGCAGAGCAGCACCAAACACCACCAGCCCCCGAGAAAAGGCCT

GCAGCCCAGAAAAGCTAGAACAAGCCTAGCCAGGCCGTGAAAAGATCTGCCTAATTCTCG

GACAGTCTTTTCGAGTCGGCCTGCCCTGGGGAAGAGCCTCTTGGGTTTGCAGCAGCCCAG

CTAGCCGCCCAAGCCCTCAGGGGGTGCCAGCTCTGTGGATCAGCTGCATAGGACTGCCAG

CTCCAGGCAGGACCCCCTGCAGCCCAGAAAAGCTACAACAAGCCTGGCCGGCCGTGAAAA

GATCTGCCTAATTCTCGACGGTCTTTCTGAGTCAGGCTGCCCTGGGGAGGAGCCTCTGGG

TTCGCAGCGGCCCAGCTAGCCGCCCAAGCCCTCAGGGGGTGCCGAGCTCTCGTGGATCAG

CTGCATAGGACTGCCAGCTCCAGGCAGGACCCCCTGCAGCCCAGAAAAGCTACAACAAGC

CTGGCCGGGCCGTGAAAAGATCTGCCTAATTCTCGGACAGTCTTTCTGAGTCGGGCTGCC

CTGGGGAGAGCCTCTTGGGTTTTCAGCGGCCCAGCTAGCTGCTCAGCCCTCAGGGGGGCC

CACTCTCGCGGAACAGCTGCAAGGACTGCCAGCTCCCTGCAGGACCCCCTGCAGCCCAGA

AAAGCTACAACAAGCTTGGCCAGACTGTGAAAAGATCTGCCTACATTCTCAGGCCGTCCT

TCTGAGTTGGGCTGCCCTAGGGAAGAGCCTCTTAGGTTCTCAGTGACCCAGATAGCTGCT

CCAGCCCTCAGGGGGTGCTGCACTCCTGAGGAACAGCTGCCCAACACCCCAACCCCCTGC

AAGAACCCCACAGCCTAAAAACACCAGAGCAAGCTCTGCATGACCAAGTGAAATCTGCTA

CCATCGTGGTGTGGACCTCCCAGTCCTGTCTGCCCTCAGGAAGTCCTCCTTTGCTTCAAA

GAGACCCTGTTAGCCCCATCAACACTCCAGAAAAGCCACACTGCCTCAAAAAAGACTGAC

CAACAATGCCAGCCCTCAGGAAACATTCCACGGCAGTGACAAGGCAAACACTGCCTGGTC

ACGGAGAGTACAACTCCCTCAGGAAAAAGAAAACAACAAGCAAGATGAAGAAGCTGAGAA

ACCACCCCCAGTCAAACCAACAGGAGAACTCACCTAAAACAGTCAACAATGAAACAGATC

TCTGCAGTCTGACAGACCTGGAGTTCAAAAGAGAAATAGTGAAAATACTGAAGGAATTAA

GAGAAGATATGAACAGTAATGCAGATACCCTCAGAAAGGAACTAGAAAATATAAGGAGGA

GCCAAGAAAAACTAGAACATTCATTTGCAGAGATACAAACTGAACTAAGGGCAGTAAAAA

CCAGAATGAATAATGCAGAAGAACGAATCAGTGATATGGAAGATAGAATAATGGAAATCA

CCCAATCAGGTCAGCAGACAGAAAACCAAATCAAAAAACAGGAAAGCAATATAAGAGACC

TATGGGATAATATAAAGCGGGCCAATCTACGCATAATAGGAATTCCAGAAGGAGTAGAAA

AAGATAAGGGGATGGAAAATATATTTGAAGAAATTATCGCTGGAAACTTCCCAAATCTAA

AGGATACTGAGTTCAAGATACAGGAAGCACAGAGGGCCCCAAACAAATGAACCCAAATAG

ACCCACACCAAGACACATCATAATAAAAATGGCAAAAGTTAGTGATAAAGAGAGGATCCT

AAAGGCAGCAAGAGAAAAGCAGAATGTTACCTACAAGGGAACCCCCATAAGATTATCAGC

TGATTTCTCTACAGAAACACTACAGGCCAGGAGGGAATGGCAAGAGATATTTAAAGTGCT

AAAAGGAAAAAATATGCAACCTAGAATACTCTATCCAGCAAGAATATCATTTAAAATAGA

AGGGGAAATAAAAATTTTTTCCAACAAACAAAAACTAAAGAATACAGCAACACAAAACCC

AGGCTAAAAGAAATACTGAAAGGGCTTCTCTAAACCAAAAAGAAAGGAAGGAAAGGGAAG

AAAAAAGAAAAGAAAAAAAAAAGAAGAAGAGGAAGAACTAGGACTGAGGAAACCCAATCA

GAGAGCAGTCACTCAAATAAGCCAGCATACAGATTTAATCATGAACATGCTTCAAACAAA

ATAAAATTAAAAAGAAAAAAAAAAAAAGAGTCATCAAAATCATAAAATGTGGGCAAGGGA

TGTTAGGAAATAAATAACCCCTTTTTTTGTTTGTTTGTATGTTTCTCTTCTTAATTTTAA

TATAGTAATGAAGTGTTTGAACTTACAGGACCATCAGGCTAAAACACACAATTATAGGAA

GGGGTTAGCATACTTAAAAAACAGGGCAACCACAAGCCAAAACCAAATATTGCATTCGCA

AAAAATGAAAAAAAAAAAAAAAAACACTCAAGCAGATAATAACAGGAGACCATCCAACCA

AAAAAAAAAAAAAAAAAAAAAAAAGGAAGAATGGAGAACCATAGAATCAACTGGAACACG

AGGTTCAAAATGGCAATAAATAATCATCTATCAATTATCACCTTAAATGTCAATGGACTG

AATGCCCCAATCAAAAGACACAGAGTGGCTGAGTGGATAAAAAGGCAAAAACCTTCAATA

TGCTGCCTACAAGAAACTCACCTTAGGACAAAGGATACATATAGATTGAAAGTGAAAGGG

TGGGGAAAAATATTTCACGCCAATAGACATGACAGAAAAGCAGGAGTCGCAACACTCATA

TCAGACAAAATAGACTTTAAAACAAAAGACATAAAGAAAGACAAAGAAGGACACTACTTA

ATGATTAAGGGATCCATCCAAGGAGAGGATGTTACTATCGTCAACATATATGCCCCAAAT

ATAGGAGCACCCAGATACATACAACAAATATTAACAGACATAAAGGGAGATATTGATGAG

AATACAATCATAGTAGGAGACCTTAATACCCCCCTCACATCAATGGACAGATCCTCTAGA

CAGAAAACCAATAAAGCAACAGAGATCCTAAAGGAAACAATAGAAAAGTTAGACTTAATT

GATATCTTCAGGACACTACATCCAAAAAAATCAGAATACACATTCTTCTCAAATGCTCAT

GGAACATTCTCAAGAATCGACCACATATTGGGACACAAAGCTAATCTCAATAAATTTAGG

AGCATAGAAATTATCTCAAGTATCTTCTCTGACCACAATGCCATGAAATTAGAAATCAAC

CATGGGAAAAGGAAAGAGAAAAAACCTACTACATGGAGACTAAACAACATGCTACTAAAA

AACCAATGGGTCAATGAGGAAATCAAGAAGGAAATTAAAAACTACCTTGAAACAAATGAT

AATGAAGACACAACCTCTCAAAATCTATGGGATGCTGCGAAAGCAGTGCTCAGAGGGAAA

TTTATAGCAATACAGGCCTTTCTCAAAAAAGAAGAAAGATCCCAAATTGACAACTTAACC

CTCCACCTAAATGAATTAGAAAAAGAAGAACAAAAAAGACCTAAAGTCAGCAGAAGGAAG

GAAATTATAAAGATCAAAGAAGAAATCAATAAAATAGAGACTCAAAAAACAATAGAGAAA

ATTAATAAAACCAAGAGCTGGTTCTTTGAAAAGGTGAACAAAATTGACAAACCCCTGGCC

AGACTCACTAAAAAGAGGAGAGAAAGAACCCAAATAACCAAAATTATAAATGAAAAAGGA

GAAATCACAACGGATACAGCAGAAATACAAAAAACCATAAGAGAATACTATGAACAACTA

TATGGCAACAAGTTTGACAATCTGGAAGAAATGGACAATTTTCTAGAATCTTACAGCCTG

CCAAAACTGAATCAAGAAGAAACAGACCAACTGAACAGACCATCACTAGAAATGAAATTG

AAGAGGTCATAAAATCACTCCCTACAAATAAAAGTCCAGGACCAGATGGCTTCACAGGTG

AATTCTATCAAACATATAAAGAGGAATTGGTGCCCATCCTCCTTAAACTCTTTCAAAAGG

TTGAAGAAGAAGGAATACTCCCAAAGACATTCTATGATGCCACCATCACCCTCATTCCAA

AACCAGACAGAGATACCACCAAAAAAGAAAACTATCGCCCAATATCATTGATGAATATAG

ATGCAAAAATTCTCAACAAAATCTTAGCCAACCGAATCCAACAACATATCAAAAAAATTA

TACACCATGACCAGGTAGGGTTCATCCCAGGTTCACAAGGATGGTTCAACATATGCAAAT

CAATCAGCATCATACACCACATTAACAAAAAAAAAGTCAAAAATCATATGATCATCTCAA

TAGACGCAGAAAAAGCATTTGACAAAGTCCAACATCCATTCATGATCAAGACCCTCGCCA

AAGTGGGTATAGAGGGAACATTCCTGAATATAATCAAAGCCATTTATGATAAACCCACAG

CAAATATAATCCTCAATGGGGAAAAACTGAAAGCCTTCTCACTCAAATCTGGAACAAGAC

AGGGATGCCCACTCTCACCACTGCTCTTCAACATAGTTTTGGAAGTCCTAGCCACAGCAA

TTAGACAAACAAAAGAAATAAAAGGCATCCATATAGGAAGAGAAGAGATCAAACTGTCAC

TGTATGCAGATGACATGATACTATACTAGAAAACCCTAAGGACTCAACCCCAAAACTCCT

TGAACTGATTAATAAATTCAGCAAAGTAGCAGGATATAAGATTAACATTCAGAAGTCAGT

TGCATTTCTGTATACCAGCAATGAAATATTAGAAAAGGAATACAAAAATACGATACCTTT

TAAAATTGCACCTCACAAAATCAAATACCTCGGAATACACCTGACCAAGGAGGTAAAGGA

CCTATATGCCGAGAACTATAAAACTTTAATCAAAGAAATCAAAGAAGATGTAAAGAAATG

GAAAGATATTCCATGTTCCTGGATTGGAAAATCAATATTGTAAAAATGGCCATACTACCC

AAAGCAATCTACAGATTCAATGCAATCCCTATCAAATTACCCATGACATTTTTCACAGAA

CTAGAACAAACAATCCAAACATTTATATGGAACCACAAAAGACCCAGAATCGCCAAAGCA

ATCCTGAGAAACAAAAACCAAGCAGGAGGCATAACTCTCCCAGACTTCAAGAAATACTAC

AAAGCCACAGTCATCAAAACAGTGTGGTACTGGTATCAAAACAGACAGACAGACCAATGG

AACAGAATAGAGAACCCGGAAATAAACCCTGACACCTATGGTCAATTAATCTTTGACAAG

GGAGGCAAGAACATAAAATGGGAAAAAGAAAGTCTATTCAGCAAGCATTGCTGGGAAACC

TGGACAGCTGCATGCAAAGCAATGAAACTAGAACACACCCTCACACCATGCACAAAAATA

AACTCCAAATGGCTGAAAGACTTAAATATAGACAGGACACCATCAAACTCCTAGAAGAAA

ACATAGGCAAAACACTCTCTGACATCAACATCATGAATATTTTCTCAGGTCAGTCTCCCA

AAGCAATAGAAATTAGAGCAAAAATAAACCCATGGGACCTCATCAAACTGAAAAGCTTTT

GCACAGCAAAGGAAACCCAAAAGAAAACAAAAAGACAACTTACAGAATGGGAGAAAATAG

TTTCAAATGATGCAACCGACAAGGGCTTAATCTCTAGAATATATAACAACTTATACAACC

CAACAGCAAAAAAGCCAATCAATCAATGGAAAAATGGGCAAAAGACCTGAATAGACATTT

CTCCAAAGAAGATATACAGATGGCCAACAAACACATGAAAAAATGCTCAACATCGCTGAT

TATAAGAGAAATGCAAATCAAAACTACCATGAGATACCACCTCACACCAGTCAGAATGGC

CATCATTAATAAATCCACAAATAACAAGTGCTGGAGGGGCTGTGGAGAAAAGGGAACCCT

CCTGCACTGTTGGTGGGAATGTAAACTGGTACAGCCACTATGGAGAACAGTTTGGAGATA

CCTTAGAAATCTATACATAGAACTTCCATATGACCCCGCAATCCCACTCTTGGGCATCTA

TCCGGACAAAACTCTACTTAAAAGAGACACATGCACCCGCATGTTCATTGCAGCACTATT

CACAATAGCCAGGACATGGAAACAACCCAAATGTCCATCACAGATGATTGGATTCGGAAG

AGTGGTATATATACACAATGGAATACTACTCAGCCATAAAAAAGAATGACATAATGCCAT

TTGCAGCAACATGGATGGAACTAGAGAATCTCATCCTGAGTGAAATGAGCCAGAAAGACA

AAGACAAATACCATATGATATCACTTATAACTGGAATCTAATATCCAGCACAAATGAACA

TCTCCTCAGAAAAGAAAATCATGGACTTGGAGAAAAGACTTGTGGCTGCCTGATGGGAGG

GGGAGGGAGTGGGAGGGATCGGGAGCTTGGGCTTATCAGACACAACTTAGAATAGATTTA

CAAGGAGATCCTGCTGAGTAGCATTGAGAACTATGTCTAGATACTCATGTTGCAACAGAA

AAAGGGTGGGGAAAAATGTAATTGTAATGTATACATGTAAGGATAACTGACCCCTTGCTG

TACAGTGGGAAAATAAAAAAAAAAAAAAAAAA

>L1C3#LINE/L1C

GGAGGAGGCAAGATGGCGGAAGAGTAGGGGGACACGCTCGCCCTCTCCCACAAACACAAC

AACAAAAAAAACACATCTACAGGATAAACGACTCGCACAGAACAGCAACCAATCGCTGGC

AGAAGAACCTAAACTCCAATAATGGCAAGAATCTCGTGACATTACTGGGTAAAACAAGAG

AAAAGAGGAGAGTGAGAGAAGGTGAATCCGAGCTGGACGGGCGCTCCCGAAAGGGAACTG

TGGAGGAGAAAGGGATCCCACACCCTGGAAAGTCACCTACTCGAGGAAAGATCAACCGAA

CTGGAGGAATCTCCAGATGCAGAGAAGAGTGTAGCAGTAAGTCGGAGTTCTGAAAAGCAG

AGCGAGAACCGAACAGATCATCTGAACTACTGGCACAGTCACCAAAAATTGAGACGCTTG

GGTGGGGGCTGGGCACCGAGACCTCGGCTCCGGAGGTTAGTCCCCGGGAAAGGGCTGGGG

GGGGTGGAGCGGAGACTGCTTGGGAGGTCTAGGAACCAGTCTGTCAGTTTGACGGGCAGA

GACTGCCTGGGAGACTAGAAAGCAGAGTGTCCGGGGGAGGGAGCAATACGCTAAGGGCGG

GAAGTGGAAAGCCACATCAGAGGGAACCTGGGAGAAGAGCTGGGTCTGCGCCAGTGTTGG

GGAGGGGAGAGAAGAAGGGGTGGGCCCCCATAGAATACTCCCCACGCCACAGCGAGCTTA

CTGGCCCGCCAGCTATCAGAAAGCTGTGCTTCCCAGTGCATCCCCCCCCCCGCCACCCCT

TACCACTGCCGGACCTGGGGCTGCCTGCCATCCGGAGGGCTGGCCTCAACAATTGCCGGA

AGCCTACCACCACAGGGGCTGTCCCTGCCCTGGCCTGCTTGCCCTTTGGAGGGGCTACAC

CCCCGCAGAGCAGCACCAAACACCCCAGCCCCCGGGAAAAGGCCTGCAGCCCAGAAAAGC

TAGAACAAGCTTGGCCAGGCGTGAAAAGATCTGCCTACATTCTCAGACAGTCCTTCTGAG

TCGGGCTGCCCTGGGGAAGAGCCTCTTGGGTTCTCAGTGGCCCAGCTAGCTGCTCCAGCC

CTGGGGGGTGCTGCACTCCTGTGGAACAGCTGCCCAGCACCCCAGCCCCCTGCAAGAGCC

CCACAGCCCAAAAACACCAGAGCAAGCTCTGCCCGCCGAGTGAAATCTGCTCCATCGTGG

TGTGGACCTCCCAGTCCTGTCTGCCCTCAGGAAGTCCTCCTTTGCTTCAGAGAGACCCTG

TCAGCCCCACCCACCCTCCAGAAAAGCCACACTGCCTCAAAAAAGACTGACCAACAATGC

CAGCCCTCAGGAAACATTCCACGGCAGTGACAAGGCAAACACTGCCCGGCCACGGAGAGT

ACAACTCCCCAGGAAAAAGAAAACAACAAGCAAGATGAAGAAGCTGAGAAACCACCCCCA

GTCAAACCAACAGGAGAACTCACCTAAAACAGTCAACAATGAAACAGATCTCTGCAGTCT

GACAGACCTGGAGTTCAAAAGAGAAATAGTGAAAATACTGAAGGAATTAAGAGAAGATAT

GAACAGTAATGCAGATACCCTCAGAAAGGAACTAGAAAATATAAGGAGGAGCCAAGAAAA

ACTAGAACATTCATTTGCAGAGATACAAACTGAACTAAGGGCAGTAAAAACCAGAATGAA

TAATGCAGAAGAACGAATCAGTGATGTGGAAGATAGAATAATGGAAATCACCCAATCAGG

TCAGCAGACAGAAAACCAAATCAAAAAACAGGAAAGCAATATAAGAGACCTATGGGATAA

TATAAAGCGGGCCAATCTACGCATAATAGGAATTCCAGAAGGAGTAGAAAAAGATAAGGG

GATGGAAAATATATTTGAAGAAATTATCACTGGAAACTTCCCAAATCTAAAGGATACTGA

GTTCAAGATACAGGAAGCACAGAGGGCCCCAAACAAGTTGAACCCAAATAGACCCACACC

AAGACACATTATAATAAAAATGGCAAAAGTTAGTGATAAAGAGAGGATCCTAAAGGCAGC

AAGAGAAAAGCAGAATGTTACCTACAAGGGAACCCCCATAAGATTATCAGCTGATTTCTC

TACAGAAACACTACAGGCCAGGAGGGAATGGCAAGAGATATTTAAAGTGCTAAAAGGAAA

AAATATGCAACCTAGAATACTCTATCCAGCAAGAATATCATTTAAAATAGAAGGGGAAAT

AAAAATTTTTTCCAACAAACAAAACTAAAAGAATACAGCAATACAAAACCCAGGCTAAAA

GAAATACTGAAAGGGCTTCTCTAAACCAAAAAGAAAGGAAGGAAAGAAAGGAGAAAAAAA

AAAAAAAAAAAGAAGAAGAGGAAGAACTAGGATTGAGGAAACCACAATCAGAGAGCAGTC

ACTCAAATAAGCCAGCATACAGATTTAATCATGAACATGTTTCAAACAAAATAAAATTAA

AAAGAAAAAAAAGAGTCATCAAAATCATAAAATGTGGGCAAGGGATGTTAGGAAATAAAT

AGACCTTTTTTTTTGTTTGTTGTTTCTCTTCTTAATTTTAATATAGTAATGAAGTGTTTG

AACCTACAGGACCATCAGGCTAAAACACACAATTATAGGAAGGGGTTAGCATACTTAAAA

AACAGGGCAACCACAAGCCAAAACCAAACATTGCATTCGCAAAAAATGAAAAAAAAAAAA

ACACTCAAGCAGATAATAACCAGAGACCATCCAACCAAAAAAAAAAAAGGAAGAATGGAG

AACCATAGAATCAACTGGAACACGAGGTTCAAAATGGCAATAAATAATCATCTATCAATT

ATCACCTTAAATGTCAATGGACTGAATGCCCCAATCAAAAGACACAGAGTGGCTGAGTGG

ATAAAAAGGCAAAAACCTTCAATATGCTGCCTACAAGAAACTCACCTTAGGACAAAGGAT

ACATATAGATTGAAAGTGAAAGGGTGGGAAAAAATATTTCATGCCAATAGACATGACAGG

AAAGCAGGAGTTGCAACACTCATATCAGACAAAATAGACTTTAAAACAAAAGACATAAAG

AAAGACAAAGAAGGACACTATTTAATGATTAAGGGATCCATCCAAGGAGAGGATGTTACT

ATCATCAACATATATGCCCCAAATACAGGAGCACCCAGATACATACAACAAATATTAACA

GACATAAAGGGAGATATTGATGGGAATACAATCATAGTAGGAGACCTTAATACCCCCCTC

ACATCAATGGACAGATCCTCTAGACAGAAAACCAATAAAGCAACAGAGATCCTAAAGGAA

ACAATAGAAAAGTTAGACTTAATTGATATCTTCAGGACACTACATCCAAAAAAATCAGAA

TACACATTCTTCTCAAATGCACATGGAACATTCTCAAGAATCGACCACATATTGGGACAC

AAAGCTAACCTCAATAAATTTAGGAGCATAGAAATTATCTCAAGTATCTTCTCTGACCAC

AATGCCATGAAATTAGAAATCAACCATGGGAAAAGAAAGAGAAAAAACCTACTACATGGA

GACTAAACAACATGCTACTAAAAAACCAATGGGTCAATGAGGAAATCAAGAAGGAAATTA

AAAACTACCTTGAAACAAATGATAATGAAGACACAACCTCTCAAAATCTATGGGATGCTG

CAAAAGCAGTGCTCAGAGGGAAATTTATAGCAATACAGGCCTTTCTCAAAAAAGAAGAAA

GATCCCAAATTGACAACTTAACCCTCCACCTAAACGAATTAGAAAAAGAAGAACAAAAAA

GACCTAAAGTCAGCAGAAGGAAGGAAATTATAAAGATCAAAGAAGAAATCAATAAAATAG

AGATTCAAAAAACAATAGAGAAAATTAATAAAACCAAGAGCTGGTTCTTTGAAAAGGTAA

ACAAAATTGACAAACCCCTGGCCAGACTCACTAAAAAGAGGAGAGAAAGAACCCAAATAA

CCAAAATTATAAATGAAAAAGGAGAAATCACAACGGATACTGCAGAAATACAAAAAACCA

TAAGAGAATACTATGAACAACTATATGGCAACAAATTTGACAATCTGGAAGAAATGGACA

ATTTTCTAGAATCTTACAGCCTGCCAAAACTGAATCAAGAAGAAACAGACCAACTGAACA

GACCGATCACTAGAAATGAAATTGAAGAGGTCATAAAAACACTCCCTACAAATAAAAGTC

CAGGACCAGATGGCTTCACAGGTGAATTCTATCAAACATATAAAGAGGAACTGGTGCCCA

TCCTCCTTAAACTCTTTCAAAAGGTTGAAGAAGAAGGAATACTCCCAAAGACATTCTATG

ATGCCACCATCACCCTCATTCCAAAACCAGACAGAGATACCACCAAAAAAGAAAACTATC

GGCCAATATCTTTGATGAATATAGATGCAAAAATTCTCAACAAAATCTTAGCCAACCGAA

TCCAACAACATATCAAAAAAATTATACACCATGACCAGGTAGGGTTCATCCCAGGTTCAC

AAGGATGGTTCAACATACGCAAATCAATCAACATCATACACCACATTAACAAAAGAAAAG

TCAAAAATCATATGATCATCTCAATAGATGCAGAAAAAGCATTTGACAAAGTCCAACATC

CATTCATGATCAAGACCCTCGCCAAAGTGGGTATAGAGGGAACATTCCTGAACATAATCA

AAGCCATTTATGATAAACCCACAGCAAATATAATACTCAATGGGGAAAAACTGAAAGCCT

TCTCACTCAAATCTGGAACAAGACAGGGATGCCCACTCTCACCACTGCTCTTCAACATAG

TTTTGGAAGTCCTAGCCACAGCAATTAGACAAACAAAAGAAATAAAAGGCATCCATATAG

GAAGAGAAGAGATCAAACTGTCACTGTATGCAGATGACATGATACTATACATAGAAAACC

CTAAGGACTCAACCCCAAAACTACTTGAACTGATTAATAAATTCAGCAAAGTAGCAGGAT

ATAAGATTAACATTCAGAAGTCAGTTGCATTTCTGTATACCAGCAATGAAATATTAGAAA

AGGAATACAAAAATACAATACCTTTTAAAATTGCACCTCACAAAATCAAATACCTCGGAA

TACACCTGACCAAGGAGGTAAAGGACCTATATGCCGAGAACTATAAAACTTTAATCAAAG

AAATCAAAGAAGATGTAAAGAAATGGAAAGATATTCCATGTTCCTGGATTGGAAAAATCA

ATATTGTAAAAATGGCCATACTACCCAAAGCAATCTACAGATTCAATGCAATCCCTATCA

AATTACCCATGACATTTTTCACAGAACTAGAACAAACAATCCAAACATTTATATGGAACA

CAAAAGACCCAGAATCGCCAAAGCAATCCTGAGAAACAAAAACCAAGCAGGAGGCATAAC

TCTCCCAGACTTCAAGAAATACTACAAAGCCACAGTCATCAAAACAGTGTGGTACTGGTA

TCAAAACAGACAGACAGACCAATGGAACAGAATAGAGAACCCGGAAATAAACCCTGACAC

CTATGGTCAATTAATCTTTGACAAGGGAGGCAAGAACATAAAATGGGAAAAAGAAAGTCT

ATTCAGCAAGCATTGCTGGGAAACCTGGACAGCTGCATGCAAAGCAATGAAACTAGAACA

CACCCTCACACCATGCACAAAAATAAACTCAAAATGGCTGAAAGACTTAAATATACGACA

GGACACCATCAAACTCCTAGAAGAAAACATAGGCAAAACACTCTCTGACATCAACATCAT

GAATATTTTCTCAGGTCAGTCTCCCAAAGCAATAGAAATTAGAGCAAAAATAAACCCATG

GGACCTCATCAAACTGAAAAGCTTTTGCACAGCAAAGGAAACCCAAAAGAAAACAAAAAG

ACAACTTACAGAATGGGAGAAAATAGTTTCAAATGATGCAACGACAAGGGCTTAATCTCT

AGAATATATAAGCAACTTATACAACTCAACAGCAAAAAAGCCAATCAATCAATGGAAAAA

TGGGCAAAAGACCTGAATAGACATTTCTCCAAAGAAGATATACAGATGGCCAGCAAACAC

ATGAAAAAATGCTCAACATCGCTGATTATAAGAGAAATGCAAATCAAAACTACCATGAGA

TACCACCTCACACCAGTCAGAATGGCCATCATTAATAAATCCACAAATAACAAGTGCTGG

AGGGGCTGTGGAGAAAAGGGAACCCTCCTGCACTGTTGGTGGGAATGTAAACTGGTACAG

CCACTATGGAGAACAGTTTGGAGATACCTTAGAAATCTATACATAGAACTTCCATATGAC

CCGCAATCCCACTCTTGGGCATCTATCCGGACAAAACTCTACTTAAAAGAGACACATGCA

CCCGCATGTTCATTGCAGCACTATTCACAATAGCCAGGACATGGAAACAACCCAAATGTC

CATCGACAGATGATTGGATTCGGAAGAGGTGGTATATATACACAATGGAATACTACTCAG

CCATAAAAAAGAATGACATAATGCCATTTGCAGCAACATGGATGGAACTAGAGATCTCAT

ACTGAGTGAAATGAGCAGAAAGACAAAGACAAATACCATATGATATCACTTATAACTGGA

ATCTAATATCCAGCACAAATGAACATCTCCTCAGAAAAGAAAATCATGGACTTGGAGAAA

GACTTGTGGCTGCCTGATGGGAGGGGGAGGGAGTGGGAGGGATCGGGAGCTTGGGCTTAT

CAGACACAACTTAGAATAGATTTACAAGGAGATCCTGCTGAGTAGCATTGAGAACTTGTC

TAGATACTCATGTTGCAACAGAACAAAGGGTGGGGGAAAAATAATGTATACATGTAAGGA

TAACTTGATCCCCTGCTGTACAGTGGGAAAAAAAAAAAAAAAAAAAAAAAAAAAAAA

>L1C4#LINE/L1C

GGAGGCAAGATGGCGGAAGAGTAGGGGGACACGCTCGCCCTCTCCCACAAACACAACAAA

AAAAAAACACATCTACAGGTTAAACGACTCACACAGAACAGCAACCAATCGCTGGCAGAA

GAACCTAAACTCCAATAATGGCAAGAATCTCGTGACATTACTAGGTAAAACAAGAGAAAA

GAGGAGAGTGAGAGAAGGTGAATCCGAGCTGGACGGGCGCTCCCGAAAGGGAACTGTGGA

GGAGAAAGGGATCCCACACCCTGGAAAGTCACCTACTCGACGGAAAGATCAACCGAACCG

GAGGAATCTCCAGATGCAGAGAAGAGTGTAGCAGTAAGTCGGAGTTCTGAAAAGCAGAGC

GAGAACCGAACAGATCATCTGAACTACTGGCACAGTCACCAAAAATTGAGACGCTTGGGT

GGGGGCTGGGCACCGAGACCTCGGCTCCGGAGGTTAGTCCCCGGGAAAGGGCTGGGGGGG

GTGGAGCGGAGACTGCTTGGGGGGTCTAGGAACTGGTCTGTCAAGTTTGACGGGGCAGAG

ACTGCCTGGGAGACTAGAAAGCAGAGCGTCCGGGGGAGGGAGCAATACGCTAAGGGCGGG

AAGTGGAAAGCCACATCAGAGGGAACCTGGGAGAAGAGCTGGGTCTGCGCCAGTGTTGGG

GAGGGGAGAGAAGAAGGGGTGGGCCCCCATAGAATACTCCCCATGCCACAGCGAGCTCAC

TGGCCCGCCAGCTATCAGAAAGCTGTGCTTCCCAGTGCATCCCCCCCCCCCCCACCCCTT

ACGCACACGCCGACCTGGGGCTGCCTGCCATCCGGAGGGCTGGCCTCAACAATTGCCGAA

GCCTACCACCCAGGGGCTGTCCCTGCCCTGGCCTGCTTGCCCTTTGGAGGGGCTACACCC

CCGCGGAGCAGCACCAAACACCGCCAGCCCCCGGAAAAGGCCTGCAGCCCAGAAAAGCTA

GAACAAGCTTGGCCGGGCCTGAAAAGATCTGCCTACATTCTCAGACAGTCCTTCCGAGTC

GGCTGCCCTGGGGAAGAGCCTCTTGGGTTCTCAGTGGCCCAGCTAGCTGCTCCAGCCCTT

GGGGGGTGCTGCACTCCTGGGAACAGCTGCCCAGCACCGCCAGCCCCCTGCAAGAGCCCC

ACAGCCCAAAAACACCAGAGCAAGCTCTGCCCGGCCGAGTGAAATCTGCTCCATCGTGGT

GTGGACCTCCCAGTCCTGTCTGCCCTCAGGAAGTCCTCCTTTGCTTCAGAGAGACCCTGT

CAGCCCCACCCACCCTCCAGAAAAGCCACACTGCCTCAAAGAAGACTGACCAACAATGCC

AGCCCTCAGGAAACATTCCACGCAGTGACAAGGCAAACACTGCCCGGCCACGGAGAGTAC

AACTCCACCAGGAAAAAGAAAACAACAAGCAAGATGAAGAAGCTGAGAAACCACCCCCAG

TTAAACCAACAGGAGAACTCACCTAAAGCAGTCAACAATGAAACAGATCTCTGCAGTCTG

ACAGACCTGGAGTTCAAAAGAGAAATAGTGAAAATACTGAAGGAATTAAGAGAAGATATG

AACAGTAATGCAGACACCCTCAGAAAGGAACTAGAAAATATAAGGAGGAGCCAAGAAAAA

CTAGAAAATTCATTTGCAGAGATACAAACTGAACTAAGGGCAGTAAAAACCAGAATGAAT

AATGCAGAAGAACGAATCAGTGATGTGGAAGATAGAATAATGGAAATCACCCAATCAGGT

CAGCAGACAGAAAACCAAATGAAAAAACATGAAAGCAATATAAGAGACCTATGGGATAAT

ATAAAGCGGGCCAATCTACGCATAATAGGAATTCCAGAAGGAGTAGAAAAAGATAAGGGG

ATGGAAAATATATTTGAAGAAATTATCGCTGGAAACTTCCCAAATCTAAAGGATACTGAG

TTCAAGATACAGGAAGCACAGAGGGCCCCAAACAAGTTGAACCCAAATAGACCCACACCA

AGACACATTATAATAAAAATGGCAAAAGTTAGTGATAAAGAGAGGATCCTAAAGGCAGCA

AGAGAAAAGCAGAATGTTACCTACAAGGGAACCCCCATAAGGTTATCAGCTGATTTCTCT

ACAGAAACACTACAGGCCAGGAGGGAATGGCAAGAGATATTTAAAGTGCTAAAAGGAAAA

AATATGCAACCTAGAATACTCTATCCAGCAAGAATATCATTTAAAATAGAAGGGGAAATA

AAAATTTTTTCCAACAAACAAAAGCTAAAAGAATACAGCAATACAAAACCCAGGCTAAAA

GAAATACTGAAAGGGCTTCTCTAAACCAAAAAGAAAGGAAGGAAAGAAAGGGAGGAAAAA

GAAAAAAAAAAAAAGAAGAGGAAGAACTAGGATTGAGGAAACCACAATCAGAGAGCAGTC

ACTCAAATAAGCCAGCATACAGATTTAATCATGAACATGTTTCAAACAAAATAAAATTAA

AAAGAAAAAAAGAGTCATCAAAATCATAAAATGTGGGCAAGGGATGTTAGGAAATAAATA

GACCCTTTTTTTTGTTTGTTTGTTTCTCTTCTTAATTTTAATATAGTAATGAAGTGTTTG

AACCTACAGGACCATCAGGCTAAAACACACAATTATAGGAAGGGGTTAGCATACTTAAAA

AACAGGGCAACCACAAGCCAAAACCAAACATTGCATTCGCAAAAAATAAAAAAAAAAACA

CTCAAGCAGATAATAACGGAGACCATCCAACCAAAAAAAGAAAGGAAGAATGGAGAACCA

TAGAATCAACTGGAACACGAGGTTCAAAATGGCAATAAATAATCATCTATCAATTATCAC

CTTAAATGTCAATGGACTGAATGCTCCAATCAAAAGACACAGAGTGGCTGAGTGGATAAA

AAGGCAAAAACCTTCAATATGCTGCCTACAAGAAACTCACCTTAGGACAAAGGATACATA

TAGATTGAAAGTGAAGGGGTGGGAAAAAATATTTCATGCCAATAGACATGACAGGAAAGC

AGGAGTTGCAATACTCATATCAGACAAAATAGACTTTAAAACAAAAGACATAAAGAAAGA

CAAAGAAGGACACTATTTAATGATTAAGGGATCCATCCAAGGAGAGGATGTTACTATCGT

CAACATATATGCCCCAAATACAGGAGCACCCAGATACATACAACAAATATTAACAGACAT

AAAGGGAGAAATTGATGGGAATACAATCATAGTAGGAGACTTTAATACCCCACTCACACC

AATGGACAGATCCTCTAGACAGAAAACCAATAAAGCAACAGAGATCCTAAAGGAAACAAT

AGAAAAGTTAGACTTAATTGATATCTTCAGGACACTACATCCAAAAAAATCAGAATACAC

ATTCTTCTCAAGTGCACATGGAACATTCTCAAGAATCGACCACATATTGGGACACAAAGC

TAACCTCAACAAATTTAGGAGCATAGAAATTATCTCAAGTATCTTCTCTGACCACAATGC

CATGAAATAGAAATCAACCATGGGAAAAGAAATGAGAAAAAACCTACTACATGGAGACTA

AACAACATGCTACTAAAAAACCAATGGGTCAATGAGGAAATCAAGAAGGAAATTAAAAAT

ACCTTGAAACAAATGATAATGAAGACACAACCTCTCAAAATCTATGGGATGCTGCAAAAG

CAGTGCTCAGAGGGAAATTTATAGCAATACAGGCCTTTCTCAAAAAAGAAGAAAGATCCC

AAATTGACAACTTAACCCTCCACCTAAATGAATTAGAAAAAGAAGAACAAAAAAGACCTA

AAGTCAGCAGAAGGAAGGAAATTATAAAGATCAAAGAAGAAATCAATAAAATAGAGATTC

AAAAAACAATAGAGAAAATTAATAAAACCAAGAGCTGGTTCTTTGAAAAGGTAAACAAAA

TTGACAAACCCCTGGCCAGACTCACTAAAAAGAGGAGAGAAAGAACTCAAATAAACAAAA

TTATAAATGAAAAAGGAGAAATCACAACGGATACTGCAGAAATACAAAAAACCATAAGAG

AATACTATGAACAACTATATGCCAACAAATTTGACAATCTGGAAGAAATGGACAATTTTC

TAGAATCTTACAGCCTGCCAAAACTGAATCAAGAAGAAACAGACCAACTGAACAGACCGA

TCACTAGAAATGAAATTGAAGAGTCATAAAAACACTCCCTACAAATAAAAGTCCAGGACC

AGATGGCTTCACAGGCGAATTCTATCAAACATATAAAGAGGATCTGGTGCCCATCCTCCT

TAAACTCTTTCAAAAGGTTGAAGAAGAAGGAATACTCCCAAAGACATTCTATGATGCCAC

CATCACCCTATTCCAAAACCAGACAAAGATACCACCAAAAAAGAAAACTATCGGCCAATA

TCTTTGATGAATATAGATGCAAAAATTCTCAACAAAATCTTAGCCAACCGAATCCAACAA

CATATCAAAAAAATCATACACCATGACCAGGTAGGGTTCATCCCAGGTTCACAAGGATGG

TTCAACATAGCAAATCAATCAACGTCATACACCACATTAACAAAAGAAAAGTCAAAAATC

ATATGATCATCTCAATAGATGCAGAAAAAGCATTTGACAAAGTCCAACATCCATTCATGA

TCAAGACCCTCGCCAAAGTGGGTATAGAGGGAACATTCCTGAACATAATCAAAGCCATTT

ATGACAAACCCACAGCAAATATAATACTCAATGGGGAAAAACTGAAAGCCTTCTCACTCA

AATCTGGAACAAGACAGGGATGCCCACTCTCACCACTGCTCTTCAACATAGTTTTGGAAG

TCCTAGCCACAGCAATTAGACAAACAAAAGAAATAAAAGGCATCCATATAGGAAGAGAAG

AGATAAAACTGTCACTGTATGCAGATGACATGATACTATACATAGAAAACCCTAAGGACT

CAACCCAAAAACTACTTGAACTGATTAATAAATTCAGCAAAGTAGCAGGATATAAGATTA

ACATTCAGAAGTCAGTGCATTTCTGTATACCAGCAATGAAATATTAGAAAAGGAATACAA

AAATACGATACCTTTTAAAATTGCACCTCACAAAATCAAATACCTCGGAATACACCTGAC

CAAGGAGGTAAAGGACTTATATGCCGAGAACTATAAAACTTTAATCAAAGAAATCAAAGA

AGATGTAAAGAAATGGAAAGATATTCCATGTTCTGGATTGGAAAAATCAATATTGTAAAA

ATGGCCATACTACCCAAAGCAATCTACAGATTCAATGCAATCCCTATCAAATTACCCATG

ACATTTTTCACAGAACTAGAACAAACAATCCAAACATTTATATGGAACCACAAAAGACCC

AGAATGCCAAAGCAATCCTGAGAAACAAAAACCAAGCAGGAGGCATAACTCTCCCAGACT

TCAAGAAATATTACAAAGCCACAGTCATCAAAACAGTGTGGTACTGGTATCAAAACAGAC

AGACAGACCAATGGAACAGAATAGAGAACCCGAAATAAACCCTGACACCTATGGTCAATT

AATCTTTGACAAGGGAGGCAAGAACATAAAATGGGAAAAAGAAAGTCTATTCAGCAAGCA

TTGCTGGGAAACCTGGACAGCTGCATGCAAAGCAATGAAACTAGAACACACCCTCACACC

ATGCACAAAAATAAACTCAAAATGGCTGAAAGACTTAAATATAAGACAGGACACCATCAA

ACTCCTAGAAGAGAACATAGGCAAAACACTCTCTGACATCAACATCATGAATATTTTCTC

AGGTCAGTCTCCCAAAGCAATAGAAATAAGAGCAAAAATAAACCCATGGGACCTATCAAA

CTGAAAAGCTTTTGCACAGCAAAGGAAACCAAAAGAAAACAAAAAGACAACTTACAGAAT

GGGAGAAAATAGTTTCAAATGATGCAACGACAAGGGCTTAATCTCTAGAATATATAAGCA

ACTTATACAACTCAACAGCAAAAAAGCCAATCAATCAATGGAAAAATGGGCAAAAGACCT

GAATAGACTGTTCTCCAAGGAAGATATACAGATGGCCAGCAAACACATGAAAAAATGCTC

AACATCGCTGATTATAAGAGAAATGCAAATCAAAACTACCATGAGATACCACCTCACACC

AGTCAGAATGGCCATCATTAATAAGTCCACAAATAACAAGTGCTGGAGGGGTGTGGAGAA

AAGGGAACCCTCCTGCACTGTTGGTGGGAATGTAAACTGGTACAGCCACTATGGAGAACA

GTTTGGAGATACCTTAGAAATCTATACATAGAACTTCCATATGACCCCGCAATCCCACTC

TTGGGCATCTATCCGGACAAAACTCTACTTAAAAGAGACACATGCACCCGCATGTTCATT

GCAGCACTATTCACAATAGCCAGGACATGGAAACAACCCAAATGTCCATCACAGATGATT

GGATTGGAAGAGTGGTATATATACACAATGGAATACTACTCAGCCATAAAAAAGAATGAC

ATAATGCCATTTGCAGCAACATGGATGGAACTAGAGACTCTCATACTGAGTGAAATGAGC

CAGAAAGACAAAGACAAATACCATATGATATCACTTATAACTGGAATCTAATATCCAGCA

CAAATGAACATCTCCACAGAAAAGAAAATCATGGACTTGGAGAATAGACTTGTGGCTGCC

TGATGGGAGAGGGAGGGAGTGGGAGGGATCGGAGCTTGGGGTTATCAGATACAACTTAGA

ATAGATTTACAAGGAGATCCTGCTGAGTAGCATTGAGAACTATGTCTAGATACTCATATT

GCAACAGAACAAAGGGTGGGGGAAAAAATGTATACATGTAAGAATAACTTGATCCCCATG

CTGTACAGTGGGAAAAAAAAAAAAAAAAAAAAA

>L1C5#LINE/L1C

GGAGGCAAGATGGCGGAAGAGTAGGGGGACACGCTCGCCCTCTCCCACAAACACAACAAA

AAAAAACACATCTACAGGTTAAAGACTCACACAGAACAGCAACAATCGCTGGCAGAAGAA

CCTAAACTCCAATAACGGCAAGAATCTCGTGACATAACTGGGTAAAACAAGAGAAAAGAG

GAGAGTGAGAGAAGGGGAATCGGGCTGGACGGGGCTCCCGAAAGGGAACTGTGGAGGAGA

AAGGGATCCCACACCCTGGAAAGTCACCTACTCGAGGAAAGATCAACCGAATCGGAGGAT

CTCCAGATGCAGAGAAGAGTGCAGCAGTAAGTCTGAGTTCTGAAAAGCAGAGTGAGAACC

GAACAGATCATCTGAACTACTGGCACAGTCACCAAAAATGAGAGCTTGGGTGGGGGCTGG

GCACCGAGACCTCGGCTCCGAGGTTAGTCCCCGGAGGCGGGTGGGCGGGGAGGGCTAGGA

ACCGTCTGTCAGGTTGCGGGGCAGAGACTGCCTGGGAGACTAGGAAGCAGAGCGTCGCGG

GTGGAGGGAGCAATACGCTAAGGGCTGGGGAGTGGAAAGCCACATCAGAGGGAACCTGGG

AGAAGAGCTGGATCTGCAGGAGAGACAAGGGCCAGTGTTGGGGAGGGGAGAGAGGAGGGG

TGGGCCACCATAGAATACTCCCCACGCCACAGCAGCTCACTTGCCCGCCAGCTATCAGAA

AGCTGTGCTTCCCAGTGCATCCCCCCCCCTACCCCTCGCACCCGACCTGAGGCTGCCTGC

CATCCGGAGGGCTGGCCTCACCATTGCGGAAGCCACCACCGCAGGGGCTTTCCCTGCCCT

GGCCTGCCTGCCCTCTGGAGGGGCTACACCCCCGCGAGCAGCACCCAGCACCCCAGCCCC

CGGAAAAGGCCTCAGCCCAGAAAAGCTAGAACAAGCTCGCCGGCCTGAAAATCTGCCTCC

ATTGCGGGCAGTCCTCCAATCGGCTGCCCTGGGGAAGAGCCCCTTGGGTTCTCAGTGGCC

CAGCTAGCTCTCCGCCCTGGGGGGTGCTGCACTCCCGTGGAACAGCTGCCCAGCACCGCC

AGCCCCCTGAAGAGCCCCACAGCCCAGAAACACCAGAGCAAGCTCTGCCCAGCCAGTGAA

ATCTGCTTCCATCGGGTGCGGACCTCCCAGTCCTGTCTGCCCTCAGGAAGTCCTCCTTTG

CTTCAGAGAGACCCTGTCAGCCCCACCCACCCTCGGAAAAGCCACACTGCCTCAAAGAAG

ACTGACCAACAACGCCAGCCCTCAGGAAACATTCCACAGCAGTGCCAAGGCAAACCTGCC

CGGCCACGGAGAGTACAACTCCACCAGAAAAAGAAAACAACAAGCAAGATGAAGAAGCTG

AGAAACCACCCCCAGTTAAACCAACAGGAGAACTCACCTAAAGCAGTCAACAATGAAACA

GATCTCTGCAGTCTGACAGACCTGGAGTTCAAAAGAGAAATAGTGAAAATACTGAAGGAA

TTAAGAGAAGATATGAACAGTAATGCAGACACCCTCAGAAAGGAACTAGAAAATATAAGG

AGGAGCCAAGAAAAACTAGAAAATTCATTTGCAGAGATACAAACTGAGCTAAGGGCAGTA

AAAACCAGAATGAATAATGCAGAAGAACGAATTAGTGATGTGGAAGATAGAATAATGGAA

ATCACCCAATCAGGCAGCAGACAGAAAACCAAATGAAAAAACATGAAAGCAATATAAGAG

ACCTATGGGATAATATAAAGCGGGCCAATCTACGCATAATAGGAATTCCAGAAGGAGTAG

AAAAAGATAAGGGGATGGAAAATATATTTGAAGAAATTATCACTGGAAACTTCCCAAATC

TAAAGGATACTGATTCAAGATACAGGAAGCACAGAGGGCCCCAAACAAGTTGAACCCAAA

TAGACCCACACCAAGACACATTATAATAAAAATGGCAAAAGTTAGTGATAAAGAGAGGAT

CCTAAAGGCAGCAAGAGAAAAGCAGAATGTTACCTATAAGGGAACCCCCATAAGGTTATC

AGCTGATTTCTCTACAGAAACTCTACAGGCCAGGAGGGAATGGCAAGAGATATTTAAAGT

GCTAAAAGGAAAAAATATGCAACCTAGAATACTCTATCCAGCAAGAATATCATTTAAAAT

AGAAGGGGAAATAAAAATTTTTTCCAACAAACAAAAGCTAAAAGAATACAGCAATACAAA

ACCCAGGCTAAAAGAAATATTGAAAGGGCTTCTCTAAACCAAAAAAAAGGAAGAAGGAAG

AAAAAAAACCACAATCAGAGAGCAGTCACTCAAATAAGCCAGCATACAGATTTAATCATG

AACATGTTTAAACAAAATAAAATTAAAAAAAAAAAAAGAGCATCAAAATCATAAAATGTG

GGCAAGGGAGTAAGGAAATAAATAGACTCTTTTTTTTTTTGTTTTTTCTCTTCTTAATTT

TAGTATAGTAATGAAGTGTTTGAACCTACAGGACCATCAGGCTAAAACACACAATTATAG

GAAGGGGTTAGCATACTTAAAAAACAGGGCAACCACAACAAAACCAAACATTGCATTCCA

AAAAAAAAAAAAAACACTCAAGCAGAAAATAATTGGAGACCATCCAACCAAAAAAAGAAA

GGAAGAATGGAGAACATAGAATCAACTGGAACACGAGGTTAAAATGGCAATAAATAATCA

TCTATCAATTATCACCTTAAATGTCAATGGACTGAATGCTCCAATCAAAAGACACAGAGT

GGCTGAGTGGATAAAAAGCAAAAACCTTCAATTGCTGCCTACAAGAAACTCACCTTAGGA

CAAAGGAACATATAGATTGAAAGTGAAGGGGTGGGAAAAAATATTTCATGCCAATAGACA

TGACAGGAAAGCAGGAGTTGCAATACTCATATCAGACAAAATAGACTTTAAAACAAAAGA

CATAAAGAAAGACAAAGAAGGACACTATTTAATGATTAAGGGATCCATCCAAGGAGAGGA

TTTACTATCGTCAACATATATGCCCCAAATATAGGAGCACCCAGATACATACAACAAATA

TTAACAGACATAAAGGGAGAAATTGATGGGAATACAATCATAGTAGGAGACTTTAATACC

CCACTCACATCAATGGACAGATCCTCTAGACAGAAAACCAATAAAGCAACAGAGATCCTA

AAGGAAACAATAGAAAAGTTAGACTTAATTGACATCTTCAGGACACTACATCCAAAAAAA

TCAGAATACACATTCTTCTCAAGTGCACATGGAACATTCTCAAGAATCGACCACATATTG

GGACACAAAGCTAACCTCAACAAATTTAGGAGCATAGAAATTATTTCAAGTATCTTCTCT

GACCACAATGGCATGAAACTAGAAATCAACCACAGGAAAAGAAATGAGAAAAAACCTACT

ACATGGAGACTAAACAACATGCTACTAAAAAACCAATGGGTCAATGAGGAAATCAAGAAG

GAAATTAAAAAATACCTTGAGACAAATGATAATGAAGACACAACCTCTCAAAATCTATGG

GATGCTGCAAAAGCAGTGCTCAGAGGGAAATTATAGCAATACAGGCCTTTCTCAAAAAAG

AAGAAAGATCCCAAATTGACAACTTAACCCTCCACCTAAATGAATTAGAAAAAGAAGAAC

AAAAAAGACCTAAAGTCAGCAGAAGGAAGGAAATTATAAAGATCAAAGAGGAAATCAATA

AAATAGAGATTCAAAAAACAATAGAGAAAATTAATAAAACCAAGAGCTGGTTCTTTGAAA

AGGTAAACAAAATTGACAAACCCCTGGCAGACTCACTAAAAGAGGAGAGAAAGAACCCAA

ATAAACAAAATTATAAATGAAAAAGGAGAAATCACAACGGATACTGCAGAAATACAAAAA

ACCATAAGAGAATACTATGAACAACTATATGCCAACAAATTTGACAATCTGGAAGAAATG

GACAATTTTCTAGAATCTTACAGCCTGCCAAAACTGAATCAAGAAGAAACAGACCAACTG

AACAGACCGATCACTAGAAATGAAATTGAAGAGTCATAAAAACACTCCCTACAAATAAAA

GTCCAGGACCAGATGGCTTCACAGGGAATTCTACCAAACATATAAAGAGGATCTGGTGCC

CATCCTCCTTAAACTTTTTCAAAAGGTTGAAGAAGAAGGAACACTCCCAAAGACATTCTA

TGATGCCACCATCACCCTAATTCCAAAACCAGACAAAGATACCACCAAAAAAGAAAACTA

TCGGCCAATATCTTTGATGAATATAGATGCAAAAATTCTCAACAAAATCTTAGCCAACCG

AATCCAACAACATATCAAAAAGATCATACACCATGACCAGGTGGGTTCATCCCAGGTTCA

CAAGGATGGTTCAACATATGCAAATCAATCAACGTCATACACCACATTAACAAAAGAAAA

GTCAAAAATCATATGATCATCTCAATAGATGCAGAAAAAGCATTTGACAAAGTCCAACAT

CCATTCATGATCAAACCTCGCCAAAGTGGGTATAGAGGGAACATTCCTGAACATAATCAA

AGCCATTTATGACAAACCCACAGCAAATATAATACTCAATGGAGAAAAACTGAAAGCCTT

CTCACTCAAATCTGGAACAAGACAGGGATGCCCACTCTCACCACTGCTCTTCAACATAGT

TTTGGAAGTCCTAGCCACAGCAATTAGACAAACAAAAGAAATAAAAGGCATCCATATAGG

AAGAGAAGAGATAAAACTGTCACTGTATGCAGATGACATGATACTATACATAGAAAACCC

TAAGGACTCAACCCAAAAACTACTTGAACTGATTAATAAATTCAGCAAAGTAGCAGGATA

TAAGATTAACATTCAGAAATCAGTCGCATTTCTGTATACCAACAATGAAATATTAGAAAA

GGAATACAAAAATACAATACCTTTTAAAATTGCACCTCAAAAAATCAAATACCTGGAATA

CACCTGACCAAGGAGGTAAAGGACTTATATGCCGAGAACTATAAAACTTTAATCAAAGAA

ATTAAAGAAGATGTAAAGAAATGGAAAGATATTCCATGTTCCTGGATTGGAAAAATCAAT

ATTGTAAAAATGGCCATACTACCCAAAGCAATCTACAGATTCAATGCAATCCCTATCAAA

TTACCCATGACATTTTTCACAGAACTAGAACAAACAATCCAAAATTTATATGGAACCACA

AAAGACCCAGAATTGCCAAAGCAATCCTGAGAAACAAAAACCAAGCAGGAGGCATAACTC

TCCCAGACTTCAAGAATATTACAAAGCCACAGTCATCAAAACAGTGTGGTACTGGTATCA

AAACAGACAGACAGACCAATGGAACAGAATAGAGAACCCAGAAATAAACCCTGACACCTA

TGGTCAATTAATCTTTGACAAGGGAGGCAAGAACATAAAATGGGAAAAAGAAAGTCTATT

CAGCAAGCATTGCTGGGAAACCTGGACAGCTGCATGCAAAGCAATGAAACTAGAACACAC

CCTCACACCATGCACAAAAATAAACTCAAAATGGCTGAAAGACTTAAATATAAGACAAGA

CACCATCAAACTCCTAGAAGAGAACATAGGCAAAACACTCTCTGACATCAACTCATGAAT

ATTTTCTCAGGTCAGTCTCCCAAAGCAACAGAAATAAGAGCAAAAATAAACCATGGGACC

TAATCAAACTGAAAGCTTTTGCACAGCAAAGGAAACCAAAAAGAAAACAAAAAGACAACT

TACAGAATGGGAGAAAATAGTTTCAAATGATGCAACGACAAGGGCTTAATCTCTAGAATA

TATAAGCAACTTATACAACTCAACAGCAAAAAAGCCAATCACCCAATGGAAAAATGGGCA

AAAGACCTGAATAGACTTCTCCAAGGAAGATATACAGATGGCCACAAACACATGAAAAAA

TGCTCAACATCCTGATTATAGAGAAATGCAAATCAAAACTACCATGAGATACCACCTCAC

ACCAGTCAGAATGGCCATCATTAATAAGTCCACAAATAACAAGTGCTGGAGGGGTGTGGA

GAAAAGGGAACCCTCCTGCACTGTTGGTGGGAATGTAAACTGGTACAGCCACTATGGAGA

ACAGTTTGGAGATACCTTAGAAATCTATACATAGAACTTCCATATGACCCGCAATCCCAC

TCTTGGGCATATATCCAGACAAAACTTCCTTAAAAAGACACATGCACCCGCATGTTCATT

GCAGCACTATTCACAATAGCCAAGACATGGAAACAACCCAAATGTCCATCGACAGATGAT

TGGATTAGGAAGAGTGGTATATATACACAATGGAATACTACTCAGCCATAAAAAAGAATG

ACATAATGCCATTTGCAGCAACATGGATGGAACTAGAGACTCTCATCTGAGTGAAATGAG

TCAGAAAGACAAAGACAAATACCATATGATATCACTTATAACTGGAATCTAATATCAGCA

CAAATGAACATTCCACAGAAAAGAAAATCATGGACTTGGAGAATAGACTTGTGGCTGCCG

GGGAGAGGGAGGGAGTGGGAGGGATCGGGAGCTTGGGGTTATCAGATACAACTTGGAATG

ATTTACAAGAGATCCTGCTGAGTAGCATTGAGAACTATGTCTAGATACTATATTGCAACA

GAACAAAGGGGGAAAAAAATGTATACATGTAAGTTAACTTGTCCCCATGCTGTACAGGGA

AAAAAAAAAAAAAAAAAAAAAAAA

>L1C6#LINE/L1C

GAAGGCAAGATGGCAGAAGAGTAGGGGGACACGCTCGCCCTCTCCCACAAACACACAACA

AAAAACACATCTACAGGTTAAACGACTCGCACAGAACAGCAATTAATCGCTGGCAGAAGA

ACCTAAACTCCAATAATGGCAAGAATCTCGTGACATAACTGGGTAAAACAAGAGAAAAGA

GGAGAGTGAGAGAAGGGGAATCGGGGCTGGACGGGCGCTCCCGAAAGGGAACTGTGGAGG

AGAAAGGGATCCCACACCCTGGAAAGTCACCTACTCGACGGAAAGATCAACCGAATCGGA

GGGATCTCCAGATGCAGAGAAGAGCGCAGCAGTAAGTCTGAGATCTGAAGAGCAGAGTGA

GAACCGAACAGATCATCTGAACTACTGGCACAGTCACCAAAAACCGAGACGCTTGGGTGG

GGGCTGGGCACGAGACCTCGCTCCGGAGGTTAGTCCCCGGAACGGCTGGGGTGGGCGGTG

CGAGACTGCTTGGGGGACTAGGAACCGGTCGTCGTTCAGGGCAGAGACTGCCGGGACTAG

GAATTGCGGCAGAGCTGACTAGAAGAGTGCGGGTGGAGGGAGCAATACGCTAAGGGCTGG

GGAGTGGAAAGCCACATCAGAGGGAACCTGGGAGAAGAGCTGGATCTGCAGGAGAGACAA

GGCGCCAGTGTTGGGGAGGGGAGAGGAGGAGGGGTGGGCCACCATAGAATACTCCCCACA

CCACAGTGAGCTCACTTGCCCCCAGCTATCAGAAAGCTGTGCTTCCCAGTGCATCCCCCC

TCCCCCTACCCCGCGGCCGGACCTGAGGCTGCCTGCCATCCCGGAGGGCTGGCCTCACCA

TTTGCAGGAAGCCGACCACGCAGGGGCTTTCCCTGCCCTGGCCTGCCTGCCCTCTGGAGG

GGCTACACCCCCGCAGAGCAGCACCCAGCACCGCCAGCCCCCGGAAAAGGCCCCAGCCCA

GAAAAGCTAGAACAAGCTCGGCCGGCCGTGCAAAATCTGCCTCCATTGCGGCTCCTGCCA

ATTCCGCTGCCCTGGGGAAGAGCCCCTTGGGTTCTCAGGGCCCAGCTGCTGCTCCCACCC

TTGGGGGGTGCTGCACTCCGTGGAACAGCTGCCAGCACCGCCAGCCCCCTAGAAGAGCCC

CACAGCCCAGAAACACCAGAGCAAGCTCTGCCCAGCCGCGTGAAATCTGCTTCCATCCAG

TGCAGACCTCCCAGTCCTGTCTGCCCTCAGGAAGTCCTCCTTTGCTTCAGAGAGACCCTG

TCAGCCCCGCCCACCCTCAGAAAACCAGCTGCCTCAAAGAAGACTGACCAACAACGCCAG

CCCTCGGAAACATTCCACAGCAGTGCCAAGGCAAACCCTGCCTGGCCACGGGGAGTACAA

CTCCACCAAGAAAAAGAAAACAACAAGCAAGATGAAGAAGCTGAGAAACCACCCCCAGTT

AAACCAACAGGAGAACTCACCTAAAGCAGTCAACAATGAAACAGACCTCTGCAGTCTGAC

AGACTTGGAGTTCAAAAGGGAAATAGTGAAAATACTGAAGGAATTAAGAGAAGATATGAA

CAGTAATGCAGATGCCCTCAGAAAGGAACTAGAAAATATAAGGAGGAGCCAAGAAAAACT

AGAAAATTCATTTGCAGAGATACAAACTGAGCTAAGGGCAGTAAAAACCAGAATGAATAA

TGCAGAAGAACGAATTAGTGATGTGGAAGATAGAATAATGGAAATCACCCAAACAGGACA

GCAGACAGAAAACCAAATGAAAAAACATGAAAGCAATATAAGAGACCTATGGGATAATAT

AAAGCGGGCCAATCTAGCATAATAGGAATTCCAGAAGGAGTAGAAAAAGATAAGGGGATG

GAAAATATATTTGAAGAAATTATCACTGGAAACTTCCCAAATCTAAAGGATACTGATTTC

AAGATACAGGAAGCACAGAGGGCCCCAAACAAGTTGAACCCAAATAGACCCACACCAAGA

CACATTATAATAAAAATGGCAAAAGTTAATGATAAAGAGAGGATCCTAAAGGCAGCAAGA

GAAAAGCAAAATGTTACCTATAAGGGAACCCCCATAAGGTTATCAGCTGATTTCTCTACA

GAAACTCTACAGGCCAGGAGGGAATGGCAAGAGATATTTAAAGTGCTAAAAGGAAAAAAT

ATGCAACCTAGAATACTCTATCCAGCAAGAATATCATTTAAAATAGAAGGGGAAATAAAA

TTTTTTTCCAACAAACAAAAGCTAAAAGAATACAGCAATACAAAACCCAGGCTAAAAGAA

ATATTGAAAGGGCTTCTCTAAACCAAAAAGAAAGGAGAATGAGGAAACCACAATCAGAGA

GCAGTCACTCAAATAAGCCAGCATACAGATTTAATCATGAACATGTTTAAAACAAAATAA

AATTAAAAAGAAAAAAAGAGTCATCAAAATCATAAAATGTGGGCAAGGGAAGTAAGGAAA

TAAATAGACTCTTTTTAAAATTTTTTTTTTTTTCTTTTTTTTTTTTTTTTCTTCTTAATT

TTAGTATAGTAATGAAGTGTTTGAACCTACAGGACCATCAGGCTAAAACACACAATTATA

GGAAGGGGTTAGCATACTTAAAAACAGGGCAACCACAAATCAAAACCAAACATTGCATTC

GCAAAAAATGAAAAGAAAAACACTCAAGCAGAAAATAATTGGAGACCATCCAACCAAAAA

AAGAAAGGAAGAATGGAGAATCATAGAATCAACTGGAACACGAGGTTTAAAATGGCAATA

AATAATCATCTATCAATTATCACCTTAAATGTCAATGGACTGAATGCTCCAATCAAAAGA

CACAGAGTGGCTGAGTGGATAAAAAAGCAAAAACCTTCAATCTGCTGCCTACAAGAAACT

CACCTTAGGACAAAGGACACATATAGATTGAAAGTGAAGGGGTGGGAAAAAATATTTCAT

GCCAATAGACATGACAGGAAAGCAGGAGTTGCAATACTCATATCAGACAAAATAGACTTT

AAAACAAAAGACATAAAGAAAGACAAAGAAGGACACTATTTAATGATTAAGGGATCCATC

CAAGAAGAGGATATTACTATCGTCAACATATATGCCCCAAATACAGGAGCACCCAGATAC

ATACAACAAATATTAACAGACATAAAGGGAGAAATTGATGGGAATACAATCATAGTAGGA

GACTTTAATACCCCACTCACATCAATGGACAGATCCTCTAGACAGAAAACCAATAAAGCA

ACAGAGATCCTAAAGGAAACAATAGAAAAGTTAGACTTAATTGACATCTTCAGGACACTA

CATCCAAAAAAATCAGAATACACATTCTTCTCAAGTGCACATGGAACATTCTCAAGAATC

GACCACATATTGGGACACAAAGCTAACCTCAACAAATTTAGGAGCATAGAAATTATTTCA

AGTATCTTCTCTGACCACAATGGCATGAAACTAGAAATCAACCACAGGAAAAAAAATGAG

AAAAAACCTACTACATGGAGACTAAACAACATGCTACTAAAAAACCAATGGGTCAATGAG

GAAATCAAGAAGGAAATTAAAAAATACCTTGAGACAAATGATAATGAAGACACAACCTCT

CAAAATCTATGGGAGCTGCAAAAGCAGTGCTCAGAGGGAAATTCATAGCGATACAGGCCT

TCCTCAAAAAAGAAGAAAGATCTCAAATTGACAACTTAACCCTCCACCTAAACGAATTAG

AAAAAGAAGAACAAAAAAGACCTAAAGTCAGCAGAAGGAAGGAAATTATAAAGATCAAAG

AGGAAATCAATAAAATAGAGATTCAAAAAACAATAGAGAAAATTAATAAAACCAAGAGCT

GGTTCTTTGAAAAGGTAAACAAAATTGACAAACCCCTGGCTAGACTCACTAAGAAGAGGA

GAGAAAGAACCCAAATAAACAAAATTAGAAATGAAAAAGGAGAAATCACAACGGATACTG

CAGAAATACAAAAAACTAAGAGAATACTATGAACAACTATATGCCAACAAATTTGACAAT

CTGGAAGAAATGGACAATTTTCTAGAATCTTACAGCCTGCCAAAACTGAATCAAGAAGAA

ACAGACCAACTGAACAGACCGATCACTAGAAATGAAATTGAAGATGTCATAAAAACACTC

CCTACAAATAAAAGTCCAGGACCAGATGGCTTCACAGGGAATTCTACCAAACATATAAAG

AGGATCTGGTGCCCATCCTCCTTAAACTTTTTCAAAAGGTTGAAGAAGAAGGAACACTCC

CAAAGACATTCTATGATGCCACCATCACCCTAATTCCAAAACCAGACAAAGATACCACCA

AAAAAGAAAACTATCGGCCAATATCTTTGATGAATATAGATGCAAAAATTCTCAACAAAA

TCTTAGCCAACCGAATCCAACAACATATCAAAAAGATCATACACCATGACCAGGTAGGGT

TCATCCCAGGTTCACAAGGATGGTTCAACATACGCAAATCAATCAACGTCATACACCACA

TTAACAAAAGAAAAGTCAAAAACCATATGATCATCTCAATAGATGCAGAAAAAGCATTTG

ACAAAGTCCAACATCCATTCATGATCAAAACTCTCGCCAAAGTGGGTATAGAGGGAACAT

TCCTGAACATAATCAAAGCCATTTATGACAAACCCACAGCAAATATAATACTCAATGGAG

AAAAACTGAAAGCCTTCTCACTCAAATCTGGAACAAGACAGGGATGCCCACTCTCACCAC

TGCTATTCAACATAGTTTTGGAAGTCCTAGCCACAGCAATTAGACAAACAAAAGAAATAA

AAGGCATCCAAATAGGAAGAGAAGAGATAAAACTGTCACTGTATGCAGATGACATGATAC

TATACATAGAAAACCCTAAGGACTCAACCCAAAAACTACTTGAACTGATTAATAAATTCA

GCAAAGTAGCAGGATATAAGATTAACATTCAGAAATCAGTCGCATTTCTGTATACCAACA

ATGAAATATTAGAAAAGGAATACAAAAATACAATACCTTTTAAAATTGCACCTCAAAAAA

TCAAATACCTCGGAATACACCTGACCAAGGAGGTAAAGGACTTATATGCCGAGAACTATA

AAACTTTAATCAAAGAAATAAAGAAGATGTAAAGAAATGGAAAGATATTCCATGTTCATG

GATTGGAAAAATCAATATTGTAAAAATGGCCATACTACCCAAAGCAATCTACAGATTCAA

TGCAATCCCTATCAAATTACCCATGACATTTTTCACAGAACTAGAACAAACAATCCAAAC

ATTTATATGGAACCACAAAAGACCCAGAATTGCCAAAGCAATCCTGAGAAACAAAAACCA

AGCAGGGGCATAACTCTCCCAGACTTCAAGAAATATTACAAAGCCACAGTCATCAAAACA

GTGTGGTACTGGTATCAAAACAGACAGACAGACCAATGGAACAGAATAGAGAACCCAGAA

ATAAACCCTGACACCTATGGTCAATTAATCTTTGACAAGGGAGGCAAGAACATAAAATGG

GAAAAAGAAAGTCTATTCAGCAAGCATTGCTGGGAAACCTGGACAGCTGCATGCAAAGCA

ATGAAATTAGAACACACCCTCACACCATGCACAAAAATAAACTCAAAATGGCTGAAAGAC

TTAAATATAAGACAAGACACCATCAAACTCCTAGAAGAGAACATAGGCAAAACACTCTCT

GACATCAACCTCATGAATATTTTCTCAGGTCAGTCTCCCAAAGCAACAGAAATAAGAGCA

AAAATAAACCAATGGGACCTAATCAAACTGAAAAGCTTTTGCACAGCAAAGGAAACCAAA

AAGAAAACAAAAAGACAACTTACAGAATGGGAGAAAATAGTTTCAAATGATGCAACGACA

AGGGCTTAATCTCTAGAATATAAAGCAACTTATACAACTCAACAGCAAAAAAGCCAATCA

CCCAATGGAAAAATGGGCAAAAGACCTGAATAGACTTTCTCCAAGGAAGATATACAGATG

GCCAACAAGCACATGAAAAAATGCTCAACATCGCTGATTATAAGAGAAATGCAAATCAAA

ACTACCATGAGATACCACCTCACACCAGTCAGAATGGCCATCATTAATAAGTCCACAAAT

AACAAGTGCTGGAGGGGTGTGGAGAAAAGGGAACCCTCCTGCACTGTTGGTGGGAATGTA

AACTGGTACAGCCACTATGGAGAACAGTTTGGAGATACCTTAGAAATCTATACATAGAAC

TTCCATATGACCCCGCAATCCCACTCTTGGGCATATATCCGGACAAAACTCTACTTAAAA

GAGACACATGCACCCGCATGTTCATTGCAGCACTATTCACAATAGCCAAGACATGGAAAC

AACCCAAATGTCCATCGACAGATGATTGGATTAGGAAGATGTGGTATATATACACAATGG

AATACTACTCAGCCATAAAAAAGAATGACATAATGCCATTTCAGCAACATGGATGGAACT

AGAGACTCTCATACTGAGTGAAATAAGTCAGAAAGACAAAGACAAATACCATATGATATC

ACTTATAACTGGAATCTAATATACAGCACAAATGAACTTTCCACAGAAAAGAAAATCATG

GACTTGGAGAATAGACTTGTGGCTGCCCGGGGGGAGAGGGAGGGAGTGGGAGGGATCGGA

GCTTGGGGTTATCAGATACAACTTGGAATGGATTTACAATGAGATCCTGCTGAGTAGCAT

TGAGAACTATGTCTAGATACTTATATTGAACAGAACAAAGGGTGGGAAAAAAAATGTATA

CATGTAAGTGTAACTTGGCCCCATGCTGTACAGCGGAAAAAAAAAATAAAAAAAAAAAAA

AA

>L1C7#LINE/L1C

GAGAGGAGACAAGATGGCGGAAGAGTAGGGGGACACGCTCGCCCTCTCCCACAAACACAA

CAAAAAAAAACACATCTACAGGATAAATGACTCACACAGAACAGCAACCAATCGCTGGCA

GAAGAACCTAAACTCCAATAACGGCAAGAATTCGTGACATTACTAGGTAAAACAAGAGAA

AAGAGGAGAGTGAGAGAAGGTGAATCCGAGCTGGAGGGCGCTCCCGAAAGGGAACTGTGG

AGGAGAAAGGGATCCCACACCCTGGAAAGTCACCTACTCGAGGAAAGATCAACGAACCGA

GGAATCTCCAGATGCAGAGAAGAGTGTAGCAGTAAGTTGGAGTTCTGAAAAGCAGACGAG

AACCGAACAGATCATCTGAACTACGGGCACAGTCACCAAAAATTGAGACGCCTGGGTGGG

GGCTGGGCACCGAGACCTCGGCTCCGGAGGTTAGTCCCCGGGAAAGGGCTGGGGGGTGGA

GCGGAGACTGCTTGGGAGGTCTAGAAACCGGTCTGTCAAGTTTGACGGGGCAGAGACTGC

CTGGGAGACTAGAAAGCAGAGCTTCAGGGAGGGAGCAATACCTAAGGGCGGGGAAGTGGA

AAGCCACATCAGAGGGAACCTGGGAGAAGAGCCGGTCTGCGCCAGTGTTGGGGAGGGGAG

AGAAGAAGGGGTGGGCCCCCATAGAATACTCCCCACGCCACAGCAAGCTTACGGCCCGCT

AGCTACAGAAAGCTGTGCTTCCCAGTGCATCCCCCCCACCCCCCCACCCCTACGCACCGC

CGGACCTGGGGCTGCCTGCCATCCGGGAGGGCTGGCCTCAACAATTGCCTGAAGCCTACC

ACCGCAGGGGCTGTCCCTGCCTGGCCTGCTTGCCCTTTGGAGGGGCTACACCCCCGCAGA

GCAGCACCAAACACCACCAGCCCCCGGAAAAGGCCTGCAGCCCAGAAAAGCTAGAACAAG

CTTGGCCAGGCCGTGAAAAGATCTGCCTAATTCTCAGACAGTCTTTCGAGTCGGGCTGCC

CTGGGGAAGAGCCTCTTGGGTTCAGCGGCCCAGCTAGCTGCTCCAGCCCTCAGGGGGTGC

TGCACTCCGGGAACAGCTGCCCAGCACTGCCAGCCCCCTGCAGGACCCCTGCAGCCCAGA

AAAGCTAGAACAAGCTTGGCCAGGCCGTGAAAAGATCTGCCTACATTCTCAGGCAGTCCT

TCTGAGTTGGGCTGCCCTGGGGAAGAGCCTCTTGGGTTCTCAGTGACCCAGATAGCTGCT

CCAGCCCTCAGGGGGTGCTGCACTCCTGAGGAACAGCTGCCCAGCACCGCCAGCCCCCTG

CAAGAGCCCCACAGCCTAAAAACACCAGAGCAAGCTCTGCCTGCCAAGTGAAATCTGCTA

CCATCGTGGTGTGGACCTCCCAGTCCTGTCTGCCCTCAGGAAGTCCTCCTTTGCTTCAAA

GAGACCCTGTTAGCCCCATCAACACTCCAGAAAAGCCACACTGCCTCAAAAAAGACTGAC

CAACAACGCCAGCCCTCAGGAAACATTCCACGGCAGTGACAAGGCAAACACTGCCCGGTC

ACGGAGAGTACAACTCCCCAGGAAAAAGAAAACAACAAGCAAGATGAAGAAGCTGAGAAA

CCACCCCCAGTCAAACCAACAGGAGAACTCACCTAAAACAGTCAACAATGAAACAGATCT

CTGCAGTCTGACAGACCTGGAGTTCAAAAGAGAAATAGTGAAAATACTGAAGGAATTAAG

AGAAGATATGAACAGTAATGCAGACACCCTCAGAAAGGAACTAGAAAATATAAGGAGGAG

CCAAGAAAAACTAGAACATTCATTTGCAGAGATACAAACTGAACTAAGGGCAGTAAAAAC

CAGAATGAATAATGCAGAAGAACGAATCAGTGATATGGAAGATAGAATAATGGAAATCAC

CCAATCAGGTCAGCAGACAGAAAACCAAATCAAAAAACAGGAAAGCAATATAAGAGACCT

ATGGGATAATATAAAGCGGGCCAATCTACGCATAATAGGAATTCCAGAAGGAGTAGAAAA

AGATAAGGGAATGGAAAATATATTTGAAGAAATTATCGCTGGAAACTTCCCAAATCTAAA

GGATACTGAGTTCAAGATACAGGAAGCACAGAGGGCCCCAAACAAGTTGAACCCAAATAG

ACCCACACCAAGACACATATAATAAAAATGGCAAAAGTTAGTGATAAAGAGAGGATCCTA

AAGGCAGCAAGAGAAAAGCAGAATGTTACCTACAAGGGAACCCCCATAAGATTATCAGCT

GATTTCTCTACAGAAACACTACAGGCCAGGAGGGAATGGCAAGAGATATTTAAAGTGCTA

AAAGGAAAAAATATGCAACCTAGAATACTCTATCCAGCAAGAATATCATTTAAAATAGAA

GGGGAAATAAAAATTTTTTCCAACAAACAAAAACTAAAAGAATACAGCAACACAAAACCC

AGGCTAAAAGAAATACTGAAAGGGCTTCTCTAAACCAAAAAGAAAGGAAGGAAAGAAGAA

AAAAAAAAAAAAAAAAAAAAGAAGAGGAAGAACTAGGACTGAGGAAACCACAATCAGAGA

GCAGTCACTCAAATAAGCCAGCATACAGATTTAATCATGAACATGCTTCAAACAAAATAA

AATTAAAAAGAAAAAAAAAAAAGAGTCATCAAAATCATAAAATGTGGGCAAGGGATGTTA

GGAAATAAATAGACCCTTTTTTTTGTGTTTGTGTTTCTCTTCTTAATTTTAATATAGTAA

TGAAGTGTTTGAACCTACAGGACCATCAGGCTAAAACACACAATTATAGGAAGGGGTTAG

CATACTTAAAAAACAGGGCAACCACAAGCCAAAACCAAATATTGCATTCGCAAAAAATGA

AAAAAAAAAAAAAGCAGATAATAACAGGAGACCATCCAACCAAAAAAAAAAAAAAAAGAA

AGGAAGAATGGAGAACCATAGAATCAACTGGAACACGAGGTTCAAAATGGCAATAAATAA

TCATCTATCAATTATCACCTTAAATGTCAATGGACTGAATGCCCAATCAAAAGACACAGA

GTGGCTGAGTGGATAAAAAGGCAAAAACCTTCAATATGCTGCCTACAAGAAACTCACCTT

AGGACAAAGGATACATATAGATTGAAAGTGAAAGGGTGGGGAAAAATATTTCACGCCAAT

AGACATGACAGAAAAGCAGGAGTTGCAACACTCATATCAGACAAAATAGACTTTAAAACA

AAAGACATAAAGAAAGACAAAGAAGGACACTATTTAATGATTAAGGGATCCATCCAAGGA

GAGGATGTTACTATCGTCAACATATATGCCCCAAATATAGGAGCACCCAGATACATACAA

CAAATATTAACAGACATAAAGGGAGATATTGATGGAATACAATCATAGTAGGAGACCTTA

ATACCCCCCTCACATCAATGGACAGATCCTCTAGACAGAAAACCAATAAAGCAACAGAGA

TCCTAAAGGAAACAATAGAAAAGTTAGACTTAATTGATATCTTCAGGACACTACATCCAA

AAAAATCAGAATACACATTCTTCTCAAATGCCATGGAACATTCTCAAGAATCGACCACAT

ATTGGGACACAAAGCTAACCTCAATAAATTTAGGAGCATAGAAATTATCTCAAGTATCTT

CTCTGACCACAATGCCATGAAATTAGAAATCAACCATGGGAAAAGAAAGAGAAAAAACCT

ACTACATGGAGACTAAACAACATGCTACTAAAAAACCAATGGGTCAATGAGGAAATCAAG

AAGGAAATTAAAAACTACCTTGAAACAAATGATAATGAAGACACAACCTCTCAAAATCTA

TGGGATGCTGCGAAAGCAGTGCTCAGAGGGAAATTTATAGCAATACAGGCCTTTCTCAAA

AAAGAAGAAAGATCCCAAATTGACAACTTAACCCTCCACCTAAACGAATTAGAAAAAGAA

GAACAAAAAAGACCTAAAGTCAGCAGAAGGAAGGAAATTATAAAGATCAAAGAAGAAATC

AATAAAATAGAGATTCAAAAAACAATAGAGAAAATTAATAAAACCAAGAGCTGGTTCTTT

GAAAAGGTAACAAAATTGACAAACCCCTGGCCAGACTCACTAAAAAGAGGAGAGAAAGAA

CCCAAATAACCAAAATTATAAATGAAAAAGGAGAAATCACAACGGATACAGCAGAAATAC

AAAAAACCATAAGAGAATACTATGAACAACTATATGGCAACAAGTTTGACAATCTGGAAG

AAATGGACAATTTTCTAGAATCTTACAGCCTGCCAAAACTGAATCAAGAAGAAACAGACC

AACTGAACAGACCGATCACTAGAAATGAAATTGAAGAGGTCATAAAAACACTCCCTACAA

ATAAAAGTCCAGGACCAGATGGCTTCACAGGCGAATTCTATCAAACATATAAAGAGGAAC

TGGTGCCCATCCTCCTTAAACTCTTTCAAAAGGTTGAAGAAGAAGGAATACTCCCAAAGA

CATTCTATGATGCCACCATCACCCTCATTCCAAAACCAGACAGAGATACCACCAAAAAAG

AAAACTATCGGCCAATATCATTGATGAATATAGATGCAAAAATTCTCAACAAAATCTTAG

CCAACCGAATCCAACAACATATCAAAAAAATTATACACCATGACCAGGTTGGGTTCATCC

CAGGTTCACAAGGATGGTTCAACATACGCAAATCAATCACATCATACACCACATTAACAA

AAAAAAGTCAAAAATCATATGATCATCTCAATAGACGCAGAAAAAGCATTTGACAAAGTC

CAACATCCATTCATGATCAAGACCCTCGCCAAAGTGGGTATAGAGGGAACATTCCTGAAC

ATAATCAAAGCCATTTATGATAAACCCACAGCAAATATAATACTCAATGGGGAAAAACTG

AAAGCCTTCTCACTCAAATCTGGAACAAGACAGGGATGCCCACTCTCACCACTGCTCTTC

AACATAGTTTTGGAAGTCCTAGCCACAGCAATTAGACAAACAAAAGAAATAAAAGGCATC

CATATAGGAAGAGAAGAGATAAACTGTCACTGTATGCAGATGACATGATACTATACATAG

AAAACCCTAAGGACTCAACCCAAAAACTACTTGAACTGATTAATAAATTCAGCAAAGTAG

CAGGATATAAGATTAACATTCAGAAGTCAGTTGCATTTCTGTATACCAGCAATGAAATAT

TAGAAAAGGAATACAAAAATACGATACCTTTTAAAATTGCACCTCACAAAATCAAATACC

TCGGAATACACCTGACCAAGGAGGTAAAGGACCTATATGCCGAGAACTATAAAACTTTAA

TCAAAGAAATCAAAGAAGATGTAAAGAAATGGAAAGATATTCCATGTTCCTGGATTGGAA

AAATCAATATTGTAAAAATGGCCATACTACCCAAAGCAATCTACAGATTCAATGCAATCC

CTATCAAATTACCCATGACATTTTTCACAGAACTAGAACAAACAATCCAAACATTTATAT

GGAACCACAAAAGACCCAGAATCGCCAAAGCAATCCTGAGAAACAAAAACCAAGCAGGAG

GCATAACTCTCCCAGACTTCAAGAAATACTACAAAGCCACAGTCATCAAAACAGTGTGGT

ACTGGTATCAAAACAGACAGACAGACCAATGGAACAGAATAGAGAACCCGGAAATAAACC

CTGACACCTATGGTCAATTAATCTTTGACAAGGGAGGCAAGAACATAAAATGGGAAAAAG

AAAGTCTATTCAGCAAGCATTGCTGGGAAACCTGGACAGCTGCATGCAAAGCAATGAAAC

TAGAACACACCCTCACACCATGCACAAAAATAAACTCAAATGGCTGAAAGACTTAAATAT

ACGACAGGACACCATCAAACTCCTAGAAGAAAACATAGGCAAAACACTCTCTGACATCAA

CATCATGAATATTTTCTCAGGTCAGTCTCCCAAAGCAATAGAAATTAGAGCAAAAATAAA

CCCATGGGACCTATCAAACTGAAAAGCTTTTGCACAGCAAAGGAAACCCAAAAGAAAACA

AAAAGACAACTTACAGAATGGGAGAAAATAGTTTCAAATGATGCAACGACAAGGGCTTAA

TCTCTAGAATATATAAGCAACTTATACAACCCAACAGCAAAAAAGCCAATCAATCAATGG

AAAAATGGGCAAAAGACCTGAATAGACATTTCTCCAAGGAAGATATACAGATGGCCACAA

ACACATGAAAAAATGCTCAACATCGCTGATTATAAGAGAAATGCAAATCAAAACTACCAT

GAGATACCACCTCACACCAGTCAGAATGGCCATCATTAATAAATCCACAAATAACAAGTG

CTGGAGGGGCTGTGGAGAAAAGGGAACCCTCCTGCACTGTTGGTGGGAATGTAAACTGGT

ACAGCCACTATGGAGAACAGTTTGGAGATACCTTAGAAATCTATACATAGAACTTCCATA

TGACCCCGCAATCCCACTCTTGGGCATCTATCCGGACAAAACTCTACTTAAAAGAGACAC

ATGCACCCGCATGTTCATTGCAGCACTATTCACAATAGCCAGGACATGGAAACAACCCAA

ATGTCCATCACAGATGATTGGATTCGGAAGAGTGGTATATATACACAATGGAATACTACT

CAGCCATAAAAAAGAATGACATAATGCCATTTGCAGCAACATGGATGGAACTAGAGATCT

CATACTGAGTGAAATGAGCCAGAAAGACAAAGACAAATACCATATGATATCACTTATAAC

TGGAATCTAATATCCAGCACAAATGAACATCTCCTCAGAAAAGAAAATCATGGACTTGGA

GAAAGACTTGTGGCTGCCTGATGGGAGGGGAGGGAGTGGGAGGGATCGGGAGCTTGGGCT

TATCAGACACAACTTAGAATAGATTTACAAGGAGATCCTGCTGAGTAGCATTGAGAACTA

TGTCTAGATACTCATGTTGCAACAGAACAAAGGGTGGGGGAAAAAAATGTATACATGTAA

GGATAACTTGATCCCCTTGCTGTACAGTGGGAAAAAAAAAAAATAAAAAAAAAAAAAAAA

A

>L1C8#LINE/L1C

GGAGCAAGATGGCGGAAGAGTAGGGGGACACGCTCGCCCTCTCCCACAAACACAACAAAA

AAAACACATCTACAGGATAAAGACTCGCACAGAACAGCAACCAATCGCTGGCAGAAGAAC

CTAAACTCCAATAACGGCAAGAATTTCGTGACATTACTAGGTAAAACAAGAGAAAAGAGG

AGAGTGAGAGAAGGGAATCCGAGCTGGACGGTGCTCCTGAAAGGGAACTGGGAGGAGAAA

GGGATCCCGCACCCTGGAAAGTCACCTACTGAGGAAAGATCAACCGAACCGAGGAATCTC

CAGATGCAGAGAAGAGTGTAGCAGTAAGTTGGAGTTCTGAAAAGCAGATCGAGAACCGAA

CAGATCATCTGAACTATGGGCACAGTCACCAAAAATTGAGACGCCTGGGTGGGGGCTGGG

CACCGAGACCTCGGCTCCGAGGTTAGTCCCCAGGAAAGGGCTGGGGGGGGGGGTGGAGCG

GAGACTGCTTGGGAGGTCTAGAAACCGTCTGTCAAGTTTGACGGGGCAGAGACTGCCTGG

GAGACTAGAAAGCAAGCGTCCAGGGGGAGGGAGCAATACTCTAGGGGCGGGGAAGTGGAA

AGCCACATCAGAGGGAACCTGGGAGAAGAGCCTGGTCTGCGCCCGTGCTGGGGAGGGGAG

AGAAGAAGGGGTGGGTCCCCATAGAATACTCCCCATGCCACAGCAAGCTTACTGGCCCGC

TAGCTATCAGAAAGCTGTGCTTCCCAGTGCATCCCCTCCCCCCACCCCCCTATGCACACG

CCAGACCTGGGGCTGCCTGCCATCCGGGAGGGCTGGCCTCAACAATTGCCGAAGCCTACC

ACCACAGGGGCTGTCCCTGCCCTGGCCTGCTTGCCCTTTGGAGGGGCTACACCCCCGCAG

AGCAGCACCAAACACCACCAGCCCCCGGGAAAAGGCCTGCAGCCCAGAAAAGCTAGAACA

AGCTTAGCCAGGCCATGAAAAGATCTGCCTAATTCTCAGACAGTCCTTCTGAGTCAGGCT

GCCCTGGGGAAGAGCCTCTTGGGTTTGCAGGGCCCAGCTAGCTGCTCCAGCCCTCGGGGG

GTGCCGCGCTCCCTGGAACAGCTGCCCAGGACTGCCAGCTCCTGCAGGAACCCCTGCACC

CCAGAAAAGCTAAACAAGCCTGGCCGGGCCGTGAAAAGATCTGCCTAATTCTCGACAGTC

TTTCTGAGTCGGGCTGCCCTGGGGAAGAGCCTCTTGGGTTTGCAGCGCCCAGCTAGCTGC

TCCAGCCCTCGGGGGTGCGCTCGGGACAGCTGCAGGACTGCCAGCCCGAGGACCCCTGCA

GCCCAGAAAAGCTAAACAAGCCTGGCGGCCTGAAAAGATCTGCCTAATTCTCGACAGTCT

TCTGAGTGGGCTGCCCTGGGGAGAGCCTCTTGGGTTTCAGGCCCAGCTAGCGCTCCAGCC

CTCGGGGGGTGCCCACTCCTGCAGAACAGCTGCCAGCACGCCAGCCTCTGAGGAGCCCCT

GCAGCCCAGAAAAGCTACAACAAGCTTGGCCAGGCTGTGAAAAGATCTGCCTACATTCTC

AGACAGTCCTTCTGAGTTGGGCTGCCCTGGGGAAGAGCCTCTTGGGTTCTCAGTGACCCA

GATAGCTGCTCAGCCCTCGGGGGGTGCTGCACTCCTGGGAACAGCTGCCCAGCACCGCCA

GCCCCCTGCAAGAGCCCCACAGCCTAAAAACACCAGAGCAAGCTCTGCCTGGCCGAGTGA

AATCTGCTACCATCGTGGTGTGGACCTCCCAGTCCTGTCTGCCCTCAGGAAGTCCTCCTT

TGCTTCAAAGAACCCTGTTAGCCCCACAACACTCCAGAAAAGCCACACTGCCTCAAAAAA

GACTGACCAACAACGCCAGCCCTCAGGAAATATTCCACGGCAGTGACAAGGCAAACACTG

CCCGGTCACGGAGAGTACAACTCCCTCAGGAAAAAGAAAACAACAAGCAAGATGAAGAAG

CTGAGAAACCACCCCCAGTCAAACCAACAGGAGAACTCACCTAAAGCAGTCAACAATGAA

ACAGATCTCTGCAGTCTGACAGACCTGGAGTTCAAAAGAGAAATAGTGAAAATACTGAAG

GAATTAAGAGAAGATATGAACAGTAATGCAGATACCCTCAGAAAGGAACTAGAAAATATA

AGGAGGAGCCAAGAAAAACTAGAACATTCATTTGCAGAGATACAAACTGAACTAAGGGCA

GTAAAAACCAGAATGAATAATGCAGAAGAACGAATCAGTGATATGGAAGATAGAATAATG

GAAATCACCCAATCAGGTCAGCAGACAGAAAACCAAATCAAAAAACAGGAAAGCAATATA

AGAGACCTATGGGATAATATAAAGCGGGCCAATCTACACATAATAGGAATTCCAGAAGGA

GTAGAAAAAGATAAGGGAATGGAAAATATATTTGAAGAAATTATCGCTGGAAACTTCCCA

AATCTAAAGGATACTGGTTCAAGATACAGGAAGCACAGAGGGCCCCAAACAAGTTGAACC

CAAATAGACCCACACCAAGACACATCATAATAAAAATGGCAAAAGTTAGTGATAAAGAGA

GGATCCTAAAGGCAGCAAGAGAAAAGCAGAATGTTACCTACAAGGGAACCCCCATAAGAT

TATCAGCTGATTTCTCTACAGAAACACTACAGGCCAGGAGGGAATGGCAAGAGATATTTA

AAGTGCTAAAAGGAAAAAATATGCAACCTAGAATACTCTATCCAGCAAGAATATCATTTA

AAATAGAAGGGGAAATAAAATTTTTTCCAACAAACAAAAACTAAAAGAATACAGCAACAC

AAAACCCAGGCTAAAAGAAATACTGAAAGGGCTTCTCTAAACCAAAAAGAAAGGAAGGAA

AGAAAGGGAGGAAAAAGAAAAAAAAAAAAAAAAGAAGAGGAAGAACTAGGACTGAGGAAA

CCACAATCAGAGAGCAGTCACTCAAATAAGCCAGCATACAGATTTAATCATGAACATGCT

TCAAACAAAATAAAATTAAAAAGAAAAAAAAAAGAGTCATCAAAATCATAAAATGTGGGC

AAGGGATGTTAGGAAATAAATAGACCCTTTTTGTTTGTTTGTATGTTTCTCTTCTTAATT

TTAATATAGTAATGAAGTGTTTGAACCTACAGGACCATCAGGCTAAAACACACAATTATA

GGAAGGGGTTAGCATACTTAAAAAACAGGGCAACCACAAGCCAAAACCAAACATTGCATT

TGCAAAAAATGAAAAAAAAAAAAACACTCAAGCAGATAATAACGGAGACCATCCAACCAA

AAAAAAAAAAGAAAGAATGGAGAACCATAGAATCAACTGGAACACGAGGTTCAAAATGGC

AATAAATAATCATCTATCAATTATCACCTTAAATGTCAATGGACTGAATGCCCCAATCAA

AAGACACAGAGTGGCTGAGTGGATAAAAAGGCAAAAACCTTCAATATGCTGCCTACAAGA

AACTCACCTTAGGACAAAGGATACATATAGATTGAAAGTGAAGGGGTGGGGAAAAATATT

TCATGCCAATAGACATGACAGAAAAGCAGGAGTTGCAATACTCATATCAGACAAAATAGA

CTTTAAAACAAAAGACATAAAGAAAGACAAAGAAGGACACTACTTAATGATTAAGGGATC

CATCCAAGGAGAGGATGTTACTATCATCAACATATATGCCCCAAATACAGGAGCACCCAG

ATACATACAACAAATATTAACAGACATAAAGGGAGAAATTGATGGAATACAATCATAGTA

GGAGACTTTAATACCCCCCTCACATCAATGGACAGATCCTCTAGACAGAAAACCAATAAA

GCAACAGAGATCCTAAAGGAAACAATAGAAAAGTTAGACTTAATTGATATCTTCAGGACA

CTACATCCAAAAAAATCAGAATACACATTCTTCTCAAATGCTCATGGAACATTCTCAAGA

ATCGACCACATATTGGGACACAAAGCTAACCTCAATAAATTTAGGAGCATAGAAATTATC

TCAAGTATCTTCTCTGACCACAATGCCATGAAATTAGAAATCAACCATGGGAAAAGAAAT

GAGAAAAAACCTACTACATGGAGACTAAACAACATGCTACTAAAAAACCAATGGGTCAAT

GAGGAAATCAAGAAGGAAATTAAAAAATACCTTGAAACAAATGATAATGAAGACACAACC

TCTCAAAATCTATGGGATGCTGCGAAAGCAGTGCTCAGAGGGAAATTTATAGCAATACAG

GCCTTTCTCAAAAAAGAAGAAAGATCCCAAATTGACAACTTAACCCTCCACCTAAACGAA

TTAGAAAAAGAAGAACAAAAAAGACCTAAAGTCAGCAGAAGGAAGGAAATTATAAAGATC

AAAGAAGAAATCAATAAAATAGAGACTCAAAAAACAATAGAGAAAATTAATAAAACCAAG

AGCTGGTTCTTTGAAAAGGTAAACAAAATTGACAAACCCCTGGCCAGACTCACTAAAAAG

AGGAGAGAAAGAACCCAAATAACCAAAATTATAAATGAAAAAGGAGAAATCACAACGGAT

ACTGCAGAAATACAAAAAACCATAAGAGAATACTATGAACAACTATATGGCAACAAGTTT

GACAATCTGGAAGAAATGGACAATTTTCTAGAATCTTACAGCCTGCCAAAACTGAATCAA

GAAGAAACAGACCAACTGAACAGACCGATCACTAGAAATGAAATTGAAGAGGTCATAAAA

ACACTCCCTACAAATAAAAGTCCAGGACCAGATGGCTTCACAGGCGAATTCTATCAAACA

TATAAAGAGGAATTGGTGCCCATCCTCCTTAAACTCTTTCAAAAGGTTGAAGAAGAAGGA

ATACTCCCAAAGACATTCTATGATGCCACCATCACCCTCATTCCAAAACCAGACAGAGAT

ACCACCAAAAAAGAAAACTATCGGCCAATATCATTGATGAATATAGATGCAAAAATTCTC

AACAAAATCTTAGCCAACCGAATCCAACAACATATCAAAAAAATTATACACCATGACCAG

GTTGGGTTCATCCCAGGTTCACAAGGATGGTTCAACATACGCAAATCAATCAACATCATA

CACCACATTAACAAAAAAAAGTCAAAAATCATATGATCATCTCAATAGAGCAGAAAAAGC

ATTTGACAAAGTCCAACATCCATTCATGATCAAGACCCTCACCAAAGTGGGTATAGAGGG

AACATTCCTGAACATAATCAAAGCCATTTATGATAAACCCACAGCAAATATAATACTCAA

TGGGGAAAAACTGAAAGCCTTCTCACTCAAATCTGGAACAAGACAGGGATGCCCACTCTC

ACCACTGCTCTTCAACATAGTTTTGGAAGTCCTAGCCAAGCAATTAGACAAACAAAAGAA

ATAAAAGGCATCCATATAGGAAGAAAGAGATAAAACTGTCACTGTATGCAGATGACATGA

TACTATACATAGAAAACCCTAAGGACTCAACCCAAAAACTCTTGAACTGATTAATAAATT

CAGCAAAGTAGCAGGATATAAGATTAACATTCAGAAGTCAGTGCATTTCTGTATACCAGC

AATGAAATATTAGAAAAGGAATACAAAAATAGATACCTTTTAAAATTGCACCTCACAAAA

TCAAATACCTCGAATAACCTGACCAAGGAGGTAAAGGACCTATATGCGAGAACTATAAAA

CTTTAATCAAAGAAATCAAAGAAGATGTAAAGAAATGGAAAGATATTCCATGTTCCTGGA

TTGGAAAATCAATATTGTAAAAATGGCCATACTACCCAAAGCAATCTACAGATTCAATGC

AATCCCTATCAAATTACCCATGACATTTTTCACAGAACTAGAACAAACAATCCAAACATT

TATATGGAACAACAAAAGACCCAGAATCGCCAAAGCAATCCTGAGAAACAAAAACCAAGC

AGGAGGCATAACTCTCCCAGACTTCAAAAATATACAAAGCCACAGTCATCAAAACAGTTG

ACTGGTATCAAAAGACAGACAGACCAATGGACAGAATAGAGAACCCGAAATAAACCCGAC

ACCTATGGTCAATTAATCTTTGACAAGGGAGGCAAAACATAAAATGGGAAAAAGAAAGTC

TATTCGCAAGCATTGCTGGAAACCTGGACAGCGCATGCAAAGCAATGAATAGCACCCCCA

CACCATGCACAAAAATAAACTCAAATGGCTGAAGACTTAAATATAGACAGACACCATCAA

CTCTAGAAGAAACATAGGCAAAACACTCTCTGACATCAACATCATGAATATTTTCTCAGG

TCAGTCTCCCAAAGCAATAGAAATTAGAGCAAAAATAAACCCATGGGACCTAATCAAACT

GAAAAGCTTTTGCACAGCAAAGGAAACCAAAAGAAAACAAAAAGACAACTTCAGAATGGG

AGAAAATAGTTTCAAATGATGCAACGACAAGGGCTTAATCTCTAGAATATATAACAACTT

ATACAACTCAACAGCAAAAAAGCCAATCAACCAATGGAAAAATGGGCAAAAGACCTGAAT

AGACATTTCTCCAAGGAAGATATACAGATGGCCACAAACACATGAAAAAATGCTCAACAT

CGCTGATTATAAGAGAAATGCAAATCAAAACTACCATGAGATACCACCTCACACCAGTCA

GAATGGCCATCATTAATAATCCACAAATAACAAGTGCTGGAGGGGTGTGGAGAAAAGGGA

ACCCTCCTGCACTGTTGGTGGGAATGTAAACTGGTACAGCCACTATGGAGAACAGTTTGG

AGATACCTTAGAAATCTATACATAGAACTTCCATATGACCCCCAATCCCACTCTTGGGCA

TTATCCGACAAAACTCTACTTAAAAGAGACACATGCACCGCATGTTCATTGCAGCACTAT

TCACAATAGCCAGGACATGGAAACAACCCAAATGTCCATCGACAGATGATTGGATTGGAA

GAGTGGTATATATACACAATGGAATACTACTCAGCCATAAAAAAGAATGACATAATGCCA

TTTGCAGCAACAGGATGGAACTAGAGAATCTCATACTGAGTGAAATGAGCCAGAAAGACA

AAGACAAATACCATATGATATCACTTATAACTGGAATCTAATATCCAGCACAAATGAACA

TCTCCTCAGAAAAGAAAATCATGGACTTGGAGAAAGACTTGTGGCTGCCTGATGGGAGAG

GGAGGGAGTGGGAGGGATGGGAGCTTGGGTTATCAGACACAACTTAGAATAGATTTACAA

GGAGATCCTGCTGATAGCATTGAGAACTTGTCTAGATACTCATTTGCAACAGAAAAAGGT

GGGGAAAATGTATACATGTAAGGATAACTTGATCCCCTTGCTGTACAGTGGGAAAAAAAA

AAAAAAAAA

>L1C9#LINE/L1C

GGAGGCAAGATGGCGGAAGAGTAGGGGGACACGCTCGCCCTCTCCCACAAACACACAAAA

AAACACATCTACAGGTTAAACGACTCACACAGAACAGCAATAATCGCTGGCAGAAGAACC

TAAACTCCAATAATGGCAAGAATCTCGTGACATAACTGGGTAAAACAAGAGAAAAGAGGA

GAGTGAGAGAAGGGGAATCGGGCTGGACGGCGCTCCCGAAAGGGAACTGTGGAGGAGAAA

GGGATCCCACACCCTGGAAAGTCACCTACTCGATGGAAAGATCAACCGAATCGGAGGGAT

CTCCAGATGCAGAGAAGAGTGCAGCAGTAAGTCTGAGTTCTGAAAAGCAGAGTGAGAACC

GAACAGATCATCTGAACTACTGGCACAGTCACCAAAAACTGAGACGCTTGGGTGGGGGCT

GGGCACCGAGACCTCGGCTCCGGAGGTTAGTCCCGGGAAGGGCTGGGGTGGGGGGTGACT

GTGGGGGGGCTAGGAACGTCTGTCGGTTGCGGGCAGAGACTGCCTGGGAGACTAGAAAGC

AGAGCTCGCGGGTGGAGGGAGCAATACGCTAAGGGCTGGGGAGTGGAAAGCCACATCAGA

GGGAACCTGGGAGAAGAGCTGGATCTGCAGGAGAGACAAGGGCCAGTGTTGGGGAGGGGA

GAGAGAAGGGGTGGGCCACCATAGAATACTCCCCACACCACAGGAGCTCACTTGCCCCCA

GCTATCAGAAAGCTGTGCTTCCCAGTGCATCCCCCCTCCCCCTACCCCACCACGTGCCGG

ACCTGAGGCTGCCTGCCATCCCGGAGGGCTGGCCTCACCATTGCGGAAGCCACCACCCAG

GGGCTTTCCCTGCCCTGGCCTGCCTGCCCTCTGGAGGGGCTACACCCCCGCGAGCAGCAC

CCAGCACTGCCAGCCCCCTGGAAAAGGCCTGCAGCCCAGAAAAGCTAGAACAAGCTTGGC

CGGCCTGAAAATCTGCCTCCATTCTAGGCAGTCCTGCCAATTCGGCTGCCCTGGGGAAGA

GCCCCTTGGGTTCTCAGTGGCCCAGCTAGCCGCTCCCACCCTTGGGGGGTGCTGCACTCC

CGTGGAACAGCTGCCCAGCACTGCCAGCCCCCTGCAAGAGCCCCGCAGCCCAGAAAAGCT

AGAACAAGCTCGCCGGCCATGAAAATCTGCCTCCATTCAGGCAGTCCTCCAATTCGGCTG

CCCTGGGGAAGAGCCCCTTGGGTTCTCAGTGGCCCAGCTAGCTCTCCGCCCTCGGGGGGT

GCTGCACTCCGTGGAACAGCTGCCCAGCACCGCCAGCCCCCTGGAAGAGCCCCACAGCCC

AAAAACACCAGAGCAAGCTCTGCCCGCCAAGTGAAATCTGCTTCCATCTGGTGCAGACCT

CCCAGTCCTGTCTGCCCTCAGGAAGTCCTCCTTTGCTTCAGAGAGACCCTGTCAGCCCCG

CCCACCCTCGGAAAAGCCACGCTGCCTCAAAGAAGACTGACCAACAACGCCAGCCCTCAG

GAAACATTCCACAGCAGTGCCAAGGCAAACCCTGCCCGGCCACGGGGAGTACAACTCCAC

CAAGAAAAAGAAAACAACAAGCAAGATGAAGAAGCTGAGAAACCACCCCCAGTTAAACCA

ACAGGAGAACTCACCTAAAGCAGTCAACAATGAAACAGACTCTGCAGTCTGACAGACTTG

GAGTTCAAAAGGGAAATAGTGAAAATACTGAAGGAATTAAGAGAAGATATGAACAGTAAT

GCAGACACCCTCAGAAAGGAACTAGAAAATATAAGGAGGAGCCAAGAAAAACTAGAAAAT

TCATTTGCAGAGATACAAACTGAACTAAGGGCAGTAAAAACCAGAATGAATAATGCAGAA

GAACGAATTAGTGATGTGGAAGATAGAATAATGGAAATCACCCAATCAGGACAGCAGACA

GAAAACCAAATGAAAAAACATGAAAGCAATATAAGAGACCTATGGGATAATATAAAGCGG

CCAATCTACGCATAATAGGAATTCCAGAAGGAGTAGAAAAAGATAAGGGGATGGAAAATA

TATTTGAAGAAATTATCACTGGAAACTTCCCAAATCTAAAGGATACTGAGTTCAAGATAC

AGGAAGCACAGAGGGCCCCAAACAAGTTGAACCCAAATAGACCCACACCAAGACACATTA

TAATAAAAATGGCAAAAGTTAGTGATAAAGAGAGGATCCTAAAGGCAGCAAGAGAAAAGC

AGAATGTTACCTATAAGGGAACCCCCATAAGGTTATCAGCTGATTTCTCTACAGAAACTC

TACAGGCCAGGAGGGAATGGCAAGAGATATTTAAAGTGCTAAAAGGAAAAAATATGCAAC

CTAGAATACTCTATCCAGCAAGAATATCATTTAAAATAGAAGGGGAAATAAAATTTTTTT

CCAACAAACAAAAGCTAAAAGAATACAGCAATACAAAACCCAGGCTAAAAGAAATATTGA

AAGGGCTTCTCTAAACCAAAAAGAAAGGAAGGAAAAAAAAAAAAAAAAAAAAGAGGAAGA

ACTAGGATTGAGGAAACCACAATCAGAGAGCAGTCACTCAAATAAGCCAGCATACAGATT

TAATCATGAACATGTTTCAAACAAAATAAAATTAAAAAGAAAAAAAGAGTCATCAAAATC

ATAAAATGTGGGCAAGGGAAGTAAGGAAATAAATAGACTCTTTTTTTTTTTTCTCTTCTT

AATTTTAGTATAGTAATGAAGTGTTTGAACCTACAGGACCATCAGGCTAAAACACACAAT

TATAGGAAGGGGTTAGCATACTTAAAAAACAGGGCAACCACAAACCAAAACCAAACATTG

CATTCGCAAAAAAAAAAAAAAAAACACTCAAGCAGAAAATAATCGGAGACCATCCAACCA

AAAAAAGAAAGGAAGAATGGAGAACCATAGAATCAACTGGAACACGAGGTTTAAAATGGC

AATAAATAATCATCTATCAATTATCACCTTAAATGTCAATGGACTGAATGCTCCAATCAA

AAGACACAGAGTGGCTGAGTGGATAAAAAGGCAAAAACCTTCAATTGCTGCCTACAAGAA

ACTCACCTTAGGACAAAGGACACATATAGATTGAAAGTGAAGGGGTGGGAAAAAATATTT

CATGCCAATAGACATGACAGGAAAGCAGGAGTTGCAATACTCATATCAGACAAAATAGAC

TTTAAAACAAAAGACATAAAGAAAGACAAAGAAGGACACTATTTAATGATTAAGGGATCC

ATCCAAGGAGAGGATGTTACTATCGTCAACATATATGCCCCAAATACAGGAGCACCCAGA

TACATACAACAAATATTAACAGACATAAAGGGAGAAATTGATGGGAATACAATCATAGTA

GGAGACTTTAATACCCCACTCACACCAATGGACAGATCCTCTAGACAGAAAACCAATAAA

GCAACAGAGATCCTAAAGGAAACAATAGAAAAGTTAGACTTAATTGACATCTTCAGGACA

CTACATCCAAAAAAATCAGAATACACATTCTTCTCAAGTGCACATGGAACATTCTCAAGA

ATCGACCACATATTGGGACACAAAGCTAACCTCAACAAATTTAGGAGCATAGAAATTATT

TCAAGTATCTTCTCTGACCACAATGCCATGAAACTAGAAATCAACCACAGGAAAAGAAAT

GAGAAAAAACCTACTACATGGAGACTAAACAACATGCTACTAAAAAACCAATGGGTCAAT

GAGGAAATCAAGAAGGAAATTAAAAAATACCTTGAGACAAATGATAATGAAGACACAACC

TCTCAAAATCTATGGGATGCTGCAAAAGCAGTGCTCAGAGGGAAATTCATAGCAATACAG

GCCTTTCTCAAAAAAGAAGAAAGATCTCAAATTGACAACTTAACCCTCCACCTAAACGAA

TTAGAAAAAGAAGAACAAAAAAGACCTAAAGTCAGCAGAAGGAAGGAAATTATAAAGATC

AAAGAGGAAATCAATAAAATAGAGATTCAAAAAACAATAGAGAAAATTAATAAAACCAAG

AGCTGGTTCTTTGAAAAGGTAAACAAAATTGACAAACCCCTGGCTAGACTCACTAAGAAG

AGGAGAGAAAGAACCCAAATAAACAAAATTAGAAATGAAAAAGGAGAAATCACAACGGAT

ACTGCAGAAATACAAAAAACCATAAGAGAATACTATGAACAACTATATGCCAACAAATTT

GACAATCTGGAAGAAATGGACAATTTTCTAGAATCTTACAGCCTGCCAAAACTGAATCAA

GAAGAAACAGACCAACTGAACAGACCGATCACTAGAAATGAAATTGAAGAGTCATAAAAA

CACTCCCTACAAATAAAAGTCCAGGACCAGATGGCTTCACAGGCGAATTCTACCAAACAT

ATAAAGAGGATCTGGTGCCCATCCTCCTTAAACTTTTTCAAAAGGTTGAAGAAGAAGGAA

CACTCCCAAAGACATTCTATGATGCCACCATCACCCTAATTCCAAAACCAGACAAAGATA

CCACCAAAAAAGAAAACTATCGGCCAATATCTTTGATGAATATAGATGCAAAAATTCTCA

ACAAAATCTTAGCCAACCGAATCCAACAACATATCAAAAAGATCATACACCATGACCAGG

TAGGGTTCATCCCAGGTTCACAAGGATGGTTCAACATATGCAAATCAATCAACGTCATAC

ACCACATTAACAAAAGAAAAGTCAAAAACCATATGATCATCTCAATAGATGCAGAAAAAG

CATTTGACAAAGTCCAACATCCATTCATGATCAAGACCTCGCCAAAGTGGGTATAGAGGG

AACATTCCTGAACATAATCAAAGCCATTTATGACAAACCCACAGCAAATATAATACTCAA

TGGAGAAAAACTGAAAGCCTTCTCACTCAAATCTGGAACAAGACAGGGATGCCCACTCTC

ACCACTGCTCTTCAACATAGTTTTGGAAGTCCTAGCCACAGCAATTAGACAAACAAAAGA

AATAAAAGGCATCCATATAGGAAGAGAAGAGATAAAACTGTCACTGTATGCAGATGACAT

GATACTATACATAGAAAACCCTAAGGACTCAACCCAAAAACTACTTGAACTGATTAATAA

ATTCAGCAAAGTAGCAGGATATAAGATTAACATTCAGAAATCAGTCGCATTTCTGTATAC

CAACAATGAAATATTAGAAAAGGAATACAAAAATACAATACCTTTTAAAATTGCACCTCA

AAAAATCAAATACCTCGGAATACACCTGACCAAGGAGGTAAAGGACTTATATGCCGAGAA

CTATAAAACTTTAATCAAAGAAATTAAAGAAGATGTAAAGAAATGGAAAGATATTCCATG

TTCCTGGATTGGAAAAATCAATATTGTAAAAATGGCCATACTACCCAAAGCAATCTACAG

ATTCAATGCAATCCCTATCAAATTACCCATGACATTTTTCACAGAACTAGAACAAACAAT

CCAAACATTTATATGGAACCACAAAAGACCCAGAATTGCCAAAGCAATCCTGAGAAACAA

AAACCAAGCAGGAGGCATAACTCTCCCAGACTTCAAGAAATATTACAAAGCCACAGTCAT

CAAAACAGTGTGGTACTGGTATCAAAACAGACAGACAGACCAATGGAACAGAATAGAGAA

CCCAGAAATAAACCCTGACACCTATGGTCAATTAATCTTTGACAAGGGAGGCAAGAACAT

AAAATGGGAAAAAGAAAGTCTATTCAGCAAGCATTGCTGGGAAACCTGGACAGCTGCATG

CAAAGCAATGAAACTAGAACACACCCTCACACCATGCACAAAAATAAACTCAAAATGGCT

GAAAGACTTAAATATAAGACAGACACCATCAAACTCCTAGAAGAGAACATAGGCAAAACA

CTCTCTGACATCAACATCATGAATATTTTCTCAGGTCAGTCTCCCAAAGCAACAGAAATA

AGAGCAAAAATAAACCATGGGACCTATCAAACTGAAAAGCTTTTGCACAGCAAAGGAAAC

CAAAAGAAAACAAAAAGACAACTTACAGAATGGGAGAAAATAGTTTCAAATGATGCAACG

ACAAGGGCTTAATCTCTAGAATATAAAGCAACTTATACAACTCAACAGCAAAAAAGCCAA

TCAACCAATGGAAAAATGGGCAAAAGACCTGAATAGACTTCTCCAAGGAAGATATACAGA

TGGCCAACAAACACATGAAAAAATGCTCAACATCCTGATTATAAGAGAAATGCAAATCAA

AACTACCATGAGATACCACCTCACACCAGTCAGAATGGCCATCATTAATAAGTCCACAAA

TAACAAGTGCTGGAGGGGTGTGGAGAAAAGGGAACCCTCCTGCACTGTGGTGGGAATGTA

AACTGGTACAGCCACTATGGAGAACAGTTTGGAGATACCTTAGAAATCTATACATAGAAC

TTCCATATGACCCGCAATCCCACTCTTGGGCATATATCCGGACAAAACTCTACTTAAAAG

AGACACATGCACCCGCATGTTCATTGCAGCACTATTCACAATAGCCAGACATGGAAACAA

CCCAAATGTCCATCACAGATGATTGGATTGGAAGATGTGGTATATATACACAATGGAATA

CTACTCAGCCATAAAAAAATGACATAATGCCATTTGCAGCAACATGGATGGAACTAGAGA

CTCTCATACTGAGTGAAATGAGTCAGAAAGACAAAGACAAATACCATATGATATCACTTA

TAACTGGAATCTAATATCAGCACAAATGAACATTTCCACAGAAAAGAAAATCATGGACTT

GGAGAATAGACTTGTGGCTGCCGGGGGAGAGGGAGGGAGTGGGAGGGATGGGAGCTTGGG

GTTATGATACAACTTGGAATGGATTTACAAGAGATCCTGCTGAGTAGCATTGAGAACTAT

GTCTAGATACTTATATTGCAACAGAACAAAGGGTGGGAAAAAATGTATACATGTAAGGTA

ACTTGGTCCCCATGCTGTACAGTGGAAAAAAAAAAAAAAAAAAAAAAA

>L1C10#LINE/L1C

GGGAGGAGCAAGATGGCGGAAGAGTAGGGGGACATGCTCGCCCTCTCCCACAAACACAAC

AACAAAAAAACACATCTACAGGTTAAATGACTCACACAGAACAGCAATAATCGCTGGCAG

AAGAACCTAAACTCCAATAATGGCAAGAATCTCGTGACATAACTGGGTAAAACAAGAGAA

AAGAGGAGAGTGAGAGAAGGGGAATCGAGCTGGACGGGGCTCCTGAAAGGGAACTGTGGA

GGAGAAAGGGATCCCACACCCTGGAAAGTCACCTACTCGAGGAAAGATCAACCAATCGGA

GGGATCTCCAGATGCAGAGAAGAGTGCAGCAGTAAGTCGAGTTCTGAAAAGCAGAGCGAG

AACCAAACAGATCATCTGAACTACTGGCACAGTCACCAAAAATTGAGACGCTTGGGTGGG

GGCTGGGCACCGAGACCTCGGCTCTGGAGGTTAGTCCCCGGGAACGTGGGGGCGGGGGGA

GACTGCTTGGGGGGTCTAGGAACCGTCTGTCAGTTTGACAGGGCAGAGACTGCCTGGGAG

ACTAGAAAGCAGAGCGTCACGGGGGGAGGGAGCAATACCTAAGGGCTGGGAAGTGGAAAG

CCACATCAGAGGGAACCTGGGAGAAGAGCTGGATCTGCAGGAGAGACAAGGTGCCAGTGT

TGGGGAGGGGAGAGAAGAGGGGTGGGCCCCATAGAATACTCCCCACCCACAGGAGCTCAC

TGCCCACCAGCTAGCAGAAAGCTGTGCTTCCCAGTGCATCCCCCCCCCCTACCCCTACCA

CCGCCGGACCTGGGCTGCCTGCCATCCGGAGGGCTGGCCTCAACAATTGCCGAAGCCTAC

CACGCAGGGGCTTTCCCTGCCCTGGCCTGCTTGCCCTCTGGAGGGGCTACACCCCCGCAG

AGCAGCACCAAACACCACCAGCCCCCGGAAAAGGCCTGCAGCCCAGAAAAGCTAGAACAA

GCTTGCCGGCCGTGAAAAATCTGCCTATTCTCGGCAGTCCTTCCAAGTCGGCTGCCCTGG

GGAAGAGCCCCTTGGGTTCCAGCGGCCCAGCTAGCTCTCCAGCCCTCAGGGGGTGCCACT

CCGGAACAGCTGCCAGCACCCAGCCCTGCAAGACCCGCCCAGAAAAGCTAAAAGCTGCCA

CTGAAAAATCTGCCTCATTTGGCGTCCTCTGAGTGGCTGCCCTGGGAAGAGCCCTTGGGT

TCTCAGGCCCAGTAGCTCTCCAGCCCCGGGGGTGCTGCACTCCTGTGGAACAGCTGCCCA

GCACCACCAGCCCCCTGCAAGAGCCCCACAGCCCAAAAACACCAGAGCAAGCTCTGCCTG

GCCAGTGAAATCTGCTTCCATCTGGTGTGGACCTCCCAGTCCTGTCTGCCCTCAGGAAGT

CCTCCTTTGCTTCAGAGAGACCCTGTCAGCCCCACCCACCCTCAGAAAAGCCACACTGCC

TCAAAGAAGACTGACCAACAATGCCAGCCCTCAGGAAACATTCCACAGCAGTGCCAAGGC

AAACCCTGCCGGCCACGAGAGTACAACTCCACCAGAAAAAGAAAACAACAAGCAAGATGA

AGAAGCTGAGAAACCACCCCCAGTTAAACCAACAGGAGAACTCACCTAAAGCAGTCAACA

ATGAAACAGACTCTGCAGTCTGACAGACTGGAGTTCAAAAGGGAAATAGTGAAAATACTG

AAGGAATTAAGAGAAGATATGAACAGTAATGCAGATACCCTCAGAAAGGAACTAGAAAAT

ATAAGGAGGAGCCAAGAAAAACTAGAACATTCATTTGCAGAGATACAAACTGAACTAAGG

GCAGTAAAAACCAGAATGAATAATGCAGAAGAACGAATAGTGATGTGGAAGATAGAATAA

TGGAAATCACCCAATCAGGCAGCAGACAGAAAACCAAATAAAAAACATGAAAGCAATATA

AGAGACCTATGGGATAATATAAAGCGGCCAATCTACACATAATAGGAATTCCAGAAGGAG

AGAAAAAGATAAGGGGATGGAAAATATATTTGAAGAAATTATCACTGGAAACTTCCCAAA

TCTAAAGGATACTGATTCAAGATACAGGAAGCACAGAGGGCCCCAAACAAGTTGAACCCA

AATAGACCCACACCAAGACACATTATAATAAAAATGGCAAAAGTTAGTGATAAAGAGAGG

ATCCTAAAGGCAGCAAGAGAAAAGCAAATGTTACCTAAAGGGAACCCCCATAAGGTTATC

AGCTGATTTCTCTACAGAAACTCTACAGGCCAGGAGGGAATGGCAAGAGATATTTAAAGT

GCTAAAAGGAAAAAATATGCAACCTAGAATACTCTATCCAGCAAGAATATCATTTAAAAT

AGAAGGGGAAATAAAATTTTTTCCAACAAACAAAACTAAAAGAATACAGCAATACAAAAC

CCAGGCTAAAAGAAATATTGAAAGGGCTTCTCTAAACCAAAAAGAAAGAAGAATGAGGAA

ACCACATCAGAAGCATCACTCAATAAGCAGCATACAGATTTATATGAACATGTTTCAAAC

AAAATAAATAAAAGAAAAAAAAGCATCAAAATCATAAAATGTGGGCAAGGGAGTAGGAAA

TAATAATTTTTTTTTTTTTTTTCTCTTCTTAATTTTAGTATAGTAATGAAGTGTTTGAAC

CTAAGGACCATCAGGCTAAAACACACAATTATAGGAAGGGGTTAGCATACTTAAAAAACA

GGGCAACCACAAACAAAACCAAAATTGCATTTCAAAAAATGAAAAAAAAAAAAAAAAACA

AACAAAAAAAAGAAAGGAAGAATGGAGAACCATAGAATCAACTGGAACACGAGGTTTAAA

ATGGCAATAAATAATCATCTATCAATTATCACCTTAAATGTCAATGGACTGAATGCCCCA

ATCAAAAGACACAGAGTGGCTGAGTGGATAAAAAGGCAAAAACCTTCAATATGCTGCCTA

CAAGAAACTCACCTTAGGACAAAGGATACATATAGATTGAAAGTGAAAGGGTGGGAAAAA

TATTTCATGCCAATAGACATGACAGGAAAGCAGGAGTTGCAATACTCATATCAGACAAAA

TAGACTTTAAAACAAAAGACATAAAGAAAGACAAAGAAGGACACTATTTAATGATTAAGG

GATCCATCCAAGAAGAGGATATTACTATCGTCAACATATATGCCCCAAATATAGGAGCAC

CCAGATACATACAACAAATATTAACAGACATAAAGGGAGAAATTGATGGGAATACAATCA

TAGTAGGAGACTTTAATACCCCCTCACATCAATGGACAGATCCTCTAGACAGAAAACCAA

TAAAGCAACAGAGATCCTAAAGGAAACAATAGAAAAGTTAGACTTAATTGACATCTTCAG

GACACTACATCCAAAAAAATCAGAATACACATTCTTCTCAAGTGCCATGGAACATTCTCA

AGAATCGACCACATATTGGGACACAAAGCTAACCTCAACAAATTTAGGAGCATAGAAATT

ATTTCAAGTATCTTCTCTGACCACAATGCCATGAAATTAGAAATCAACCATGGGAAAAGA

AAAGAGAAAAAACCTACTACATGGAGACTAAACAACATGCTACTAAAAAACCAATGGGTC

AATGAGGAAATCAAGAAGGAAATTAAAAACTACCTTGAGACAAATGATAATGAAGACACA

ACCTCTCAAAATCTATGGGATGCTGCAAAAGCAGTGCTCAGAGGGAAATTTATAGCAATC

AGGCCTTTCTCAAAAAAGAAGAAAGATCCAAATTGACAACTTAACCCTCCACCTAAACGA

ATTAGAAAAAGAAGAACAAAAAAGACCTAAAGTCAGCAGAAGGAAGGAAATTATAAAGAT

CAAAGAAGAAATCAATAAAATAGAGATTCAAAAAACAATAGAGAAAATTAATAAAACCAA

GAGCTGGTTCTTTGAAAAGGTAAACAAAATTGACAAACCCCTGGCTAGACTCACTAAAAG

AGGAGAGAAAGAACCCAAATAAACAAAATTAGAAATGAAAAAGGAGAAATCACAATGGAT

ACTGCAGAAATACAAAAAACCATAAGAGAATACTATGAACAACTATATGCAACAAGTTTG

ACAATCTGGAAGAAATGGACAATTTTCTAGAATCTTACAGCCTGCCAAAACTGAATCAAG

AAGAAACAGACCAACTGAACAGACCATCACTAGAAATGAAATTGAAGAGTCATAAAAACA

CTCCCTACAAATAAAAGTCCAGGACCAGATGGCTTCACAGGTGAATTCTATCAAACATAT

AAAGAGGAATTGGTGCCCATCCTCCTTAAACTCTTTCAAAAGGTTGAAGAAGAAGGAACA

CTCCCAAAGACATTCTATGAGCCACCATCACCCTCATTCCAAAACCAGACAGAGATACCA

CCAAAAAAGAAAACTATCGGCCAATATCTTTGATGAATATAGATGCAAAAATTCTCAACA

AAATCTTAGCCAACCGAATCCAACAACATATCAAAAAGATCATACACCATGACCAGGTAG

GGTTCATCCCAGGTTCACAAGGATGGTTCAACATACGCAAATCAATCACATCATACACCA

CATTAACAAAAAAAAGTCAAAAATCATATGATCATCTCAATAGACGCAGAAAAAGCATTT

GACAAAGTCCAACATCCATTCATGATCAAGACCCTCCCAAAGTGGGTATAGAGGGAACAT

TCCTGAAATAATCAAAGCCATTTATGAAAACCCACAGCAAATATAATACTCAATGGAGAA

AAACTGAAAGCCTTCTCACTCAAATCTGGAACAAGACAGGGATGCCCACTCTCACCACTG

CTCTTCAACATAGTTTTGGAAGTCCTAGCCACAGCAATTAGACAAACAAAAGAAATAAAA

GGCATCCATATAGGAAGAGAAGAGATAAAACTGTCACTGTATGCAGATGACATGATACTA

TACATAGAAAACCCTAAGGACTCAACCCAAAAACTCTTGAACTGATTAATAAATTCAGCA

AAGTAGCAGGATATAAGATTAACATTCAGAAGTCAGTGCATTTCTGTATACCAGCAATGA

AATATTAGAAAAGGAATACAAAAATACATACCTTTTAAAATTGCACCTCACAAAATCAAA

TACCTCGGAATACACCTGACCAAGGAGGTAAAGGACTATATGCCGAGAACTATAAAACTT

TAATCAAAGAAATCAAAGAAGATGTAAAGAAATGGAAAGATATTCCATGTTCCTGGATTG

GAAAAATCAATATTGTAAAAATGGCCATACTACCCAAAGCAATCTACAGATTCAATGCAA

TCCCTATCAAATTACCCATGACATTTTTCACAGAACTAGAACAAACAATCCAAAATTTAT

ATGGAACCACAAAAGACCCAGAATCGCCAAAGCAATCCTGAGAAACAAAAACCAAGCAGG

AGGCATAACTCTCCCAGACTTCAAGAAATACTACAAAGCCACAGTCATCAAAACAGTGTG

GTACTGGTATCAAAACAGACAGACAGACCAATGGAACAGAATAGAGAACCCGGAAATAAA

CCCTGACACCTATGGTCAATTAATCTTTGACAAGGGAGGCAAGAACATAAAATGGGAAAA

AGAAAGTCTATTCAGCAAGCATTGCTGGGAAACCTGGACAGCTGCATGCAAAGCAATGAA

ACTAGAACACACCCTCACACCATGCACAAAAATAAACTCCAAATGGCTGAAAGACTTAAA

TATACGACAGGACACCATCAAACTCCTAGAAGAAAACATAGGCAAAACACTCTCTGACAT

CAACCTCATGAATATTTTCTCAGGTCAGTCTCCCAAAGCAATAGAAATAAGAGCAAAAAT

AAACCCATGGGACCTCATCAAACTGAAAAGCTTTTGCACAGCAAAGGAAACCAAAAGAAA

ACAAAAAGACAACTTACAGAATGGGAGAAAATAGTTTCAAATGATGCAACGACAAGGGCT

TAATCTCTAGAATATATAAGCAACTTATACAACCCAACAGCAAAAAAGCCAATCAATCAA

TGGAAAAATGGGCAAAAGACCTGAATAGACATTTCTCCAAAGAAGATATACAGATGGCCA

ACAAACACATGAAAAAATGCTCAACATCGCTGATTATAAGAGAAATGCAAATCAAAACTA

CCATGAGATACCACCTCACACCAGTCAGAATGGCCATCATTAATAAGTCCACAAATAACA

AGTGCTGGAGGGGCTGTGGAGAAAAGGGAACCCTCCTGCACTGTTGGTGGGAATGTAAAC

TGGTACAGCCACTATGGAGAACAGTTTGGAGATACCTTAGAAATCTATACATAGAACTTC

CATATGACCCCACAATCCCACTCTTGGGCATATATCCGGACAAAACTCTACTTAAAAGAG

ACACATGCACCCGCATGTTCATTGCAGCACTATTCACAATAGCCAGGACATGGAAACAAC

CCAAATGTCCATCGACAGATGATTGGATTAGGAAGAAGTGGTATATATACACAATGGAAT

ACTACTCAGCCATAAAAAAGAATGACATAATGCCATTTGCAGCAACATGGATGGAACTAG

AGAATCTCATACTGAGTGAAATGAGCCAGAAAGACAAAGACAAATACCATATGATATCAC

TTATAACTGGAATCTAATATCCAGCACAAATGAACATCTCCTCAGAAAAGAAAATCATGG

ACTTGGAGAATAGACTTGTGGCTGCCGAGGGAGAGGGAGGGAGTGGGAGGGATCGGGAGC

TTGGGCTTATCAGATACAACTTGGAATAGATTTACAAGGAGATCCTGCTGAGTAGCATTG

AGAACTATGTCTAGATACTATATTGCAACAGAACAAAGGGTGGGGAAAAAAAATGTATAC

ATGTAAGGATAACTTGATCCCCTGCTGTACAGTGGAAAAAAAAATAAAAAAATAAAAAAT

TATTATAATAAATTTAGA

>L1C11#LINE/L1C

GAAGGCAAGATGGTGGAAGAGTAGGGGGATGGCTCACCCTCTCCCACAAACACAAAAAAA

AAAAACACATCTACATGTTAAACGACTCCACAGAACAGCAACTAAACGCTGGCAGAAGAA

CTTAAACCTCCAATAATGGCAAGAATCTCTGACATAACTGGGTAAAACAAGAGAAAAGAG

GAGAGTGAGAGAAGGGGAATCAGGACCGACGGCGCTCCCGAAAGGGAACTGTGAAGGAGA

AAGGGAACCCACACCCTGGAAAGTCACCTAATCGACGGAAAGATCAACCGAGTCGGAGGG

ATCTCCAGACGCCGAGAAGAGCGCAGCAGTAGGTCTGAGATCTGAAAAGCAGAGTGAGAG

CCGAACAGATCATCTGAACTACTGGCACAGTCACCAAAAACCGAGACGCTCGGGTGGGGG

CTGGGCACCGAGACCTGGCTCCAGAGGTTAGTCCCTGGGAGCAGGCTGGGGTTGGTGGTG

TGGAGACAGCCTGAGGGATTAGGAAGCGGTGCGTCGTGGGTGGAGGGAGCAATACGCTAA

GGGCTGGGGAGTGGAAAGCCACAACAGAGGGAACCTGGGAGAAGACTGGACCCCAGGAGA

GACAAGGCGCCAGTGTTGGGGAGGGGAGAGGAGGAGGGGTGGGCCACCATAGAATACTCC

TTGGCCCCAGGAGCAGCTTGCCCACCAGCTAGCAGAGAGCAGTGCTTCCCAGTGCATCCC

CCCTCCCCCCCCCACGCACCTGACCTGAGGCCGCCTGCCATCCTGGAGGGCTGGCCTCAC

CACTCGTGGGAAGCCAACCACCTCCGGGGCTTTCCCCGCCCTGGCCTGCCCCCCTCTGGA

GGGGCTACACTCCCACGGAGCAGGCCCAGCACCCCGCCCCCTGGAAGAGCTCCGCAGCCC

AGAAACACCAGGGCAAGCTCTGCCGGCCACGGGAAGTCTGCCTCCATCGCGGGGAGTCCT

GCCAGTTCCAGCTGCCCTGGGGAAGTGCTCCTTTGTCTCCCAGCGGCCCAGCTAGCCCTC

CCGCCCTCGGGAGGTGCTGCACTCCCGCAGAGCAGCTGCCCAGCACCGCCGCCCCCTGGA

AGAGCTCCGCAGCCCAGAAATGCCAGGGCAAGCCCTGCCTGGCCACGGGAAGTCTGCCTC

CATCGTGGTGCAGACCTGCCAGTCCCACCTGCCCTCGGGAAGTCCTCCTTTGCTTCAGAG

TGACCCTGCTAGCCCCGCCCACCCTCGGGAAATGCCCCTGCCTCAAAGAAGACCAGCCAA

CACCGCCAGCCCTCGGGAAACACTCCACAGCAGTGCCAGGGCAAACCCTGCCCGCCACGG

GGAGTGCACCTCCACCAGAAAAAGAAAATAACAAGCAAGATGAAGAAGCTCAGAAACCAT

TCCCAGTTAAACCAACAGGAGAACTCACCTAAAGCAGTCAACAATGAAACAGACTCTGCA

GTCTGACAGACTTGGAGTTCAAAAGGGAGATAGTGAAAATACTGAAGGAATTAAGAGAAG

ATATGAACAGTAATGCAGACTCCCTCAGAAAGGAACTAGAAAATATAAGGAGGAGCCAAG

AAAAACTAGAAAATTCATTTGCAGAGATACAAACTGAGCTAAGGGCAGTAAAAACCAGAA

TGAATAATGCAGAAGAACGAATTAGTGATGTGGAAGATAGAATAATGGAAATCACCCAAA

CAGGACAGCAGACAGAAAACCAAATGAAAAAACATGAAAGCAATATAAGAGACCTATGGG

ATAATATAAAGTGGGCCAATCTATGCATAATAGGAATTCCAGAAGGAGAAGAAAAAGATA

AGGGGATGGAAAATATATTTGAAGAAATTATCCTGGAAACTTCCCAAATCTAAAGGATAC

TGATTTCAAGATACAGGAAGCACAGAGGGCCCCAAACAAGTTGAACCCAAATAGACCCAC

ACCAAGACACATTATAATAAAAATGGCAAAAGTTAATGATAAAGAGAGGATCCTAAAGGC

AGCAAGAGAAAAGCAAAGTGTCACTTATAAGGGAACCCCCATAAGGCTATCAGCTGATTT

CTCTACAGAAACTCTACAGGCCAGGAGGGAATGGCAAGAGATATTTAAAGCGCTGAAAGG

AAAAAATATGCAACCTAGAATACTCTATCCAGCAAGAATATCATTTAAAATAGAAGGGGA

AATAAAAATTTTTTCCAACAAACAAAAGCTAAAAGAATACAGCAATACAAAACCCATTCT

AAAAGAAATATTGAAAGGGCTTCTCTAAATCAAAAAAAAAAAAAAAAGAGGAAGAACTAG

GATGGAGGAAACCACAATCAGAGAGCAGTCACTCAAATAAGCCAGCATACAGATCTAATC

ATGAAGATGTTTAAAACAAAATAAAATTAAAAAGAAAAAAAGAGACATCAAAATCATAAA

ATGTGGGCAAGGGAAGTAAGGAAATAAATAGATTCTTTTTTTAATTTTCTTTTTTTAATT

TTAGTATGGTAATGAAGTGTTTGAACCTACAGGACTATCAGGCTAAAACACACAATTATA

GGAAGGGGTTAACATACTTAAAAAACAGGGCAAGCACAAATCAAAACCAAACATTACATT

CACAAAAAATGAAAAGAAAAATACTCAAGCAGAAAATAATTGGAGACCATCCAACCAAAA

AAAGAAAGGAAGAATGGAGAATCATAGAATCAACTGGAAAACGAGGTTTAAAATGGCAAT

AAATAATCATCTATCAATTATCACCTTAAATGTCAATGGACTGAATGCTCCAATCAAAAG

ACACAGAGTGGCTGATTGGATAAAAAAGCAAAAACCTTCAATCTGCTGCCTACAAGAAAC

TCACCTTAGGACAAAGGACACATATAGATTGAAAGTGAGGGGGTGGGAAAAAATATTTCA

TGCCAATGGACATGACAGGAAAGCAGGAGTTGCAATACTCATATCAGACAAAATAGACTT

TAAAACGAAGGCCATAAAGAAAGACAAAGAAGGACACTATTTAATGATTAAAGGATCCAT

TCAAGAAGAGGATATTACTATCATCAACATATATGCCCCAAATATAGGAGCACCCAGATA

CATACAACAAATATTAACAGACATAAAGGGAGAAATTGATGGGAATACAATCATAGTAGG

AGACTTTAACACCCCACTCACATCAATGGACAGATCCTCTAGACAGAAAACCAATAAAGC

AACAGAGATCCTAAAGGAAACAATAGAAAAGTTAGACTTAATTGACATCTTCAGGACACT

ACATCCAAAAAAATCAGAATACACATTCTTCTCAAGTGCGCATGGAACATTCTCAAGAAT

TGATCACATATTGGGGCACAAAGCTAACCTCAACAAATTTAGGAGCATAGAAATTATTTC

AAGTATCTTCTCTGACCACAATGGTATGAAACTAGAAATCAACCACAGGAAAAGAAATGA

GAAAAAACCTACTACATGGAGACTAAACAACATGCTACTAAAAAACCAATGGGTCAATGA

GGAAATCAAGAAGGAAATTAAAAAATACCTCGAGACAAACGATAATGAAGGCACAACCTC

TCAAAATCTATGGGATGCCGCAAAAGCAGTGCTCAGAGGGAAATTCATAGCAATACAGGC

CTTCCTCAAAAAAGAAGAAAGATCTCAAATTGACAACTTAACCCACCACCTAAATGAATT

AGAAAAAGAAGAACAAAAAAGACCTAAAGTCAGCAGAAGGAAGGAAATCATAAAGATCAA

AGAGGAAATCAATAAAATAGAGATTCAAAAAACAATAGAGAAAATTAATAAAACCAAGAG

CTGGTTCTTTGAAAAGGTAAACAAAATTGACAAACCCCTGGCTAGACTCACTAAGAAGAG

GAGAGAAAGAACCCAAATAAACAAAATTAGAAATGAAAAAGGAGAAATCACAACGGATAC

TGCAGAAATACAAAAAACCATAAGAGAATACTATGAACAACTATATGCCAACAAATTTGA

CAATCTGGAAGAAATGGACAACTTTCTAGAATCTTACAGCCTGCCAAAACTGAATCAAGA

AGAAACAGACCAACTGAACAGACCGATCACTAGAAATGAAATTGAATACGTCATAAAAAC

ACTCCCTACAAATAAAAGCCCAGGACCAGATGGCTTCACAGGGAATTCTACCAAACATAC

AAAGAGGATCTGGTGCCCATCCTCCTTAAACTTTTTCAAAAGGTTGAGGAAGAAGGAACA

CTCCCAAAGACATTCTATGATGCCACCATCACCCTAATTCCAAAACCAGACAAAGATACC

ACCAAAAAAGAAAACTATCAGCCAATATCTTTGATGAATATAGACGCAAAAATTCTCAAC

AAAATTTTAGCCAACCGAATCCAACAACATATCAAAAAGATCGTACACCATGACCAGGTG

GGGTTCATCCCAGGTTCACAAGGATGGTTCAACATATGCAAATCAATCAACATCATACAC

CACATTAACAAAAGAAAAGTCAAAAACCATATGATCATCTCAATAGATGCAGAAAAAGCA

TTTGACAAAGTCCAACATCCATTTATGATAAAAACTCTCACCAAAGTGGGTATAGAGGGA

ACATTCCTGAACATAATCAAAGCCATTTATGACAAACCCACAGCAAATATAATACTCAAT

GGAGAAAAATTGAAAGCCTTCTCACTCAAATCTGGAACAAGACAGGGATGCCCACTCTCA

CCACTGCTATTCAACATAGTTTTGGAAGTCCTAGCCACAGCAATTAGACAAACAAAAGAA

ATAAAAGGCATCCAAATAGGAAGAGAAGAGATAAAACTGTCACTGTATGCAGATGACATG

ATACTATACATAGAAAACCCTAAGGACTCAACCCAAAAACTACTTGAACTGATTAACAAA

TTCAGCAAAGTAGCAGGATATAAGATTAACATTCAGAAATCAGTCGCATTTCTGTATACT

AACAATGAAATATTAGAAAAGGAATACAAAAATACAATACCTTTTAAAATTGCACCCCAA

AAAATCAAATACCTGGGAATACACCTGACCAAGGAGGTAAAGGACTTATATGCTGAGAAC

TATAAAACATTAATCAAGGAAATTAAAGAAGATGTAAAGAAATGGAAAGATATTCCATGT

TCCTGGATTGGAAAAATTAATATTGTAAAAATGGCCATACTACCCAAAGCAATCTACAGA

TTCAATGCAATCCCTATCAAATTACCCATGACATTTTTCACAGAACTAGAACAAACAATC

CAAAAATTTATATGGAACCACAAAAGACCCAGAATTGCCAAAGCAATCCTGAGAAACAAA

AACCAAGCAGGAGGCATAACTCTCCCAGACTTCAAGCAATATTACAAAGCCACAGTCATC

AAAACAGTGTGGTACTGGTACCAAAACAGACAGACAGACCAATGGAACAGAATAGAGAAC

CCAGAAATAAACCCTGACACCTATGGTCAATTAATCTTTGACAAAGGAGGCAAGAGCATA

AAATGGGAAAAAGAAAGTCTATTCAGCAAGAATTGCTGGGAAACCTGGACAGCTGCATGC

AAATCAATGAAACTAGAACACACCCTCACACCATGCACAAAAATAAACTCAAAATGGCTG

AAAGACTTAAATATAAGACAAGACACCATCAAACTCCTGGAAGAGAACATAGGCAAAACA

TTCTCTGACATCAACCTCATGAATATTTTCTCAGGTCATTCTCCCAAGGCAACAGAAATA

AGAGCAAAAATAAACCAATGGGACCTAATCAAACTGACAAGCTTTTGCACAGCAAAGGAA

ACCAAAAAGAAAACAAAAAGACAACTTACAGAATGGGAGAAAATAGTTTCAAATGATGCA

ACGACAAGGGCTTAATCTCTAGAATATACAAGCAACTTATACAACTCAACAGCAAAAAAG

CCAACCACCCAATGGAAAAATGGGCAAAAGACCTGAATAGACATTCTCCAAGGAAGATAT

ACAGATGGCCAACAAGCACATGAAAAAATGCTCAACATCCCTGATTATTAGAGAAATGCA

AATCAAAACTACCATGAGATACCACCTCACACCAGTCAGAATGGCCATCATTAATAAGTC

CACAAATAACAAGTGCTGGAGGGGTGTGGAGAAAAGGGAACCCTCCTGCACTGTTGGTGG

GAATGTAAGCTGGTACAGCCACTATGGAGAACAGTATGGAGGTACCTTAGAAATCTATAC

ATAGAACTACCATATGACCCAGCAATCCCACTCTTGGGCATATATCCGGACAAAACTTTC

CTTAAAAAAGACACATGCACCGCATGTTCATTGCAGCACTATTCACAATAGCCAAGACAT

GGAAACAACCCAAATGTCCATTGACAGATGATTGGATTAGGAAGATGTGGTATATATACA

CAATGGAATACTACTCAGCCATAAAAAAGAATGACATAATGCCATTTGCAGCAACATGGA

TGGAACTAGAGACTCTCATACTGAGTGAAATAAGTCAGAAAGAGAAAGACAAATACCATA

TGATATCACTTATAACTGGAATCTAATATACAGCACAAATGAACCTTTCCACAGAAAAGA

AAATCATGACTTGGAGAATAGACTTGTGGCTGCCGGGGGGAGAGGGAGGGAGTGGGAGGG

ATGGGAGCTTGGGGTTAAGGATGCAAACTATTGCTCTTGGAATGGATTTACAATGAGATC

CTGCTGTGTAGCATTGAGAACTATGTCTAGATACTTACATCGCAACACAACAATGGGAGG

AAAAATATGTATACATGTATGTGTAACTTGGTCCCCATGCTGTACAGTGGAAAAAAAAAA

ATAAAAAAAAAAAAAAAAA

>L1C12#LINE/L1C

GGAGGCAAGATGGCGGAAGAGTAGGGGGACGCGCTCGCCCTCTCCCACAAACACAACAAA

AAAACACATCTACACGTAAAGACTCACACAGAACAGCAACTGAATGCTGGCAGAAGAACT

TAAACCTCCAAAAAGGCAAGAATCTCTTGACATAACTGGGTAAAACAAGAGAAAAGAGGA

GAGAGAGAGAAGGGGAATCAGGACGGACTGGCACTCCTGAGAGGGAACTGTGAAGGAGAA

AGGGAACCCACACCCTGGAAAGTCACCTAACCACGGAAAGATCAACTGAGTTGGAGGGAT

CTCCAGACCGAGAAAAGTGCAGCAGCAGGTCTGAGATCTGAAAAGCAGAGTGAGAGCCAC

ACAGATCATCTGAACCACTGGCACAGACACCAAAAACGAGATGCTTGGGTGGGGGCTGGG

CACCGAGACCTAGGCTCCGAGGTTAGTCCCTGGGAGCGGGCTGGGGTTGGCGGTGTGGAG

ACAGCCTGAGGGACTAGGAAGTGGTGATCGTGGGTGGAGGGAGCAATACCTAAGGGCTGG

GGAGTGGAAAGCCACAACAGAGGGAACCTGGGAGAAGGTCTGGACCCACAGGAGAGACAA

GGTGCCAGTGTTGGGGAGGGGAGAGGAGGAGGGGCGGGCCACCATAGAATACTCCTTGCA

CCCCAGCGTGCACACTTGCCTCCAGCTAGCAGAGAGCAGAGCTTCCCAGACATCCCCCCT

CCCCCACCCCACACGCCCTGGCCAGAAGCCACCTGCCATCCGGCAGACTGGCCTCACCAC

CCGTGGGAAGCCAACCACCACGGGCTTTCCCTGGCCGGCCTGCCCCCGGAGGCCCACTCT

GCGAGCAACCCCGCCGGAAGAGCCGGCCCAGGACCAGGGCAAGCCCTGCCCGCCATGGGA

AGTCCTCCAGGTGCACCTGCCAGTCCCTGCCCTGGGAAGTGCTCCTTTGCCTCGAGAGCC

GTAGCCCCCCACCCTCAGGAAGTGCCACGAACCCCAGCACCACCCCTGGGAAGAGCTCCG

CCCAGGAGCACCAGGGCAAGCCCTGCCACGAAGTGGCTCCAGTGAACCTCCAGTCCTGCC

GCCCTCAGGAAGTCTCCTTTGCTCGAGGACCGTAGCCCCCCTCAGGAAAGCCCTGCCTCA

AAGAAGGCCGCCAACACCACCGCCCTCAGGAAGCACTCCACAGCAGTGCCAGGGCAAACC

CTGCCGGCCATGGGGAGTGCACCTCCACCAGAAAAAGAAAAACACAAGCAAGATGAAGAA

GCTCAGAAACCATTCCCAGTTAAAGCAACAGGAGAACTCACCTAAAGCAGTCAACAATGA

AACAGACCTCTGCAGTCTGACAGACTTGGAGTTCAAAAGGGAGATAGTGAAAATACTGAA

GGAATTAAGAGAAGATATGAACAGTAATGCAGACTCCCTCAGAAAGGAACTAGAAAATAT

AAGGAGGAGCCAAGAAAAACTAGAAAATTCATTTGCAGAGATACAAACTGAGCTAAGGGC

AGTAAAAACCAGAATGAATAATGCAGAGGAACGAATTAGTGATGTGGAAGATAGAATAAT

GGAAATCACCCAAACAGGACAGCAGACAGAAAACCAAATGAAAAAACATGAAAGCAATAT

AAGAGACCTATGGGATAATATAAAGTGGGCCAATCTATACATAATAGGAATTCCAGAAGG

AGAAGAAAAAGAAAAGGGGATTGAAAATATATTTGAAGAAATTATCCTGGAAACTTTCCA

AATCTAAAGGATACTGATTTCAAGATACAGGAAGCACAGAGGGCCCCAAACAAGTTGAAC

CCAAATAGACCCACACCAAGACATATTATAATAAAAATGGCAAAAGTTAATGATAAAGAG

AGGATCCTAAAGGCAGCAAGAGAAAAGCAAAGTGTCACTTATAAGGGAACCCCCATAAGG

CTATCAGCTGATTTCTCTACAGAAACTCTACAGGCCAGGAGGGAATGGCAAGAGATATTT

AAAGCACTGAAAGGAAAAAATATGCAACCTAGAATACTCTATCCAGCAAGAATATCATTT

AAAATAGAAGGGGAAATAAAAAATTTCTCCAACAAACAAAAGCTAAAAGAATACAGCAAT

ACAAAACCCATTCTAAAAGAAATACTGAAAGGGCTTCTCTAAATTAAAAAAAAAAAAAAA

GAGGAAGAACTAGGATGGAGGAAACCACAATCAGAGAGCAGTCACTTAAATAAGCCAGCA

TACAGATCTAATCATGAAGATGTTTAAAATAAAATAAAATTAAAAAAAAGACATCAAAAT

CATAAAATGTGGGCAAGGGAAGTAAGGAAATAAATAGATTCTTTTTTTTTTTTTTTTTAA

ATTTTATTATGGTAATGAAGTGTTTGAACCTACAGGACTATCAGGCTAAAACACACAATT

ATAGGAAGGGGTTAACATACTTAAAAAAAAGGGCAAGCACAAATCAAAACCAAACATTAC

ATTCACAAAAAATGAAAAGAAAAGTACTCAAGCAGAAAATAATTGGAGACCATCCAACAA

AAAAAAGAAAGGAAGAATGGAGAATCATAGAATCAACTGGAAAACGAGGTTTAAAATGGC

AATAAATAAACATCTATCAATTATCACCTTAAATGTCAATGGACTGAATGCTCCAATCAA

AAGACACAGAGTGGCTGATTGGATAAAAAAGCAAAAACCTTCAATCTGCTGCCTACAAGA

AACTCACCTTAGGACAAAGGACACATATAGATTGAAAGTGAGGGGGTGGGAAAAGATATT

TCATGCCAATGGACAAGACAGGAAAGCAGGAGTTGCAATACTCATATCAGACAAAATAGA

CTTTAAAATGAAGGCCATAAAGAAAGACAAAGAAGGACACTATTTAATGGTTAAAGGATC

CATTCAAGAAGAGGATATTACAATCGTCAACATATATGCCCCAAATATAGGAGCACCCAG

ATACATACAACAAATATTAACAGACATAAAAGGAGAAATTGATGGGAATACAATCATAGT

AGGAGACTTTAACACCCCACTCACATCAATGGACAGATCCTCTAGACAGAAAACCAATAA

AGCAACAGAGATCCTAAAGGAAACAATAGAAAAGTTAGACTTAATTGACATTTTCAGGAC

ATTACATCCAAAAAAATCAGAATACACATTCTTCTCAAGTGCGCATGGAACATTCTCAAG

AATTGATCACATATTGGGGCACAAAGCTAACCTCAACAAATTTAAGAGTATAGAAATTAT

TTCAAGTATCTTCTCTGACCACAATGGCATGAAACTAGAAATCAACCACAGGAAAAGAAA

TGAGAAAAAACCTACTACATGGAGACTAAACAACATGCTACTAAAAAACCAATGGGTCAA

TGAGGAAATCAAGAAGGAAATTAAAAAATACCTTGAGACAAATGATAATGAAGACACAAC

CTCTCAAAATCTATGGGATGCTGCAAAAGCAGTGCTCAGAGGGAAATTCATAGCAATACA

GGCCTTCCTCAAAAAAGAAGAAAAATCTCAAATTGACAACTTAACCCACCACCTAAATGA

ATTAGAAAAAGAAGACAAAAAAACCTAAAGTCAGCAGAAGGAAGGAAATCATAAAGATCA

AAGAGGAAATCAATAAAATAGAGATTCAAAAAAAAAAAAAAAATCAATAAAACCAAGAGC

TGGTTCTTTGAAAAGGTAAACAAAATTGACAAACCTCTGGCTAGACTCACCAAGAAGAGG

AGAGAAAGAACCCAAATAAACAAAATAGAAATGAAAAAGGAGAAATCACAATGGATACTG

CAGAAATACAAAAAAACATAAGAGAATACTATGAACAATTATATGCCAACAAATTTGACA

ATCTGGAAGAAATGGACAACTTTCTAGAGTCTTACAGCCTGCCAAAACTGAATCAAGAAG

AAATAGACCAACTGAACAGACCGATCACTAGAAATGAAATTGAATAGTCATAAAAACACT

CCCTACAAATAAAAGTCCAGGACCAGATGGCTTCACAGGCGAATTCTACCAAACATACAA

AGAGGAACTGGTGCCCATCCTCCTTAAACTTTTTCAAAAGGTTGAAGAAGAAGGAACACT

CCCAAAGACATTCTATGATGCCACCATCACCCTAATTCCAAAACCAGACAAAGATACCAC

CAAAAAAGAAAACTATGGCCAATATCTTTGATGAATATAGACGCAAAAATTCTCAACAAA

ATTTTAGCCAACCGAATCCAACAACATATCAAAAAGATCATACACCATGACCAGGTGGGA

TTCATCCCAGGTCACAAGGATGGTTCAACATATGCAAATCAATCAACGTCATACACCACA

TTAACAAAAGAAAAGTCAAAAACCATATGATCATCTCAATAGATGCAGAAAAAGCATTTG

ACAAAGTCCAACATCCATTCATGATAAAAACTCTCACCAAAGTGGGTATAGAGGGAACAT

TCCTTAACATAATCAAAGCCATTTATGACAAACCCACAGCAAATATAATACTCAATGGAG

AAAAACTGAAAGCCTTCCCACTAAAATCTGGAACAAGACAGGGATGCCCACTCTCACCAC

TGCTATTCAACATAGTATTGGAAGTCCTAGCCACAGCAATCAGACAAACAAAAGAAATAA

AAGGCATCCAAATAGGAAGAGAAGAGATAAAACTGTCACTGTATGCAGATGACATGATAC

TATACATAGAAAACCCTAAGGACTCAACCCAAAAACTACTTGAACTGATCAACAAATTCA

GCAAAGTAGCAGGATATAAGATTAACATTCAGAAATCAGTCACATTTCTGTATACTAACA

ATGAAATATTAGAAAAGGAATACAAAAATACAATACCTTTTAAAATTGCACCCCAAAAAT

CAAATACCTGGGAATACACCTGACCAAGGAGGTAAAGGACTTATATGCGAGAACTATAAA

ACATTAATCAAGGAAATTAAAGAAGATGTAAAGAAATGGAAAGATATTCCATGCTCCTGG

GTTGGAAAAATTAATATTGTAAAAATGGCCATACTACCCAAAGCAATCTACAGATTCAAT

GCAATCCCTATCAAATTACCCATGACATTTTTCACAGAACTAGAACAAACAATCCAAAAA

TTTATATGGAACCACAAAAGACCCAGAATTGCCAAAGCAATCCTGAGAAACAAAAACCAA

GCAGGAGGCATAACTCTCCCAGACTTCAAGCAATATTACAAAGCCACAGTCATCAAGACA

GTGTGGTACTGGTACCAAAACAGACATACAGACCAATGGAACAGAATAGAGAACCCAGAA

ATAAACCCTGACACCTATGGTCAATTAATCTTTGACAAAGGAGGCAAGAACATAAAATGG

GAAAAAGACAGTCTATTCAGCAAGAATTGCTGGGAAACCTGGACAGCTGCATGCAAATCA

ATGAAACTAGAACACACCCTCACACCATGCACAAAAATAAACTCAAAATGGCTTAAAGAC

TTAAATATAAGACAAGACACCATCAAACTCCTGGAAGAGAACATAGGCAAAACATTCTCT

GACATCAACCTTATGAATATTTTCTCAGGTCAGTCTCCCAAAGCAACAGAAATAAAAGCA

AAAATAAACCAATGGGACCTAATCAAACTGACAAGCTTTTGCACAGCAAAGGAAACCAAA

AAGAAAACAAAAAGACAACTTACAGAATGGGAGAAAATAGTTTCAAATGATGCAACGACA

AGGGCTTAATCTCTAGAATATACAACAACTTATACAACTCAACAGCAAAAAAGCCAACAA

CCCAATGGAAAAATGGGCAAAAGACCTGAATAGACATTTCTCCAAGGAAGATATACAGAT

GGCCAACAAGCACATGAAAAATGCTCAACATCCTGATTATTAGAGAAATGCAAATCAAAA

CTACCATGAGATACCACCTCACACCAGTCAGAATGGCCATCATTAATAAGTCCACAAATA

ACAAATGCTGGAGGGGGTGTGGAGAAAAGGGAACCCTCCTGCACTGTTGGTGGGAATGTA

AGCTGGTACAGCCACTATGGAGAACAGTATGGAGGTACCTTAGAAACTATACATAGAACT

ACCATATGACCCAGCAATCCCACTCTTGGGCATATATCCAGACAAAACTTTCCTTAAAAA

AGACACATGCACCCCATGTTCATTGCAGCTCTATTCACAATAGCCAAGACATGGAAACAA

CCCAAATGTCCATACAGATGATTGGATTAGGAAGATGTGGTATATATACACAATGGAATA

CTACTCAGCCATAAAAAAGAACAAATAATGCCATTTGCAGCAACATGGATGGAACTAGAG

ACTCTCATACTGAGTGAATAAGTCAGAAAGAGAAAGACAAATACCATATGATATCACTAT

ATCTGGAATCTAATATACAGACAAATGAACCTTTCCACAGAAAAGAAAATCATGGACTTG

GAGAATAGACTTGTGGTTGCCTGGGGGAGGGGAGGGAGTGGGAGGGATTGGGAGCTTGGG

GTTAAGGATGCAAACTATTGCTCTTGGAATGGATTTACAATGAGATCCTGCTGTGTAGCA

CTGAGAACTATGTCTAGATACTAAAGCAGCATGACAATGGGAGAAAAAATTATGTATACA

TGTATGTGTAACTGGTCCCATGCTGTACAGTGGAAAAAAAAAGTGTTAAAAATAAAAAAA

AAATAAAAA

>L1C13#LINE/L1C

GGAGCAAGATGGGAAGAGTAGGGGGAGCTGCCCTCTCCCACAAACACAAAAAAAACACAT

CTACAGTAAACACTCACAGAACAGCAACTGAATGCTGGCAGAAGAACTTAAACCTCCAAA

AAGGCAAGAATCTCTTGACATAACTGGGTAAAACAAGAGAAAAGAGGAGAGGAGAGAAGG

GGAATCAGGACGGACGGCACTCCGAGAGGGAACTGTGAAGGAGAAAGGGAACCCACACCC

TGGAAAGTCACCTAAAGAAAGATCAACAGTCGAGGGATCTCCAGACAGAAAAGCCAGCAG

AGGTCTGAGATCTGAAAAGCAGAGTGAGAGCACAGATCATCTGAACCACTGGCACAGACA

CCAAAAACGAGATGCTGGGTGGGGGCTGGGCACCAGACCTAGGCTCGAGGTTAGTCCCTG

GGAGGGCTGGGGTTGGGTGTGGAGACAGCCTGAGGGATAGGAAGGTGTGGTGGAGGGAGC

AATACTAAGGGCTGGGGAGTGGAAAGCCACAACAGAGGGAACCTGGGAGAAGGTCTGGAC

CCACAGGAGAGACAAGGGCCAGTGTTGGGGAGGGGAGAGGAGGAGGGGGGCCCATAGAAT

ACTCCTTGCCCCAGGGCTTGCCCCAGCTAGCAGAGAGCAGAGCTTCCCAGGCATCCCCCC

TCCCCACCCGCGCCTGGCCAGAAGCCCTGCCATCCGGGACTGGCCTCACCACCGGAAGCC

ACCACCCGGCTTTCCCGCCGCCTGCCCCACAGGAGGCCACTCCTGAGCACACACCCCCCT

GGAAGAGTCCTGCCCAGGAGCCAGGGCAAGCCCTGCCCAGCCATGGGAAGTGTGCCTCCA

CGCAGTGCAACCTGCCAGTCCCCCCCTCGGAAGCTCCTTTGCTCGAGTGACCGCTAGCCC

CGCCCACCCTGGGAAATGCCCCTGCCTCAAAGAAGACCAGCCAACACCCCGCCCTCGGAA

GACTCCACAGCAGCCCAGGGCAAACCCTGCCGGCCACGGGGAGTGCACCTCCACCACAAA

AAGAAAAAACAAGCAAGATGAAGAAGCTCAGAAACCATTCCCAGTTAAACCAACAGGAGA

ACTCACCTAAAGCAGTCAACAATGAAACAGACCTCTGCAGTCTGACAGACTTGGAGTTCA

AAAGGGAGATAGTGAAAATACTGAAGGAATTAAGAGAAGATATGAACAGTAATGCAGACT

CCCTCAGAAAGGAACTAGAAAATATAAGGAGGAGCCAAGAAAAACTAGAAAATTCATTTG

CAGAGATACAAACTGAGCTAAGGGCAGTAAAAACCAGAATGAATAATGCAGAGGAATGAA

TTAGTGATGTGGAAGATAGAATAATGGAAATCACCCAAACAGGACAGCAGACAGAAAACC

AAATGAAAAAACATGAAAGCAATATAAGAGACCTATGGGATAATATAAAGTGGGCCAATC

TACGCATAATAGGAATTCCAGAAGGAGAAGAAAAAGAAAAGGGGATTGAAAATATATTTG

AAGAAATTATCACTGGAAACTTTCCAAATCTAAAGGATACTGATTTCAAGATACAGGAAG

CACAGAGGGCCCCAAACAAGTTGAACCCAAATAGACCCACACCAAGACATATTATAATAA

AAATGGCAAAAGTTAATGATAAAGAGAGGATCCTAAAGGCAGCAAGAGAAAAGCAAAGTG

TCATTATAAGGGAACCCCCATAAGGCTATCAGCTGATTTCTCTACAGAAACTCTACAGGC

CAGGAGGGAATGGCAAGAGATATTTAAAGCCTGAAAGGAAAAAATATGCAACCTAGAATA

CTCTATCCAGCAAGAATATCATTTAAAATAGAAGGGGAAATAAAAAATTTTCCAACAAAC

AAAAGCTAAAAGAATACAGCAATACAAAACCCATTCTAAAAGAAATACTGAAAGGGCTTC

TCTAAATTAAAAAAAAAAAAAAAAAAAGAGGAAGAACTAGGATGGAGGAAACCACAATCA

GAGAGCAGTCACTCAAATAAGCCAGCATACAGATCTAATCATGAAGATGTTTAAAATAAA

ATAAAATTAAAAAAAAAAGACATCAAAATCATAAAATGTGGGCAAGGGAAGTAAGGAAAT

AAATAGATTCTTTTTTTTTTTTTTTTTTTAAATTTTATTATGGTAATGAAGTGTTTGAAC

CTACAGGACTATCAGGCTAAAACACACAATTATAGGAAGGGGTTAACATACTTAAAAAAC

AGGGCAAGCACAAATCAAAACCAAACATTACATTCACAAAAAATGAAAAGAAAATACTCA

AGCAGAAAATAATTGGAGACCATCCAACCAAAAAAAGAAAGGAAGAATGGAGAATCATAG

AATCAACTGGAAAACGAGGTTTAAAATGGCAATAAATAATCATCTATCAATTATCACCTT

AAATGTCAATGGACTGAATGCTCCAATCAAAAGACACAGAGTGGCTGATTGGATAAAAAA

GCAAAAACCTTCAATCTGCTGCCTACAAGAAACTCACCTTAGGACAAAGGACACATATAG

ATTGAAAGTGAGGGGGTGGGAAAAGATATTTCATGCCAATGGACAAGACAGGAAAGCAGG

AGTTGCAATACTCATATCAGACAAAATAGACTTTAAAACGAAGGCCATAAAGAAAGACAA

AGAAGGACACTATTTAATGGTTAAAGGATCCATTCAAGAAGAGGATATTACAATCTCAAC

ATATATGCCCCAAATATAGGAGCACCCAGATACATACAACAAATATTAACAGACATAAAA

GGAGAAATTGATGGGAATACAATCATAGTAGGAGACTTTAACACCCCACTCACATCAATG

GACAGATCCTCTAGACAGAAAACCAATAAAGCAACAGAGATCCTAAAGGAAACAATAGAA

AAGTTAGACTTAATTGACATCTTCAGGACACTACATCCAAAAAAATCAGAATACACATTC

TTCTCAAGTGCACATGGAACATTCTCAAGAATTGATCACATATTGGGGCACAAAGCTAAC

CTCAACAAATTTAAGAGTATAGAAATTATTTCAAGTATCTTCTCTGACCACAATGGCATG

AAACTAGAAATCAACCACAGGAAAAGAAATGAGAAAAAACCTACTACATGGAGACTAAAC

AACATGCTACTAAAAAACAATGGGTCAATGAGGAAATCAAGAAGGAAATTAAAAAATACC

TCGAGACAAATGATAATGAAGACACAACCTCTCAAAATCTATGGGATGCCGCAAAAGCAG

TGCTCAGAGGGAAATTCATAGCAATACAGGCCTTCCTCAAAAAAGAAGAAAGATCTCAAA

TTGACAACTTAACCCACCACCTAAATGAATTAGAAAAAGAAGAACAAAAAAAACCTAAAG

TCAGCAGAAGGAAGGAAATCATAAAGATCAAAGAGGAAATCAATAAAATAGAGATTCAAA

AAACAATAGAAAAAATCAATAAAACCAAGAGCTGGTTCTTTGAAAAGGTAAACAAAATTG

ACAAACCTCTGGCTAGACTCACTAAGAAGAGGAGAGAAAGAACCCAAATAAACAAAATTA

GAAATGAAAAAGGAGAAATCACAACAGATACTGCAGAAATACAAAAAACCATAAGAGAAT

ACTATGAACAATTATATGCCAACAAATTTGACAATCTGGAAGAAATGGACAACTTTCTAG

AGTCTTACAGCCTGCCAAAACTGAATCAAGAAGAAATAGACCAACTGAACAGACCATCAC

TAGAAATGAAATTGAATAGTCATAAAAACACTCCCTACAAATAAAAGTCCAGGACCAGAT

GGCTTCACAGGTGAATTCTACCAAACATACAAAGAGGAACTGGTGCCCATCCTCCTTAAA

CTTTTTCAAAAGGTTGAAGAAGAAGGAACACTCCCAAAGACATTCTATGATGCCACCATC

ACCCTAATTCCAAAACCAGACAAAGATACCACCAAAAAAGAAAACTATCGGCCAATATCT

TTGATGAATATAGATGCAAAAATTCTCAACAAAATTTTAGCCAACCGAATCCAACAACAT

ATCAAAAAGATCATACACCATGACCAGGTGGGATTCATCCCAGGTTCACAAGGATGGTTC

AACATACGCAAATCAATCAACGTCATACACCACATTAACAAAAGAAAAGTCAAAAACCAT

ATGATCATCTCAATAGATGCAGAAAAAGCATTTGACAAAGTCCAACATCCATTTATGATA

AAAACTCTCACCAAAGTGGGTATAGAGGGAACATTCCTTAACATAATCAAAGCCATTTAT

GACAAACCCACAGCAAATATAATACTCAATGGAGAAAAACTGAAAGCCTTCCACTCAAAT

CTGGAACAAGACAGGGATGCCCACTCTCACCACTGCTATTCAACATAGTTTGGAAGTCCT

AGCCACAGCAATTAGACAAACAAAAGAAATAAAAGGCATCCAAATAGGAAGAGAAGAGAT

AAAACTGTCACTGTATGCAGATGACATGATACTATACATAGAAAACCCTAAGGACTCAAC

CCAAAAACTACTTGAACTGATCAACAAATTCAGCAAAGTAGCAGGATATAAGATTAACAT

TCAGAAATCAGTCACATTTCTGTATACTAACAATGAAATATTAGAAAAGGAATACAAAAA

TACAATACCTTTTAAAATTGCACCCCAAAAAATCAAATACCTGGGAATACACCTGACCAA

GGAGGTAAAGGACTTATATGCCGAGAACTATAAAACATTAATCAAGGAAATTAAAGAAGA

TGTAAAGAAATGGAAAGATATTCCATGTTCCTGGATTGGAAAAATTAATATTGTAAAAAT

GGCCATACTACCCAAAGCAATCTACAGATTCAATGCAATCCCTATCAAATTACCCATGAC

ATTTTTCACAGAACTAGAACAAACAATCCAAAAATTTATATGGAACCACAAAAGACCCAG

AATTGCCAAAGCAATCCTGAGAAACAAAAACCAAGCAGGAGGCATAACTCTCCCAGACTT

CAAGCAATATTACAAAGCCACAGTCATCAAGACAGTGTGGTACTGGTACCAAAACAGACA

GACAGACCAATGGAACAGAATAGAGAACCCAGAAATAAACCCTGACACCTATGGTCAATT

AATCTTTGACAAAGGAGGCAAGAACATAAAATGGGAAAAAGAAAGTCTATTCAGCAAGAT

TGCTGGGAAACCTGGACAGCTGCATGCAAATCAATGAAACTAGAACACACCCTCACACCA

TGCACAAAAATAAACTCAAAATGGCTGAAAGACTTAAATATAAGACAAGACACCATCAAA

CTCCTGGAAGAGAACATAGGCAAAACATTCTCTGACATCAACCTCATGAATATTTTCTCA

GGTCAGTCTCCCAAAGCAACAGAAATAAAGCAAAAATAAACCAATGGGACCTAATCAAAC

TGACAAGCTTTTGCACAGCAAAGGAAACCAAAAAGAAAACAAAAAGACAACTTACAGAAT

GGGAGAAAATAGTTTCAAATGATGCAACTGACAAGGGCTTAATCTCTAGAATATACAAGC

AACTTATACAACTCAACAGCAAAAAAGCCAACAACCCAATGGAAAAATGGGCAAAAGACC

TGAATAGACATTTCTCCAAGGAAGATATACAGATGGCCAACAAGCACATGAAAAAAATGC

TCAACATCCCTGATTATTAGAGAAATGCAAATCAAAACTACCATGAGATACCACCTCACA

CCAGTCAGAATGGCCATCATTAATAAGTCCACAAATAACAATGCTGGAGGGGTGTGGAGA

AAAGGGAACCCTCCTGCACTGTTGGTGGGAATGTAAGCTGGTACAGCCACTATGGAGAAC

AGTATGGAGGTACCTTAGAAATCTATACATAGAACTACCATATGACCCAGCAATCCCACT

CTTGGGCATATATCTGGACAAAACTTTCCTTAAAAAAGACACATGCACCCGCATGTTCAT

TGCAGCTCTATTCACAATAGCCAAGACATGGAAACAACCCAAATGTCCATTGACAGATGA

TTGGATTAGGAAGATGTGGTATATATACACAATGGAATACTACTCAGCCATAAAAAAGAA

TGAATAATGCCATTTGCAGCAACATGGATGGAACTAGAGACTCTCATACTGAGTGAAATA

AGTCAGAAAGAGAAAGACAAATACCATATGATATCACTATATCTGGAATCTAATATACAG

CACAAATGAACCTTTCCACAGAAAAGAAAATCATGGACTTGGAGAATAGACTTGTGGTGC

CGGGGAGGGGGAGGGAGTGGGAGGGATTGGGAGCTTGGGGTTAAGGATGCAAACTATTGC

TCTTGGAATGGATTTACAATGAGATCCTGCTGTGTAGCACTGAGAACTATGTCTAGATAC

TTACATGCACAACAATGGGAGAAAAAATTATGTATACATGTATGTGTAACTGGTCCCCAT

GCTGTACAGTGGAAAAAAAAAATAAAAAAAAAAAAAAAAAAA

>L1D1#LINE/L1D

GGGAGAGAGGACAAGATGGCGGAGGAGTAGGGGGACACGCTCGCCCTCTCCCACAAACAC

AACAAAAAAAGCACATCTACAGAAGAAATGACTCGCACAGAACAACAACCAATCGCTGGC

AGAGGAACCTAAACTCCAATAACGGCAAGAAGTTCGTGACATTATTGGGCAGAACGGGAG

AAAAGAGGAGAGTGAGAGAAGGTGAATCCGAGCGGGACGGGCGCTCCCGAAAGGGAACTG

CGGAGGAGAAAGGGATCCCGCACCCTGGAAAGTCTCCTACCGGGGGAAAGATCAAACGAA

CCGGAGGAATCTCCAGATGCAGAGAAGAGTGTAGCAGTAAGTCGGAGTACGGAAAAACGA

TCAAGAACCCAACGGACCATCTGAACTACGGGCACAGTCACCAAAAATTGAGACGCCTGG

GTGGGGGCTGGGCACCGAATCCTCGGCTCCAGAGGTTAGTCCCCGGGAAAGGGCCGGGGG

ACGCCTGGGTGGGGGCTGGGCACCGAGACCTCAGCTCTGAAGGTTGGTCCCCGAGAGGGG

GCCGGGGGACGCCTGGGTTGGGGCTGGGCACCGAGACCTCGCCTCCGAAGGTTAGTCCCC

AAGAAAGGGCCGGGGGACGCCTGGGGGGGGGGGCTGGGCACCGAGACCTCGGCTCCAGAG

ATTAGTCCCCGGGCTAGGGGGGCGGGGAAGAGCGGAAACTGCTTGGGAGGTCTCTAAACC

ATTTGACGGGGCAGAGACTGCCTGGGAGACTAGAAAACAAAGCTGTCGCAGAGGAAGGGA

GCAATACTCTAGGGGCGGGGAAGTGGAAAGCCGCCTCAGAGGGAACCTGGGAGAAGAGCC

TGGTCTGCGCCCGTGCTGGGGAGGGGAGAGAAGAAGGGGTGGGTCCCCATAGAATACCCC

CCACGCCACAGCAAGCTTACAGGCCCGCTAGCTAGCAGAAAGCTGTGCTTCCCAGTGCAT

TCCCTCCCCCCACCCCCGCCACCCCCTACGCTCTCGCCGAACCTGGGGCTGCCTGCCATC

CAGGAGGGCTGGCCTCAACAATTGCCTGAAGCCTACCACCGCAGGGGCTCTCCCTGCACA

GGCCTGCTTGCCCTTTGGAGGGGCTACACTTCCACAGAGCAGCACCAAACACCACCAGCC

CCCTAGAAAAGGCCTGCAGCCCAGAAAAGCTAGAACAAGCCTAGCCAGGCCGTGAATAGA

TCGACCTAATTCTCCGACGGTTTTTCTGAGACGGGCTCCCCCGGGGAGGAGCCTCTTGAG

TCTCCAAGGGCCTTGCTACACGCCCAAGACCCCAGGGGGTGCTAAGACCTAGTGGACCAG

CTGCATAGGACTGCCAGCTCCAGGCAGGACCCCCTGCAGCCCAGAAAAGCTGCAACAAGC

CTGGCCGAATTGGGAAAAGATCTCAGACGGTCTTTCTGAGTCGGGCTGTCCTGGGGAGGA

GCCTCTTGAGTCTCCAAGGGCCCTGCTACCCGCCCAAGACCCCAAGGGGTGCTGAGACCT

AGTGGACCAGCTGCATAGGACTGCCAGCTCCAGGCAGGACCCCCTGCAGCCCAGAAAAGC

TGCAACAAGCCTGGCCGAATCGGGAAAAGATCTCAGACGGTCTTTCTGAGTCGGGCTGTC

CTGGGGAGGAGCCTCTTGAGCCTCCAAGGGCCCTGCTACCCGCCCAAGACCCCAGGGGGT

GCTGAGACCTAGTGGACCAGCTGCATAGGACTGCCAGCTCCAGGCAGGACCCCCTGCAGC

CCAGAAAAGCTGCAACAAGCCTGGCCGAATTGGGAAAAGATCTCAGACGGTCTTTCTGAG

TCGGGCTGTCCTGGGGAGGAGCCTCTTGAGCCTCCAAGGGCCCTGCTACCCGCCCAAGAC

CCCAGGGGGTGCTGAGACCTAGTGGACCAGCTGCATAGGACTGCCAGCTCCAGGCAGGAC

CCCCTGCAGCCCAGAAAAGCTGCAACAAGCCTGGCCGACTTGGGAAAAGATCTCAGACGG

TCTTTCTGAGTCGGGCTGTTCTGGGGAGGAGCCTCTTGAGTCTCCAAAGGCCCTGCTACC

CGCCCAAGACCCCAAGGGGTGCTGAGACCTAGTGGACCAGCTGCATAGGACTGCCAGCTC

CAGGCAGGACCCCCTGCAGCCCAGAAAAGCTGCAACAAGCCTGGCGGAATCGGGAAAAGA

TCTCAGACGGTCTTTCTGAGTCGGGCTGCCCTGGGGAGGAACCTCTTGAGTCTCCAAGGG

CCCTGCTACCCGCCCAAGACCCCAGGGGGTGCTGAGAACCAAGTGAAATCTGCTACCATC

GTGGGGTGGACCTCCCAGTCCTGTCTGCCCTCAGGAAGTCCTCCTTTGCTTCAAAGAAAC

ACTGTTAGTCCCATCAACACTCCAGAAAAGCCACACTGCCTCAAAAAAAGATTGACCAAC

AACGACAGCCCTCAGGAAATATTCCAGGGCAGTGACAAGGCAAACACTACCCGATAACGG

AGAGTACAACTCCCTCAGGAGAAAGAAGACAACAAGCAAGATGAAGAAGCTGAGAAACCA

CCCCCAGTCAAACCAACAGGAGAACTCACCTAAAACAGTCAACAATGAAACTGATCTCTG

CAGTCTGACAGACCTGGAGTTCAAAAGAGAAATAGTGAAAATACTGAAGGAATTAAGAGA

AGATATGAACAGCAATGCAGATACCCTCAGAAAGGAGCTAGAAAATATAAGGAGGAGCCA

AGAAAAACTAGAACATTCATTTGCAGAGATGCAAACTGAACTAGGGGCAGTAAAAACCAG

AATGAATAATGCAGAAGAACGAATCAGTGATATGGAAGATAGAATAATGGAAATCACTCA

ATCTGGTCAACAGACAGAAAACCGAATCAAAAAACTGGAAAGCAATATAAGAGACCTATG

GGATAATATAAAACGGGCCAATCTACGCATAATAGGAATTCCAGAAGGAGTAGAAAAAGA

TAAAGGAATGGAAAATATATTTGAAGAAATTATCGCTGGAAACTTCCCAAATCTAAAGGA

TACTGGATTCAAGATACAAGAAGCACAGAGGGCCCCAAACAAACTGAACCCAAACAGACC

CACACCAAGACACATCATAATAAAAATGGCAAAAGTTAGTGATAAAGAGAGGATCCTAAA

GGCAGCAAGAGAAAAACAGAATGTTACCTACAAGGGAACCCCCATAAGAATATCAGCTGA

TTTCTCTACAGAAACACTACAGGCCAGGAGGGAATGGCAAGAGATATTTAAAGTGCTCAA

AGGAAAAAATATGCAACCTAGAATACTTTATCCAGCAAGAATATCATTTAAAATAGAAGG

GGAAATAAAAATTTTTCCCAACAAACAAAAACTTAAAGAATACAGCAACACAAAACCCAG

GTTAAAGGAAATATTGAAAGGGCTTCTCTAAACCAAAAAGAAAGGAAGGAAAGGGAAGAA

AAAAGAAAAGAAAAAAAAAAAAAAAAAGAAGAAGAAGAAGAGGAAGAACTAGGACTGAGG

AAGATACAATCAGAGAGCAGTCACTCAAATAAGCCAGCATACAGATTTAATCATGAACAT

GCTTCAAACAAAATAAAATTAAAAAGAAAAAAATAAAAGAGTCATCAAAACCATAAAATG

TGGGCAAGGGATGTTAGGAGGTAAATAATCCTTTTTGTTTGTATGTATGTCTCTCTTCTT

AATTTTAATATAATAATGAAGTGTTTGAACTTACAGGACCATCAGGCTAAAACACACAAT

TATGGGAAGGGGTTAGCATACTTAAAAAACAGGGCAACCACAAGCCAAAACCAAATATTG

CATTTGCAAAAAATGAAAAAAAAAAATACACTCAAGCAGATAATAACAGGAGACCATCCA

ACCAAAAAAAAAAAAAAAAAAAAAAAAGAAGAATGGAGAACCATAGAATCAACTGGAACA

CGAGGATCAAATGGCAATAAATAATCATCTATCAATTATCACCTTAAATGTCAATGGACT

GAATGCCCCAATCAAAAGACACAGAATGGCTGAGTGGATAAAACGGCAAAAACCTTCAAT

ATGCTGCCTACAAGAAACTCACCTTAGGACAAAAGATACATATAGATTGAAAGTGAAAGG

CTGGGGAAAAGTATTTCATGCCAATAGACATGACAGAAAAGCAGGAGTTGCAACGCTCAT

ATCAGACAAAATAGACTTTAAAACAAAAGACATAAAGAAAGACAAAGAAGGACACTATTT

AATGATTAAGGGATCCATCCAAGGAGAGGATGTTACTATCATCAACATATATGCCCCAAA

CATAGGAGCACCCAGATACATACAACAAATATTAACAGACATAAAGGGAGATATTGATGA

GAATACAATCATAGTAGGAGACCTAAATACCCCCCTCACATCAATGGACAGATCCTCTAG

ACAGAAAACCAATAAAGCAACAGAGATCCTAAAGGAAACAATAGAAAAGTTAGACTTAAT

TGATATCTTCAGGACACTACATCCAAAAAAATCAGAATACACATTCTTCTCAAATGCTCA

TGGAACATTCTCAAGAATCGACCACATATTGGGACATAAAGCGAATCTCAATAAATTTAG

GAGCATAGAAATTATCTCAAGTATCTTCTCTGACCACAATGCCATGAAATTAGAAATCAA

CCATGGGAAAAGGAAAGAGAAAAAACCTACTCCATGGAGACTAAACAACATGCTACTAAA

AAACCAATGGGTCAATGAGGAAATCAAGAAGGAAATTAAAAACTATCTTGAAACAAATGA

TAATGAAGACACAACCTCTCAAAATCTATGGGATGCTGCGAAAGCAGTGCTCAGAGGGAA

ATTTATAGCAATCCAGGCCTTTCTCAAAAAAGAAGAAAGATCCCAAATTGACAACTTAAC

CCTCCACCTAAATGAATTAGAAAAAGAAGAACAAAGAAGTCCTAAAGTCAGCAGAAGGAA

GGAAATTGTAAAGATCAAAGAAGAAATCAATAAAATAGAGACTCAAAAAACAATAGAGAA

AATTAATAAAACCAAGAGCTGGTTCTTTGAAAAGGTGAACAAAATTGATAAACCCCTGGC

CAGACTCACTAAAAAGAGGAGAGAAAGAACCCAAATCACCAAAATTATAAATGAAAAAGG

AGAAATCACAACGGATACAGCAGAAATACAAAAAACCATAAGAGAATACTATGAACAACT

ATATGGCAATAAGTTTGACAATCTGGAAGAAATGGACAATTTTCTAGAATCTTACAGCCT

GCCAAAACTGAATCAAGCAGAAACAGACCAACTGAACAGACCAATCACTAGAAATGAAAT

TGAAGAGGTCATAAAATCACTCCCTACAAATAAAAGCCCAGGACCAGATGGCTTCACAGG

TGAATTCTATCAAACATATAAAGAGGAATTGGTGCCCATCCTCCTTAAACTCTTTCAAAA

GGTTGAAGAAGAAGGAATACTCCCAAAGACATTCTATGAGGCCACCATCACCCTCATTCC

AAAACCAGGCAGAGATACCACCAAAAAAGAAAACTATCGCCCAATATCATTGATGAATAT

AGATGCAAAACTTCTCAACAAAATCTTAGCCAACCGAATCCAACAACATATCAAAAAAAT

TATACACCATGACCAGGTTGGGTTCATCCCAGGTTCACAAGGATGGTTCAACATACGCAA

ATCAATCAACATCATACACCACATTAACAAAAAAAAAGTCAAAAATCATATGATCATCTC

AATAGATGCAGAAAAAGCATTTGACAAAGTTCAACATCCATTCATGATCAAGACCCTCGC

CAAAGTGGGTATAGAGGGAACATTCCTGAATATAATCAAAGCCATTTATGATAAACCCAC

AGCAAATATAATCCTCAATGGGGAAAAACTGAAAGCCTTCTCACTCAAATCTGGAACAAG

ACAGGGATGCCCACTCTCACCACTGCTCTTCAACATAGTTTTGGAAGTCTTAGCCACAGC

AATTAGACAAACAAAAGAAATCAAAGGCATCCATATAGGAAGAGAAGAGATCAAACTGTC

ACTGTATGCAGATGACATGATTCTATACCTAGAAAACCCTAAGGACTCAACCCCAAAACT

CCTTGAACTGATTAATAAATTCAGCAAAGTGGCAGGATATAAGATTAACATTCAGAAGTC

AGTTGCATTTCTGTATACCAGCAATGAAGCATTAGAAAAGGAATACAAAAATACGATACC

TTTTAAAATTGTACCTCACAAAATCAAATACCTCGGAATACACCTAACCAAAGAGGTAAA

GGACCTATATGCCGAGAACTATAAAACCTTAATCAAAGAAATCAAAGAAGATGTAAAAAA

ATGGAAAGATATTCCATGTTCCTGGATTGGAAAAATCAATATTGTGAAAATGGCCATCCT

ACCCAAAGCAATCTACAGATTCAATGCAATCCCTATCAAATTACCCATGACATTTTTCAC

AGAACTAGAACAAACAATCCAAACATTTATATGGAACAACAAAAGACCCAGAATCGCCAA

AGCAATCCTGAGAAACAAAAACCAAGCAGGAGGCATAACTCTCCCAGACTTCAAGAAATA

CTACAAAGCCACAGTCATCAAAACAGTGTGGTACTGGTATCAAAACAGACAGACAGACCA

ATGGAACAGAATAGAGAATCTGGAAATTAACCCTGACACCTATGGTCAATTAATCTTTGA

CAAGGGAGGCAAGAACATCAAATGGGAAAAGGAAAGTCTATTCAGCAAGCATTGCTGGGA

AACCTGGACAGCTGTATGCAAAGCAATGAAACTAGAACACACCCTCACACCATGCACAAA

AATAAACTCCAAATGGCTGAAAGACTTAAATATACGACAGGACACCATCAAACTCCTAGA

AGAAAACATAGGCAAAACACTCTCTGACATCAACATCATGAATATTTTCTCAGGTCAGTC

TCCCAAAGCAATAGAAACTAGAGCAAAAATAAACCCATGGGACCTCATCAAACTGAAAAG

CTTTTGCACAGCAAAGGAAACCCAAAAGAAAACAAAAAGACAACTTACAGAATGGGAGAA

AATAGTTTCAAATGATGCAACTGACAAGGGCTTAATCTCTAGAATATACAAGCAACTTAT

ACAACTCAACAGCAAAAAAACCAATCAATCAATGGAAAAATGGGCAAAAGACCTGAATAG

ACATTTCTCCAAGGAAGATATACAGATGGCCAACAAACACATGAAAAAATGCTCAACATC

GCTGATTATAAGAGAAATGCAAATCAAAACTACCATGAGATACCACCTCACACCAGTCAG

AATGGCCATCATTAATAAATCCACAAATAACAAGTGCTGGAGGGGCTGTGGAGAAAAGGG

AACCCTCCTGCACTGTTGGTGGGAATGTAAACTGGTACAGCCACTATGGAGAACAGTTTG

GAGATACCTTAGAAATCTATACATAGAACTTCCATATGACCCTGCAATCCCACTCTTGGG

CATCTATCCGGACAAAGCTCTACTTAAAAGAGACACATGCACCCGCATGTTCATTGCAGC

ACTATTCACAATAGCCAGGACATGGAAACAATCCAAATGTCCATCGACAGAGGATTGGAT

TCGGAAGATGTGGTATATATACACGATGGAATACTACTCAGCCATAAAAAAGGATGACAT

CATGCCATTTGCAGCAACATGGATGGAACTAGAGAATCTCATACTGAGTGAAATGAGCCA

GAAAGACAAAGACAAATACCATATGACATCACTTATAACTGGAATCTAATATCCAGCACA

AATGAACATCTCCTCAGAAAAGAAAATCAATCATGGACTTGGAGAAGAGACTTGTGGTTG

CCTGATGGGAGGGGGAGGGAGTGGGAGGGATCGGGAGCTTGGGCTTATCAGTCACAACGT

AGAATAGATTTACAAGGAGATCCCGCTGAATAGCATTGAGAACTATGTCTAGATACTCAT

GTTGCAACAGAAGAAATGGTGGGGGAAAAACTGTAATTGTAATGTATACATGTAAGGATA

ACCTGACCCCCTTGCTGTACAGTGGGAAAATAAAATTAAAAAAAAAAAAAAAAAAAAAAA

AAAAAAAAAAAAAAAAAAAAAAA

>L1D2#LINE/L1D

GAGAGGACAAGATGGCGGAGGAGTAGGGGGACACGCTCGCCCTCTCCCACAAACACAACA

AAAAAAGCACATCTACAGAAGAAATGACTCGCACAGAACAACAACCAATCGCTGGCAGAG

GAACCTAAACTCCAATAACGGCAAGAAGTTCGTGACATTATTGGGCAGAACGGGAGAAAA

GAGGAGAGTGAGAGAAGGTGAATCCGAGCGGGACGGGCGCTCCCGAAAGGGAACTGCGGA

GGAGAAAGGGATCCCGCACCCTGGAAAGTCTCCTACCGGGGGAAAGATCAAACGAACCGG

AGGAATCTCCAGATGCAGAGAAGAGTGTAGCAGTAAGTCGGAGTACGGAAAAACGATCAA

GAACCCAACGGACCATCTGAACTACGGGCACAGTCACCAAAAATTGAGACGCCTGGGTGG

GGGCTGGGCACCGAATCCTCGGCTCCAGAGGTTAGTCCCCGGGAAAGGGCCGGGGGACGC

CTGGGTGGGGGCTGGGCACCGAGACCTCAGCTCTGAAGGTTGGTCCCCGAGAGGGGGCCG

GGGGACGCCTGGGTTGGGGCTGGGCACCGAGACCTCGCCTCCGAAGGTTAGTCCCCAAGA

AAGGGCCGGGGGACGCCTGGTGGGGGGGGGCTGGGCACCGAGACCTCGGCTCCAGAGATT

AGTCCCCGGGCTAGGGGGGCGGGGAAGAGCGGAAACTGCTTGGGAGGTCTCTAAACCATT

TGACGGGGCAGAGACTGCCTGGGAGACTAGAAAACAAAGCTGTCGCAGAGGAAGGGAGCA

ATACTCTAGGGGCGGGGAAGTGGAAAGCCGCCTCAGAGGGAACCTGGGAGAAGAGCCTGG

TCTGCGCCCGTGCTGGGGAGGGGAGAGAAGAAGGGGTGGGTCCCCATAGAATACCCCCCA

CGCCACAGCAAGCTTACAGGCCCGCTAGCTAGCAGAAAGCTGTGCTTCCCAGTGCATTCC

CTCCCCCCACCCCCGCCACCCCCTACGCTCTCGCCGAACCTGGGGCTGCCTGCCATCCAG

GAGGGCTGGCCTCAACAATTGCCTGAAGCCTACCACCGCAGGGGCTCTCCCTGCACAGGC

CTGCTTGCCCTTTGGAGGGGCTACACTTCCACAGAGCAGCACCAAACACCACCAGCCCCC

TAGAAAAGGCCTGCAGCCCAGAAAAGCTAGAACAAGCCTAGCCAGGCCGTGAATAGATCG

ACCTAATTCTCCGACGGTTTTTCTGAGACGGGCTCCCCCGGGGAGGAGCCTCTTGAGTCT

CCAAGGGCCTTGCTACACGCCCAAGACCCCAGGGGGTGCTAAGACCTAGTGGACCAGCTG

CATAGGACTGCCAGCTCCAGGCAGGACCCCCTGCAGCCCAGAAAAGCTGCAACAAGCCTG

GCCGAATTGGGAAAAGATCTCAGACGGTCTTTCTGAGTCGGGCTGTCCTGGGGAGGAGCC

TCTTGAGTCTCCAAGGGCCCTGCTACCCGCCCAAGACCCCAAGGGGTGCTGAGACCTAGT

GGACCAGCTGCATAGGACTGCCAGCTCCAGGCAGGACCCCCTGCAGCCCAGAAAAGCTGC

AACAAGCCTGGCCGAATCGGGAAAAGATCTCAGACGGTCTTTCTGAGTCGGGCTGTCCTG

GGGAGGAGCCTCTTGAGTCTCCAAGGGCCCTGCTACCCGCCCAAGACCCCAGGGGGTGCT

GAGACCTAGTGGACCAGCTGCATAGGACTGCCAGCTCCAGGCAGGACCCCCTGCAGCCCA

GAAAAGCTGCAACAAGCCTGGCCGAATCGGGAAAAGATCTCAGACGGTCTTTCTGAGTCG

GGCTGTCCTGGGGAGGAGCCTCTTGAGCTCCAAGGGCCCTGCTACCCGCCCAAGACCCCA

GGGGGTGCTGAGACCTAGTGGACCAGCTGCATAGGACTGCCAGCTCCAGGCAGGACCCCC

TGCAGCCCAGAAAAGCTGCAACAAGCCTGGCCGAATCGGGAAAAGATCTCAGACGGTCTT

TCTGAGTCGGGCTGTCCTGGGGAGGAGCCTCTTGAGCCTCCAAGGGCCCTGCTACCCGCC

CAAGACCCCAGGGGGTGCTGAGACCTAGTGGACCAGCTGCATAGGACTGCCAGCTCCAGG

CAGGACCCCCTGCAGCCCAGAAAAGCTGCAACAAGCCTGGCCGAATTGGGAAAAGATCTC

AGACGGTCTTTCTGAGTCGGGCTGTCCTGGGGAGGAGCCTCTTGAGTCTCCAAGGGCCCT

GCTACCCGCCCAAGACCCCAGGGGGTGCTGAGACCTAGTGGACCAGCTGCATAGGACTGC

CAGCTCCAGGCAGGACCCCCTGCAGCCCAGAAAAGCTGCAACAAGCCTGGCCGAATTGGG

AAAAGATCTCAGACGGTCTTTCTGAGTCGGGCTGTTCTGGGGAGGAGCCTCTTGAGTCTC

CAAAGGCCCTGCTACCCGCCCAAGACCCCAAGGGGTGCTGAGACCTAGTGGACCAGCTGC

ATAGGACTGCCAGCTCCAGGCAGGACCCCCTGCAGCCCAGAAAAGCTGCAACAAGCCTGG

CGGAATCGGGAAAAGATCTCAGACGGTCTTTCTGAGTCGGGCTGCCCTGGGGAGGAACCT

CTTGAGTCTCCAAGGGCCCTGCTACCCGCCCAAGACCCCAGGGGGTGCTGAGAACCAAGT

GAAATCTGCTACCATCGTGGGGTGGACCTCCCAGTCCTGTCTGCCCTCAGGAAGTCCTCC

TTTGCTTCAAAGAAACACTGTTAGTCCCATCAACACTCCAGAAAAGCCACACTGCCTCAA

AAAAAGATTGACCAACAACGACAGCCCTCAGGAAATATTCCAGGGCAGTGACAAGGCAAA

CACTACCCGATAACGGAGAGTACAACTCCCTCAGGAGAAAGAAGACAACAAGCAAGATGA

AGAAGCTGAGAAACCACCCCCAGTCAAACCAACAGGAGAACTCACCTAAAACAGTCAACA

ATGAAACTGATCTCTGCAGTCTGACAGACCTGGAGTTCAAAAGAGAAATAGTGAAAATAC

TGAAGGAATTAAGAGAAGATATGAACAGCAATGCAGATACCCTCAGAAAGGAGCTAGAAA

ATATAAGGAGGAGCCAAGAAAAACTAGAACATTCATTTGCAGAGATGCAAACTGAACTAG

GGGCAGTAAAAACCAGAATGAATAATGCAGAAGAACGAATCAGTGATATGGAAGATAGAA

TAATGGAAATCACTCAATCTGGTCAACAGACAGAAAACCGAATCAAAAAACTGGAAAGCA

ATATAAGAGACCTATGGGATAATATAAAACGGGCCAATCTACGCATAATAGGAATTCCAG

AAGGAGTAGAAAAAGATAAAGGAATGGAAAATATATTTGAAGAAATTATCGCTGGAAACT

TCCCAAATCTAAAGGATACTGGATTCAAGATACAAGAAGCACAGAGGGCCCCAAACAAAC

TGAACCCAAACAGACCCACACCAAGACACATCATAATAAAAATGGCAAAAGTTAGTGATA

AAGAGAGGATCCTAAAGGCAGCAAGAGAAAAACAGAATGTTACCTACAAGGGAACCCCCA

TAAGAATATCAGCTGATTTCTCTACAGAAACACTACAGGCCAGGAGGGAATGGCAAGAGA

TATTTAAAGTGCTCAAAGGAAAAAATATGCAACCTAGAATACTTTATCCAGCAAGAATAT

CATTTAAAATAGAAGGGGAAATAAAAATTTTTCCCAACAAACAAAAACTTAAAGAATACA

GCAACACAAAACCCAGGTTAAAGGAAATATTGAAAGGGCTTCTCTAAACCAAAAAGAAAG

GAAGGAAAGGGAAGAAAAAAGAAAAGAAAAAAAAAAAAAAAGAAGAAGAAGAAGAGGAAG

AACTAGGACTGAGGAAGATACAATCAGAGAGCAGTCACTCAAATAAGCCAGCATACAGAT

TTAATCATGAACATGCTTCAAACAAAATAAAATTAAAAAGAAAAAAATAAAAGAGTCATC

AAAACCATAAAATGTGGGCAAGGGATGTTAGGAGGTAAATAATCCTTTTTGTTTGTATGT

ATGTCTCTCTTCTTAATTTTAATATAATAATGAAGTGTTTGAACTTACAGGACCATCAGG

CTAAAACACACAATTATGGGAAGGGGTTAGCATACTTAAAAAACAGGGCAACCACAAGCC

AAAACCAAATATTGCATTTGCAAAAAATGAAAAAAAAAAATACACTCAAGCAGATAATAA

CAGGAGACCATCCAACCAAAAAAAAAAAAAAAAAAAAAAAAGAAGAATGGAGAACCATAG

AATCAACTGGAACACGAGGATCAAATGGCAATAAATAATCATCTATCAATTATCACCTTA

AATGTCAATGGACTGAATGCCCCAATCAAAAGACACAGAATGGCTGAGTGGATAAAACGG

CAAAAACCTTCAATATGCTGCCTACAAGAAACTCACCTTAGGACAAAAGATACATATAGA

TTGAAAGTGAAAGGCTGGGGAAAAGTATTTCATGCCAATAGACATGACAGAAAAGCAGGA

GTTGCAACGCTCATATCAGACAAAATAGACTTTAAAACAAAAGACATAAAGAAAGACAAA

GAAGGACACTATTTAATGATTAAGGGATCCATCCAAGGAGAGGATGTTACTATCATCAAC

ATATATGCCCCAAACATAGGAGCACCCAGATACATACAACAAATATTAACAGACATAAAG

GGAGATATTGATGAGAATACAATCATAGTAGGAGACCTAAATACCCCCCTCACATCAATG

GACAGATCCTCTAGACAGAAAACCAATAAAGCAACAGAGATCCTAAAGGAAACAATAGAA

AAGTTAGACTTAATTGATATCTTCAGGACACTACATCCAAAAAAATCAGAATACACATTC

TTCTCAAATGCTCATGGAACATTCTCAAGAATCGACCACATATTGGGACATAAAGCGAAT

CTCAATAAATTTAGGAGCATAGAAATTATCTCAAGTATCTTCTCTGACCACAATGCCATG

AAATTAGAAATCAACCATGGGAAAAGGAAAGAGAAAAAACCTACTCCATGGAGACTAAAC

AACATGCTACTAAAAAACCAATGGGTCAATGAGGAAATCAAGAAGGAAATTAAAAACTAT

CTTGAAACAAATGATAATGAAGACACAACCTCTCAAAATCTATGGGATGCTGCGAAAGCA

GTGCTCAGAGGGAAATTTATAGCAATCCAGGCCTTTCTCAAAAAAGAAGAAAGATCCCAA

ATTGACAACTTAACCCTCCACCTAAATGAATTAGAAAAAGAAGAACAAAGAAGTCCTAAA

GTCAGCAGAAGGAAGGAAATTGTAAAGATCAAAGAAGAAATCAATAAAATAGAGACTCAA

AAAACAATAGAGAAAATTAATAAAACCAAGAGCTGGTTCTTTGAAAAGGTGAACAAAATT

GATAAACCCCTGGCCAGACTCACTAAAAAGAGGAGAGAAAGAACCCAAATCACCAAAATT

ATAAATGAAAAAGGAGAAATCACAACGGATACAGCAGAAATACAAAAAACCATAAGAGAA

TACTATGAACAACTATATGGCAATAAGTTTGACAATCTGGAAGAAATGGACAATTTTCTA

GAATCTTACAGCCTGCCAAAACTGAATCAAGCAGAAACAGACCAACTGAACAGACCAATC

ACTAGAAATGAAATTGAAGAGGTCATAAAATCACTCCCTACAAATAAAAGCCCAGGACCA

GATGGCTTCACAGGTGAATTCTATCAAACATATAAAGAGGAATTGGTGCCCATCCTCCTT

AAACTCTTTCAAAAGGTTGAAGAAGAAGGAATACTCCCAAAGACATTCTATGAGGCCACC

ATCACCCTCATTCCAAAACCAGGCAGAGATACCACCAAAAAAGAAAACTATCGCCCAATA

TCATTGATGAATATAGATGCAAAACTTCTCAACAAAATCTTAGCCAACCGAATCCAACAA

CATATCAAAAAAATTATACACCATGACCAGGTTGGGTTCATCCCAGGTTCACAAGGATGG

TTCAACATACGCAAATCAATCAACATCATACACCACATTAACAAAAAAAAAGTCAAAAAT

CATATGATCATCTCAATAGATGCAGAAAAAGCATTTGACAAAGTTCAACATCCATTCATG

ATCAAGACCCTCGCCAAAGTGGGTATAGAGGGAACATTCCTGAATATAATCAAAGCCATT

TATGATAAACCCACAGCAAATATAATCCTCAATGGGGAAAAACTGAAAGCCTTCTCACTC

AAATCTGGAACAAGACAGGGATGCCCACTCTCACCACTGCTCTTCAACATAGTTTTGGAA

GTCTTAGCCACAGCAATTAGACAAACAAAAGAAATCAAAGGCATCCATATAGGAAGAGAA

GAGATCAAACTGTCACTGTATGCAGATGACATGATTCTATACCTAGAAAACCCTAAGGAC

TCAACCCCAAAACTCCTTGAACTGATTAATAAATTCAGCAAAGTGGCAGGATATAAGATT

AACATTCAGAAGTCAGTTGCATTTCTGTATACCAGCAATGAAGCATTAGAAAAGGAATAC

AAAAATACGATACCTTTTAAAATTGTACCTCACAAAATCAAATACCTCGGAATACACCTA

ACCAAAGAGGTAAAGGACCTATATGCCGAGAACTATAAAACCTTAATCAAAGAAATCAAA

GAAGATGTAAAAAAATGGAAAGATATTCCATGTTCCTGGATTGGAAAAATCAATATTGTG

AAAATGGCCATCCTACCCAAAGCAATCTACAGATTCAATGCAATCCCTATCAAATTACCC

ATGACATTTTTCACAGAACTAGAACAAACAATCCAAACATTTATATGGAACAACAAAAGA

CCCAGAATCGCCAAAGCAATCCTGAGAAACAAAAACCAAGCAGGAGGCATAACTCTCCCA

GACTTCAAGAAATACTACAAAGCCACAGTCATCAAAACAGTGTGGTACTGGTATCAAAAC

AGACAGACAGACCAATGGAACAGAATAGAGAATCTGGAAATTAACCCTGACACCTATGGT

CAATTAATCTTTGACAAGGGAGGCAAGAACATCAAATGGGAAAAGGAAAGTCTATTCAGC

AAGCATTGCTGGGAAACCTGGACAGCTGTATGCAAAGCAATGAAACTAGAACACACCCTC

ACACCATGCACAAAAATAAACTCCAAATGGCTGAAAGACTTAAATATACGACAGGACACC

ATCAAACTCCTAGAAGAAAACATAGGCAAAACACTCTCTGACATCAACATCATGAATATT

TTCTCAGGTCAGTCTCCCAAAGCAATAGAAATTAGAGCAAAAATAAACCCATGGGACCTC

ATCAAACTGAAAAGCTTTTGCACAGCAAAGGAAACCCAAAAGAAAACAAAAAGACAACTT

ACAGAATGGGAGAAAATAGTTTCAAATGATGCAACTGACAAGGGCTTAATCTCTAGAATA

TACAAGCAACTTATACAACTCAACAGCAAAAAAACCAATCAATCAATGGAAAAATGGGCA

AAAGACCTGAATAGACATTTCTCCAAGGAAGATATACAGATGGCCAACAAACACATGAAA

AAATGCTCAACATCGCTGATTATAAGAGAAATGCAAATCAAAACTACCATGAGATACCAC

CTCACACCAGTCAGAATGGCCATCATTAATAAATCCACAAATAACAAGTGCTGGAGGGGC

TGTGGAGAAAAGGGAACCCTCCTGCACTGTTGGTGGGAATGTAAACTGGTACAGCCACTA

TGGAGAACAGTTTGGAGATACCTTAGAAATCTATACATAGAACTTCCATATGACCCTGCA

ATCCCACTCTTGGGCATCTATCCGGACAAAGCTCTACTTAAAAGAGACACATGCACCCGC

ATGTTCATTGCAGCACTATTCACAATAGCCAGGACATGGAAACAATCCAAATGTCCATCG

ACAGAGGATTGGATTCGGAAGATGTGGTATATATACACGATGGAATACTACTCAGCCATA

AAAAAGGATGACATCATGCCATTTGCAGCAACATGGATGGAACTAGAGAATCTCATACTG

AGTGAAATGAGCCAGAAAGACAAAGACAAATACCATATGACATCACTTATAACTGGAATC

TAATATCCAGCACAAATGAACATCTCCTCAGAAAAGAAAATCAATCATGGACTTGGAGAA

GAGACTTGTGGTTGCCTGATGGGAGGGGGAGGGAGTGGGAGGGATCGGGAGCTTGGGCTT

ATCAGTCACAACGTAGAATAGATTTACAAGGAGATCCCGCTGAATAGCATTGAGAACTAT

GTCTAGATACTCATGTTGCAACAGAAGAAATGGTGGGGGAAAAACTGTAATTGTAATGTA

TACATGTAAGGATAACCTGACCCCCTTGCTGTACAGTGGGAAAATAAAATTAAAAAAAAA

AAAAAAAAAAAAAAAAAAAAAAAAAAAAAAAAAAAAAA

>L1D3#LINE/L1D

GAGAGGACAAGATGGCGGAGGAGTAGGGGGACACGCTCGCCCTCTCCCACAAACACAACA

AAAAAAGCACATCTACAGAAGAAATGACTCGCACAGAACAACAACCAATCGCTGGCAGAG

GAACCTAAACTCCAATAACGGCAAGAAGTTCGTGACATTATTGGGCAGAACGGGAGAAAA

GAGGAGAGTGAGAGAAGGTGAATCCGAGCGGGACGGGCGCTCCCGAAAGGGAACTGCGGA

GGAGAAAGGGATCCCGCACCCTGGAAAGTCTCCTACCGGGGGAAAGATCAAACGAACCGG

AGGAATCTCCAGATGCAGAGAAGAGTGTAGCAGTAAGTCGGAGTACGGAAAAACGATCAA

GAACCCAACGGACCATCTGAACTACGGGCACAGTCACCAAAAATTGAGACGCCTGGGTGG

GGGCTGGGCACCGAATCCTCGGCTCCAGAGGTTAGTCCCCGGGAAAGGGCCGGGGGACGC

CTGGGTGGGGGCTGGGCACCGAGACCTCAGCTCTGAAGGTTGGTCCCCGAGAGGGGGCCG

GGGGACGCCTGGGTTGGGGCTGGGCACCGAGACCTCGCCTCCGAAGGTTAGTCCCCAAGA

AAGGGCCGGGGGACGCCTGGGGGGGGGGGGGCTGGGCACCGAGACCTCGGCTCCAGAGAT

TAGTCCCCGGGCTAGGGGGGCGGGGAAGAGCGGAAACTGCTTGGGAGGTCTCTAAACCAT

TTGACGGGGCAGAGACTGCCTGGGAGACTAGAAAACAAAGCTGTCGCAGAGGAAGGGAGC

AATACTCTAGGGGCGGGGAAGTGGAAAGCCGCCTCAGAGGGAACCTGGGAGAAGAGCCTG

GTCTGCGCCCGTGCTGGGGAGGGGAGAGAAGAAGGGGTGGGTCCCCATAGAATACCCCCC

ACGCCACAGCAAGCTTACAGGCCCGCTAGCTAGCAGAAAGCTGTGCTTCCCAGTGCATTC

CCTCCCCCCACCCCCGCCACCCCCTACGCTCTCGCCGAACCTGGGGCTGCCTGCCATCCA

GGAGGGCTGGCCTCAACAATTGCCTGAAGCCTACCACCGCAGGGGCTCTCCCTGCACAGG

CCTGCTTGCCCTTTGGAGGGGCTACACTTCCACAGAGCAGCACCAAACACCACCAGCCCC

CTAGAAAAGGCCTGCAGCCCAGAAAAGCTAGAACAAGCCTAGCCAGGCCGTGAATAGATC

GACCTAATTCTCCGACGGTTTTTCTGAGACGGGCTCCCCCGGGGAGGAGCCTCTTGAGTC

TCCAAGGGCCTTGCTACACGCCCAAGACCCCAGGGGGTGCTAAGACCTAGTGGACCAGCT

GCATAGGACTGCCAGCTCCAGGCAGGACCCCCTGCAGCCCAGAAAAGCTGCAACAAGCCT

GGCCGAATTGGGAAAAGATCTCAGACGGTCTTTCTGAGTCGGGCTGTCCTGGGGAGGAGC

CTCTTGAGTCTCCAAGGGCCCTGCTACCCGCCCAAGACCCCAAGGGGTGCTGAGACCTAG

TGGACCAGCTGCATAGGACTGCCAGCTCCAGGCAGGACCCCCTGCAGCCCAGAAAAGCTG

CAACAAGCCTGGCCGAATCGGGAAAAGATCTCAGACGGTCTTTCTGAGTCGGGCTGTCCT

GGGGAGGAGCCTCTTGAGTCTCCAAGGGCCCTGCTACCCGCCCAAGACCCCAGGGGGTGC

TGAGACCTAGTGGACCAGCTGCATAGGACTGCCAGCTCCAGGCAGGACCCCCTGCAGCCC

AGAAAAGCTGCAACAAGCCTGGCCGAATCGGGAAAAGATCTCAGACGGTCTTTCTGAGTC

GGGCTGCCCTGGGGAGGAGCCTCTTGAGCCTCCAAGGGCCCTGCTACCCGCCCAAGACCC

CAAGGGGTGCTGAGACCTAGTGGACCAGCTGCATAGGACTGCCAGCTCCAGGCAGGACCC

CCTGCAGCCCAGAAAAGCTGCAACAAGCCTGGCCGATTGGGAAAAGATCTCAGACGGTCT

TTCTGAGTCGGGCTGTCCTGGGGAGGAGCCTCTTGAGCCTCCAAGGGCCCTGCTACCCGC

CCAAGACCCCAGGGGGTGCTGAGACCTAGTGGACCAGCTGCATAGGACTGCCAGCTCCAG

GCAGGACCCCCTGCAGCCCAGAAAAGCTGCAACAAGCCTGGCCGACTTGGGAAAAGATCT

CAGACGGTCTTTCTGAGTCGGGCTGTTCTGGGGAGGAGCCTCTTGAGTCTCCAAAGGCCC

TGCTACCCGCCCAAGACCCCAAGGGGTGCTGAGACCTAGTGGACCAGCTGCATAGGACTG

CCAGCTCCAGGCAGGACCCCCTGCAGCCCAGAAAAGCTGCAACAAGCCTGGCGGAATCGG

GAAAAGATCTCAGACGGTCTTTCTGAGTCGGGCTGCCCTGGGGAGGAACCTCTTGAGTCT

CCAAGGGCCCTGCTACCCGCCCAAGACCCCAGGGGGTGCTGAGAACCAAGTGAAATCTGC

TACCATCGTGGGGTGGACCTCCCAGTCCTGTCTGCCCTCAGGAAGTCCTCCTTTGCTTCA

AAGAAACACTGTTAGTCCCATCAACACTCCAGAAAAGCCACACTGCCTCAAAAAAAGATT

GACCAACAACGACAGCCCTCAGGAAATATTCCAGGGCAGTGACAAGGCAAACACTACCCG

ATAACGGAGAGTACAACTCCCTCAGGAGAAAGAAGACAACAAGCAAGATGAAGAAGCTGA

GAAACCACCCCCAGTCAAACCAACAGGAGAACTCACCTAAAACAGTCAACAATGAAACTG

ATCTCTGCAGTCTGACAGACCTGGAGTTCAAAAGAGAAATAGTGAAAATACTGAAGGAAT

TAAGAGAAGATATGAACAGCAATGCAGATACCCTCAGAAAGGAGCTAGAAAATATAAGGA

GGAGCCAAGAAAAACTAGAACATTCATTTGCAGAGATGCAAACTGAACTAGGGGCAGTAA

AAACCAGAATGAATAATGCAGAAGAACGAATCAGTGATATGGAAGATAGAATAATGGAAA

TCACTCAATCTGGTCAACAGACAGAAAACCGAATCAAAAAACTGGAAAGCAATATAAGAG

ACCTATGGGATAATATAAAACGGGCCAATCTACGCATAATAGGAATTCCAGAAGGAGTAG

AAAAAGATAAAGGAATGGAAAATATATTTGAAGAAATTATCGCTGGAAACTTCCCAAATC

TAAAGGATACTGGATTCAAGATACAAGAAGCACAGAGGGCCCCAAACAAACTGAACCCAA

ACAGACCCACACCAAGACACATCATAATAAAAATGGCAAAAGTTAGTGATAAAGAGAGGA

TCCTAAAGGCAGCAAGAGAAAAACAGAATGTTACCTACAAGGGAACCCCCATAAGAATAT

CAGCTGATTTCTCTACAGAAACACTACAGGCCAGGAGGGAATGGCAAGAGATATTTAAAG

TGCTCAAAGGAAAAAATATGCAACCTAGAATACTTTATCCAGCAAGAATATCATTTAAAA

TAGAAGGGGAAATAAAAATTTTTCCCAACAAACAAAAACTTAAAGAATACAGCAACACAA

AACCCAGGTTAAAGGAAATATTGAAAGGGCTTCTCTAAACCAAAAAGAAAGGAAGGAAAG

GGAAGAAAAAAGAAAAGAAAAAAAAAAAAAAAGAAGAAGAAGAAGAGGAAGAACTAGGAC

TGAGGAAGATACAATCAGAGAGCAGTCACTCAAATAAGCCAGCATACAGATTTAATCATG

AACATGCTTCAAACAAAATAAAATTAAAAAGAAAAAAATAAAAGAGTCATCAAAACCATA

AAATGTGGGCAAGGGATGTTAGGAGGTAAATAATCCTTTTTGTTTGTATGTATGTCTCTC

TTCTTAATTTTAATATAATAATGAAGTGTTTGAACTTACAGGACCATCAGGCTAAAACAC

ACAATTATGGGAAGGGGTTAGCATACTTAAAAAACAGGGCAACCACAAGCCAAAACCAAA

TATTGCATTTGCAAAAAATGAAAAAAAAAAATACACTCAAGCAGATAATAACAGGAGACC

ATCCAACCAAAAAAAAAAAAAAAAAAAAAAGAAGAATGGAGAACCATAGAATCAACTGGA

ACACGAGGATCAAATGGCAATAAATAATCATCTATCAATTATCACCTTAAATGTCAATGG

ACTGAATGCCCCAATCAAAAGACACAGAATGGCTGAGTGGATAAAACGGCAAAAACCTTC

AATATGCTGCCTACAAGAAACTCACCTTAGGACAAAAGATACATATAGATTGAAAGTGAA

AGGCTGGGGAAAAGTATTTCATGCCAATAGACATGACAGAAAAGCAGGAGTTGCAACGCT

CATATCAGACAAAATAGACTTTAAAACAAAAGACATAAAGAAAGACAAAGAAGGACACTA

TTTAATGATTAAGGGATCCATCCAAGGAGAGGATGTTACTATCATCAACATATATGCCCC

AAACATAGGAGCACCCAGATACATACAACAAATATTAACAGACATAAAGGGAGATATTGA

TGAGAATACAATCATAGTAGGAGACCTAAATACCCCCCTCACATCAATGGACAGATCCTC

TAGACAGAAAACCAATAAAGCAACAGAGATCCTAAAGGAAACAATAGAAAAGTTAGACTT

AATTGATATCTTCAGGACACTACATCCAAAAAAATCAGAATACACATTCTTCTCAAATGC

TCATGGAACATTCTCAAGAATCGACCACATATTGGGACATAAAGCGAATCTCAATAAATT

TAGGAGCATAGAAATTATCTCAAGTATCTTCTCTGACCACAATGCCATGAAATTAGAAAT

CAACCATGGGAAAAGGAAAGAGAAAAAACCTACTCCATGGAGACTAAACAACATGCTACT

AAAAAACCAATGGGTCAATGAGGAAATCAAGAAGGAAATTAAAAACTATCTTGAAACAAA

TGATAATGAAGACACAACCTCTCAAAATCTATGGGATGCTGCGAAAGCAGTGCTCAGAGG

GAAATTTATAGCAATCCAGGCCTTTCTCAAAAAAGAAGAAAGATCCCAAATTGACAACTT

AACCCTCCACCTAAATGAATTAGAAAAAGAAGAACAAAGAAGTCCTAAAGTCAGCAGAAG

GAAGGAAATTGTAAAGATCAAAGAAGAAATCAATAAAATAGAGACTCAAAAAACAATAGA

GAAAATTAATAAAACCAAGAGCTGGTTCTTTGAAAAGGTGAACAAAATTGATAAACCCCT

GGCCAGACTCACTAAAAAGAGGAGAGAAAGAACCCAAATCACCAAAATTATAAATGAAAA

AGGAGAAATCACAACGGATACAGCAGAAATACAAAAAACCATAAGAGAATACTATGAACA

ACTATATGGCAATAAGTTTGACAATCTGGAAGAAATGGACAATTTTCTAGAATCTTACAG

CCTGCCAAAACTGAATCAAGCAGAAACAGACCAACTGAACAGACCAATCACTAGAAATGA

AATTGAAGAGGTCATAAAATCACTCCCTACAAATAAAAGCCCAGGACCAGATGGCTTCAC

AGGTGAATTCTATCAAACATATAAAGAGGAATTGGTGCCCATCCTCCTTAAACTCTTTCA

AAAGGTTGAAGAAGAAGGAATACTCCCAAAGACATTCTATGAGGCCACCATCACCCTCAT

TCCAAAACCAGGCAGAGATACCACCAAAAAAGAAAACTATCGCCCAATATCATTGATGAA

TATAGATGCAAAACTTCTCAACAAAATCTTAGCCAACCGAATCCAACAACATATCAAAAA

AATTATACACCATGACCAGGTTGGGTTCATCCCAGGTTCACAAGGATGGTTCAACATACG

CAAATCAATCAACATCATACACCACATTAACAAAAAAAAAGTCAAAAATCATATGATCAT

CTCAATAGATGCAGAAAAAGCATTTGACAAAGTTCAACATCCATTCATGATCAAGACCCT

CGCCAAAGTGGGTATAGAGGGAACATTCCTGAATATAATCAAAGCCATTTATGATAAACC

CACAGCAAATATAATCCTCAATGGGGAAAAACTGAAAGCCTTCTCACTCAAATCTGGAAC

AAGACAGGGATGCCCACTCTCACCACTGCTCTTCAACATAGTTTTGGAAGTCTTAGCCAC

AGCAATTAGACAAACAAAAGAAATCAAAGGCATCCATATAGGAAGAGAAGAGATCAAACT

GTCACTGTATGCAGATGACATGATTCTATACCTAGAAAACCCTAAGGACTCAACCCCAAA

ACTCCTTGAACTGATTAATAAATTCAGCAAAGTGGCAGGATATAAGATTAACATTCAGAA

GTCAGTTGCATTTCTGTATACCAGCAATGAAGCATTAGAAAAGGAATACAAAAATACGAT

ACCTTTTAAAATTGTACCTCACAAAATCAAATACCTCGGAATACACCTAACCAAAGAGGT

AAAGGACCTATATGCCGAGAACTATAAAACCTTAATCAAAGAAATCAAAGAAGATGTAAA

AAAATGGAAAGATATTCCATGTTCCTGGATTGGAAAAATCAATATTGTGAAAATGGCCAT

CCTACCCAAAGCAATCTACAGATTCAATGCAATCCCTATCAAATTACCCATGACATTTTT

CACAGAACTAGAACAAACAATCCAAACATTTATATGGAACAACAAAAGACCCAGAATCGC

CAAAGCAATCCTGAGAAACAAAAACCAAGCAGGAGGCATAACTCTCCCAGACTTCAAGAA

ATACTACAAAGCCACAGTCATCAAAACAGTGTGGTACTGGTATCAAAACAGACAGACAGA

CCAATGGAACAGAATAGAGAATCTGGAAATTAACCCTGACACCTATGGTCAATTAATCTT

TGACAAGGGAGGCAAGAACATCAAATGGGAAAAGGAAAGTCTATTCAGCAAGCATTGCTG

GGAAACCTGGACAGCTGTATGCAAAGCAATGAAACTAGAACACACCCTCACACCATGCAC

AAAAATAAACTCCAAATGGCTGAAAGACTTAAATATACGACAGGACACCATCAAACTCCT

AGAAGAAAACATAGGCAAAACACTCTCTGACATCAACATCATGAATATTTTCTCAGGTCA

GTCTCCCAAAGCAATAGAAACTAGAGCAAAAATAAACCCATGGGACCTCATCAAACTGAA

AAGCTTTTGCACAGCAAAGGAAACCCAAAAGAAAACAAAAAGACAACTTACAGAATGGGA

GAAAATAGTTTCAAATGATGCAACTGACAAGGGCTTAATCTCTAGAATATACAAGCAACT

TATACAACTCAACAGCAAAAAAACCAATCAATCAATGGAAAAATGGGCAAAAGACCTGAA

TAGACATTTCTCCAAGGAAGATATACAGATGGCCAACAAACACATGAAAAAATGCTCAAC

ATCGCTGATTATAAGAGAAATGCAAATCAAAACTACCATGAGATACCACCTCACACCAGT

CAGAATGGCCATCATTAATAAATCCACAAATAACAAGTGCTGGAGGGGCTGTGGAGAAAA

GGGAACCCTCCTGCACTGTTGGTGGGAATGTAAACTGGTACAGCCACTATGGAGAACAGT

TTGGAGATACCTTAGAAATCTATACATAGAACTTCCATATGACCCTGCAATCCCACTCTT

GGGCATCTATCCGGACAAAGCTCTACTTAAAAGAGACACATGCACCCGCATGTTCATTGC

AGCACTATTCACAATAGCCAGGACATGGAAACAATCCAAATGTCCATCGACAGAGGATTG

GATTCGGAAGATGTGGTATATATACACGATGGAATACTACTCAGCCATAAAAAAGGATGA

CATCATGCCATTTGCAGCAACATGGATGGAACTAGAGAATCTCATACTGAGTGAAATGAG

CCAGAAAGACAAAGACAAATACCATATGACATCACTTATAACTGGAATCTAATATCCAGC

ACAAATGAACATCTCCTCAGAAAAGAAAATCAATCATGGACTTGGAGAAGAGACTTGTGG

TTGCCTGATGGGAGGGGGAGGGAGTGGGAGGGATCGGGAGCTTGGGCTTATCAGTCACAA

CGTAGAATAGATTTACAAGGAGATCCCGCTGAATAGCATTGAGAACTATGTCTAGATACT

CATGTTGCAACAGAAGAAATGGTGGGGGAAAAACTGTAATTGTAATGTATACATGTAAGG

ATAACCTGACCCCCTTGCTGTACAGTGGGAAAATAAAATTAAAAAAAAAAAAAAAAAAAA

AAAAAAAAAAAAAA

>L1D4#LINE/L1D

GAGAGGACAAGATGGCGGAGGAGTAGGGGGACACGCTCGCCCTCTCCCACAAACACAACA

AAAAAAGCACATCTACAGAAGAAATGACTCGCACAGAACAACAACCAATCGCTGGCAGAG

GAACCTAAACTCCAATAACGGCAAGAAATTCGTGACATTATTGGGCAGAACGGGAGAAAA

GAGGAGAGTGAGAGAAGGTGAATCCGAGCGGGACGGGCGCTCCCGAAAGGGAACTGCGGA

GGAGAAAGGGATCCCGCACCCTGGAAAGTCTCCTACCGGGGGAAAGATCAAACGAACCGG

AGGAATCTCCAGATGCAGAGAAGAGTGTAGCAGTAAGTCGGAGTACGGAAAAACGATCAA

GAACCCAACGGACCATCTGAACTACGGGCACAGTCACCAAAAATTGAGACGCCTGGGTGG

GGGCTGGGCACCGAATCCTCGGCTCCAGAGGTTAGTCCCCGGGAAAGGGCCGGGGGACGC

CTGGGTGGGGGCTGGGCACCGAGACCTCAGCTCTGAAGGTTGGTCCCCGAGAGGGGGCCG

GGGACGCCTGGGTTGGGGCTGGGCACCGAGACCTCGCCTCCGAAGGTTAGTCCCCAAGAA

AGGGCCGGGGGACGCCTGGGGGGGGGGGCTGGGCACCGAGACCTCGGCTCCAGAGATTAG

TCCCCGGGCTAGGGGGGCGGGGAAGAGCGGAAACTGCTTGGGAGGTCTCTAAACCATTTG

ACGGGGCAGAGACTGCCTGGGAGACTAGAAAACAAAGCTGTCGCAGAGGAAGGGAGCAAT

ACTCTAGGGGCGGGGAAGTGGAAAGCCGCCTCAGAGGGAACCTGGGAGAAGAGCCTGGTC

TGCGCCCGTGCTGGGGAGGGGAGAGAAGAAGGGGTGGGTCCCCATAGAATACCCCCCACG

CCACAACAAGCTTACAGGCCCGCTAGCTAGCAGAAAGCTGTGCTTCCCAGTGCATTCCCT

CCCCCCACCCCCGCCACCCCCTACGCTCTCGCCGAACCTGGGGCTGCCTGCCATCCAGGA

GGGCTGGCCTCAACAATTGCCTGAAGCCTACCACCGCAGGGGCTCTCCCTGCACAGGCCT

GCTTGCCCTTTGGAGGGGCTACACTTCCACAGAGCAGCACCAAACACCACCAGCCCCCTA

GAAAAGGCCTGCAGCCCAGAAAAGCTAGAACAAGCCTAGCCAGGCCGTGAATAGATCGAC

CTAATTCTCCGACGGTTTTTCTGAGACGGGCTCCCCCGGGGAGGAGCCTCTTGAGTCTCC

AAGGGCCTTGCTACACGCCCAAGACCCCAGGGGGTGCTAAGACCTAGTGGACCAGCTGCA

TAGGACTGCCAGCTCCAGGCAGGACCCCCTGCAGCCCAGAAAAGCTGCAACAAGCCTGGC

CGAATTGGGAAAAGATCTCAGACGGTCTTTCTGAGTCGGGCTGTCCTGGGGAGGAGCCTC

TTGAGTCTCCAAGGGCCCTGCTACCCGCCCAAGACCCCAAGGGGTGCTGAGACCTAGTGG

ACCAGCTGCATAGGACTGCCAGCTCCAGGCAGGACCCCCTGCAGCCCAGAAAAGCTGCAA

CAAGCCTGGCCGAATCGGGAAAAGATCTCAGACGGTCTTTCTGAGTCGGGCTGTCCTGGG

GAGGAGCCTCTTGAGCCTCCAAGGGCCCTGCTACCCGCCCAAGACCCCAGGGGGTGCTGA

GACCTAGTGGACCAGCTGCATAGGACTGCCAGCTCCAGGCAGGACCCCCTGCAGCCCAGA

AAAGCTGCAACAAGCCTGGCCGAATCGGGAAAAGATCTCAGACGGTCTTTCTGAGTCGGG

CTGTCCTGGGGAGGAGCCTCTTGAGCCTCCAAGGGCCCTGCTACCCGCCCAAGACCCCAG

GGGGTGCTGAGACCTAGTGGACCAGCTGCATAGGACTGCCAGCTCCAGGCAGGACCCCCT

GCAGCCCAGAAAAGCTGCAACAAGCCTGGCCGACTGGGAAAAGATCTCAGACGGTCTTTC

TGAGTCGGGCTGTCCTGGGGAGGAGCCTCTTGAGCCTCCAAGGGCCCTGCTACCCGCCCA

AGACCCCAGGGGGTGCTGAGACCTAGTGGACCAGCTGCATAGGACTGCCAGCTCCAGGCA

GGACCCCCTGCAGCCCAGAAAAGCTGCAACAAGCCTGGCCGAATTGGGAAAAGATCTCAG

ACGGTCTTTCTGAGTCGGGCTGTCCTGGGGAGGAGCCTCTTGAGCCTCCAAGGGCCCTGC

TACCCGCCCAAGACCCCAGGGGGTGCTGAGACCTAGTGGACCAGCTGCATAGGACTGCCA

GCTCCAGGCAGGACCCCCTGCAGCCCAGAAAAGCTGCAACAAGCCTGGCCGACTTGGGAA

AAGATCTCAGACGGTCTTTCTGAGTCGGGCTGTCCTGGGGAGGAGCCTCTTGAGCCTCCA

AGGGCCCTGCTACCCGCCCAAGACCCCAGGGGGTGCTGAGACCTAGTGGACCAGCTGCAT

AGGACTGCCAGCTCCAGGCAGGACCCCCTGCAGCCCAGAAAAGCTGCAACAAGCCTGGCC

GAATTGGGAAAAGATCTCAGACGGTCTTTCTGAGTCGGGCTGTCCTGGGGAGGAGCCTCT

TGAGTCTCCAAAGGCCCTGCTACCCGCCCAAGACCCCAAGGGGTGCTGAGACCTAGTGGA

CCAGCTGCATAGGACTGCCAGCTCCAGGCAGGACCCCCTGCAGCCCAGAAAAGCTGCAAC

AAGCCTGGCCGAATCGGGAAAAGATCTCAGACGGTCTTTCTGAGTCGGGCTGCCCTGGGG

AGGAACCTCTTGAGTCTCCAAGGGCCCTGCTACCCGCCCAAGACCCCAGGGGGTGCTGAG

AACCAAGTGAAATCTGCTACCATCGTGGGGTGGACCTCCCAGTCCTGTCTGCCCTCAGGA

AGTCCTCCTTTGCTTCAAAGAAACACTGTTAGTCCCATCAACACTCCAGAAAAGCCACAC

TGCCTCAAAAAAAGATTGACCAACAACGACAGCCCTCAGGAAATATTCCAGGGCAGTGAC

AAGGCAAACACTACCCGATAACGGAGAGTACAACTCCCTCAGGAGAAAGAAGACAACAAG

CAAGATGAAGAAGCTGAGAAACCACCCCCAGTCAAACCAACAGGAGAACTCACCTAAAAC

AGTCAACAATGAAACTGATCTCTGCAGTCTGACAGACCTGGAGTTCAAAAGAGAAATAGT

GAAAATACTGAAGGAATTAAGAGAAGATATGAACAGCAATGCAGATACCCTCAGAAAGGA

GCTAGAAAATATAAGGAGGAGCCAAGAAAAACTAGAACATTCATTTGCAGAGATGCAAAC

TGAACTAGGGGCAGTAAAAACCAGAATGAATAATGCAGAAGAACGAATCAGTGATATGGA

AGATAGAATAATGGAAATCACTCAATCTGGTCAACAGACAGAAAACCGAATCAAAAAACT

GGAAAGCAATATAAGAGACCTATGGGATAATATAAAACGGGCCAATCTACGCATAATAGG

AATTCCAGAAGGAGTAGAAAAAGATAAAGGAATGGAAAATATATTTGAAGAAATTATCGC

TGGAAACTTCCCAAATCTAAAGGATACTGGATTCAAGATACAAGAAGCACAGAGGGCCCC

AAACAAACTGAACCCAAACAGACCCACACCAAGACACATCATAATAAAAATGGCAAAAGT

TAGTGATAAAGAGAGGATCCTAAAGGCAGCAAGAGAAAAACAGAATGTTACCTACAAGGG

AACCCCCATAAGAATATCAGCTGATTTCTCTACAGAAACACTACAGGCCAGGAGGGAATG

GCAAGAGATATTTAAAGTGCTCAAAGGAAAAAATATGCAACCTAGAATACTTTATCCAGC

AAGAATATCATTTAAAATAGAAGGGGAAATAAAAATTTTTCCCAACAAACAAAAACTTAA

AGAATACAGCAACACAAAACCCAGGTTAAAGGAAATATTGAAAGGGCTTCTCTAAACCAA

AAAGAAAGGAAGGAAAGGGAAGAAAAAAGAAAAGAAAAAAAAAAAAAAAGAAGAAGAAGA

AGAAGAGGAAGAACTAGGACTGAGGAAGATACAATCAGAGAGCAGTCACTCAAATAAGCC

AGCATACAGATTTAATCATGAACATGCTTCAAACAAAATAAAATTAAAAAGAAAAAAATA

AAAGAGTCATCAAAACCATAAAATGTGGGCAAGGGATGTTAGGAGGTAAATAATCCTTTT

TGTTTGTATGTATGTCTCTCTTCTTAATTTTAATATAATAATGAAGTGTTTGAACTTACA

GGACCATCAGGCTAAAACACACAATTATGGGAAGGGGTTAGCATACTTAAAAAACAGGGC

AACCACAAGCCAAAACCAAATATTGCATTTGCAAAAAATGAAAAAAAAAAATACACTCAA

GCAGATAATAACAGGAGACCATCCAACCAAAAAAAAAAAAAAAAAAAAAAAGAAGAATGG

AGAACCATAGAATCAACTGGAACACGAGGATCAAATGGCAATAAATAATCATCTATCAAT

TATCACCTTAAATGTCAATGGACTGAATGCCCCAATCAAAAGACACAGAATGGCTGAGTG

GATAAAACGGCAAAAACCTTCAATATGCTGCCTACAAGAAACTCACCTTAGGACAAAAGA

TACATATAGATTGAAAGTGAAAGGCTGGGGAAAAGTATTTCATGCCAATAGACATGACAG

AAAAGCAGGAGTTGCAACGCTCATATCAGACAAAATAGACTTTAAAACAAAAGACATAAA

GAAAGACAAAGAAGGACACTATTTAATGATTAAGGGATCCATCCAAGGAGAGGATGTTAC

TATCATCAACATATATGCCCCAAACATAGGAGCACCCAGATACATACAACAAATATTAAC

AGACATAAAGGGAGATATTGATGAGAATACAATCATAGTAGGAGACCTAAATACCCCCCT

CACATCAATGGACAGATCCTCTAGACAGAAAACCAATAAAGCAACAGAGATCCTAAAGGA

AACAATAGAAAAGTTAGACTTAATTGATATCTTCAGGACACTACATCCAAAAAAATCAGA

ATACACATTCTTCTCAAATGCTCATGGAACATTCTCAAGAATCGACCACATATTGGGACA

TAAAGCGAATCTCAATAAATTTAGGAGCATAGAAATTATCTCAAGTATCTTCTCTGACCA

CAATGCCATGAAATTAGAAATCAACCATGGGAAAAGGAAAGAGAAAAAACCTACTCCATG

GAGACTAAACAACATGCTACTAAAAAACCAATGGGTCAATGAGGAAATCAAGAAGGAAAT

TAAAAACTATCTTGAAACAAATGATAATGAAGACACAACCTCTCAAAATCTATGGGATGC

TGCGAAAGCAGTGCTCAGAGGGAAATTTATAGCAATCCAGGCCTTTCTCAAAAAAGAAGA

AAGATCCCAAATTGACAACTTAACCCTCCACCTAAATGAATTAGAAAAAGAAGAACAAAG

AAGTCCTAAAGTCAGCAGAAGGAAGGAAATTGTAAAGATCAAAGAAGAAATCAATAAAAT

AGAGACTCAAAAAACAATAGAGAAAATTAATAAAACCAAGAGCTGGTTCTTTGAAAAGGT

GAACAAAATTGATAAACCCCTGGCCAGACTCACTAAAAAGAGGAGAGAAAGAACCCAAAT

CACCAAAATTATAAATGAAAAAGGAGAAATCACAACGGATACAGCAGAAATACAAAAAAC

CATAAGAGAATACTATGAACAACTATATGGCAATAAGTTTGACAATCTGGAAGAAATGGA

CAATTTTCTAGAATCTTACAGCCTGCCAAAACTGAATCAAGCAGAAACAGACCAACTGAA

CAGACCAATCACTAGAAATGAAATTGAAGAGGTCATAAAATCACTCCCTACAAATAAAAG

CCCAGGACCAGATGGCTTCACAGGTGAATTCTATCAAACATATAAAGAGGAATTGGTGCC

CATCCTCCTTAAACTCTTTCAAAAGGTTGAAGAAGAAGGAATACTCCCAAAGACATTCTA

TGAGGCCACCATCACCCTCATTCCAAAACCAGGCAGAGATACCACCAAAAAAGAAAACTA

TCGCCCAATATCATTGATGAATATAGATGCAAAACTTCTCAACAAAATCTTAGCCAACCG

AATCCAACAACATATCAAAAAAATTATACACCATGACCAGGTTGGGTTCATCCCAGGTTC

ACAAGGATGGTTCAACATACGCAAATCAATCAACATCATACACCACATTAACAAAAAAAA

AGTCAAAAATCATATGATCATCTCAATAGATGCAGAAAAAGCATTTGACAAAGTTCAACA

TCCATTCATGATCAAGACCCTCGCCAAAGTGGGTATAGAGGGAACATTCCTGAATATAAT

CAAAGCCATTTATGATAAACCCACAGCAAATATAATCCTCAATGGGGAAAAACTGAAAGC

CTTCTCACTCAAATCTGGAACAAGACAGGGATGCCCACTCTCACCACTGCTCTTCAACAT

AGTTTTGGAAGTCTTAGCCACAGCAATTAGACAAACAAAAGAAATCAAAGGCATCCATAT

AGGAAGAGAAGAGATCAAACTGTCACTGTATGCAGATGACATGATTCTATACCTAGAAAA

CCCTAAGGACTCAACCCCAAAACTCCTTGAACTGATTAATAAATTCAGCAAAGTGGCAGG

ATATAAGATTAACATTCAGAAGTCAGTTGCATTTCTGTATACCAGCAATGAAGCATTAGA

AAAGGAATACAAAAATACGATACCTTTTAAAATTGTACCTCACAAAATCAAATACCTCGG

AATACACCTAACCAAAGAGGTAAAGGACCTATATGCCGAGAACTATAAAACCTTAATCAA

AGAAATCAAAGAAGATGTAAAAAAATGGAAAGATATTCCATGTTCCTGGATTGGAAAAAT

CAATATTGTGAAAATGGCCATCCTACCCAAAGCAATCTACAGATTCAATGCAATCCCTAT

CAAATTACCCATGACATTTTTCACAGAACTAGAACAAACAATCCAAACATTTATATGGAA

CAACAAAAGACCCAGAATCGCCAAAGCAATCCTGAGAAACAAAAACCAAGCAGGAGGCAT

AACTCTCCCAGACTTCAAGAAATACTACAAAGCCACAGTCATCAAAACAGTGTGGTACTG

GTATCAAAACAGACAGACAGACCAATGGAACAGAATAGAGAATCTGGAAATTAACCCTGA

CACCTATGGTCAATTAATCTTTGACAAGGGAGGCAAGAACATCAAATGGGAAAAGGAAAG

TCTATTCAGCAAGCATTGCTGGGAAACCTGGACAGCTGTATGCAAAGCAATGAAACTAGA

ACACACCCTCACACCATGCACAAAAATAAACTCCAAATGGCTGAAAGACTTAAATATACG

ACAGGACACCATCAAACTCCTAGAAGAAAACATAGGCAAAACACTCTCTGACATCAACAT

CATGAATATTTTCTCAGGTCAGTCTCCCAAAGCAATAGAAACTAGAGCAAAAATAAACCC

ATGGGACCTCATCAAACTGAAAAGCTTTTGCACAGCAAAGGAAACCCAAAAGAAAACAAA

AAGACAACTTACAGAATGGGAGAAAATAGTTTCAAATGATGCAACTGACAAGGGCTTAAT

CTCTAGAATATACAAGCAACTTATACAACTCAACAGCAAAAAAACCAATCAATCAATGGA

AAAATGGGCAAAAGACCTGAATAGACATTTCTCCAAGGAAGATATACAGATGGCCAACAA

ACACATGAAAAAATGCTCAACATCGCTGATTATAAGAGAAATGCAAATCAAAACTACCAT

GAGATACCACCTCACACCAGTCAGAATGGCCATCATTAATAAATCCACAAATAACAAGTG

CTGGAGGGGCTGTGGAGAAAAGGGAACCCTCCTGCACTGTTGGTGGGAATGTAAACTGGT

ACAGCCACTATGGAGAACAGTTTGGAGATACCTTAGAAATCTATACATAGAACTTCCATA

TGACCCTGCAATCCCACTCTTGGGCATCTATCCGGACAAAGCTCTACTTAAAAGAGACAC

ATGCACCCGCATGTTCATTGCAGCACTATTCACAATAGCCAGGACATGGAAACAATCCAA

ATGTCCATCGACAGAGGATTGGATTCGGAAGATGTGGTATATATACACGATGGAATACTA

CTCAGCCATAAAAAAGGATGACATCATGCCATTTGCAGCAACATGGATGGAACTAGAGAA

TCTCATACTGAGTGAAATGAGCCAGAAAGACAAAGACAAATACCATATGACATCACTTAT

AACTGGAATCTAATATCCAGCACAAATGAACATCTCCTCAGAAAAGAAAATCAATCATGG

ACTTGGAGAAGAGACTTGTGGTTGCCTGATGGGAGGGGGAGGGAGTGGGAGGGATCGGGA

GCTTGGGCTTATCAGTCACAACGTAGAATAGATTTACAAGGAGATCCCGCTGAATAGCAT

TGAGAACTATGTCTAGATACTCATGTTGCAACAGAAGAAATGGTGGGGGAAAAACTGTAA

TTGTAATGTATACATGTAAGGATAACCTGACCCCCTTGCTGTACAGTGGGAAAATAAAAT

TTAATAAAAAAAAAAAAAAAAAAAAAAAAA

>L1D5#LINE/L1D

GAGGACAAGATGGCGGAGGAGTAGGGGGACACGCTCGCCCTCTCCCACAAACACAACAAA

AAAAGCACATCTACAGAAGAAATGACTCGCACAGAACAGCAACCAATCGCTGGCAGAGGA

ACCTAAACTCCAATAATGGCAAGAAGTTCGTGACATTATTGGGCAGAACGGGAGAAAAGA

GGAGAGTGAGAGAAGGTGAATCCGAGCGGGACGGGCGCTCCCGAAAGGGAACTGCGGAGG

AGAAAGAGATCCTGCACCCTGGAAAGTCTCCTACCGGGCGAAAGATCAAATGAACCGGAG

GAATCCCCAGATGCAGAGAAGAGTGTAGCAGTAAGTCGGAGTACGGAAAAACGGATCAAG

AACCCAACGGACCATCTGAACTACGGGCACAGTCACCAAAAATTGAGACGCCTGGGTGGG

GGCTGGGCACCGAATCCTCGGCTCCAGAGGTTAGTCCCCGGGAAAGGGCCGGGGGACGCC

TGGGTGGGGGCTGGGCACCGAGACCTCCTCTGAAGGTTGGTCCCCGAGAGGGGGCCGGGG

GACGCCTGGGTGGGGGCTGGGCACCGAGACCTCGCCTCCGAAGGTTAGTCCCCAAGAAAG

GGCCGGGGGACGCCTGGGGGGGGCTGGGCACCGAGACCTCGGCTCCAGAGATTAGTCCCC

GGGCTAGGGGGGCGGGGAAGAGCGGAAACTGCTTGGGAGGTCTCTAAACCATTTGACGGG

GCAGAGACTGCCTGGGAGACTAGAAAACAAAGCTGTCGCAGAGGAAGGGAGCAATACTCT

AGGGGCGGGGAAGTGGAAAGCCGCCTCAGAGGGAACCTGGGAGAAGAGCCTGGTCTGCGC

CCGTGCTGGGGAGCGGAGAGAAGAAGGGGTGGGTCCCCATAGAATACCCCCCACGCCACA

GCAAGCTTACAGGCCCGCTAGCTAGCAGAAAGCTGTGCTTCCCAGTGCATTCCCTCCCCC

CACCCCCGCCACCCCCTACGCTCTCGCGAACCTGGGGCTGCCTGCCATCCAGGAGGGCTG

GCCTCAACAATTGCCTGAAGCCTACCACCGCAGGGGCTCTCCCTGCACAGGCCTGCTTGC

CCTTTGGAGGGGCTACACTTCCACAGAGCAGCACCAAACACCACCAGCCCCCTAGAAAAG

GCCTGCAGCCCAGAAAAGCTAGAACAAGCCTAGCCAGGCCGTGAATAGATCGACCTAATT

CTCCGACGGTTTTTCTGAGACGGGCTCCCCCGGGGAGGAGCCTCTTGGGTCTCCAAGGGC

CTTGCTACCCGCCCAAGACCCCAGGGGGTGCTAAGACCTAGTGGACCAGCTGCATAGGAC

TGCCAGCTCCAGGCAGGACCCCCTGCAGCCCAGAAAAGCTGCAACAAGCCTGGCCGACTT

AGGAAAAGATCTCCGACGGTCTTTCTGAGTCGGGCTGCCCTGGGGAGGAGCCTCTTGAGT

CTCCAAGGGCCCTGCTACCCGCCCAAGACCCCAAGGGGTGCTGAGACCTAGTGGACCAGC

TGCATAGGACTGCCAGCTCCAGGCAGGACCCCCTGCAGCCCAGAAAAGCTGCAACAAGCC

TGGCCGATCGGGAAAAGATCTCAGACGGTCTTTCTGAGTCGGGCTGCCCTGGGGAGGAGC

CTCTTGAGTCTCCAAGGGCCCTGCTACCCGCCCAAGACCCCAGGGGGTGCTGAGACCTAG

TGGACCAGCTGCATAGGACTGCCAGCTCCAGGCAGGACCCCCTGCAGCCCAGAAAAGCTG

CAACAAGCCTGGCCGACTTGGGAAAAGATCTCAGACGGTCTTTCTGAGTCGGGCTGCCCT

GGGGAGGAGCCTCTTGAGTCTCCAAGGGCCCTGCTACCCGCCCAAGACCCCAAGGGGTGC

TGAGACCTAGTGGACCAGCTGCATAGGACTGCCAGCTCCAGGCAGGACCCCCTGCAGCCC

AGAAAAGCTGCAACAAGCCTGGCCGAATCGGGAAAAGATCTCAGACGGTCTTTCTGAGTC

GGGCTGCCCTGGGGAGGAGCCTCTTGAGTCTCCAAGGGCCCTGCTACCTGCCCAAGACCC

CAGGGGGTGCTGAGAACCAAGTGAAATCTGCTACCATCGTGGGGTGGACCTCCCAGTCCT

GTCTGCCCTCAGGAAGTCCTCCTTTGCTTCAAAGAAACACTGTTAGTCCCATCAACACTC

CAGAAAAGCCACACTGCCTCAAAAAAGATTGACCAACAACGACAGCCCTCAGGAAATATT

CCACGGCAGTGACAAGGCAAACACTACCCGATAACGGAGAGTACAACTCCCTCAGGAGAA

AGAAGACAACAAGCAAGATGAAGAAGCTGAGAAACCACCCCCAGTCAAACCAACAGGAGA

ACTCACCTAAAACAGTCAACAATGAAACAGATCTCTGCAGTCTGACAGACCTAGAATTCA

AAAGAGAAATACTGAAAATACTGAAGGAATTAAGAGAAGATATGAACAGCAATGCAGATA

CCCTCAGAAAGGAGCTAGAAAATATAAGGAGGAGCCAAGAAAAACTAGAACATTCATTTG

CAGAGATGCAAACTGAACTAGGGGCAGTAAAAACCAGAATGAATAATGCAGAAGAACGAA

TCAGTGATGTGGAAGATAGAATAATGGAAATCACTCAATCTGGTCAACAGACAGAAAACC

GAATCAAAAAACTGGAAAGCAATATAAGAGACCTATGGGATAATATAAAGCGGGCCAATC

TACGCATAATAGGAATTCCAGAAGGAGTAGAAAAAGATAAGGGAATGGAAAATATATTTG

AAGAAATTATCGCTGGAAACTTCCCAAATCTAAAGGATACTGGATTCAAGATACAAGAAG

CACAGAGGGCCCCAAACAAACTGAACCCAAACAGACCCACACCAAGACACATCATAATAA

AAATGGCAAAAGTTAGTGATAAAGAGAGGATCCTAAAGGCAGCAAGAGAAAAACAGAATG

TTACCTACAAGGGAACCCCCATAAGAATATCAGCTGATTTCTCTACAGAAACACTACAGG

CCAGGAGGGAATGGCAAGAGATATTTAAAGTGCTCAAAGGAAAAAATATGCAACCTAGAA

TACTTTATCCAGCAAGAATATCATTTAAAATAGAAGGGGAAATAAAAATTTTTCCCAACA

AACAAAAACTTAAAGAATACAGCAACACAAAACCCAGGTTAAAGGAAATATTGAAAGGGC

TTCTCTAAACCAAAAAGAAAGGAAGGAAAGGGAAGAAAAAAGAAAAGAAAAAAAAAAAAA

AAGAAGAAGAAGAAGAGGAAGAACTAGGACTGAGGAAACTGTAATCAGAGAGCAGTCACT

CAAATAAGCCAGCATACAGATTTAATCATGAACATGCTTCAAACAAAATAAAATTAAAAA

GAAAAAAATAAAAGAGTCATCAAAACCATAAAATGTGGGCAAGGGATGTTAGGAGGTAAA

TAATCCTTTTTGTTTGTATGTATGTCTCTCTTCTTAATTTTAATATAATAATGAAGTGTT

TGAACTTACAGGACCATCAGGCTAAAACACACAATTATGGGAAGGGGTTAGCATACTTAA

AAAACAGGGCAACCACAAGCCAAAACCAAATATTGCATTTGCAAAAAATGAAAAAAAAAA

ATACACTCAAGCAGATAATAACAGGAGACCATCCAACCAAAAAAAAAAAAAAAAAAAAAA

AAAGAAGAATGGAGAACCATAGAATCAACTGGAACACGAGGATCAAATGGCAATAAATAA

TCATCTATCAATTATCACCTTAAATGTCAATGGACTGAATGCCCCAATCAAAAGACACAG

AATGGCTGAGTGGATAAAACGGCAAAAACCTTCAATATGCTGCCTACAAGAAACTCACCT

TAGGACAAAAGATACATATAGATTGAAAGTGAAAGGCTGGGGAAAAGTATTTCATGCCAA

TAGACATGACAGAAAAGCAGGAGTTGCAACGCTCATATCAGACAAAATAGACTTTAAAAC

AAAAGACATAAAGAAAGACAAAGAAGGACACTATTTAATGATTAAGGGATCCATCCAAGG

AGAGGATGTTACTATCATCAACATATATGCCCCAAACATAGGAGCACCCAGATACATACA

ACAAATATTAACAGACATAAAGGGAGATATTGATGAGAATACAATCATAGTAGGAGACCT

AAATACCCCCCTCACATCAATGGACAGATCCTCTAGACAGAAAACCAATAAAGCAACAGA

GATCCTAAAGGAAACAATAGAAAAGTTAGACTTAATTGATATCTTCAGGACACTACATCC

AAAAAAATCAGAATACACATTCTTCTCAAATGCTCATGGAACATTCTCAAGAATCGACCA

CATATTGGGACACAAAGCGAATCTCAATAAATTTAGGAGCATAGAAATTATCTCAAGTAT

CTTCTCTGACCACAATGCCATGAAATTAGAAATCAACCATGGGAAAAGGAAAGAGAAAAA

ACCTACTCCATGGAGACTAAACAACATGCTACTAAAAAACCAATGGGTCAATGAGGAAAT

CAAGAAGGAAATTAAAAACTACCTTGAAACAAATGATAATGAAGACACAACCTCTCAAAA

TCTATGGGATGCTGCGAAAGCAGTGCTCAGAGGGAAATTTATAGCAATCCAGGCCTTTCT

CAAAAAAGAAGAAAGATCCCAAATTGACAACTTAACCCTCCACCTAAATGAATTAGAAAA

AGAAGAACAAAAAAGTCCTAAAGTCAGCAGAAGGAAGGAAATTATAAAGATCAAAGAAGA

AATCAATAAAATAGAGACTCAAAAAACAATAGAGAAAATTAATAAAACCAAGAGCTGGTT

CTTTGAAAAGGTGAACAAAATTGATAAACCCCTGGCCAGACTCACTAAAAAGAGGAGAGA

AAGAACCCAAATCACCAAAATTATAAATGAAAAAGGAGAAATCACAACGGATACAGCAGA

AATACAAAAAACCATAAGAGAATACTATGAACAACTATATGGCAATAAGTTTGACAATCT

GGAAGAAATGGACAATTTTCTAGAATCTTACAGCCTGCCAAAACTGAATCAAGCAGAAAC

AGACCAACTGAACAGACCATCACTAGAAATGAAATTGAAGAGGTCATAAAATCACTCCCT

ACAAATAAAAGCCCAGGACCAGATGGCTTCACAGGTGAATTCTATCAAACATATAAAGAG

GAATTGGTGCCCATCCTCCTTAAACTCTTTCAAAAGGTTGAAGAAGAAGGAATACTCCCA

AAGACATTCTATGAGGCCACCATCACCCTCATTCCAAAACCAGGCAGAGATACCACCAAA

AAAGAAAACTATCGCCCAATATCATTGATGAATATAGATGCAAAAATTCTCAACAAAATC

TTAGCCAACCGAATCCAACAACATATCAAAAAAATTATACACCATGACCAGGTTGGGTTC

ATCCCAGGTTCACAAGGATGGTTCAACATACGCAAATCAATCAACATCATACACCACATT

AACAAAAAAAAAGTCAAAAATCATATGATCATCTCAATAGATGCAGAAAAAGCATTTGAC

AAAGTTCAACATCCATTCATGATCAAGACCCTCGCCAAAGTGGGTATAGAGGGAACATTC

CTGAATATAATCAAAGCCATTTATGATAAACCCACAGCAAATATAATCCTCAATGGGGAA

AAACTGAAAGCCTTCTCACTCAAATCTGGAACAAGACAGGGATGCCCACTCTCACCACTG

CTCTTCAACATGTTTTGGAAGTCTTAGCCACAGCAATTAGACAAACAAAAGAAATCAAAG

GCATCCATATAGGAAGAGAAGAGATCAAACTGTCACTGTATGCAGATGACATGATTCTAT

ACCTAGAAAACCCTAAGGACTCAACCCCAAAACTCCTTGAACTGATTAATAAATTCAGCA

AAGTGGCAGGATATAAGATTAACATTCAGAAGTCAGTTGCATTTCTGTATACCAGCAATG

AAACATTAGAAAAGGAATACAAAAATACGATACCTTTTAAAATTGTACCCACAAAATCAA

ATACCTCGGAATACACCTGACCAAAGAGGTAAAGGACCTATATGCCGAGAACTATAAAAC

CTTAATCAAAGAAATCAAAGAAGATGTAAAAAATGGAAAGATATTCCATGTTCCTGGATT

GGAAAAATCAATATTGTGAAAATGGCCATCCTACCCAAAGCAATCTACAGATTCAATGCA

ATCCCTATCAAATTACCCATGACATTTTTCACAGAACTAGAACAAACAATCCAAACATTT

ATATGGAACAACAAAAGACCCAGAATCGCCAAAGCAATCCTGAGAAACAAAAACCAAGCA

GGAGGCATAACTCTCCCAGACTTCAAGAAATACTACAAAGCCACAGTCATCAAAACAGTG

TGGTACTGGTATCAAAACAGACAGACAGACCAATGGAACAGAATAGAGAATCGGAAATTA

ACCCTGACACCTATGGTCAATTAATCTTTGACAAGGGAGGCAAGAACATAAAATGGGAAA

AGAAAGTCTATTCAGCAAGCATTGCTGGGAAACCTGGACAGCTGCATGCAAAGCAATGAA

ACTAGAACACACCCTCACACCATGCACAAAAATAAACTCCAAATGGCTGAAAGACTTAAA

TATACGACAGGACACCATCAAACTCCTAGAAGAAAACATAGGCAAAACACTCTCTGACAT

CAACATCATGAATATTTTCTCAGGTCAGTCTCCCAAAGCAATAGAAATTAGAGCAAAAAT

AAACCCATGGGACCTCATCAAACTGAAAAGCTTTTGCACAGCAAAGGAAACCCAAAAGAA

AACAAAAAGACAACTTACAGAATGGGAGAAAATAGTTTCAAATGATGCAACTGACAAGGG

CTTAATCTCTAGAATATATAAGCAACTTATACAACTCAACAGCAAAAAAACCAATCAATC

AATGGAAAAATGGGCAAAAGACCTGAATAGACATTTCTCCAAAGAAGATATACAGATGGC

CAACAAACACATGAAAAAATGCTCCACATCGCTGATTATAAGAGAAATGCAAATCAAAAC

TACCATGAGATACCACCTCACACCAGTCAGAATGGCCATCATTAATAAATCCACAAATAA

CAAGTGCTGGAGGGGCTGTGGAGAAAAGGGAACCCTCCTGCACTGTTGGTGGGAATGTAA

ACTGGTACAGCCACTATGGAGAACAGTTTGGAGATACCTTAGAAATCTATACATAGACTT

CCATATGACCCTGCAATCCCACTCTTGGGCATCTATCCGGACAAAGCTCTACTTAAAAGA

GACACATGCACCCGCATGTTCATTGCAGCACTATTCACAATAGCCAGGACATGGAAACAA

TCCAAATGTCCATCGACAGAGGATTGGATTCGGAAGATGTGGTATATATACACGATGGAA

TACTACTCAGCCATAAAAAAGGATGACATCATGCCATTTGCAGCAACATGGATGGAACTA

GAGAATCTCATACTGAGTGAAATGAGCCAGAAAGACAAAGACAAATACCATATGATATCA

CTTATAACTGGAATCTAATATCCAGCACAAATGAACATCTCCTCAGAAAAGAAAATCATG

GACTTGGAGAAGAGACTTGTGGTTGCCTGATGGGAGGGGGAGGGAGTGGGAGGGATCGGG

AGCTTGGGCTTATCAGTCACAACCTAGAATAGATTTACAAGGAGATCCCGCTGAATAGCA

TTGAGAACTATGTCTAGATACTCATGTTGCAACAGAAGAAATGGTGGGGGAAAAACTGTA

ATTGTAATGTATACATGTAAGGATAACCTGACCCCCTTGCTGTACAGTGGGAAAATAAAA

TTAAAAAAATAAAAAAAAAAAAAAAAAAAAAAAAAAAA

>L1D6#LINE/L1D

GGAGAGAGGACAAGATGGCGGAGGAGTAGGGGGACACGCTCGCCCTCTCCCACAAACACA

ACAAAAAAAGCACATCTACAGAAGAAATGACTCGCACAGAACAACAACCAATCGCTGGCA

GAGGAACCTAAACTCCAATAACGGCAAGAAATTCGTGACATTATTGGGCAGAACGGGAGA

AAAGAGGAGAGTGAGAGAAGGTGAATCCGAGCGGGACGGGCGCTCCCGAAAGGGAACTGC

GGAGGAGAAAGGGATCCCGCACCCTGGAAAGTCTCCTACCGGGGGAAAGATCAAACGAAC

CGGAGGAATCTCCAGATGCAGAGAAGAGTGTAGCAGTAAGTCGGAGTACGGAAAAACGAT

CAAGAACCCAACGGACCATCTGAACTACGGGCACAGTCACCAAAAATTGAGACGCCTGGG

TGGGGGCTGGGCACCGAATCCTCGGCTCCAGAGGTTAGTCCCCGGGAAAGGGCCGGGGGA

CGCCTGGGTGGGGGCTGGGCACCGAGACCTCAGCTCTGAAGGTTGGTCCCCGAGAGGGGG

CCGGGGGACGCCTGGGTTGGGGCTGGGCACCGAGACCTCGCCTCCGAAGGTTAGTCCCCA

AGAAAGGGCCGGGGGACGCCTGGGGGGGGGGGCTGGGCACCGAGACCTCGGCTCCAGAGA

TTAGTCCCCGGGCTAGGGGGGCGGGGAAGAGCGGAAACTGCTTGGGAGGTCTCTAAACCA

TTTGACGGGGCAGAGACTGCCTGGGAGACTAGAAAACAAAGCTGTCGCAGAGGAAGGGAG

CAATACTCTAGGGGCGGGGAAGTGGAAAGCCGCCTCAGAGGGAACCTGGGAGAAGAGCCT

GGTCTGCGCCCGTGCTGGGGAGGGGAGAGAAGAAGGGGTGGGTCCCCATAGAATACCCCC

CACGCCACAACAAGCTTACAGGCCCGCTAGCTAGCAGAAAGCTGTGCTTCCCAGTGCATT

CCCTCCCCCCACCCCCGCCACCCCCTACGCTCTCGCCGAACCTGGGGCTGCCTGCCATCC

AGGAGGGCTGGCCTCAACAATTGCCTGAAGCCTACCACCGCAGGGGCTCTCCCTGCACAG

GCCTGCTTGCCCTTTGGAGGGGCTACACTTCCACAGAGCAGCACCAAACACCACCAGCCC

CCTAAAAAGGCCTGCAGCCCAGAAAAGCTAGAACAAGCCTAGCCAGGCCGTGAATAGATC

GACCTAATTCTCCGACGGTTTTTCTGAGACGGGCTCCCCCGGGGAGGAGCCTCTTGAGTC

TCCAAGGGCCTTGCTACACGCCCAAGACCCCAGGGGGTGCTAAGACCTAGTGGACCAGCT

GCATAGGACTGCCAGCTCCAGGCAGGACCCCCTGCAGCCCAGAAAAGCTGCAACAAGCCT

GGCCGAATGGGAAAAGATCTCAGACGGTCTTTCGAGTCGGGCTGTCCTGGGGAGGAGCCT

CTTGAGCTCCAAGGGCCCTGCTACCCGCCCAAGACCCCAGGGGTGCTGAGACCTAGTGGA

CCAGCTGCATAGGACTGCCAGCTCCAGGCAGGACCCCCTGCAGCCCAGAAAAGCTGCAAC

AAGCCTGGCCGAATCGGGAAAAGATCTCAGACGGTCTTTCTGAGTCGGGCTGTCCTGGGG

AGGAGCCTCTTGAGCCTCCAAGGGCCCTGCTACCCGCCCAAGACCCCAGGGGGTGCTGAG

ACCTAGTGGACCAGCTGCATAGGACTGCCAGCTCCAGGCAGGACCCCCTGCAGCCCAGAA

AAGCTGCAACAAGCCTGGCCGAATCGGGAAAAGATCTCAGACGGTCTTTCTGAGTCGGGC

TGTCCTGGGGAGGAGCCTCTTGAGCCTCCAAGGGCCCTGCTACCCGCCCAAGACCCCAGG

GGGTGCTGAGACCTAGTGGACCAGCTGCATAGGACTGCCAGCTCCAGGCAGGACCCCCTG

CAGCCCAGAAAAGCTGCAACAAGCCTGGCCGAATCGGGAAAAGATCTCAGACGGTCTTTC

TGAGTCGGGCTGTCCTGGGGAGGAGCCTCTTGAGCCTCCAAGGGCCCTGCTACCCGCCCA

AGACCCCAGGGGGTGCTGAGACCTAGTGGACCAGCTGCATAGGACTGCCAGCTCCAGGCA

GGACCCCCTGCAGCCCAGAAAAGCTGCAACAAGCCTGGCCGACTTGGGAAAAGATCTCAG

ACGGTCTTTCTGAGTCGGGCTGTCCTGGGGAGGAGCCTCTTGAGTCTCCAAGGGCCCTGC

TACCCGCCCAAGACCCCAGGGGGTGCTGAGACCTAGTGGACCAGCTGCATAGGACTGCCA

GCTCCAGGCAGGACCCCCTGCAGCCCAGAAAAGCTGCAACAAGCCTGGCCGAATCGGGAA

AAGATCTCAGACGGTCTTTCTGAGTCGGGCTGTCCTGGGGAGGAGCCTCTTGAGCCTCCA

AGGGCCCTGCTACCCGCCCAAGACCCCAGGGGGTGCTGAGACCTAGTGGACCAGCTGCAT

AGGACTGCCAGCTCCAGGCAGGACCCCCTGCAGCCCAGAAAAGCTGCAACAAGCCTGGCC

GACTTGGGAAAAGATCTCAGACGGTCTTTCTGAGTCGGGCTGTCCTGGGGAGGAGCCTCT

TGAGCCTCCAAGGGCCCTGCTACCCGCCCAAGACCCCAGGGGGTGCTGAGACCTAGTGGA

CCAGCTGCATAGGACTGCCAGCTCCAGGCAGGACCCCCTGCAGCCCAGAAAAGCTGCAAC

AAGCCTGGCCGACTTGGGAAAAGATCTCAGACGGTCTTTCTGAGTCGGGCTGTTCTGGGG

AGGAGCCTCTTGAGTCTCCAAAGGCCCTGCTACCCGCCCAAGACCCCAAGGGGTGCTGAG

ACCTAGTGGACCAGCTGCATAGGACTGCCAGCTCCAGGCAGGACCCCCTGCAGCCCAGAA

AAGCTGCAACAAGCCTGGCGGAATCGGGAAAAGATCTCAGACGGTCTTTCTGAGTCGGGC

TGCCCTGGGGAGGAACCTCTTGAGTCTCCAAGGGCCCTGCTACCCGCCCAAGACCCCAGG

GGGTGCTGAGAACCAAGTGAAATCTGCTACCATCGTGGGGTGGACCTCCCAGTCCTGTCT

GCCCTCAGGAAGTCCTCCTTTGCTTCAAAGAAACACTGTTAGTCCCATCAACACTCCAGA

AAAGCCACACTGCCTCAAAAAAAGATTGACCAACAACGACAGCCCTCAGGAAATATTCCA

GGGCAGTGACAAGGCAAACACTACCCGATAACGGAGAGTACAACTCCCTCAGGAGAAAGA

AGACAACAAGCAAGATGAAGAAGCTGAGAAACCACCCCCAGTCAAACCAACAGGAGAACT

CACCTAAAACAGTCAACAATGAAACTGATCTCTGCAGTCTGACAGACCTGGAGTTCAAAA

GAGAAATAGTGAAAATACTGAAGGAATTAAGAGAAGATATGAACAGCAATGCAGATACCC

TCAGAAAGGAGCTAGAAAATATAAGGAGGAGCCAAGAAAAACTAGAACATTCATTTGCAG

AGATGCAAACTGAACTAGGGGCAGTAAAAACCAGAATGAATAATGCAGAAGAACGAATCA

GTGATATGGAAGATAGAATAATGGAAATCACTCAATCTGGTCAACAGACAGAAAACCGAA

TCAAAAAACTGGAAAGCAATATAAGAGACCTATGGGATAATATAAAACGGGCCAATCTAC

GCATAATAGGAATTCCAGAAGGAGTAGAAAAAGATAAAGGAATGGAAAATATATTTGAAG

AAATTATCGCTGGAAACTTCCCAAATCTAAAGGATACTGGATTCAAGATACAAGAAGCAC

AGAGGGCCCCAAACAAACTGAACCCAAACAGACCCACACCAAGACACATCATAATAAAAA

TGGCAAAAGTTAGTGATAAAGAGAGGATCCTAAAGGCAGCAAGAGAAAAACAGAATGTTA

CCTACAAGGGAACCCCCATAAGAATATCAGCTGATTTCTCTACAGAAACACTACAGGCCA

GGAGGGAATGGCAAGAGATATTTAAAGTGCTCAAAGGAAAAAATATGCAACCTAGAATAC

TTTATCCAGCAAGAATATCATTTAAAATAGAAGGGGAAATAAAAATTTTTCCCAACAAAC

AAAAACTTAAAGAATACAGCAACACAAAACCCAGGTTAAAGGAAATATTGAAAGGGCTTC

TCTAAACCAAAAAGAAAGGAAGGAAAGGGAAGAAAAAAGAAAAGAAAAAAAAAAAAAAAA

GAAGAAGAAGAAGAAGAGGAAGAACTAGGACTGAGGAAGATACAATCAGAGAGCAGTCAC

TCAAATAAGCCAGCATACAGATTTAATCATGAACATGCTTCAAACAAAATAAAATTAAAA

AGAAAAAAATAAAAGAGTCATCAAAACCATAAAATGTGGGCAAGGGATGTTAGGAGGTAA

ATAATCCTTTTTGTTTGTATGTATGTCTCTCTTCTTAATTTTAATATAATAATGAAGTGT

TTGAACTTACAGGACCATCAGGCTAAAACACACAATTATGGGAAGGGGTTAGCATACTTA

AAAAACAGGGCAACCACAAGCCAAAACCAAATATTGCATTTGCAAAAAATGAAAAAAAAA

AATACACTCAAGCAGATAATAACAGGAGACCATCCAACCAAAAAAAAAAAAAAAAAAAAA

AAAGAAGAATGGAGAACCATAGAATCAACTGGAACACGAGGATCAAATGGCAATAAATAA

TCATCTATCAATTATCACCTTAAATGTCAATGGACTGAATGCCCCAATCAAAAGACACAG

AATGGCTGAGTGGATAAAACGGCAAAAACCTTCAATATGCTGCCTACAAGAAACTCACCT

TAGGACAAAAGATACATATAGATTGAAAGTGAAAGGCTGGGGAAAAGTATTTCATGCCAA

TAGACATGACAGAAAAGCAGGAGTTGCAACGCTCATATCAGACAAAATAGACTTTAAAAC

AAAAGACATAAAGAAAGACAAAGAAGGACACTATTTAATGATTAAGGGATCCATCCAAGG

AGAGGATGTTACTATCATCAACATATATGCCCCAAACATAGGAGCACCCAGATACATACA

ACAAATATTAACAGACATAAAGGGAGATATTGATGAGAATACAATCATAGTAGGAGACCT

AAATACCCCCCTCACATCAATGGACAGATCCTCTAGACAGAAAACCAATAAAGCAACAGA

GATCCTAAAGGAAACAATAGAAAAGTTAGACTTAATTGATATCTTCAGGACACTACATCC

AAAAAAATCAGAATACACATTCTTCTCAAATGCTCATGGAACATTCTCAAGAATCGACCA

CATATTGGGACATAAAGCGAATCTCAATAAATTTAGGAGCATAGAAATTATCTCAAGTAT

CTTCTCTGACCACAATGCCATGAAATTAGAAATCAACCATGGGAAAAGGAAAGAGAAAAA

ACCTACTCCATGGAGACTAAACAACATGCTACTAAAAAACCAATGGGTCAATGAGGAAAT

CAAGAAGGAAATTAAAAACTATCTTGAAACAAATGATAATGAAGACACAACCTCTCAAAA

TCTATGGGATGCTGCGAAAGCAGTGCTCAGAGGGAAATTTATAGCAATCCAGGCCTTTCT

CAAAAAAGAAGAAAGATCCCAAATTGACAACTTAACCCTCCACCTAAATGAATTAGAAAA

AGAAGAACAAAGAAGTCCTAAAGTCAGCAGAAGGAAGGAAATTGTAAAGATCAAAGAAGA

AATCAATAAAATAGAGACTCAAAAAACAATAGAGAAAATTAATAAAACCAAGAGCTGGTT

CTTTGAAAAGGTGAACAAAATTGATAAACCCCTGGCCAGACTCACTAAAAAGAGGAGAGA

AAGAACCCAAATCACCAAAATTATAAATGAAAAAGGAGAAATCACAACGGATACAGCAGA

AATACAAAAAACCATAAGAGAATACTATGAACAACTATATGGCAATAAGTTTGACAATCT

GGAAGAAATGGACAATTTTCTAGAATCTTACAGCCTGCCAAAACTGAATCAAGCAGAAAC

AGACCAACTGAACAGACCAATCACTAGAAATGAAATTGAAGAGGTCATAAAATCACTCCC

TACAAATAAAAGCCCAGGACCAGATGGCTTCACAGGTGAATTCTATCAAACATATAAAGA

GGAATTGGTGCCCATCCTCCTTAAACTCTTTCAAAAGGTTGAAGAAGAAGGAATACTCCC

AAAGACATTCTATGAGGCCACCATCACCCTCATTCCAAAACCAGGCAGAGATACCACCAA

AAAAGAAAACTATCGCCCAATATCATTGATGAATATAGATGCAAAACTTCTCAACAAAAT

CTTAGCCAACCGAATCCAACAACATATCAAAAAAATTATACACCATGACCAGGTTGGGTT

CATCCCAGGTTCACAAGGATGGTTCAACATACGCAAATCAATCAACATCATACACCACAT

TAACAAAAAAAAAGTCAAAAATCATATGATCATCTCAATAGATGCAGAAAAAGCATTTGA

CAAAGTTCAACATCCATTCATGATCAAGACCCTCGCCAAAGTGGGTATAGAGGGAACATT

CCTGAATATAATCAAAGCCATTTATGATAAACCCACAGCAAATATAATCCTCAATGGGGA

AAAACTGAAAGCCTTCTCACTCAAATCTGGAACAAGACAGGGATGCCCACTCTCACCACT

GCTCTTCAACATAGTTTTGGAAGTCTTAGCCACAGCAATTAGACAAACAAAAGAAATCAA

AGGCATCCATATAGGAAGAGAAGAGATCAAACTGTCACTGTATGCAGATGACATGATTCT

ATACCTAGAAAACCCTAAGGACTCAACCCCAAAACTCCTTGAACTGATTAATAAATTCAG

CAAAGTGGCAGGATATAAGATTAACATTCAGAAGTCAGTTGCATTTCTGTATACCAGCAA

TGAAGCATTAGAAAAGGAATACAAAAATACGATACCTTTTAAAATTGTACCTCACAAAAT

CAAATACCTCGGAATACACCTAACCAAAGAGGTAAAGGACCTATATGCCGAGAACTATAA

AACCTTAATCAAAGAAATCAAAGAAGATGTAAAAAAATGGAAAGATATTCCATGTTCCTG

GATTGGAAAAATCAATATTGTGAAAATGGCCATCCTACCCAAAGCAATCTACAGATTCAA

TGCAATCCCTATCAAATTACCCATGACATTTTTCACAGAACTAGAACAAACAATCCAAAC

ATTTATATGGAACAACAAAAGACCCAGAATCGCCAAAGCAATCCTGAGAAACAAAAACCA

AGCAGGAGGCATAACTCTCCCAGACTTCAAGAAATACTACAAAGCCACAGTCATCAAAAC

AGTGTGGTACTGGTATCAAAACAGACAGACAGACCAATGGAACAGAATAGAGAATCTGGA

AATTAACCCTGACACCTATGGTCAATTAATCTTTGACAAGGGAGGCAAGAACATCAAATG

GGAAAAGGAAAGTCTATTCAGCAAGCATTGCTGGGAAACCTGGACAGCTGTATGCAAAGC

AATGAAACTAGAACACACCCTCACACCATGCACAAAAATAAACTCCAAATGGCTGAAAGA

CTTAAATATACGACAGGACACCATCAAACTCCTAGAAGAAAACATAGGCAAAACACTCTC

TGACATCAACATCATGAATATTTTCTCAGGTCAGTCTCCCAAAGCAATAGAAACTAGAGC

AAAAATAAACCCATGGGACCTCATCAAACTGAAAAGCTTTTGCACAGCAAAGGAAACCCA

AAAGAAAACAAAAAGACAACTTACAGAATGGGAGAAAATAGTTTCAAATGATGCAACTGA

CAAGGGCTTAATCTCTAGAATATACAAGCAACTTATACAACTCAACAGCAAAAAAACCAA

TCAATCAATGGAAAAATGGGCAAAAGACCTGAATAGACATTTCTCCAAGGAAGATATACA

GATGGCCAACAAACACATGAAAAAATGCTCAACATCGCTGATTATAAGAGAAATGCAAAT

CAAAACTACCATGAGATACCACCTCACACCAGTCAGAATGGCCATCATTAATAAATCCAC

AAATAACAAGTGCTGGAGGGGCTGTGGAGAAAAGGGAACCCTCCTGCACTGTTGGTGGGA

ATGTAAACTGGTACAGCCACTATGGAGAACAGTTTGGAGATACCTTAGAAATCTATACAT

AGAACTTCCATATGACCCTGCAATCCCACTCTTGGGCATCTATCCGGACAAAGCTCTACT

TAAAAGAGACACATGCACCCGCATGTTCATTGCAGCACTATTCACAATAGCCAGGACATG

GAAACAATCCAAATGTCCATCGACAGAGGATTGGATTCGGAAGATGTGGTATATATACAC

GATGGAATACTACTCAGCCATAAAAAAGGATGACATCATGCCATTTGCAGCAACATGGAT

GGAACTAGAGAATCTCATACTGAGTGAAATGAGCCAGAAAGACAAAGACAAATACCATAT

GACATCACTTATAACTGGAATCTAATATCCAGCACAAATGAACATCTCCTCAGAAAAGAA

AATCAATCATGGACTTGGAGAAGAGACTTGTGGTTGCCTGATGGGAGGGGGAGGGAGTGG

GAGGGATCGGGAGCTTGGGCTTATCAGTCACAACGTAGAATAGATTTACAAGGAGATCCC

GCTGAATAGCATTGAGAACTATGTCTAGATACTCATGTTGCAACAGAAGAAATGGTGGGG

GAAAAACTGTAATTGTAATGTATACATGTAAGGATAACCTGACCCCCTTGCTGTACAGTG

GGAAAATAAAATTTAATAAAAAAAAAAAAAAAAAAAAAAAAAAAAAAAAAAAAAAAAAAA

AA

>L1D7#LINE/L1D

GACAAGATGGCGGAGGAGTAGGGGGACACGCTCGCCCTCTCCCACAAACACAACAAAAAA

AGCACATCTACAGAAGAAATGACTCGCACAGAACAGCAACCAATCGCTGGCAGAGGAACC

TAAACTCCAATAACGGCAAGAAGTTCGTGACATTATTGGGCAGAACGGGAGAAAAGAGGA

GAGTGAGAGAAGGTGAATCCGAGCGGGACGGGCGCTCCCGAAAGGGAACTGCGGAGGAGA

AAGGGATCCCGCACCCTGGAAAGTCTCCTACCGGGCGAAAGATCAAATGAACCGGAGGAA

TCCCCAGATGCAGAGAAGAGTGTAGCAGTAAGTCGGAGTACGGAAAAACGGATCAAGAAC

CCAACGGACCATCTGAACTACGGGCACAGTCACCAAAAATTGAGACGCCTGGGTGGGGGC

TGGGCACCGAATCCTCGGCTCCAGAGGTTAGTCCCCGGGAAAGGGCCGGGGGACGCCTGG

GTGGGGGCTGGGCACCGAGACCTCGCCTCTGAAGGTTGGTCCCCGAGAGGGGGCCGGGGG

ACGCCTGGGTGGGGCTGGGCACCGAGACCTCGCCTCCGAAGGTTAGTCCCCAAGAAAGGG

CCGGGGGACGCCGGGGGGGGGCTGGGCACCGAGACCTCGGCTCCAGAGATTAGTCCCCGG

GCTAGGGGGGCGGGGAAGAGCGGAAACTGCTTGGGAGGTCTCTAAACCATTTGACGGGGC

AGAGACTGCCTGGGAGACTAGAAAACAAAGCTGTCGCAGAGGAAGGGAGCAATACTCTAG

GGGCGGGGAAGTGGAAAGCCGCCTCAGAGGGAACCTGGGAGAAGAGCCTGGTCTGCGCCC

GTGCTGGGGAGGGGAGAGAAGAAGGGGTGGGTCCCCATAGAATACCCCCCACGCCACAGC

AAGCTTACAGGCCCGCTAGCTAGCAGAAAGCTGTGCTTCCCAGTGCATTCCCTCCCCCCA

CCCCCGCCACCCCCTACGCTCTCGCCGAACCTGGGGCTGCCTGCCATCCAGGAGGGCTGG

CCTCAACAATTGCCTGAAGCCTACCACCGCAGGGGCTCTCCCTGCACAGGCCTGCTTGCC

CTTTGGAGGGGCTACACTTCCACAGAGCAGCACCAAACACCACCAGCCCCCTAGAAAAGG

CCTGCAGCCCAGAAAAGCTAGAACAAGCCTAGCCAGGCCGTGAATAGATCGACCTAATTC

TCCGACGGTTTTTCTGAGACGGGCTCCCCCGGGGAGGAGCCTCTTGGGTCTCCAAGGGCC

TTGCTACCCGCCCAAGACCCCAGGGGGTGCTAAGACCTAGTGGACCAGCTGCATAGGACT

GCCAGCTCCAGGCAGGACCCCCTGCAGCCCAGAAAAGCTGCAACAAGCCTGGCCGATTGG

GAAAAGATCTCAGACGGTCTTTCTGAGTCGGGCTGCCCTGGGGAGGAGCCTCTTGAGTCT

CCAAGGGCCCTGCTACCCGCCCAAGACCCCAAGGGGTGCTGAGACCTAGTGGACCAGCTG

CATAGGACTGCCAGCTCCAGGCAGGACCCCCTGCAGCCCAGAAAAGCTGCAACAAGCCTG

GCCGAATCGGGAAAAGATCTCAGACGGTCTTTCTGAGTCGGGCTGCCCTGGGGAGGAGCC

TCTTGAGTCTCCAAGGGCCCTGCTACCCGCCCAAGACCCCAGGGGGTGCTGAGAACCAAG

TGAAATCTGCTACCATCGTGGGGTGGACCTCCCAGTCCTGTCTGCCCTCAGGAAGTCCTC

CTTTGCTTCAAAGAAACACTGTTAGTCCCATCAACACTCCAGAAAAGCCACACTGCCTCA

AAAAAGATTGACCAACAACGACAGCCCTCAGGAAATATTCCACGGCAGTGACAAGGCAAA

CACTACCCGATAACGGAGAGTACAACTCCCTCAGGAGAAAGAAGACAACAAGCAAGATGA

AGAAGCTGAGAAACCACCCCCAGTCAAACCAACAGGAGAACTCACCTAAAACAGTCAACA

ATGAAACAGATCTCTGCAGTCAGACAGACCTGGAATTCAAAAGAGAAATACTGAAAATAC

TGAAGGAATTAAGAGAAGATATGAACAGTAATGCAGATACCCTCAGAAAGGAGCTAGAAA

ATATAAGGAGGAGCCAAGAAAAACTAGAACATTCATTTGCAGAGATGCAAACTGAACTAG

GGGCAGTAAAAACCAGAATGAATAATGCAGAAGAACGAATCAGTGATATGGAAGATAGAA

TAATGGAAATCACTCAATCTGGTCAACAGACAGAAAACCGAATCAAAAAACTGGAAAGCA

ATATAAGAGACCTATGGGATAATATAAAGCGGGCCAATCTACGCATAATAGGAATTCCAG

AAGGAGTAGAAAAAGATAAGGGAATGGAAAATATATTTGAAGAAATTATCGCTGGAAACT

TCCCAAATCTAAAGGATACTGGATTCAAGATACAAGAAGCACAGAGGGCCCCAAACAAAC

TGAACCCAAACAGACCCACACCAAGACACATCATAATAAAAATGGCAAAAGTTAGTGATA

AAGAGAGGATCCTAAAGGCAGCAAGAGAAAAACAGAATGTTACCTACAAGGGAACCCCCA

TAAGAATATCAGCTGATTTCTCTACAGAAACACTACAGGCCAGGAGGGAATGGCAAGAGA

TATTTAAAGTGCTCAAAGGAAAAAATATGCAACCTAGAATACTTTATCCAGCAAGAATAT

CATTTAAAATAGAAGGGGAAATAAAAATTTTTCCCAACAAACAAAAACTTAAAGAATACA

GCAACACAAAACCCAGGTTAAAGGAAATATTGAAAGGGCTTCTCTAAACCAAAAAGAAAG

GAAGGAAAGGGAAGAAAAAAGAAAAGAAAAAAAAAAAAAGAAGAAGAAGAAGAGGAAGAA

CTAGGACTGAGGAAACCGCAATCAGAGAGCAGTCACTCAAATAAGCCAGCATACAGATTT

AATCATGAACATGCTTCAAACAAAATAAAATTAAAAAGAAAAAAATAAAAGAGTCATCAA

AACCATAAAATGTGGGCAAGGGATGTTAGGAGGTAAATAATCCTTTTTGTTTGTATGTAT

GTCTCTCTTCTTAATTTTAATATAATAATGAAGTGTTTGAACTTACAGGACCATCAGGCT

AAAACACACAATTATGGGAAGGGGTTAGCATACTTAAAAAACAGGGCAACCACAAGCCAA

AACCAAATATTGCATTTGCAAAAAATGAAAAAAAAAATACACTCAAGCAGATAATAACAG

GAGACCATCCAACCAAAAAAAAAAAAAAAAAAAAAAAAAAAGAAGAATGGAGAACCATAG

AATCAACTGGAACACGAGGATCAAATGGCAATAAATAATCATCTATCAATTATCACCTTA

AATGTCAATGGACTGAATGCCCCAATCAAAAGACACAGAATGGCTGAGTGGATAAAAAGG

CAAAAACCTTCAATATGCTGCCTACAAGAAACTCACCTTAGGACAAAAGATACATATAGA

TTGAAAGTGAAAGGCTGGGGAAAAGTATTTCATGCCAATAGACATGACAGAAAAGCAGGA

GTTGCAACGCTCATATCAGACAAAATAGACTTTAAAACAAAAGACATAAAGAAAGACAAA

GAAGGACACTATTTAATGATTAAGGGATCCATCCAAGGAGAGGATGTTACTATCATCAAC

ATATATGCCCCAAACATAGGAGCACCCAGATACATACAACAAATATTAACAGACATAAAG

GGAGATATTGATGAGAATACAATCATAGTAGGAGACCTAAATACCCCCCTCACATCAATG

GACAGATCCTCTAGACAGAAAACCAATAAAGCAACAGAGATCCTAAAGGAAACAATAGAA

AAGTTAGACTTAATTGATATCTTCAGGACACTACATCCAAAAAAATCAGAATACACATTC

TTCTCAAATGCTCATGGAACATTCTCAAGAATCGACCACATATTGGGACATAAAGCGAAT

CTCAATAAATTTAGGAGCATAGAAATTATCTCAAGTATCTTCTCTGACCACAATGCCATG

AAATTAGAAATCAACCATGGGAAAAGCAAAGAGAAAAAACCTACTCCATGGAGACTAAAC

AACATGCTACTAAAAAACCAATGGGTCAATGAGGAAATCAAGAAGGAAATTAAAAACTAC

CTTGAAACAAATGATAATGAAGACACAACCTCTCAAAATCTATGGGATGCTGCGAAAGCA

GTGCTCAGAGGGAAATTTATAGCAATCCAGGCCTTTCTCAAAAAAGAAGAAAGATCCCAA

ATTGACAACTTAACCCTCCACCTAAATGAATTAGAAAAAGAAGAACAAAAAAGTCCTAAA

GTCAGCAGAAGGAAGGAAATTATAAAGATCAAAGAAGAAATCAATAAAATAGAGACTCAA

AAAACAATAGAGAAAATTAATAAAACCAAGAGCTGGTTCTTTGAAAAGGTGAACAAAATT

GATAAACCCCTGGCCAGACTCACTAAAAAGAGGAGAGAAAGAACCCAAATCACCAAAATT

ATAAATGAAAAAGGAGAAATCACAACGGATACAGCAGAAATACAAAAAACCATAAGAGAA

TACTATGAACAACTATATGGCAATAAGTTTGACAATCTGGAAGAAATGGACAATTTTCTA

GAATCTTACAGCCTGCCAAAACTGAATCAAGCAGAAACAGACCAACTGAACAGACCGATC

ACTAGAAATGAAATTGAAGAGGTCATAAAATCACTCCCTACAAATAAAAGCCCAGGACCA

GATGGCTTCACAGGTGAATTCTATCAAACATATAAAGAGGAATTGGTGCCCATCCTCCTT

AAACTCTTTCAAAAGGTTGAAGAAGAAGGAATACTCCCAAAGACATTCTATGAGGCCACC

ATCACCCTCATTCCAAAACCAGGCAGAGATACCACCAAAAAAGAAAACTATCGCCCAATA

TCATTGATGAATATAGATGCAAAAATTCTCAACAAAATCTTAGCCAACCGAATCCAACAA

CATATCAAAAAAATTATACACCATGACCAGGTTGGGTTCATCCCAGGTTCACAAGGATGG

TTCAACATACGCAAATCAATCAACATCATACACCACATTAACAAAAAAAAAGTCAAAAAT

CATATGATCATCTCAATAGATGCAGAAAAAGCATTTGACAAAGTTCAACATCCATTCATG

ATCAAGACCCTCGCCAAAGTGGGTATAGAGGGAACATTCCTGAATATAATCAAAGCCATT

TATGATAAACCCACAGCAAATATAATCCTCAATGGGGAAAAACTGAAAGCCTTCTCACTC

AAATCTGGAACAAGACAGGGATGCCCACTCTCACCACTGCTCTTCAACATCGTTTTGGAA

GTCTTAGCCACAGCAATTAGACAAACAAAAGAAATCAAAGGCATCCATATAGGAAGAGAA

GAGATCAAACTGTCACTGTATGCAGATGACATGATTCTATACCTAGAAAACCCTAAGGAC

TCAACCCCAAAACTCCTTGAACTGATTAATAAATTCAGCAAAGTGGCAGGATATAAGATT

AACATTCAGAAGTCAGTTGCATTTCTGTATACCAGCAATGAAACATTAGAAAAGGAATAC

AAAAATACGATACCTTTTAAAATTGTACCACACAAAATCAAATACCTCGGAATACACCTG

ACCAAAGAGGTAAAGGACCTATATGCCGAGAACTATAAAACCTTAATCAAAGAAATCAAA

GAAGATGTAAAGAAATGGAAAGATATTCCATGTTCCTGGATTGGAAAAATCAATATTGTG

AAAATGGCCATCCTACCCAAAGCAATCTACAGATTCAATGCAATCCCTATCAAATTACCC

ATGACATTTTTCACAGAACTAGAACAAACAATCCAAACATTTATATGGAACAACAAAAGA

CCCAGAATCGCCAAAGCAATCCTGAGAAACAAAAACCAAGCAGGAGGCATAACTCTCCCA

GACTTCAAGAAATACTACAAAGCCACAGTCATCAAAACAGTGTGGTACTGGTATCAAAAC

AGACAGACAGACCAATGGAACAGAATAGAGAATCCGGAAATTAACCCTGACACCTATGGT

CAATTAATCTTTGACAAGGGAGGCAAGAACATAAAATGGGAAAAGGAAAGTCTATTCAGC

AAGCATTGCTGGGAAACCTGGACAGCTGCATGCAAAGCAATGAAACTAGAACACACCCTC

ACACCATGCACAAAAATAAACTCCAAATGGCTGAAAGACTTAAATATACGACAGGACACC

ATCAAACTCCTAGAAGAAAACATAGGCAAAACACTCTCTGACATCAACATCATGAATATT

TTCTCAGGTCAGTCTCCCAAAGCAATAGAAATTAGAGCAAAAATAAACCCATGGGACCTC

ATCAAACTGAAAAGCTTTTGCACAGCAAAGGAAACCCAAAAGAAAACAAAAAGACAACTT

ACAGAATGGGAGAAAATAGTTTCAAATGATGCAACTGACAAGGGCTTAATCTCTAGAATA

TATAAGCAACTTATACAACTCAACAGCAAAAAAACCAATCAATCAATGGAAAAATGGGCA

AAAGACCTGAATAGACATTTCTCCAAGGAAGATATACAGATGGCCAACAAACACATGAAA

AAATGCTCAACATCGCTGATTATAAGAGAAATGCAAATCAAAACTACCATGAGATACCAC

CTCACACCAGTCAGAATGGCCATCATTAATAAATCCACAAATAACAAGTGCTGGAGGGGC

TGTGGAGAAAAGGGAACCCTCCTGCACTGTTGGTGGGAATGTAAACTGGTACAGCCACTA

TGGAGAACAGTTTGGAGATACCTTAGAAATCTATACATAGAACTTCCATATGACCCTGCA

ATCCCACTCTTGGGCATCTATCCGGACAAAGCTCTACTTAAAAGAGACACATGCACCCGC

ATGTTCATTGCAGCACTATTCACAATAGCCAGGACATGGAAACAATCCAAATGTCCATCG

ACAGAGGATTGGATTCGGAAGATGTGGTATATATACACGATGGAATACTACTCAGCCATA

AAAAAGGATGACATCATGCCATTTGCAGCAACATGGATGGAACTAGAGAATCTCATACTG

AGTGAAATGAGCCAGAAAGACAAAGACAAATACCATATGATATCACTTATAACTGGAATC

TAATATCCAGCACAAATGAACATCTCCTCAGAAAAGAAAATCATGGACTTGGAGAAGAGA

CTTGTGGTTGCCTGATGGGAGGGGGAGGGAGTGGGAGGGATCGGGAGCTTGGGCTTATCA

GTCACAACCTAGAATAGATTTACAAGGAGATCCCGCTGAATAGCATTGAGAACTATGTCT

AGATACTCATGTTGCAACAGAAGAAATGGTGGGGGAAAAACTGTAATTGTAATGTATACA

TGTAAGGATAACCTGACCCCCTTGCTGTACAGTGGGAAAATAAAATTAAAAAAAAAAAAA

AAAAAAAAAAAAAAAAAAAAAA

>L1D8#LINE/L1D

GGACAAGATGGCGGAGGAGTAGGAAGACACGCTCGCCCTCTCCCACAAACACAACAAAAA

AAGCACATCTACAGAATAAATGACTCGCACAGAACAGCAACCAATCGCTGGCAGAGGAAC

CTAAACTCCAATAACGGCAAGAAATTCGTGACATTATTGGGCAGAACAGGAGAAAAGAGG

AGAGTGAGAGAAGGCGAATCCGAGCGAGACGGGGGCTCCCGAAAGGGAACTGCGGAGGAG

AAAGGGATCCCGCACCCTGGAAAGTCACCTACCGGGCGAAAGATCAAACGAACCGGAGGA

ATCTCCAGATGCAGAGAAGAGTGTAGCAGTAAGTTGGAGTACGGAAAAGCCGATCAAGAA

CCCAACGGACCATCTGAACTACGGGCAGAGTCACCAAAAATTGAGACGCCTGGGTGGGGG

CTGGGCACCGAGACCTCGCCTCGAAGGTTAGTCCCCGAGAGGGGGCCGGGGGACGCCGGG

GCTGCTGGGTGGGGGCTGCACCGAGACCTCGCCTCCGAAGGTTATCCCTGAGAAAGGGCC

GGGGGACGCCTGGGGGGGGGCTGGGCACCGAGACCTCGCCCCGAAGGTTAGTCCCCGAGA

AAGGGCCGGGGGACGCCTGGGGGGGGCGCCTGGGTGGGGGCTGGGCACCGAGACCTCGGC

TCCAGAGATTAGTCCCCGGGCTAGGGGGGCGGGGCAGAGCGGAAACTGCTTGGGAGGTCT

AGAAACCAGTTGACGGGGCAGAGACTGCCTGGGAGACTAGAAAACAAAGCTGTCGCAGAG

GAAGGGAGCAATACTCCAGGAGTGGGGAAGGGGAAAGCCACATCAGAGGGAACCTGGGAG

AAGAGCCTGGTCTGCGCCCGTGCTGGGGAGGGGAGAGAAGAAGGGGTGGGTCCCCATAGA

ATACCCCCCACGCCACAGCAAGCTTACAGGCCCGCTAGCTAGCTGAAAACTGTGCTTCCC

AGTGCATCCCCTCCCCCCACCCCCGCCACGCCCTACGCTCTCACGGACCTGGGGCTGCCT

GCCATCCAGGAGGGCTGGCCTCAACAATTGCCTGAAGCCTACCACCGCAGGGGCTGTCCC

TGCACAGGCCTGCTTGCCCTTTGGAGGGGCTACACTTCCGCAGAGCAGCACCAAACAGCA

CCAGCCCCCGAGAAAAGGCCTGCAGCCTAGAAAAGCTAGAACAAGCTTAGCCAGGCTGTG

AATAGATCGGCCTAATTCTCGGACGGTTTTTCTGAGTCGGGTTGCCCCGGGGAGGAGCCT

CTTCGGTTTCCAACGGCCCTGCTACCCGCCCAAGCCCCCAGGGGATGCTCTAGTGGACCA

GCTGCATAGGACTGCCAGCTCCAGGCAGGACCCCCTGCAGCCCAGAAAAGCTGCAACAAG

CTCAGCCAGACTGTGAAAAGATCCGCCTACATTCTCAGGCTGTCCTTCTGAGTTGGGCTG

CCCTAGGGAAGAGCCTCTCAGGTTCTCAGTGCCCCAGATAGCTGCTCCAGCCCCCAGGGG

GTGATGCACCCCTAAAAAGCAGCTGCCCAACACCGCCAACCCCCTGCAAGAACCCCACAG

CCTAAAAACACCAGAGCAAGCTCTGCATGGCCAAGTGAAATCTGCTACCATCGCGGTGTG

GACCTCTCAGTCCTGTCTGCCCTCAGGAAGTCCTCCTTTGCTTCAAAGAAACACTGTTAG

CCCCATCAACACTCCAGAAAAGCCACACTGCCTCAAAAAAGATTGACCAACAACGCCAGC

CCTCAGGAAATATTCCACGGCAGTGACAAGGCAAACACTGCCCGATCACGGAGAGTACAA

CTCCCTCAGGAGAAAGAAAACAACAAGCAAGATGAAGAAGCTGAGAAACCACCCCCAGTC

AAACCAACAGGAGAACTCACCTAAAACAGTCAACAATGAAACAGATCTCGGCAGTCTGAC

AGACCTGGAGTTCAAAAGAGAAATAGTGAAAATACTGAAGGAATTAAGAGAAGATATGAA

CAGTAATGCAGATACCCTCAGAAAGGAACTAGAAAATATAAGGAGGAGCCAAGAAAAACT

AGAACATTCATTTGCAGAGATGCAAACTGAACTAGGGGCAGTAAAAACCAGAATGAATAA

TGCAGAAGAACGAATCAGTGATATGGAAGATAGAATAATGGAAATCACTCAATCTGGTCA

ACAGACAGAAAACCGAATCAAAAAACTGGAAAGCAATATAAGAGACCTATGGGATAATAT

AAAGCGGGCCAATCTACGCATAATAGGAATTCCAGAAGGAGTAGAAAAAGATAAGGGAAT

GGAAAATATATTTGAAGAAATTATTGCTGGAAACTTCCCAAATCTAAAGGATACTGGATT

CAAGATACAAGAAGCACAGAGGGCCCCAAACAAACTGAACCCAAACAGACCCACACCAAG

ACACATCATAATAAAAATGGCAAAAGTTAGTGATAAAGAGAGGATCCTAAAGGCAGCAAG

AGAAAAACAGAATGTTACCTACAAGGGAACCCCCATAAGAATATCAGCTGATTTCTCTAC

AGAAACACTACAGGCCAGGAGGGAATGGCAAGAGATATTTAAAGTGCTCAAAGGAAAAAA

TATGCAACCTAGAATACTCTATCCAGCAAGAATATCATTTAAAATAGAAGGGGAAATAAA

AATTTTTCCCAACAAACAAAAACTTAAAGAATACAGCAACACAAAACCCAGGTTAAAGGA

AATATTGAAAGGGCTTCTCTAAACCAAAAAGAAAGGAAGGAAAGGGAAGAAAAAAGAAAA

GAAAAAAAAAAAAAAAGAAGAAGAAGAGGAAGAACTAGGACTGAGGAAACTGCAATCAGA

GAGCAGTCACTCAAATAAGCCAGCATACAGATTTAATCATGAACATGCTTCAAACAAAAT

AAAATTAAAAAGAAAAAAATAAAAAAGAGTCATCAAAACCATAAAATGTGGGCAAGGGAT

GTCAGGAGGTAAATAACCCTTTTTGTTTATATGTATGTCTCTCTTCTTAATTTTAATATA

GTAATGAAGTGTTTGAACTTACAGGACCATCAGGCTAAAACACACATTTATGGGAAGGGG

TTAGCATACTTAAAAAACAGGGCAATCACAAGCCAAAACCAAATATTGCATTTGCAAAAA

ATGAAAAAAAAAACACTCAAGCAGATAATAACAGGAGACCATCCAACCAAAAAAAAAAAA

AAAAAATGGAGAACCATAGAATCAACTGGAACACGAGGTTCAAATGGCAATAAATAATCA

TCTATCAATTATCACCTTAAATGTCAATGGACTGAATGCCCCAATCAAAAGACACAGAGT

GGCTGAGTGGATAAAAAGGCAAAAACCTTCAATATGCTGCCTACAAGAAACTCACCTTAG

GACAAAAGATACATATAGATTGAAAGTGAAAGGGTGGGGAAAAATATTTCACGCCAATAG

ACATGACAGAAAAGCAGGAGTCGCAACGCTCATATCAGACAAAATAGACTTTAAAACAAA

AGGCATAAAGAAAGACAAAGAAGGACACTATTTAATGATTAAGGGATCCATCCAAGGAGA

GGATGTTACTATTGTCAACATATATGCCCCAAATACAGGAGCACCCAGATACACACAACA

AATATTAACAGACATAAAGGGAGATATTGATGAGAATACAATCATAGTAGGAGACCTAAA

TACCCCCCTCACATCAATGGACAGATCCTCTAGACAGAAAACCAATAAAGCAACAGAGAT

CCTAAAGGAAACAATAGAAAAGTTAGACTTAATTGATATCTTCAGGACACTACATCCAAA

AAAAGCAGAATACACATTCTTCTCAAATGCTCATGGAACATTCTCAAGAATCGACCACAT

ATTGGGACACAAAGCGAATCTCAATAAATTTAGGAGCGTAGAAATTATCTCAAGTATCTT

CTCTGACCACAATGCCATGAAATTAGAAATCAACCATGGGAAAAGCAAAGAGAAAAAACC

TACTCCATGGAGACTAAACAACATGCTACTAAAAAACCAATGGGTCAATGAGGAAATCAA

GAAGGAAATTAAAAACTACCTTGAAACAAATGATAATGAAGACACAACCTCTCAAAATCT

ATGGGATGCTGCGAAAGCAGTGCTCAGAGGGAAATTTATAGCAATCCAGGCCTTTCTCAA

AAAAGAAGAAAGATCCCAAATTGACAACTTAACCCTCCACCTAAACGAATTAGAAAAAGA

AGAACAAAAAAGTCCTAAAGTCAGCAGAAGGAAGGAAATTATAAAGATCAAAGAAGAAAT

CAATAAAATAGAGACTCAAAAAACAATAGAGAAAATTAATAAAACCAAGAGCTGGTTCTT

TGAAAAGGTGAACAAAATTGACAAACCCCTGGCCAGACTCACTAAAAAGAGGAGAGAAAG

AACCCAAATAACCAAAATTATAAATGAAAAAGGAGAAATCACAACGGATACAGCAGAAAT

ACAAAAAACCATAAGAGAATACTATGAACAACTGTATGGCAACAAGTTTGACAATCTGGA

AGAAATGGACAATTTTCTAGAATCTTACAGCTTGCCAAAACTGAATCAAGCAGAAACAGA

CCAACTGAACAGACCGATCACTAGAAATGAAATTGAAGAGGTCATAAAATCACTCCCTAC

AAATAAAAGTCCAGGACCAGATGGCTTCACAGGTGAATTTTATCAAACATATAAAGAGGA

ATTGGTGCCCATCCTCCTTAAACTCTTTCAAAAGGTTGAAGAAGAAGGAATACTCCCAAA

GACATTCTATGAGGCCACCATCACCCTCATTCCAAAACCAGACAGAGATACCACCAAAAA

AGAAAACTATCGGCCAATATCATTGATGAATATAGATGCAAAAATTCTCAACAAAATCTT

AGCCAACCGAATCCAACAACATATCAAAAAAATTATACACCATGACCAGGTTGGGTTCAT

CCCAGGTTCACAAGGATGGTTCAACATACGCAAATCAATCAGCATCATACACCACATTAA

CAAAAAAAAGGTCAAAAATCATATGATCATCTCAATAGACGCAGAAAAAGCATTTGACAA

AGTTCAACATCCATTCATGATCAAGACCCTCGCCAAAGTGGGTATAGAGGGAACATTCCT

GAATATAATCAAAGCCATTTATGATAAACCCACAGCAAATATAATCCTCAATGGGGAAAA

ACTGAAAGCCTTCTCACTCAAATCTGGAACAAGACAGGGATGCCCACTCTCACCACTGCT

CTTCAACATAGTTTTGGAAGTCTTAGCCACAGCAATTAGACAAACAAAAGAAATAAAAGG

CATCCATATAGGAAGAGAAGAGATCAAACTGTCACTGTATGCAGATGATATGATACTATA

CCTAGAAAACCCTAAGGACTCAACCCCAAAACTCCTTGAACTGATTAATAAATTCAGCAA

AGTGGCAGGATATAAGATTAACATTCAGAAGTCAGTTGCATTTCTGTATACCAGCAATGA

AACATTAGAAAAGGAATACAAAAAAATGATACCTTTTAAAATTGTACCTCACAAAATCAA

ATACCTCGGAATACACCTGACCAAAGAGGTAAAGGACCTATATGCCGAGAACTATAAAAC

TTTAATCAAAGAAATCAAAGAAGATGTAAAGAAATGGAAAGATATTCCATGTTCCTGGAT

TGGAAAAATCAATATTGTGAAAATGGCCATCCTACCCAAAGCAATCTACAGATTCAATGC

AATCCCTATCAAATTACCCATGACATTGTTCACAGAACTAGAACAAACAATCCAAACATT

TATATGGAACCACAAAAGACCCAGAATCGCCAAAGCAATCCTGAGAAACAAAAACCAAGC

AGGAGGCATAACTCTCCCAGACTTCAAGAAATACTACAAAGCCACAGTCATCAAAACAGT

GTGGTACTGGTATCAAAACAGACAGACAGACCAATGGAACAGAATAGAGAATCCGGAAAT

AAACCCTGACACCTATGGTCAATTAATCTTTGACAAGGGAGGCAAGAACATCAAATGGGA

AAAGGAAAGTCTATTCAGCAAGCATTGCTGGGAAACCTGGACAGCTGCATGCAAAGCAAT

GAAACTAGAACACACCCTCACACCATGCACAAAAATAAACTCCAAATGGCTGAAAGACTT

AAATATACGACAGGACACCATCAAACTCCTAGAAGAAAACATAGGCAAAACACTCTCTGA

CATCAACATCATGAATATTTTCTCAGGTCAGTCTCCCAAAGCAATAGAAATTAGAGCAAA

AATAAACCCATGGGACCTCATCAAACTGAAAAGCTTTTGCACAGCAAAGGAAACCAAAAA

GAAAACAAAAAGACAACTTACAGAATGGGAGAAAATAGTTTCAAATGATGCAACTGACAA

GGGCTTAATCTCTAGAATATACAAGCAACTTATACAACTCAACAGCAAAAAAGCCAATCA

ATCAATGGAAAAATGGGCAAAAGACCTGAATAGACATTTCTCCAAGGAAGATATACAGAT

GGCCAACAAACACATGAAAAAATGCTCAACATCGCTGATTATAAGAGAAATGCAAATCAA

AACTACCATGAGATACCACCTCACACCAGTCAGAATGGCCATCATTAATAAATCCACAAA

TAACAAGTGCTGGAGGGGCTGTGGAGAAAAGGGAACCCTCCTGCACTGCTGGTGGGAATG

TAAACTGGTACAGCCACTATGGAGAACAGTTTGGAGATACCTTAGAAATCTATACATAGA

ACTTCCATATGACCCTGCAATCCCACTCTTGGGCATCTATCCGGACAAAGCTCTACTTAA

AAGAGACACATGCACCCGCATGTTCATTGCAGCCCTATTCACAATAGCCAGGACATGGAA

ACAATCCAAATGTCCATCGACAGATGATTGGATTCGGAAGAGTGGTATATATACACAATG

GAATACTACTCAGCCATAAAAAAGGATGACATAATGCCATTTGCAGCAACATGGATGGAA

CTAGAGAATCTCATACTGAGTGAAATGAGCCAGAAAGACAAAGACAAATACCATATGATA

TCACTTATAACTGGAATCTAATATCCAGCACAAATGAACATCTCCTCAGAAAAGAAAATC

ATGGACTTGGAGAAGAGACTTGTGGTTGCCTGATGGGAGGGGGAGGGAGTGGGAGGGATC

GGGAGCTTGGGCTTATCAGACACAACCTAGAATAGATTTATAAGGAGATCCTGCTGAATA

GCATTGAGAACTATGTCTAGATACTCATGTCGCAACAGAAGAAAGGGTGGGGGAAAAAAC

TGTAACTGCAATGTATACATCTAAGGATGACCTGACCCCCTTGCTGTACAGTGGGAAAAA

AAAAAAAAAAAAAAAAAAAA

>L1D9#LINE/L1D

GAGAGGACAAGATGGCGGAGGAGTAGGGGGACACGCTCGCCCTCTCCCACAAACACAACA

AAAAAAGCACATCTACAGAAGAAATGACTCGCACAGAACAACAACCAATCGCTGGCAGAG

GAACCTAAACTCCAATAACGGCAAGAAGTTCGTGACATTATTGGGCAGAACGGGAGAAAA

GAGGAGAGTGAGAGAAGGTGAATCCGAGCGGGACGGGCGCTCCCGAAAGGGAACTGCGGA

GGAGAAAGGGATCCCGCACCCTGGAAAGTCTCCTACCGGGGAAAGATCAAACGAACCGGA

GGAATCCCCAGATGCAGAGAAGAGTGTAGCAGTAAGTCGGAGTACGGAAAAACGATCAAG

AACCCAACGGACCATCTGAACTACGGGCACAGTCACCAAAAATTGAGACGCCTGGGTGGG

GGCTGGGCACCGAATCCTCGGCTCCAGAGGTTAGTCCCCGGGAAAGGGCCGGGGGACGCC

TGGGTGGGGGCTGGGCACCGAGACCTCGCCTCTGAAGGTTGGTCCCCGAGAGGGGGCCGG

GGGACGCCTGGGTGGGGCTGGGCACCGAGACCTCGCCTCCGAAGGTTAGTCCCCAAGAAA

GGGCCGGGGGACGCCTGGGGGGGGGCTGGGCACCGAGACCTCGGCTCCAGAGATTAGTCC

CCGGGCTAGGGGGGCGGGGAAGAGCGGAAACTGCTTGGGAGGTCTCTAAACCATTTGACG

GGGCAGAGACTGCCTGGGAGACTAGAAAACAAAGCTGTCGCAGAGGAAGGGAGCAATACT

CTAGGGGCGGGGAAGTGGAAAGCCGCCTCAGAGGGAACCTGGGAGAAGAGCCTGGTCTGC

GCCCGTGCTGGGGAGGGGAGAGAAGAAGGGGTGGGTCCCCATAGAATACCCCCCACGCCA

CAGCAAGCTTACAGGCCCGCTAGCTAGCAGAAAGCTGTGCTTCCCAGTGCATTCCCTCCC

CCCACCCCCGCCACCCCCTACGCTCTCGCGAACCTGGGGCTGCCTGCCATCCAGGAGGGC

TGGCCTCAACAATTGCCTGAAGCCTACCACCGCAGGGGCTCTCCCTGCACAGGCCTGCTT

GCCCTTTGGAGGGGCTACACTTCCACAGAGCAGCACCAAACACCACCAGCCCCCTAGAAA

AGGCCTGCAGCCCAGAAAAGCTAGAACAAGCCTAGCCAGGCCGTGAATAGATCGACCTAA

TTCTCCGACGGTTTTTCTGAGACGGGCTCCCCCGGGGAGGAGCCTCTTGAGTCTCCAAGG

GCCTTGCTACCCGCCCAAGACCCCAGGGGGTGCTAAGACCTAGTGGACCAGCTGCATAGG

ACTGCCAGCTCCAGGCAGGACCCCCTGCAGCCCAGAAAAGCTGCAACAAGCCTGGCCGAA

TTGGGAAAAGATCTCAGACGGTCTTTCTGAGTCGGGCTGCCCTGGGGAGGAGCCTCTTGA

GTCTCCAAGGGCCCTGCTACCCGCCCAAGACCCCAAGGGGTGCTGAGACCTAGTGGACCA

GCTGCATAGGACTGCCAGCTCCAGGCAGGACCCCCTGCAGCCCAGAAAAGCTGCAACAAG

CCTGGCCGATTGGGAAAAGATCTCAGACGGTCTTTCTGAGTCGGGCTGCCTGGGGAGGAG

CCTCTTGAGTCTCCAAGGGCCCTGCTACCCGCCCAAGACCCCAAGGGGTGCTGAGACCTA

GTGGACCAGCTGCATAGGACTGCCAGCTCCAGGCAGGACCCCCTGCAGCCCAGAAAAGCT

GCAACAAGCCTGGCCGAATCGGGAAAAGATCTCAGACGGTCTTTCTGAGTCGGGCTGCCC

TGGGGAGGAACCTCTTGAGTCTCCAAGGGCCCTGCTACCCGCCCAAGACCCCAGGGGGTG

CTGAGAACCAAGTGAAATCTGCTACCATCGTGGGGTGGACCTCCCAGTCCTGTCTGCCCT

CAGGAAGTCCTCCTTTGCTTCAAAGAAACACTGTTAGTCCCATCAACACTCCAGAAAAGC

CACACTGCCTCAAAAAAGATTGACCAACAACGACAGCCCTCAGGAAATATTCCACGGCAG

TGACAAGGCAAACACTACCCGATAACGGAGAGTACAACTCCCTCAGGAGAAAGAAGACAA

CAAGCAAGATGAAGAAGCTGAGAAACCACCCCCAGTCAAACCAACAGGAGAACTCACCTA

AAACAGTCAACAATGAAACAGATCTCTGCAGTCTGACAGACCTGGAGTTCAAAAGAGAAA

TAGTGAAAATACTGAAGGAATTAAGAGAAGATATGAACAGCAATGCAGATACCCTCAGAA

AGGAGCTAGAAAATATAAGGAGGAGCCAAGAAAAACTAGAACATTCATTTGCAGAGATGC

AAACTGAACTAGGGGCAGTAAAAACCAGAATGAATAATGCAGAAGAACGAATCAGTGATA

TGGAAGATAGAATAATGGAAATCACTCAATCTGGTCAACAGACAGAAAACCGAATCAAAA

AACTGGAAAGCAATATAAGAGACCTATGGGATAATATAAAACGGGCCAATCTACGCATAA

TAGGAATTCCAGAAGGAGTAGAAAAAGATAAGGGAATGGAAAATATATTTGAAGAAATTA

TCGCTGGAAACTTCCCAAATCTAAAGGATACTGGATTCAAGATACAAGAAGCACAGAGGG

CCCCAAACAAACTGAACCCAAACAGACCCACACCAAGACACATCATAATAAAAATGGCAA

AAGTTAGTGATAAAGAGAGGATCCTAAAGGCAGCAAGAGAAAAACAGAATGTTACCTACA

AGGGAACCCCCATAAGAATATCAGCTGATTTCTCTACAGAAACACTACAGGCCAGGAGGG

AATGGCAAGAGATATTTAAAGTGCTCAAAGGAAAAAATATGCAACCTAGAATACTTTATC

CAGCAAGAATATCATTTAAAATAGAAGGGGAAATAAAAATTTTTCCCAACAAACAAAAAC

TTAAAGAATACAGCAACACAAAACCCAGGTTAAAGGAAATATTGAAAGGGCTTCTCTAAA

CCAAAAAGAAAGGAAGGAAAGGGAAGAAAAAAGAAAAGAAAAAAAAAAAAAGAAGAAGAA

GAAGAGGAAGAACTAGGACTGAGGAAGATGCAATCAGAGAGCAGTCACTCAAATAAGCCA

GCATACAGATTTAATCATGAACATGCTTCAAACAAAATAAAATTAAAAAGAAAAAAATAA

AAGAGTCATCAAAACCATAAAATGTGGGCAAGGGATGTTAGGAGGTAAATAATCCTTTTT

GTTTGTATGTATGTCTCTCTTCTTAATTTTAATATAATAATGAAGTGTTTGAACTTACAG

GACCATCAGGCTAAAACACACAATTATGGGAAGGGGTTAGCATACTTAAAAAACAGGGCA

ACCACAAGCCAAAACCAAATATTGCATTTGCAAAAAATGAAAAAAAAAATACACTCAAGC

AGATAATAACAGGAGACCATCCAACCAAAAAAAAAAAAAAAAAAAAAAAAAAGAAGAATG

GAGAACCATAGAATCAACTGGAACACGAGGATCAAATGGCAATAAATAATCATCTATCAA

TTATCACCTTAAATGTCAATGGACTGAATGCCCCAATCAAAAGACACAGAATGGCTGAGT

GGATAAAACGGCAAAAACCTTCAATATGCTGCCTACAAGAAACTCACCTTAGGACAAAAG

ATACATATAGATTGAAAGTGAAAGGCTGGGGAAAAGTATTTCATGCCAATAGACATGACA

GAAAAGCAGGAGTTGCAACGCTCATATCAGACAAAATAGACTTTAAAACAAAAGACATAA

AGAAAGACAAAGAAGGACACTATTTAATGATTAAGGGATCCATCCAAGGAGAGGATGTTA

CTATCATCAACATATATGCCCCAAACATAGGAGCACCCAGATACATACAACAAATATTAA

CAGACATAAAGGGAGATATTGATGAGAATACAATCATAGTAGGAGACCTAAATACCCCCC

TCACATCAATGGACAGATCCTCTAGACAGAAAACCAATAAAGCAACAGAGATCCTAAAGG

AAACAATAGAAAAGTTAGACTTAATTGATATCTTCAGGACACTACATCCAAAAAAATCAG

AATACACATTCTTCTCAAATGCTCATGGAACATTCTCAAGAATCGACCACATATTGGGAC

ATAAAGCGAATCTCAATAAATTTAGGAGCATAGAAATTATCTCAAGTATCTTCTCTGACC

ACAATGCCATGAAATTAGAAATCAACCATGGGAAAAGGAAAGAGAAAAAACCTACTCCAT

GGAGACTAAACAACATGCTACTAAAAAACCAATGGGTCAATGAGGAAATCAAGAAGGAAA

TTAAAAACTATCTTGAAACAAATGATAATGAAGACACAACCTCTCAAAATCTATGGGATG

CTGCGAAAGCAGTGCTCAGAGGGAAATTTATAGCAATCCAGGCCTTTCTCAAAAAAGAAG

AAAGATCCCAAATTGACAACTTAACCCTCCACCTAAATGAATTAGAAAAAGAAGAACAAA

GAAGTCCTAAAGTCAGCAGAAGGAAGGAAATTGTAAAGATCAAAGAAGAAATCAATAAAA

TAGAGACTCAAAAAACAATAGAGAAAATTAATAAAACCAAGAGCTGGTTCTTTGAAAAGG

TGAACAAAATTGATAAACCCCTGGCCAGACTCACTAAAAAGAGGAGAGAAAGAACCCAAA

TCACCAAAATTATAAATGAAAAAGGAGAAATCACAACGGATACAGCAGAAATACAAAAAA

CCATAAGAGAATACTATGAACAACTATATGGCAATAAGTTTGACAATCTGGAAGAAATGG

ACAATTTTCTAGAATCTTACAGCCTGCCAAAACTGAATCAAGCAGAAACAGACCAACTGA

ACAGACCGATCACTAGAAATGAAATTGAAGAGGTCATAAAATCACTCCCTACAAATAAAA

GCCCAGGACCAGATGGCTTCACAGGTGAATTCTATCAAACATATAAAGAGGAATTGGTGC

CCATCCTCCTTAAACTCTTTCAAAAGGTTGAAGAAGAAGGAATACTCCCAAAGACATTCT

ATGAGGCCACCATCACCCTCATTCCAAAACCAGGCAGAGATACCACCAAAAAAGAAAACT

ATCGCCCAATATCATTGATGAATATAGATGCAAAAATTCTCAACAAAATCTTAGCCAACC

GAATCCAACAACATATCAAAAAAATTATACACCATGACCAGGTTGGGTTCATCCCAGGTT

CACAAGGATGGTTCAACATACGCAAATCAATCAACATCATACACCACATTAACAAAAAAA

AAGTCAAAAATCATATGATCATCTCAATAGATGCAGAAAAAGCATTTGACAAAGTTCAAC

ATCCATTCATGATCAAGACCCTCGCCAAAGTGGGTATAGAGGGAACATTCCTGAATATAA

TCAAAGCCATTTATGATAAACCCACAGCAAATATAATCCTCAATGGGGAAAAACTGAAAG

CCTTCTCACTCAAATCTGGAACAAGACAGGGATGCCCACTCTCACCACTGCTCTTCAACA

TCGTTTTGGAAGTCTTAGCCACAGCAATTAGACAAACAAAAGAAATCAAAGGCATCCATA

TAGGAAGAGAAGAGATCAAACTGTCACTGTATGCAGATGACATGATTCTATACCTAGAAA

ACCCTAAGGACTCAACCCCAAAACTCCTTGAACTGATTAATAAATTCAGCAAAGTGGCAG

GATATAAGATTAACATTCAGAAGTCAGTTGCATTTCTGTATACCAGCAATGAAACATTAG

AAAAGGAATACAAAAATACGATACCTTTTAAAATTGTACCTCACAAAATCAAATACCTCG

GAATACACCTAACCAAAGAGGTAAAGGACCTATATGCCGAGAACTATAAAACCTTAATCA

AAGAAATCAAAGAAGATGTAAAAAAATGGAAAGATATTCCATGTTCCTGGATTGGAAAAA

TCAATATTGTGAAAATGGCCATCCTACCCAAAGCAATCTACAGATTCAATGCAATCCCTA

TCAAATTACCCATGACATTTTTCACAGAACTAGAACAAACAATCCAAACATTTATATGGA

ACCACAAAAGACCCAGAATCGCCAAAGCAATCCTGAGAAACAAAAACCAAGCAGGAGGCA

TAACTCTCCCAGACTTCAAGAAATACTACAAAGCCACAGTCATCAAAACAGTGTGGTACT

GGTATCAAAACAGACAGACAGACCAATGGAACAGAATAGAGAATCCGGAAATTAACCCTG

ACACCTATGGTCAATTAATCTTTGACAAGGGAGGCAAGAACATCAAATGGGAAAAGGAAA

GTCTATTCAGCAAGCATTGCTGGGAAACCTGGACAGCTGCATGCAAAGCAATGAAACTAG

AACACACCCTCACACCATGCACAAAAATAAACTCCAAATGGCTGAAAGACTTAAATATAC

GACAGGACACCATCAAACTCCTAGAAGAAAACATAGGCAAAACACTCTCTGACATCAACA

TCATGAATATTTTCTCAGGTCAGTCTCCCAAAGCAATAGAAATTAGAGCAAAAATAAACC

CATGGGACCTCATCAAACTGAAAAGCTTTTGCACAGCAAAGGAAACCCAAAAGAAAACAA

AAAGACAACTTACAGAATGGGAGAAAATAGTTTCAAATGATGCAACTGACAAGGGCTTAA

TCTCTAGAATATATAAGCAACTTATACAACTCAACAGCAAAAAAACCAATCAATCAATGG

AAAAATGGGCAAAAGACCTGAATAGACATTTCTCCAAGGAAGATATACAGATGGCCAACA

AACACATGAAAAAATGCTCAACATCGCTGATTATAAGAGAAATGCAAATCAAAACTACCA

TGAGATACCACCTCACACCAGTCAGAATGGCCATCATTAATAAATCCACAAATAACAAGT

GCTGGAGGGGCTGTGGAGAAAAGGGAACCCTCCTGCACTGTTGGTGGGAATGTAAACTGG

TACAGCCACTATGGAGAACAGTTTGGAGATACCTTAGAAATCTATACATAGAACTTCCAT

ATGACCCTGCAATCCCACTCTTGGGCATCTATCCGGACAAAGCTCTACTTAAAAGAGACA

CATGCACCCGCATGTTCATTGCAGCACTATTCACAATAGCCAGGACATGGAAACAATCCA

AATGTCCATCGACAGAGGATTGGATTCGGAAGATGTGGTATATATACACGATGGAATACT

ACTCAGCCATAAAAAAGGATGACATCATGCCATTTGCAGCAACATGGATGGAACTAGAGA

ATCTCATACTGAGTGAAATGAGCCAGAAAGACAAAGACAAATACCATATGACATCACTTA

TAACTGGAATCTAATATCCAGCACAAATGAACATCTCCTCAGAAAAGAAAATCATGGACT

TGGAGAAGAGACTTGTGGTTGCCTGATGGGAGGGGGAGGGAGTGGGAGGGATCGGGAGCT

TGGGCTTATCAGTCACAACGTAGAATAGATTTACAAGGAGATCCCGCTGAATAGCATTGA

GAACTATGTCTAGATACTCATGTTGCAACAGAAGAAATGGTGGGGGAAAAACTGTAATTG

TAATGTATACATGTAAGGATAACCTGACCCCCTTGCTGTACAGTGGGAAAATAAAATTAA

AAAAAAAAAAAAAAAAAAAAAAAAA

>L1D10#LINE/L1D

GAGGACAAGATGGCGGAGGAGTAGGGGGACACGCTCGCCCTCTCCCACAAACACAACAAA

AAAAGCACATCTACAGAAGAAATGACTCGCACAGAACAGCAACCAATCGCTGGCAGAGGA

ACCTAAACTCCAATAACGGCAAGAAGTTCGTGACATTATTGGGCAGAACGGGAGAAAAGA

GGAGAGTGAGAGAAGGTGAATCCAGCGGGACGGGCGCTCCCGAAAGGGAACTGCGGAGGA

GAAAGGGATCCCGCACCCTGGAAAGTCACCTACCGGGCGAAAGATCAAATGAACCGGAGG

AATCCCCAGATGCAGAGAAGAGTGTAGCAGTAAGTCGGAGTACGGAAAAACCGATCAAGA

ACCCAACGGACCATCTGAACTACGGGCACAGTCACCAAAAATTGAGACGCCTGGGTGGGG

GCTGGGCACCGACCTCGCTCCAGGTTAGTCCCCGGAAAGGGCCGGGGGACGCCTGGGTGG

GGGCTGGGCACCGAGACCTCGGCTCCAGAGATTAGTCCCCGGGCTAGGGGGGCGGGGAAG

AGCGGAAACTGCTTGGGAGGTCTCTAAACCATTTGACGGGGCAGAGACTGCCTGGGAGAC

TAGAAAACAAAGCTGTCGCAGAGGAAGGGAGCAATACTCTAGGGGCGGGGAAGTGGAAAG

CCGCCTCAGAGGGAACCTGGGAGAAGAGCCTGGTCTGCGCCCGTGCTGGGGAGGGGAGAG

AAGAAGGGGTGGGTTCCCATAGAATACCCCCCACGCCACAGCAAGCTTACAGGCCCGCTA

GCTAGCAGAAAGCTGTGCTTCCCAGTGCATTCCCTCCCCCCACCCCCGCCACCCCCTACG

CTCTCGCGGAACCTGGGGCTGCCTGCCATCCAGGAGGGCTGGCCTCAACAATTGCCTGAA

GCCTACCACCGCAGGGGCTCTCCCTGCACAGGCCTGCTTGCCCTTTGGAGGGGCTACACT

TCCACAGAGCAGCACCAAACACCACCAGCCCCCTAGAAAAGGCCTGCAGCCCAGAAAAGC

TAGAACAAGCCTAGCCAGGCCGTGAATAGATCGGCCTAATTCTCCGACGGTTTTTCTGAG

ACGGGCTCCCCCGGGGAGGAGCCTCTTGGGTCTCCAAGGGCCTTGCTACCCGCCCAAGAC

CCCAGGGGGTGCTAAGACCTAGTGGACCAGCTGCATAGGACTGCCAGCTCCAGGCAGGAC

CCCCTGCAGCCCAGAAAAGCTGCAACAAGCCTGGCCGATGGGAAAAGATCTCAGACGGTC

TTTCTGATCGGGCTGCCCTGGGGAGGAGCCTCTTGAGTCTCCAAGGGCCCTGCTACCCGC

CCAAGACCCCAGGGGGTGCTGAGACCTAGTGGACCAGCTGCATAGGACTGCCAGCTCCAG

GCAGGACCCCCTGCAGCCCAGAAAAGCTGCAACAAGCCTGGCCGAATCGGGAAAAGATCT

CAGACGGTCTTTCTGAATCGGGCTGCCCTGGGGAGGAGCCTCTTGAGTCTCCAAGGGCCC

TGCTACCCGCCCAAGACCCCAGGGGGTGCTGAGAACCAAGTGAAATCTGCTACCATCGTG

GGGTGGACCTCCCAGTCCTGTCTGCCCTCAGGAAGTCCTCCTTTGCTTCAAAGAAACACT

GTTAGTCCCATCAACACTCCAGAAAAGCCACACTGCCTCAAAAAAGATTGACCAACAACG

CCAGCCCTCAGGAAATATTCCACGGCAGTGACAAGGCAAACACTACCCGATAACGGAGAG

TACAACTCCCTCAGGAGAAAGAAGACAACAAGCAAGATGAAGAAGCTGAGAAACCACCCC

CAGTCAAACCAACAGGAGAACTCACCTAAAACAGTCAACAATGAAACAGATCTCTGCAGT

CAGACAGACCTGGAATTCAAAAGAGAAATACTGAAAATACTGAAGGAATTAAGAGAAGAT

ATGAACAGTAATGCAGATACCCTCAGAAAGGAGCTAGAAAATATAAGGAGGAGCCAAGAA

AAACTAGAACATTCATTTGCAGAGATGCAAACTGAACTAGGGGCAGTAAAAACCAGAATG

AATAATGCAGAAGAACGAATCAGTGATATGGAAGATAGAATAATGGAAATCACTCAATCT

GGTCAACAGACAGAAAACCGAATCAAAAAACTGGAAAGCAATATAAGAGACCTATGGGAT

AATATAAAACGGGCCAATCTACGCATAATAGGAATTCCAGAAGGAGTAGAAAAAGATAAG

GGAATGGAAAATATATTTGAAGAAATTATCGCTGGAAACTTCCCAAATCTAAAGGATACT

GGATTCAAGATACAAGAAGCACAGAGGGCCCCAAACAAACTGAACCCAAACAGACCCACA

CCAAGACACATCATAATAAAAATGGCAAAAGTTAGTGATAAAGAGAGGATCCTAAAGGCA

GCAAGAGAAAAACAGAATGTTACCTACAAGGGAACCCCCATAAGAATATCAGCTGATTTC

TCTACAGAAACACTACAGGCCAGGAGGGAATGGCAAGAGATATTTAAAGTGCTCAAAGGA

AAAAATATGCAACCTAGAATACTTTATCCAGCAAGAATATCATTTAAAATAGAAGGGGAA

ATAAAAATTTTTCCCAACAAACAAAAACTTAAAGAATACAGCAACACAAAACCCAGGTTA

AAGGAAATATTGAAAGGGCTTCTCTAAACCAAAAAGAAAGGAAGGAAAGGGAAGAAAAAA

GAAAAGAAAAAAAAAAAAAAGAAGAAGAAGAGGAAGAACTAGGACTGAGGAAACCGCAAT

CAGAGAGCAGTCACTCAAATAAGCCAGCATACAGATTTAATCATGAACATGCTTCAAACA

AAATAAAATTAAAAAGAAAAAAATAAAAGAGTCATCAAAACCATAAAATGTGGGCAAGGG

ATGTTAGGAGGTAAATAATCCTTTTTGTTTGTATGTATGTCTCTCTTCTTAATTTTAATA

TAATAATGAAGTGTTTGAACTTACAGGACCATCAGGCTAAAACACACAATTATGGGAAGG

GGTTAGCATACTTAAAAAACAGGGCAACCACAAGCCAAAACCAAATATTGCATTTGCAAA

AAATGAAAAAAAAAAATACACTCAAGCAGATAATAACAGGAGACCATCCAACCAAAAAAA

AAAAAAAAAAAAAAAAAGAAGAATGGAGAACCATAGAATCAACTGGAACACGAGGATCAA

ATGGCAATAAATAATCATCTATCAATTATCACCTTAAATGTCAATGGACTGAATGCCCCA

ATCAAAAGACACAGAATGGCTGAGTGGATAAAAAGGCAAAAACCTTCAATATGCTGCCTA

CAAGAAACTCACCTTAGGACAAAAGATACATATAGATTGAAAGTGAAAGGCTGGGGAAAA

GTATTTCATGCCAATAGACATGACAGAAAAGCAGGAGTCGCAACGCTCATATCAGACAAA

ATAGACTTTAAAACAAAAGACATAAAGAAAGACAAAGAAGGACACTATTTAATGATTAAG

GGATCCATCCAAGGAGAGGATGTTACTATCATCAACATATATGCCCCAAACATAGGAGCA

CCCAGATACATACAACAAATATTAACAGACATAAAGGGAGATATTGATGAGAATACAATC

ATAGTAGGAGACCTAAATACCCCCCTCACATCAATGGACAGATCCTCTAGACAGAAAACC

AATAAAGCAACAGAGATCCTAAAGGAAACAATAGAAAAGTTAGACTTAATTGATATCTTC

AGGACACTACATCCAAAAAAAGCAGAATACACATTCTTCTCAAATGCTCATGGAACATTC

TCAAGAATCGACCACATATTGGGACATAAAGCGAATCTCAATAAATTTAGGAGCATAGAA

ATTATCTCAAGTATCTTCTCTGACCACAATGCCATGAAATTAGAAATCAACCATGGGAAA

AGCAAAGAGAAAAAACCTACTCCATGGAGACTAAACAACATGCTACTAAAAAACCAATGG

GTCAATGAGGAAATCAAGAAGGAAATTAAAAACTACCTTGAAACAAATGATAATGAAGAC

ACAACCTCTCAAAATCTATGGGATGCTGCGAAAGCAGTGCTCAGAGGGAAATTTATAGCA

ATACAGGCCTTTCTCAAAAAAGAAGAAAGATCCCAAATTGACAACTTAACCCTCCACCTA

AATGAATTAGAAAAAGAAGAACAAAAAAGTCCTAAAGTCAGCAGAAGGAAGGAAATTATA

AAGATCAAAGAAGAAATCAATAAAATAGAGACTCAAAAAACAATAGAGAAAATTAATAAA

ACCAAGAGCTGGTTCTTTGAAAAGGTGAACAAAATTGATAAACCCCTGGCCAGACTCACT

AAAAAGAGGAGAGAAAGAACCCAAATCACCAAAATTATAAATGAAAAAGGAGAAATCACA

ACGGATACAGCAGAAATACAAAAAACCATAAGAGAATACTATGAACAACTATATGGCAAC

AAGTTTGACAATCTGGAAGAAATGGACAATTTTCTAGAATCTTACAGCCTGCCAAAACTG

AATCAAGCAGAAACAGACCAACTGAACAGACCGATCACTAGAAATGAAATTGAAGAGGTC

ATAAAATCACTCCCTACAAATAAAAGTCCAGGACCAGATGGCTTCACAGGTGAATTTTAT

CAAACATATAAAGAGGAATTGGTGCCCATCCTCCTTAAACTCTTTCAAAAGGTTGAAGAA

GAAGGAATACTCCCAAAGACATTCTATGAGGCCACCATCACCCTCATTCCAAAACCAGGC

AGAGATACCACCAAAAAAGAAAACTATCGCCCAATATCATTGATGAATATAGATGCAAAA

ATTCTCAACAAAATCTTAGCCAACCGAATCCAACAACATATCAAAAAAATTATACACCAT

GACCAGGTTGGGTTCATCCCAGGTTCACAAGGATGGTTCAACATACGCAAATCAATCAGC

ATCATACACCACATTAACAAAAAAAAAGTCAAAAATCATATGATCATCTCAATAGACGCA

GAAAAAGCATTTGACAAAGTTCAACATCCATTCATGATCAAGACCCTCGCCAAAGTGGGT

ATAGAGGGAACATTCCTGAATATAATCAAAGCCATTTATGATAAACCCACAGCAAATATA

ATCCTCAATGGGGAAAAACTGAAAGCCTTCTCACTCAAATCTGGAACAAGACAGGGATGC

CCACTCTCACCACTGCTCTTCAACATCGTTTTGGAAGTCTTAGCCACAGCAATTAGACAA

ACAAAAGAAATCAAAGGCATCCATATAGGAAGAGAAGAGATCAAACTGTCACTGTATGCA

GATGACATGATTCTATACCTAGAAAACCCTAAGGACTCAACCCCAAAACTCCTTGAACTG

ATTAATAAATTCAGCAAAGTGGCAGGATATAAGATTAACATTCAGAAGTCAGTTGCATTT

CTGTATACCAGCAATGAAACATTAGAAAAGGAATACAAAAATACGATACCTTTTAAAATT

GTACCACACAAAATCAAATACCTCGGAATACACCTGACCAAAGAGGTAAAGGACCTATAT

GCCGAGAACTATAAAACCTTAATCAAAGAAATCAAAGAAGATGTAAAGAAATGGAAAGAT

ATTCCATGTTCCTGGATTGGAAAAATCAATATTGTGAAAATGGCCATCCTACCCAAAGCA

ATCTACAGATTCAATGCAATCCCTATCAAATTACCCATGACATTTTTCACAGAACTAGAA

CAAACAATCCAAACATTTATATGGAACAACAAAAGACCCAGAATCGCCAAAGCAATCCTG

AGAAACAAAAACCAAGCAGGAGGCATAACTCTCCCAGACTTCAAGAAATACTACAAAGCC

ACAGTCATCAAAACAGTGTGGTACTGGTATCAAAACAGACAGACAGACCAATGGAACAGA

ATAGAGAATCCGGAAATTAACCCTGACACCTATGGTCAATTAATCTTTGACAAGGGAGGC

AAGAACATAAAATGGGAAAAGGAAAGTCTATTCAGCAAGCATTGCTGGGAAACCTGGACA

GCTGCATGCAAAGCAATGAAACTAGAACACACCCTCACACCATGCACAAAAATAAACTCC

AAATGGCTGAAAGACTTAAATATACGACAGGACACCATCAAACTCCTAGAAGAAAACATA

GGCAAAACACTCTCTGACATCAACATCATGAATATTTTCTCAGGTCAGTCTCCCAAAGCA

ATAGAAATTAGAGCAAAAATAAACCCATGGGACCTCATCAAACTGAAAAGCTTTTGCACA

GCAAAGGAAACCAAAAGAAAACAAAAAGACAACTTACAGAATGGGAGAAAATAGTTTCAA

ATGATGCAACTGACAAGGGCTTAATCTCTAGAATATATAAGCAACTTATACAACTCAACA

GCAAAAAAACCAATCAATCAATGGAAAAATGGGCAAAAGACCTGAATAGACATTTCTCCA

AAGAAGATATACAGATGGCCAACAAACACATGAAAAAATGCTCAACATCGCTGATTATAA

GAGAAATGCAAATCAAAACTACCATGAGATACCACCTCACACCAGTCAGAATGGCCATCA

TTAATAAATCCACAAATAACAAGTGCTGGAGGGGCTGTGGAGAAAAGGGAACCCTCCTGC

ACTGCTGGTGGGAATGTAAACTGGTACAGCCACTATGGAGAACAGTTTGGAGATACCTTA

GAAATCTATACATAGAACTTCCATATGACCCTGCAATCCCACTCTTGGGCATCTATCCGG

ACAAAGCTCTACTTAAAAGAGACACATGCACCCGCATGTTCATTGCAGCACTATTCACAA

TAGCCAGGACATGGAAACAATCCAAATGTCCATCGACAGATGATTGGATTCGGAAGAGTG

GTATATATACACAATGGAATACTACTCAGCCATAAAAAAGGATGACATCATGCCATTTGC

AGCAACATGGATGGAACTAGAGAATCTCATCCTGAGTGAAATGAGCCAGAAAGACAAAGA

CAAATACCATATGATATCACTTATAACTGGAATCTAATATCCAGCACAAATGAACATCTC

CTCAGAAGAGAAAATCATGGACTTGGAGAAGAGACTTGTGGTTGCCTGATGGGAGGGGGA

GGGAGTGGGAGGGATCGGGAGCTTGGGCTTATCAGACACAACCTAGAATAGATTTACAAG

GAGATCCCGCTGAATAGCATTGAGAACTATGTCTAGATACTCATGTTGCAACAGAAGAAA

TGGTGGGGGAAAAACTGTAATTGTAATGTATACATGTAAGGATAACCTGACCCCCTTGCT

GTACAGTGGGAAAATAAAATAAAAAAAAAAAAAAAAAAAAAAA

>L1D11#LINE/L1D

GAGAGAGGACAAGATGGCGGAGGAGTAGGAAGACACGCTCGCCCTCTCCCACAAACACAA

CAAAAAAAGCACATCTACAGAATAAATGACTCGCACAGAACAGCAACCAATCGCTGGCAG

AGGAACCTAAACTCCAATAACGGCAAGAAATTCGTGACATTATTGGGCAGAACAGGAGAA

AAGAGGAGAGTGAGAGAAGGCGAATCCGAGCGAGACGGGGGCTCCCGAAAGGGAACTGCG

GAGGAGAAAGGGATCCCGCACCCTGGAAAGTCACCTACCGGGCGAAAGATCAAACGAACC

GGAGGAATCTCCAGATGCAGAGAAGAGTGTAGCAGTAAGTTGGAGTACGGAAAAGCCGAT

CAAGAACCCAACGGACCATCTGAACTACGGGCAGAGTCACCAAAAATTGAGACGCCTGGG

TGGGGGCTGGGCACCGAGACCTCGCCTCTGAAGGTTAGTCCCCGAGAGGGGGCCGGGGGA

CGCCTGGGTGGGGGCTGGGCACCGAGACCTCGCCTCCGAAGGTTAGTCCCCGAGAGGGGG

CCGGGGGACGCCTGGGTGGGGGCTGGGCACCGAGACCTCGCCGCCGAAGGTTAATCCCCG

AGAAAGGGCCGGGGGACGCCGGGGGGGGCTGGGCACCGAGACCTCGCCGCCGAAGGTTAG

TCCCCGAGAGGGGGCCGGGGGACGCCTGGGGGGGGCTGCACCGAGACCTCGCCCCGAAGG

TTATCCCGAGAGGGCCGGGGGACGCCTGGGTGGGGGCTGGGCACCGAGACCTCGCCTCCG

AAGGTTAATCCCTGAGAAAGGGCCGGGGGACGCCTGGGAGGGGGGCTGGGCACCGAGACC

TCGCCGCGAAGGTTAGTCCCCGAGAAAGGGCCGGGGGGCGCCGCCTGGGTGGGGGCTGGG

CACCGAGACCTCGGCTCCAGAGATTAGTCCCCGGGCTAGGGGGGCGGGGCAGAGCGGAAA

CTGCTTGGGAGGTCTAGAAACCAGTTGACGGGGCAGAGACTGCCTGGGAGACTAGAAAAC

AAAGCTGTCGCAGAGGAAGGGAGCAATACTCCAGGAGTGGGGAAGGGGAAAGCCACATCA

GAGGGAACCTGGGAGAAGAGCCTGGTCTGCGCCCGTGCTGGGGAGGGGAGAGAAGAAGGG

GTGGGTCCCCATAGAATACCCCCCACGCCACAGCAAGCTTACAGGCCCGCTAGCTAGCTG

AAAACTCTGCTTCCCAGTGCATCCCCTCCCCCCACCCCCGCCACGCCCTACGCTCTCACG

GACCTGGGGCTGCCTGCCATCCAGGAGGGCTGGCCTCAACAATTGCCTGAAGCCTACCAC

CGCAGGGGCTGTCCCTGCACAGGCCTGCTTGCCCTTTGGAGGGGCTACACTTCCGCAGAG

CAGCACCAAACAGCACCAGCCCCCGAGAAAAGGCCTGCAGCCTAGAAAAGCTAGAACAAG

CTTAGCCAGGCTGTGAATAGATCGGCCTAATTCTCGGACGGTTTTTCTGAGTCGGGTTGC

CCCGGGGAGGAGCCTCTTCGGTTTCCAACGGCCCTGCTACCCGCCCAAGCCCCCAGGGGA

TGCTCTAGTGGACCAGCTGCATAGGACTGCCAGCTCCAGGCAGGACCCCCTGCAGCCCAG

AAAAGCTGCAACAAGCTCAGCCAGACTGTGAAAAGATCCGCCTACATTCTCAGGCTGTCC

TTCTGAGTTGGGCTGCCCTAGGGAAGAGCCTCTCAGGTTCTCAGTGCCCCAGATAGCTGC

TCCAGCCCCCAGGGGGTGATGCACCCCTAAAAAGCAGCTGCCCAACACCGCCAACCCCCT

GCAAGAACCCCACAGCCTAAAAACACCAGAGCAAGCTCTGCATGGCCAAGTGAAATCTGC

TACCATCGCGGTGTGGACCTCTCAGTCCTGTCTGCCCTCAGGAAGTCCTCCTTTGCTTCA

AAGAAACACTGTTAGCCCCATCAACACTCCAGAAAAGCCACACTGCCTCAAAAAAGATTG

ACCAACAACGCCAGCCCTCAGGAAATATTCCACGGCAGTGACAAGGCAAACACTGCCCGA

TCACGGAGAGTACAACTCCCTCAGGAGAAAGAAAACAACAAGCAAGATGAAGAAGCTGAG

AAACCACTCCCAGTCAAACCAACAGGAGAACTCACCTAAAACAGTCAACAATGAAACAGA

TCTCGGCAGTCTGACAGACCTGGAGTTCAAAAGAGAAATAGTGAAAATACTGAAGGAATT

AAGAGAAGATATGAACAGTAATGCAGATACCCTCAGAAAGGAACTAGAAAATATAAGGAG

GAGCCAAGAAAAACTAGAACATTCATTTGCAGAGATGCAAACTGAACTAGGGGCAGTAAA

AACCAGAATGAATAATGCAGAAGAACGAATCAGTGATATGGAAGATAGAATAATGGAAAT

CACTCAATCTGGTCAACAGACAGAAAACCGAATCAAAAAACTGGAAAGCAATATAAGAGA

CCTATGGGATAATATAAAGCGGGCCAATCTACGCATAATAGGAATTCCAGAAGGAGTAGA

AAAAGATAAGGGAATGGAAAATATATTTGAAGAAATTATTGCTGGAAACTTCCCAAATCT

AAAGGATACTGGATTCAAGATACAAGAAGCACAGAGGGCCCCAAACAAACTGAACCCAAA

CAGACCCACACCAAGACACATCATAATAAAAATGGCAAAAGTTAGTGATAAAGAGAGGAT

CCTAAAGGCAGCAAGAGAAAAACAGAATGTTACCTACAAGGGAACCCCCATAAGAATATC

AGCTGATTTCTCTACAGAAACACTACAGGCCAGGAGGGAATGGCAAGAGATATTTAAAGT

GCTCAAAGGAAAAAATATGCAACCTAGAATACTCTATCCAGCAAGAATATCATTTAAAAT

AGAAGGGGAAATAAAAATTTTTCCCAACAAACAAAAACTTAAAGAATACAGCAACACAAA

ACCCAGGTTAAAGGAAATATTGAAAGGGCTTCTCTAAACCAAAAAGAAAGGAAGGAAAGG

GAAGAAAAAAGAAAAGAAAAAAAAAAAAAAGAAGAAGAAGAAGAGGAAGAACTAGGACTG

AGGAAACTGCAATCAGAGAGAAGTCACTCAAATAAGCCAGCATACAGATTTAATCATGAA

CATGCTTCAAACAAAATAAAATTAAAAAGAAAAAAATAAAAAAGAGTCATCAAAACCATA

AAATGTGGGCAAGGGATGTCAGGAGGTAAATAACCCTTTTTGTTTATATGTATGTCTCTC

TTCTTAATTTTAATATAGTAATGAAGTGTTTGAACTTACAGGACCATCAGGCTAAAACAC

ACATTTATGGGAAGGGGTTAGCATACTTAAAAAACAGGGCAATCACAAGCCAAAACCAAA

TATTGCATTTGCAAAAAATGAAAAAAAAAAACCACTCAAGCAGATAATAACAGGAGACCA

TCCAACCAAAAAAAAAAAAAAAAATGGAGAACCATAGAATCAACTGGAACACGAGGTTCA

AATGGCAATAAATAATCATCTATCAATTATCACCTTAAATGTCAATGGACTGAATGCCCC

AATCAAAAGACACAGAGTGGCTGAGTGGATAAAAAGGCAAAAACCTTCAATATGCTGCCT

ACAAGAAACTCACCTTAGGACAAAAGATACATATAGATTGAAAGTGAAAGGGTGGGGAAA

AATATTTCACGCCAATAGACATGACAGAAAAGCAGGAGTCGCAACGCTCATATCAGACAA

AATAGACTTTAAAACAAAAGGCATAAAGAAAGACAAAGAAGGACACTATTTAATGATTAA

GGGATCCATCCAAGGAGAGGATGTTACTATTGTCAACATATATGCCCCAAATACAGGAGC

ACCCAGATACACACAACAAATATTAACAGACATAAAGGGAGATATTGATGAGAATACAAT

CATAGTAGGAGACCTAAATACCCCCCTCACATCAATGGACAGATCCTCTAGACAGAAAAC

CAATAAAGCAACAGAGATCCTAAAGGAAACAATAGAAAAGTTAGACTTAATTGATATCTT

CAGGACACTACATCCAAAAAAAGCAGAATACACATTCTTCTCAAATGCTCATGGAACATT

CTCAAGAATCGACCACATATTGGGACACAAAGCGAATCTCAATAAATTTAGGAGCGTAGA

AATTATCTCAAGTATCTTCTCTGACCACAATGCCATGAAATTAGAAATCAACCATGGGAA

AAGCAAAGAGAAAAAACCTACTCCATGGAGACTAAACAACATGCTACTAAAAAACCAATG

GGTCAATGAGGAAATCAAGAAGGAAATTAAAAACTACCTTGAAACAAATGATAATGAAGA

CACAACCTCTCAAAATCTATGGGATGCTGCGAAAGCAGTGCTCAGAGGGAAATTTATAGC

AATCCAGGCCTTTCTCAAAAAAGAAGAAAGATCCCAAATTGACAACTTAACCCTCCACCT

AAACGAATTAGAAAAAGAAGAACAAAAAAGTCCTAAAGTCAGCAGAAGGAAGGAAATTAT

AAAGATCAAAGAAGAAATCAATAAAATAGAGACTCAAAAAACAATAGAGAAAATTAATAA

AACCAAGAGCTGGTTCTTTGAAAAGGTGAACAAAATTGACAAACCCCTGGCCAGACTCAC

TAAAAAGAGGAGAGAAAGAACCCAAATACCAAAATTATAAATGAAAAAGGAGAAATCACA

ACGGATACAGCAGAAATACAAAAAACCATAAGAGAATACTATGAACAACTGTATGGCAAC

AAGTTTGACAATCTGGAAGAAATGGACAATTTTCTAGAATCTTACAGCTTGCCAAAACTG

AATCAAGCAGAAACAGACCAACTGAACAGACCGATCACTAGAAATGAAATTGAAGAGGTC

ATAAAATCACTCCCTACAAATAAAAGTCCAGGACCAGATGGCTTCACAGGTGAATTTTAT

CAAACATATAAAGAGGAATTGGTGCCCATCCTCCTTAAACTCTTTCAAAAGGTTGAAGAA

GAAGGAATACTCCCAAAGACATTCTATGAGGCCACCATCACCCTCATTCCAAAACCAGAC

AGAGATACCACCAAAAAAGAAAACTATCGCCCAATATCATTGATGAATATAGATGCAAAA

ATTCTCAACAAAATCTTAGCCAACCGAATCCAACAACATATCAAAAAAATTATACACCAT

GACCAGGTTGGGTTCATCCCAGGTTCACAAGGATGGTTCAACATACGCAAATCAATCAGC

ATCATACACCACATTAACAAAAAAAAGGTCAAAAATCATATGATCATCTCAATAGACGCA

GAAAAAGCATTTGACAAAGTTCAACATCCATTCATGATCAAGACCCTCGCCAAAGTGGGT

ATAGAGGGAACATTCCTGAATATAATCAAAGCCATTTATGATAAACCCACAGCAAATATA

ATCCTCAATGGGGAAAAACTGAAAGCCTTCTCACTCAAATCTGGAACAAGACAGGGATGC

CCACTCTCACCACTGCTCTTCAACATCGTTTTGGAAGTCTTAGCCACAGCAATTAGACAA

ACAAAAGAAATAAAAGGCATCCATATAGGAAGAGAAGAGATCAAACTGTCACTGTATGCA

GATGATATGATACTATACCTAGAAAACCCTAAGGACTCAACCCCAAAACTCCTTGAACTG

ATTAATAAATTCAGCAAAGTGGCAGGATATAAGATTAACATTCAGAAGTCAGTTGCATTT

CTGTATACCAGCAATGAAACATTAGAAAAGGAATACAAAAAAATGATACCTTTTAAAATT

GTACCTCACAAAATCAAATACCTCGGAATACACCTGACCAAAGAGGTAAAGGACCTATAT

GCCGAGAACTATAAAACTTTAATCAAAGAAATCAAAGAAGATGTAAAGAAATGGAAAGAT

ATTCCATGTTCCTGGATTGGAAAAATCAATATTGTGAAAATGGCCATCCTACCCAAAGCA

ATCTACAGATTCAATGCAATCCCTATCAAATTACCCATGACATTGTTCACAGAACTAGAA

CAAACAATCCAAACATTTATATGGAACCACAAAAGACCCAGAATCGCCAAAGCAATCCTG

AGAAACAAAAACCAAGCAGGAGGCATAACTCTCCCAGACTTCAAGAAATACTACAAAGCC

ACAGTCATCAAAACAGTGTGGTACTGGTATCAAAACAGACAGACAGACCAATGGAACAGA

ATAGAGAATCCGGAAATAAACCCTGACACCTATGGTCAATTAATCTTTGACAAGGGAGGC

AGGAACATCAAATGGGAAAAGGAAAGTCTATTCAGCAAGCATTGCTGGGAAACCTGGACA

GCTGCATGCAAAGCAATGAAACTAGAACACACCCTCACACCATGCACAAAAATAAACTCC

AAATGGCTGAAAGACTTAAATATACGACAGGACACCATCAAACTCCTAGAAGAAAACATA

GGCAAAACACTCTCTGACATCAACATCATGAATATTTTCTCAGGTCAGTCTCCCAAAGCA

ATAGAAATTAGAGCAAAAATAAACCCATGGGACCTCATCAAACTGAAAAGCTTTTGCACA

GCAAAGGAAACCAAAAAGAAAACAAAAAGACAACTTACAGAATGGGAGAAAATAGTTTCA

AATGATGCAACTGACAAGGGCTTAATCTCTAGAATATACAAGCAACTTATACAACTCAAC

AGCAAAAAAGCCAATCAATCAATGGAAAAATGGGCAAAAGACCTGAATAGACATTTCTCC

AAGGAAGATATACAGATGGCCAACAAACACATGAAAAAATGCTCAACATCGCTGATTATA

AGAGAAATGCAAATCAAAACTACCATGAGATACCACCTCACACCAGTCAGAATGGCCATC

ATTAATAAATCCACAAATAACAAGTGCTGGAGGGGCTGTGGAGAAAAGGGAACCCTCCTG

CACTGCTGGTGGGAATGTAAACTGGTACAGCCACTATGGAGAACAGTTTGGAGATACCTT

AGAAATCTATACATAGAACTTCCATATGACCCTGCAATCCCACTCTTGGGCATCTATCCG

GACAAAGCTCTACTTAAAAGAGACACATGCACCCGCATGTTCATTGCAGCCCTATTCACA

ATAGCCAGGACATGGAAACAATCCAAATGTCCATCGACAGATGATTGGATTCGGAAGAGG

TGGTATATATACACAATGGAATACTACTCAGCCATAAAAAAGGATGACATAATGCCATTT

GCAGCAACATGGATGGAACTAGAGAATCTCATCTGAGTGAAATGAGCCAGAAAGACAAAG

ACAAATACCATATGATATCACTTATAACTGGAATCTAATATCCAGCACAAATGAACATCT

CCTCAGAAAAGAAAATCATGGACTTGGAGAAGAGACTTGTGGTTGCCTGATGGGAGGGGG

AGGGAGTGGGAGGGATCGGGAGCTTGGGCTTATCAGACACAACCTAGAATAGATTTATAA

GGAGATCCTGCTGAATAGCATTGAGAACTATGTCTAGATACTCATGTCGCAACAGAAGAA

AGGGTGGGGGAAAAAACTGTAACTGCAATGTATACATCTAAGGATGACCTGACCCCCTTG

CTGTACAGTGGGAAAATAATAAAAAAAAAAAAAAAAAAAAAAAAAAAAAAAAAAAA

>L1D12#LINE/L1D

GAGAGGACAAGATGGCGGAGGAGTAGGGGGACACGCTCGCCCTCTCCCACAAACACAACA

AAAAAAGCACATCTACAGAATAAATGACTCGCACAGAACAGCAACCAATCGCTGGCAGAG

GAACCTAAACTCCAATAACGGCAAGAAGTTCGTGACATTATTGGGCAGAACGGGAGAAAA

GAGGAGAGTGAGAGAAGGTGAATCCGAGCGGGACGGGCGCTCCCGAAAGGGAACTGCGGA

GGAGAAAGGGATCCCGCACCCTGGAAAGTCACCTACCGGGGGAAAGATCAAACGAACCGG

AGGAATCTCCAGATGCAGAGAAGAGTGTAGCAGTAAGTTGGAGTACGGAAAAGCCGATCA

AGAACCCAACGGACCATCTGAACTACGGGCACAGTCACCAAAAATTGAGACGCCTGGGTG

GGGGCTGGGCACCGAGCCTCGCTCCAAGGTTAGTCCCCGGAAAGGGCCGGGGGACCCTGG

GTGGGGGCTGGGCACCGAGACCTCGCCAGGTTAGTCCCAGAAGGGCGGGGGGCACCGAGA

CCTCGGCTCCAGAGTTAGTCCCCGGGCTGGGGGCGGGGCAGAGCGGAAACTGCTTGGGAG

GTCTAGAAACCATTTGACGGGGCAGAGACTGCCTGGGAGACTAGAAAACAAAGCTGTCGC

AGAGGAAGGGAGCAATACTCTAGGGGCGGGGAAGTGGAAAGCCACATCAGAGGGAACCTG

GGAGAAGAGCCTGGTCTGCGCCCGTGCTGGGGAGGGGAGAGAAGAAGGGGTGGGTCCCCA

TAGAATACCCCCCACGCCACAGCAAGCTTACAGGCCCGCTAGCTAGCTGAAAGCTGTGCT

TCCCAGTGCATCCCCTCCCCCCACCCCCGCCACCCCTACGCTCTCCCGGACCTGGGGCTG

CCTGCCATCCAGGAGGGCTGGCCTCAACAATTGCCTGAAGCCTACCACCGCAGGGGCTGT

CCCTGCACAGGCCTGCTTGCCCTTTGGAGGGGCTACACTTCCGCAGAGCAGCACCAAACA

CCACCAGCCCCCGAGAAAAGGCCTGCAGCCCAGAAAAGCTAGAACAAGCTTAGCCAGGCT

GTGAATAGATCGCCTAATTCTCGGACGGTTTTTCTGAGTCGGTGCCCCGGGGAGGAGCCT

CTTGGGTTTCCAACGGCCCTGCTACCCGCCCAAGCCCCCAGGGGGTGCCCCACTCCCGTG

GAATAGCTGCTCAGCACCACCAGCCCCCTGGAGGACCCCCTGCAGCCCAGAAAAGCTGCA

ACAAGCTCGGCCAGACTGTGAAAAGATCCGCCTACATTCTCAGGCTGTCCTTCTGAGTTG

GGCTGCCCTAGGGAAGAGCCTCTTAGGTTCTCAGTGACCCAGATAGCTGCTCCAGCCCCC

AGGGGGTGATGCACTCCTGAGGAGCAGCTGCCCAACACCGCCAACCCCCTGCAAGAACCC

CACAGCCTAAAAACACCAGAGCAAGCTCTGCATGACCAAGTGAAATCTGCTACCATCGTG

GTGTGGACCTCCCAGTCCTGTCTGCCCTCAGGAAGTCCTCCTTTGCTTCAAAGAAACACT

GTTAGCCCCATCAACACTCCAGAAAAGCCACACTGCCTCAAAAAAGATTGACCAACAACG

CCAGCCCTCAGGAAATATTCCACGGCAGTGACAAGGCAAACACTGCCCGATACGGAGAGT

ACAACTCCCTCAGGAGAAAGAAAACAACAAGCAAGATGAAGAAGCTGAGAAACCACCCCC

AGTCAAACCAACAGGAGAACTCACCTAAAACAGTCAACAATGAAACAGATCTCTGCAGTC

TGACAGACCTGGAGTTCAAAAGAGAAATAGTGAAAATACTGAAGGAATTAAGAGAAGATA

TGAACAGTAATGCAGATACCCTCAGAAAGGAACTAGAAAATATAAGGAGGAGCCAAGAAA

AACTAGAACATTCATTTGCAGAGATGCAAACTGAACTAGGGGCAGTAAAAACCAGAATGA

ATAATGCAGAAGAACGAATCAGTGATATGGAAGATAGAATAATGGAAATCACTCAATCTG

GTCAACAGACAGAAAACCGAATCAAAAAACTGGAAAGCAATATAAGAGACCTATGGGATA

ATATAAAGCGGGCCAATCTACGCATAATAGGAATTCCAGAAGGAGTAGAAAAAGATAAGG

GGATGGAAAATATATTTGAAGAAATTATCGCTGGAAACTTCCCAAATCTAAAGGATACTG

GGTTCAAGATACAAGAAGCACAGAGGGCCCCAAACAAACTGAACCCAAACAGACCCACAC

CAAGACACATCATAATAAAAATGGCAAAAGTTAGTGATAAAGAGAGGATCCTAAAGGCAG

CAAGAGAAAAACAGAATGTTACCTACAAGGGAACCCCCATAAGAATATCAGCTGATTTCT

CTACAGAAACACTACAGGCCAGGAGGGAATGGCAAGAGATATTTAAAGTGCTAAAAGGAA

AAAATATGCAACCTAGAATACTCTATCCAGCAAGAATATCATTTAAAATAGAAGGGGAAA

TAAAAATTTTTCCCAACAAACAAAAACTTAAAGAATACAGCAACACAAAACCCAGGTTAA

AGGAAATATTGAAAGGGCTTCTCTAAACCAAAAAGAAAGGAAGGAAAGGGAAGAAAAAAG

AAAAGAAAAAAAAAAAAGAAGAAGAAGAGGAAGAACTAGGACTGAGGAAACCGCAATCAG

AGAGCAGTCACTCAAATAAGCCAGCATACAGATTTAATCATGAACATGCTTCAAACAAAA

TAAAATTAAAAAGAAAAAAATAAAAAAGAGTCATCAAAACCATAAAATGTGGGCAAGGGA

TGTTAGGAAGTAAATAACCCTTTTTGTTTGTTTGTATGTCTCTCTTCTTAATTTTAATAT

AGTAATGAAGTGTTTGAACTTACAGGACCATCAGGCTAAAACACACAATTATGGGAAGGG

GTTAGCATACTTAAAAAACAGGGCAACCACAAGCCAAAACCAAATATTGCATTTGCAAAA

AAGAAAAAAAAAAACACTCAAGCAGATAATAACAGGAGACCATCCAACCAAAAAAAAAAA

AAAAAAAAAAAAAAAGGAAGAATGGAGAACCATAGAATCAACTGGAACACGAGGTTCAAA

TGGCAATAAATAATCATCTATCAATTATCACCTTAAATGTCAATGGACTGAATGCCCCAA

TCAAAAGACACAGAGTGGCTGAGTGGATAAAAAGGCAAAAACCTTCAATATGCTGCCTAC

AAGAAACTCACCTTAGGACAAAAGATACATATAGATTGAAAGTGAAAGGGTGGGGAAAAA

TATTTCACGCCAATAGACATGACAGAAAAGCAGGAGTCGCAACGCTCATATCAGACAAAA

TAGACTTTAAAACAAAAGACATAAAGAAAGACAAAGAAGGACACTATTAATGATTAAGGG

ATCCATCCAAGGAGAGGATGTTACTATCGTCAACATATATGCCCCAAATACAGGAGCACC

CAGATACATACAACAAATATTAACAGACATAAAGGGAGATATTGATGAGAATACAATCAT

AGTAGGAGACCTTAATACCCCCCTCACATCAATGGACAGATCCTCTAGACAGAAAACCAA

TAAAGCAACAGAGATCCTAAAGGAAACAATAGAAAAGTTAGACTTAATTGATATCTTCAG

GACACTACATCCAAAAAAAGCAGAATACACATTCTTCTCAAATGCTCATGGAACATTCTC

AAGAATCGACCACATATTGGGACACAAAGCGAATCTCAATAAATTTAGGAGCATAGAAAT

TATCTCAAGTATCTTCTCTGACCACAATGCCATGAAATTAGAAATCAACCATGGGAAAAG

CAAAGAGAAAAAACCTACTCCATGGAGACTAAACAACATGCTACTAAAAAACCAATGGGT

CAATGAGGAAATCAAGAAGGAAATTAAAAACTACCTTGAAACAAATGATAATGAAGACAC

AACCTCTCAAAATCTATGGGATGCTGCGAAAGCAGTGCTCAGAGGGAAATTTATAGCAAT

CAGGCCTTTCTCAAAAAAGAAGAAAGATCCCAAATTGACAACTTAACCCTCCACCTAAAC

GAATTAGAAAAAGAAGAACAAAAAAGTCCTAAAGTCAGCAGAAGGAAGGAAATTATAAAG

ATCAAAGAAGAAATCAATAAAATAGAGACTCAAAAAACAATAGAGAAAATTAATAAAACC

AAGAGCTGGTTCTTTGAAAAGGTGAACAAAATTGACAAACCCCTGGCCAGACTCACTAAA

AAGAGGAGAGAAAGAACCCAAATAACCAAAATTATAAATGAAAAAGGAGAAATCACAACG

GATACAGCAGAAATACAAAAAACCATAAGAGAATACTATGAACAACTATATGGCAACAAG

TTTGACAATCTGGAAGAAATGGACAATTTTCTAGAATCTTACAGCCTGCCAAAACTGAAT

CAAGCAGAAACAGACCAACTGAACAGACCGATCACTAGAAATGAAATTGAAGAGGTCATA

AAATCACTCCCTACAAATAAAAGTCCAGGACCAGATGGCTTCACAGGTGAATTTATCAAA

CATATAAAGAGGAATTGGTGCCCATCCTCCTTAAACTCTTTCAAAAGGTTGAAGAAGAAG

GAATACTCCCAAAGACATTCTATGAGGCCACCATCACCCTCATTCCAAAACCAGACAGAG

ATACCACCAAAAAAGAAAACTATCGCCCAATATCATTGATGAATATAGATGCAAAAATTC

TCAACAAAATCTTAGCCAACCGAATCCAACAACATACCAAAAAAATTATACACCATGACC

AGGTTGGGTTCATCCCAGGTTCACAAGGATGGTTCAACATACGCAAATCAATCAGCATCA

TACACCACATTAACAAAAAAAAAGTCAAAAATCATATGATCATCTCAATAGACGCAGAAA

AAGCATTTGACAAAGTCAACATCCATTCATGATCAAGACCCTCGCCAAAGTGGGTATAGA

GGGAACATTCCTGAATATAATCAAAGCCATTTATGATAAACCCACAGCAAATATAATCCT

CAATGGGGAAAAACTGAAAGCCTTCTCACTCAAATCTGGAACAAGACAGGGATGCCCACT

CTCACCACTGCTCTTCAACATAGTTTTGGAAGTCCTAGCCACAGCAATTAGACAAACAAA

AGAAATAAAAGGCATCCATATAGGAAGAGAAGAGATCAAACTGTCACTGTATGCAGATGA

CATGATACTATACCTAGAAAACCCTAAGGACTCAACCCCAAAACTCCTTGAACTGATTAA

TAAATTCAGCAAAGTAGCAGGATATAAGATTAACATTCAGAAGTCAGTTGCATTTCTGTA

TACCAGCAATGAAACATTAGAAAAGGAATACAAAAATACGATACCTTTTAAAATTGTACC

TCACAAAATCAAATACCTCGGAATACACCTGACCAAGGAGGTAAAGGACCTATATGCCGA

GAACTATAAAACTTTAATCAAAGAAATCAAAGAAGATGTAAAGAAATGGAAAGATATTCC

ATGTTCCTGGATTGGGAAAATCAATATTGTAAAAATGGCCATACTACCCAAAGCAATCTA

CAGATTCAATGCAATCCCTATCAAATTACCCATGACATTTTTCACAGAACTAGAACAAAC

AATCCAAACATTTATATGGAACCACAAAAGACCCAGAATCGCCAAAGCAATCCTGAGAAA

CAAAAACCAAGCAGGAGGCATAACTCTCCCAGACTTCAAGAAATACTACAAAGCCACAGT

CATCAAAACAGTGTGGTACTGGTATCAAAACAGACAGACAGACCAATGGAACAGAATAGA

GAATCCGGAAATAAACCCTGACACCTATGGTCAATTAATCTTTGACAAGGGAGGCAAGAA

CATAAAATGGGAAAAGGAAAGTCTATTCAGCAAGCATTGCTGGGAAACCTGGACAGCTGC

ATGCAAAGCAATGAAACTAGAACACACCCTCACACCATGCACAAAAATAAACTCCAAATG

GCTGAAAGACTTAAATATACGACAGGACACCATCAAACTCCTAGAAGAAAACATAGGCAA

AACACTCTCTGACATCAACATCATGAATATTTTCTCAGGTCAGTCTCCCAAAGCAATAGA

AATTAGAGCAAAAATAAACCCATGGGACCTCATCAAACTGAAAAGCTTTTGCACAGCAAA

GGAAACCCAAAAGAAAACAAAAAGACAACTTACAGAATGGGAGAAAATAGTTTCAAATGA

TGCAACTGACAAGGGCTTAATCTCTAGAATATATAACAACTTATACAACCAACAGCAAAA

AACCAATCAATCAATGGAAAAATGGGCAAAAGACCTGAATAGACATTTCTCCAAAGAAGA

TATACAGATGGCCAACAAACACATGAAAAAATGCTCAACATCGCTGATTATAAGAGAAAT

GCAAATCAAAACTACCATGAGATACCACCTCACACCAGTCAGAATGGCCATCATTAATAA

ATCCACAAATAACAAGTGCTGGAGGGGCTGTGGAGAAAAGGGAACCCTCCTGCACTGCTG

GTGGGAATGTAAACTGGTACAGCCACTATGGAGAACAGTTTGGAGATACCTTAGAAATCT

ATACATAGAACTTCCATATGACCCTGCAATCCCACTCTTGGGCATCTATCCGGACAAACT

CTACTTAAAAGAGACACATGCACCCGCATGTTCATTGCAGCACTATTCACAATAGCCAGG

ACATGGAAACAACCCAAATGTCCATGACAGATGATTGGATTCGGAAGAGTGGTATATATA

CACAATGGAATACTACTCAGCCATAAAAAAGATGACATAATGCCATTTGCAGCAACATGG

ATGGAACTAGAGAATCTCATCTGAGTGAAATGAGCCAGAAAGACAAAGACAAATACCATA

TGATATCACTTATAACTGGAATCTAATATCCAGCACAAATGAACATCTCCTCAGAAAAGA

AAATCATGGACTTGGAGAAGAGACTTGTGGCTGCCTGATGGGAGGGGGAGGGAGTGGGAG

GGATCGGGAGCTTGGGCTTATCAGACACAACTTAGAATAGATTTACAAGGAGATCCTGCT

GAATAGCATTGAGAACTATGTCTAGATACTCATGTTGCAACAGAAGAAAGGGTGGGGGAA

AAATGTAATTGAATGTATACATGTAAGGATAACCTGACCCCCTTGCTGTACAGTGGGAAA

ATAAAAAAAAAAAAAAAAAAAAAA

>L1D13#LINE/L1D

GAGAGGACAAGATGGCGGAGGAGTAGGGGGACACGCTCGCCCTCTCCCACAAACACAACA

AAAAAAGCACATCTACAGAATAAATGACTCGCACAGAACAGCAACCAATCGCTGGCAGAG

GAACCTAAACTCCAATAACGGCAAGAAGTTCGTGACATTACTGGGCAGAACGGGAGAAAA

GAGGAGAGTGAGAGAAGGTGAATCCGAGCGGGACGGGCGCTCCCGAAAGGGAACTGCGGA

GGAGAAAGGGATCCCGCACCCTGGAAAGTCACCTACCGGGGGAAAGATCAAACGAACCGG

AGGAATCTCCAGATGCAGAGAAGAGTGTAGCAGTAAGTTGGAGTACGAAAAGCCGATCAA

GAACCGAACGGACCATCTGAACTACGGGCACAGTCACCAAAAATTGAGACGCCTGGGTGG

GGGCTGGGCACCGAGACCTCGGCTCGAGGTTAGTCCCCGAGGGGGGGGGGAGTCGGAGAA

CGGAACTGTTGGGAGGTTTGAGGGGCAGAGACTGCCTGGGAGACTAGAAAACAAAGCTGT

CGCAGAGGAAGGGAGCAATACTCTAGGGGCGGGGAAGTGGAAAGCCACATCAGAGGGAAC

CTGGGAGAAGAGCCTGGTCTGCGCCCGTGCTGGGGAGGGGAGAGAAGAAGGGGTGGGTCC

CCATAGAATACCCCCCACGCCACAGCAAGCTTACAGGCCCGCTAGCTAGCAGAAAGCTGT

GCTTCCCAGTGCATCCCCTCCCCCCACCCCCGCCACCCCCTACGCTCTCGCCGGACCTGG

GGCTGCCTGCCATCCAGGAGGGCTGGCCTCAACAATTGCCTGAAGCCTACCACCGCAGGG

GCTGTCCCTGCACAGGCCTGCTTGCCCTTTGGAGGGGCTACACTTCCGCAGAGCAGCACC

AAACACCACCAGCCCCCGAGAAAAGGCCTGCAGCCCAGAAAAGCTAGAACAAGCCTAGCC

AGGCCGTGAATAGATCTGCCTAATTCTCGAGTTTTCTGAGTGTCCCGGGAGAGCCTCTTT

TCAGCCCGCTACCACCCCAGGGGGTGCCCACTCCGGGAAAGCTGCCAGACCCAGCCCCTG

AGACCCCTGCAGCCCAGAAAAGCTGCAACAAGCTTGGCCAGACTGTGAAAAGATCTGCCT

ACATTCTCAGGCGTCCTTCTGAGTTGGGCTGCCCTAGGGAAGAGCCTCTTAGGTTCTCAG

TGACCCAGATAGCTGCTCCAGCCCCCAGGGGGTGCTGCACTCCTGAGGAACAGCTGCCCA

ACACCGCCAACCCCCTGCAAGAACCCCACAGCCTAAAAACACCAGAGCAAGCTCTGCATG

ACCAAGTGAAATCTGCTACCATCGTGGTGTGGACCTCCCAGTCCTGTCTGCCCTCAGGAA

GTCCTCCTTTGCTTCAAAGAAACACTGTTAGCCCCATCAACACTCCAGAAAAGCCACACT

GCCTCAAAAAAGATTGACCAACAACGCCAGCCCTCAGGAAATATTCCACGGCAGTGACAA

GGCAAACACTGCCCGATCACGGAGAGTACAACTCCCTCAGGAGAAAGAAAACAACAAGCA

AGATGAAGAAGCTGAGAAACCACCCCCAGTCAAACCAACAGGAGAACTCACCTAAAACAG

TCAACAATGAAACAGATCTCTGCAGTCTGACAGACCTGGAGTTCAAAAGAGAAATAGTGA

AAATACTGAAGGAATTAAGAGAAGATATGAACAGTAATGCAGATACCCTCAGAAAGGAAC

TAGAAAATATAAGGAGGAGCCAAGAAAAACTAGAACATTCATTTGCAGAGATGCAAACTG

AACTAGGGCAGTAAAAACCAGAATGAATAATGCAGAAGAACGAATCAGTGATATGGAAGA

TAGAATAATGGAAATCACTCAATCGGTCACAGACAGAAAACCGAATCAAAAAACTGGAAA

GCAATATAAGAGACCTATGGGATAATATAAAGCGGGCCAATCTACGCATAATAGGAATTC

CAGAAGGAGTAGAAAAAGATAAGGGATGGAAAATATATTTGAAGAAATTATCGCTGGAAA

CTTCCCAAATCTAAAGGATACTGGGTTCAAGATACAAGAAGCACAGAGGGCCCCAAACAA

ACTGAACCCAAACAGACCCACACCAAGACACATCATAATAAAAATGGCAAAAGTTAGTGA

TAAAGAGAGGATCCTAAAGGCAGCAAGAGAAAAACAGAATGTTACCTACAAGGGAACCCC

CATAAGAATATCAGCTGATTTCTCTACAGAAACACTACAGGCCAGGAGGGAATGGCAAGA

GATATTTAAAGTGCTAAAAGGAAAAAATATGCAACCTAGAATACTCTATCCAGCAAGAAT

ATCATTTAAAATAGAAGGGGAAATAAAAATTTTTTCCAACAAACAAAAACTTAAAGAATA

CAGCAACACAAAACCCAGGTTAAAGGAAATATTGAAAGGGCTTCTCTAAACCAAAAAGAA

AGGAAGGAAAGGGAAGAAAAAAGAAAAAAAAAAAAAAAGAAGAAGAGGAAGAACTAGGAC

TGAGGAAACCGCAATCAGAGAGCAGTCACTCAAATAAGCCAGCATACAGATTTAATCATG

AACATGCTTCAAACAAAATAAAATTAAAAAGAAAAAAATAAAAAAGAGTCATCAAAACCA

TAAAATGTGGGCAAGGGATGTTAGGAAGTAAATAACCCTTTTTGTTTGTTTGTTGTATGT

TTCTCTTCTTAATTTTAATATAGTAATGAAGTGTTTGAACTTACAGGACCATCAGGCTAA

AACACACAATTATGGGAAGGGGTTAGCATACTTAAAAAACAGGGCAACCACAAGCCAAAA

CCAAATATTGCATTTGCAAAAAATGAAAAAAAAAAACACTCAAGCAGATAATAACAGGAG

ACCATCCAACCAAAAAAAAAAAAAAAAAAAAAAAGGAAGAATGGAGAACCATAGAATCAA

CTGGAACACGAGGTTCAAATGGCAATAAATAATCATCTATCAATTATCACCTTAAATGTC

AATGGACTGAATGCCCCAATCAAAAGACACAGAGTGGCTGAGTGGATAAAAAGGCAAAAA

CCTTCAATATGCTGCCTACAAGAAACTCACCTTAGGACAAAAGATACATATAGATTGAAA

GTGAAAGGGTGGGGAAAAATATTTCACGCCAATAGACATGACAGAAAAGCAGGAGTCGCA

ACCTCATATCAGACAAAATAGACTTTAAAACAAAAGACATAAAGAAAGACAAAGAAGGAC

ACTACTTAATGATTAAGGGATCCATCCAAGGAGAGGATGTTACTATCATCAACATATATG

CCCCAAATATAGGAGCACCCAGATACATACAACAAATATTAACAGACATAAAGGGAGATA

TTGATGAGAATACAATCATAGTAGGAGACCTTAATACCCCCCTCACATCAATGGACAGAT

CCTCTAGACAGAAAACCAATAAAGCAACAGAGATCCTAAAGGAAACAATAGAAAAGTTAG

ACTTAATTGATATCTTCAGGACACTACATCCAAAAAAATCAGAATACACATTCTTCTCAA

ATGCTCATGGAACATTCTCAAGAATCGACCACATATTGGGACACAAAGCTAACCTCAATA

AATTTAGGAGCATAGAAATTATCTCAAGTATCTTCTCTGACCACAATGCCATGAAATTAG

AAATCAACCATGGGAAAAGGAAAGAGAAAAAACCTACTACATGGAGACTAAACAACATGC

TACTAAAAAACCAATGGGTCAATGAGGAAATCAAGAAGGAAATTAAAAACTACCTTGAAA

CAAATGATAATGAAGACACAACCTCTCAAAATCTATGGGATGCTGCGAAAGCAGTGCTCA

GAGGGAAATTTATAGCAATACAGGCCTTTCTCAAAAAAGAAGAAAGATCCCAAATTGACA

ACTTAACCCTCCACCTAAACGAATTAGAAAAAGAAGAACAAAAAAGTCCTAAAGTCAGCA

GAAGGAAGGAAATTATAAAGATCAAAGAAGAAATCAATAAAATAGAGACTCAAAAAACAA

TAGAGAAAATTAATAAAACCAAGAGCTGGTTCTTTGAAAAGGTGAACAAAATTGACAAAC

CCCTGGCCAGACTCACTAAAAAGAGGAGAGAAAGAACCCAAATAACCAAAATTATAAATG

AAAAAGGAGAAATCACAACGGATACAGCAGAAATACAAAAAACCATAAGAGAATACTATG

AACAACTATATGGCAACAAGTTTGACAATCTGGAAGAAATGGACAATTTTCTAGAATCTT

ACAGCCTGCCAAAACTGAATCAAGAGAAACAGACCAACTGAACAGACCGATCACTAGAAA

TGAAATTGAAGAGGTCATAAAATCACTCCCTACAAATAAAAGTCCAGGACCAGATGGCTT

CACAGGTGAATTCTATCAAACATATAAAGAGGAATTGGTGCCCATCCTCCTTAAACTCTT

TCAAAAGGTTGAAGAAGAAGGAATACTCCCAAAGACATTCTATGAGCCACCATCACCCTC

ATTCCAAAACCAGACAGAGATACCACCAAAAAAGAAAACTATCGCCCAATATCATTGATG

AATATAGATGCAAAAATTCTCAACAAAATCTTAGCCAACCGAATCCAACAACATATCAAA

AAAATTATACACCATGACCAGGTTGGGTTCATCCCAGGTTCACAAGGATGGTTCAACATA

CGCAAATCAATCAGCATCATACACCACATTAACAAAAAAAAAGTCAAAAATCATATGATC

ATCTCAATAGACGCAGAAAAAGCATTTGACAAAGTCCAACATCCATTCATGATCAAGACC

CTCGCCAAAGTGGGTATAGAGGGAACATTCCTGAATATAATCAAAGCCATTTATGATAAA

CCCACAGCAAATATAATCCTCAATGGGGAAAAACTGAAAGCCTTCTCACTCAAATCTGGA

ACAAGACAGGGATGCCCACTCTCACCACTGCTCTTCAACATAGTTTTGGAAGTCCTAGCC

ACAGCAATTAGACAAACAAAAGAAATAAAAGGCATCCATATAGGAAGAGAAGAGATCAAA

CTGTCACTGTATGCAGATGACATGATACTATACATAGAAAACCCTAAGGACTCAACCCCA

AAACTCCTTGAACTGATTAATAAATTCAGCAAAGTAGCAGGATATAAGATTAACATTCAG

AAGTCAGTTGCATTTCTGTATACCAGCAATGAAATATTAGAAAAGGAATACAAAAATACG

ATACCTTTTAAAATTGCACCTCACAAAATCAAATACCTCGGAATACACCTGACCAAGGAG

GTAAAGGACCTATATGCCGAGAACTATAAAACTTTAATCAAAGAAATCAAAGAAGATGTA

AAGAAATGGAAAGATATTCCATGTTCCTGGATTGGGAAAATCAATATTGTAAAAATGGCC

ATACTACCCAAAGCAATCTACAGATTCAATGCAATCCCTATCAAATTACCCATGACATTT

TTCACAGAACTAGAACAAACAATCCAAACATTTATATGGAACCACAAAAGACCCAGAATC

GCCAAAGCAATCCTGAGAAACAAAAACCAAGCAGGAGGCATAACTCTCCCAGACTTCAAG

AAATACTACAAAGCCACAGTCATCAAAACAGTGTGGTACTGGTATCAAAACAGACAGACA

GACCAATGGAACAGAATAGAGAATCCGGAAATAAACCCTGACACCTATGGTCAATTAATC

TTTGACAAGGGAGGCAAGAACATAAAATGGGAAAAAGAAAGTCTATTCAGCAAGCATTGC

TGGGAAACCTGGACAGCTGCATGCAAAGCAATGAAACTAGAACACACCCTCACACCATGC

ACAAAAATAAACTCCAAATGGCTGAAAGACTTAAATATACGACAGGACACCATCAAACTC

CTAGAAGAAAACATAGGCAAAACACTCTCTGACATCAACATCATGAATATTTTCTCAGGT

CAGTCTCCCAAAGCAATAGAAATTAGAGCAAAAATAAACCCATGGGACCTCATCAAACTG

AAAAGCTTTTGCACAGCAAAGGAAACCCAAAAGAAAACAAAAAGACAACTTACAGAATGG

GAGAAAATAGTTTCAAATGATGCAACGACAAGGGCTTAATCTCTAGAATATATAACAACT

TATACAACCAACAGCAAAAAACCAATCAATCAATGGAAAAATGGGCAAAAGACCTGAATA

GACATTTCTCCAAAGAAGATATACAGATGGCCAACAAACACATGAAAAAATGCTCAACAT

CGCTGATTATAAGAGAAATGCAAATCAAAACTACCATGAGATACCACCTCACACCAGTCA

GAATGGCCATCATTAATAAATCCACAAATAACAAGTGCTGGAGGGGCTGTGGAGAAAAGG

GAACCCTCCTGCACTGTTGGTGGGAATGTAAACTGGTACAGCCACTATGGAGAACAGTTT

GGAGATACCTTAGAAATCTATACATAGAACTTCCATATGACCCTGCAATCCCACTCTTGG

GCATCTATCCGGACAAAACTCTACTTAAAAGAGACACATGCACCCGCATGTTCATTGCAG

CACTATTCACAATAGCCAGGACATGGAAACAACCCAAATGTCCATCGACAGATGATTGGA

TTCGGAAGAGTGGTATATATACACAATGGAATACTACTCAGCCATAAAAAAGAATGACAT

AATGCCATTTGCAGCAACATGGATGGAACTAGAGAATCTCATACTGAGTGAAATGAGCCA

GAAAGACAAAGACAAATACCATATGATATCACTTATAACTGGAATCTAATATCCAGCACA

AATGAACATCTCCTCAGAAAAGAAAATCATGGACTTGGAGAAGAGACTTGTGGCTGCCTG

ATGGGAGGGGGAGGGAGTGGGAGGGATCGGGAGCTTGGGCTTATCAGACACAACTTAGAA

TAGATTTACAAGGAGATCCTGCTGAATAGCATTGAGAACTTGTCTAGATACTCATGTTGC

AACAGAAAAAGGGTGGGGGAAAAATGTAATTGTAATGTATACATGTAAGGATAACCTGAC

CCCCTTGCTGTACAGTGGGAAAATAAAAAAAAAAAAAAAAAAAAAAAAAAAA

>L1D14#LINE/L1D

GAGAGAGGACAAGATGGCGGAGGAGTAGGGGGACACGCTCGCCCTCTCCCACAAACACAA

CAAAAAAAGCACATCTACAGAATAAATGACTCGCACAGAACAGCAACCAATCGCTGGCAG

AGGAACCTAAACTCCAATAACGGCAAGAAGTTCGTGACATTATTGGGCAGAACGGGAGAA

AAGAGGAGAGTGAGAGAAGGTGAATCCGAGCGGGACGGGCGCTCCCGAAAGGGAACTGCG

GAGGAGAAAGGGATCCCGCACCCTGGAAAGTCACCTACCGGGGGAAAGATCAAACGAACC

GGAGGAATCTCCAGATGCAGAGAAGAGTGTAGCAGTAAGTTGGAGTACGGAAAAGCCGAT

CAAGAACCCAACGGACCATCTGAACTACGGGCACAGTCACCAAAAATTGAGACGCCTGGG

TGGGGGCTGGGCACCGAGACCTCGGCTCCAGAGGTTAGTCCCCGAGAAAGGGCCGGGGGA

CGCCTGGGTGGGGGCTGGGCACCGAGACCTCTCAGGTTATCCCGAGAGGGCCGGGGGGGC

TGGGCACCGAGACCTCGGCTCCAGAGTTAGTCCCCGGGCTAGGGGGGCGGGGCAGAGCGG

AAACTGCTTGGGAGGTCTAGAAACCATTTGACGGGGCAGAGACTGCCTGGGAGACTAGAA

AACAAAGCTGTCGCAGAGGAAGGGAGCAATACTCTAGGGGCGGGGAAGTGGAAAGCCCAT

CAGAGGGAACCTGGGAGAAGAGCCTGGTCTGCGCCCGTGCTGGGGAGGGGAGAGAAGAAG

GGGTGGGTCCCCATAGAATACCCCCCACGCCACAGCAAGCTTACAGGCCCGCTAGCTAGC

TGAAAGCTGTGCTTCCCAGTGCATCCCCTCCCCCCACCCCCGCCACCCCTACGCTCTCAC

CGAACCTGGGGCTGCCTGCCATCCAGGAGGGCTGGCCTCAACAATTGCCTGAAGCCTACC

ACCGCAGGGGCTGTCCCTGCACAGGCCTGCTTGCCCTTTGGAGGGGCTACACTTCCGCAG

AGCAGCACCAAACACCACCAGCCCCCGAGAAAAGGCCTGCAGCCCAGAAAAGCTAGAACA

AGCTTAGCCAGGCTGTGAATAGATCGGCCTAATTCTCGGACGGTTTTTCTGAGTCGGGCT

GCCCCGGGGAGGAGCCTCTTGGTTTCCAACGGCCCTGCTACCCGCCCAAGCCCCCAGGGG

GTGCCCCACTCCCGTGGAATAGCTGCTCAGCACCACCAGCCCCCTGGAGGAGCCCCTGCA

GCCCAGAAAAGCTGCAACAAGCTCGGCCAGACTGTGAAAAGATCCGCCTACATTCTCAGG

CTGTCCTTCTGAGTTGGGCTGCCCTAGGGAAGAGCCTCTTAGGTTCTCAGTGACCCAGAT

AGCTGCTCCAGCCCCCAGGGGGTGATGCACCCCTGAAGAGCAGCTGCCCAACACCGCCAA

CCCCCTGCAAGAACCCCACAGCCTAAAAACACCAGAGCAAGCTCTGCATGACCAAGTGAA

ATCTGCTACCATCGTGGTGTGGACCTCCCAGTCCTGTCTGCCCTCAGGAAGTCCTCCTTT

GCTTCAAAGAAACACTGTTAGCCCCATCAACACTCCAGAAAAGCCACACTGCCTCAAAAA

AGATTGACCAACAACGCCAGCCCTCAGGAAATATTCCACGGCAGTGACAAGGCAAACACT

GCCCGATCACGGAGAGTACAACTCCCTCAGGAGAAAGAAAACAACAAGCAAGATGAAGAA

GCTGAGAAACCACCCCCAGTCAAACCAACAGGAGAACTCACCTAAAACAGTCAACAATGA

AACAGATCTCTGCAGTCAGACAGACCTGGAGTTCAAAAGAGAAATAGTGAAAATACTGAA

GGAATTAAGAGAAGATATGAACAGTAATGCAGATACCCTCAGAAAGGAACTAGAAAATAT

AAGGAGGAGCCAAGAAAAACTAGAACATTCATTTGCAGAGATGCAAACTGAACTAGGGGC

AGTAAAAACCAGAATGAATAATGCAGAAGAACGAATCAGTGATATGGAAGATAGAATAAT

GGAAATCACTCAATCTGGTCAACAGACAGAAAACCGAATCAAAAAACTGGAAAGCAATAT

AAGAGACCTATGGGATAATATAAAGCGGGCCAATCTACGCATAATAGGAATTCCAGAAGG

AGTAGAAAAAGATAAGGGAATGGAAAATATATTTGAAGAAATTATCGCTGGAAACTTCCC

AAATCTAAAGGATACTGGATTCAAGATACAAGAAGCACAGAGGGCCCCAAACAAACTGAA

CCCAAACAGACCCACACCAAGACACATCATAATAAAAATGGCAAAAGTTAGTGATAAAGA

GAGGATCCTAAAGGCAGCAAGAGAAAAACAGAATGTTACCTACAAGGGAACCCCCATAAG

AATATCAGCTGATTTCTCTACAGAAACACTACAGGCCAGGAGGGAATGGCAAGAGATATT

TAAAGTGCTCAAAGGAAAAAATATGCAACCTAGAATACTCTATCCAGCAAGAATATCATT

TAAAATAGAAGGGGAAATAAAAATTTTTCCCAACAAACAAAAACTTAAAGAATACAGCAA

CACAAAACCCAGGTTAAAGGAAATATTGAAAGGGCTTCTCTAAACCAAAAAGAAAGGAAG

GAAAGGGAAGAAAAAAGAAAAGAAAAAAAAAAAGAAGAAGAAGAGGAAGAACTAGGACTG

AGGAAACTGCAATCAGAGAGCAGTCACTCAAATAAGCCAGCATACAGATTTAATCATGAA

CATGCTTCAAACAAAATAAAATTAAAAAGAAAAAAATAAAAAAGAGTCATCAAAACCATA

AAATGTGGGCAAGGGATGTTAGGAGGTAAATAACCCTTTTTGTTTTATGTATGTCTCTCT

TCTTAATTTTAATATAGTAATGAAGTGTTTGAACTTACAGGACCATCAGGCTAAAACACA

CAATTATGGGAAGGGGTTAGCATACTTAAAAAACAGGGCAACCACAAGCCAAAACCAAAT

ATTGCATTTGCAAAAAATGAAAAAAAAAAACACTCAAGCAGATAATAACAGGAGACCATC

CAACCAAAAAAAAAAAAAAAAAAAAGGAAGAATGGAGAACCATAGAATCAACTGGAACAC

GAGGTTCAAATGGCAATAAATAATCATCTATCAATTATCACCTTAAATGTCAATGGACTG

AATGCCCCAATCAAAAGACACAGAGTGGCTGAGTGGATAAAAAGGCAAAAACCTTCAATA

TGCTGCCTACAAGAAACTCACCTTAGGACAAAAGATACATATAGATTGAAAGTGAAAGGG

TGGGGAAAAATATTTCACGCCAATAGACATGACAGAAAAGCAGGAGTCGCAACGCTCATA

TCAGACAAAATAGACTTTAAAACAAAAGACATAAAGAAAGACAAAGAAGGACACTATTTA

ATGATTAAGGGATCCATCCAAGGAGAGGATGTTACTATCGTCAACATATATGCCCCAAAT

ACAGGAGCACCCAGATACATACAACAAATATTAACAGACATAAAGGGAGATATTGATGAG

AATACAATCATAGTAGGAGACCTAAATACCCCCCTCACATCAATGGACAGATCCTCTAGA

CAGAAAACCAATAAAGCAACAGAGATCCTAAAGGAAACAATAGAAAAGTTAGACTTAATT

GATATCTTCAGGACACTACATCCAAAAAAAGCAGAATACACATTCTTCTCAAATGCTCAT

GGAACATTCTCAAGAATCGACCACATATTGGGACACAAAGCGAATCTCAATAAATTTAGG

AGCGTAGAAATTATCTCAAGTATCTTCTCTGACCACAATGCCATGAAATTAGAAATCAAC

CATGGGAAAAGCAAAGAGAAAAAACCTACTACATGGAGACTAAACAACATGCTACTAAAA

AACCAATGGGTCAATGAGGAAATCAAGAAGGAAATTAAAAACTACCTTGAAACAAATGAT

AATGAAGACACAACCTCTCAAAATCTATGGGATGCTGCGAAAGCAGTGCTCAGAGGGAAA

TTTATAGCAATACAGGCCTTTCTCAAAAAAGAAGAAAGATCCCAAATTGACAACTTAACC

CTCCACCTAAACGAATTAGAAAAAGAAGAACAAAAAAGTCCTAAAGTCAGCAGAAGGAAG

GAAATTATAAAGATCAAAGAAGAAATCAATAAAATAGAGACTCAAAAAACAATAGAGAAA

ATTAATAAAACCAAGAGCTGGTTCTTTGAAAAGGTGAACAAAATTGACAAACCCCTGGCC

AGACTCACTAAAAAGAGGAGAGAAAGAACCCAAATAACCAAAATTATAAATGAAAAAGGA

GAAATCACAACGGATACAGCAGAAATACAAAAAACCATAAGAGAATACTATGAACAACTG

TATGGCAACAAGTTTGACAATCTGGAAGAAATGGACAATTTTCTAGAATCTTACAGCCTG

CCAAAACTGAATCAAGCAGAAACAGACCAACTGAACAGACCGATCACTAGAAATGAAATT

GAAGAGGTCATAAAATCACTCCCTACAAATAAAAGTCCAGGACCAGATGGCTTCACAGGT

GAATTCTATCAAACATATAAAGAGGAATTGGTGCCCATCCTCCTTAAACTCTTTCAAAAG

GTTGAAGAAGAAGGAATACTCCCAAAGACATTCTATGAGGCCACCATCACCCTCATTCCA

AAACCAGACAGAGATACCACCAAAAAAGAAAACTATCGCCCAATATCATTGATGAATATA

GATGCAAAAATTCTCAACAAAATCTTAGCCAACCGAATCCAACAACATATCAAAAAAATT

ATACACCATGACCAGGTTGGGTTCATCCCAGGTTCACAAGGATGGTTCAACATACGCAAA

TCAATCAGCATCATACACCACATTAACAAAAAAAAAGTCAAAAATCATATGATCATCTCA

ATAGACGCAGAAAAAGCATTTGACAAAGTTCAACATCCATTCATGATCAAGACCCTCGCC

AAAGTGGGTATAGAGGGAACATTCCTGAATATAATCAAAGCCATTTATGATAAACCCACA

GCAAATATAATCCTCAATGGGGAAAAACTGAAAGCCTTCTCACTCAAATCTGGAACAAGA

CAGGGATGCCCACTCTCACCACTGCTCTTCAACATAGTTTTGGAAGTCCTAGCCACAGCA

ATTAGACAAACAAAAGAAATAAAAGGCATCCATATAGGAAGAGAAGAGATCAAACTGTCA

CTGTATGCAGATGACATGATACTATACCTAGAAAACCCTAAGGACTCAACCCCAAAACTC

CTTGAACTGATTAATAAATTCAGCAAAGTGGCAGGATATAAGATTAACATTCAGAAGTCA

GTTGCATTTCTGTATACCAGCAATGAAACATTAGAAAAGGAATACAAAAATACGATACCT

TTTAAAATTGTACCTCACAAAATCAAATACCTCGGAATACACCTGACCAAAGAGGTAAAG

GACCTATATGCCGAGAACTATAAAACTTTAATCAAAGAAATCAAAGAAGATGTAAAGAAA

TGGAAAGATATTCCATGTTCCTGGATTGGGAAAATCAATATTGTGAAAATGGCCATCCTA

CCCAAAGCAATCTACAGATTCAATGCAATCCCTATCAAATTACCCATGACATTTTTCACA

GAACTAGAACAAACAATCCAAACATTTATATGGAACCACAAAAGACCCAGAATCGCCAAA

GCAATCCTGAGAAACAAAAACCAAGCAGGAGGCATAACTCTCCCAGACTTCAAGAAATAC

TACAAAGCCACAGTCATCAAAACAGTGTGGTACTGGTATCAAAACAGACAGACAGACCAA

TGGAACAGAATAGAGAATCCGGAAATAAACCCTGACACCTATGGTCAATTAATCTTTGAC

AAGGGAGGCAAGAACATCAAATGGGAAAAGGAAAGTCTATTCAGCAAGCATTGCTGGGAA

ACCTGGACAGCTGCATGCAAAGCAATGAAACTAGAACACACCCTCACACCATGCACAAAA

ATAAACTCCAAATGGCTGAAAGACTTAAATATACGACAGGACACCATCAAACTCCTAGAA

GAAAACATAGGCAAAACACTCTCTGACATCAACATCATGAATATTTTCTCAGGTCAGTCT

CCCAAAGCAATAGAAATTAGAGCAAAAATAAACCCATGGGACCTCATCAAACTGAAAAGC

TTTTGCACAGCAAAGGAAACCCAAAAGAAAACAAAAAGACAACTTACAGAATGGGAGAAA

ATAGTTTCAAATGATGCAACTGACAAGGGCTTAATCTCTAGAATATATAAGCAACTTATA

CAACCCAACAGCAAAAAAACCAATCAATCAATGGAAAAATGGGCAAAAGACCTGAATAGA

CATTTCTCCAAAGAAGATATACAGATGGCCAACAAACACATGAAAAAATGCTCAACATCG

CTGATTATAAGAGAAATGCAAATCAAAACTACCATGAGATACCACCTCACACCAGTCAGA

ATGGCCATCATTAATAAATCCACAAATAACAAGTGCTGGAGGGGCTGTGGAGAAAAGGGA

ACCCTCCTGCACTGCTGGTGGGAATGTAAACTGGTACAGCCACTATGGAGAACAGTTTGG

AGATACCTTAGAAATCTATACATAGAACTTCCATATGACCCTGCAATCCCACTCTTGGGC

ATCTATCCGGACAAAGCTCTACTTAAAAGAGACACATGCACCCGCATGTTCATTGCAGCA

CTATTCACAATAGCCAGGACATGGAAACAACCCAAATGTCCATCGACAGATGATTGGATT

CGGAAGAGTGGTATATATACACAATGGAATACTACTCAGCCATAAAAAAGGATGACATAA

TGCCATTTGCAGCAACATGGATGGAACTAGAGAATCTCATCCTGAGTGAAATGAGCCAGA

AAGACAAAGACAAATACCATATGATATCACTTATAACTGGAATCTAATATCCAGCACAAA

TGAACATCTCCTCAGAAAAGAAAATCATGGACTTGGAGAAGAGACTTGTGGTTGCCTGAT

GGGAGGGGGAGGGAGTGGGAGGGATCGGGAGCTTGGGCTTATCAGACACAACTAGAATAG

ATTTACAAGGAGATCCTGCTGAATAGCATTGAGAACTATGTCTAGATACTCATGTTGCAA

CAGAAGAAAGGGTGGGGGAAAAACTGTAATTGTAATGTATACATGTAAGGATAACCTGAC

CCCCTTGCTGTACAGTGGGAAAATAAAAAAAAAAAAAAAAAAAAAA

>L1D15#LINE/L1D

GGAGAGAGGACAAGATGGCGGAGGAGTAGGGGGACACGCTCGCCCTCTCCCACAAACACA

ACAAAAAAAGCACATCTACAGAATAAATGACTCGCACAGAACAGCAACCAATCGCTGGCA

GAGGAACCTAAACTCCAATAACGGCAAGAAGTTCGTGACATTATTGGGCAGAACGGGAGA

AAAGAGGAGAGTGAGAGAAGGTGAATCCGAGCGGGACGGGCGCTCCCGAAAGGGAACTGC

GGAGGAGAAAGGGATCCCGCACCCTGGAAAGTCACCTACCGGGGGAAAGATCAAACGAAC

CGGAGGAATCTCCAGATGCAGAGAAGAGTGTAGCAGTAAGTTGGAGTACGGAAAAGCCGA

TCAAGAACCCAACGGACCATCTGAACTACGGGCACAGTCACCAAAAATTGAGACGCCTGG

GTGGGGGCTGGGCACCGAGACCTCGCTCCAGGTTAGTCCCCGAGAAAGGGCCGGGGGACG

CCTGGGTGGGGGCTGGGCACCGAGACCTCGGCTCCAGAGGTTAGTCCCCGGGCTGGGGGG

GCGGGGCAGAGCGGAAACTGCTTGGGAGGTCTAGAAACCATTTGACGGGGCAGAGACTGC

CTGGGAGACTAGAAAACAAAGCTGTCGCAGAGGAAGGGAACAATACTCTAGGGGCGGGGA

AGTGGAAAGCCGCATCAGAGGGAACCTGGGAGAAGAGCCTGGTCTGCGCCCGTGCTGGGG

AGGGGAGAGAAGAAGGGGTGGGTCCCCATAGAATACCCCCCACGCCACAGCAAGCTTACA

GGCCCGCTAGCTAGCAGAAAGCTGTGCTTCCCAGTGCATCCCCTCCCCCCACCCCCGCCA

CCCCCTACGCTCTCACCGGACCTGGGGCTGCCTGCCATCCAGGAGGGCTGGCCTCAACAA

TTGCCTGAAGCCTACCACCGCAGGGGCTGTCCCTGCACAGGCCTGCTTGCCCTTTGGAGG

GGCTACACTTCCGCAGAGCAGCACCAAACACCACCAGCCCCCGAGAAAAGGCCTGCAGCC

CAGAAAAGCTAGAACAAGCCTAGCCAGGCCGTGAATAGATCGGCCTAATTCTCGGACGGT

TTTTCTGAGTCGGGCTGCCCCGGGGAGGAGCCTCTTGGGTTTCCAACGGCCCTGCTACCC

GCCCAAGCCCCCAGGGGGTGCTGAGCCTAGTGGACCAGCTGCATAGGACTGCCAGCTCCA

GGCAGGACCCCCTGCAGCCCAGAAAAGCTGCAACAAGCCTGGCCGAGTGGGAAAAGATCT

CAGAGGTCTTTCTGAGTCGGGCTGCCCTGGGGAGGAGCCTCTTGGGTTCCAGGCCCTGCT

ACCGCCCAAGCCCCCAGGGGGTGCCCCACTCCCGCGGAATAGCTGCTCAGCACCACCAGC

CCCCTGGAAGAGCCCCTGCAGCCCAGAAAAGCTGCAACAAGCTTGGCCAGACTGTGAAAA

GATCCGCCTACATTCTCAGGCCGTCCTTCTGAGTTGGGCTGCCCTAGGGAAGAGCCTCTT

AGGTTCTCAGTGACCCAGATAGCTGCTCCAGCCCCCAGGGGGTGCTGCACTCCTGAGGAG

CAGCTGCCCAACACCGCCAACCCCCTGCAAGAACCCCACAGCCTAAAAACACCAGAGCAA

GCTCTGCATGACCAAGTGAAATCTGCTACCATCGTGGTGTGGACCTCCCAGTCCTGTCTG

CCCTCAGGAAGTCCTCCTTTGCTTCAAAGAAACACTGTTAGCCCCATCAACACTCCAGAA

AAGCCACACTGCCTCAAAAAAGATTGACCAACAACGCCAGCCCTCAGGAAATATTCCACG

GCAGTGACAAGGCAAACACTGCCCGATACGGAGAGTACAACTCCCTCAGGAGAAAGAAAA

CAACAAGCAAGATGAAGAAGCTGAGAAACCACCCCCAGTCAAACCAACAGGAGAACTCAC

CTAAAACAGTCAACAATGAAACAGATCTCTGCAGTCTGACAGACCTGGAGTTCAAAAGAG

AAATAGTGAAAATACTGAAGGAATTAAGAGAAGATATGAACAGTAATGCAGATACCCTCA

GAAAGGAACTAGAAAATATAAGGAGGAGCCAAGAAAAACTAGAACATTCATTTGCAGAGA

TGCAAACTGAACTAGGGGCAGTAAAAACCAGAATGAATAATGCAGAAGAACGAATCAGTG

ATATGGAAGATAGAATAATGGAAATCACTCAATCCGGTCAACAGACAGAAAACCGAATCA

AAAAACTGGAAAGCAATATAAGAGACCTATGGGATAATATAAAGCGGGCCAATCTACGCA

TAATAGGAATTCCAGAAGGAGTAGAAAAAGATAAGGGAATGGAAAATATATTTGAAGAAA

TTATCGCTGGAAACTTCCCAAATCTAAAGGATACTGGATTCAAGATACAAGAAGCACAGA

GGGCCCCAAACAAACTGAACCCAAACAGACCCACACCAAGACACATCATAATAAAAATGG

CAAAAGTTAGTGATAAAGAGAGGATCCTAAAGGCAGCAAGAGAAAAACAGAATGTTACCT

ACAAGGGAACCCCCATAAGAATATCAGCTGATTTCTCTACAGAAACACTACAGGCCAGGA

GGGAATGGCAAGAGATATTTAAAGTGCTCAAAGGAAAAAATATGCAACCTAGAATACTCT

ATCCAGCAAGAATATCATTTAAAATAGAAGGGGAAATAAAAATTTTTCCCAACAAACAAA

AACTTAAAGAATACAGCAACACAAAACCCAGGTTAAAGGAAATATTGAAAGGGCTTCTCT

AAACCAAAAAGAAAGGAAGGAAAGGGAAGAAAAAAGAAAAGAAAAAAAAAAAAAGAAGAA

GAGGAAGAACTAGGACTGAGGAAACCGCAATCAGAGAGCAGTCACTCAAATAAGCCAGCA

TACAGATTTAATCATGAACATGCTTCAAACAAAATAAAATTAAAAAGAAAAAAATAAAAA

AGAGTCATCAAAACCATAAAATGTGGGCAAGGGATGTTAGGAGTAAATAACCCTTTTTGT

TTGTTGTATGTCTCTCTTCTTAATTTTAATATAGTAATGAAGTGTTTGAACTTACAGGAC

CATCAGGCTAAAACACACAATTATGGGAAGGGGTTAGCATACTTAAAAAACAGGGCAACC

ACAAGCCAAAACCAAATATTGCATTTGCAAAAAATAAAAAAAAATACACTCAAGCAGATA

ATAACAGGAGACCATCCAACCAAAAAAAAAAAAAAAAAAGAAAGGAAGAATGGAGAACCA

TAGAATCAACTGGAACACGAGGTTCAAATGGCAATAAATAATCATCTATCAATTATCACC

TTAAATGTCAATGGACTGAATGCCCCAATCAAAAGACACAGAGTGGCTGAGTGGATAAAA

AGGCAAAAACCTTCAATATGCTGCCTACAAGAAACTCACCTTAGGACAAAAGATACATAT

AGATTGAAAGTGAAAGGGTGGGGAAAAATATTTCACGCCAATAGACATGACAGAAAAGCA

GGAGTCGCAACGCTCATATCAGACAAAATAGACTTTAAAACAAAAGACATAAAGAAAGAC

AAAGAAGGACACTACTTAATGATTAAGGGATCCATCCAAGGAGAGGATGTTACTATCGTC

AACATATATGCCCCAAATATAGGAGCACCCAGATACATACAACAAATATTAACAGACATA

AAGGGAGATATTGATGAGAATACAATCATAGTAGGAGACCTTAATACCCCCCTCACATCA

ATGGACAGATCCTCTAGACAGAAAACCAATAAAGCAACAGAGATCCTAAAGGAAACAATA

GAAAAGTTAGACTTAATTGATATCTTCAGGACACTACATCCAAAAAAAGCAGAATACACA

TTCTTCTCAAATGCTCATGGAACATTCTCAAGAATCGACCACATATTGGGACACAAAGCG

AATCTCAATAAATTTAGGAGCATAGAAATTATCTCAAGTATCTTCTCTGACCACAATGCC

ATGAAATTAGAAATCAACCATGGGAAAAGGAAAGAGAAAAAACCTACTCCATGGAGACTA

AACAACATGCTACTAAAAAACCAATGGGTCAATGAGGAAATCAAGAAGGAAATTAAAAAC

TACCTTGAAACAAATGATAATGAAGACACAACCTCTCAAAATCTATGGGATGCTGCGAAA

GCAGTGCTCAGAGGGAAATTTATAGCAATCCAGGCCTTTCTCAAAAAAGAAGAAAGATCC

CAAATTGACAACTTAACCCTCCACCTAAACGAATTAGAAAAAGAAGAACAAAAAAGTCCT

AAAGTCAGCAGAAGGAAGGAAATTATAAAGATCAAAGAAGAAATCAATAAAATAGAGACT

CAAAAAACAATAGAGAAAATTAATAAAACCAAGAGCTGGTTCTTTGAAAAGGTGAACAAA

ATTGACAAACCCCTGGCCAGACTCACTAAAAAGAGGAGAGAAAGAACCCAAATAACCAAA

ATTATAAATGAAAAAGGAGAAATCACAACGGATACAGCAGAAATACAAAAAACCATAAGA

GAATACTATGAACAACTATATGGCAACAAGTTTGACAATCTGGAAGAAATGGACAATTTT

CTAGAATCTTACAGCCTGCCAAAACTGAATCAAGCAGAAACAGACCAACTGAACAGACCG

ATCACTAGAAATGAAATTGAAGAGGTCATAAAATCACTCCCTACAAATAAAAGTCCAGGA

CCAGATGGCTTCACAGGTGAATTCTATCAAACATATAAAGAGGAATTGGTGCCCATCCTC

CTTAAACTCTTTCAAAAGGTTGAAGAAGAAGGAATACTCCCAAAGACATTCTATGAGGCC

ACCATCACCCTCATTCCAAAACCAGACAGAGATACCACCAAAAAAGAAAACTATCGCCCA

ATATCATTGATGAATATAGATGCAAAAATTCTCAACAAAATCTTAGCCAACCGAATCCAA

CAACATACCAAAAAAATTATACACCATGACCAGGTTGGGTTCATCCCAGGTTCACAAGGA

TGGTTCAACATACGCAAATCAATCAGCATCATACACCACATTAACAAAAAAAAAAGTCAA

AAATCATATGATCATCTCAATAGACGCAGAAAAAGCATTTGACAAAGTTCAACATCCATT

CATGATCAAGACCCTCGCCAAAGTGGGTATAGAGGGAACATTCCTGAAATAATCAAAGCC

ATTTATGATAAACCCACAGCAAATATAATCCTCAATGGGGAAAAACTGAAAGCCTTCTCA

CTCAAATCTGGAACAAGACAGGGATGCCCACTCTCACCACTGCTCTTCAACATCGTTTTG

GAAGTCTTAGCCACAGCAATTAGACAAACAAAAGAAATAAAAGGCATCCATATAGGAAGA

GAAGAGATAAACTGTCACTGTATGCAGATGACATGATACTATACCTAGAAAACCCTAAGG

ACTCAACCCCAAAACTCCTTGAACTGATTAATAAATTCAGCAAAGTGGCAGGATATAAGA

TTAACATTCAGAAGTCAGTTGCATTTCTGTATACCAGCAATGAAACATTAGAAAAGGAAT

ACAAAAATACGATACCTTTTAAAATTGACCTCACAAAATCAAATACCTCGGAATACACCT

GACCAAGGAGGTAAAGGACCTATATGCCGAGAACTATAAAACTTTAATCAAAGAAATCAA

AGAAGATGTAAAGAAATGGAAAGATATTCCATGTTCCTGGATTGGAAAATCAATATTGTA

AAAATGGCCATACTACCCAAAGCAATCTACAGATTCAATGCAATCCCTATCAAATTACCC

ATGACATTTTTCACAGAACTAGAACAAACAATCCAAACATTTATATGGAACACAAAAGAC

CCAGAATCGCCAAAGCAATCCTGAGAAACAAAAACCAAGCAGGAGGCATAACTCTCCCAG

ACTTCAAGAAATACTACAAAGCCACAGTCATCAAAACAGTGTGGTACTGGTACAAAACAG

ACAGACAGACCAATGGAACAGAATAGAGAATCCGGAAATAAACCCTGACACCTATGGTCA

ATTAATCTTTGACAAGGGAGGCAAGAACATAAAATGGGAAAAGGAAAGTCTATTCAGCAA

GCATTGCTGGGAAACCTGGACAGCTGCATGCAAAGCAATGAAACTAGAACACACCCTCAC

ACCATGCACAAAAATAAACTCCAAATGGCTGAAAGACTTAAATATAGACAGGACACCATC

AAACTCCTAGAAGAAAACATAGGCAAAACACTCTCTGACATCAACATCATGAATATTTTC

TCAGGTCAGTCTCCCAAAGCAATAGAAATTAGAGCAAAAATAAACCCATGGGACCTCATC

AAACTGAAAAGCTTTTGCACAGCAAAGGAAACCCAAAAGAAAACAAAAAGACAACTTACA

GAATGGGAGAAAATAGTTTCAAATGATGCAACTGACAAGGGCTTAATCTCTAGAATATAT

AACAACTTATACAACCCAACAGCAAAAAAACCAATCAATCAATGGAAAAATGGGCAAAAG

ACCTGAATAGACATTTCTCCAAAGAAGATATACAGATGGCCAACAAACACATGAAAAAAT

GCTCAACATCGCTGATTATAAGAGAAATGCAAATCAAAACTACCATGAGATACCACCTCA

CACCAGTCAGAATGGCCATCATTAATAAATCCACAAATAACAAGTGCTGGAGGGGCTGTG

GAGAAAAGGGAACCCTCCTGCACTGTTGGTGGGAATGTAAACTGGTACAGCCACTATGGA

GAACAGTTTGGAGATACCTTAGAAATCTATACATAGAACTTCCATATGACCCCGCAATCC

CACTCTTGGGCATCTATCCGGACAAACTCTACTTAAAAGAGACACATGCACCCGCATGTT

CATTGCAGCACTATTCACAATAGCCAGGACATGGAAACAACCCAAATGTCCATCGACAGA

TGATTGGATTCGGAAGAGGTGGTATATATACACAATGGAATACTACTCAGCCATAAAAAA

GAATGACATAATGCCATTTGCAGCAACATGGATGGAACTAGAGAATCTCATCCTGAGTGA

AATGAGCCAGAAAGACAAAGACAAATACCATATGATATCACTTATAACTGGAATCTAATA

TCCAGCACAAATGAACATCTCCTCAGAAAAGAAAATCATGGACTTGGAGAAGAGACTTGT

GGCTGCCTGATGGGAGGGGGAGGGAGTGGGAGGGATCGGGAGCTTGGGCTTATCAGACAC

AACTTAGAATAGATTTACAAGGAGATCCTGCTGAATAGCATTGAGAACTATGTCTAGATA

CTCATGTTGCAACAGAAGAAAGGGTGGGGGAAAAATGTAATTGTAATGTATACATGTAAG

GATAACCTGACCCCCTTGCTGTACAGTGGGAAAATAAAAAAAAAAAAAAAAAAAAAAAA

>L1D16#LINE/L1D

GAGAGAGGACAAGATGGCGGAGGAGTAGGGGGACACGCTCGCCCTCTCCCACAAACACAA

CAAAAAAAGCACATCTACAGAATAAATGACTCGCACAGAACAGCAACCAATCGCTGGCAG

AGGAACCTAAACTCCAATAACGGCAAGAAGTTCGTGACATTACTGGGCAGAACGGGAGAA

AAGAGGAGAGTGAGAGAAGGTGAATCCGAGCGGGACGGGCGCTCCCGAAAGGGAACTGCG

GAGGAGAAAGGGATCCCGCACCCTGGAAAGTCACCTACCGGGGGAAAGATCAAACGAACC

GGAGGAATCTCCAGATGCAGAGAAGAGTGTAGCAGTAAGTTGGAGTACGGAAAAGCCGAT

CAAGAACCCAACGGACCATCTGAACTACGGGCACAGTCACCAAAAATTGAGACGCCTGGG

TGGGGGCTGGGCACCGAGCCTCGCTCCAGAGGTTAGTCCCCGGGAAAGGGCCGGGGGACG

CCTGGGTGGGGGCTGGGGCACCGAGACCTCGGCTCCAGAGTTAGTCCCCGGGGGGGGGCG

GGGCAGAGCGGAAACTGCTTGGGAGGTCTAGAAACCATTTGACGGGGCAGAGACTGCCTG

GGAGACTAGAAAACAAAGCTGTCGCAGAGGAAGGGAGCAATACTCTAGGGGCGGGGAAGT

GGAAAGCCGCATCAGAGGGAACCTGGGAGAAGAGCCTGGTCTGCGCCCGTGCTGGGGAGG

GGAGAGAAGAAGGGGTGGGTCCCCATAGAATACCCCCCACGCCACAGCAAGCTTACAGGC

CCGCTAGCTAGCTGAAAGCTGTGCTTCCCAGTGCATCCCCTCCCCCCACCCCCGCCACCC

CCTACGCTCTCACCGGACCTGGGGCTGCCTGCCATCCAGGAGGGCTGGCCTCAACAATTG

CCTGAAGCCTACCACCGCAGGGGCTGTCCCTGCACAGGCCTGCTTGCCCTTTGGAGGGGC

TACACTTCCGCAGAGCAGCACCAAACACCACCAGCCCCCGAGAAAAGGCCTGCAGCCCAG

AAAAGCTAGAACAAGCCTAGCCAGGCTGTGAATAGATCGGCCTAATTCTCGAGTTTTTCT

GAGTCGGGCTGCCCCGGGGAGGAGCCTCTTGGGTTTCCAACGGCCCTGCTACCCGCCCAA

GCCCCCAGGGGGTGCTCTAGTGGACCAGCTGCATAGGACTGCCAGCTCCAGGCAGGACCC

CCTGCAGCCCAGAAAAGCTGCAACAAGCCTGGCCGAGTCGGGAAAAGATCTCAGACGGTT

TTTCTGAGTCGGGCTGCCCTGGGGAGGAGCCTCTTGGGTCTCCAAGGCCCTGCTACCCGC

CCAAGCCCCCAGGGGGTGCCCCACTCCCGTGGAATAGCTGCTCAGCACCACCAGCCCCCT

GGAAGAGCCCCTGCAGCCCAGAAAAGCTGCAACAAGCTCGGCCAGACTGTGAAAAGATCC

GCCTACATTCTCAGGCTGTCCTTCTGAGTTGGGCTGCCCTAGGGAAGAGCCTCTTAGGTT

CTCAGTGACCCAGATAGCTGCTCCAGCCCCCAGGGGGTGCTGCACTCCTGAGGAGCAGCT

GCCCAACACCGCCAACCCCCTGCAAGAACCCCACAGCCTAAAAACACCAGAGCAAGCTCT

GCATGACCAAGTGAAATCTGCTACCATCGTGGTGTGGACCTCCCAGTCCTGTCTGCCCTC

AGGAAGTCCTCCTTTGCTTCAAAGAAACACTGTTAGCCCCATCAACACTCCAGAAAAGCC

ACACTGCCTCAAAAAAGATTGACCAACAACGCCAGCCCTCAGGAAATATTCCACGGCAGT

GACAAGGCAAACACTGCCCGATAACGGAGAGTACAACTCCCTCAGGAGAAAGAAAACAAC

AAGCAAGATGAAGAAGCTGAGAAACCACCCCCAGTCAAACCAACAGGAGAACTCACCTAA

AACAGTCAACAATGAAACAGATCTCTGCAGTCAGACAGACCTGGAGTTCAAAAGAGAAAT

AGTGAAAATACTGAAGGAATTAAGAGAAGATATGAACAGTAATGCAGATACCCTCAGAAA

GGAACTAGAAAATATAAGGAGGAGCCAAGAAAAACTAGAACATTCATTTGCAGAGATGCA

AACTGAACTAGGGGCAGTAAAAACCAGAATGAATAATGCAGAAGAACGAATCAGTGATAT

GGAAGATAGAATAATGGAAATCACTCAATCGGTCAACAGACAGAAAACCGAATCAAAAAA

CTGGAAAGCAATATAAGAGACCTATGGGATAATATAAAGCGGGCCAATCTACGCATAATA

GGAATTCCAGAAGGAGTAGAAAAAGATAAGGGGATGGAAAATATATTTGAAGAAATTATC

GCTGGAAACTTCCCAAATCTAAAGGATACTGGGTTCAAGATACAAGAAGCACAGAGGGCC

CCAAACAAACTGAACCCAAACAGACCCACACCAAGACACATCATAATAAAAATGGCAAAA

GTTAGTGATAAAGAGAGGATCCTAAAGGCAGCAAGAGAAAAACAGAATGTTACCTACAAG

GGAACCCCCATAAGAATATCAGCTGATTTCTCTACAGAAACACTACAGGCCAGGAGGGAA

TGGCAAGAGATATTTAAAGTGCTAAAAGGAAAAAATATGCAACCTAGAATACTCTATCCA

GCAAGAATATCATTTAAAATAGAAGGGGAAATAAAAATTTTTCCCAACAAACAAAAACTT

AAAGAATACAGCAACACAAAACCCAGGTTAAAGGAAATATTGAAAGGGCTTCTCTAAACC

AAAAAGAAAGGAAGGAAAGGGAAGAAAAAAGAAAAGAAAAAAAAAAAAAAAGAAGAAGAG

GAAGAACTAGGACTGAGGAAACCGCAATCAGAGAGCAGTCACTCAAATAAGCCAGCATAC

AGATTTAATCATGAACATGCTTCAAACAAAATAAAATTAAAAAGAAAAAAATAAAAAAGA

GTCATCAAAACCATAAAATGTGGGCAAGGGATGTTAGGAAGTAAATAACCCTTTTTGTTT

GTTTGTATGTTTCTCTTCTTAATTTTAATATAGTAATGAAGTGTTTGAACTTACAGGACC

ATCAGGCTAAAACACACAATTATGGGAAGGGGTTAGCATACTTAAAAAACAGGGCAACCA

CAAGCCAAAACCAAATATTGCATTTGCAAAAAATGAAAAAAAAAAATACACTCAAGCAGA

TAATAACAGGAGACCATCCAACCAAAAAAAAAAAAAAAAAGAAAGGAAGAATGGAGAACC

ATAGAATCAACTGGAACACGAGGTTCAAATGGCAATAAATAATCATCTATCAATTATCAC

CTTAAATGTCAATGGACTGAATGCCCCAATCAAAAGACACAGAGTGGCTGAGTGGATAAA

AAGGCAAAAACCTTCAATATGCTGCCTACAAGAAACTCACCTTAGGACAAAAGATACATA

TAGATTGAAAGTGAAAGGGTGGGGAAAAATATTTCACGCCAATAGACATGACAGAAAAGC

AGGAGTCGCAACGCTCATATCAGACAAAATAGACTTTAAAACAAAAGACATAAAGAAAGA

CAAAGAAGGACACTATTTAATGATTAAGGGATCCATCCAAGGAGAGGATGTTACTATCAT

CAACATATATGCCCCAAATATAGGAGCACCCAGATACATACAACAAATATTAACAGACAT

AAAGGGAGATATTGATGAGAATACAATCATAGTAGGAGACCTAAATACCCCCCTCACATC

AATGGACAGATCCTCTAGACAGAAAACCAATAAAGCAACAGAGATCCTAAAGGAAACAAT

AGAAAAGTTAGACTTAATTGATATCTTCAGGACACTACATCCAAAAAAACAGAATACACA

TTCTTCTCAAATGCTCATGGAACATTCTCAAGAATCGACCACATATTGGGACACAAAGCG

AATCTCAATAAATTTAGGAGCATAGAAATTATCTCAAGTATCTTCTCTGACCACAATGCC

ATGAAATTAGAAATCAACCATGGGAAAAGAAAGAGAAAAAACCTACTCCATGGAGACTAA

ACAACATGCTACTAAAAAACCAATGGGTCAATGAGGAAATCAAGAAGGAAATTAAAAACT

ACCTTGAAACAAATGATAATGAAGACACAACCTCTCAAAATCTATGGGATGCTGCGAAAG

CAGTGCTCAGAGGGAAATTTATAGCAATACAGGCCTTTCTCAAAAAAGAAGAAAGATCCC

AAATTGACAACTTAACCCTCCACCTAAATGAATTAGAAAAAGAAGAACAAAAAAGTCCTA

AAGTCAGCAGAAGGAAGGAAATTATAAAGATCAAAGAAGAAATCAATAAAATAGAGACTC

AAAAAACAATAGAGAAAATTAATAAAACCAAGAGCTGGTTCTTTGAAAAGGTGAACAAAA

TTGACAAACCCCTGGCCAGACTCACTAAAAAGAGGAGAGAAAGAACCCAAATAACCAAAA

TTATAAATGAAAAAGGAGAAATCACAACGGATACAGCAGAAATACAAAAAACCATAAGAG

AATACTATGAACAACTTATGGCAACAAGTTTGACAATCTGGAAGAAATGGACAATTTTCT

AGAATCTTACAGCCTGCCAAAACTGAATCAAGCAGAAACAGACCAACTGAACAGACCGAT

CACTAGAAATGAAATTGAAGAGGTCATAAAATCACTCCCTACAAATAAAAGTCCAGGACC

AGATGGCTTCACAGGTGAATTCTATCAAACATATAAAGAGGAATTGGTGCCCATCCTCCT

TAAACTCTTTCAAAAGGTTGAAGAAGAAGGAATACTCCCAAAGACATTCTATGAGGCCAC

CATCACCCTCATTCCAAAACCAGACAGAGATACCACCAAAAAAGAAAACTATCGCCCAAT

ATCATTGATGAATATAGATGCAAAAATTCTCAACAAAATCTTAGCCAACCGAATCCAACA

ACATACCAAAAAAATTATACACCATGACCAGGTTGGGTTCATCCCAGGTTCACAAGGATG

GTTCAACATACGCAAATCAATCAGCATCATACACCACATTAACAAAAAAAAAGTCAAAAA

TCATATGATCATCTCAATAGACGCAGAAAAAGCATTTGACAAAGTTCAACATCCATTCAT

GATCAAGACCCTCGCCAAAGTGGGTATAGAGGGAACATTCCTGAATATAATCAAAGCCAT

TTATGATAAACCCACAGCAAATATAATCCTCAATGGGGAAAAACTGAAAGCCTTCTCACT

CAAATCTGGAACAAGACAGGGATGCCCACTCTCACCACTGCTCTTCAACATAGTTTTGGA

AGTCCTAGCCACAGCAATTAGACAAACAAAAGAAATAAAAGGCATCCATATAGGAAGAGA

AGAGATCAAACTGTCACTGTATGCAGATGACATGATACTATACCTAGAAAACCCTAAGGA

CTCAACCCCAAAACTCCTTGAACTGATTAATAAATTCAGCAAAGTGGCAGGATATAAGAT

TAACATTCAGAAGTCAGTTGCATTTCTGTATACCAGCAATGAAACATTAGAAAAGGAATA

CAAAAATACGATACCTTTTAAAATTGTACCTCACAAAATCAAATACCTCGGAATACACCT

GACCAAGGAGGTAAAGGACCTATATGCCGAGAACTATAAAACTTTAATCAAAGAAATCAA

AGAAGATGTAAAGAAATGGAAAGATATTCCATGTTCCTGGATTGGGAAAATCAATATTGT

AAAAATGGCCATACTACCCAAAGCAATCTACAGATTCAATGCAATCCCTATCAAATTACC

CATGACATTTTTCACAGAACTAGAACAAACAATCCAAACATTTATATGGAACCACAAAAG

ACCCAGAATCGCCAAAGCAATCCTGAGAAACAAAAACCAAGCAGGAGGCATAACTCTCCC

AGACTTCAAGAAATACTACAAAGCCACAGTCATCAAAACAGTGTGGTACTGGTATCAAAA

CAGACAGACAGACCAATGGAACAGAATAGAGAATCCGGAAATAAACCCTGACACCTATGG

TCAATTAATCTTTGACAAGGGAGGCAAGAACATAAAATGGGAAAAAGAAAGTCTATTCAG

CAAGCATTGCTGGGAAACCTGGACAGCTGCATGCAAAGCAATGAAACTAGAACACACCCT

CACACCATGCACAAAAATAAACTCCAAATGGCTGAAAGACTTAAATATACGACAGGACAC

CATCAAACTCCTAGAAGAAAACATAGGCAAAACACTCTCTGACATCAACATCATGAATAT

TTTCTCAGGTCAGTCTCCCAAAGCAATAGAAATTAGAGCAAAAATAAACCCATGGGACCT

CATCAAACTGAAAAGCTTTTGCACAGCAAAGGAAACCCAAAAGAAAACAAAAAGACAACT

TACAGAATGGGAGAAAATAGTTTCAAATGATGCAACTGACAAGGGCTTAATCTCTAGAAT

ATATAAGCAACTTATACAACCCAACAGCAAAAAAACCAATCAATCAATGGAAAAATGGGC

AAAAGACCTGAATAGACATTTCTCCAAAGAAGATATACAGATGGCCAACAAACACATGAA

AAAATGCTCAACATCGCTGATTATAAGAGAAATGCAAATCAAAACTACCATGAGATACCA

CCTCACACCAGTCAGAATGGCCATCATTAATAAATCCACAAATAACAAGTGCTGGAGGGG

CTGTGGAGAAAAGGGAACCCTCCTGCACTGCTGGTGGGAATGTAAACTGGTACAGCCACT

ATGGAGAACAGTTTGGAGATACCTTAGAAATCTATACATAGAACTTCCATATGACCCGCA

ATCCCACTCTTGGGCATCTATCCGGACAAAACTCTACTTAAAAGAGACACATGCACCCGC

ATGTTCATTGCAGCACTATTCACAATAGCCAGGACATGGAAACAACCCAAATGTCCATCG

ACAGATGATTGGATTCGGAAGAGGTGGTATATATACACAATGGAATACTACTCAGCCATA

AAAAAGGATGACATAATGCCATTTGCAGCAACATGGATGGAACTAGAGAATCTCATCTGA

GTGAAATGAGCCAGAAAGACAAAGACAAATACCATATGATATCACTTATAACTGGAATCT

AATATCCAGCACAAATGAACATCTCCTCAGAAAAGAAAATCATGGACTTGGAGAAGAGAC

TTGTGGCTGCCTGATGGGAGGGGGAGGGAGTGGGAGGGATCGGGAGCTTGGGCTTATCAG

ACACAACTTAGAATAGATTTACAAGGAGATCCTGCTGAATAGCATTGAGAACTATGTCTA

GATACTCATGTTGCAACAGAAGAAAGGGTGGGGGAAAAATGTAATTGTAATGTATACATG

TAAGGATAACCTGACCCCCTTGCTGTACAGTGGGAAAATAAAAAAAAAAAAAAAAAAAAA

>L1D17#LINE/L1D

GAGAGAGGACAAGATGGCGGAGGAGTAGGGGGACACGCTCGCCCTCTCCCACAAACACAA

CAAAAAAAGCACATCTACAGAAGAAATGACTCGCACAGAACAGCAACCAATCGCTGGCAG

AGGAACCTAAACTCCAATAACGGCAAGAAGTTCGTGACATTATTGGGCAGAACGGGAGAA

AAGAGGAGAGTGAGAGAAGGTGAATCCCAGCGGGACGGGCGCTCCCGAAAGGGAACTGCG

GAGGAGAAAGGGATCCCGCACCCTGGAAAGTCACCTACCGGGCGAAAGATCAAATGAACC

GGAGGAATCCCCAGATGCAGAGAAGAGTGTAGCAGTAAGTCGGAGTACGGAAAAACCGAT

CAAGAACCCAACGGACCATCTGAACTACGGGCACAGTCACCAAAAATTGAGACGCCTGGG

TGGGGGCTGGGCACCGAATCCTCGGCTCCAGAGGTTAGTCCCCGGGAAAGGGCCGGGGGA

CGCCTGGGTGGGGGCTGGGCACCGAGACCTCGCCTCTGAAGGTTAGTCCCCGAGAGGGGG

CCGGGGGACGCCTGGGTGGGGGCTGGGCACCGAGACCTCGGCTCCAGAGATTAGTCCCTG

GGCTAGGGGGGCGGGGCAGAGCGGAAACTGCTTGGGAGGTCTCGAAACCATTTGACGGGG

CAGAGACTGCCTGGGAGACTAGAAAACAAAGCTGTCGCAGAGGAAGGGAGCAATACTCTA

GGGGCGGGGAAGTGGAAAGCCGCCTCAGAGGGAACCTGGGAGAAGAGCCTGGTCTGCGCC

CGTGCTGGGGAGGGGAGAGAAGAAGGGGTGGGTCCCCATAGAATACCCCCCACGCCACAG

CAAGCTTACAGGCCCGCTAGCTAGCAGAAAGCTGTGCTTCCCAGTGCATTCCCTCCCCCC

ACCCCCGCCACCCCCTACGCTCTCGCGAACCTGGGGCTGCCTGCCATCCAGGAGGGCTGG

CCTCAACAATTGCCTGAAGCCTACCACCGCAGGGGCTCTCCCTGCACAGGCCTGCTTGCC

CTTTGGAGGGGCTACACTTCCACAGAGCAGCACCAAACACCACCAGCCCCCTAGAAAAGG

CCTGCAGCCCAGAAAAGCTAGAACAAGCCTAGCCAGGCCGTGAATAGATCGGCCTAATTC

TCCGACGGTTTTTCTGAGACGGGCTGCCCCGGGGAGGAGCCTCTTGGGTCTCCAAGGGCC

CTGCTACCCGCCCAAGCCCCCAGGGGGTGCTAAGACCTAGTGGACCAGCTGCATAGGACT

GCCAGCTCCAGGCAGGACCCCCTGCAGCCCAGAAAAGCTGCAACAAGCCTGGCCGAGTTG

GGAAAAGATCTCAGACGGTCTTTCTGAGTCGGGCTGCCCTGGGGAGGAGCCTCTTGGGTC

TCCAAGGGCCCTGCTACCCGCCCAAGCCCCCAGGGGGTGCACCATGAACTGCAGACCAGC

CCGAGACCCTGCAGCCCAGAAAAGCTGCAACAAGCGGCCGAAAAGATCTTGACGGGGACC

CCCACCAAACCCCAGGGGGTGCTGAGAACCAAGTGAAATCTGCTACCATCGTGGGGTGGA

CCTCCCAGTCCTGTCTGCCCTCAGGAAGTCCTCCTTTGCTTCAAAGAAACACTGTTAGCC

CCATCAACACTCCAGAAAAGCCACACTGCCTCAAAAAAGATTGACCAACAACGCCAGCCC

TCAGGAAATATTCCACGGCAGTGACAAGGCAAACACTGCCCGATAACGGAGAGTACAACT

CCCTCAGGAGAAAGAAAACAACAAGCAAGATGAAGAAGCTGAGAAACCACCCCCAGTCAA

ACCAACAGGAGAACTCACCTAAAACAGTCAACAATGAAACAGATCTCTGCAGTCAGACAG

ACCTGGAGTTCAAAAGAGAAATAGTGAAAATACTGAAGGAATTAAGAGAAGATATGAACA

GTAATGCAGATACCCTCAGAAAGGAACTAGAAAATATAAGGAGGAGCCAAGAAAAACTAG

AACATTCATTTGCAGAGATGCAAACTGAACTAGGGGCAGTAAAAACCAGAATGAATAATG

CAGAAGAACGAATCAGTGATATGGAAGATAGAATAATGGAAATCACTCAATCTGGTCAAC

AGACAGAAAACCGAATCAAAAAACTGGAAAGCAATATAAGAGACCTATGGGATAATATAA

AGCGGGCCAATCTACGCATAATAGGAATTCCAGAAGGAGTAGAAAAAGATAAGGGAATGG

AAAATATATTTGAAGAAATTATCGCTGGAAACTTCCCAAATCTAAAGGATACTGGATTCA

AGATACAAGAAGCACAGAGGGCCCCAAACAAACTGAACCCAAACAGACCCACACCAAGAC

ACATCATAATAAAAATGGCAAAAGTTAGTGATAAAGAGAGGATCCTAAAGGCAGCAAGAG

AAAAACAGAATGTTACCTACAAGGGAACCCCCATAAGAATATCAGCTGATTTCTCTACAG

AAACACTACAGGCCAGGAGGGAATGGCAAGAGATATTTAAAGTGCTCAAAGGAAAAAATA

TGCAACCTAGAATACTTTATCCAGCAAGAATATCATTTAAAATAGAAGGGGAAATAAAAA

TTTTTCCCAACAAACAAAAACTTAAAGAATACAGCAACACAAAACCCAGGTTAAAGGAAA

TATTGAAAGGGCTTCTCTAAACCAAAAAGAAAGGAAGGAAAGGGAAGAAAAAAGAAAAGA

AAAAAAAAAAAAGAAGAAGAAGAAGAGGAAGAACTAGGACTGAGGAAACCGCAATCAGAG

AGCAGTCACTCAAATAAGCCAGCATACAGATTTAATCATGAACATGCTTCAAACAAAATA

AAATTAAAAAGAAAAAAATAAAAGAGTCATCAAAACCATAAAATGTGGGCAAGGGATGTT

AGGAGGTAAATAACCCTTTTTGTTTGTATGTATGTCTCTCTTCTTAATTTTAATATAATA

ATGAAGTGTTTGAACTTACAGGACCATCAGGCTAAAACACACAATTATGGGAAGGGGTTA

GCATACTTAAAAAACAGGGCAACCACAAGCCAAAACCAAATATTGCATTTGCAAAAAATG

AAAAAAAAAAATACACTCAAGCAGATAATAACAGGAGACCATCCAACCAAAAAAAAAAAA

AAAAAAAAAAAAAAGAAGAATGGAGAACCATAGAATCAACTGGAACACGAGGATCAAATG

GCAATAAATAATCATCTATCAATTATCACCTTAAATGTCAATGGACTGAATGCCCCAATC

AAAAGACACAGAATGGCTGAGTGGATAAAAAGGCAAAAACCTTCAATATGCTGCCTACAA

GAAACTCACCTTAGGACAAAAGATACATATAGATTGAAAGTGAAAGGCTGGGGAAAAGTA

TTTCATGCCAATAGACATGACAGAAAAGCAGGAGTCGCAACGCTCATATCAGACAAAATA

GACTTTAAAACAAAAGACATAAAGAAAGACAAAGAAGGACACTATTTAATGATTAAGGGA

TCCATCCAAGGAGAGGATGTTACTATCATCAACATATATGCCCCAAACATAGGAGCACCC

AGATACATACAACAAATATTAACAGACATAAAGGGAGATATTGATGAGAATACAATCATA

GTAGGAGACCTAAATACCCCCCTCACATCAATGGACAGATCCTCTAGACAGAAAACCAAT

AAAGCAACAGAGATCCTAAAGGAAACAATAGAAAAGTTAGACTTCATTGATATCTTCAGG

ACACTACATCCAAAAAAATCAGAATACACATTCTTCTCAAATGCTCATGGAACATTCTCA

AGAATCGACCACATATTGGGACACAAAGCGAATCTCAATAAATTTAGGAGCATAGAAATT

ATCTCAAGTATCTTCTCTGACCACAATGCCATGAAATTAGAAATCAACCATGGGAAAAGC

AAAGAGAAAAAACCTACTCCATGGAGACTAAACAACATGCTACTAAAAAACCAATGGGTC

AATGAGGAAATCAAGAAGGAAATTAAAAACTACCTTGAAACAAATGATAATGAAGACACA

ACCTCTCAAAATCTATGGGATGCTGCGAAAGCAGTGCTCAGAGGGAAATTTATAGCAATC

CAGGCCTTTCTCAAAAAAGAAGAAAGATCCCAAATTGACAACTTAACCCTCCACCTAAAC

GAATTAGAAAAAGAAGAACAAAAAAGTCCTAAAGTCAGCAGAAGGAAGGAAATTATAAAG

ATCAAAGAAGAAATCAATAAAATAGAGACTCAAAAAACAATAGAGAAAATTAATAAAACC

AAGAGCTGGTTCTTTGAAAAGGTGAACAAAATTGACAAACCCCTGGCCAGACTCACTAAA

AAGAGGAGAGAAAGAACCCAAATCACCAAAATTATAAATGAAAAAGGAGAAATCACAACG

GATACAGCAGAAATACAAAAAACCATAAGAGAATACTATGAACAACTATATGGCAACAAG

TTTGACAATCTGGAAGAAATGGACAATTTTCTAGAATCTTACAGCCTGCCAAAACTGAAT

CAAGCAGAAACAGACCAACTGAACAGACCGATCACTAGAAATGAAATTGAAGAGGTCATA

AAATCACTCCCTACAAATAAAAGTCCAGGACCAGATGGCTTCACAGGTGAATTTTATCAA

ACATATAAAGAGGAATTGGTGCCCATCCTCCTTAAACTCTTTCAAAAGGTTGAAGAAGAA

GGAATACTCCCAAAGACATTCTATGAGGCCACCATCACCCTCATTCCAAAACCAGGCAGA

GATACCACCAAAAAAGAAAACTATCGCCCAATATCATTGATGAATATAGATGCAAAAATT

CTCAACAAAATCTTAGCCAACCGAATCCAACAACATATCAAAAAAATTATACACCATGAC

CAGGTTGGGTTCATCCCAGGTTCACAAGGATGGTTCAACATACGCAAATCAATCAGCATC

ATACACCACATTAACAAAAAAAAAGTCAAAAATCATATGATCATCTCAATAGACGCAGAA

AAAGCATTTGACAAAGTTCAACATCCATTCATGATCAAGACCCTCGCCAAAGTGGGTATA

GAGGGAACATTCCTGAATATAATCAAAGCCATTTATGATAAACCCACAGCAAATATAATC

CTCAATGGGGAAAAACTGAAAGCCTTCTCACTCAAATCTGGAACAAGACAGGGATGCCCA

CTCTCACCACTGCTCTTCAACATCGTTTTGGAAGTCTTAGCCACAGCAATTAGACAAACA

AAAGAAATCAAAGGCATCCATATAGGAAGAGAAGAGATCAAACTGTCACTGTATGCAGAT

GACATGATTCTATACCTAGAAAACCCTAAGGACTCAACCCCAAAACTCCTTGAACTGATT

AATAAATTCAGCAAAGTGGCAGGATATAAGATTAACATTCAGAAGTCAGTTGCATTTCTG

TATACCAGCAATGAAACATTAGAAAAGGAATACAAAAATACGATACCTTTTAAAATTGTA

CCTCACAAAATCAAATACCTCGGAATACACCTGACCAAAGAGGTAAAGGACCTATATGCC

GAGAACTATAAAACCTTAATCAAAGAAATCAAAGAAGATGTAAAGAAATGGAAAGATATT

CCATGTTCCTGGATTGGAAAAATCAATATTGTGAAAATGGCCATCCTACCCAAAGCAATC

TACAGATTCAATGCAATCCCTATCAAATTACCCATGACATTTTTCACAGAACTAGAACAA

ACAATCCAAACATTTATATGGAACCACAAAAGACCCAGAATCGCCAAAGCAATCCTGAGA

AACAAAAACCAAGCAGGAGGCATAACTCTCCCAGACTTCAAGAAATACTACAAAGCCACA

GTCATCAAAACAGTGTGGTACTGGTATCAAAACAGACAGACAGACCAATGGAACAGAATA

GAGAATCCGGAAATAAACCCTGACACCTATGGTCAATTAATCTTTGACAAGGGAGGCAAG

AACATAAAATGGGAAAAGGAAAGTCTATTCAGCAAGCATTGCTGGGAAACCTGGACAGCT

GCATGCAAAGCAATGAAACTAGAACACACCCTCACACCATGCACAAAAATAAACTCCAAA

TGGCTGAAAGACTTAAATATACGACAGGACACCATCAAACTCCTAGAAGAAAACATAGGC

AAAACACTCTCTGACATCAACATCATGAATATTTTCTCAGGTCAGTCTCCCAAAGCAATA

GAAATTAGAGCAAAAATAAACCCATGGGACCTCATCAAACTGAAAAGCTTTTGCACAGCA

AAGGAAACCCAAAAGAAAACAAAAAGACAACTTACAGAATGGGAGAAAATAGTTTCAAAT

GATGCAACTGACAAGGGCTTAATCTCTAGAATATATAAGCAACTTATACAACTCAACAGC

AAAAAAACCAATCAATCAATGGAAAAATGGGCAAAAGACCTGAATAGACATTTCTCCAAA

GAAGATATACAGATGGCCAACAAACACATGAAAAAATGCTCAACATCGCTGATTATAAGA

GAAATGCAAATCAAAACTACCATGAGATACCACCTCACACCAGTCAGAATGGCCATCATT

AATAAATCCACAAATAACAAGTGCTGGAGGGGCTGTGGAGAAAAGGGAACCCTCCTGCAC

TGTTGGTGGGAATGTAAACTGGTACAGCCACTATGGAGAACAGTTTGGAGATACCTTAGA

AATCTATACATAGAACTTCCATATGACCCTGCAATCCCACTCTTGGGCATCTATCCGGAC

AAAGCTCTACTTAAAAGAGACACATGCACCCGCATGTTCATTGCAGCACTATTCACAATA

GCCAGGACATGGAAACAATCCAAATGTCCATCGACAGAGATTGGATTCGGAAGAGGTGGT

ATATATACACAATGGAATACTACTCAGCCATAAAAAAGGATGACATCATGCCATTTGCAG

CAACATGGATGGAACTAGAGAATCTCATCCTGAGTGAAATGAGCCAGAAAGACAAAGACA

AATACCATATGATATCACTTATAACTGGAATCTAATATCCAGCACAAATGAACATCTCCT

CAGAAAAGAAAATCATGGACTTGGAGAAGAGACTTGTGGTTGCCTGATGGGAGGGGGAGG

GAGTGGGAGGGATCGGGAGCTTGGGCTTATCAGACACAACTAGAATAGATTTACAAGGAG

ATCCCGCTGAATAGCATTGAGAACTATGTCTAGATACTCATGTTGCAACAGAAGAAATGG

TGGGGGAAAAACTGTAATTGTAATGTATACATGTAAGGATAACCTGACCCCCTTGCTGTA

CAGTGGGAAAATAAAATTAAAAAAAAAAAAAAAAAA

>L1D18#LINE/L1D

GAGAGGACAAGATGGCGGAGGAGTAGGGGGACACGCTCGCCCTCTCCCACAAACACAACA

AAAAAAGCACATCTACAGAATAAATGACTCGCACAGAACAGCAACCAATCGCTGGCAGAG

GAACCTAAACTCCAATAACGGCAAGAAGTTCGTGACATTACTGGGCAGAACGGGAGAAAA

GAGGAGAGTGAGAGAAGGTGAATCCGAGCGGGACGGGCGCTCCCGAAAGGGAACTGCGGA

GGAGAAAGGGATCCCGCACCCTGGAAAGTCACCTACCGGGGGAAAGATCAAACGAACCGG

AGGAATCTCCAGATGCAGAGAAGAGTGTAGCAGTAAGTTGGAGTACGGAAAAGCCGATCA

AGAACCCAACGGACCATCTGAACTACGGGCACAGTCACCAAAAATTGAGACGCCTGGGTG

GGGGCTGGGCACCGAGTCCTCGGCTCCAGAGGTTAGTCCCCGAGAAAGGGCCGGGGGACG

CCTGGGTGGGGGCTGGGCACCGAGACCTCGGCTCCAGAGGTTAGTCCCGGAGGGGGCTGG

GGGGGGCGGGGCGGAGCGGAAACTGCTTGGGAGGTCTAGAAACCATTTGACGGGGCAGAG

ACTGCCTGGGAGACTAGAAAACAAAGCTGTCGCAGAGGAAGGGAACAATACTCTAGGGGC

GGGGAAGTGGAAAGCCGCATCAGAGGGAACCTGGGAGAAGAGCCTGGTCTGCGCCCGTGC

TGGGGAGGGGAGAGAAGAAGGGGTGGGTCCCCATAGAATACCCCCCACGCCACAGCAAGC

TTACAGGCCCGCTAGCTAGCAGAAAGCTGTGCTTCCCAGTGCATCCCCTCCCCCCACCCC

CGCCACCCCCTACGCTCTCACCGGACCTGGGGCTGCCTGCCATCCAGGAGGGCTGGCCTC

AACAATTGCCTGAAGCCTACCACCGCAGGGGCTGTCCCTGCACAGGCCTGCTTGCCCTTT

GGAGGGGCTACACTTCCGCAGAGCAGCACCAAACACCACCAGCCCCCGAGAAAAGGCCTG

CAGCCCAGAAAAGCTAGAACAAGCCTAGCCAGGCCGTGAATAGATCGCCTAATTCTCGGA

CGGTTTTTCTGAGTCGGGCTGCCCCGGGGAGGAGCCTCTTGGGTTTCCAACGGCCCTGCT

ACCCGCCCAAGCCCCCAGGGGGTGCCTAGTGGATCAGCTGCATAGGACTGCCAGCTCCAG

GCAGGACCCCCTGCAGCCCAGAAAAGCTGCAACAAGCCTGGCCGAGTCGGGAAAAGATCT

CAGACGGTTTTTCTGAGTCGGGCTGCCCTGGGGAGGAGCCTCTTGGGTCTCCAACGGCCC

TGCTACCCGCCCAAGCCCCCAGGGGGTGCAGCCTAGTGGACCAGCTGCATAGGACTGCCA

GCTCCAGGCAGGACCCCCTGCAGCCCAGAAAAGCTGCAACAAGCCTGGCCGAGTCGGGAA

AAGATCTCAGACGGTCTTTCTGAGTCGGGCTGCCCTGGGGAGGAGCCTCTTGGGTTTCCA

GTGGCCCTGCTACCCGCCCAAGCCCCCAGGGGGTGCCCCACTCCCGCGGAATAGCTGCTC

AGCACCACCAGCCCCCTGGAAGAGCCCCTGCAGCCCAGAAAAGCTGCAACAAGCTCGGCC

AGACTGTGAAAAGATCCGCCTACATTCTCAGGCTGTCCTTCTGAGTTGGGCTGCCCTAGG

GAAGAGCCTCTTAGGTTCTCAGTGACCCAGATAGCTGCTCCAGCCCCCAGGGGGTGCTGC

ACTCCTGAGGAACAGCTGCCCAACACCGCCAACCCCCTGCAAGAACCCCACAGCCTAAAA

ACACCAGAGCAAGCTCTGCATGACCAAGTGAAATCTGCTACCATCGTGGTGTGGACCTCC

CAGTCCTGTCTGCCCTCAGGAAGTCCTCCTTTGCTTCAAAGAAACACTGTTAGCCCCATC

AACACTCCAGAAAAGCCACACTGCCTCAAAAAAGATTGACCAACAACGCCAGCCCTCAGG

AAATATTCCACGGCAGTGACAAGGCAAACACTGCCCGATAACGGAGAGTACAACTCCCTC

AGGAGAAAGAAAACAACAAGCAAGATGAAGAAGCTGAGAAACCACCCCCAGTCAAACCAA

CAGGAGAACTCACCTAAAACAGTCAACAATGAAACAGATCTCTGCAGTCAGACAGACCTG

GAGTTCAAAAGAGAAATAGTGAAAATACTGAAGGAATTAAGAGAAGATATGAACAGTAAT

GCAGATACCCTCAGAAAGGAACTAGAAAATATAAGGAGGAGCCAAGAAAAACTAGAACAT

TCATTTGCAGAGATGCAAACTGAACTAGGGGCAGTAAAAACCAGAATGAATAATGCAGAA

GAACGAATCAGTGATATGGAAGATAGAATAATGGAAATCACTCAATCCGGTCAACAGACA

GAAAACCGAATCAAAAAACTGGAAAGCAATATAAGAGACCTATGGGATAATATAAAGCGG

GCCAATCTACGCATAATAGGAATTCCAGAAGGAGTAGAAAAAGATAAGGGGATGGAAAAT

ATATTTGAAGAAATTATCGCTGGAAACTTCCCAAATCTAAAGGATACTGGGTTCAAGATA

CAAGAAGCACAGAGGGCCCCAAACAAACTGAACCCAAACAGACCCACACCAAGACACATC

ATAATAAAAATGGCAAAAGTTAGTGATAAAGAGAGGATCCTAAAGGCAGCAAGAGAAAAA

CAGAATGTTACCTACAAGGGAACCCCCATAAGAATATCAGCTGATTTCTCTACAGAAACA

CTACAGGCCAGGAGGGAATGGCAAGAGATATTTAAAGTGCTAAAAGGAAAAAATATGCAA

CCTAGAATACTCTATCCAGCAAGAATATCATTTAAAATAGAAGGGGAAATAAAAATTTTT

CCCAACAAACAAAAACTTAAAGAATACAGCAACACAAAACCCAGGTTAAAGGAAATATTG

AAAGGGCTTCTCTAAACCAAAAAGAAAGGAAGGAAAGGGAAGAAAAAAGAAAAGAAAAAA

AAAAAAAGAAGAAGAGGAAGAACTAGGACTGAGGAAACCGCAATCAGAGAGCAGTCACTC

AAATAAGCCAGCATACAGATTTAATCATGAACATGCTTCAAACAAAATAAAATTAAAAAG

AAAAAAATAAAAAAGAGTCATCAAAACCATAAAATGTGGGCAAGGGATGTTAGGAAGTAA

ATAACCCTTTTTGTTTGTTTGTTGTATGTTTCTCTTCTTAATTTTAATATAGTAATGAAG

TGTTTGAACTTACAGGACCATCAGGCTAAAACACACAATTATGGGAAGGGGTTAGCATAC

TTAAAAAACAGGGCAACCACAAGCCAAAACCAAATATTGCATTTGCAAAAAATGAAAAAA

AAAAACACTCAAGCAGATAATAACAGGAGACCATCCAACCAAAAAAAAAAAAAAAAAAAA

AAAGAAAGGAAGAATGGAGAACCATAGAATCAACTGGAACACGAGGTTCAAATGGCAATA

AATAATCATCTATCAATTATCACCTTAAATGTCAATGGACTGAATGCCCCAATCAAAAGA

CACAGAGTGGCTGAGTGGATAAAAAGGCAAAAACCTTCAATATGCTGCCTACAAGAAACT

CACCTTAGGACAAAAGATACATATAGATTGAAAGTGAAAGGGTGGGGAAAAATATTTCAC

GCCAATAGACATGACAGAAAAGCAGGAGTCGCAACGCTCATATCAGACAAAATAGACTTT

AAAACAAAAGACATAAAGAAAGACAAAGAAGGACACTATTTAATGATTAAGGGATCCATC

CAAGGAGAGGATGTTACTATCATCAACATATATGCCCCAAATATAGGAGCACCCAGATAC

ATACAACAAATATTAACAGACATAAAGGGAGATATTGATGAGAATACAATCATAGTAGGA

GACCTTAATACCCCCCTCACATCAATGGACAGATCCTCTAGACAGAAAACCAATAAAGCA

ACAGAGATCCTAAAGGAAACAATAGAAAAGTTAGACTTAATTGATATCTTCAGGACACTA

CATCCAAAAAAATCAGAATACACATTCTTCTCAAATGCTCATGGAACATTCTCAAGAATC

GACCACATATTGGGACACAAAGCGAATCTCAATAAATTTAGGAGCATAGAAATTATCTCA

AGTATCTTCTCTGACCACAATGCCATGAAATTAGAAATCAACCATGGGAAAAGAAAGAGA

AAAAACCTACTACATGGAGACTAAACAACATGCTACTAAAAAACCAATGGGTCAATGAGG

AAATCAAGAAGGAAATTAAAAACTACCTTGAAACAAATGATAATGAAGACACAACCTCTC

AAAATCTATGGGATGCTGCGAAAGCAGTGCTCAGAGGGAAATTTATAGCAATACAGGCCT

TTCTCAAAAAAGAAGAAAGATCCCAAATTGACAACTTAACCCTCCACCTAAATGAATTAG

AAAAAGAAGAACAAAAAAGTCCTAAAGTCAGCAGAAGGAAGGAAATTATAAAGATCAAAG

AAGAAATCAATAAAATAGAGACTCAAAAAACAATAGAGAAAATTAATAAAACCAAGAGCT

GGTTCTTTGAAAAGGTGAACAAAATTGACAAACCCCTGGCCAGACTCACTAAAAAGAGGA

GAGAAAGAACCCAAATAACCAAAATTATAAATGAAAAAGGAGAAATCACAACGGATACAG

CAGAAATACAAAAAACCATAAGAGAATACTATGAACAACTATATGGCAACAAGTTTGACA

ATCTGGAAGAAATGGACAATTTTCTAGAATCTTACAGCCTGCCAAAACTGAATCAAGAGA

AACAGACCAACTGAACAGACCGATCACTAGAAATGAAATTGAAGAGGTCATAAAATCACT

CCCTACAAATAAAAGTCCAGGACCAGATGGCTTCACAGGTGAATTCTATCAAACATATAA

AGAGGAATTGGTGCCCATCCTCCTTAAACTCTTTCAAAAGGTTGAAGAAGAAGGAATACT

CCCAAAGACATTCTATGAGGCCACCATCACCCTCATTCCAAAACCAGACAGAGATACCAC

CAAAAAAGAAAACTATCGCCCAATATCATTGATGAATATAGATGCAAAAATTCTCAACAA

AATCTTAGCCAACCGAATCCAACAACATACCAAAAAAATTATACACCATGACCAGGTTGG

GTTCATCCCAGGTTCACAAGGATGGTTCAACATACGCAAATCAATCAGCATCATACACCA

CATTAACAAAAAAAAAGTCAAAAATCATATGATCATCTCAATAGACGCAGAAAAAGCATT

TGACAAAGTCCAACATCCATTCATGATCAAGACCCTCGCCAAAGTGGGTATAGAGGGAAC

ATTCCTGAATATAATCAAAGCCATTTATGATAAACCCACAGCAAATATAATCCTCAATGG

GGAAAAACTGAAAGCCTTCTCACTCAAATCTGGAACAAGACAGGGATGCCCACTCTCACC

ACTGCTCTTCAACATAGTTTTGGAAGTCCTAGCCACAGCAATTAGACAAACAAAAGAAAT

AAAAGGCATCCATATAGGAAGAGAAGAGATCAAACTGTCACTGTATGCAGATGACATGAT

ACTATACATAGAAAACCCTAAGGACTCAACCCCAAAACTACTTGAACTGATTAATAAATT

CAGCAAAGTAGCAGGATATAAGATTAACATTCAGAAGTCAGTTGCATTTCTGTATACCAG

CAATGAAACATTAGAAAAGGAATACAAAAATACGATACCTTTTAAAATTGTACCTCACAA

AATCAAATACCTCGGAATACACCTGACCAAGGAGGTAAAGGACCTATATGCCGAGAACTA

TAAAACTTTAATCAAAGAAATCAAAGAAGATGTAAAGAAATGGAAAGATATTCCATGTTC

CTGGATTGGGAAAATCAATATTGTAAAAATGGCCATACTACCCAAAGCAATCTACAGATT

CAATGCAATCCCTATCAAATTACCCATGACATTTTTCACAGAACTAGAACAAACAATCCA

AACATTTATATGGAACCACAAAAGACCCAGAATCGCCAAAGCAATCCTGAGAAACAAAAA

CCAAGCAGGAGGCATAACTCTCCCAGACTTCAAGAAATACTACAAAGCCACAGTCATCAA

AACAGTGTGGTACTGGTATCAAAACAGACAGACAGACCAATGGAACAGAATAGAGAATCC

GGAAATAAACCCTGACACCTATGGTCAATTAATCTTTGACAAGGGAGGCAAGAACATAAA

ATGGGAAAAAGAAAGTCTATTCAGCAAGCATTGCTGGGAAACCTGGACAGCTGCATGCAA

AGCAATGAAACTAGAACACACCCTCACACCATGCACAAAAATAAACTCCAAATGGCTGAA

AGACTTAAATATACGACAGGACACCATCAAACTCCTAGAAGAAAACATAGGCAAAACACT

CTCTGACATCAACATCATGAATATTTTCTCAGGTCAGTCTCCCAAAGCAATAGAAATTAG

AGCAAAAATAAACCCATGGGACCTCATCAAACTGAAAAGCTTTTGCACAGCAAAGGAAAC

CAAAAGAAAACAAAAAGACAACTTACAGAATGGGAGAAAATAGTTTCAAATGATGCAACT

GACAAGGGCTTAATCTCTAGAATATATAAACAACTTATACAACCCAACAGCAAAAAAACC

AATCAATCAATGGAAAAATGGGCAAAAGACCTGAATAGACATTTCTCCAAAGAAGATATA

CAGATGGCCAACAAACACATGAAAAAATGCTCAACATCGCTGATTATAAGAGAAATGCAA

ATCAAAACTACCATGAGATACCACCTCACACCAGTCAGAATGGCCATCATTAATAAATCC

ACAAATAACAAGTGCTGGAGGGGCTGTGGAGAAAAGGGAACCCTCCTGCACTGCTGGTGG

GAATGTAAACTGGTACAGCCACTATGGAGAACAGTTTGGAGATACCTTAGAAATCTATAC

ATAGAACTTCCATATGACCCCGCAATCCCACTCTTGGGCATCTATCCGGACAAAACTCTA

CTTAAAAGAGACACATGCACCCGCATGTTCATTGCAGCACTATTCACAATAGCCAGGACA

TGGAAACAACCCAAATGTCCATCGACAGATGATTGGATTCGGAAGAGTGGTATATATACA

CAATGGAATACTACTCAGCCATAAAAAAGAATGACATAATGCCATTTGCAGCAACATGGA

TGGAACTAGAGAATCTCATCTGAGTGAAATGAGCCAGAAAGACAAAGACAAATACCATAT

GATATCACTTATAACTGGAATCTAATATCCAGCACAAATGAACATCTCCTCAGAAAAGAA

AATCATGGACTTGGAGAAGAGACTTGTGGCTGCCTGATGGGAGGGGGAGGGAGTGGGAGG

GATCGGGAGCTTGGGCTTATCAGACACAACTTAGAATAGATTTACAAGGAGATCCTGCTG

AATAGCATTGAGAACTATGTCTAGATACTCATGTTGCAACAGAAGAAAGGGTGGGGGAAA

AATGTAATTGTAATGTATACATGTAAGGATAACCTGACCCCCTTGCTGTACAGTGGGAAA

AAAAAAAAAAAAAAAAAAAAAAAAAA

>L1D19#LINE/L1D

GAGAGAGGACAAGATGGCGGAGGAGTAGGGGGACACGCTCGCCCTCTCCCACAAACACAA

CAAAAAAAGCACATCTACAGAATAAATGACTCGCACAGAACAGCAACCAATCGCTGGCAG

AGGAACCTAAACTCCAATAACGGCAAGAAGTTCGTGACATTACTGGGCAGAACGGGAGAA

AAGAGGAGAGTGAGAGAAGGTGAATCCGAGCGGGACGGGCGCTCCCGAAAGGGAACTGCG

GAGGAGAAAGGGATCCCGCACCCTGGAAAGTCACCTACCGGGGGAAAGATCAAACAAACC

GGAGGAATCTCCAGATGCAGAGAAGAGTGTAGCAGTAAGTTGGAGTACGGAAAAGCCGAT

CAAGAACCCAACGGACCATCTGAACTACGGGCACAGTCACCAAAAATTGAGACGCCTGGG

TGGGGGCTGGGCACCGAGACCTCGGCTCCAGAGGTTAGTCCCCGGGCTGGGGGGGGCGGG

GCGGAGCGGAAACTGCTTGGGAGGTCTAGAAACCATTTGACGGGGCAGAGACTGCCTGGG

AGACTAGAAAACAAAGCTGTCGCAGAGGAAGGGAGCAATACTCTAGGGGCGGGGAAGTGG

AAAGCCGCATCAGAGGGAACCTGGGAGAAGAGCCTGGTCTGCGCCCGTGCTGGGGAGGGG

AGAGAAGAAGGGGTGGGTCCCCATAGAATACCCCCCACGCCACAGCAAGCTTACAGGCCC

GCTAGCTAGCAGAAAGCTGTGCTTCCCAGTGCATCCCCTCCCCCCACCCCCGCCACCCCC

TACGCTCTCACCGGACCTGGGGCTGCCTGCCATCCAGGAGGGCTGGCCTCAACAATTGCC

TGAAGCCTACCACCGCAGGGGCTGTCCCTGCACAGGCCTGCTTGCCCTTTGGAGGGGCTA

CACTTCCGCAGAGCAGCACCAAACACCACCAGCCCCCGAGAAAAGGCCTGCAGCCCAGAA

AAGCTAGAACAAGCCTAGCCAGGCCGTGAATAGATCGGCCTAATTCTCGGATGGTTTTTC

TGAGTCGGGCTGCCCCGGGGAGGAGCCTCTTGGGTCTCCAACGGCCCTGCTACCCGCCCA

AGCCCCCAGGGGGTGCCCTAGTGGATCAGCTGCATAGGACTGCCAGCTCCAGGCAGGACC

CCCTGCAGCCCAGAAAAGCTGCAACAAGCCTGGCCGAGTCGGGAAAAGATCTCAGACGGT

TTTTCTGAGTCGGGCTGCCCCGGGGAGGAGCCTCTTGGGTCTCCAAGGCCCTGCTACCCG

CCCAAGCCCCCAGGGGGTGCTGAGACCTAGTGGACCAGCTGCATAGGACTGCCAGCTCCA

GGCAGGACCCCCTGCAGCCCAGAAAAGCTGCAACAAGCCTGGCCGAGTCGGGAAAAGATC

TCAGACGGTCTTTCTGAGTCGGGCTGCCCTGGGGAGGAGCCTCTTGGGTTTCCAGTGGCC

CTGCTACCCGCCCAAGCCCCCAGGGGGTGCCCCACTCCCGCGGAATAGCTGCTCAGCACC

ACCAGCCCCCTGGAAGAGCCCCTGCAGCCCAGAAAAGCTGCAACAAGCTCAGCCAGACTG

TGAAAAGATCCGCCTACATTCTCAGGCTGTCCTTCTGAGTTGGGCTGCCCTAGGGAAGAG

CCTCTTAGGTTCTCAGTGACCCAGATAGCTGCTCCAGCCCCCAGGGGGTGCTGCACTCCT

GAGGAACAGCTGCCCAACACCGCCAACCCCCTGCAAGAACCCCACAGCCTAAAAACACCA

GAGCAAGCTCTGCATGACCAAGTGAAATCTGCTACCATCGTGGTGTGGACCTCCCAGTCC

TGTCTGCCCTCAGGAAGTCCTCCTTTGCTTCAAAGAAACACTGTTAGCCCCATCAACACT

CCAGAAAAGCCACACTGCCTCAAAAAAGATTGACCAACAACGCCAGCCCTCAGGAAATAT

TCCACGGCAGTGACAAGGCAAACACTGCCCGATAACGGAGAGTACAACTCCCTCAGGAGA

AAGAAAACAACAAGCAAGATGAAGAAGCTGAGAAACCACCCCCAGTCAAACCAACAGGAG

AACTCACCTAAAACAGTCAACAATGAAACAGATCTCTGCAGTCAGACAGACCTGGAGTTC

AAAAGAGAAATAGTGAAAATACTGAAGGAATTAAGAGAAGATATGAACAGTAATGCAGAT

ACCCTCAGAAAGGAACTAGAAAATATAAGGAGGAGCCAAGAAAAACTAGAACATTCATTT

GCAGAGATGCAAACTGAACTAGGGGCAGTAAAAACCAGAATGAATAATGCAGAAGAACGA

ATCAGTGATATGGAAGATAGAATAATGGAAATCACTCAATCCGGTCAACAGACAGAAAAC

CGAATCAAAAAACTGGAAAGCAATATAAGAGACCTATGGGATAATATAAAGCGGGCCAAT

CTACGCATAATAGGAATTCCAGAAGGAGTAGAAAAAGATAAGGGGATGGAAAATATATTT

GAAGAAATTATCGATGGAAACTTCCCAAATCTAAAGGATACTGGGTTCAAGATACAAGAA

GCACAGAGGGCCCCAAACAAACTGAACCCAAACAGACCCACACCAAGACACATCATAATA

AAAATGGCAAAAGTTAGTGATAAAGAGAGGATCCTAAAGGCAGCAAGAGAAAAACAGAAT

GTTACCTACAAGGGAACCCCCATAAGAATATCAGCTGATTTCTCTACAGAAACACTACAG

GCCAGGAGGGAATGGCAAGAGATATTTAAAGTGCTAAAAGGAAAAAATATGCAACCTAGA

ATACTCTATCCAGCAAGAATATCATTTAAAATAGAAGGGGAAATAAAAATTTTTCCCAAC

AAACAAAAACTTAAAGAATACAGCAACACAAAACCCAGGTTAAAGGAAATATTGAAAGGG

CTTCTCTAAACCAAAAAGAAAGGAAGGAAAGGGAAGAAAAAAGAAAAAAAAAAAAAAAAA

AGAAGAAGAAGAGGAAGAACTAGGACTGAGGAAACCGCAATCAGAGAGCAGTCACTCAAA

TAAGCCAGCATACAGATTTAATCATGAACATGCTTCAAACAAAATAAAATTAAAAAGAAA

AAAATAAAAAAGAGTCATCAAAACCATAAAATGTGGGCAAGGGATGTTAGGAAGTAAATA

ACCCTTTTTGTTTGTTTGTATGTTTCTCTTCTTAATTTTAATATAGTAATGAAGTGTTTG

AACTTACAGGACCATCAGGCTAAAACACACAATTATGGGAAGGGGTTAGCATACTTAAAA

AACAGGGCAACCACAAGCCAAAACCAAATATTGCATTTGCAAAAAATGAAAAAAAAAATA

CACTCAAGCAGATAATAACAGGAGACCATCCAACCAAAAAAAAAAAAAAAAAAAAAAGAA

AGGAAGAATGGAGAACCATAGAATCAACTGGAACACGAGGTTCAAATGGCAATAAATAAT

CATCTATCAATTATCACCTTAAATGTCAATGGACTGAATGCCCCAATCAAAAGACACAGA

GTGGCTGAGTGGATAAAAAGGCAAAAACCTTCAATATGCTGCCTACAAGAAACTCACCTT

AGGACAAAAGATACATATAGATTGAAAGTGAAAGGGTGGGGAAAAATATTTCACGCCAAT

AGACATGACAGAAAAGCAGGAGTCGCAACGCTCATATCAGACAAAATAGACTTTAAAACA

AAAGACATAAAGAAAGACAAAGAAGGACACTACTTAATGATTAAGGGATCCATCCAAGGA

GAGGATGTTACTATCATCAACATATATGCCCCAAATACAGGAGCACCCAGATACATACAA

CAAATATTAACAGACATAAAGGGAGATATTGATGAGAATACAATCATAGTAGGAGACCTT

AATACCCCCCTCACATCAATGGACAGATCCTCTAGACAGAAAACCAATAAAGCAACAGAG

ATCCTAAAGGAAACAATAGAAAAGTTAGACTTAATTGATATCTTCAGGACACTACATCCA

AAAAAAGCAGAATACACATTCTTCTCAAATGCTCATGGAACATTCTCAAGAATCGACCAC

ATATTGGGACACAAAGCGAATCTCAATAAATTTAGGAGCATAGAAATTATCTCAAGTATC

TTCTCTGACCACAATGCCATGAAATTAGAAATCAACCATGGGAAAAGGAAAGAGAAAAAA

CCTACTACATGGAGACTAAACAACATGCTACTAAAAAACCAATGGGTCAATGAGGAAATC

AAGAAGGAAATTAAAAACTACCTTGAAACAAATGATAATGAAGACACAACCTCTCAAAAT

CTATGGGATGCTGCGAAAGCAGTGCTCAGAGGGAAATTTATAGCAATACAGGCCTTTCTC

AAAAAAGAAGAAAGATCCCAAATTGACAACTTAACCCTCCACCTAAATGAATTAGAAAAA

GAAGAACAAAAAAGTCCTAAAGTCAGCAGAAGGAAGGAAATTATAAAGATCAAAGAAGAA

ATCAATAAAATAGAGACTCAAAAAACAATAGAGAAAATTAATAAAACCAAGAGCTGGTTC

TTTGAAAAGGTGAACAAAATTGACAAACCCCTGGCCAGACTCACTAAAAAGAGGAGAGAA

AGAACCCAAATCACCAAAATTATAAATGAAAAAGGAGAAATCACAACGGATACAGCAGAA

ATACAAAAAACCATAAGAGAATACTATGAACAACTATATGGCAACAAGTTTGACAATCTG

GAAGAAATGGACAATTTTCTAGAATCTTACAGCCTGCCAAAACTGAATCAAGTAGAAACA

GACCAACTGAACAGACCGATCACTAGAAATGAAATTGAAGAGGTCATAAAATCACTCCCT

ACAAATAAAAGTCCAGGACCAGATGGCTTCACAGGTGAATTCTATCAAACATATAAAGAG

GAATTGGTGCCCATCCTCCTTAAACTCTTTCAAAAGGTTGAAGAAGAAGGAATACTCCCA

AAGACATTCTATGAGGCCACCATCACCCTCATTCCAAAACCAGACAGAGATACCACCAAA

AAAGAAAACTATCGCCCAATATCATTGATGAATATAGATGCAAAAATTCTCAACAAAATC

TTAGCCAACCGAATCCAACAACATACCAAAAAAATTATACACCATGACCAGGTTGGGTTC

ATCCCAGGTTCACAAGGATGGTTCAACATACGCAAATCAATCAGCATCATACACCACATT

AACAAAAAAAAAGTCAAAAATCATATGATCATCTCAATAGACGCAGAAAAAGCATTTGAC

AAAGTCCAACATCCATTCATGATCAAGACCCTCGCCAAAGTGGGTATAGAGGGAACATTC

CTGAATATAATCAAAGCCATTTATGATAAACCCACAGCAAATATAATCCTCAATGGGGAA

AAACTGAAAGCCTTCTCACTCAAATCTGGAACAAGACAGGGATGCCCACTCTCACCACTG

CTCTTCAACATAGTTTTGGAAGTCCTAGCCACAGCAATTAGACAAACAAAAGAAATAAAA

GGCATCCATATAGGAAGAGAAGAGATCAAACTGTCACTGTATGCAGATGACATGATACTA

TACCTAGAAAACCCTAAGGACTCAACCCCAAAACTCCTTGAACTGATTAATAAATTCAGC

AAAGTAGCAGGATATAAGATTAACATTCAGAAGTCAGTTGCATTTCTGTATACCAGCAAT

GAAACATTAGAAAAGGAATACAAAAATACGATACCTTTTAAAATTGCACCTCACAAAATC

AAATACCTCGGAATACACCTGACCAAGGAGGTAAAGGACCTATATGCCGAGAACTATAAA

ACTTTAATCAAAGAAATCAAAGAAGATGTAAAGAAATGGAAAGATATTCCATGTTCCTGG

ATTGGGAAAATCAATATTGTGAAAATGGCCATACTACCCAAAGCAATCTACAGATTCAAT

GCAATCCCTATCAAATTACCCATGACATTTTTCACAGAACTAGAACAAACAATCCAAACA

TTTATATGGAACCACAAAAGACCCAGAATCGCCAAAGCAATCCTGAGAAACAAAAACCAA

GCAGGAGGCATAACTCTCCCAGACTTCAAGAAATACTACAAAGCCACAGTCATCAAAACA

GTGTGGTACTGGTACCAAAACAGACAGACAGACCAATGGAACAGAATAGAGAATCCGGAA

ATAAACCCTGACACCTATGGTCAATTAATCTTTGACAAGGGAGGCAAGAACATAAAATGG

GAAAAAGAAAGTCTATTCAGCAAGCATTGCTGGGAAACCTGGACAGCTGCATGCAAAGCA

ATGAAACTAGAACACACCCTCACACCATGCACAAAAATAAACTCAAAATGGCTGAAAGAC

TTAAATATACGACAGGACACCATCAAACTCCTAGAAGAAAACATAGGCAAAACACTCTCT

GACATCAACATCATGAATATTTTCTCAGGTCAGTCTCCCAAAGCAATAGAAATTAGAGCA

AAAATAAACCCATGGGACCTCATCAAACTGAAAAGCTTTTGCACAGCAAAGGAAACCCAA

AAGAAAACAAAAAGACAACTTACAGAATGGGAGAAAATAGTTTCAAATGATGCAACTGAC

AAGGGCTTAATCTCTAGAATATATAAGCAACTTATACAACCCAACAGCAAAAAAGCCAAT

CAATCAATGGAAAAATGGGCAAAAGACCTGAATAGACATTTCTCCAAAGAAGATATACAG

ATGGCCAACAAACACATGAAAAAATGCTCAACATCGCTGATTATAAGAGAAATGCAAATC

AAAACTACCATGAGATACCACCTCACACCAGTCAGAATGGCCATCATTAATAAATCCACA

AATAACAAGTGCTGGAGGGGCTGTGGAGAAAAGGGAACCCTCCTGCACTGCTGGTGGGAA

TGTAAACTGGTACAGCCACTATGGAGAACAGTTTGGAGATACCTTAGAAATCTATACATA

GAACTTCCATATGACCCCGCAATCCCACTCTTGGGCATCTATCCGGACAAAACTCTACTT

AAAAGAGACACATGCACCCGCATGTTCATTGCAGCACTATTCACAATAGCCAGGACATGG

AAACAACCCAAATGTCCATCGACAGATGATTGGATTCGGAAGAGGTGGTATATATACACA

ATGGAATACTACTCAGCCATAAAAAAGGATGACATAATGCCATTTGCAGCAACATGGATG

GAACTAGAGAATCTCATCCTGAGTGAAATGAGCCAGAAAGACAAAGACAAATACCATATG

ATATCACTTATAACTGGAATCTAATATCCAGCACAAATGAACATCTCCTCAGAAAAGAAA

ATCATGGACTTGGAGAAGAGACTTGTGGCTGCCTGATGGGAGGGGGAGGGAGTGGGAGGG

ATCGGGAGCTTGGGCTTATCAGACACAACTTAGAATAGATTTACAAGGAGATCCTGCTGA

ATAGCATTGAGAACTATGTCTAGATACTCATGTTGCAACAGAACAAAGGGTGGGGGAAAA

AATGTAATTGTAATGTATACATGTAAGGATAACCTGACCCCCTTGCTGTACAGTGGGAAA

ATAAAAAAAAAAATAAAAAAAAA

>L1D20#LINE/L1D

GAGAGGACAAGATGGCGGAGGAGTAGGGGGACACGCTCGCCCTCTCCCACAAACACAACA

AAAAAAGCACATCTACAGAATAAATGACTCGCACAGAACAGCAACCAATCGCTGGCAGAG

GAACCTAAACTCCAATAACGGCAAGAAGTTCGTGACATTATTGGGCAGAACGGGAGAAAA

GAGGAGAGTGAGAGAAGGTGAATCCGAGCGGGACGGGCGCTCCCGAAAGGGAACTGCGGA

GGAGAAAGGGATCCCGCACCCTGGAAAGTCACCTACCGGGGAAAGATCAAACGAACCGGA

GGAATCTCCAGATGCAGAGAAGAGTGTAGCAGTAAGTTGGAGTACGGAAAAGCCGATCAA

GAACCCAACGGACCATCTGAACTACGGGCACAGTCACCAAAAATTGAGACGCCTGGGTGG

GGGCTGGGCACCGAATCCTCGGCTCCAGAGGTTAGTCCCCGGGAAAGGGCCGGGGGACGC

CTGGGTGGGGGCTGGGCACCGAGACCTCGCCTCCGAAGGTTAGTCCCCGAGAAAGGGCCG

GGGGACGCCTGGGTGGGGGCTGGGCACCGAGACCTCGCCTCCGAAGGTTAGTCCCCGAGA

AAGGGCCGGGGGACGCCTGGGTGGGGGCTGGGCACCGAGACCTCGGCTCCAGAGATTAGT

CCCCGGGCTAGGGGGGCGGGGCAGAGCGGAAACTGCTTGGGAGGTCTAGAAACCATTTGA

CGGGGCAGAGACTGCCTGGGAGACTAGAAAACAAAGCTGTCGCAGAGGAAGGGAGCAATA

CTCTAGGGGCGGGGAAGTGGAAAGCCGCATCAGAGGGAACCTGGGAGAAGAGCCTGGTCT

GCGCCCGTGCTGGGGAGGGGAGAGAAGAAGGGGTGGGTCCCCATAGAATACCCCCCACGC

CACAGCAAGCTTACAGGCCCGCTAGCTAGCTGAAAGCTGTGCTTCCCAGTGCATCCCCTC

CCCCCACCCCCGCCACGCCCTACGCTCTCACCGGACCTGGGGCTGCCTGCCATCCAGGAG

GGCTGGCCTCAACAATTGCCTGAAGCCTACCACCGCAGGGGCTGTCCCTGCACAGGCCTG

CTTGCCCTTTGGAGGGGCTACACTTCCGCAGAGCAGCACCAAACACCACCAGCCCCCGAG

AAAAGGCCTGCAGCCCAGAAAAGCTAGAACAAGCTTAGCCAGGCTGTGAATAGATCGGCC

TAATTCTCGGACGGTTTTTCTGAGTCGGGTTGCCCCGGGGAGGAGCCTCTTGGGTTTCCA

ACGGCCCTGCTACCCGCCCAAGCCCCCAGGGGATGCTCTAGTGGACCAGCTGCATAGGAC

TGCCAGCTCCAGGCAGGACCCCCTGCAGCCCAGAAAAGCTGCAACAAGCCTGGCCGAGTC

GGGAAAAGATCTCAGACGGTTTTTCTGAGTCGGGCTGCCCTGGGGAGGAGCCTCTTGGGT

CTCCAAGGCCCTGCTACCCGCCCAAGCCCCCAGGGGGTGCCCCACTCCCGTGAAATAACT

GCTCAGCACCACCAGCCCCCTGGAAGAGCCCCTGCAGCCCAGAAAAGCTGCAACAAGCTC

GGCCAGACTGTGAAAAGATCCGCCTACATTCTCAGGCTGTCCTTCTGAGTTGGGCTGCCC

TAGGGAAGAGCCTCTTAGGTTCTCAGTGACCCAGATAGCTGCTCCAGCCCCCAGGGGGTG

ATGCACCCCTGAAGAGCAGCTGCCCAACACCGCCAACCCCCTGCAAGAACCCCACAGCCT

AAAAACACCAGAGCAAGCTCTGCATGACCAAGTGAAATCTGCTACCATCGTGGTGTGGAC

CTCCCAGTCCTGTCTGCCCTCAGGAAGTCCTCCTTTGCTTCAAAGAAACACTGTTAGCCC

CATCAACACTCCAGAAAAGCCACACTGCCTCAAAAAAGATTGACCAACAACGCCAGCCCT

CAGGAAATATTCCACGGCAGTGACAAGGCAAACACTGCCCGATAACGGAGAGTACAACTC

CCTCAGGAGAAAGAAAACAACAAGCAAGATGAAGAAGCTGAGAAACCACCCCCAGTCAAA

CCAACAGGAGAACTCACCTAAAACAGTCAACAATGAAACAGATCTCTGCAGTCAGACAGA

CCTGGAGTTCAAAAGAGAAATAGTGAAAATACTGAAGGAATTAAGAGAAGATATGAACAG

TAATGCAGATACCCTCAGAAAGGAACTAGAAAATATAAGGAGGAGCCAAGAAAAACTAGA

ACATTCATTTGCAGAGATGCAAACTGAACTAGGGGCAGTAAAAACCAGAATGAATAATGC

AGAAGAACGAATCAGTGATATGGAAGATAGAATAATGGAAATCACTCAATCTGGTCAACA

GACAGAAAACCGAATCAAAAAACTGGAAAGCAATATAAGAGACCTATGGGATAATATAAA

GCGGGCCAATCTACGCATAATAGGAATTCCAGAAGGAGTAGAAAAAGATAAGGGAATGGA

AAATATATTTGAAGAAATTATCGCTGGAAACTTCCCAAATCTAAAGGATACTGGATTCAA

GATACAAGAAGCACAGAGGGCCCCAAACAAACTGAACCCAAACAGACCCACACCAAGACA

CATCATAATAAAAATGGCAAAAGTTAGTGATAAAGAGAGGATCCTAAAGGCAGCAAGAGA

AAAACAGAATGTTACCTACAAGGGAACCCCCATAAGAATATCAGCTGATTTCTCTACAGA

AACACTACAGGCCAGGAGGGAATGGCAAGAGATATTTAAAGTGCTAAAAGGAAAAAATAT

GCAACCTAGAATACTCTATCCAGCAAGAATATCATTTAAAATAGAAGGGGAAATAAAAAT

TTTTCCCAACAAACAAAAACTTAAAGAATACAGCAACACAAAACCCAGGTTAAAGGAAAT

ATTGAAAGGGCTTCTCTAAACCAAAAAGAAAGGAAGGAAAGGGAAGAAAAAAGAAAAGAA

AAAAAAAAAAAGAAGAAGAAGAGGAAGAACTAGGACTGAGGAAACCGCAATCAGAGAGCA

GTCACTCAAATAAGCCAGCATACAGATTTAATCATGAACATGCTTCAAACAAAATAAAAT

TAAAAAGAAAAAAATAAAAAAGAGTCATCAAAACCATAAAATGTGGGCAAGGGATGTTAG

GAGGTAAATAACCCTTTTTGTTTGTATGTATGTCTCTCTTCTTAATTTTAATATAGTAAT

GAAGTGTTTGAACTTACAGGACCATCAGGCTAAAACACACAATTATGGGAAGGGGTTAGC

ATACTTAAAAAACAGGGCAACCACAAGCCAAAACCAAATATTGCATTTGCAAAAAATGAA

AAAAAAATACACTCAAGCAGATAATAACAGGAGACCATCCAACCAAAAAAAAAAAAAAAA

AAAAAAAAGAAAGGAAGAATGGAGAACCATAGAATCAACTGGAACACGAGGTTCAAATGG

CAATAAATAATCATCTATCAATTATCACCTTAAATGTCAATGGACTGAATGCCCCAATCA

AAAGACACAGAGTGGCTGAGTGGATAAAAAGGCAAAAACCTTCAATATGCTGCCTACAAG

AAACTCACCTTAGGACAAAAGATACATATAGATTGAAAGTGAAAGGGTGGGGAAAAATAT

TTCACGCCAATAGACATGACAGAAAAGCAGGAGTCGCAACGCTCATATCAGACAAAATAG

ACTTTAAAACAAAAGACATAAAGAAAGACAAAGAAGGACACTATTTAATGATTAAGGGAT

CCATCCAAGGAGAGGATGTTACTATCATCAACATATATGCCCCAAATATAGGAGCACCCA

GATACATACAACAAATATTAACAGACATAAAGGGAGATATTGATGAGAATACAATCATAG

TAGGAGACCTAAATACCCCCCTCACATCAATGGACAGATCCTCTAGACAGAAAACCAATA

AAGCAACAGAGATCCTAAAGGAAACAATAGAAAAGTTAGACTTAATTGATATCTTCAGGA

CACTACATCCAAAAAAACAGAATACACATTCTTCTCAAATGCTCATGGAACATTCTCAAG

AATCGACCACATATTGGGACACAAAGCGAATCTCAATAAATTTAGGAGCATAGAAATTAT

CTCAAGTATCTTCTCTGACCACAATGCCATGAAATTAGAAATCAACCATGGGAAAAGCAA

AGAGAAAAAACCTACTCCATGGAGACTAAACAACATGCTACTAAAAAACCAATGGGTCAA

TGAGGAAATCAAGAAGGAAATTAAAAACTACCTTGAAACAAATGATAATGAAGACACAAC

CTCTCAAAATCTATGGGATGCTGCGAAAGCAGTGCTCAGAGGGAAATTTATAGCAATACA

GGCCTTTCTCAAAAAAGAAGAAAGATCCCAAATTGACAACTTAACCCTCCACCTAAACGA

ATTAGAAAAAGAAGAACAAAAAAGTCCTAAAGTCAGCAGAAGGAAGGAAATTATAAAGAT

CAAAGAAGAAATCAATAAAATAGAGACTCAAAAAACAATAGAGAAAATTAATAAAACCAA

GAGCTGGTTCTTTGAAAAGGTGAACAAAATTGACAAACCCCTGGCCAGACTCACTAAAAA

GAGGAGAGAAAGAACCCAAATAACCAAAATTATAAATGAAAAAGGAGAAATCACAACGGA

TACAGCAGAAATACAAAAAACCATAAGAGAATACTATGAACAACTGTATGGCAACAAGTT

TGACAATCTGGAAGAAATGGACAATTTTCTAGAATCTTACAGCCTGCCAAAACTGAATCA

AGCAGAAACAGACCAACTGAACAGACCGATCACTAGAAATGAAATTGAAGAGGTCATAAA

ATCACTCCCTACAAATAAAAGTCCAGGACCAGATGGCTTCACAGGTGAATTCTATCAAAC

ATATAAAGAGGAATTGGTGCCCATCCTCCTTAAACTCTTTCAAAAGGTTGAAGAAGAAGG

AATACTCCCAAAGACATTCTATGAGGCCACCATCACCCTCATTCCAAAACCAGACAGAGA

TACCACCAAAAAAGAAAACTATCGCCCAATATCATTGATGAATATAGATGCAAAAATTCT

CAACAAAATCTTAGCCAACCGAATCCAACAACATACCAAAAAAATTATACACCATGACCA

GGTTGGGTTCATCCCAGGTTCACAAGGATGGTTCAACATACGCAAATCAATCAGCATCAT

ACACCACATTAACAAAAAAAAGTCAAAAATCATATGATCATCTCAATAGACGCAGAAAAA

GCATTTGACAAAGTTCAACATCCATTCATGATCAAGACCCTCGCCAAAGTGGGTATAGAG

GGAACATTCCTGAATATAATCAAAGCCATTTATGATAAACCCACAGCAAATATAATCCTC

AATGGGGAAAAACTGAAAGCCTTCTCACTCAAATCTGGAACAAGACAGGGATGCCCACTC

TCACCACTGCTCTTCAACATAGTTTTGGAAGTCTTAGCCACAGCAATTAGACAAACAAAA

GAAATAAAAGGCATCCATATAGGAAGAGAAGAGATCAAACTGTCACTGTATGCAGATGAC

ATGATACTATACCTAGAAAACCCTAAGGACTCAACCCCAAAACTCCTTGAACTGATTAAT

AAATTCAGCAAAGTGGCAGGATATAAGATTAACATTCAGAAGTCAGTTGCATTTCTGTAT

ACCAGCAATGAAACATTAGAAAAGGAATACAAAAATACGATACCTTTTAAAATTGTACCT

CACAAAATCAAATACCTCGGAATACACCTGACCAAGGAGGTAAAGGACCTATATGCCGAG

AACTATAAAACTTTAATCAAAGAAATCAAAGAAGATGTAAAGAAATGGAAAGATATTCCA

TGTTCCTGGATTGGAAAAATCAATATTGTGAAAATGGCCATACTACCCAAAGCAATCTAC

AGATTCAATGCAATCCCTATCAAATTACCCATGACATTTTTCACAGAACTAGAACAAACA

ATCCAAACATTTATATGGAACCACAAAAGACCCAGAATCGCCAAAGCAATCCTGAGAAAC

AAAAACCAAGCAGGAGGCATAACTCTCCCAGACTTCAAGAAATACTACAAAGCCACAGTC

ATCAAAACAGTGTGGTACTGGTATCAAAACAGACAGACAGACCAATGGAACAGAATAGAG

AATCCGGAAATAAACCCTGACACCTATGGTCAATTAATCTTTGACAAGGGAGGCAAGAAC

ATAAAATGGGAAAAAGAAAGTCTATTCAGCAAGCATTGCTGGGAAACCTGGACAGCTGCA

TGCAAAGCAATGAAACTAGAACACACCCTCACACCATGCACAAAAATAAACTCCAAATGG

CTGAAAGACTTAAATATACGACAGGACACCATCAAACTCCTAGAAGAAAACATAGGCAAA

ACACTCTCTGACATCAACATCATGAATATTTTCTCAGGTCAGTCTCCCAAAGCAATAGAA

ATTAGAGCAAAAATAAACCCATGGGACCTCATCAAACTGAAAAGCTTTTGCACAGCAAAG

GAAACCCAAAAGAAAACAAAAAGACAACTTACAGAATGGGAGAAAATAGTTTCAAATGAT

GCAACTGACAAGGGCTTAATCTCTAGAATATATAAGCAACTTATACAACTCAACAGCAAA

AAAACCAATCAATCAATGGAAAAATGGGCAAAAGACCTGAATAGACATTTCTCCAAAGAA

GATATACAGATGGCCAACAAACACATGAAAAAATGCTCAACATCGCTGATTATAAGAGAA

ATGCAAATCAAAACTACCATGAGATACCACCTCACACCAGTCAGAATGGCCATCATTAAT

AAATCCACAAATAACAAGTGCTGGAGGGGCTGTGGAGAAAAGGGAACCCTCCTGCACTGC

TGGTGGGAATGTAAACTGGTACAGCCACTATGGAGAACAGTTTGGAGATACCTTAGAAAT

CTATACATAGAACTTCCATATGACCCTGCAATCCCACTCTTGGGCATCTATCCGGACAAA

GCTCTACTTAAAAGAGACACATGCACCCGCATGTTCATTGCAGCACTATTCACAATAGCC

AGGACATGGAAACAACCCAAATGTCCATCGACAGATGATTGGATTCGGAAGAGTGGTATA

TATACACAATGGAATACTACTCAGCCATAAAAAAGGATGACATAATGCCATTTGCAGCAA

CATGGATGGAACTAGAGAATCTCATCCTGAGTGAAATGAGCCAGAAAGACAAAGACAAAT

ACCATATGATATCACTTATAACTGGAATCTAATATCCAGCACAAATGAACATCTCCTCAG

AAAAGAAAATCATGGACTTGGAGAAGAGACTTGTGGTTGCCTGATGGGAGGGGGAGGGAG

TGGGAGGGATCGGGAGCTTGGGCTTATCAGACACAACCTAGAATAGATTTACAAGGAGAT

CCTGCTGAATAGCATTGAGAACTATGTCTAGATACTCATGTTGCAACAGAAGAAAGGGTG

GGGGAAAAAACTGTAATTGCAATGTATACATGTAAGGATAACCTGACCCCCTTGCTGTAC

AGTGGGAAAAAAAAAAAAAAAAAAAAAAAA

>L1D21#LINE/L1D

GAGAGAGGACAAGATGGCGGAGGAGTAGGGGGACACGCTCGCCCTCTCCCACAAACACAA

CAAAAAAAGCACATCTACAGAATAAATGACTCGCACAGAACAGCAACCAATCGCTGGCAG

AGGAACCTAAACTCCAATAACGGCAAGAAGTTCGTGACATTACTGGGCAGAACGGGAGAA

AAGAGGAGAGTGAGAGAAGGTGAATCCGAGCGGGACGGGCGCTCCCGAAAGGGAACTGCG

GAGGAGAAAGGGATCCCGCACCCTGGAAAGTCACCTACCGGGGGAAAGATCAAACGAACC

GGAGGAATCTCCAGATGCAGAGAAGAGTGTAGCAGTAAGTTGGAGTACGGAAAAGCCGAT

CAAGAACCAACGGACCATCTGAACTACGGGCACAGTCACCAAAAATTGAGACGCCTGGGT

GGGGGCTGGGCACCGAACCTCGGCTCCAGAGGTTAGTCCCCGGGGGGGGGGCGGGGAGCG

GAAACTGCTTGGGAGGTCTAGAAACCATTTGACGGGGCAGAGACTGCCTGGGAGACTAGA

AAACAAAGCTGTCGCAGAGGAAGGGAGCAATACTCTAGGGGCGGGGAAGTGGAAAGCCGC

ATCAGAGGGAACCTGGGAGAAGAGCCTGGTCTGCGCCCGTGCTGGGGAGGGGAGAGAAGA

AGGGGTGGGTCCCCATAGAATACCCCCCACGCCACAGCAAGCTTACAGGCCCGCTAGCTA

GCTGAAAGCTGTGCTTCCCAGTGCATCCCCTCCCCCCACCCCCCCACCCCCTACGCTCTC

GCCGACCTGGGGCTGCCTGCCATCCAGGAGGGCTGGCCTCAACAATTGCCTGAAGCCTAC

CACCGCAGGGGCTGTCCCTGCACAGGCCTGCTTGCCCTTTGGAGGGGCTACACTTCCGCA

GAGCAGCACCAAACACCACCAGCCCCCGAGAAAAGGCCTGCAGCCCAGAAAAGCTAGAAC

AAGCCTAGCCAGGCCGTGAATAGATCTGCCTAATTCTCGGACGGTTTTTCTGAGTCGGGC

TGCCCCGGGGAGGAGCCTCTTGGGTTTCCAACGGCCCTGCTACCCGCCCAAGCCCCCAGG

GGGTGCAGCTAGTGGATCAGCTGCATAGGACTGCCAGCTCCAGGCAGGACCCCCTGCAGC

CCAGAAAAGCTGCAACAAGCCTGGCCGAGTCGGGAAAAGATCTCAGACGGTCTTTCTGAG

TCGGGCTGCCCTGGGGAGGAGCCTCTTGGGTTTCCAGGGCCCTGCTACCCGCCCAAGCCC

CCAGGGGGTGCCCCACTCCCGCGGAATAGCTGCTCAGCACCACCAGCCCCCTGGAAGAGC

CCCTGCAGCCCAGAAAAGCTGCAACAAGCTCGGCCAGACTGTGAAAAGATCCGCCTACAT

TCTCAGGCGTCCTTCTGAGTTGGGCTGCCCTAGGGAAGAGCCTCTTAGGTTCTCAGTGAC

CCAGATAGCTGCTCCAGCCCCCAGGGGGTGCTGCACTCCTGAGGAACAGCTGCCCAACAC

CGCCAACCCCCTGCAAGAACCCCACAGCCTAAAAACACCAGAGCAAGCTCTGCATGACCA

AGTGAAATCTGCTACCATCGTGGTGTGGACCTCCCAGTCCTGTCTGCCCTCAGGAAGTCC

TCCTTTGCTTCAAAGAAACACTGTTAGCCCCATCAACACTCCAGAAAAGCCACACTGCCT

CAAAAAAGATTGACCAACAACGCCAGCCCTCAGGAAATATTCCACGGCAGTGACAAGGCA

AACACTGCCCGATAACGGAGAGTACAACTCCCTCAGGAGAAAGAAAACAACAAGCAAGAT

GAAGAAGCTGAGAAACCACCCCCAGTCAAACCAACAGGAGAACTCACCTAAAACAGTCAA

CAATGAAACAGATCTCTGCAGTCAGACAGACCTGGAGTTCAAAAGAGAAATAGTGAAAAT

ACTGAAGGAATTAAGAGAAGATATGAACAGTAATGCAGATACCCTCAGAAAGGAACTAGA

AAATATAAGGAGGAGCCAAGAAAAACTAGAACATTCATTTGCAGAGATGCAAACTGAACT

AGGGGCAGTAAAAACCAGAATGAATAATGCAGAAGAACGAATCAGTGATATGGAAGATAG

AATAATGGAAATCACTCAATCCGGTCAACAGACAGAAAACCGAATCAAAAAACTGGAAAG

CAATATAAGAGACCTATGGGATAATATAAAGCGGGCCAATCTACGCATAATAGGAATTCC

AGAAGGAGTAGAAAAAGATAAGGGGATGGAAAATATATTTGAAGAAATTATCGCTGGAAA

CTTCCCAAATCTAAAGGATACTGGGTTCAAGATACAAGAAGCACAGAGGGCCCCAAACAA

ACTGAACCCAAACAGACCCACACCAAGACACATCATAATAAAAATGGCAAAAGTTAGTGA

TAAAGAGAGGATCCTAAAGGCAGCAAGAGAAAAACAGAATGTTACCTACAAGGGAACCCC

CATAAGAATATCAGCTGATTTCTCTACAGAAACACTACAGGCCAGGAGGGAATGGCAAGA

GATATTTAAAGTGCTAAAGGAAAAAATATGCAACCTAGAATACTCTATCCAGCAAGAATA

TCATTTAAAATAGAAGGGGAAATAAAAATTTTTCCCAACAAACAAAAACTTAAAGAATAC

AGCAACACAAAACCCAGGTTAAAGGAAATATTGAAAGGGCTTCTCTAAACCAAAAAGAAA

GGAAGGAAAGGGAAGAAAAAAGAAAAGAAAAAAAAAAAAAAGAAGAAGAAGAGGAAGAAC

TAGGACTGAGGAAACCGCAATCAGAGAGCAGTCACTCAAATAAGCCAGCATACAGATTTA

ATCATGAACATGCTTCAAACAAAATAAAATTAAAAAGAAAAAAATAAAAAAGAGTCATCA

AAACCATAAAATGTGGGCAAGGGATGTTAGGAAGTAAATAACCCTTTTTGTTTGTTTGTA

TGTTTCTCTTCTTAATTTTAATATAGTAATGAAGTGTTTGAACTTACAGGACCATCAGGC

TAAAACACACAATTATGGGAAGGGGTTAGCATACTTAAAAAACAGGGCAACCACAAGCCA

AAACCAAATATTGCATTTGCAAAAAATGAAAAAAAAAAATACACTCAAGCAGATAATAAC

AGGAGACCATCCAACCAAAAAAAAAAAAAAAAAAAAAAAAAGGAAGAATGGAGAACCATA

GAATCAACTGGAACACGAGGTTCAAATGGCAATAAATAATCATCTATCAATTATCACCTT

AAATGTCAATGGACTGAATGCCCCAATCAAAAGACACAGAGTGGCTGAGTGGATAAAAAG

GCAAAAACCTTCAATATGCTGCCTACAAGAAACTCACCTTAGGACAAAAGATACATATAG

ATTGAAAGTGAAAGGGTGGGGAAAAATATTTCACGCCAATAGACATGACAGAAAAGCAGG

AGTCGCAACGCTCATATCAGACAAAATAGACTTTAAAACAAAAGACATAAAGAAAGACAA

AGAAGGACACTACTTAATGATTAAGGGATCCATCCAAGGAGAGGATGTTACTATCATCAA

CATATATGCCCCAAATATAGGAGCACCCAGATACATACAACAAATATTAACAGACATAAA

GGGAGATATTGATGAGAATACAATCATAGTAGGAGACCTTAATACCCCCCTCACATCAAT

GGACAGATCCTCTAGACAGAAAACCAATAAAGCAACAGAGATCCTAAAGGAAACAATAGA

AAAGTTAGACTTAATTGATATCTTCAGGACACTACATCCAAAAAAACAGAATACACATTC

TTCTCAAATGCTCATGGAACATTCTCAAGAATCGACCACATATTGGGACACAAAGCGAAT

CTCAATAAATTTAGGAGCATAGAAATTATCTCAAGTATCTTCTCTGACCACAATGCCATG

AAATTAGAAATCAACCATGGGAAAAGGAAAGAGAAAAAACCTACTACATGGAGACTAAAC

AACATGCTACTAAAAAACCAATGGGTCAATGAGGAAATCAAGAAGGAAATTAAAAACTAC

CTTGAAACAAATGATAATGAAGACACAACCTCTCAAAATCTATGGGATGCTGCGAAAGCA

GTGCTCAGAGGGAAATTTATAGCAATACAGGCCTTTCTCAAAAAAGAAGAAAGATCCCAA

ATTGACAACTTAACCCTCCACCTAAACGAATTAGAAAAAGAAGAACAAAAAAGTCCTAAA

GTCAGCAGAAGGAAGGAAATTATAAAGATCAAAGAAGAAATCAATAAAATAGAGACTCAA

AAAACAATAGAGAAAATTAATAAAACCAAGAGCTGGTTCTTTGAAAAGGTGAACAAAATT

GACAAACCCCTGGCCAGACTCACTAAAAAGAGGAGAGAAAGAACCCAAATAACCAAAATT

ATAAATGAAAAAGGAGAAATCACAACGGATACAGCAGAAATACAAAAAACCATAAGAGAA

TACTATGAACAACTATATGGCAACAAGTTTGACAATCTGGAAGAAATGGACAATTTTCTA

GAATCTTACAGCCTGCCAAAACTGAATCAAGAGAAACAGACCAACTGAACAGACCGATCA

CTAGAAATGAAATTGAAGAGGTCATAAAATCACTCCCTACAAATAAAAGTCCAGGACCAG

ATGGCTTCACAGGTGAATTCTATCAAACATATAAAGAGGAATTGGTGCCCATCCTCCTTA

AACTCTTTCAAAAGGTTGAAGAAGAAGGAATACTCCCAAAGACATTCTATGAGGCCACCA

TCACCCTCATTCCAAAACCAGACAGAGATACCACCAAAAAAGAAAACTATCGCCCAATAT

CATTGATGAATATAGATGCAAAAATTCTCAACAAAATCTTAGCCAACCGAATCCAACAAC

ATACCAAAAAAATTATACACCATGACCAGGTTGGGTTCATCCCAGGTTCACAAGGATGGT

TCAACATACGCAAATCAATCAGCATCATACACCACATTAACAAAAAAAAAGTCAAAAATC

ATATGATCATCTCAATAGACGCAGAAAAAGCATTTGACAAAGTCCAACATCCATTCATGA

TCAAGACCCTCGCCAAAGTGGGTATAGAGGGAACATTCCTGAATATAATCAAAGCCATTT

ATGATAAACCCACAGCAAATATAATCCTCAATGGGGAAAAACTGAAAGCCTTCTCACTCA

AATCTGGAACAAGACAGGGATGCCCACTCTCACCACTGCTCTTCAACATAGTTTTGGAAG

TCCTAGCCACAGCAATTAGACAAACAAAAGAAATAAAAGGCATCCATATAGGAAGAGAAG

AGATCAAACTGTCACTGTATGCAGATGACATGATACTATACATAGAAAACCCTAAGGACT

CAACCCCAAAACTCCTTGAACTGATTAATAAATTCAGCAAAGTAGCAGGATATAAGATTA

ACATTCAGAAGTCAGTTGCATTTCTGTATACCAGCAATGAAACATTAGAAAAGGAATACA

AAAATACGATACCTTTTAAAATTGCACCTCACAAAATCAAATACCTCGGAATACACCTGA

CCAAGGAGGTAAAGGACCTATATGCCGAGAACTATAAAACTTTAATCAAAGAAATCAAAG

AAGATGTAAAGAAATGGAAAGATATTCCATGTTCCTGGATTGGAAAATCAATATTGTAAA

AATGGCCATACTACCCAAAGCAATCTACAGATTCAATGCAATCCCTATCAAATTACCCAT

GACATTTTTCACAGAACTAGAACAAACAATCCAAACATTTATATGGAACCACAAAAGACC

CAGAATCGCCAAAGCAATCCTGAGAAACAAAAACCAAGCAGGAGGCATAACTCTCCCAGA

CTTCAAGAAATACTACAAAGCCACAGTCATCAAAACAGTGTGGTACTGGTATCAAAACAG

ACAGACAGACCAATGGAACAGAATAGAGAATCCGGAAATAAACCCTGACACCTATGGTCA

ATTAATCTTTGACAAGGGAGGCAAGAACATAAAATGGGAAAAAGAAAGTCTATTCAGCAA

GCATTGCTGGGAAACCTGGACAGCTGCATGCAAAGCAATGAAACTAGAACACACCCTCAC

ACCATGCACAAAAATAAACTCCAAATGGCTGAAAGACTTAAATATACGACAGGACACCAT

CAAACTCCTAGAAGAAAACATAGGCAAAACACTCTCTGACATCAACATCATGAATATTTT

CTCAGGTCAGTCTCCCAAAGCAATAGAAATTAGAGCAAAAATAAACCCATGGGACCTCAT

CAAACTGAAAAGCTTTTGCACAGCAAAGGAAACCCAAAAGAAAACAAAAAGACAACTTAC

AGAATGGGAGAAAATAGTTTCAAATGATGCAACGACAAGGGCTTAATCTCTAGAATATAT

AAACAACTTATACAACCCAACAGCAAAAAAACCAATCAATCAATGGAAAAATGGGCAAAA

GACCTGAATAGACATTTCTCCAAAGAAGATATACAGATGGCCAACAAACACATGAAAAAA

TGCTCAACATCGCTGATTATAAGAGAAATGCAAATCAAAACTACCATGAGATACCACCTC

ACACCAGTCAGAATGGCCATCATTAATAAATCCACAAATAACAAGTGCTGGAGGGGCTGT

GGAGAAAAGGGAACCCTCCTGCACTGTGGTGGGAATGTAAACTGGTACAGCCACTATGGA

GAACAGTTTGGAGATACCTTAGAAATCTATACATAGAACTTCCATATGACCCCGCAATCC

CACTCTTGGGCATCTATCCGGACAAAACTCTACTTAAAAGAGACACATGCACCCGCATGT

TCATTGCAGCACTATTCACAATAGCCAGGACATGGAAACAACCCAAATGTCCATCGACAG

ATGATTGGATTCGGAAGAGGTGGTATATATACACAATGGAATACTACTCAGCCATAAAAA

AGAATGACATAATGCCATTTGCAGCAACATGGATGGAACTAGAGAATCTCATCCTGAGTG

AAATGAGCCAGAAAGACAAAGACAAATACCATATGATATCACTTATAACTGGAATCTAAT

ATCCAGCACAAATGAACATCTCCTCAGAAAAGAAAATCATGGACTTGGAGAAGAGACTTG

TGGCTGCCTGATGGGAGGGGGAGGGAGTGGGAGGGATCGGGAGCTTGGGCTTATCAGACA

CAACTTAGAATAGATTTACAAGGAGATCCTGCTGAATAGCATTGAGAACTATGTCTAGAT

ACTCATGTTGCAACAGAAGAAAGGGTGGGGGAAAAATGTAATTGTAATGTATACATGTAA

GGATAACCTGACCCCCTTGCTGTACAGTGGGAAAATAAAAAAAAAAAAAAAAAAAAAA

>7SLRNA#SINE

GCCGGGCGCGGTGGCGCGTGCCTGTAGTCCCAGCTACTCGGGAGGCTGAGGTGGGAGGAT

CGCTTGAGTCCAGGAGTTCTGGGCTGTAGTGCGCTATGCCGATCGGGTGTCCGCACTAAG

TTCGGCATCAATATGGTGACCTCCCGGGAGCGGGGGACCACCAGGTTGCCTAAGGAGGGG

TGAACCGGCCCAGGTCGGAAACGGAGCAGGTCAAAACTCCCGTGCTGATCAGTAGTGGGA

TCGCGCCTGTGAATAGCCACTGCACTCCAGCCTGAGCAACATAGCGAGACCCCGTCTCTT

AAAAAAAAAAAAAAAAAAAA

>AmnSINE1#SINE

GCCTGCAGCCATACCACCTCGGGCTGTGATCTCGTCAGATCTCACAAGCTAAGCAGGGTC

GGGCCTGGTCAGTACTTGGATGGGAGACCTCCAAGGAAACCCAGGTGCTGCAGGAAGTGG

TGCTGGTGATTCAGTAGGTGGCACTCTTCCCTCTGAGTCAGTACTGAACCAGTGCCCCAG

CGTGGTGTTAGGGGGCACTGTGCTGCTGGAGGTGCCGTCTTTCGGATGAGACGTAAAACC

GAGGTCCTGACCACTTGTGGTCATTAAAGATCCCATGGCACTTTTCGCAAGAGTAGGGGT

GTTAACCCCGGTGTCCTGGCCAAATTCCAATTTGGGTAATTACATTCTGCCTACCTAAAT

TCCCCCTGCAGTTTCAATTGGATACGGTATTCTTCACTTCCTGTCCTAAACTGTTGTGTA

GTGTTGCTGTGCGCTGTTAAACAGCTGCCGCGTTCCACCCCAGAGGTGGCTGCATTTCAG

TGGTGGGTGAAGTGATCCCTGTATGTAGCTTGTAAAGCGCTTTGGGATCCTTCGGGATGA

AAGGTGCTATATAAATGTAAGGTATTATTATTATT

>AmnSINE2#SINE

GCCGGAGGGGATGGCTTAGTGGTCTAAGCATCAGGTTTGAAATACCTAGACTCCCTGGAA

CCACAGGTTCAAATCCCAGCAGGGTCGACTCAGCCCTTCATCCTTCCGAGGTAGATAAAT

TGAGTNCCACGCAGTTTACTGTGTGNGGGTCTTTCGGATGAGACCTTAAAAACCAAAGCC

CTGTCTGCTCTGCGTGGACATTAAAGATCCCGTGGCGCTTTTCGTAAGAGTAGGGGTTTG

CCCCGGTGCCCTTGGCCGAATTTCTGGAACCCGGCTGAAANCCCGGGGAAACCGAGAGCC

TGGCTTATCCCTATAGGGTATTAGNCACCCTATAAAAGTTAAATTTATTTATTTATTT

>CHR-1#SINE

GGAGTTCCCTGGTGGCCTAGCGGTTAAGGATCCGGCGTTGTCACTGCTGTGGCNCAGGTT

CGATCCCTGGCCTGGGAACTTCCGCATGCCGCGGGCGCGGCCAAAAAAAAAAAAA

>CHR-2#SINE

GGGGCTTCCCTGGTGGCGCAGTGGTTAAGAATCCGCCTGCCAATGCAGGGGACACGGGTT

CGANCCCTGGTCCAGGAAGATCCCACATGCCGCGGAGCAACTAAGCCCGTGCGCCACAAC

TACTGAGCCTGCGCTCTAGAGCCCGCGAGCCACAACTACTGAGCCCATGTGCCGCAACTA

CTGAAGCCCACGCGCCTAGAGCCCGTGCTCCGCAACAAGAGAAGCCACCGCAATGAGAAG

CCCGCGCACCGCAACGAAGAGTAGCCCCCGCTCGCCGCAACTAGAGAAAGCCCGCGCGCA

GCAACGAAGACCCAACGCAGCCAATAAATAAATAAATAAATAAA

>CHRL#SINE

GGACTTCCCTGGTGGTCCAGTGGCTAAGACTCCACGCTCCCAATGCAGGGGGCCCAGGTT

CGATCCCTGGTCAGGGAACTAGATCCCACATGCTACAACTAAGAGTTCGCATGCCACAAC

TAAAGATCCCATGTGCYGCAACTAAGACCCGGCACAGCCAAATAAATA

>CRP1#SINE

GTGGTGGTGCCATGCCCACTTTTGCATAATAGCCTAGTGGTTAGAGCACTTGCCCAGGAA

GTGGGAGACCTGGGTTCTAGGCCCTCCTCAGCCTGAGGGGATTCAAACCCACATCTCCCA

CCTCCCAGGAGAGTGCCCTAACCACCAGGCTATAGGCTAGGCTGGGTGGGGGCACCCTCC

ACCCCTCCTGTTGAAGCTGGGCCACTGGGCAGCCAAGAATGCAAGATTCATGGGGCCAGA

AGGAGAGCAAGCAAGAG

>LFSINE_Vert#SINE

TCGGGGGGACTGGATGGCTCAGGGGATTGGTAATGGGATACGGAGCCTTTCACCTCTAGG

TCACTGGTTCGAATCCAGCCCAGGTCAGTAGTGACCGAAAGTCATTACCATCTGACGGCT

GTTCGGTGGCCTATGTGAAATGAGTTGGTGGTCTCAGTCCAGTTCCTAGTGGACAGGTGT

CCACATCACAAAACCACCATCACAATTGGCACTAATTGGCACCCTTGTTGGCAGTCTCAG

CAGAGAGGCCAAGGACTGAATGGGCATGGAGACTGAACTACCCTCTCACCTCTAGAGGTG

GTCCCTCCAGGTCAGGGTTGAGGCACATTGGCGGGGCAGTGTGGGGAAGCTTGCACTGCC

GCTGCCCGTGCTGTACCTGTTCTGTGGATAAACAGAGGACTTCAGTCTCCAGGGCTGTCA

ATCCGGCACCTTTCACGAGCACTAAATTCACTTAAAAAA

>LmeSINE1c#SINE

GGGTGTCGGTGRCTCAGTAGGTAGCACTCTTGCCTCTTAGTCAGAAGGTCGCAGGTTCAA

ATCCCACTTGAGGAACCTGGACAAACCCCCAGCAGGTTTTTGGAGTGCTATGCTACTGAA

GGTGTTTTCTTTCAGATGAGATGCCAAAGGAATGTTTTGCCTACCTTTTAGAGATCATAA

ATTCTATGCTATGTTTTGAAAGAATATGGGAGTCCTAGTCAAGTTCCCTCAATAGAGTCT

ATCAGGTCATAATGATTGGAAGTTGTTGTGTGATATGCATTCAAAACATAGTTGTTGTGT

TCCATCCCAAAGACAACACTTCAGTGACTTACATATGCCCTCAATGCAATGTAACATCCA

TGGATAAAACACTACAGAAATGTAAATTCTACTCTACTCTAATT

>MamSINE1#SINE

GTCTGTGGCTGGTTAGCTCAGTTGGTTAGAGCACGGTGCTAATGAGGCCAAGGTCACGGG

TTCGATCCCCGTGTGGGCCAGTTAGCTTTGCTCTGTTCCATGGCCACAGACTGCACCCCT

AACCCCAGCCAGCCGTCTCGCAAATGCGTGCTGTTGGTCACAAGGGGGACCGGCAGAGAG

TGTGGATGGNTCAGCGCAAATCCATCMCCACTACTGGAAAAACAACTCAAAGCACATGTC

CTACTGATAGTGGGTCAGTAGCGTCATCTTCGTGTACGAAGGACAGCACA

>MIR#SINE

ACAGTATAGCATAGTGGTTAAGAGCACGGACTCTGGAGCCAGACTGCCTGGGTTCGAATC

CCGGCTCTGCCACTTACTAGCTGTGTGACCTTGGGCAAGTTACTTAACCTCTCTGTGCCT

CAGTTTCCTCATCTGTAAAATGGGGATAATAATAGTACCTACCTCATAGGGTTGTTGTGA

GGATTAAATGAGTTAATACATGTAAAGCGCTTAGAACAGTGCCTGGCACATAGTAAGCGC

TCAATAAATGTTGGTTATTATT

>MIR1_Amn#SINE

TAGGGAGGCAGTGTGGTCTAGTGGATAGAGCACTGGACTGGGACTCGGGAGACCTGGGTT

CTATTCCCGGCTCTGCCACTGGCCTGCTGGGTGACCTTGGGCAAGTCACTTCACCTCTCT

GTGCCTCAGTTTCCCCATCTGTAAAATGGGGATAATGATACTGACCTCCTTTGTAAAGTG

CTTTGAGATCTACTGATGAAAAGTGCTACATAAGAGCTAGGTATTATTAT

>MIR3#SINE

CTGGCAGAGTGGCTGAGCAGAGAGAGCACGGACTGGGAGTCAGGAGACCTGGGTTCTAGT

CCCAGCTCTGCCACTAACTMGCTGTGTGACCTTGGGCAAGTCACTTCACCTCTCTGGGCC

TCAGTTTCCTCATCTGTAAAATGAGGGGGTTGGACTAGATGATCTCTAAGGTCCCTTCCA

GCTCTGACATTCTATGATTCTATGATTC

>MIRb#SINE

CAGAGGGGCAGCGTGGTGCAGTGGAAAGAGCACGGGCTTTGGAGTCAGGCAGACCTGGGT

TCGAATCCTGGCTCTGCCACTTACTAGCTGTGTGACCTTGGGCAAGTCACTTAACCTCTC

TGAGCCTCAGTTTCCTCATCTGTAAAATGGGGATAATAATACCTACCTCGCAGGGTTGTT

GTGAGGATTAAATGAGATAATGCATGTAAAGCGCTTAGCACAGTGCCTGGCACACAGTAA

GCGCTCAATAAATGGTAGCTCTATTATT

>MIRc#SINE

CGAGGCAGTGTGGTGCAGTGGAAAGAGCACTGGACTTGGAGTCAGGAAGACCTGGGTTCG

AGTCCTGGCTCTGCCACTTACTAGCTGTGTGACCTTGGGCAAGTCACTTAACCTCTCTGA

GCCTCAGTTTCCTCATCTGTAAAATGGGGATAATAATACCTGCCCTGCCTACCTCACAGG

GTTGTTGTGAGGATCAAATGAGATAATGTATGTGAAAGCGCTTTGTAAACTGTAAAGTGC

TATACAAATGTAAGGGGTTATTATTATT

>SINEA1_SSc#SINE/SINEA

GGAGTTCCCGTCGTGGCGCAGTGGTTAACGAATCCGACTAGGAACCATGAGGTTGCGGGT

TCGGTCCCTGCCCTTGCTCAGTGGGTTAACGATCCGGCGTTGCCGTGAGCTGTGGTGTAG

GTTGCAGACGCGGCTCGGATCCCGCGTTGCTGTGGCTCTGGCGTAGGCCGGTGGCTACAG

CTCCGATTCGACCCCTAGCCTGGGAACCTCCATATGCCGCGGGAGCGGCCCAAGAAATAG

CAACAACAACAACAACAAAAAAGACAAAAAGACCAAAAAAAAAAAAAAAAAAAA

>SINEA2_SSc#SINE/SINEA

GGAGTTCCCGTCGTGGCGCAGTGGTTAACGAATCCGACTAGGAACCATGAGGTTGCGGGT

TCGGTCCCTGCCCTTGCTCAGTGGGTTAACGATCCGGCGTTGCCGTGAGCTGTGGTGTAG

GTTGCAGACGCGGCTCGGATCCCGCGTTGCTGTGGCTCTGGCGTAGGCCGGCGGCTACAG

CTCCGATTCGACCCCTAGCCTGGGAACCTCCATATGCCGCGGGAGCGGCCCAAGAAATAG

CAAAAAGACAAAAAAAAAAAAAA

>SINEA3_SSc#SINE/SINEA

GGAGTTCCCGTCGTGGCGCAGTGGTTAACGAATCCGACTAGGAACCATGAGGTTGCGGGT

TCGGTCCCTGCCCTTGCTCAGTGGGTTAACGATCCGGCGTTGCCGTGAGCTGTGGTGTAG

GTTGCAGACGCGGCTCGGATCCCGCGTTGCTGTGGCTCTGGCGTAGGCCGGTGGCTACAG

CTCCGATTCGACCCCTAGCCTGGGAACCTCCATATGCCGCGGGAGCGGCCCAAGAAATAG

CAAAAAGACAAAAAAAAAAA

>SINEA4_SSc#SINE/SINEA

GGAGTTCCCGTCGTGGCNCAGTGGTTAACGAATCCGACTAGGAACCATGAGGTTGCGGGT

TCGATCCCTGGCCTTGCTCAGTGGGTTAAGGATCCGGCGTTGCCGTGAGCTGTGGTGTAG

GTCGCAGACGCGGCTCGGATCCCGCGTTGCTGTGGCTCTGGCGTAGGCCGGCGGCTACAG

CTCCGATTAGACCCCTAGCCTGGGAACCTCCATATGCCGCGGGAGCGGCCCTAGAAAAGG

CAAAAAGACAAAAAAAAAAAAAA

>SINEA5_SSc#SINE/SINEA

GGAGTTCCCGTCGTGGCTCAGTGGTTAACGAATCCGACTAGGAACCATGAGGTTGCGGGT

TCGATCCCTGGCCTCGCTCAGTGGGTTAAGGATCCGGCGTTGCCGTGAGCTGTGGTGTAG

GTCGCAGACGCGGCTCGGATCCCGCGTTGCTGTGGCTGTGGCGTAGGCCGGCGGCTACAG

CTCCGATTNGACCCCTAGCCTGGGAACCTCCATATGCCGCGGGTGCGGCCCTAGAAAAGA

CAAAAAGACAAAAAAAAAAAAAA

>SINEA6_SSc#SINE/SINEA

GGAGTTCCCGTCGTGGCGCAGCGGAAACGAATCCGACTAGGAACCATGAGGTTGCGGGTT

CGATCCCTGGCCTCGCTCAGTGGGTTAAGGATCCGGCGTTGCCGTGAGCTGTGGTGTAGG

TCGCAGACGCGGCTCGGATCCSGCGTTGCTGTGGCTGTGGCGTAGGCCGGCAGCTGCAGC

TCCGATTMGACCCCTAGCCTGGGAACCTCCATATGCCGCGGGTGCGGCCCTAAAAAGACA

AAAAACAAAAAAAAAAAAA

>SINEA7_SSc#SINE/SINEA

GGAGTTCCCGTCGTGGCTCAGCGGAAACGAATCCGACTAGTATCCATGAGGACGCGGGTT

CGATCCCTGGCCTCGCTCAGTGGGTTAAGGATCCGGCGTTGCCGTGAGCTGTGGTGTAGG

TCGCAGACGCGGCTCGGATCCGGCGTTGCTGTGGCTGTGGTGTAGGCCGGCAGCTGCAGC

TCCGATTCGACCCCTAGCCTGGGAACCTCCATATGCCGCGGGTGCGGCCCTAAAAAGACA

AAAAAAAAAAAAA

>SINEA8_SSc#SINE/SINEA

GGAGTTCCCGTCGTGGCTCAGCGGTAACGAACCCGACTAGTATCCATGAGGACGCGGGTT

CGATCCCTGGCCTCGCTCAGTGGGTTAAGGATCCGGCGTTGCCGTGAGCTGTGGTGTAGG

TCGCAGACGCGGCTCGGATCCCGCGTTGCTGTGGCTGTGGCGTAGGCCGGCAGCTGCAGC

TCCGATTCGACCCCTAGCCTGGGAACCTCCATATGCCGCGGGTGCGGCCCTAAAAAGACA

AAAAAAAAAAAAA

>SINEA9_SSc#SINE/SINEA

GGAGTTCCCGTTGTGGCTCAGCGGGTTAAGAACCCGACTAGTATCCATGAGGATGCGGGT

TCGATCCCTGGCCTCGCTCAGTGGGTTAAGGATCCGGCGTTGCCGCGAGCTGCGGTGTAG

GTCGCAGATGCGGCTCGGATCCGGCGTTGCTGTGGCTGTGGCGTAGGCCGGCAGCTGCAG

CTCCGATTCGACCCCTAGCCTGGGAACTTCCATATGCCGCAGGTGCGGCCCTAAAAAGAA

AAAAAAAAAAAAAA

>SINEA10_SSc#SINE/SINEA

GGAGTTCCCGCTGTGGCTCAGCGGGTTAAGRACCCGACGTWGTSTCCGTGAGGATGCGGG

TTCGATCCCTGGCCTCGCTCAGTGGGTTAAGGATCCGGCGTTGCCGCAAGCTGCGGCGTA

GGTCGCAGATGCGGCTCGGATCCGGCGTTGCCGTGGCTGTGGCGTAGGCCGGCAGCTGCA

GCTCCGATTCGACCCCTAGCCCGGGAACTTCCATATGCCGCAGGTGCGGCCNTAAAAAGA

AAAAAAAAAAAAAAA

>SINEA11_SSc#SINE/SINEA

GGAGTTCCCGTCGTGGCGCAGTGGTTAACGAATCCGACTAGGAACCACGAGGTTGCGGGT

TCGATCCCTGGCCTCGCTCAGTGGGTTAAGGATCCGGCGTTGCCGTGAGCTGTGGTGTAG

GTCGCAGACGCGGCTCGGATCCCGCGTTGCTGTGGCTCTGGCGTAGGCCGGCGGCTACAG

CTCCGATTNGACCCCTAGCCTGGGAACCTCCNTATGCCNCAGGNGCGGCCCTAGAAAAGG

CAAAAAAAAAAAAA

>SINEB1_SSc#SINE/SINEB

GGAGTTCCCGTCGTGGCGCAGCGGAAACGAATCCGACTAGGAACCATGAGGTTGCGGGTT

CGATCCCTGGCCTCGCTCAGTGGGTTAAGGATCCGGCGTTGCCGTGAGCTGTGGTGTAGG

CCGGCGGCTGCAGCTCCGATTNGACCCCTAGCCTGGGAACCTCCATATGCCGCGGGTGCG

GCCCTAAAAAAGGCAAAAAAAAAAAAAAAAAAAAAA

>SINEB2_SSc#SINE/SINEB

GGAGTTCCCGTCGTGGCTCAGCGGTTAACGAACCCGACTAGTATCCATGAGGACGCGGGT

TCGATCCCTGGCCTCGCTCAGTGGGTTAAGGATCCGGCGTTGCTGTGGCTGTGGCGTAGG

CCGGCAGCTGCAGCTCCGATTCGACCCCTAGCCTGGGAACTTCCATATGCCGCGGGTGCG

GCCCTAAAAAAGGCAAAAAAANAAAAAAAAAAAAAA

>SINEB3_SSc#SINE/SINEB

GGAGTTCCCGCTGTGGCGCAACGGGATCGGCGGCGTCTCGGGAGCGCCGGGACGCAGGTT

CGATCCCCGGCCCGGCACAGTGGGTTAAGGATCCGGCGTTGCCGCAGCTGCGGCKTAGGT

CGCAACTGCGGCTCGGATCTGATCCCTGGCCCGGGAACTCCATATGCCGCGGGGCGGCCA

AAAAAGAAAAAAAAAAAAAAAA

>SINEB4_SSc#SINE/SINEB

GGAGTTCCCGCTGTGGCGCAGTGGGTTAAGGATCCGACTGCAGCGGCTCGGGTCGCTGCG

GAGGCGCGGGTTCGATCCCCGGCCCGGCGCAGTGGGTTAAAGGATCCGGCGTTGCCGCAG

CTGTGGCGTAGGTCGCAGCTGCGGCTCGGATTCAATCCCTGGCCCGGGAACTTCCATATG

CCGCGGGTGCGGCCAAAAAAAGAAAAAAAANAAAAAAAAAAAAAA

>SINEB5_SSc#SINE/SINEB

GGAGTTCCCGCTGTGGCGCAGTGGGTTAAGGATCCGGCGTTGTCTCTGTGGCGGCGCGGG

TTCGATCCCCGGCCTGGCGCAGTGGGTTAAGGATCCGGCGTTGCCGCAGCTGTGGCGTAG

GTCGCAGCTGCGGCTCGGATTCGATCCCTGGCCCGGGAACTTCCATATGCCGCGGGTGCG

GCCAAAAAAAGAAAAAAAANAAAAAAAAAAAAAA

>SINEB6_SSc#SINE/SINEB

GGGAGTTCCCGCCGTGGCGCAGTGGGTTAAGAATCCGACTGCAGCGGCTCGGGTCGCTGC

GGAGGCGCGGGTTCGATCCCCGGCCCGGCGCAGTGGGTTAAAGGATCCGGCGTTGCTGCA

GCTGCGGCGTAGGTCGCAGCTGCGGCTCGGATTCGATCCCTGGCCCGGGAACTTCCATAT

GCCGCGGGTGCGGCCGTAAAAAGAAAAAAAAAAAAAATCGT

>SINEC1_SSc#SINE/SINEC

GGAGTTCCCTGGTGGCTCAGYGGGTTAAGGATCTAGTGTTGTCACTGCTGTGGCTCRGGT

CACTGCTGTGGTGCAGGTTTGATCCCTGGCCTGGGAACTTCCACATGCYGYRGGCATGGC

CAAAAAAAAAA

>SINEC2_SSc#SINE/SINEC

GGGAGTTCTCTTGTGGCACAGCAGGTTAAGGATCCAGCGTTGTCACTGCAGTGGCTTGGG

TCGCTGCTGTGGCACGGGTTCAATCCCTGGCCCAGGAACTTCCACATGCCACGGGCATGG

CCAAAAAAAAA

>SINEC3_SSc#SINE/SINEC

GGAGTTCCCTGGTGGCTCAGTGGGTTAAGGATCCGGCATTGTCACTGCTGTGGCTCGGGT

TCGATCCCTGGCCCAGGAACTTCTGCATGCCGTGGGCGCGGCCAAAAAAAAAAAAAAAA

>SINEC4_SSc#SINE/SINEC

GGAGTTCCCTGGTGGCTCAGCGGGTTAAGGATCCGGCGTTGTCACTGCTGTGGCTCTGGT

TACNGCTGTGGCACGGGTTCAATCCCTGGCCCGGGAACTTCCGCATGCCACGGGCGCGGC

CAAAAAAAAAAAAA

>SINEC5_SSc#SINE/SINEC
[truncated: 164,556 more chars]
