# Supplementary material for: Retrotransposons evolution and impact on lncRNA and protein coding genes in pigs
Source: Mob DNA. 2019 May 6;10:19. doi: 10.1186/s13100-019-0161-8 (PMC6501411; doi:10.1186/s13100-019-0161-8)
Supplement: Supplementary file 2 — Table S1. Detail information of pig L1 families in the pig genome. Table S2. (SINE name and length information after reclassification). Table S3. (Detailed information on ERV in the pig genome). Table S4. (Composition of interspersed repeats in the pig genome). Table S5. (Primers for insertion polymorphism detection of youngest retrotransposons). Table S6. (Primers for promoter activity assay of LTR /5′UTR of ERV/ L1). Table S7. (Primers for retrotransposon assay of L1). Table S8. (Primers for detection of the expression of retrotransposons by RT-qPCR). Figure S1. (Comparison and reclassification of SINE transposons derived from tRNA in Repbase libraries by sequence alignment). Figure S2. (ERVs were classified into three classes based on the NJ phylogenetic tree). Figure S3. (Schematic of the protein structures of full-length ERV6 members in pig genome). Figure S4. (Retrotransposon distribution in pig genome and the impact on genes). Figure S5. (Primers designed for the youngest retrotransposons insertion polymorphism detection). (DOCX 2580 kb) [file 13100_2019_161_MOESM2_ESM.docx]

**Table S1.** Detail information of pig L1 families in the pig genome

| **Family** | **Subfamily** | **Length (bp)** | | | | | | **Genomic copy number used for calssification ^a^** | **No of acitve L1s^b^** | **5'UTR Divergence (K) mean±SE ^c^** |
| --- | --- | --- | --- | --- | --- | --- | --- | --- | --- | --- |
|  |  | **Consensus** | **5’UTR** | **ORF1** | **IGR** | **ORF2** | **3’UTR(No PolyA)** |  |  |  |
| L1A | L1A1 | 5937 | 941 | 905 | 68 | 3814 | 187 | 375 |  | 0.063±0.001(N=1190) |
|  | L1A2 | 5837 | 939 | 904 | 67 | 3725 | 183 | 19 |  | 0.077±0.002(N=1212) |
|  | L1A3 | 6240 | 932 | 902 | 392 | 3810 | 186 | 524 |  | 0.134±0.003(N=970) |
|  | L1A4 | 6170 | 941 | 904 | 393 | 3663 | 235 | 16 |  | 0.199±0.001(N=6264) |
|  | L1A5 | 6073 | 931 | 901 | 393 | 3655 | 180 | 36 |  | 0.216±0.004(N=830) |
|  | L1A6(HAL1_SSc) | 3016 | 944 | 906 |  |  |  |  |  | 0.069±0.001(N=1482) |
|  | L1A7(L1_3_SSc) | 7404 | 1959 | 897 | 396 | 3828 | 305 |  |  | 0.221±0.002(N=1792) |
| L1B | L1B1(L1B-SS) | 7419 | 2005 | 891 | 392 | 3808 | 268 | 48 |  | 0.106±0.002(N=665) |
|  | L1B2 | 7740 | 2335 | 889 | 390 | 3805 | 267 | 49 |  | 0.124±0.002(N=732) |
|  | L1B3 | 7442 | 2041 | 884 | 390 | 3805 | 269 | 34 |  | 0.126±0.003(N=235) |
|  | L1B4 | 7108 | 1640 | 901 | 438 | 3797 | 305 | 17 |  | 0.165±0.006(N=146) |
|  | L1B5 | 7163 | 1680 | 905 | 444 | 3801 | 269 | 24 |  | 0.155±0.002(N=1161) |
|  | L1B6 | 7545 | 2033 | 910 | 447 | 3807 | 269 | 12 |  | 0.137±0.002(N=785) |
|  | L1B7 | 7688 | 2208 | 908 | 422 | 3813 | 269 | 12 |  | 0.123±0.003(N=110) |
|  | L1B8 | 6297 | 873 | 884 | 399 | 3807 | 267 | 11 |  | 0.134±0.003(N=349) |
|  | L1B9 | 6699 | 1250 | 907 | 434 | 3783 | 272 | 29 |  | 0.17±0.002(N=1368) |
|  | L1B10 | 6597 | 1214 | 878 | 422 | 3766 | 272 | 14 |  | 0.189±0.001(N=1633) |
|  | L1B11 | 5975 | 551 | 894 | 405 | 3795 | 267 | 11 |  | 0.187±0.011(N=42) |
|  | L1B12 | 6545 | 1203 | 887 | 433 | 3798 | 217 | 14 |  | 0.200±0.002(N=1444) |
| L1C | L1C1 | 7125 | 1641 | 891 | 529 | 3794 | 262 | 14 |  | 0.077±0.003(N=187) |
|  | L1C2 | 7532 | 2024 | 889 | 516 | 3812 | 268 | 13 |  | 0.102±0.004(N=127) |
|  | L1C3 | 6897 | 1414 | 890 | 489 | 3813 | 261 | 22 |  | 0.095±0.010(N=41) |
|  | L1C4 | 6873 | 1413 | 891 | 484 | 3803 | 261 | 37 |  | 0.087±0.003(N=118) |
|  | L1C5 | 6744 | 1368 | 889 | 426 | 3789 | 247 | 25 |  | 0.168±0.040(N=8) |
|  | L1C6 | 6842 | 1411 | 890 | 450 | 3808 | 257 | 33 |  | 0.101±0.004(N=170) |
|  | L1C7 | 7081 | 1603 | 890 | 493 | 3804 | 261 | 29 |  | 0.111±0.005(N=112) |
|  | L1C8 | 7389 | 1971 | 889 | 495 | 3766 | 251 | 11 |  | 0.173±0.010(N=43) |
|  | L1C9 | 7008 | 1585 | 889 | 461 | 3799 | 251 | 48 |  | 0.121±0.003(N=137) |
|  | L1C10 | 6919 | 1556 | 879 | 385 | 3797 | 258 | 9 |  | 0.182±0.017(N=19) |
|  | L1C11 | 6799 | 1359 | 889 | 444 | 3814 | 264 | 22 |  | 0.178±0.004(N=302) |
|  | L1C12 | 6669 | 1252 | 890 | 433 | 3798 | 260 | 18 |  | 0.172±0.003(N=215) |
|  | L1C13 | 6462 | 1037 | 888 | 440 | 3808 | 259 | 24 |  | 0.209±0.009(N=23) |
| L1D | L1D1 | 8063 | 2500 | 891 | 519 | 3819 | 277 | 23 | 9 | 0.043±0.010(N=73) |
|  | L1D2 | 8438 | 2876 | 891 | 517 | 3819 | 277 | 28 | 22 | 0.036±0.007(N=92) |
|  | L1D3 | 8234 | 2687 | 891 | 515 | 3819 | 277 | 34 | 18 | 0.020±0.005(N=65) |
|  | L1D4 | 8610 | 3064 | 891 | 519 | 3819 | 277 | 12 | 7 | 0.036±0.011(N=40) |
|  | L1D5 | 7838 | 2297 | 891 | 518 | 3812 | 273 | 19 | 6 | 0.044±0.009(N=60) |
|  | L1D6 | 8822 | 3254 | 891 | 521 | 3819 | 277 | 33 | 14 | 0.027±0.011(N=43) |
|  | L1D7 | 7462 | 1916 | 891 | 517 | 3819 | 273 | 22 | 9 | 0.041±0.008(N=50) |
|  | L1D8 | 7340 | 1831 | 891 | 501 | 3818 | 274 | 78 | 3 | 0.038±0.004(N=140) |
|  | L1D9 | 7645 | 2108 | 891 | 516 | 3819 | 273 | 31 | 4 | 0.027±0.003(N=67) |
|  | L1D10 | 7303 | 1776 | 891 | 513 | 3817 | 273 | 27 | 3 | 0.057±0.015(N=37) |
|  | L1D11 | 7616 | 2086 | 891 | 504 | 3817 | 274 | 13 |  | 0.06±0.008(N=79) |
|  | L1D12 | 7224 | 1715 | 891 | 513 | 3807 | 271 | 135 | 1 | 0.044±0.004(N=23) |
|  | L1D13 | 7072 | 1562 | 887 | 509 | 3811 | 270 | 148 | 2 | 0.048±0.007(N=47) |
|  | L1D14 | 7246 | 1731 | 891 | 506 | 3818 | 272 | 19 |  | 0.063±0.005(N=202) |
|  | L1D15 | 7379 | 1871 | 891 | 505 | 3811 | 272 | 14 |  | 0.054±0.004(N=126) |
|  | L1D16 | 7380 | 1867 | 890 | 511 | 3814 | 272 | 161 |  | 0.043±0.004(N=104) |
|  | L1D17 | 7296 | 1769 | 891 | 517 | 3818 | 272 | 47 |  | 0.114±0.017(N=47) |
|  | L1D18 | 7586 | 2064 | 891 | 516 | 3814 | 272 | 124 |  | 0.050±0.003(N=145) |
|  | L1D19 | 7523 | 1998 | 891 | 516 | 3819 | 273 | 24 |  | 0.050±0.002(N=143) |
|  | L1D20 | 7530 | 2008 | 891 | 517 | 3816 | 274 | 44 |  | 0.056±0.003(N=233) |
|  | L1D21 | 7318 | 1798 | 890 | 517 | 3814 | 272 | 197 |  | 0.080±0.003(N=353) |

^a^ Genomic copy number elements indicated the elements with 5’UTR, ORF1, ORF2, and 3’UTR and were used for classification.

^b^ Number of active elements indicated the FL elements with intact ORF1 and ORF2 frames with coding capability.

^c^ Divergence (k) 5’UTR mean±SE: The average number of substitutions per site (K) for 5’UTR was estimated according to the divergence levels reported by RepeatMasker using the one-parameter Jukes-Cantor formula K = -300/4×Ln(1-D×4/300) as described previously (Waterston et al. 2002), where D represents the proportion of sites that differ between the fragmented repeat and the consensus sequence.

**Table S2. SINE name and length information after reclassification**

| **Family** | **Subfamily name** | **Repbase name** | **Length(no polyA tail/bp)** |
| --- | --- | --- | --- |
| SINEA | SINEA1 | PRE1_SS#SINE | 265 |
|  | SINEA2 | PRE1a#SINE | 249 |
|  | SINEA3 | Pre0_SS#SINE | 249 |
|  | SINEA4 | PRE1b#SINE | 249 |
|  | SINEA5 | PRE1c#SINE | 249 |
|  | SINEA6 | PRE1d#SINE | 239 |
|  | SINEA7 | PRE1e#SINE | 239 |
|  | SINEA8 | PRE1f#SINE | 239 |
|  | SINEA9 | PRE1g#SINE | 238 |
|  | SINEA10 | PRE1h#SINE | 239 |
|  | SINEA11 | SINE5-SS#SINE | 241 |
| SINEB | SINEB1 | PRE1d2#SINE | 194 |
|  | SINEB2 | PRE1f2#SINE | 194 |
|  | SINEB3 | PRE1i#SINE | 186 |
|  | SINEB4 | PRE1j#SINE | 202 |
|  | SINEB5 | PRE1k#SINE | 191 |
|  | SINEB6 | SINE4-SS#SINE | 203 |
| SINEC | SINEC1 | SINE1_SS#SINE | 121 |
|  | SINEC2 | SINE1A_SS#SINE | 122 |
|  | SINEC3 | SINE1B_SS#SINE | 103 |
|  | SINEC4 | SINE1C_SS#SINE | 121 |
|  | SINEC5 | SINE1D_SS#SINE | 121 |
|  | SINEC6 | SINE3_SS#SINE | 102 |
|  | SINEC7 | SINE4_SS#SINE | 113 |
|  | SINEC8 | SUSINE2#SINE | 123 |

**Table S3. Detail information about ERV in the pig Genome**

| **Family/Subfamily** | **No of ERVs with RT** | | **Repbase**  **name** | **Length (bp)** | | | | | **Number of LTR sequences aligned** | **Genomic**  **Copy number** | **Full**  **ERV** | **Active**  **ERV ^a^** |
| --- | --- | --- | --- | --- | --- | --- | --- | --- | --- | --- | --- | --- |
|  | **LTRHarvest** | **Retrotector** |  | **Consensus/**  **Representative (chromosome: start-end strand Sscrofa11.1)** | **LTR** | ***gag*** | ***pol*** | ***env*** |  |  |  |  |
| **Total** | 240 | 29 |  |  |  |  |  |  |  |  |  |  |
| **Class I (Gamma)** |  |  |  |  |  |  |  |  |  |  |  |  |
| **ERV1** | 10 | 65 | ERV1-1_SSc | 8970 | 431 | 1450 | 3299 |  | 388 | 3002 |  |  |
| **ERV2** |  | 15 | ERV1-4_SSc | 7250 | 347 | 1279 | 2822 |  | 217 | 449 |  |  |
| **ERV3** |  | 4 |  | 9059  (chrX:61527959-61537017 -) | 448 | 1440 | 3361 |  | 69 | 114 |  |  |
| **ERV4** | 1 | 8 | ERV1N-3_SSc | 5018 | 429 |  | 1769 |  | 126 | 198 |  |  |
| **ERV5** | 1 | 3 |  | 8402  (chr1:51158530-51166931 +) | 720 |  | 3366 |  | 12 | 88 |  |  |
| **ERV6** | 13 | 29 |  |  |  |  |  |  |  |  |  |  |
| **ERV6A** |  |  | ERV1-2_SSc | 8918  (chr5:92185133-92194050 -) | 702 | 1575 | 3435 | 1977 | 66 | 117 | 11 | 1 |
| **ERV6B** |  |  |  | 8757  (chr9:138895584-138904340 -) | 629 | 1575 | 3441 | 1963 | 94 | 129 | 8 | 1 |
| **ERV7** |  | 3 |  | 11283  (chr12:45523978-45535562 -) | 268 |  |  |  |  |  |  |  |
| **ERV8** | 1 | 7 | ERV1-3_SSc | 6412 | 308 |  | 3057 |  | 1673 | 2373 |  |  |
| **ERV9** |  | 11 |  | 8180  (chr6:150080007-150088187 +) | 104 |  | 1728 |  |  |  |  |  |
| **ERV10** |  | 13 | ERV1N-1A2_SSc | 7868 | 359 |  | 1846 |  | 301 | 432 |  |  |
| **ERV11** | 1 | 9 |  | 8491  (chr15:114036218-114044703 +) | 318 |  |  |  | 14 | 400 |  |  |
| **ERV12** |  | 18 |  | 10008  (chr18:29379437-29389439 +) | 119 |  | 2974 |  |  |  |  |  |
| **ERV13** | 1 | 10 |  | 14157  (chr4:53228382-53242538 +) | 252 |  |  |  | 1672 | 325374 |  |  |
| **Class III (Spuma)** |  |  |  |  |  |  |  |  |  |  |  |  |
| **ERV14** |  | 2 |  | 11719  (chrX:76070252-76081970 +) | 281 |  |  |  | 224 | 154512 |  |  |
| **Class II (Beta)** |  |  |  |  |  |  |  |  |  |  |  |  |
| **ERV15** |  | 2 |  | 4890  (chrX:51285507-51290396 -) |  |  |  |  |  |  |  |  |
| **ERV16** | 1 | 16 |  | 9740  (chr4:82737665-82747402 -) | 352 |  |  |  | 4 | 173990 |  |  |
| **ERV17** |  | 3 |  | 6666  (chr1:195801779-195808444 -) | 184 |  |  |  | 635 | 61202 |  |  |
| **ERV18** | 1 | 22 |  | 8229  (chr2:145909645-145917873 -) | 498 |  |  |  | 2791 | 3208 |  |  |

a Full length (FL) ERV indicated the ERV with LTR-GAG-POL-ENV-LTR features.

b Active ERV indicated the ERV harboring ENV, GAG, POL coding sequences, coding sequences predicted by the Sorted Six-Frame translation program of Bioediter

**Table S4. Composition of interspersed repeats in the pig genome**

| **Type of repeat** | **Count** | **Length (Mb)** | **Percent (%)** |
| --- | --- | --- | --- |
| **RNA transposons** | **3736482** | **929086546** | **37.13** |
| **LINE** | **957337** | **463445362** | **18.52** |
| L1A | 230442 | 97616013 | 3.90 |
| L1B | 237169 | 116283428 | 4.65 |
| L1C | 17962 | 12350299 | 0.49 |
| L1D | 25327 | 28322858 | 1.13 |
| L1other | 338301 | 176410030 | 7.05 |
| L2 | 91745 | 28281933 | 1.13 |
| other | 16391 | 4180801 | 0.17 |
| **LTR** | **1277102** | **189214071** | **7.56** |
| ERVI | 744584 | 77721039 | 3.11 |
| ERVII | 187314 | 17585930 | 0.70 |
| ERVIII | 332347 | 90579758 | 3.62 |
| Gypsy | 7062 | 1840357 | 0.07 |
| other | 5795 | 1486987 | 0.06 |
| **SINE** | **1502043** | **276427113** | **11.05** |
| SINEA | 842774 | 191219168 | 7.64 |
| SINEB | 280796 | 39407938 | 1.58 |
| SINEC | 164839 | 15849919 | 0.63 |
| MIR | 184041 | 27291907 | 1.09 |
| other | 29593 | 2658181 | 0.11 |
| **DNA transposons** | **232700** | **50559049** | **1.99** |
| TcMar | 5298 | 935083 | 0.03 |
| hAT | 45681 | 12786097 | 0.50 |
| other | 181721 | 36837869 | 1.46 |
| **Others** | **760216** | **39813821** | **1.60** |
| **Total** | **4729398** | **1019459416** | **40.72** |

These data are annotated with the custom library by using RepeatMasker (RepeatMasker -open-4.0.5) with cutoff 250 of Sscrofa11.1 genome.

**Table S5. Primers for insertion polymorphism detection of youngest retrotransposons**

| **Primer name** | **Primer sequence** | **Location for polymorphism detection** | **Introduction of primers** |
| --- | --- | --- | --- |
| L1D1-IP1-F | TCACCAAGTCTCTGATCATCTTACA | Sscrofa11.1 chr13:160714048-160714448 + | Primers for polymorphism detection of two insertion sites of L1D1 |
| L1D1-IP1-R | CTTTCTCCTCCGCAGTTCC |  |  |
| L1D1-IP2-F | GAAAAATGACCCTCCCCATT | Sscrofa11.1 chr1:131728148-131728549 - |  |
| L1D1-IP2-R | TCTGCCCAATAATGTCACGA |  |  |
| SINEA1-IP1-F | CTACACAACCCCCAGTTGGT | Sscrofa11.1 chr9:85579482-85580111 + | Primers for polymorphism detection of two insertion sites of SINEA1 |
| SINEA1-IP1-R | GAAAACGGAGAGCTTGATGG |  |  |
| SINEA-IP2-F | CATGGTGTTTGTCAGGAGGA | Sscrofa11.1 chr1:184927250-184927926 + |  |
| SINEA-IP2-R | GGAAAGAAAGTGCAATCCAGA |  |  |
| ERV6B-IP1-F | TTGCACAGAGATTGTGGACTT | Sscrofa11.1 chrX:70660337-70660842 - | Primers for polymorphism detection of two insertion sites of ERV6B |
| ERV6B-IP1-R | GGCAGAGTGACAGATTGCAG |  |  |
| ERV6B-IP2-F | CTGCCACCTGGAGACAGAAT | Sscrofa11.1 chr9:138904057-138904619 - |  |
| ERV6B-IP2-R | CCTGGGCAGAGTGACAGATT |  |  |

**Table S6. Primers for promoter activity assay of LTR /5’UTR of ERV/ L1**

| **Primer name** | **Primer sequence** | **Introduction of primers** |
| --- | --- | --- |
| L1D1-5’UTR-pro-F1 | ACCAAGCAGGAGGCATCACT | Primers for first round of PCR amplification based on Sscrofa11.1:chrX:62454289-62456959:-(L1D1) |
| L1D1-5’UTR-pro-R1 | TGCAGAGATCAGTTTCATTGTTG |  |
| L1D1-5’UTR-sense-pro-F2 (Kpn l) | **GGTACC**CACAGTCATCAAATCAGTGTGGT | Primers for second round of PCR amplification for 5'UTR fragment to construct pGL3-L1D1-5'UTR-sense-Luc vector |
| L1D1-5’UTR-sense-pro-R2 (MluI) | **ACGCGT**CTTGCTTGTTGTCTTCTTTCTC |  |
| L1D1-5’UTR-antisense-pro-F2 (MluI) | **ACGCGT**CACAGTCATCAAATCAGTGTGGT | Primers for second round of PCR amplification for 5'UTR fragment to construct pGL3-L1D1-5'UTR-antisense-Luc vector |
| L1D1-5’UTR-antisense-pro-R2 (Kpn l) | **GGTACC**CTTGCTTGTTGTCTTCTTTCTCCTG |  |
| L1D2-5’UTR-1-pro-F1 | TACCTCTTTCACCCCAAGTGT | Primers for first round of PCR amplification based on Sscrofa11.1:chr11:57675261-57678312:-(L1D2) |
| L1D2-5’UTR-1-pro-R1 | TGCAGAGATCAGTTTCATTGTTG |  |
| L1D2-5’UTR-sense1-pro-F2 (Kpn l) | **GGTACC**TACCTCAAATGAAAGATCCTTTG | Primers for second round of PCR amplification for 5'UTR fragment to construct pGL3-L1D2-5'UTR-sense1-Luc vector |
| L1D2-5’UTR-sense1-pro-R2 (MluI) | **ACGCGT**CTTGCTTGTTGTCTTCTTTCTC |  |
| L1D2-5’UTR-antisense1-pro-F2 (MluI) | **ACGCGT**TACCTCAAATGAAAGATCCTTTG | Primers for second round of PCR amplification for 5'UTR fragment to construct pGL3-L1D2-5'UTR-antisense1-Luc vector |
| L1D2-5’UTR-antisense1-pro-R2 (Kpn l) | **GGTACC**CTTGCTTGTTGTCTTCTTTCTCCTG |  |
| L1D2-5’UTR-2-pro-F1 | ACAGAATTGTGCATTCACCAACC | Primers for first round of PCR amplification based on Sscrofa11.1:chrX:60938371-60941402:+(L1D2) |
| L1D2-5’UTR-2-pro-R1 | GCAGAGATCAGTTTCATTGTTGACT |  |
| L1D2-5’UTR-sense2-pro-F2 (Kpn l) | **GGTACC**CCAACCCTGTCTAATTCTAGAACAT | Primers for second round of PCR amplification for 5'UTR fragment to construct pGL3-L1D2-5'UTR-sense2-Luc vector |
| L1D2-5’UTR-sense2-pro-R2 (MluI) | **ACGCGT**CTTGCTTGTTGTCTTCTTTCTC |  |
| L1D3-5’UTR-1-pro-F1 | CCTCACAACCGCCTATCATCCAC | Primers for first round of PCR amplification based on Sscrofa11.1:chr6:169299813-169302525:-(L1D3) |
| L1D3-5’UTR-1-pro-R1 | GCTCCTTTCTGAGGGTATCTGCAT |  |
| L1D3-5’UTR-sense1-pro-F2 (Kpn l) | **GGTACC**AAAAATGGTGGGAGAGAGGACA | Primers for second round of PCR amplification for 5'UTR fragment to construct pGL3-L1D3-5'UTR-sense1-Luc vector |
| L1D3-5’UTR-sense1-pro-R2 (MluI) | **ACGCGT**TCTCAGCTTCTTCATCTTGC |  |
| L1D3-5’UTR-antisense1-pro-F2 (MluI) | **ACGCGT**AAAAATGGTGGGAGAGAGGACA | Primers for second round of PCR amplification for 5'UTR fragment to construct pGL3-L1D3-5'UTR-antisense1-Luc vector |
| L1D3-5’UTR-antisense1-pro-R2 (Kpn l) | **GGTACC**TCTCAGCTTCTTCATCTTGC |  |
| L1D3-5’UTR-2-pro-F1 | TTGAAAGATGCAAGAACTTGGC | Primers for first round of PCR amplification based on Sscrofa11.1:chr9:95589565-95592407:-(L1D3) |
| L1D3-5’UTR-2-pro-R1 | TGCAGAGATCAGTTTCATTGTTG |  |
| L1D3-5’UTR-sense2-pro-F2 (Kpn l) | **GGTACC**TGCCACAAAATGCCCCAAGT | Primers for second round of PCR amplification for 5'UTR fragment to construct pGL3-L1D3-5'UTR-sense2-Luc vector |
| L1D3-5’UTR-sense2-pro-R2 (MluI) | **ACGCGT**CTTGCTTGTTGTCTTCTTTCTC |  |
| L1D4-5’UTR-pro-F1 | TGAAAAAGCCTAAACACTACCATT | Primers for first round of PCR amplification based on Sscrofa11.1:chrX:99122574-99125802:-(L1D4) |
| L1D4-5’UTR-pro-R1 | TGCAGAGATCAGTTTCATTGTTG |  |
| L1D4-5’UTR-sense-pro-F2 (Kpn l) | **GGTACC**CATTGTCTTTCAGAGTGCCCTA | Primers for second round of PCR amplification for 5'UTR fragment to construct pGL3-L1D4-5'UTR-sense-Luc vector |
| L1D4-5’UTR-sense-pro-R2 (MluI) | **ACGCGT**CTTGCTTGTTGTCTTCTTTCTC |  |
| L1D6-5’UTR-pro-F1 | CTGCAAAAGCTTACTAAAAAG | Primers for first round of PCR amplification based on Sscrofa11.1:chr8:42602320-42605653:+(L1D6) |
| L1D6-5’UTR-pro-R1 | TGCAGAGATCAGTTTCATTGTTG |  |
| L1D6-5’UTR-sense-pro-F2 (Kpn l) | **GGTACC**CCAGTAGTATTCATTCTGGTTAG | Primers for second round of PCR amplification for 5'UTR fragment to construct pGL3-L1D6-5'UTR-sense-Luc vector |
| L1D6-5’UTR-sense-pro-R2 (MluI) | **ACGCG**TCTTGCTTGTTGTCTTCTTTCTC |  |
| L1D7-5’UTR-pro-F1 | CCAGAAATCTGAGTCCAACAGCC | Primers for first round of PCR amplification based on Sscrofa11.1:chr15:25442410-25444511:-(L1D7) |
| L1D7-5’UTR-pro-R1 | TGTCTGACTGCAGAGATCTGTTT |  |
| L1D7-5’UTR-sense-pro-F2 (Kpn l) | **GGTACC**TGCCCTGCATTAAAAGTCTCT | Primers for second round of PCR amplification for 5'UTR fragment to construct pGL3-L1D7-5'UTR-sense-Luc vector |
| L1D7-5’UTR-sense-pro-R2 (MluI) | **ACGCGT**CTTGCTTGTTGTCTTCTTTCTC |  |
| L1D7-5’UTR-antisense-pro-F2 (MluI) | **ACGCGT**TGCCCTGCATTAAAAGTCTCT | Primers for second round of PCR amplification for 5'UTR fragment to construct pGL3-L1D7-5'UTR-antisense-Luc vector |
| L1D7-5’UTR-antisense-pro-R2 (Kpn l) | **GGTACC**CTTGCTTGTTGTCTTCTTTCTCCTG |  |
| mL1-5’UTR-pro-F1 | TTCTCTGAGTTTCTCTACCCTGTT | Primers for first round of PCR amplification based on GRCm38:chr4:53668350-53670248:+(mL1) |
| mL1-5’UTR-pro-R1 | GTTCCCTGGACTGGGCGAA |  |
| mL1-5’UTR-sense-pro-F2 (Kpn l) | **GGTACC**TAATCACAAAACCCCACATCC | Primers for second round of PCR amplification for 5'UTR fragment to construct pGL3-mL1-5'UTR-sense-Luc vector |
| mL1-5’UTR-sense-pro-R2 (MluI) | **ACGCGT**TAAGATTCCTCCGTTTACCTT |  |
| hL1.3-5’UTR-pro-F1 | ACCTCTAATCCCAGCACTTTCG | Primers for first round of PCR amplification based on GRCh38:chr16:33957524-33958684:-(hL1.3) |
| hL1-5’UTR-pro-R1 | TTGAATGTCCTCCCGTAGCTC |  |
| hL1.3-5’UTR-sense-pro-F2 (MluI) | **ACGCGT**TCAGGAGTTCAAGACCAG | Primers for second round of PCR amplification for 5'UTR fragment to construct pGL3-hL1.3-5'UTR-sense-Luc vector |
| hL1.3-5’UTR-sense-pro-R2 (SmalI) | **CCCGGG**CTTTGTGGTTTTATCTACTTTTGG |  |
| hL1M-5’UTR-pro-F1 | GCCAAGGTATTTTAGATCCTCGT | Primers for first round of PCR amplification based on GRCh38:chrX:142477755-142478930:+(hL1M) |
| hL1-5’UTR-pro-R1 | TTGAATGTCCTCCCGTAGCTC |  |
| hL1M-5’UTR-sense-pro-F2 (MluI) | **ACGCGT**GGAGCTTGTTAGAAATACAGCA | Primers for second round of PCR amplification for 5'UTR fragment to construct pGL3-hL1M-5'UTR-sense-Luc vector |
| hL1M-5’UTR-sense-pro-R2 (SmalI) | **CCCGGG**CTTTGTGGTTTTATCTACTTTTGG |  |
| ERV6A-LTR-pro-F1 | TGGCAAGATTACATTCCTCCCT | Primers for first round of PCR amplification based on Sscrofa11.1:chr12:28221336-28222215:+ (ERV6A) |
| ERV6A-LTR-pro-R1 | CGCATCACCGAAAACAGACA |  |
| ERV6A-LTR-sense-pro-F2 (Kpn l) | **GGTACC**TGAAAGGATGAAAATGCAACCT | Primers for second round of PCR amplification for LTR fragment to construct pGL3-ERV6A-LTR-sense-Luc vector |
| ERV6A-LTR-sense-pro-R2 (MluI) | **ACGCGT**TGAAAGGCCAGTCGAGTGAA |  |
| ERV6A-LTR-antisense-pro-F2 (MluI) | **ACGCGT**TGAAAGGATGAAAATGCAACCT | Primers for second round of PCR amplification for LTR fragment to construct pGL3-ERV6A-LTR-antisense-Luc vector |
| ERV6A-LTR-antisense-pro-R2 (Kpn l) | **GGTACC**TGAAAGGCCAGTCGAGTGAA |  |
| ERV6B-LTR-pro-F1 | TTTCACCTGCCTGTCACTTC | Primers for first round of PCR amplification based on Sscrofa11.1:chr9:138903642-138904481:- (ERV6B) |
| ERV6B-LTR-pro-R1 | TCCCTTTACCTCCAAGTCGG |  |
| ERV6B-LTR-sense-pro-F2 (Kpn l) | **GGTACC**TGAAAGGATGAAAATGCAACCT | Primers for second round of PCR amplification for LTR fragment to construct pGL3-ERV6B-LTR-sense-Luc vector |
| ERV6B-LTR-sense-pro-R2 (MluI) | **ACGCGT**TGAAAGGCCAGTAAAAGAACAA |  |
| ERV6B-LTR-antisense-pro-F2 (MluI) | **ACGCGT**TGAAAGGATGAAAATGCAACCT | Primers for second round of PCR amplification for LTR fragment to construct pGL3-ERV6B-LTR-antisense-Luc vector |
| ERV6B-LTR-antisense-pro-R2 (Kpn l) | **GGTACC**TGAAAGGCCAGTAAAAGAACAA |  |

**Table S7. Primers for retrotransposon assay of L1**

| **Primer name** | **Primer sequence** | **Fragment be amplified** |
| --- | --- | --- |
| L1D1-F | GTTTCCTATTCCAAATACCGAA | L1D1 fragment from genome |
| L1D1-R | TCATCCTCTGTACTAGCCAAC |  |
| CMV-F (KpnI) | **GGTACC**ATAGTAATCAATTACGGGGTCAT | CMV fragment from pEGFP-N1 vector |
| CMV-F (KpnI) | **GGTACC**GCTTATATAGACCTCCCACCG |  |
| L1D1-5’UTR-F (KpnI) | **GGTACC**CTGTGGGGTAAATTAGTATGG | 5’UTR fragment from the L1D1 fragment |
| L1D1-5’UTR-R (KpnI) | **GGTACC**TCTTGCTTGTTGTCTTCTTTCTC |  |
| L1D1-ORF1-F (NheI) | **GCTAGC**GCCACCATGAAGAAGCTGAGAAACCA | ORF1 fragment from L1D1 fragment |
| L1D1-ORF1-R | TTAGAGAAGCCCTTTCAATATTT |  |
| pIGR-F | *TTGAAAGGGCTTCTCTAA*ACCAAAAAGAAAGGAAGGAAAGGG | pIGR fragment from L1D1 fragment |
| pIGR-R | *ATTTATTGCCATGGTGGC*TTGATCCTCGTGTTCCAGTTGA |  |
| hIGR-F | *TTGAAAGGGCTTCTCTAA*AGACCATCAAGACTAGGAAG | hIGR fragment from hL1 vector |
| hIGR-R | *ATTTATTGCCATGGTGGC*TATGATGTTAGCTGGTGATTTTG |  |
| L1D1-ORF2-F | GCCACCATGGCAATAAATAATCATCTATCAAT | ORF2 fragment from L1D1 fragment |
| L1D1-ORF2-R (FseI, NruI) | **GGCCGGCCTCGCGA**TTAGATTCCAGTTATAAGTGATGT |  |
| p3’UTR-F (FseI) | **GGCCGGCC**TATCCAGCACAAATGAACATCT | p3’UTR fragment from L1D1 fragment |
| p3’UTR-R (FseI) | **GGCCGGCC**CAAGGGGGTCAGGTTATCCTT |  |
| h3’UTR-F (FseI) | **GGCCGGCC**ACAATGAGATCACATGGACAC | h3’UTR fragment from hL1 vector |
| h3’UTR-R (FseI) | **GGCCGGCC**GGTTAGTTACATATGTATACATGTG |  |

**Table S8. Primers for detection the expression of retrotransposons by RT-qPCR**

| **Primer name** | **Primer sequence** | **Introduction of primers** | |
| --- | --- | --- | --- |
| L1D-sense-exp-RT | TTCCATGTCCTGGCTATTGTGA | Reverse transcription primer for L1D sense expression detection |  |
| L1D-ORF1-exp-F | GAACAGCAATGCAGATACCCTC | Primers for sense expression detection of L1D ORF1 |  |
| L1D-ORF1-exp-R | TTCGGTTTTCTGTCTGTTGACCA |  |  |
| L1D-ORF2-exp-F | CAGAATCGCCAAAGCAATCCTGA | Primers for sense expression detection of L1D ORF2 |  |
| L1D-ORF2-exp-R | TTCCCATTTGATGTTCTTGCCT |  |  |
| L1D-antisense-exp-RT | GCCCTCTCCCACAAACACAAC | Reverse transcription primer for L1D antisense expression detection |  |
| L1D-5'UTR-exp-F | GGCAGAGGAACCTAAACTCCA | Primers for antisense expression detection of L1D1 5’UTR |  |
| L1D-5'UTR-exp-R | TCCGTACTCCGACTTACTGCT |  |  |
| ERV6-sense-exp-RT | CTTCGACTTTCACAGCCGTTG | Reverse transcription primer for ERV6 sense expression detection |  |
| ERV6-gag-exp-F | TCTCACCAGCCTACTTGGGA | Primers for sense expression detection of ERV6 *gag* |  |
| ERV6-gag-exp-R | ACCTTCAGCCGTGTTGTAGTCC |  |  |
| ERV6-pol-exp-F | GACCCTCCTCCAGTACGTG | Primers for sense expression detection of ERV6 *pol* |  |
| ERV6-pol-exp-R | TACAGTTTTCTTCCGTGCCTC |  |  |
| ERV6-env-exp-F | AATTGACCACAGAGACTGGCAT | Primers for sense expression detection of ERV6 *env* |  |
| ERV6-env-exp-R | AGCGCCTTGAGCCTAGAGAAC |  |  |
| ERV6- antisense-exp-RT | TGAAAGGATGAAAATGCAAC | Reverse transcription primer for ERV6 antisense expression detection |  |
| ERV6-LTR-exp-F | TGAACCCCATAAAAGCTGTCC | Primers for antisense expression detection of ERV6 LTR |  |
| ERV6-LTR-exp-R | GAAGAGGCGACACCCCAAAT |  |  |
| SINEA-sense-exp-RT | CATATGGAGGTTCCCAGGCTA | Reverse transcription primer for SINEA sense expression detection |  |
| SINEA-antisense-exp-RT | AGTGGTTAACGAATCCGACT | Reverse transcription primer for SINEA antisense expression detection |  |
| SINEA-exp-F | CTTGCTCAGTGGGTTAACGAT | Primers for sense/antisense expression detection of SINEA |  |
| SINEA-exp-R | AGCTGTAGCCACCGGCCTA |  |  |
| GAPDH-exp-RT | AGTGTAGCCCAGGATGCCCTT | Reverse transcription primer for *GAPDH* expression detection |  |
| GAPDH-exp-F | CACGGTCCATGCCATCACT | Primers for expression detection of *GAPDH* |  |
| GAPDH-exp-R | GCAGGTCAGATCCACAACC |  |  |


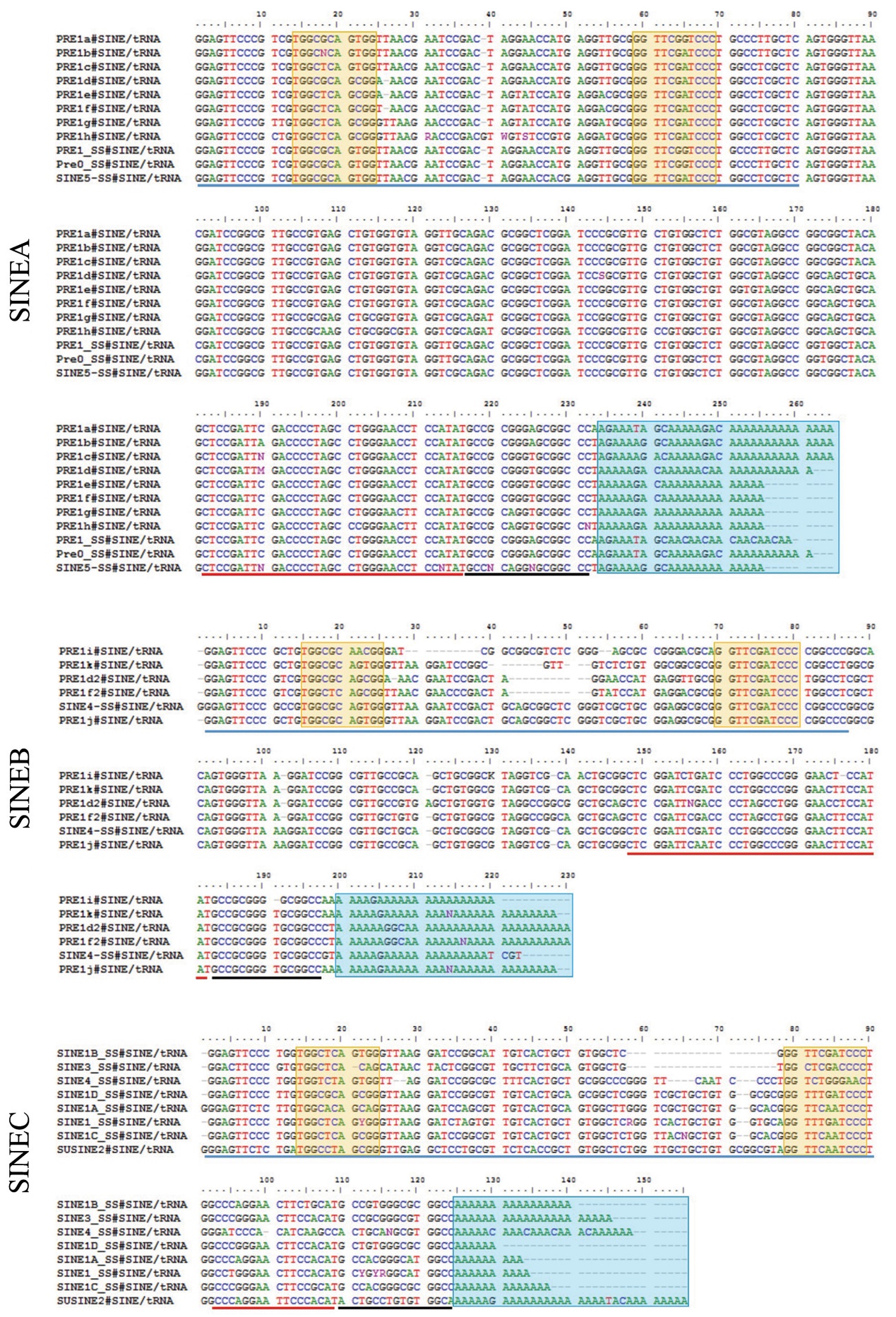


**Fig. S1.** Comparison and reclassification of SINE retrotransposons derived from tRNA in Repbase libraries by sequence alignment.


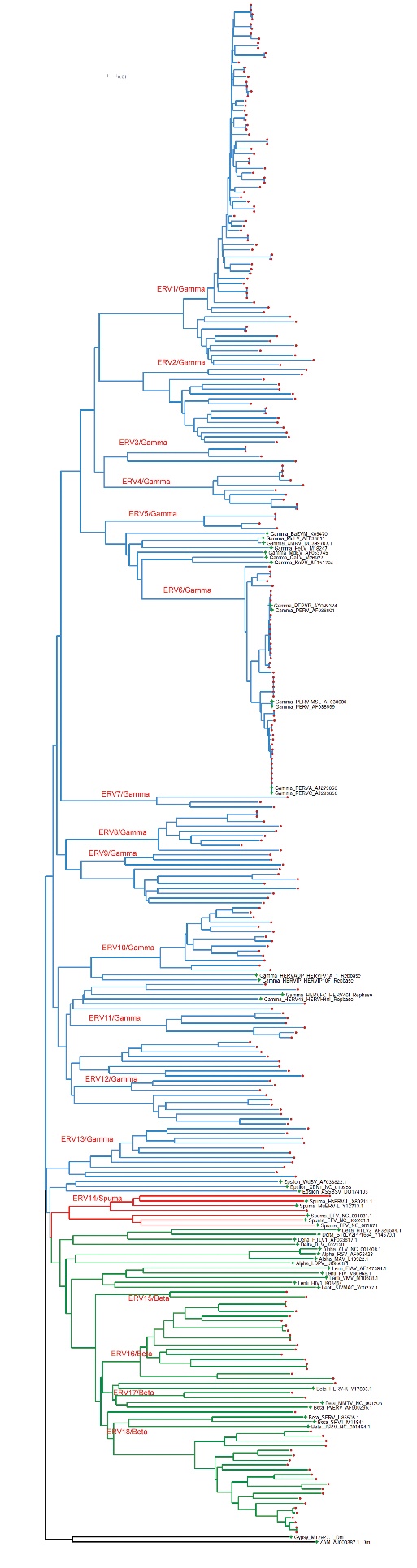


**Fig. S2.** ERVs were classified into three classes based on the Neighbor-Joining phylogenetic tree: thirteen as gamma retroviruses of class I (ERV1-ERV13), and three as beta retroviruses of class II (ERV15-ERV18), one as spuma of class III (ERV14)


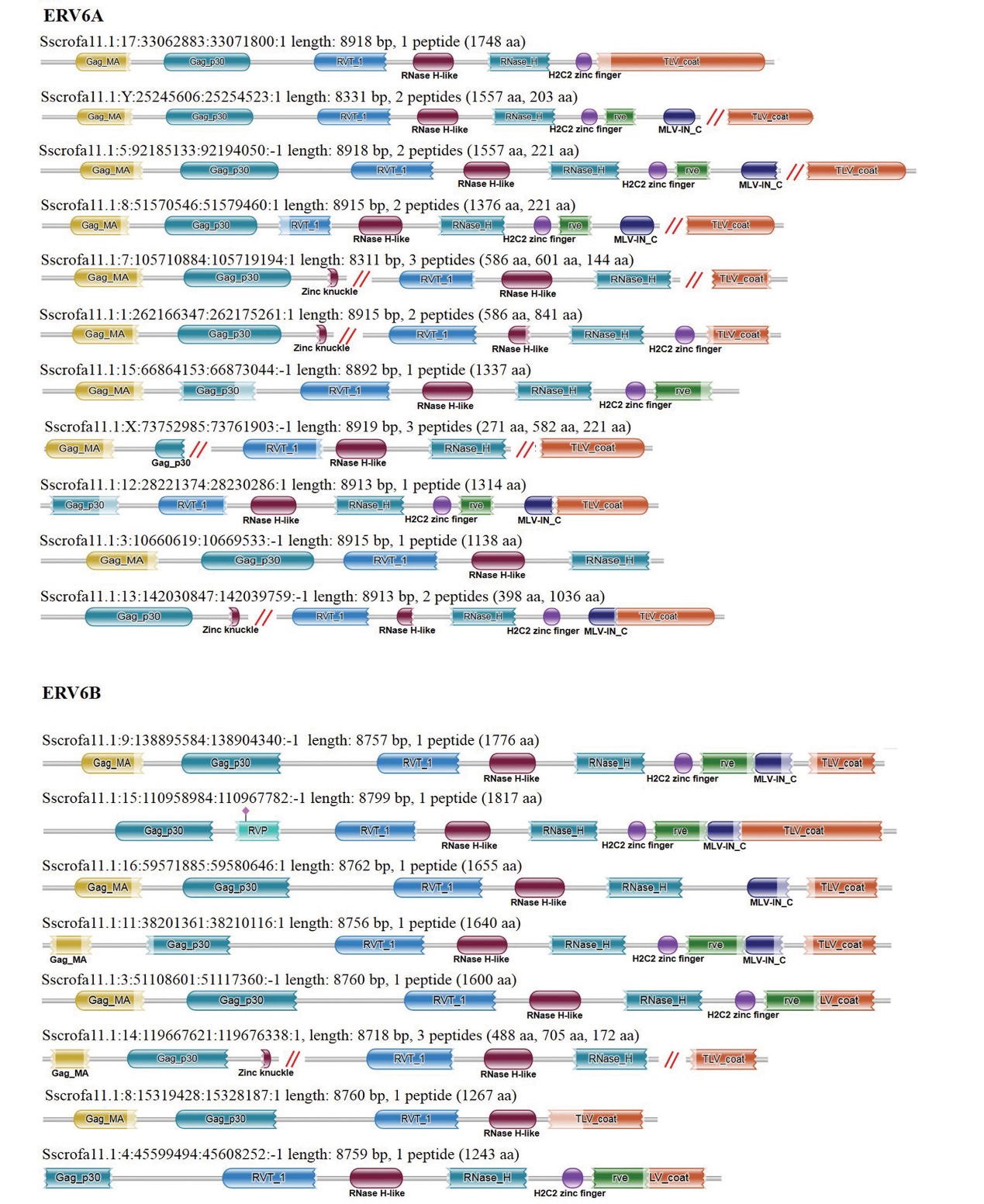


**Fig. S3.** Schematic of the protein structures of full-length ERV6 members in pig genome. Gag_MA: Matrix protein (MA), p15; Gag_p30: Gag P30 core shell protein; RVT_1: Reverse transcriptase (RNA-dependent DNA polymerase); RNase H-like: RNase H-like domain found in reverse transcriptase; rve: Integrase core domain; MLV-IN_C: Murine leukemia virus (MLV) integrase (IN) C-terminal domain; TLV_coat: ENV polyprotein (coat polyprotein); RVP: Retroviral aspartyl protease.


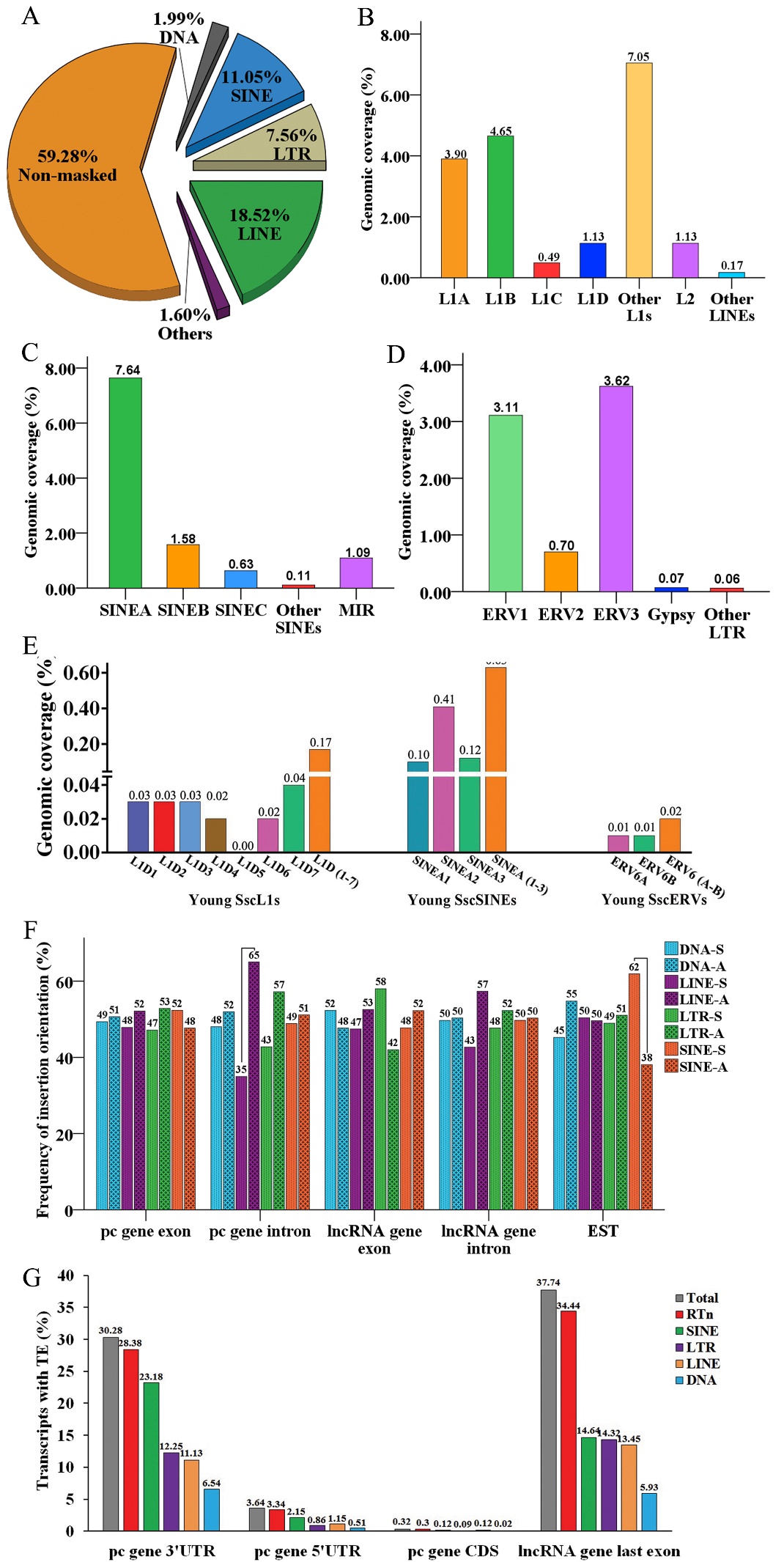


Fig. S4 Retrotransposon distribution in pig genome and the impact on genes.

(A) Genomic coverage of retrotransposon types (LINEs, LTRs, SINEs) in the pig genome. (B) Genomic coverage of pig-specific L1 families (L1A–D). (C) Genomic coverages of pig-specific SINE families (SINEA–C). (D) Genomic coverage of pig-specific ERV classes (ERVI–III). (E) Genomic coverage of youngest retrotransposon subfamilies (L1D1–7, SINEA1–3, and ERV6) in the pig genome. (F) Insertion orientation frequencies in of introns and exons protein coding and lncRNA genes; the bias of insertion orientation was detected by the χ^2^ test, and p<0.05 was considered a significant difference. (G) Distribution bias of retrotransposons in mRNAs and lncRNAs.


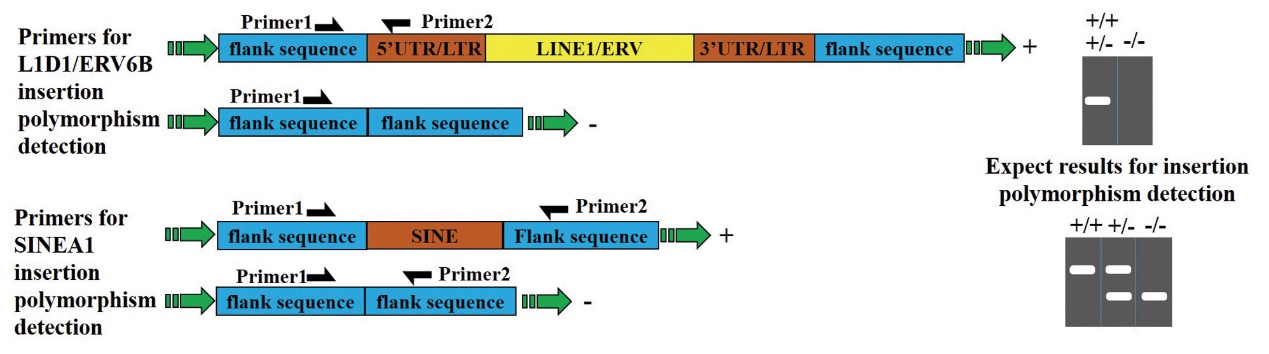


**Fig. S5.** Primers designed for youngest retrotransposons insertion polymorphism detection. For L1D1 and ERV6B, we designed one primer in its flank region and another in their 5’UTR/LTR. For SINEA1, the primers were designed in its flank regions, which span the SINEA1 insertion. And there are three condition: homozygous insertion (+/+), heterozygosity insertion (+/-) and no insertion (-/-). Expected test results for L1 and ERV will appear one band (+/+, +/-) and no band (-/-), for SINE will appear one lager band (+/+), two band (-/-) and one small band (-/-).
